# Adapted Mindfulness Training for Interoception and Adherence to the DASH Diet: A Phase 2 Randomized Clinical Trial

**DOI:** 10.1001/jamanetworkopen.2023.39243

**Published:** 2023-11-02

**Authors:** Eric B. Loucks, Ian M. Kronish, Frances B. Saadeh, Matthew M. Scarpaci, Jeffrey A. Proulx, Roee Gutman, Willoughby B. Britton, Zev Schuman-Olivier

**Affiliations:** 1 Department of Epidemiology, Brown University School of Public Health, Providence, Rhode Island; 2 Department of Behavioral Sciences, Brown University School of Public Health, Providence, Rhode Island; 3 Mindfulness Center at Brown University, Providence, Rhode Island; 4 Center for Behavioral Cardiovascular Health, Columbia University Irving Medical Center, New York, New York; 5 Hassenfeld Child Health Innovation Institute, Brown University School of Public Health, Rhode Island; 6 Department of Biostatistics, Brown University School of Public Health, Providence, Rhode Island; 7 Department of Psychiatry and Human Behavior, The Warren Alpert Medical School of Brown University, Providence, Rhode Island; 8 Cambridge Health Alliance, Cambridge, Massachusetts; 9 Department of Psychiatry, Harvard Medical School, Boston, Massachusetts; 10 Center for Mindfulness and Compassion, Cambridge, Massachusetts

## Abstract

**Importance:**

Hypertension is a major cause of cardiovascular disease, and although the Dietary Approaches to Stop Hypertension (DASH) diet lowers blood pressure (BP), adherence is typically low. Mindfulness training adapted to improving health behaviors that lower BP could improve DASH adherence, in part through improved interoceptive awareness relevant to dietary consumption.

**Objective:**

To evaluate the effects of the Mindfulness-Based Blood Pressure Reduction (MB-BP) program on interoceptive awareness and DASH adherence.

**Design, Setting, and Participants:**

Parallel-group, phase 2, sequentially preregistered randomized clinical trials were conducted from June 1, 2017, to November 30, 2020. Follow-up was 6 months. Participants with elevated unattended office BP (≥120/80 mm Hg) were recruited from the population near Providence, Rhode Island. Of 348 participants assessed for eligibility, 67 did not meet inclusion criteria, 17 declined, and 63 did not enroll prior to study end date. In total, 201 participants were randomly assigned, 101 to the MB-BP program and 100 to the enhanced usual care control group, with 24 (11.9%) unavailable for follow-up. Outcome assessors and the data analyst were blinded to group allocation. Analyses were performed using intention-to-treat principles from June 1, 2022, to August 30, 2023.

**Interventions:**

The 8-week MB-BP program was adapted for elevated BP, including personalized feedback, education, and mindfulness training directed to hypertension risk factors. Both MB-BP and control groups received home BP monitoring devices with instructions and options for referral to primary care physicians. The control group also received educational brochures on controlling high BP.

**Main Outcomes and Measures:**

The primary outcome was Multidimensional Assessment of Interoceptive Awareness (MAIA) questionnaire scores (range 0-5, with higher scores indicating greater interoceptive awareness), and the secondary outcome was DASH adherence scores assessed via a 163-item Food Frequency Questionnaire (range 0-11, with higher scores indicating improved DASH adherence), all compared using regression analyses.

**Results:**

Among 201 participants, 118 (58.7%) were female, 163 (81.1%) were non-Hispanic White, and the mean (SD) age was 60.0 (12.2) years. The MB-BP program increased the MAIA score by 0.54 points (95% CI, 0.35-0.74 points; *P* < .001; Cohen *d* = 0.45) at 6 months vs control. In participants with poor baseline DASH adherence, the MB-BP program also significantly increased the DASH score by 0.62 points (95% CI, 0.13-1.11 points; *P* = .01; Cohen *d* = 0.71) at 6 months vs controls. The intervention was also associated with a 0.34-point improvement in the DASH diet score in all MB-BP participants from baseline (95% CI, 0.09-0.59 points; *P* = .01; Cohen *d* = 0.27), while the control group showed a −0.04 point change in DASH diet score from baseline to 6 months (95% CI, −0.31 to 0.24 points; *P* = .78; Cohen *d* = −0.03).

**Conclusions and Relevance:**

A mindfulness program adapted to improving health behaviors to lower BP improved interoceptive awareness and DASH adherence. The MB-BP program could support DASH dietary adherence in adults with elevated BP.

**Clinical Trial Registration:**

ClinicalTrials.gov Identifiers: NCT03859076 and NCT03256890

## Key Points

**Question:**

Can an adapted mindfulness training program improve interoception and adherence to the Dietary Approaches to Stop Hypertension (DASH) diet?

**Findings:**

This randomized clinical trial of 201 participants showed that by 6 months’ follow-up, the Mindfulness-Based Blood Pressure Reduction (MB-BP) program improved the Multidimensional Assessment of Interoceptive Awareness score by a statistically significant 0.54 points, and the DASH score by a significant 0.62 points, compared with control.

**Meaning:**

The results suggest that the MB-BP program may improve eating patterns, one of the primary drivers of hypertension.

## Teaser

This randomized clinical trial assesses the effects of a Mindfulness-Based Blood Pressure Reduction program on interoceptive awareness and adherence to the DASH (Dietary Approaches to Stop Hypertension) diet among adults with elevated unattended office blood pressure.

## Introduction

Hypertension is a primary cause of cardiovascular disease, which is the major cause of mortality in the US and worldwide.^1^ In the United States, 47% of adults have hypertension, and of them, less than 25% are controlled.^2^ Evidence shows that dietary patterns that emphasize vegetables, fruit, whole grains, lean meats, nuts and legumes, while limiting consumption of saturated fat, red and processed meats, sweets, added sugars, and sodium, can reduce blood pressure (BP) and incident cardiovascular disease.^3,4^ While several dietary patterns are known to improve BP, evidence suggests that the Dietary Approaches to Stop Hypertension (DASH) diet is particularly effective, outperforming the Mediterranean diet and caloric restriction.^3^

Mindfulness interventions have been tested in many diet studies, but often focus on eating disorders^5^ or weight loss.^6^ To our knowledge, no adequately powered randomized clinical trials (RCTs) have investigated the effects of mindfulness training on dietary patterns known to influence hypertension, such as the DASH diet.^6,7^ In particular, the Mindfulness-Based Blood Pressure Reduction (MB-BP) program was developed to train participants in mindfulness practices applied to their relationships with modifiable determinants of BP, including the DASH diet.^8,9,10^ No RCT has yet evaluated the impacts of MB-BP on diet or plausible mechanisms, such as self-awareness.

Self-awareness can be defined as awareness of one’s thoughts, emotions, and physical sensations. One key aspect of self-awareness is interoceptive awareness, which is defined as the conscious level of interoception with its multiple dimensions that are accessible to self-report.^11^ Interoception is the process of sensing, interpreting, and integrating signals originating from inside the body, including processes of interoceptive regulation.^12^ Within the context of dietary behavior and BP regulation, interoceptive awareness can be the conscious experience arising from the attention, appraisal, integration, and regulation of internal sensations related to the physiological condition of the body, such as hunger and satiety cues,^13^ and noticing how different foods make participants feel.^6^

We hypothesized that interoceptive awareness is a mechanism by which MB-BP influences DASH dietary pattern, and we preregistered interoceptive awareness as the primary outcome for this RCT on ClinicalTrials.gov. The primary objective was to evaluate the effects of MB-BP on interoceptive awareness. Secondary objectives, also preregistered on ClinicalTrials.gov, assessed whether the MB-BP impacts DASH adherence.

## Methods

### Study Design, Setting, and Participants

The MB-BP study was a parallel-group phase 2 RCT comparing group-based mindfulness meditation training adapted to improving health behaviors that lower BP vs an enhanced usual care control. Participants were recruited and enrolled from June 1, 2017, to November 30, 2020, through advertising in local communities and referrals from clinicians. The study protocol was approved by the Brown University institutional review board. The trial was registered with ClinicalTrials.gov at the onset of participant recruitment (applied in July 2017, approved in August 2017) and prior to outcome assessment in participants. Participants provided written informed consent. Data were not examined prior to registration. This study followed the Consolidated Standards of Reporting Trials (CONSORT) guideline for RCT reporting.^14^

Inclusion criteria were English-speaking adults (≥18 years of age) with elevated unattended office BP (ie, systolic blood pressure [SBP] ≥120 mm Hg or diastolic BP [DBP] ≥80 mm Hg).^15^ Exclusion criteria were current regular meditation practice (>1 per week); serious medical illness precluding class attendance; current substance use disorder, suicidal ideation, or eating disorder; and history of bipolar or psychotic disorders or self-injurious behaviors. The trial protocol is available in Supplement 1.

This study combined 2 preregistered RCTs with identical methods that were part of a larger study, described elsewhere.^10^ Specifically, the study received a National Institutes of Health UH2/UH3 cooperative agreement that funded 3 years (UH2), followed by an application for 2 additional years of funding (UH3). During the UH2 phase, we conducted a single-group pilot MB-BP trial, reported elsewhere.^9^ We were encouraged to shift from a single-group trial to an RCT before the UH3 phase, which we did, preregistering the trial on ClinicalTrials.gov. That trial identified DASH diet and SBP at 6 months’ follow-up as the primary outcomes. The SBP findings are reported elsewhere.^10^ For the UH3 phase, we were required to create a new ClinicalTrials.gov registration focused on mechanisms of behavior change. We preregistered interoceptive awareness as the primary outcome, to be consistent with the Science of Behavior Change model.^10^ We planned to combine both data sets for meta-analyses, as the clinical trial methods were identical. Limitations of this approach are described in the present article’s Discussion.

### Blinding and Randomization

**Figure 1.**
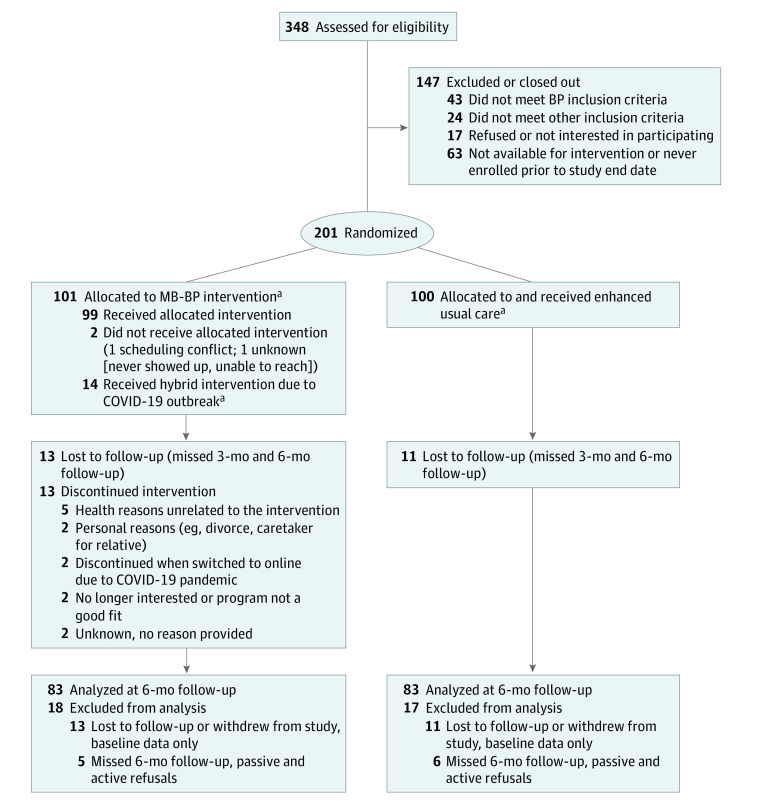
CONSORT Flow Diagram for Participants Randomly Assigned to Mindfulness-Based Blood Pressure Reduction (MB-BP) or Enhanced Usual Care Control ^a^Data substantially affected by the COVID-19 pandemic; online 6-month data collection only for 14 participants in the MB-BP group and 16 participants in the control group. CONSORT indicates Consolidated Standards of Reporting Trials.

Eligible participants who completed baseline assessments were randomly assigned to receive either the MB-BP or control condition (Figure 1). Randomization was stratified by 2 potential determinants of blood pressure at follow-up^16^: sex and BP status (ie, SBP ≥140 mm Hg or DBP ≥90 mm Hg vs other). Randomization occurred 1 week prior to beginning each cycle of group MB-BP classes; participants were informed within 24 hours after randomization. Randomization was performed by a researcher (E.L.) blinded to participant identification using Research Randomizer, version 4.0. The senior project manager (F.S.) notified participants of randomization results. Staff members (including W.N.) conducting follow-up activities of primary outcomes and the data analyst (M.S.), were blinded to group allocation.

### Intervention Descriptions and Theoretical Framework

**Figure 2.**
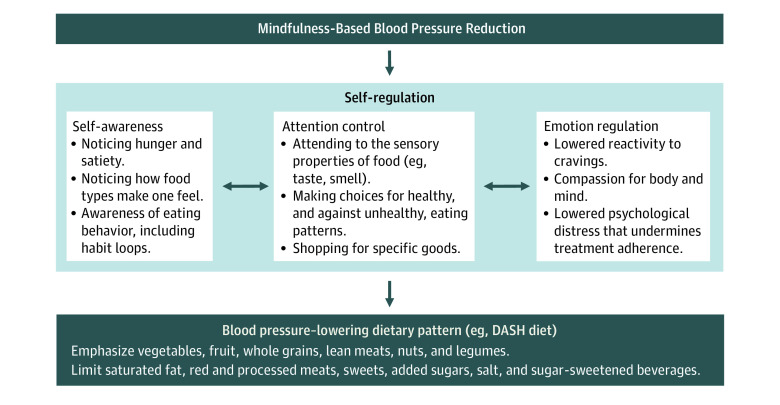
Theoretical Framework of Mechanisms Through Which Mindfulness-Based Blood Pressure Reduction May Influence Dietary Patterns That Lower Blood Pressure

The MB-BP program is based on, and time-matched to, the standardized Mindfulness-Based Stress Reduction (MBSR) program.^9,10^ Both consist of a group orientation session, eight 2.5-hour weekly group sessions, and a 7.5-hour 1-day group session (total of 10 sessions). Recommended home mindfulness practice was at least 45 minutes per day, 6 days per week. Adaptations to the MBSR program that were specific to the MB-BP program are described in eMethods 1 in Supplement 2 and elsewhere.^9,10^ Briefly, MB-BP builds a foundation of mindfulness skills (eg, meditation, yoga, self-awareness, attention control, and emotion regulation) through the MBSR curriculum, and then directs those skills toward participants’ adhering to behaviors that can lower BP, such as the DASH diet, greater physical activity, lowered alcohol consumption, and taking antihypertensive medication (Figure 2).^9,10^ The MB-BP program was led by qualified MBSR instructors with prior expertise in cardiovascular disease etiology, treatment, and prevention. They were further trained and certified to teach the MB-BP program. Classes were provided in person for the first 10 cohorts of 173 persons, whereas the final 2 cohorts of 28 persons began in person and shifted to online via Zoom because of the COVID-19 pandemic. Classes were held in Providence Rhode Island, at Brown University, or a health center in a low income, urban neighborhood. The MB-BP participants also received a home BP device (Omron, model PB786N) and training in home BP monitoring,^17^ and if office BP was 140/90 mm Hg or higher, primary care physicians were notified about the BP results; participants without a primary care physician were offered assistance finding a primary care physician within the constraints of their health insurance.

Participants assigned to the enhanced usual care control group received the same home BP device, training in home BP monitoring, and an option for referral to primary care as participants in the intervention group. They also received an American Heart Association brochure about high BP and how to treat it.^18^ Control group participants did not receive mindfulness training as part of the study.

### Outcomes

The prespecified primary outcome was interoceptive awareness, measured using the validated Multidimensional Assessment of Interoceptive Awareness (MAIA) at 6 months from baseline assessment and randomization.^19,20^ Although the MAIA has 8 dimensions, we used the 8-dimension mean score (range 0-5, with higher scores indicating greater awareness) to avoid issues of multiple statistical testing, similar to prior research that also used the 8-dimension average.^9,21^ Secondary analyses evaluated the effect of the intervention on a 6-dimension mean MAIA score shown in factor analyses to represent 1 construct (with higher scores indicating greater interoceptive awareness),^20,21^ and on each of the 8 MAIA dimensions. Details are given in eMethods 2 in Supplement 2.

Adherence to the DASH diet, the preregistered secondary outcome, was assessed with the Harvard 163-item 2007 Grid Food Frequency Questionnaire,^22^ and coded using methods developed by Folsom et al.^23^ Specifically, the components of the DASH diet index were weighted and summed to a single dietary concordance score (range 0-11, with higher scores indicating greater DASH adherence; details in eMethods 2 in Supplement 2).

Demographics, including age, sex, race and ethnicity, and educational attainment, were assessed using self-report. Race and ethnicity were assessed to evaluate generalizability of the findings; it was self-classified in predefined categories (Asian, Black or African American, Hispanic, Native American, and non-Hispanic White) with the option to self-identify outside the categories. Covariate modifiable determinants of BP included measures of health behaviors and perceived stress, delineated in Table 1, using methods described in eMethods 3 in Supplement 2. Mindfulness was assessed using the validated Five Facet Mindfulness Questionnaire (FFMQ); scores ranged from 39 to 195, with higher scores indicating greater mindfulness levels.^24^ We systematically measured adverse events, including monitoring of serious adverse events and physical injuries a minimum of once a month via online safety checks, detailed elsewhere.^10^

**Table 1.** Baseline Characteristics by Group

Variable	Participants	
	Control group (n = 100)	MB-BP group (n = 101)
Demographics		
Age, mean (SD), y	60.0 (12.3)	61.0 (12.2)
Sex, No. (%)		
Male	42 (42.0)	41 (40.6)
Female	58 (58.0)	60 (59.4)
Race and ethnicity, No. (%)		
Asian	2 (2.0)	3 (3.0)
Black or African American	2 (2.0)	7 (6.9)
Hispanic	5 (5.0)	3 (3.0)
Native American	0	3 (3.0)
Non-Hispanic White	83 (83.0)	80 (79.2)
All other races and ethnicities^a^	5 (5.0)	5 (5.0)
Don’t know or refused	3 (3.0)	3 (3.0)
Highest educational level completed, No. (%)^b^		
High school	12 (12.0)	10 (9.9)
Associate’s degree	5 (5.0)	3 (3.0)
College (4 y)	28 (28.0)	34 (33.7)
Graduate school	43 (43.0)	41 (40.6)
Other	10 (10.0)	10 (9.9)
Blood pressure		
Systolic blood pressure, median (IQR), mm Hg	136.0 (127.0-147.3)	136.8 (129.5-145.0)
Diastolic blood pressure, median (IQR), mm Hg	82.6 (75.3-89.3)	81.9 (76.0-86.8)
First-degree relative with hypertension, No. (%)	86 (86.0)	83 (82.1)
Health behavior		
Physical activity, MET min/wk, median (IQR)	3321 (1602.5-6600.0)	3397 (1854.8-6636.5)
Sedentary activity, sitting min/wk, median (IQR)	2220 (1560.0-3060.0)	1980 (1440.0-3000.0)
DASH diet score^c^	5.1 (1.5)	5.3 (1.3)
BMI, median (IQR)	28.8 (24.8-32.6)	28.8 (25.4-33.0)
Daily alcohol use, median (IQR), drinks/d	0.3 (0.1-1.0)	0.4 (0.1-1.0)
Antihypertensive medication use, No. (%)	70 (70.0)	64 (63.4)
Mindfulness and stress, score, mean (SD)		
Perceived stress scale (PSS-10)^d^	22.3 (8.6)	23.6 (9.0)
Mindfulness (FFMQ)^e^	133.4 (20.8)	132.9 (19.2)
Interoceptive awareness, mean (SD) MAIA score^f^		
8-dimension	2.6 (0.82)	2.5 (0.77)
6-dimension^g^	2.6 (0.96)	2.5 (0.92)
Noticing dimension	2.9 (1.18)	2.9 (1.18)
Not distracting dimension	2.5 (1.06)	2.5 (0.99)
Not worrying dimension	3.0 (1.01)	2.7 (0.98)
Attention regulation dimension	2.3 (1.13)	2.2 (1.10)
Emotional awareness dimension	3.1 (1.03)	2.8 (1.18)
Self-regulation dimension	2.5 (1.22)	2.2 (1.09)
Body listening dimension	1.8 (1.25)	1.7 (1.14)
Trusting dimension	3.0 (1.49)	2.8 (1.36)

Abbreviations: BMI, body mass index (calculated as weight in kilograms divided by height in meters squared); DASH, Dietary Approaches to Stop Hypertension; FFMQ, Five Facet Mindfulness Questionnaire; MAIA, Multidimensional Assessment of Interoceptive Awareness; MB-BP, Mindfulness-Based Blood Pressure Reduction; MET, metabolic equivalent of task.

^a^ Participants who stated that they were races or ethnicities other than the described options self-identified as follows: in the MB-BP group, Asian/White mix, South Asian, Jewish, and English/Scottish. In the control group, Cape Verdean, Jewish, and did not further disclose.

^b^ There were 2 participants in the control group missing education data, and 3 participants in the MB-BP group missing education data.

^c^ Components of the DASH diet index were weighted and summed to a single dietary concordance score (range 0-11), with higher scores indicating greater DASH adherence.

^d^ Perceived stress scale (PSS-10) scores range from 0 to 40, with higher scores indicating higher perceived stress.

^e^ FFMQ scores range from 39 to 195, with higher scores indicating greater mindfulness.

^f^ MAIA scores range from 0 to 5, with higher scores indicating interoceptive awareness.

^g^ The 6-dimension mean score excludes “not distracting” and “not worrying dimensions.”

### Statistical Analysis

For statistical power considerations, an RCT of a similar program, Mindfulness-Based College, demonstrated a between-group MAIA scale effect size (SD) of 0.63 (0.83).^25^ Analyses using the *t* statistic and noncentrality parameter, with α (2-tailed) of 0.05 and β of 0.2, a MAIA difference of 0.40 or 0.50, and SD of 0.83 showed sample size requirements of 68 or 45 per group, respectively. With approximately 100 participants in each group, the power was adequate assuming MAIA effect sizes of at least 0.40 and 20% dropout.

We used generalized estimating equations to evaluate the effects of the MB-BP program on outcomes compared with the control program at 3 and 6 months using an identity link and autoregressive covariance structure. Unadjusted analyses were prespecified due to risks for covariate adjustment making models less precise and inducing bias.^16^ Analyses adjusted for the strata used to randomize participants (ie, sex and BP status) can increase statistical power.^16^ We present analyses adjusting for the randomization strata variables, while also providing unadjusted analyses. Because some participants had healthy DASH-consistent diets at baseline and did not need dietary improvement, DASH diet analyses comprised the full sample as well as only participants with poor DASH diet scores of lower than 5.5.^9,23^ Exploratory mediation analyses used methods based on the counterfactual framework, which allows for decomposition of a total effect into direct and indirect effects.^26^

Analyses were performed from June 1, 2022, to August 30, 2023, using intention-to-treat principles. We analyzed all participants with data regardless of whether they completed the MB-BP or control program, detailed elsewhere.^10^ We performed sensitivity analyses using multiple imputation. Predictive mean matching based on 14 baseline variables was used to impute missing SBP values at 6 months, described elsewhere.^10,27^ Fifty imputations were generated, and generalized estimating equations analysis was performed in each. All analyses were performed using SAS, version 9.4 (SAS Institute Inc), except for multiple imputation, which was performed in R, version 4.2.2 (R Foundation for Statistical Computing), using the mice package.^28^ Statistical significance was defined as a 2-sided *P* < .05 or 95% CI excluding 0.

## Results

Among 201 participants, 118 (58.7%) were female and 83 (41.3%) were male; 5 (2.5%) were Asian, 9 (4.5%) were Black or African American, 8 (4.0%) were Hispanic, 3 (1.5%) were Native American, 163 (81.1%) were non-Hispanic White, and 10 (5.0%) were of another race or ethnicity; 146 (72.6%) had a college or graduate degree; and the mean (SD; range) age was 60.0 (12.2; 22.5-84.3) years (Table 1).

The CONSORT flow diagram is shown in Figure 1. In total, 101 participants were randomly assigned to the MB-BP group and 100 participants to the enhanced usual care group. Of 101 participants assigned to the MB-BP group, 99 received the intervention, whereas all 100 assigned to the control group received the enhanced usual care components. Of 101 MB-BP group participants, 84 (83.2%) attended at least 7 of the 10 MB-BP classes. The most common reason for discontinuing the intervention was for 5 participants who experienced health problems unrelated to the course (Figure 2). The numbers of participants unavailable for follow-up across the 2 groups were similar, with 13 MB-BP group participants (12.8%) discontinuing the study vs 11 control group participants (11.0%). The numbers unavailable for follow-up and missingness were not significantly different by age, race and ethnicity, or educational level (eTable 1 in Supplement 2). The MB-BP intervention was taught by 3 certified instructors (including E.L.), teaching 41, 33, and 27 participants, respectively.

**Figure 3.**
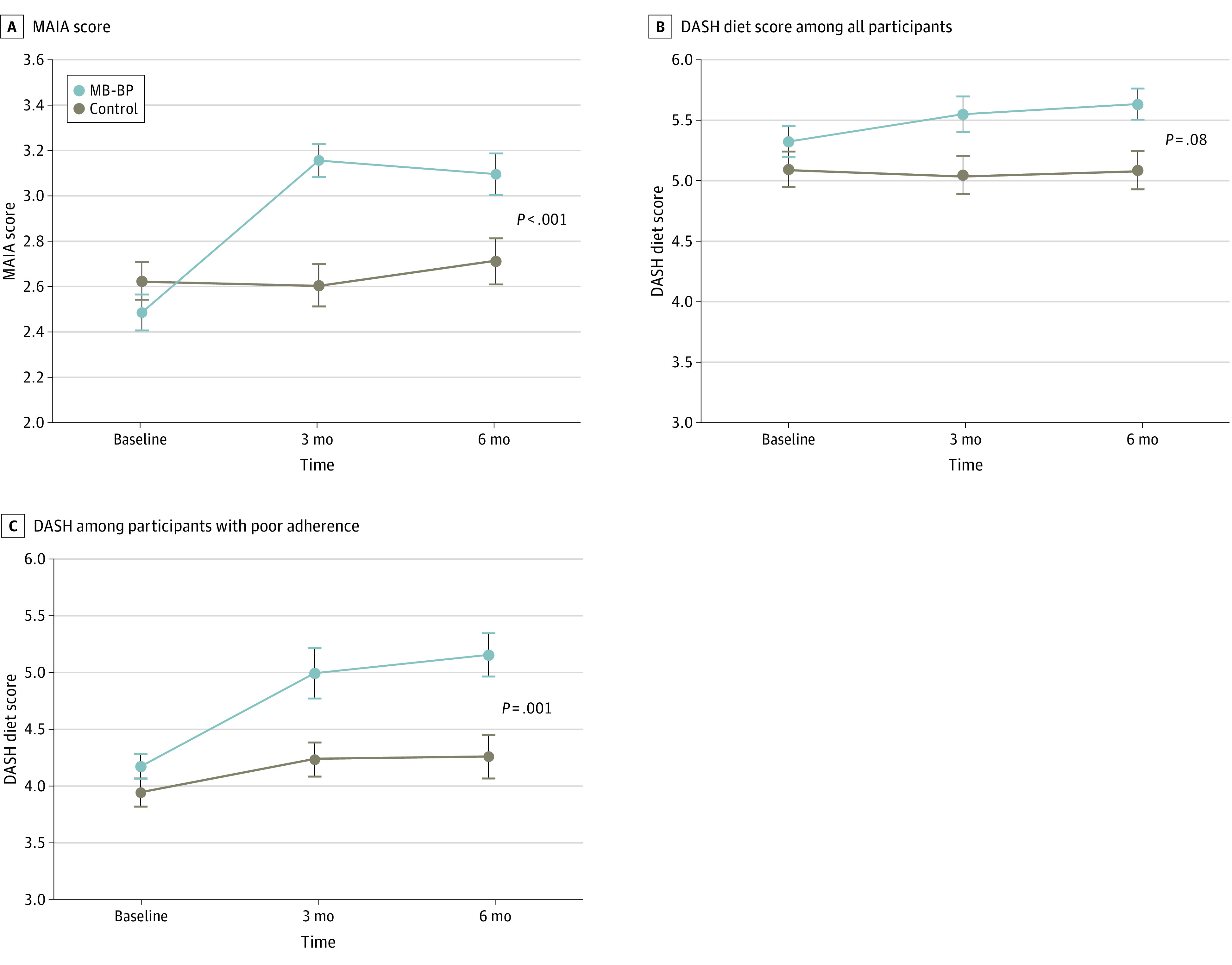
Effects of Mindfulness-Based Blood Pressure Reduction (MB-BP) vs Control on Interoceptive Awareness Assessed via Multidimensional Assessment of Interoceptive Awareness (MAIA) Score, and Dietary Approaches to Stop Hypertension (DASH) Diet Score Among All Participants and Among Participants With Poor DASH Adherence at Baseline Poor DASH adherence was defined as a DASH score lower than 5.5. Error bars represent standard error of the mean.

Primary analyses showed a 0.71-point improvement in the mean interoceptive awareness MAIA score for the MB-BP group at 6 months’ follow-up (95% CI, 0.63-0.88 points; *P* < .001; Cohen *d* = 0.72) compared with baseline. Regression analyses demonstrated a between-group difference of 0.54 points (95% CI, 0.35-0.74 points; *P* < .001; Cohen *d* = 0.45) on the MAIA score at 6 months’ follow-up (Figure 3A). Results for unadjusted analysis (between-group difference, 0.54 points; 95% CI, 0.34-0.74 points; *P* < .001) and multiple imputation (between-group difference, 0.49 points; 95% CI, 0.25-0.72 points; *P* < .001) showed similar between-group differences. Analyses of the 119 participants in the UH3 cohort showed similar findings (between-group difference, 0.64 points; 95% CI, 0.38-0.90 points; *P* < .001). Secondary analyses showed effects of MB-BP vs control on 6 of the 8 MAIA dimensions, with larger effects on body listening, emotional awareness, attention regulation, and self-regulation (Table 2).

**Table 2.** Between-Group Differences in Change of MAIA Scores From Baseline to Follow-Up, for MB-BP vs Control Groups

Outcome	Follow-up time			
	3 Months		6 Months	
	Estimate	*P* value	Estimate	*P* value
MAIA 8-dimension mean	0.68	<.001	0.55	<.001
MAIA 6-dimension mean^a^	0.78	<.001	0.65	<.001
Dimension: noticing	0.35	.02	0.36	.03
Dimension: not distracting	0.36	.02	0.13	.44
Dimension: not worrying	0.19	.15	0.10	.49
Dimension: attention regulation	0.75	<.001	0.60	<.001
Dimension: emotional awareness	0.86	<.001	0.75	<.001
Dimension: self-regulation	1.03	<.001	0.79	<.001
Dimension: body listening	1.08	<.001	0.91	<.001
Dimension: trusting	0.55	.001	0.48	.003

Abbreviations: MAIA, Multidimensional Assessment of Interoceptive Awareness; MB-BP, Mindfulness-Based Blood Pressure Reduction.

^a^ The 6-dimension mean excludes “not distracting” and “not worrying” dimensions.

The intervention was associated with a 0.34-point improvement in the DASH diet score in MB-BP participants from baseline (95% CI, 0.09-0.59 points; *P* = .01; Cohen *d* = 0.27), while the control group showed a −0.04 point change in DASH diet score from baseline to 6 months (95% CI, −0.31 to 0.24 points; *P* = .78; Cohen *d* = −0.03) (Figure 3B). Regression analyses showed a between-group difference of 0.32 points (95% CI, −0.04 to 0.68 points; *P* = .08) at 6 months’ follow-up (Cohen *d* = 0.44). This effect size can be interpreted as equivalent for a participant shifting from a vegetable intake *approaching* recommended levels (2-3 servings) to recommended levels (≥4 servings), or making similar shifts across another component of the DASH score, described in eMethods 2 in Supplement 2.^23^ Restricting the sample to 97 participants with a priori–defined poor baseline DASH diet scores (<5.5) demonstrated an improvement of 0.86 points (95% CI, 0.53-1.19 points; *P* < .001; Cohen *d* = 0.93) in the DASH diet score in the MB-BP group at 6 months vs baseline, and of 0.31 points (95% CI, −0.10 to 0.71 points; *P* = .13; Cohen *d* = 0.30) in the control group. Regression analyses demonstrated a between-group difference of 0.62 points (95% CI, 0.13-1.11 points; *P* = .01; Cohen *d* = 0.71) in MB-BP vs control groups at 6 months (Figure 3C). Results for unadjusted models (between-group difference of 0.63 points; 95% CI, 0.13-1.12 points; *P* = .01), and multiple imputation analysis showed similar findings (between-group difference, 0.59 points; 95% CI, 0.02-1.15 points; *P* = .04).

In an exploratory underpowered mediation analyses to evaluate whether MAIA may mediate effects of MB-BP on the DASH diet score, findings demonstrated partial mediation (31% mediated; *P* = .28) for the MAIA 8-dimension mean score (eTable 2 in Supplement 2). Larger mediation effects were observed for the MAIA domains “attention regulation,” “emotional awareness” and “self-regulation.” With mindfulness as a plausible active component of the program, the FFMQ score demonstrated preliminary evidence of mediation (33% mediated; *P* = .23), as shown in eTable 2 in Supplement 2.

Eight serious adverse events were observed, with 4 in the control group and 4 in the MB-BP group, during 6 months’ follow-up. Adverse events from physical injuries were equal across study groups (8 per group). No serious adverse events or physical injuries were related to study involvement. Details on adverse events are reported elsewhere.^10^

## Discussion

Overall, the findings of this phase 2 RCT showed that the MB-BP program improved interoceptive awareness compared with an enhanced usual care control group through 6 months’ follow-up. There was evidence that MB-BP improved the DASH dietary pattern, with larger effects for participants with poor baseline DASH adherence.

In comparison with other studies, we found similar effects of mindfulness training on the MAIA questionnaire.^9,29^ One notable finding was that 6 of 8 MAIA domains were significantly engaged. A recent confirmatory factor analysis study suggested that the MAIA questionnaire may not represent 8 domains but instead 1, where all 6 domains we engaged with may represent 1 overarching domain of interoception, while the 2 domains we did not engage with may represent other constructs.^20,21^ Our findings are consistent with MB-BP influencing the overall construct of interoception.

The effects of a mindfulness program on the DASH diet have only been assessed in 1 prior RCT,^7^ to our knowledge, specifically, an underpowered pilot RCT that included 25 African American participants with hypertension and cognitive impairment. Those findings suggested that the Mindfulness in Motion and DASH program had no effects on DASH adherence.^7^ The current study supports that MB-BP improves DASH adherence particularly among participants who have poor baseline adherence, which replicates a prior MB-BP single-group clinical trial.^9^ Standardized effect sizes were large compared with baseline (Cohen *d* = 0.93) and medium-large between groups (Cohen d = 0.71) for MB-BP participants with poor baseline DASH adherence in the present study.

Regarding mechanisms, a theoretical framework for how mindfulness training could influence dietary patterns is emerging,^8,9,30^ and is summarized in Figure 1 as 3 domains: self-awareness, attention control, and emotion regulation.^30^ In particular, self-awareness could influence dietary behaviors, such as via (1) noticing hunger and satiety^13^; (2) noticing how different food types makes one feel^6^; and (3) being aware of eating behaviors,^31^ including habits and reward-based learning.^32,33^ Attention control can be applied to dietary behaviors such as via (1) attending to the sensory properties of food (eg, taste, smell, and sight),^34^ (2) making conscious choices for healthy eating patterns and against possibly unconscious unhealthy eating patterns,^35^ and (3) shopping for specific (eg, health-promoting) foods.^35^ Emotion regulation can be applied to dietary behaviors, such as via (1) decreased reactivity to externally and internally initialized cravings,^35^ (2) self-kindness and compassion for one’s body and mind,^31^ and (3) lowering psychological distress, which can undermine treatment adherence.^36^ Recent mechanistic findings from the MB-BP study, using diffusion tensor magnetic resonance imaging, demonstrated that regions of the brain typically damaged by hypertension had either significantly greater connectivity via improved mean diffusivity in the right fornix, or significantly lessened damage by lower radial diffusivity in the left cingulum, in MB-BP vs control.^37^ The improvements in these regions were correlated with interoceptive awareness measured via the MAIA instrument, further supporting the role of interoception in the effect of MB-BP on hypertension-related well-being.^37^ The results from focus groups and in-depth interviews of MB-BP participants suggested an ordering of mechanisms, where self-awareness often came first, followed by strategies to regulate attention or emotion regulation.^8^ The present study is the first, to our knowledge, that performed mediation analyses to evaluate whether mindfulness training–induced changes in self-awareness translate into changes in dietary patterns, specifically the preregistered primary outcome of interoceptive awareness. Assessing the attention control and emotion regulation pathways were beyond the scope of this study. However, the observed effects of MB-BP on the MAIA domain of attention regulation in this study, which includes elements of attention control, are promising. Furthermore, the effects of MB-BP on depression symptoms and perceived stress reported elsewhere^10,37^ suggest that MB-BP may engage emotion regulation pathways. Mechanistic evidence of MB-BP is emerging.

### Limitations

This study has limitations. The first is a follow-up time of 6 months; the durability of intervention effects are unknown, although our prior single-group study of the MB-BP intervention demonstrated significant before vs after improvements in interoceptive awareness and the DASH diet through 1 year follow-up.^9^ The second limitation is a high proportion of well-educated white participants, which reduced generalizability, but this program may be a viable way of introducing health education in underserved populations in a way more specific to BP than MBSR alone, described by our group elsewhere.^10,38,39^ Development and testing of the program in more diverse cultures and languages is important. Third, this study meta-analyzed data across 2 previously unanalyzed clinical trials that preregistered 3 primary outcomes (SBP, DASH diet score, and MAIA). The SBP outcome is reported elsewhere,^10^ and the present article reports on the other 2 outcomes in detail. Recognizing a Bonferroni correction for 3 outcomes would lower the *P* value threshold to .017 for statistical significance; the between-group comparison *P* values for the MAIA in all participants and DASH diet among participants with poor diet at baseline were below those thresholds. Fourth, using the current study design, it was not possible to determine which components of MB-BP were most important for improving diet and interoceptive awareness. Future studies with dismantling or optimization designs can answer these questions.

## Conclusions

This phase 2 RCT offers evidence that an adapted mindfulness training for participants with elevated BP that targets diet and interoceptive awareness improves both. Given the high burden of hypertension on cardiovascular disease, the MB-BP program may offer an approach to improve self-awareness and adherence to evidence-based determinants of BP, such as the DASH dietary pattern.

## Notes

Supplement 1.
Trial Protocol

Supplement 2. **eMethods 1.** MB-BP Program Description, With Emphasis on Dietary Change Components **eMethods 2.** Detailed Methods on Measurement of Primary Outcome (MAIA) and Secondary Outcome (DASH Diet Adherence) **eMethods 3.** Measurement of Covariates **eTable 1.** Evaluation of Differential Missingness **eTable 2.** Mediation Analyses

Supplement 3.
Data Sharing Statement
