# Supplementary material for: Adapted Mindfulness Training for Interoception and Adherence to the DASH Diet: A Phase 2 Randomized Clinical Trial
Source: JAMA Netw Open. 2023 Nov 2;6(11):e2339243. doi: 10.1001/jamanetworkopen.2023.39243 (PMC10623198; doi:10.1001/jamanetworkopen.2023.39243)
Supplement: Supplement 1. — Trial Protocol [file jamanetwopen-e2339243-s001.pdf]

SUPPLEMENT TRIAL PROTOCOL

Full Study Protocol

FULL PROTOCOL TITLE

Mindfulness-Based Blood Pressure Reduction: Stage 2a RCT

Study Chairman or Principal Investigator:

Eric B. Loucks, PhD

Assistant Professor, Department of Epidemiology

Brown University School of Public Health

Supported by:
The National Center for Complementary and Integrative Health

1UH2AT009145-01 and 5UH3AT009145-04

Study Intervention Provided by:

N/A

Sponsor of IND (IDE):

N/A

A NOTE TO READERS:

This document is to serve as a supporting information file to the *JAMA Internal Medicine* manuscript titled, *Impacts of Adapted Mindfulness Training on Blood Pressure: The MB-BP Study: A Randomized Clinical Trial (RCT)*. The study protocol that is found within the following pages contains the complete and detailed plan (in its original language as it was submitted) for the conduct and analysis of the MB-BP Stage 2a clinical trial that the ethics committee (i.e., Brown University Institutional Review Board (IRB)) approved as well as the official study protocol approved by the funding agency, The National Center for Complementary and Integrative Health (NCCIH) that was used for the trial as part of Grant #5UH3AT009145-04. While the methods of data collection for the Stage 2a RCT remained largely the same across the history of the project, from UH2 to UH3 funding, the formal protocol did evolve. Below we have provided some historical context for the evolution of this trial in an effort to offer readers a transparent audit trail of our study.

The original study protocol that was submitted to the Brown IRB on December 5, 2014 was a pilot study and precursor to the MB-BP Stage 1 Single Arm Clinical Trial funded by NCCIH. In September 2015, the study PI (Loucks) was awarded a five-year NIH UH2/UH3 grant (1 UH2 AT009145-01 entitled "Mindfulness Influences on Self-Regulation: Mental and Physical Health Implications"). With this funding, the pilot study approved by the Brown IRB was amended to match the proposed single arm trial approved by NCCIH. Findings from this clinical trial (NCT02702258) can be found in the PLOS One manuscript published online here; stage 1 study protocol found here.

Then in the Spring of 2017 due to promising stage 1 findings, PI Loucks was given approval by NCCIH to transition ahead of schedule from the stage 1 trial to a two-arm RCT (MB-BP Stage 2a), still under the funding mechanism (NCCIH Grant #1UH2AT009145-01). The study protocol for the UH2 Stage 2a trial was submitted to the Brown University IRB as Amendment #8 on May 15, 2017. Note that the Brown IRB reviews and approves modifications to study protocols through the use of stand-alone modification submission forms and not as revised comprehensive study protocols. In other words, they review only the stand alone study documents being revised rather than the complete study package. That being said, the IRB approved study protocol for the *Mindfulness-Based Blood Pressure Reduction (MB-BP): Stage 2a Single-Arm Clinical Trial* funded under UH2 is the conglomeration of the original submission along with all approved modification submissions included in this report.

In September 2018, the study team was awarded a continuation of the Stage 2a trial funding under Grant #5UH3AT009145-04. With this new funding came additional oversight (i.e., revised Data and Safety Monitoring Protocol (DSMP) and multiple study site visits conducted by an external monitoring agency) as well as the production of a more formalized NCCIH study protocol.

The data collection tools and methods used across the UH2 and UH3 phases of the study remained the same, thus allowing for the merging of datasets for analyses. However, it is important to note that there was a shift in the primary and secondary outcomes named. For this reason, the MB-BP Stage 2a study is registered under two separate clinical trials (i.e., UH2: NCT03256890 and UH3: NCT03859076).

In summary, the UH2 Stage 2a study protocol found in this document is the conglomeration of all IRB modification submissions submitted to the Brown University IRB between May 2017 and

September 2019, while the UH3 Stage 2a protocol is found in this supplement as a stand-alone document.

To assist in navigating, please use the hyperlinked table found on the proceeding pages.

Additional questions can be directed to the study investigator: Eric_Loucks@brown.edu.

Mindfulness-Based Blood Pressure Reduction (MB-BP) Study: Brown University
IRB Submission History and Summary of Protocol Amendments

For additional detail on protocol revisions and/or for a copy of the full IRB amendment submissions, with Study PI Signature, contact the Senior Project Coordinator, Frances_Saadeh@brown.edu or study PI, Eric_Loucks@brown.edu.

IRB Submission #	Submitted	Approved	Stage	Summary of Submission / Protocol Revisions
Original Submission	12/5/14	3/12/15	Pilot	The project was submitted to the Brown IRB in December 2014. The IRB requested three iterations of revisions to the submission prior to granting approval. These revisions were submitted: 1/5/15, 2/10/15, and 2/19/15.
Amendment #1	4/8/15	7/1/15	Pilot	This amendment dated 6/17/15, (originally submitted 4/8/15) includes changing the survey software to Qualtrics, updating the phone screening questionnaire, clarifying the study safety plan, modifying the consent form, adding questionnaires to the baseline survey, and modifying recruitment procedures.
Amendment #2	7/15/15	8/6/15	Pilot	This amendment dated 7/15/15 (revised and re-submitted on 8/5/15) included: The addition of a new participant population (NEFS sub-set) and new recruitment procedures for that population, revisions to the In-person Screening Assessments 1 and 2, revisions to wording of the phone screening questionnaire, addition of a place for staff to note contact information on the safety plan document, adding a version footer to the consent document.
Amendment #3	9/1/15	9/3/15	Stage 1	This amendment dated 9/1/15 included the addition of a funding source (grant #1 UH2 AT009145-01, grant title: Mindfulness Influences on Self-Regulation: Mental and Physical Health Implications), a change in scope of work and related study activities secondary data analyses involving concurrent studies, additional measures and study procedures, and revised informed consent document.
Amendment #4	11/11/15	1/7/16	Stage 1	This amendment (dated: 11/11/2015) requests approval to (a) add compensation, (b) add a NIH safety monitoring plan, (c) update the names and content of the phone screen and assessments,(d) add a 1-year follow-up visit, and (e) update the consent document to incorporate these changes.
Amendment #5	2/26/16	2/26/16	Stage 1	This amendment (dated: 2/26/16) requests approval to allow participants, who are not able to come to the study site in person, the option to participate by online video conferencing.
Amendment #6	4/11/16	5/9/16	Stage 1	This amendment (dated: 4/11/2016) requests approval to (a) change the study title; (b) revise the NCCIH Safety Monitoring Protocol; (c) separate the Baseline and Follow-up Assessments into two visits; (d) change the data collection mode to Qualtrics for Daily Practice Forms and Class questionnaires; (e) revise study recruitment material and use online advertising; (f) revise the in-class worksheets and assessments; and (g) revise the MB-BP Questionnaire, In-Person Assessment, Home Assessment, and Measurement of Mindfulness Practices.

IRB Submission #	Submitted	Approved	Stage	Summary of Submission / Protocol Revisions
Amendment #7	8/9/16	9/13/16	Stage 1	Approval of the amendment (memo dated August 9, 2016) includes the addition of 2 new questions to the Home Baseline Assessment.
Amendment #8	5/15/17	6/12/17	Stage 2a	This amendment (dated 5/15/2017) requests approval to (a) begin Stage 2a (previously called Stage 1b) to conduct the Randomized Controlled Trial with enhanced usual care. Stage 2a comes with changes to the study flow, incentives, screening and eligibility, consent document, phone screener, in-person screener, baseline in-person assessment, baseline-online home assessment, safety protocol, and study recruitment material.
Amendment #9	8/26/17	10/13/17	Stage 2a	This amendment (dated: 8/28/2017) requests approval to (a) add an Infographic Card, (b) add a Recruitment Card, (c) conduct a direct mailing at a partnering provider office, (d) add NIH Administrative Supplement funding to award #004754-001 "Mindfulness Influences on Self-Regulation: Mental and Physical Health Implications," IP #17101586, and (e) update the consent document with Certificate of Confidentiality language.
Amendment #10	11/6/17	1/25/18	Stage 2a and additional follow up for Stage 1	This amendment (dated: 11/6/2017) requests approval to (a) modify the approved focus group discussion questions into a one-on-one qualitative phone interview, (b) re-contact Stage 1 participants for a 2-year in-person assessment, (c) rearrange compensation amounts, (d) add recruitment for and data sharing from the UMass Medical School fMRI study, (e) add the "UMass Medical School fMRI Study Recruitment Talking Points," (f) add a consent addendum for enrolled participants to complete the phone interview, and (g) revise the approved consent to reflect the appropriate changes.
Amendment #11	5/8/18	6/13/18	Stage 2a	This amendment (dated: 5/8/2018) requests approval to (a) drop the 12 month and two year follow-ups for Stage 2a participants only; (b) reduce the number of home blood pressure readings and drop the mandatory introduction session that is part of the course; (c) assist collaborators at UMass Medical School with the initial screening and scheduling processes for their fMRI study; (d) conduct the 9-week mindfulness intervention off site in safe, community-based locations; (e) recruit from the partnership at Lifespan Emergency Department; (f) conduct qualitative interviews with low SES population to explore perceptions of and openness to mindfulness-based interventions; and (g) update consent forms with new format. Also includes approval of revised phone screener (SCR-P, v.3.0 – 5/10/2018)
Amendment #12	8/7/18	10/15/18	Stage 2a	Requested the following: 1) permission to conduct abbreviated 1 year follow-up assessment with the intervention arm of the study; 2) request to use a general release form to video-record mindfulness classes; 3) video-recording; 4) removal of the actigraph device; 5) additional questions for the 6 month follow-up assessment for control participants; 6) updated consent document (v.3.1 – 10/11/18); and 7) consent addendum for already enrolled participants.

IRB Submission #	Submitted	Approved	Stage	Summary of Submission / Protocol Revisions
Amendment #13	12/14/2018	1/3/2019	Stage 2a - UH3	This amendment represents approval of the NCCIH UH3 Study Protocol (v.2.5, December 11, 2018) and DSMP (v.1.0, November 14, 2018).
Amendment #14	4/2/2019	4/11/2019	Stage 2a - UH3	(1) Correction of grammatical and spelling errors on informed consent form (now v.3.3.)<br>(2) Updated UMass MRI Study talking points document to include increase in compensation.<br>(3) New recruitment material for UMass MRI study.
Amendment #15	4/19/9	4/25/19	Stage 2a - UH3	This amendment requests approval to use Today@Brown as a recruitment tool for this study.
Amendment #16	8/15/19	8/21/19	Stage 2a - UH3	This amendment (dated: 8/15/2019) requests approval to modify recruitment advertisements (only to be used at Lifespan) to replace the word "free" with "provided at no cost to qualified participants."
Amendment #17	3/9/20	3/13/20	Stage 2a - UH3	Request to move to remote procedures (i.e, online delivery of MB-BP intervention) due to COVD-19 pandemic / University and statewide shutdowns.
Amendment #18	4/24/2020	5/8/2020	Stage 2a - UH3	Shift to online data collection for subset of in-person measures that are no longer able to be collected due to COVID-19 global pandemic.

Brown University
Research Protections Office
Institutional Review Board
Modification Request

Date of Request: 5/15/17 **Investigator's Name and Title:** Eric Loucks, PhD, Assistant Professor

Study Title: Mindfulness-based Hypertension Therapy Pilot Study (#1412001171)

Original Type of Review: ☐ Exempt ☐ Expedited ☒ Full Board

1) **Provide a brief lay summary of the overall project. Include enough detail to allow the IRB to evaluate the requested change(s) within the context of the overall project.** (Attach summary to this form)

See attached summary

2) **Provide a detailed description of the changes being requested** (Use additional pages, if necessary):

See attached summary

3.) **State the reason (justification) for the requested modification.** (Use additional pages, if necessary):

See attached summary

4.) **What is your assessment of how the changes will affect the overall risk/benefit ratio of the study and the willingness of individuals to participate?**

See attached summary

5.) **Does the requested modification require new documents or changes to the approved consent form or other documents?**

☒ Consent/assent documents (attach revised version with changes highlighted)

☒ New/revised instruments (attach - if revised, highlight changes)

☒ New/revised advertising materials (attach - if revised, highlight changes)

Do you have a conflict of interest on this project according to Brown's policy? ☐**YES** ☒**NO**

If YES, has this conflict been previously disclosed to the IRB? ☐**YES** ☐**NO**

PI signature: [signature] Date: 5/15/17

1. Provide a brief lay summary of the overall project. Include enough detail to allow the IRB to evaluate the requested change(s) within the context of the overall project.

Original lay summary providing broad overview of the project:

The World Health Organization reported that suboptimal blood pressure (BP) is responsible for more than half of cardiovascular disease mortality world-wide. Furthermore, greater than half of those with hypertension have uncontrolled BP. *A 2009 Institute of Medicine report recommended prioritizing research to "Compare the effectiveness of mindfulness-based interventions (e.g. yoga, meditation, deep breathing training) and usual care in treating… cardiovascular risk factors."* Evidence-based mindfulness interventions, including Mindfulness-Based Stress Reduction, may have some effects on blood pressure, where a recent meta-analysis and systematic review of 4 randomized controlled trials demonstrated significant effects, but evidence of heterogeneity in effect sizes. The methodologically highest quality studies had the smallest effect sizes (range 0-5 mmHg). Mindfulness-Based Stress Reduction (MBSR) has been customized to a number of disease processes, such as Mindfulness-Based Cognitive Therapy for patients with recurrent depression, and Mindfulness-Based Relapse Prevention for patients with substance use addictions. Effect sizes have been increased by customizing mindfulness interventions to diseases of interest. The same may be true for hypertension, *however mindfulness interventions customized for prehypertensive/hypertensive patients have never been investigated. Until methodologically rigorous studies to evaluate customized interventions for hypertension are performed, we will not know if the observed preliminary effects of general mindfulness interventions on blood pressure reduction could be much more effective with a tailored approach.* Consequently, we propose to conduct a stage I behavioral therapy intervention study to evaluate whether MBSR customized to prehypertensive and hypertensive patients has the potential to provide clinically relevant reductions in BP. Consequently the specific aims are:

Stage 1a: Therapy Development/Manual Writing

1. To outline and evaluate key novel elements of mindfulness-based hypertension therapy (MBHT), customized from the evidence-based MBSR. *We hypothesize that the most important novel element will be generation of mindfulness skills specifically applied to hypertension risk factors such as diet, physical activity, obesity, alcohol consumption and antihypertensive medication adherence.* This aim will be achieved using (1) focus groups of participants undergoing the MBHT behavioral intervention, (2) discussion with experts (including cardiologists, epidemiologists, mindfulness experts, mindfulness intervention instructors) prior to, and following pilot testing of MBHT in participants, and (3) clinical judgment of the investigators performing the intervention.
2. To determine effectiveness of MBHT on primary outcomes (systolic blood pressure, retention rates, recruitment rates, and adverse effects) and secondary outcomes (hypertension risk factors such as diet, physical activity, obesity, and antihypertensive medication adherence) in hypertension subgroups, specifically participants with (1) prehypertension, (2) controlled hypertension, and (3) uncontrolled hypertension. Initial decisions about the targeted sample based on hypertension status will be made.
3. To develop an MBHT therapist manual and training program, including procedures for training, supervising, and evaluating therapists. Furthermore, acceptable therapist characteristics will be developed. The manual and training program will include themes such as specification of unique and common elements of MBHT *vs.* other interventions, description of interventions excluded from MBHT, and specification of key treatment

parameters such as frequency and duration of treatment, session length, topics addressed, sequence of sessions, as well as therapist adherence and competency measures. The MBHT training will consist of a therapist manual, a formal didactic training seminar, and at least one closely supervised training session.

Stage Ib: Pilot Trial

4. To determine whether a mindfulness-based hypertension therapy (MBHT) intervention, customized from the evidence-based MBSR, has promise to be an effective behavioral therapy for participants with hypertension and/or prehypertension. We will perform a randomized controlled pilot trial for MBHT *vs.* enhanced usual care control. *We hypothesize that MBHT will have adequate recruitment rates (≥10% of prehypertensive/hypertensive participants invited from physicians' offices), fairly low drop out rates (<15%), and medium effect sizes (e.g. 5-10 mmHg systolic BP) for reduction in blood pressure.*

These findings will provide publishable pilot data that will inform future randomized clinical trials that evaluate effects of MBHT on long-term changes in blood pressure *vs.* usual care and active control groups. *If proven effective, MBHT could be offered as a complementary program in the prehypertensive/hypertensive patient population that contributes to over half of the cardiovascular disease mortality world-wide.*

Project update as of May 2017:

The Mindfulness-Based Blood Pressure Reduction (MB-BP) Study, formerly known as Mindfulness-Based Hypertension Therapy (MBHT), is currently in Year 2 of a five year NIH UH2 grant.

We are in the final data collection phase of Stage 1a of the project, which involved a prospective single arm trial during the intervention development phase. All participants under this phase have been enrolled and follow up assessments are being conducted. In total we ran three separate Mindfulness-Based Blood Pressure reduction (MB-BP) intervention courses, with a total sample size of 43 eligible participants who both enrolled and completed the 9-week intervention. We had eight eligible participants enroll in the study who later decided to not complete the course. Assessments will continue through the 12 month follow up period with an estimated stage 1 data collection completion date of October 18, 2017.

The next phase (referenced as Stage 1b above, but now called 'Stage 2a') will be to conduct a Randomized Controlled Trial (RCT) with *enhanced usual care*. The anticipated start date for the RCT intervention is May or June 2017. This modification request (#8) outlines the transition to Stage 2a, including providing details on revisions to the study protocol, informed consent, instruments and measures, and recruitment materials.

The third and final phase of the study will be an RCT with an *active* control (i.e., Mindfulness-Based Stress Reduction - MBSR) as well as ambulatory blood pressure monitoring. ***The current modification request only outlines Stage 2a details and does not deal with the final stage of the study. We will submit a separate IRB modification for this phase of the study.***

2. Provide a detailed description of the changes being requested (Use additional pages, if necessary):

This modification request outlines all of the changes required to transition from Stage 1, a prospective single arm trial, to Stage 2a, a two arm Randomized Controlled Trial (RCT) with enhanced usual care. Summaries of the revisions made to the study protocol as well as to each individual study document are outlined below, while detailed revisions can be found in Attachments 1-8.

Figure 1. Stage 2a Assessment Flow Chart

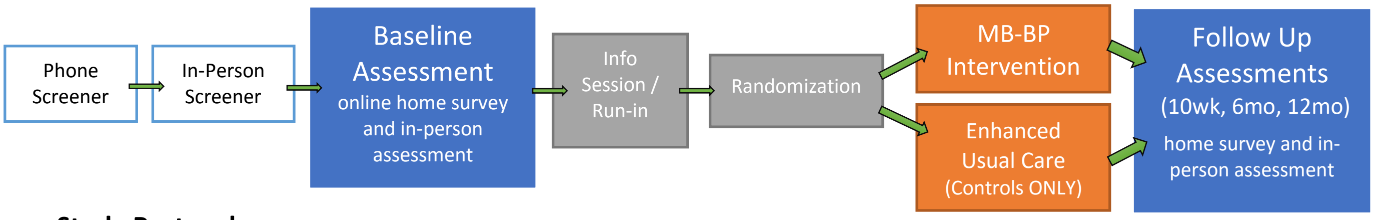

Study Protocol:

- Study Flow – the overall flow of the study has not changed significantly from Stage 1 to Stage 2a. The screening process as well as timing of assessments has remained the same. The most notable changes to the study flow are the addition of an information session that will take place 1-2 weeks prior to the start of the intervention as well as the use of a randomization process that will determine which arm of the trial participants will be assigned to.
    - *NEW* - The information or introduction session, scheduled to take place prior to the randomization process, will serve three purposes: (1) to provide eligible individuals more information about the study, including details about the 9 week mindfulness course; (2) to serve as a run-in process whereby we can gauge participants' level of interest and commitment to the study; and (3) to allow for study equipment (i.e., home BP monitors) to be handed out and explained.
    - *NEW* - The randomization process will be conducted after the information session but before the start of the intervention. Only enrolled, eligible participants who have completed the baseline assessment, attended the information session, and expressed interest in continuing with the study will be included in the randomization process. The randomization procedure will be performed using Research Randomizer (www.randomizer.org) and will be implemented by a research associate not otherwise involved with the study. The research associate will keep group assignments blinded from other study staff who perform participant assessments and/or statistical analyses. Randomization will be stratified by age, gender, BP status, and/or race (depending on numbers in each group).
- Intervention Group – similar to Stage 1, participants randomly assigned to the Intervention group will be invited to take part in the 9 week MB-BP class. The format of the class remains the same, meeting for 2.5 hours weekly as well as taking part in an all-day retreat held on a weekend day sometime between weeks 5 and 6.
- Control Group (enhanced usual care) – participants randomly assigned to the control group will not immediately take part in the 9-week intervention, but instead will participate in the research assessments only. They will be given the opportunity to take a mindfulness class *after* the completion of their 6 month follow up, but their participation in the class is optional. The

control group is designated as 'enhanced usual care' rather than simply 'usual care' for two reasons: (1) the in-person research assessments will serve as a 'screening' for uncontrolled hypertension. Any individuals who exhibit uncontrolled hypertension (i.e., systolic reading of 140 mmHg or greater OR diastolic reading of 90 mmHg or greater) will be notified and resources will be provided (see revised Safety Protocol for details). (2) The other aspect of 'enhanced usual care' is that individuals in both the control and intervention group will be given a wireless blood pressure monitor that can be used to track their blood pressure at home several times throughout the study. It is possible that access to and use of the wireless BP monitor at home will improve participants' BP levels.

- <u>Participant Incentives</u> – we are requesting permission to change the participant incentive schedule and structure. Rather than provide participants with the $100 USD at the end of their final follow up, we would like to provide participants with incentives at each of the follow up assessments. We are proposing $25, $25, and then $50 be given out at the 10wk, 6mo and 12mo follow ups respectively.

- <u>Screening and Eligibility</u> – in the preliminary analyses of Stage 1 data it appears that the greatest decrease in systolic and diastolic blood pressure was seen in individuals with uncontrolled hypertension (i.e., 140/90 mmHg or greater). For this reason, we would like to prioritize recruitment amongst individuals in this group. However, we are not certain at this time if we will be able to fill a full class with only uncontrolled hypertensive individuals. Under this rationale we are proposing the following revisions to our screening process:

    - *REVISED BP ELIGIBILTIY CRITERIA* - Based on the blood pressure readings taken at the in-person screener and at the baseline assessment, an individual will be fall into one of the three BP categories: (1) ELIGIBLE - average BP reading was 140/90 mmHg or greater; (2) WAITLIST – average BP reading was 120/80 mmHg or greater but less than 140/90 mmHg; or (3) INELIGIBLE – average BP reading at either visit was below 120/80 mmHg.

        - Eligible individuals will be enrolled on a first come first serve basis until the class reaches capacity. Any individuals who screen eligible after a class reaches capacity will be invited to take place in future cohorts.

        - If there is still room in a cohort after all uncontrolled hypertensive individuals have been enrolled, then the research staff will reach out to individuals on the wait list.

    - *NEW - BP Medication Changes* – since it can often take an individual up to three to four weeks to respond to changes in blood pressure medication, we would like to collect this information at the time of screening and recommend that individuals who underwent any changes to their blood pressure medication regime wait at least three weeks before completing the Baseline assessment (if timing allows).

Informed Consent (v.2.0) – Appendix 1:

- Updated the consent form to be relevant for the Stage 2a Randomized Controlled Trial (RCT), including adding information on the protocol revisions described above (e.g., changes to participant incentives), new and revised measures, etc.
- Clarified language about eligibility and participant involvement
- For detailed revisions, see the Informed Consent document with track changes

Phone Screener (SCR-P - v.2.0) – Appendix 2:

- No significant changes were made; updated the study summary language to describe the RCT and revised the participant incentive description.
- The actual phone screening questions and criteria did NOT change.
- Removed SQ41b since the dates of the intervention are already preset and therefore asking participant availability is not necessary.
- NO CHANGES MADE TO THE PHONE SAFETY PROTOCOL

In-Person Screener (SCR-I - v.2.0) – Appendix 3:

- No significant changes made; estimated length is the same.
- Added internal variable to note which BP monitor was used
- Updated gender variable to capture individuals who may self-identify as 'other / non-binary'
- Added two new blood pressure medication variables to capture recent changes to blood pressure medication use

Baseline – in-person assessment (v.2.0) – Appendix 4:

- *DROPPED* questions BA4-BA5 – these are internally programmed into Qualtrics.
- *NEW '*BP monitor' variable – added internal variable to note which BP monitor was used
- *REVISED* MEDICATION SECTION - To reduce participant burden we have revised the medication section such that we will only collect information about medications used to treat high blood pressure.
- *DROPPED* 'don't know' and 'prefer not to answer' response options from standardized self-report scales in order to match the original measures. However, participants always have the option of skipping over any questions they do not wish to answer.
- *NEW* Pittsburgh Stress Battery (20 minutes) - This is a battery developed by Richard Jennings et al that has gone through validity/reliability testing. We will be using three components of this stress battery (i.e., Stroop task, mirror tracing task, and arithmetic task) in order to initiate a stress response across multiple domains. Blood pressure and heart rate will be monitored both during and after the stress battery is administered.
- *REVISED* Food Frequency Questionnaire – we updated the food frequency questionnaire from a three page 1991 FFQ to a four page 2007 FFQ. Both are from the Harvard School of Public Health.
- *NEW* Global Health scale
- *MOVED* measures – the below measures were moved from the online home assessment to the in-person assessment. These scales were moved for two reasons: (1) to shorten the online home survey and (2) so that the participant would have non-stressful self-report questionnaires to work on during the 30 minute recovery period following the stress battery described above.
    - Sleep question taken from the Pittsburgh Sleep Quality Index (PSQI)
    - International Physical Activity Questionnaire (IPAQ-long)
    - Mindful Attention Awareness Scale (MAAS)
    - Five Facet Mindfulness Questionnaire (FFMQ)
- *NEW* Heart Beat Detection Task (5 minutes)
- *NEW* 5-Trial Adjusting Delay Task (2-3 minutes)
- *DROPPED* Attention Network Task (ANT) to reduce participant burden; saves 20 minutes of time

Baseline – online home assessment (v.2.0) – Appendix 5:

- Removed BQ1_01 (age) – already captured in the in-person screener
- Collapsed the education variables into one question in order to simplify
- Physical activity was moved to the In Person baseline assessment and the scale used was revised (see BASE-I revisions described above)
- *DROPPED* 'don't know' and 'prefer not to answer' response options from standardized self-report scales in order to match the original measures. Participants always have the option of skipping over any questions they do not wish to answer.
- REPLACED the 10-item Perceived Stress Scale (PSS) with the 14-item PSS
- MOVED the Mindful Attention Awareness Scale (MAAS) and the Five Facet Mindfulness Questionnaire (FFMQ) to the In Person Baseline assessment.
- REPLACED the adverse childhood experiences scale with the following: Childhood Trauma Questionnaire;the Childhood Food Insecurity Scale; and the Childhood Experiences of Care and Abuse Inventory (neglect subscale only).
- NEW – added the Connor Davidson Resilience 10 item scale
- DROPPED the 9 items on blood pressure medication adherence
- MOVED the sleep question to the in person baseline

Safety Protocol (v.2.0) – Appendices 6 and 7:

- Added guidance for situations where participants blood pressure reads above 140/90 mmHg (uncontrolled hypertension). Part of the safety protocol we have added that is new to Stage 2a is sending a letter to a participant's health care provider if he/she screens as having uncontrolled hypertension and if they indicate that his/her provider is unaware of the reading (refer to Appendix 7). If a participant does not have a PCP then we will also offer to assist them with finding one using the RI Department of Health provider search engine found online at: http://www.health.state.ri.us/find/primarycare
- New to Stage 2a is the Heart Beat Detection task (see In-Person Baseline assessment for details). The equipment used for this task is the Kardia Mobile ECG device and it is designed to record, store and transfer single-channel electrocardiogram (ECG) rhythms. Although rare, it is possible that the device will flag a 'possible atrial fibrillation'. We have added a safety protocol for this scenario including consulting the board-certified cardiologist, Dr. Hank Wu, who serves on the study data safety monitoring board.
- Updated the Anxiety and Depressive Symptomology protocol slightly to more accurately reflect the assessment room set up and process.
- Based on the consolation of our study psychiatrist, we modified the suicidal ideation follow up protocol so that we can more accurately assess and respond to immediate threat to self-harm.

Study Recruitment Material – Appendix 8:

- Added our study website to our recruitment material.
- Otherwise the recruitment material has not changed since last approved.

3. State the reason (justification) for the requested amendment:

Please see justifications in Section 2 above.

4. What is your assessment of how the changes will affect the overall risk/benefit ratio of the study and the willingness of individuals to participate?

We do not anticipate any major changes to the risk/benefit ratio. The movement to a Randomized Controlled Trial does introduce the presence of a control group, who will not have immediate access to the study intervention, but rather will have to wait until after the 6 month follow up assessments are completed before they are invited to take the 9-week course. Some participants may not find this ideal; however, the details of the RCT are reviewed with the participants at the time of informed consent. There is also some additional burden to study participants as the in-person assessments have increased in duration by around 30-45 minutes. Again participants will be made aware of their involvement at the time of informed consent.

5. Does the requested amendment require new documents or changes to the approved consent form or other documents?

Yes. Since we are transitioning from a prospective single arm trial to a two arm randomized controlled trial most of the study documents have undergone significant changes. See below for a list of attachments.

Appendix 1	Informed Consent Form (v.2.0) – *with track changes*
Appendix 2	Phone Screener (v.2.0) – *with track changes*
Appendix 3	In-person Screener (v.2.0) – *with track changes*
Appendix 4	In-Person Baseline Assessment (v.2.0) – *with track changes*
Appendix 5	Home Baseline Assessment (v.2.0) – *with track changes*
Appendix 6	MB-BP Study Safety Plan (v.2.0) – *with track changes*
Appendix 7	Letter to health care provider if a participant screens for uncontrolled hypertension (>140/90 mmHg) AND has indicated that his/her provider is unaware of high BP levels.
Appendix 8	Recruitment Materials with website noted

Appendix 1

Informed Consent Form (v.2.0) – *with track changes*

The Mindfulness-Based Blood Pressure Reduction (MB-BP) Study

Agreement to Participate in a Research Study

Investigation of the Effects of Mindfulness on Blood Pressure and Well-Being

You are being asked to take part in a Brown University research study about the effects of mindfulness and hypertension education on blood pressure and risk factors for hypertension. This form will explain the purpose of the study, how the study will be carried out and what you will be expected to do. It will also explain the possible risks and possible benefits of being in the study. If any part of the following description is not clear to you, you are encouraged to contact the researcher to answer any questions before you decide whether to take part in the study. If you decide to participate, please fill out and sign the last page of this form.

1a. Nature and Purpose of the Study

The purpose of the study is to investigate the impact of mindfulness practices and health education on blood pressure. You have been selected for this study because you expressed interest in the project and because you met entrance criteria for having prehypertension or hypertension, or another cardiovascular risk factor that could be influenced by this program. Eligibility for the study is still being assessed.
Therefore, it is possible you may not be eligible for the study even after signing this consent form. Your participation in this study is voluntary and can be withdrawn at any point in the project.

In order to assess the effects of the customized mindfulness intervention, you will be asked to complete some questionnaires and laboratory assessments before and after the intervention. Specifically, assessments will be completed at: baseline, 10 weeks, 6 months and 1 year. To express our gratitude for participation, you will be given $25, $25, and $50 (up to $100 USD total) at the 10 week, 6 month and 12 month follow ups respectively. As part of the study you will also be given a wireless blood pressure monitor (estimated value of $90) to use throughout the study.

This is a Randomized Control Trial. Using a covariate adaptive randomization process, participants enrolled into the study will be randomly assigned to one of two groups: (1) the intervention group or (2) the wait-list control group. The wait-list control group will be given the opportunity to participate in the intervention after the six month follow up assessments are completed. Both the intervention and the control group will be asked to participate in the research assessments.

~~In order to assess the effects of these practices, you will be asked to complete some questionnaires and laboratory assessments before and after learning the mindfulness practices. Assessments will be completed at: baseline, 10 weeks, 6 months and 1 year. If you complete the study, you will be given $100 USD at the time of completion for your participation, to express our gratitude.~~
~~Participation in this study involves receiving training in mindfulness practices as well as health education on blood pressure.~~

1b. Explanation of Procedures

If you agree to participate, you will be asked to consent to the following:

1) Participation in an interview in which you will be asked questions about past and present mental health, including depression and suicide (previously completed with your verbal consent).

2) Completion of an in-person screening assessment, during which your blood pressure, height, weight and other basic demographic and health data will be collected and assessed in order to determine eligibility for the study.

~~2)~~3) Completione of questionnaires administered in-person and online that ask about a wide range of topics, including your diet, physical activity, smoking, medication use, personality, emotions, attention and past experiences, including stressful or traumatic experiences. These questions will probe sensitive psychological areas, including physical, emotion and sexual abuse. These questionnaires may take up to 3 hours to complete. By completing the interviews and questionnaires, you are giving the researchers permission to use the information you have provided. You have the right not to answer any of the questions.

~~3)~~4) Directly assessed blood pressure, heart rate, height, weight, ~~waist circumference, hip circumference,~~ physical activity, and antihypertensive (blood pressure) medication use at baseline and after the mindfulness course. Physical activity will be assessed for a week at a time using small actigraphy monitors (i.e., Fitbits) that attach to your wrist ~~and hip~~. If you take antihypertensive medication, we will provide you with an electronic bottle cap that will automatically record when the pill bottle is opened during the study. This will help us measure how often the medication is used. We will also provide you with a wireless blood pressure monitor and will ask that you take your blood pressure at home systematically during each of the research assessment periods (i.e., baseline, 10 week, 6 month and 12 month).

~~4)~~5) You will be asked to perform some cognitive tasks. Some of these tasks may involve computer-based tests of attention or decision-making. Together these tests may take as long as ~~45~~20 minutes.

6) During the in-person assessments you will also be given a battery of stress tests that are designed to induce a stress response so that we can monitor your cardiovascular response and recovery.

7) Attendance at an information session that is to be held within two weeks of the start of the intervention.

~~5)~~8) If randomized into the intervention group, y~~Y~~ou will participate in the mindfulness program, which consists of 9 weekly sessions of 2.5 hours each and will include one 7.5~~8~~ hour weekend retreat. Daily ~~homework~~ at home practice assignments may take as long as one hour and consist of practicing mindfulness exercises with the aid of a guided ~~audio~~

Commented [SF1]: No longer collecting this data.

Commented [SF2]: Removed the Attention Network Task (ANT)

Commented [SF3]: NEW

Commented [SF4]: NEW

~~CD~~meditations and completing worksheets related to stress, thoughts, and common reactions to various types of events. If you are randomized into the wait-list control group, you will be invited to take part in the mindfulness class after the completion of the 6 month follow up assessments.

Commented [SF5]: Guided meditations are provided in multiple formats

~~6)~~9) Class sessions ~~will~~ may be audio taped so we can analyze the quality of the treatment you receive. The recordings will be transcribed so that we may analyze the text. The recordings will be identified by study number, will only be heard by study staff and will be destroyed after transcription.

~~7)~~10) You ~~will~~ may be asked to complete a few short questionnaires each week during the 9 week condition.

~~8)~~11) After 10 weeks from the start of the intervention, you will be asked to complete questionnaires and return to the laboratory to repeat the same procedures for a second day of testing. If in the intervention arm, y~~Y~~ou will also be invited to participate in a focus group to share any advice you may have on how to improve the intervention.

~~9)~~12) Six months and one year after the beginning of the study, you will be asked to return to the laboratory to repeat the same testing procedures.

Table Summarizing Activities and Time Commitment for this Study.

Commented [SF6]: Reorganized this table to be more clear about study components and participation.

**Activity**	**Estimated Time Commitment**
In-person screening assessment	0.5 hours
Baseline<br>In-person assessment<br>Online questionnaire<br>At home health monitoring (e.g., fitbit, BP, etc.)	 2.0 hours<br>1.0 hours<br>1.0 hours
Information session	1.0 hour
Intervention*<br>Mindfulness course<br>Home practice assigned during course<br><br>**Intervention group only; control group will be invited to take part in a class post 6 month follow up but it will not be required as part of the study.*	Nine 2.5 hour sessions<br>7.5 hour all day retreat<br>Up to 1hr daily home practice assignments<br>*Total course time: 30.0 hours*<br>*Max. practice time: 48 hours*
Focus group participation post intervention (*intervention group only*)	1.5 hours

Follow Ups – 10 week, 6 month, 12 month<br>In-person assessments<br>Online questionnaires<br>At home health monitoring (e.g., fitbit, BP, etc.)	Total follow up time:<br>6.0 hours<br>3.0 hours<br>3.0 hours
**TOTAL ESTIMATED TIME COMMITMENT**	17.5 hours – CONTROL<br>97.0 hours - INTERVENTION

Table Summarizing Activities and Time Commitment for this Study.

**Activity**	**Time Commitment**
First blood pressure screen	30 minutes
Second blood pressure screen	30 minutes
Baseline health assessments, such as questionnaires, height, weight and waist circumference.	3 hours
Mindfulness course<br>*In-person*	9 sessions that are 2.5 hours each.<br>1 retreat day on a Saturday that will be 8 hours. *Total course time: 30.5 hours*
Home practices assigned during mindfulness course.	1 hour per day, 6 days per week, for 8 weeks. *Total practice time: 48 hours*
Health assessments, such as questionnaires, blood pressure, height, weight and waist circumference, that take place *immediately after course completion.*	3 hours
Health assessments, such as questionnaires, blood pressure, height, weight and waist circumference, that begin *6 months after baseline.*	3 hours
Health assessments, such as questionnaires, blood pressure, height, weight and waist circumference, that begin *1 year after baseline.*	3 hours
Participate in focus group after intervention completion to share any advice you may have on how to improve the intervention.	1.5 hours
**TOTAL TIME COMMITMENT FOR STUDY**	**93.0 hours**

Feedback:

At the end of the study, you will receive individual feedback about the changes that occurred since the first assessment. Specifically, you will receive an individualized handout listing % change (increase or decrease) on scales of attention, stress, mood, health behaviours, weight, and blood pressure across the study.

2. Discomforts and Risks

The risks to you in this study are small. The questionnaires used in the study are routine, standardized forms for epidemiologic research. Certain questions may be upsetting as they may probe sensitive psychological areas and inquire about upsetting or traumatic events, including physical, sexual or emotional abuse and/or current psychiatric symptoms. The cognitive tests and stress battery may also invoke a stress response that may be uncomfortable. All aspects of the study are voluntary; Since your participation is voluntary, you have the right to skip anything during the study questions that makes you uncomfortable.

Meditation-based interventions may results in discomfort with attention to unpleasant thoughts, feelings or body sensations. Some individuals may experience an initial increase in undesirable feelings with increased attention to them.

It is possible that injuries could be sustained during the study either from the gentle mindful movements (i.e., yoga), or from physical activities that participants engage in as a way to reduce blood pressure. To help limit this, you will receive a handout showing the yoga poses that will be offered during the course that you can show your health care provider so that they can advise on which poses to do, and which to avoid. Modifications of poses will be available as needed. None of the poses (or the yoga as a whole) are mandatory to be done. You will also be encouraged to explore physical activities that promote strength and conditioning as a way to reduce blood pressure. You will be encouraged to not go beyond any physical limits of your body, and will be encouraged to ask your healthcare provider about advised physical activities and mindful movements if you have any physical limitations.

While physical and mental injury is always a possibility the potential for harm is limited. Note that a research injury is any physical or mental injury or illness caused by your participation in the study. If you are injured by a medical treatment or procedure that you would have received even if you were not in the study, that is not a research injury. To help avoid research injury and potential added medical expenses, it is important to follow all study directions carefully. If you are covered by insurance and suffer a research injury, it is possible that some or all of the costs of treating your condition could appropriately be billed to your insurance company. If such costs are not covered your health insurance company, it is possible you would have to pay for these costs out of pocket. Brown University's policies do not cover payment for such things as lost wages, medical care expenses, or pain and suffering.

Precautions should be taken to avoid injuries. If you do become injured during the study, you should call your doctor immediately. You should also alert the study staff that you have been injured. Heart attack and sudden death related to heart problems have been known to occur in people while they are exercising. This is very rare, however. Estimates of sudden cardiac death range from 0 to 2 per 100,000 hours. However, the researchers cannot guarantee that no complications will happen to you.

3. Benefits

We cannot and do not guarantee or promise that you will receive any direct benefits from this study. However, participation in the study creates the potential benefit of a) identifying effective treatments for elevated blood pressure, b) gaining knowledge of the effects of mindfulness practices, c) receiving information about your psychological and physical functioning. The use of the at home blood pressure monitor also provides the potential benefit of live time blood pressure monitoring.

4. Alternative Therapies

A number of different therapies, including antihypertensive medication, diet changes, physical activity, and reducing excessive alcohol consumption may also be beneficial for reducing blood pressure. Education about these therapies are integrated into this course, but other forms of these alternative therapies are also available in the community.

5. Confidentiality

Your responses for this study will be kept confidential. All data that we collect will be linked to a study ID# instead of your name. All questionnaires in this study will be filled out through an online survey or a paper version of the survey if you prefer. All of these questionnaires will be linked solely to your study ID#, so that your identity is protected and your answers are confidential. Although these measures have been taken to protect your personal information, complete confidentiality cannot be guaranteed when transmitting information over the internet.

While your confidentiality is protected to the extent of the law, there are limitations to confidentiality. If your questionnaire responses indicate that you pose a serious danger to yourself or to another person, then a collaborator (Dr. Ellen Flynn) who is a licensed psychiatrist, may contact you to discuss your responses and possible referral to a treatment provider. Questionnaire items that may warrant follow-up include endorsements of statements about hurting yourself, any high scores in depression, anxiety, or other clinically significant problems. You should also know that there are times when the law might require the release of your responses without your permission. For example, State law requires researchers to report abuse or neglect of children to the Department of Children, Youth and Families (DCYF). State law also requires researchers to report abuse or neglect of people age 60 and older to the Division of Elderly Affairs.
The findings of the study may be used for medical publication. Your name will not be used in any published reports about this study. Results will be reported in a summarized manner in such a way that you cannot be identified. All personally identifiable information will be "de-identified" and only a unique code number will be used. Study records will be identified with a unique code number and initials. All study records and specimens will be stored in a secure storage area.
Keeping study records: The Principal Investigator for this study will keep your research records indefinitely for research purposes.

6. Refusal/Withdrawal

Participation is voluntary, and you may decide to opt out or refuse any part of participation, including not answering certain questions. If you decide now to participate, you can change your mind later and quit the study. The decision to not participate or to withdraw from the study will not adversely affect current or future interactions with Brown University. The decision to not participate or to withdraw from the study will also not adversely affect your relationship with your physician.

If you decide not to participate, or if you quit the study, we will provide you with referrals for alternative treatments, if desired.

7. Contact Information

If you have questions about study procedures at any time you may contact the researchers: Dr. Eric B. Loucks, email: eric.loucks@brown.edu, telephone (401) 863-6283. If you would like more information about the rules for research studies, or the rights of people who take part in

those studies, you may contact the Brown University Human Research Protection Program, telephone number 1-866-309-2095 or 401-863-3050.

A description of this clinical trial will be available on http://www.Clinical Trials.gov, as required by U.S. Law. This Web site will not include information that can identify you. At most, the Web site will include a summary of the results. You can search this Web site at any time.

CONSENT FORM:
Please sign and return.

Please read the following agreement and sign to consent to participating.

I HAVE READ THE ABOVE DESCRIPTION OF THIS STUDY. ALL OF MY QUESTIONS HAVE BEEN SATISFACTORILY ANSWERED, AND, I AGREE TO PARTICIPATE IN THIS RESEARCH STUDY.

____
PRINT NAME

___________________________________ _________________
Signature of participant **Date**

STUDY PARTICIPANT CONTACT INFORMATION

Name (print):____

Permanent Address:____

Email(s):____

Telephone:________________________**(cell)** ________________________**(other)**

Appendix 2

Phone Screener (v.2.0) – *with track changes*

PHONE-DELIVERED SCREENING QUESTIONNAIRE

PHONE SCREENING QUESTIONNAIRE - PART 1 of 2

*The script is shown below in **bold italics.***

PID. Participant ID: ___________

SQ01. Staff ID: ______________

SQ02. Today's Date (MMDDYY): ________________

SQ03. Current Time (24 hour time, e.g. 14:45): ________

Please now call the participant.

SQ04. Was the participant reached?   YES   NO

Hello, my name is ____________. I am calling from the Brown University School of Public Health because (name of participant) expressed interest in participating in our mindfulness blood pressure study. Is he/she available to talk at this time?

I would like to do a 10-15 minute phone interview with you to determine if you are a good match for this particular study.

SQ05. ***Is now a good time to speak?***

[If yes, proceed to SQ05 script below]

If no… ***When would be a good time to talk?***

SQ06. Day to call back (DDMMYY) ____________

SQ07. Time to call back ______ AM/PM

OK, great. I'm going to give you a quick overview of the study first and can then move on to the phone screener to see if you may qualify for the next part of the study. Feel free to interrupt me at any point if you have a question. Okay?

STUDY OVERVIEW: In this study, we are looking to see if mindfulness practices improve blood pressure, and if education about hypertension risk factors may also improve blood pressure. If you are eligible and are randomly selected to take part in the intervention, we will provide you with training in meditation, mindful movements, and the roles of things like diet, physical activity and medication in reducing blood pressure.

You will be taught by a very experienced teacher who is an expert in these fields. The course is free and will take place over a 9-week period, where you come to a class once each week for 2.5 hours each time. There is also a one-day weekend retreat that will be 7.5 hours long. [Go over days / times of course and retreat]

As part of the project, we will also ask you to participate in health assessments before and after the ~~study~~intervention. Health assessments include measures such as blood

pressure, height and weight, and questionnaires about your health and experiences. ***These assessments will take place at four points throughout the study. At baseline, 10 weeks, 6 months and 12 months.***

It is important to note that this is a Randomized Controlled Trial, which means that, if eligible, you will be randomly assigned to one of two groups. The intervention group will be offered the 9 week mindfulness course right away, while the wait-list control group will be invited to take the course after the six month follow up assessment is completed. You will be notified of the randomization results 4-7 days prior to the start of class.

As an expression of gratitude for your time and effort, you will be given cash incentives at each of the three follow up assessments. You will be given $25 at the first two follow ups and then $50 at the final 12 month follow up. You will also receive a state-of-the-art wireless blood pressure monitor that will be yours to keep. You will be asked to use this monitor to take your blood pressure at home throughout the study.

~~***At the end of the study, you will be given $100 USD to express our gratitude.***~~ ***Do you have any questions about the mindfulness intervention or the research study as a whole? [****Answer questions****]***

SQ08. ***Does this study sound like something you would be interested in doing? Yes No***

[If yes, proceed to next statement below. If no, politely thank the participant for considering being in this study, and end the call].

Great. There are a few things that I would like to go over before we start the interview. First, some of the questions that I will ask now to figure out if you are eligible to be in this study will be of personal nature, including asking about your mental health and life's experiences.

SQ09. ***Are you in a private place to talk?***

[If yes, proceed to text below. If no, reschedule meeting using variables SQ06 and SQ07 above].

Because this interview is of a personal nature, it is important that you understand that everything you say will be kept strictly confidential. No one outside of our project will ever be able to see your answers, and we will not keep your name in the same place as any of your answers. If you are not eligible after the phone screen, we will destroy your information. If you like, though, we can keep your information on file for future studies.

Ok, to begin, I am going to start by recording your name and contact information.

SQ10. Participant's First Name: ______________________________________

SQ11. Participant's Last Name:____

Participant's Address (in case we need to send any study materials to you):
SQ12a. Street address: ______________
SQ12b. City: _____________________
SQ12c: State: _________
SQ12d: Zip Code: _________________

SQ13a. Participant's Phone number #1 (in case we need to contact you by phone)
______________________________________ Type of Phone: work / home / cell / other

SQ13b. Participant's Phone number #2
______________________________________ Type of Phone: work / home / cell / other

SQ14. Participant's email address (or mailing address if no email):_______________________

SQ15. Notes from interviewer related to participants' contact information (if any):
____
____
____

PHONE SCREENING QUESTIONNAIRE - PART 2 of 2

PID. Participant ID #: ______________

SQ.recruit How did you find out about the study? *Select one.*

- From a friend / family member / coworker .... [ ]
- From a former or current participant... [ ]
- From PCP or other health care provider....................................... [ ]
- Facebook.. [ ]
- Twitter... [ ]
- Craigslist... [ ]
- Saw an orange flier / card (note location __________)................ [ ]
- Other (please describe ___________________________).......... [ ]

INCLUSION CRITERIA: All answers in 3rd column must be YES. If an answer is NO, immediately proceed to question SQ40.

			Comments
SQ26.	***What is your age?*** [Is age at least 18 years?]	YES NO	
SQ27.	***Can you read and write in English?***	YES NO	

EXCLUSION CRITERIA: All answers in 3rd column must be NO with the exception of SQ32a and SQ33a. If an answer (other than SQ32a and SQ33a) is YES, then immediately proceed to question SQ40.

			Comments
I will now start to ask some questions about your mental health.			
SQ28.	***Has anyone ever told you that you have bipolar disorder or manic depression?***	**YES NO**	
SQ29.	***Has anyone ever used the word "Borderline" to describe you?***	**YES NO**	
SQ30a.<br><br>SQ30b.	***Have you ever had a hallucination or seen things that other people can't see, or hear things other people can't hear?***<br><br>***Have you ever been diagnosed with schizophrenia or psychosis?***	**YES NO**<br><br>**YES NO**	
 <br>SQ31_1<br>SQ31_2<br>SQ31_3<br>SQ31_4<br>SQ31_5<br>SQ31_6<br>SQ31_7<br>SQ31_8	***Have you ever taken any of the following medications that I am about to read to you?***<br>***Lithium***<br>***Seroquel (quetiapine)***<br>***Abilify (aripiprazole)***<br>***Zyprexa (olanzapine)***<br>***Clozaril (clozapine)***<br>***Haldol/Haloperidol***<br>***Geodon (ziprasidone)***<br>***Risperdal (risperidone)***	 <br>**YES NO DK**<br>**YES NO DK**<br>**YES NO DK**<br>**YES NO DK**<br>**YES NO DK**<br>**YES NO DK**<br>**YES NO DK**<br>**YES NO DK**	
SQ32a	***Have you ever had a suicide attempt?***	**YES NO**	

SQ32b<br><br>SQ32c<br><br><br><br>SQ32d	***[If yes, ask…] Have you considered killing yourself during the past month?***<br><br>***[If yes, ask…] Are you currently suicidal?***<br><br>***[If yes, keep participant on the phone, and follow suicide safety plan below]***<br><br>***[If no, ask…] Are you getting any help for that? If not, then provide list of resources from Safety Plan including Gateway, Anchor and The Providence Center] Not urgent, but inform Dr. Flynn about what was discussed.***	**YES NO**<br><br>**YES NO**<br><br>**YES NO**	

EXCLUSION CRITERIA: All answers in 3rd column must be NO with the exception of SQ32a and SQ33a. If an answer (other than SQ32a and SQ33a) is YES, then immediately proceed to question SQ40.

SQ33a<br><br>SQ33b<br><br>SQ33c	***Would you say you have a trauma history? [If yes…]***<br>***In the past month, have you had any problems with dissociation (memory loss)?***<br>***In the past month, have you had any flashbacks (i.e. sudden and disturbing vivid memory) about the trauma?***	**YES NO**<br><br>**YES NO**<br><br>**YES NO**	
SQ34a<br><br><br><br>SQ34b	***In the past month, have you had any problems with obsessions or compulsions, such as washing your hands or checking the oven over and over again?***<br>***[If yes…] Has anyone diagnosed you with obsessive compulsive disorder?***	**YES NO**<br><br><br><br>**YES NO**	
SQ35	***In the past month, have you had a panic attack (i.e. sweating, heart palpitations, nausea, trouble breathing, fear of dying/choking/going crazy)?***	**YES NO**	
SQ36	***Have you had any problems with alcoholism or drug use in the past year?***	**YES NO**	
SQ37	***In the past year, have you had an eating disorder, such as starving, binge eating, or vomiting?***	**YES NO**	
SQ38a<br><br><br>SQ38b	***Do you currently have a mindfulness practice, such as meditation or yoga?***<br><br>***If Yes –***<br>***Please tell me more about your mindfulness practice, including how often you practice per week.***	**YES NO**<br><br>**Fill in response in comments section to the right**	

SQ38c	***Do you currently practice meditation more than once per week? (yoga does not count as meditation in this context)***	**YES NO**	
SQ39	***This class will take place at Brown University in-person. Do you have any medical or mobility issues that would affect you being able to attend class?***	**YES NO**	
SQ40	Participant qualifies for next step of study (next step is 1st blood pressure screening)	**YES NO**<br><br>**If YES go to SQ41.**<br><br>**If NO, go to SQ42.**	

SQ41a. ***Thank you for taking the time to answer these questions. You qualify for the next stage of screening, which is to take your blood pressure at our office. If you're still interested, we'd like to schedule a time to have you come in to complete the in-person screener. It will only take 30 minutes or less and will involve taking your blood pressure 3-5 times, as well as measuring your height and weight and asking a few questions. Is there a day or time that works best for you?*** *[Schedule the In-Person Screening visit now]*

SQ41b. ***~~As I mentioned earlier, there is a mindfulness intervention that is part of this study. If you are eligible based on the In-Person screening, you will then be invited to take part in a 9 week Mindfulness Course. We will be scheduling the mindfulness intervention at a time that works best with most of the participants. What days and times of the week typically work best with your schedule? Please keep in mind that the sessions are 2.5 hours long, and take place once per week for 9 weeks.~~INTENTIONALLY REMOVED***

Commented [SF1]: Our intervention dates are already scheduled.

Thank you for your time and interest in this study. We look forward to meeting you in person on [DATE / TIME]. Do you have any questions before we end this call?

IF INELIGIBLE:

SQ42a***. Thank you for taking the time to answer these questions. According to the survey, you do not qualify for the study at this time. There may be other studies you quality for.***

SQ42b. ***Would you like me to keep your information to pass on to these studies?*** YES / NO

SQ42c. [If yes…] ***OK, thank you. We will keep this information for future studies you may qualify for. Thank you for taking the time to talk with me today. Can I answer any questions before hanging up?***

SQ42d. [If no…] ***OK, our copy of this information will be destroyed. Thank you for taking the time to talk with me today. Can I answer any questions before hanging up?***

SAFETY PLAN – PHONE SCREENER ONLY

Enter details below on paper during screening (These variables should are also be entered in the survey via questions SQ10-SQ13). Destroy this paper after screening is complete.

Participant's First Name: ____________________________________

Participant's Last Name:______________________________________

Participant's Address:

Street address: _____________

City: ____________________

State: ________

Zip Code: ________________

Participant's Phone number #1: ____________________________________

Participant's Phone number #2____________________________________

During the phone-based screening, if participants respond yes to "Are you currently suicidal?", the interviewer should perform the following 2 steps:

1. **Immediately have 911 and Dr. Ellen Flynn called by a colleague who has been informed beforehand that this is a possibility.**

<u>Specifically, while keeping the participant on the phone, show the text below in the box to a colleague.</u>

I have a study participant on the phone who is currently suicidal. Please call 911 immediately, and tell them:

"I am calling on behalf of [my name] who is performing a research study at Brown University. He has a participant on the phone who says they are currently suicidal." Please provide the participants' contact information to the 911 operator (i.e. name, address, phone #, email address) as requested. This information is shown above.

Finally, after calling 911, please call Dr. Ellen Flynn, who is the psychiatrist supporting this study. Her cell phone # is 401-258-9829. Please provide her with the same information as was done during the 911 call.

2. **While speaking calmly with the participant, let them know what you are doing. Specifically, let them know we are calling our study's psychiatrist Dr. Flynn and 911, and why you are doing that (i.e. because we are concerned about you). You can speak with participant to keep him/her on the phone, but the discussion should not be clinical in nature.**

Examples of questions that could be asked in order to keep them on the phone:

- "Tell me what is going on."
- "What's happening right now?"
- Tell me more about why you are interesting in being part of this study.
- What are you hoping to get out of this study?

The following information can be provided to study participants if they state they have had considered killing themselves in the past month. If they are currently suicidal, the main priority is to keep them on the phone while 911 and Dr. Flynn are being contacted.

National Suicide Prevention Lifeline: 1-800-273-8255

Other options are to:

- Call your doctor's office
- Call 911 for emergency services
- Go to the nearest hospital emergency room.

Local Non-Urgent Free or Inexpensive Mental Health Services:
Gateway Healthcare: 401-729-8701
Anchor Counseling Center: 401-475-9979
The Providence Center: 401-276-4020

Appendix 3

In-person Screener (v.2.0) – *with track changes*

IN-PERSON SCREENING

ASSESSMENT FORM

PID. Participant ID # __________
BA01. Staff ID # __________
BA02. Today's date (MMDDYY): __________

Blood Pressure:

BA03a. Blood pressure 1st reading, systolic blood pressure: _________ mmHg
BA03b. Blood pressure 1st reading, diastolic blood pressure: __________ mmHg
BA03c. Blood pressure 2nd reading, systolic blood pressure: _________ mmHg
BA03d. Blood pressure 2nd reading, diastolic blood pressure: __________ mmHg
BA03e. Blood pressure 3rd reading, systolic blood pressure: _________ mmHg
BA03f. Blood pressure 3rd reading, diastolic blood pressure: __________ mmHg

BA04. Were the 2nd and 3rd systolic blood pressure readings within 20 mmHg of each other?
- □ Yes
- □ No ***(repeat measurements)***

BA05. Were the 2nd and 3rd diastolic blood pressure readings within 10 mmHg of each other?
- □ Yes
- □ No ***(repeat measurements)***

BA06a. Repeated blood pressure 1st reading, systolic blood pressure: _________ mmHg
BA06b. Repeated blood pressure 1st reading, diastolic blood pressure: __________ mmHg
BA06c. Repeated blood pressure 2nd reading, systolic blood pressure: _________ mmHg
BA06d. Repeated blood pressure 2nd reading, diastolic blood pressure: _________ mmHg
BA06e. Repeated blood pressure 3rd reading, systolic blood pressure: _________ mmHg
BA06f. Repeated blood pressure 3rd reading, diastolic blood pressure: __________ mmHg

Blood Pressure Safety Protocol – The out-of-range blood pressure values are as follows: systolic blood pressure >200 mmHg or <90 mmHg; diastolic blood pressure >110 mmHg.

In absence of symptoms (chest pain, shortness of breath, dizziness, headache), for a SBP>200 or DBP>110 or both, we will strongly encourage participants to see their doctor right away or to go to urgent care. If there are symptoms, we will immediately call 911.

In absence of symptoms (chest pain, shortness of breath, dizziness, passing out), for a SBP<90, we will strongly encourage participants to see their doctor right away or to go to urgent care. If there are symptoms, we will immediately call 911.

BA07. Blood pressure cuff size used: □ S □ Reg □ L □ XL

BA08. Arm that cuff was placed on: □ L □ R

BP monitor BP monitor used:
- ❍ Unit #1 - HEM-705CP (1)
- ❍ Unit #2 - HEM-705CP (2)
- ❍ Other (specify: (3) ____________________

ELIGIBILITY OUTCOME - based on the average of the 2nd and 3rd BP (repeat) readings, note the participant's eligibility below and read the corresponding script:

Not Eligible: mean systolic BP < 120mmHg AND mean diastolic BP < 80mmHg

Unfortunately, at this time you are not eligible for the study. *If participant is interested, we can keep his/her contact information on file for future studies. END ASSESSMENT.*

Eligible: mean systolic BP ≥ 120mmHg OR mean diastolic BP ≥ 80mmHg

Congratulations, you are eligible for the study! At this time we would like to take your height and weight, ask a few follow up questions and go over the next steps of the study. *Note that if the participant does not wish to continue, thank the participant for his/her time and then END THE ASSESSMENT. Otherwise, continue below.*

BA09. Height: ______ . __ cm (one decimal place)

BA10. Weight: ______ . __ lbs (one decimal place)

BA11. What is your date of birth? _____ / _____/ _____ [*mm/dd/yyyy*]

BA12. How old are you?: ______ years

BA13. *Is participant male or female?* □ Male □ Female □ ~~Unknown~~Other/non-binary

BA14. Do you currently take any prescription medications to reduce blood pressure?

□ Yes – *go over eCAP protocol; equipment will be given to participant at baseline*

□ No ***– SKIP TO END***

BA14a Have you made any changes to your blood pressure medication in the past four weeks? (YES / NO) ***If no, skip to end.***

BA14b Describe changes to BP medication below (e.g., timing and description of change) and use this information when scheduling the baseline assessment:

Go over the next phase of the study, including discussing the Fitbits and eCAPs (if applicable). Before participant leaves, schedule the in-person baseline assessment (must be within 1-3 weeks of the scheduled intervention) or have the Project Coordinator follow up to schedule.

END ASSESSMENT

Appendix 4

In-Person Baseline Assessment (v.2.0) –

with track changes

IN-PERSON BASELINE

QUESTIONNAIRE AND ASSESSMENT FORMS

PID. Participant ID # __________
BA01. Staff ID # __________
BA02. Today's date (MMDDYY): ___________

Blood Pressure:
BA03a. Blood pressure 1st reading, systolic blood pressure: _________ mmHg
BA03b. Blood pressure 1st reading, diastolic blood pressure: __________ mmHg
BA03c. Blood pressure 2nd reading, systolic blood pressure: _________ mmHg
BA03d. Blood pressure 2nd reading, diastolic blood pressure: __________ mmHg
BA03e. Blood pressure 3rd reading, systolic blood pressure: _________ mmHg
BA03f. Blood pressure 3rd reading, diastolic blood pressure: __________ mmHg

If the difference of the 2nd and 3rd systolic BP reading is 20 mmHg or greater OR the difference of the 2nd and 3rd diastolic reading is 10 mmHg or greater, then repeat the BP readings. Otherwise, skip to BA07.

BA06a. Repeated blood pressure 1st reading, systolic blood pressure: _________ mmHg
BA06b. Repeated blood pressure 1st reading, diastolic blood pressure: __________ mmHg
BA06c. Repeated blood pressure 2nd reading, systolic blood pressure: _________ mmHg
BA06d. Repeated blood pressure 2nd reading, diastolic blood pressure: __________ mmHg
BA06e. Repeated blood pressure 3rd reading, systolic blood pressure: _________ mmHg
BA06f. Repeated blood pressure 3rd reading, diastolic blood pressure: __________ mmHg

Blood Pressure Safety Protocol – The out-of-range blood pressure values are as follows: systolic blood pressure >200 mmHg or <90 mmHg; diastolic blood pressure >110 mmHg.

In absence of symptoms (chest pain, shortness of breath, dizziness, headache), for a SBP>200 or DBP>110 or both, we will strongly encourage participants to see their doctor right away or to go to urgent care. If there are symptoms, we will immediately call 911.

In absence of symptoms (chest pain, shortness of breath, dizziness, passing out), for a SBP<90, we will strongly encourage participants to see their doctor right away or to go to urgent care. If there are symptoms, we will immediately call 911.

Follow Safety Protocol for Uncontrolled Hypertension (140/90 mmHg or greater)

BA07. Blood pressure cuff size used: ◻ S ◻ Reg ◻ L ◻ XL
BA08. Arm that cuff was placed on: ◻ L ◻ R

BP monitor BP monitor used:
❍ Unit #1 - HEM-705CP (1)
❍ Unit #2 - HEM-705CP (2)
❍ Other (specify: (3) ____________________

We are now going to take your weight. *Have ppt remove his/her shoes and empty his/her pockets. Remove bulky clothing as well.*

BA10. Weight: ______ . __ lb (one decimal place)

Commented [SF1]: This is programmed into Qualtrics. Did not need BA04 OR BA05.

Medications (ME)

The next questions are about medications used to treat hypertension or high blood pressure.

ME01. Not including vitamins and supplements, do you currently take any prescription medications or over-the-counter drugs for high blood pressure?~~Do you take any prescription medications or over-the-counter drugs?~~

☐ No ~~→ *skip to end of medications questions*~~
☐ Yes
☐ Don't know
☐ Prefer not to answer

ME01a IF NO, Have you ever taken medication for high blood pressure?

☐ No → ***skip to end of medications questions***
☐ Yes → ***skip to end of medications questions***

If you brought your medications with you, please take them out now as we will use them to complete the next section.

ME01b INTERVIEWER CHECKPOINT - Please select one:

☐ Rx info not known or available – ***skip to end of ME questions; will need to follow up***
☐ Ppt brought medications -- ***continue***
☐ Ppt did not bring medications but knows info – ***continue, may need follow up***

ME01c In total how many different medications and/or over the counter drugs do you currently take for high blood pressure? Again, do not count vitamins or supplements. ___________ # of BP meds

Next we're going to ask you questions about each of the medications you currently take for high blood pressure.

ME02-10a. What is the name of the first prescription medication or over-the-counter drug that you take?

____

☐ Don't know ☐ Prefer not to answer

ME02-10b. What is the dosage form?

Oral
☐ Pill, tablet, or capsule
☐ Sublingual or orally-disintegrating tablet
☐ Liquid solution or suspension (drink, syrup)
☐ Powder

Topical
☐ Liquid, cream, gel, or ointment
☐ Ear drops (otic)
☐ Eye drops (ophthalmic)
☐ Skin patch (transdermal)

Inhaled
☐ Inhaler or nebulizer

Injected
☐ Injection

Suppository
☐ Rectal (e.g., enema)
☐ Vaginal (e.g., douche, pessary)

Other:
☐ Other _________

Commented [SF2]: To reduce ppt burden we have decided to only collect information about medications taken for hypertension.

Commented [SF3]: For simplicity in this document, we've combined the medication questions #2-10 since they are the same. We repeat the same questions for each hypertensive medication.

☐ Don't know ☐ Prefer not to answer

ME02-10c. How frequently do you take it?

☐ _________ times per day
☐ _________ times per week
☐ _________ times per month
☐ Don't know
☐ Prefer not to answer

ME02-10d. What is the strength? ***(Record strength of how it is actually taken, not how it is prescribed.)***

☐ _________ %
☐ _________ mg
☐ _________ mcg
☐ _________ grams
☐ _________ I.U.
☐ _________ Other unit: _____________
☐ Don't know
☐ Prefer not to answer

ME02-10f. Do you take it regularly or only as needed?

☐ Regularly
☐ Only as needed
☐ Don't know
☐ Prefer not to answer

ME02-10g. For how long have you been taking it?

☐ For _________ days
☐ For _________ weeks
☐ For _________ months
☐ For _________ years
☐ Don't know
☐ Prefer not to answer

ME02-10h. What is the medication used for?

☐ High blood pressure
☐ Prescribed for something other than high blood pressure but also acts as a hypertensive medication *(describe:* ____)

ME02-10i. ***Interviewer comments*:**

____

Repeat for all blood pressure medications the participant is currently taking.

Questions for Participants to Answer on Their Own In-Person:
For each statement, please place a mark in the column that best describes how you have been feeling.

Commented [SF4]: DK and REF options were taken out as they are not part of the standardized CESD-R and BAI self-administered surveys. Participants can always choose to skip over or refuse a question.

	Not at all or less than 1 day last week	1 or 2 days last week	3 to 4 days last week	5 to 7 days last week	Nearly every day for two weeks
DS_1. My appetite was poor.	☐	☐	☐	☐	☐
DS_2. I could not shake off the blues.	☐	☐	☐	☐	☐
DS_3. I had trouble keeping my mind on what I was doing.	☐	☐	☐	☐	☐
DS_4. I felt depressed.	☐	☐	☐	☐	☐
DS_5. My sleep was restless.	☐	☐	☐	☐	☐
DS_6. I felt sad.	☐	☐	☐	☐	☐
DS_7. I could not get going.	☐	☐	☐	☐	☐
DS_8. Nothing made me happy.	☐	☐	☐	☐	☐
C_9. I felt like a bad person.	☐	☐	☐	☐	☐
DS_10. I lost interest in my usual activities.	☐	☐	☐	☐	☐
DS_11. I slept much more than usual.	☐	☐	☐	☐	☐
DS_12. I felt like I was moving too slowly.	☐	☐	☐	☐	☐
DS_13. I felt fidgety.	☐	☐	☐	☐	☐
DS_14. I wished I were dead.	☐	☐	☐	☐	☐
DS_15. I wanted to hurt myself.	☐	☐	☐	☐	☐
DS_16. I was tired all the time.	☐	☐	☐	☐	☐
DS_17. I did not like myself.	☐	☐	☐	☐	☐
DS_18. I lost a lot of weight without trying to.	☐	☐	☐	☐	☐
DS_19. I had a lot of trouble getting to sleep.	☐	☐	☐	☐	☐
DS_20. I could not focus on the important things.	☐	☐	☐	☐	☐

Below is a list of common symptoms of anxiety. Please carefully read each item in the list. Indicate how much you have been bothered by that symptom during the past month, including today, by marking the box in the corresponding space in the column next to each symptom.

	Not At All	Mildly – it didn't bother me much	Moderately – it wasn't pleasant at all times	Severely – it bothered me a lot
BE_1. Numbness or tingling	☐	☐	☐	☐
BE_2. Feeling hot	☐	☐	☐	☐
BE _3. Wobbliness in legs	☐	☐	☐	☐
BE _4. Unable to relax	☐	☐	☐	☐
BE _5. Fear of worst happening	☐	☐	☐	☐
BE _6. Dizzy or lightheaded	☐	☐	☐	☐
BE _7. Heart pounding/racing	☐	☐	☐	☐
BE _8. Unsteady	☐	☐	☐	☐
BE _9. Terrified or afraid	☐	☐	☐	☐
BE _10. Nervous	☐	☐	☐	☐
BE _11. Feeling of choking	☐	☐	☐	☐
BE _12. Hands trembling	☐	☐	☐	☐
BE _13. Shaky/unsteady	☐	☐	☐	☐
BE _14. Fear of losing control	☐	☐	☐	☐
BE _15. Difficulty in breathing	☐	☐	☐	☐
BE _16. Fear of dying	☐	☐	☐	☐
BE _17. Scared	☐	☐	☐	☐
BE _18. Indigestion	☐	☐	☐	☐
BE _19. Faint/lightheaded	☐	☐	☐	☐
BE _20. Face flushed	☐	☐	☐	☐
BE _21. Hot/cold sweats	☐	☐	☐	☐

Pittsburgh Stress Battery: Set up and Overview

Commented [SF5]: NEW MEASURE – estimated 20 minutes to complete

"During the next part of the assessment you will be asked to complete a series of three activities, each averaging around 4-6 minutes. These tests may be difficult for you to complete and may results in feelings of stress or anxiety. We are interested in how your body responds to stress both during the tests and after the stressors are encountered. For this reason, we will be monitoring your blood pressure and heart rate before, during and after the three activities. Let us know at any point if you need to take a break. Your participation in these activities is voluntary."

SETUP:

1. Have the participant sit in the assessment chair facing the table.
2. Place the appropriate blood pressure cuff on the participant's non-dominant arm and turn the monitor away from the participant and towards yourself.
3. Set up the study laptop for the first of the three activities and then read the script below.

"To begin, I am going to take your baseline blood pressure and heart rate."

4. Record the participant's blood pressure and heart rate (Reading #1) in the data table associated with the participant.
5. Keep the blood pressure cuff on the participant for the entire battery of stress tests. Move on to the first of the three stress tests below.

Pittsburgh Stress Battery: Stress Activity #1 STROOP

"The first activity we are going to have you do is a color naming task. In this task you will see one word in the middle of the screen and four words along the bottom. You will be asked to choose the word along the bottom that names the color that the center word is printed in."

"The task can be difficult because you have to focus on the COLOR in which the center word is printed in and not on what the word reads. Here is an example:"

[REFER TO SHOW CARD #1, POINT TO IMAGE #1]

"In this example, you would select the first of the four words on the bottom - that is "red" [POINT TO RED] ***since the color of the word at the top is printed in the color RED."***

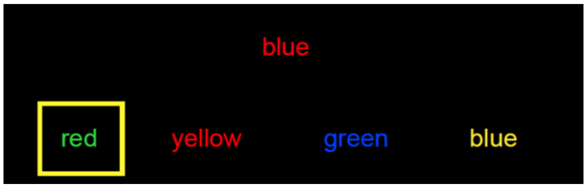

[NOW POINT TO IMAGE #2]
"Here is how we would like you to place your hand on the keyboard." [DEMONSTRATE FOR THE PPT ON THE KEYBOARD AND BY USING IMAGE #2]

"The buttons under each finger are for the four color name selections. Your index finger is on button 1, your middle finger is on button 2, ring finger on button 3 and pinky on button 4." (Note that this order would be reversed for left handed individuals.)

[STILL REFERING TO IMAGE #2]
"In the example here in Image #2, would you press button 1 (index finger), button 2 (middle finger), button 3 (ring finger) or button 4 (pinky finger)?"

[ALLOW PPT TO RESPOND BEFORE PROVIDING ANSWER]
Answer = button 2 (middle finger)

[If the participant answers, incorrectly, move on to Image #3 and ask:] ***"Let's try one more. In Image #3, which button would you press?"***

[ALLOW PPT TO RESPOND BEFORE PROVIDING ANSWER]
Answer = button 1 (index finger)

"We are almost ready to begin. While you are completing the task, we are going to turn on an audio recording that provides addition interference for the task. The entire activity takes 4 minutes to complete. We will be taking a blood pressure reading in the middle of the activity. Please continue to work on the task during the blood pressure reading. Remember to always focus on the color of the middle word and not on what the word reads. Ready?"

SET UP:

1. On study laptop, open up the ePrime software and navigate to the STROOP file.
2. Enter the PID and session number [Baseline=1; 10wk=2; 6mo=3; 12mo=4].
3. Before beginning, use the iPad to set up the timer and to open up the audio interference file.

"When I say 'begin', please hit the 'Enter' button on the keyboard. Make sure to keep your hand in the correct position on numbers 1-4."

"Ready? Begin. "

4. As soon as the ppt begins, make sure to turn on the audio interference file and start the timer.
5. At minute #2 of the test, take the participant's blood pressure and heart rate (Reading #2) and record the readings in the data table associated with the participant.
6. Once the test is complete, move the data file associated with the ppt into the correct data folder.
7. Move on to the next stress activity.

Pittsburgh Stress Battery: Stress Activity #2 Mental Math

Case Math – Mental Math Tool

https://www.preplounge.com/en/mental-math.php

INSTRUCTIONS:

1. Open up the Case Math website (overview page) on the study laptop and read the directions below before beginning.

   "You will now be asked to complete a series of mental math problems using an online program known as Case Math. To start you will be given 60 seconds to complete as many basic arithmetic problems as you can. For each problem you answer correctly, you will be given an additional two seconds of time. Answer the questions as quickly and as accurately as you are able. You will be evaluated both on your speed and your accuracy. Any questions?"

   [ANSWER QUESTIONS AND THEN USE SHOW CARD #2 FOR NEXT SCRIPT]

   "To begin, click the blue 'Start' button found in the bottom left corner under Random-Easy. The test will start right away. Solve the problems in your head as you are able. Use the keyboard on the laptop to enter your answer and then click 'Enter' to submit. Remember that you are being evaluated on both speed and accuracy."

2. Instruct the participant to complete the first Trial.
3. Once complete, record the results in the stress battery data table associated with this participant.
4. Review the results. If the participant scored 60% or greater, then have him/her move on to the "Random-Medium" level for Trial 2. Otherwise, repeat "Random-Easy" for Trial 2.
5. To start the next trial, navigate back to the 'overview' page and read the following:

   "We are now going to have you complete the exercise again. Remember that you are being evaluated on both your speed and accuracy. For this trial, please select the 'Random-[EASY/MEDIUM]' level. Click start when ready."

6. Have the participant complete Trial 2.
7. Record the results of the second math test in the table and then navigate back to the overview grid.
8. For the last trial, again increase the difficulty level if they scored 60% or greater. Otherwise make no change in difficulty. Read the following:
9. ***"You will now repeat this exercise, but during this trial we will take your heart rate and blood pressure. Please try to keep your arm with the cuff as still as possible and still attempt to do your best on the math test. For this trial, please select the 'Random-[EASY/MEDIUM/HARD]' level. Click start when ready."***
10. Once the final trial is initiated, proceed to take the BP and HR reading (Reading #3). Record the blood pressure, heart rate, and math results in the data table.

Pittsburgh Stress Battery: Stress Activity #3 Mirror Tracing

INSTRUCTIONS:

"For the last activity, you will complete four online mirror-tracing trials.

1. Open up the Mirror Tracing Qualtrics survey on the study laptop and enter the PID, cohort, and assessment info.
2. Refer to Show Card 3 and read the directions below:

   "There will be four trials to this test. In each trial, you will see two rectangular panels. The "drawing panel" is at the bottom; this is where you will move the mouse cursor. The "mirror panel" is at the top, this will record your mouse movements in mirrored format as you try to trace the figure.

   To begin a trial, move the mouse so that your cursor rests over the green circle in the drawing panel and then click the left mouse button to signal that you are ready to begin.

   Once the trial begins, a red target circle appears in the mirror panel. Trace the figure working from the starting point towards the red target circle. The trial ends automatically when you reach the red circle.

   When your mouse trail is red, you are within the lines and are earning points. When your mouse trail is blue you are outside the line and are not earning points. Your score is shown at the top of the drawing panel.

   Your score is based on the percentage of tracing made within the lines, so simply drawing straight to the red target will not earn a high score. Please try your best to earn a good score.

   You will have 45 seconds to complete each trial. You will hear an audio warning indicating when time is almost up. When 45 seconds have passed, you will be instructed to immediately move your cursor to the red dot to end the trial, regardless of whether or not you completed the tracing task. Any questions?"

3. Answer questions and then cue up the audio timer. ***"When you are ready, you may click to the next screen to begin."***
4. For all four trials, start the audio timer as soon as the participant clicks on the green circle. Stop the recording as soon as the trial is complete.
5. AFTER TRIAL 3 BUT BEFORE TRIAL 4 READ – ***"For the last trial, I will be taking your blood pressure and heart rate during the test. As much as you can, try not to move during the reading. Focus on completing the task quickly and accurately."***
6. As soon as the participant clicks the green circle in Trial 4, start the audio timer as well as the Blood Pressure cuff (Blood Pressure / Heart Rate Reading #4). Record readings on the data sheet.
7. Once complete, have the participant review his/her scores and then close the Qualtrics survey.

Pittsburgh Stress Battery

SHOW CARD #1 – STROOP TEST

Image #1 -

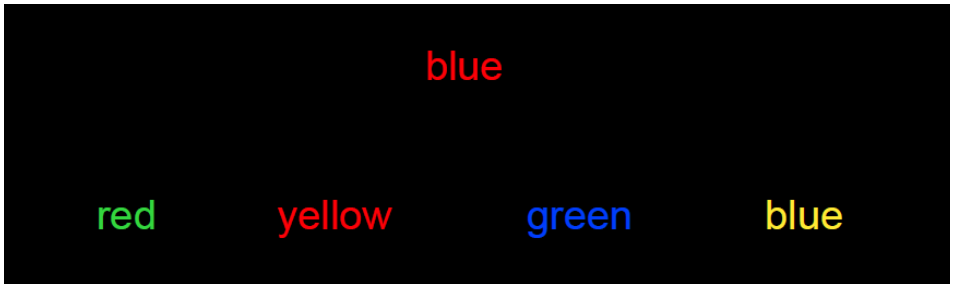

Image #2 -

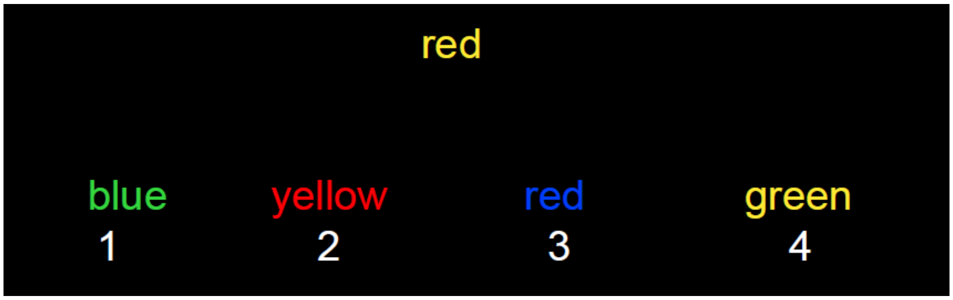

Image #3 -

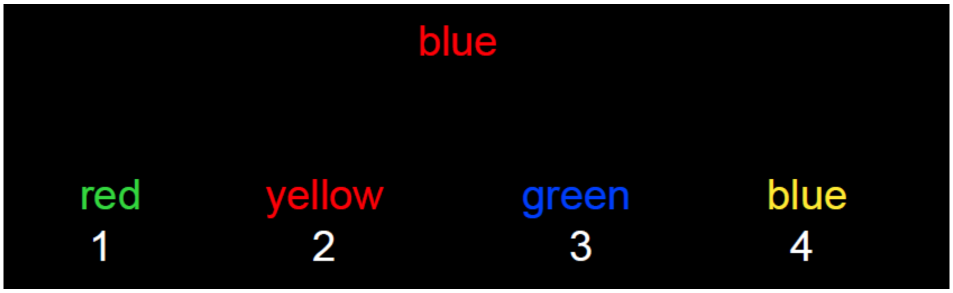

Pittsburgh Stress Battery

SHOW CARD #2 – MENTAL MATH TEST

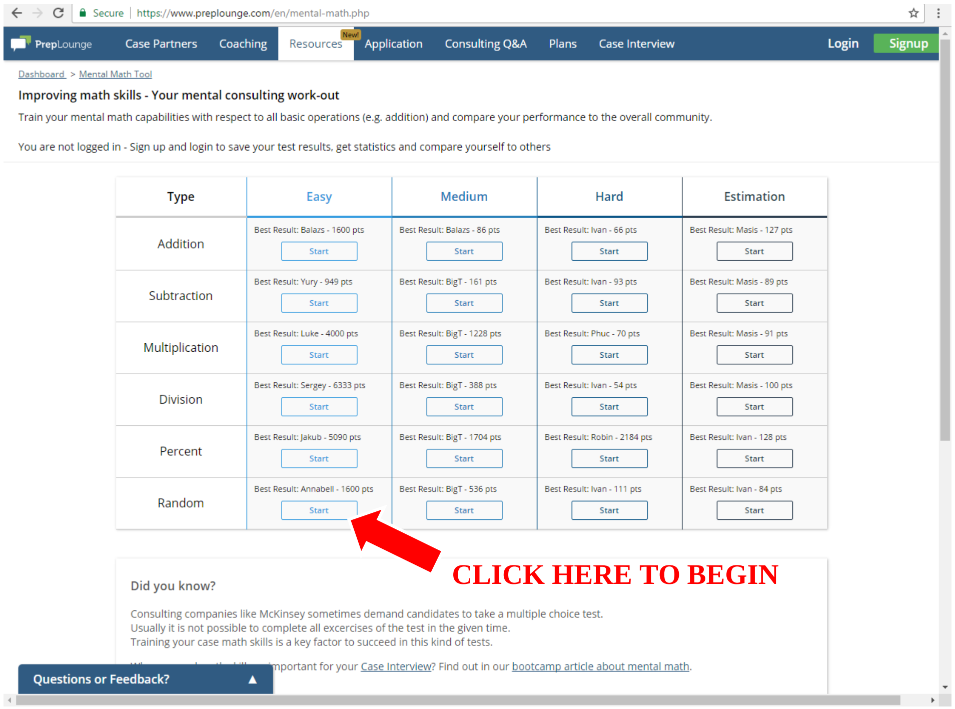

Pittsburgh Stress Battery

SHOW CARD #3 – Mirror Tracing Task

Image 1:

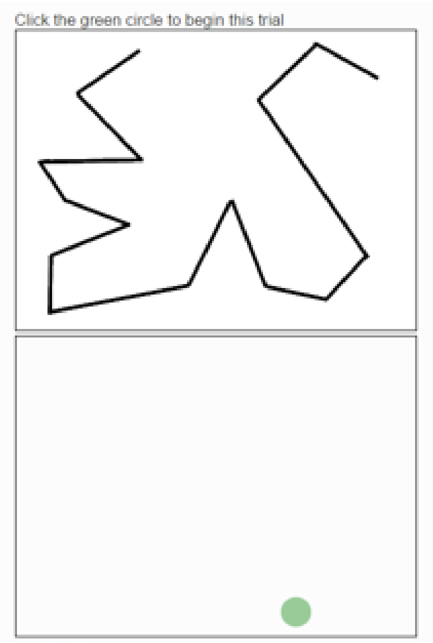

Image 2:

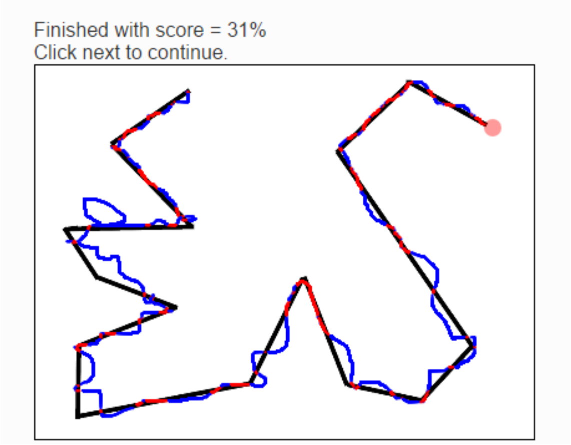

Pittsburgh Stress Battery – 30 MINUTE RECOVERY PERIOD

RA Script to be read to participants:

Great. You are all set with the stress tests. You can now go ahead and relax while I set up the next part of the study. One of the things we are interested in is how individuals recover from stress. While you are relaxing we are going to have you complete a few simple forms. While you complete the forms we will be taking your blood pressure and heart rate three more times – now, 15 minutes later, and 30 minutes later.

*Set up the participant with the below self-report forms. Before he/she begins, take the first of three recovery period readings (*Reading #5*). Start timer. Take two more readings at 15 minutes (*Reading #6*) and then 30 minutes (*Reading #7*).*

SELF REPORT FORMS TO BE COMPLETED DURING RECOVERY PERIOD:

FOOD FREQUENCY QUESTIONNAIRE: The first form we will have you complete is a Food Frequency Questionnaire that will ask you about the types of foods and drinks that you consume. It should take around 20 minutes to complete. Please let me know if you have any questions.

PROMIS Global Health Scale v.1.2 *– standardized scale (found on proceeding pages)*

SLEEP *– 1 question only taken from the Pittsburgh Sleep Quality Index*

International Physical Activity Questionnaire (IPAQ) – *standardized scale (found on proceeding pages)*

Mindful Attention Awareness Scale (MAAS) - *standardized scale (found on proceeding pages)*

Five Facet Mindfulness Questionnaire (FFMQ) - *standardized scale (found on proceeding pages)*

Commented [D6]: NEW measure. We replaced the food frequency questionnaire used in Stage 1 with a more up to date validated measure – the four page blue 2007 health grid from Harvard School of Public Health (see attachment).

Commented [D7]: NEW measure

Commented [D8]: Moved here from the online home survey.

Commented [D9]: NEW measure – this is to replace the physical activity questions asked in the online home survey used in Stage 1.

Commented [D10]: Moved here from the online home survey.

Commented [D11]: Moved here from the online home survey.

Global Health

Please respond to each question or statement by marking one box per row.

		Excellent	Very good	Good	Fair	Poor
Global01	In general, would you say your health is:	☐ 5	☐ 4	☐ 3	☐ 2	☐ 1
Global02	In general, would you say your quality of life is:..........	☐ 5	☐ 4	☐ 3	☐ 2	☐ 1
Global03	In general, how would you rate your physical health?	☐ 5	☐ 4	☐ 3	☐ 2	☐ 1
Global04	In general, how would you rate your mental health, including your mood and your ability to think?	☐ 5	☐ 4	☐ 3	☐ 2	☐ 1
Global05	In general, how would you rate your satisfaction with your social activities and relationships?	☐ 5	☐ 4	☐ 3	☐ 2	☐ 1
Global09r	In general, please rate how well you carry out your usual social activities and roles. (This includes activities at home, at work and in your community, and responsibilities as a parent, child, spouse, employee, friend, etc.)......	☐ 5	☐ 4	☐ 3	☐ 2	☐ 1
		**Completely**	**Mostly**	**Moderately**	**A little**	**Not at all**
Global06	To what extent are you able to carry out your everyday physical activities such as walking, climbing stairs, carrying groceries, or moving a chair?	☐ 5	☐ 4	☐ 3	☐ 2	☐ 1

© 2010-2016 PROMIS Health Organization and PROMIS Cooperative Group

In the past 7 days…

		Never	Rarely	Sometimes	Often	Always
Global10r	How often have you been bothered by emotional problems such as feeling anxious, depressed or irritable?	☐ 5	☐ 4	☐ 3	☐ 2	☐ 1
		**None**	**Mild**	**Moderate**	**Severe**	**Very severe**
Global08r	How would you rate your fatigue on average?	☐ 5	☐ 4	☐ 3	☐ 2	☐ 1

Global07r	How would you rate your pain on average?	☐ 0 No pain	☐ 1	☐ 2	☐ 3	☐ 4	☐ 5	☐ 6	☐ 7	☐ 8	☐ 9	☐ 10 Worst pain imaginable

SLEEP - The following question relates to your usual sleep habits during the past month only. Your answer should indicate the most accurate reply for the majority of days and nights in the past month.

SL1_04. **During the past month**, how many hours of actual sleep did you get on average at night? (This may be different than the number of hours you spent in bed.)

AVERAGE HOURS OF SL1_EEP PER NIGHT ___________

☐ I do not know

☐ I prefer not to answer

INTERNATIONAL PHYSICAL ACTIVITY QUESTIONNAIRE

We are interested in finding out about the kinds of physical activities that people do as part of their everyday lives. The questions will ask you about the time you spent being physically active in the **last 7 days**. Please answer each question even if you do not consider yourself to be an active person. Please think about the activities you do at work, as part of your house and yard work, to get from place to place, and in your spare time for recreation, exercise or sport.

Think about all the **vigorous** and **moderate** activities that you did in the **last 7 days**. **Vigorous** physical activities refer to activities that take hard physical effort and make you breathe much harder than normal. **Moderate** activities refer to activities that take moderate physical effort and make you breathe somewhat harder than normal.

PART 1: JOB-RELATED PHYSICAL ACTIVITY

The first section is about your work. This includes paid jobs, farming, volunteer work, course work, and any other unpaid work that you did outside your home. Do not include unpaid work you might do around your home, like housework, yard work, general maintenance, and caring for your family. These are asked in Part 3.

1. Do you currently have a job or do any unpaid work outside your home?

☐ ***Yes***

☐ No → ***Skip to PART 2: TRANSPORTATION***

The next questions are about all the physical activity you did in the **last 7 days** as part of your paid or unpaid work. This does not include traveling to and from work.

2. During the **last 7 days**, on how many days did you do **vigorous** physical activities like heavy lifting, digging, heavy construction, or climbing up stairs **as part of your work**? Think about only those physical activities that you did for at least 10 minutes at a time.

   _____ **days per week**

☐ No vigorous job-related physical activity → ***Skip to question 4***

3. How much time did you usually spend on one of those days doing **vigorous** physical activities as part of your work?

   _____ **hours per day**
   _____ **minutes per day**

4. Again, think about only those physical activities that you did for at least 10 minutes at a time. During the **last 7 days**, on how many days did you do **moderate** physical activities like carrying light loads **as part of your work**? Please do not include walking.

   _____ **days per week**

☐ No moderate job-related physical activity → ***Skip to question 6***

5. How much time did you usually spend on one of those days doing **moderate** physical activities as part of your work?

______ **hours per day**
______ **minutes per day**

6. During the **last 7 days**, on how many days did you **walk** for at least 10 minutes at a time **as part of your work**? Please do not count any walking you did to travel to or from work.

______ **days per week**

☐ No job-related walking → ***Skip to PART 2: TRANSPORTATION***

7. How much time did you usually spend on one of those days **walking** as part of your work?

______ **hours per day**
______ **minutes per day**

PART 2: TRANSPORTATION PHYSICAL ACTIVITY

These questions are about how you traveled from place to place, including to places like work, stores, movies, and so on.

8. During the **last 7 days**, on how many days did you **travel in a motor vehicle** like a train, bus, car, or tram?

______ **days per week**

☐ No traveling in a motor vehicle → ***Skip to question 10***

9. How much time did you usually spend on one of those days **traveling** in a train, bus, car, tram, or other kind of motor vehicle?

______ **hours per day**
______ **minutes per day**

Now think only about the **bicycling** and **walking** you might have done to travel to and from work, to do errands, or to go from place to place.

10. During the **last 7 days**, on how many days did you **bicycle** for at least 10 minutes at a time to go **from place to place**?

______ **days per week**

☐ No bicycling from place to place → ***Skip to question 12***

11. How much time did you usually spend on one of those days to **bicycle** from place to place?

_______ **hours per day**
_______ **minutes per day**

12. During the **last 7 days**, on how many days did you **walk** for at least 10 minutes at a time to go **from place to place**?

_______ **days per week**

☐ No walking from place to place → ***Skip to PART 3: HOUSEWORK, HOUSE MAINTENANCE, AND CARING FOR FAMILY***

13. How much time did you usually spend on one of those days walking from place to place?

_______ **hours per day**
_______ **minutes per day**

PART 3: HOUSEWORK, HOUSE MAINTENANCE, AND CARING FOR FAMILY

This section is about some of the physical activities you might have done in the **last 7 days** in and around your home, like housework, gardening, yard work, general maintenance work, and caring for your family.

14. Think about only those physical activities that you did for at least 10 minutes at a time. During the **last 7 days**, on how many days did you do **vigorous** physical activities like heavy lifting, chopping wood, shoveling snow, or digging **in the garden or yard**?

_______ **days per week**

☐ No vigorous activity in garden or yard → ***Skip to question 16***

15. How much time did you usually spend on one of those days doing **vigorous** physical activities in the garden or yard?

_______ **hours per day**
_______ **minutes per day**

16. Again, think about only those physical activities that you did for at least 10 minutes at a time. During the **last 7 days**, on how many days did you do **moderate** activities like carrying light loads, sweeping, washing windows, and raking **in the garden or yard**?

_______ **days per week**

☐ No moderate activity in garden or yard → ***Skip to question 18***

17. How much time did you usually spend on one of those days doing **moderate** physical activities in the garden or yard?

_____ **hours per day**
_____ **minutes per day**

18. Once again, think about only those physical activities that you did for at least 10 minutes at a time. During the **last 7 days**, on how many days did you do **moderate** activities like carrying light loads, washing windows, scrubbing floors and sweeping **inside your home**?

_____ **days per week**

☐ No moderate activity inside home → ***Skip to PART 4: RECREATION, SPORT AND LEISURE-TIME PHYSICAL ACTIVITY***

19. How much time did you usually spend on one of those days doing **moderate** physical activities inside your home?

_____ **hours per day**
_____ **minutes per day**

PART 4: RECREATION, SPORT, AND LEISURE-TIME PHYSICAL ACTIVITY

This section is about all the physical activities that you did in the **last 7 days** solely for recreation, sport, exercise or leisure. Please do not include any activities you have already mentioned.

20. Not counting any walking you have already mentioned, during the **last 7 days**, on how many days did you **walk** for at least 10 minutes at a time **in your leisure time**?

_____ **days per week**

☐ No walking in leisure time → ***Skip to question 22***

21. How much time did you usually spend on one of those days **walking** in your leisure time?

_____ **hours per day**
_____ **minutes per day**

22. Think about only those physical activities that you did for at least 10 minutes at a time. During the **last 7 days**, on how many days did you do **vigorous** physical activities like aerobics, running, fast bicycling, or fast swimming **in your leisure time**?

_____ **days per week**

☐ No vigorous activity in leisure time → ***Skip to question 24***

23. How much time did you usually spend on one of those days doing **vigorous** physical activities in your leisure time?

______ hours per day
______ minutes per day

24. Again, think about only those physical activities that you did for at least 10 minutes at a time. During the **last 7 days**, on how many days did you do **moderate** physical activities like bicycling at a regular pace, swimming at a regular pace, and doubles tennis **in your leisure time**?

______ days per week

☐ No moderate activity in leisure time → ***Skip to PART 5: TIME SPENT SITTING***

25. How much time did you usually spend on one of those days doing **moderate** physical activities in your leisure time?
______ hours per day
______ minutes per day

PART 5: TIME SPENT SITTING

The last questions are about the time you spend sitting while at work, at home, while doing course work and during leisure time. This may include time spent sitting at a desk, visiting friends, reading or sitting or lying down to watch television. Do not include any time spent sitting in a motor vehicle that you have already told me about.

26. During the **last 7 days**, how much time did you usually spend **sitting** on a **weekday**?

______ hours per day
______ minutes per day

27. During the **last 7 days**, how much time did you usually spend **sitting** on a **weekend day**?

______ hours per day
______ minutes per day

MAAS Instructions: Below is a collection of statements about your everyday experience. Using the scale below, please indicate how frequently or infrequently you currently have each experience. Please answer according to what really reflects your experience rather than what you think your experience should be. Please treat each item separately from every other item.

Please indicate the degree to which you agree with each of the following items using the scale below. Simply check your response to each item

	**Almost always**	**Very frequently**	**Somewhat frequently**	**Somewhat infrequently**	**Very infrequently**	**Almost never**
MA1_01. I could be experiencing some emotion and not be conscious of it until some time later.	☐	☐	☐	☐	☐	☐
MA1_02. I break or spill things because of carelessness, not paying attention, or thinking of something else.	☐	☐	☐	☐	☐	☐
MA1_03. I find it difficult to stay focused on what's happening in the present.	☐	☐	☐	☐	☐	☐
MA1_04. I tend to walk quickly to get where I'm going without paying attention to what I experience along the way.	☐	☐	☐	☐	☐	☐
MA1_05. I tend not to notice feelings of physical tension or discomfort until they really grab my attention.	☐	☐	☐	☐	☐	☐
MA1_06. I forget a person's name almost as soon as I've been told it for the first time.	☐	☐	☐	☐	☐	☐

	Almost always	Very frequently	Somewhat frequently	Somewhat infrequently	Very infrequently	Almost Never
MA1_07. It seems I am “running on automatic” without much awareness of what I’m doing.	☐	☐	☐	☐	☐	☐
MA1_08. I rush through activities without being really attentive to them.	☐	☐	☐	☐	☐	☐
MA1_09. I get so focused on the goal I want to achieve that I lose touch with what I am doing right now to get there.	☐	☐	☐	☐	☐	☐
MA1_10. I do jobs or tasks automatically, without being aware of what I’m doing.	☐	☐	☐	☐	☐	☐
MA1_11. I find myself listening to someone with one ear, doing something else at the same time.	☐	☐	☐	☐	☐	☐
MA1_12. I drive places on “automatic pilot” and then wonder why I went there.	☐	☐	☐	☐	☐	☐
MA1_13. I find myself preoccupied with the future or the past.	☐	☐	☐	☐	☐	☐
MA1_14. I find myself doing things without paying attention.	☐	☐	☐	☐	☐	☐
MA1_15. I snack without being aware that I’m eating.	☐	☐	☐	☐	☐	☐

FFMQ - Please rate each of the following statements using the scale provided. Write the number in the blank that best describes *your own opinion* of what is *generally true for you*.

	1 – Never or very rarely true	2 – Rarely true	3 – Sometimes true	4- Often true	5 – Very often or always true
FF1_01. When I'm walking, I deliberately notice the sensations of my body moving.	☐	☐	☐	☐	☐
FF1_02. I'm good at finding words to describe my feelings.	☐	☐	☐	☐	☐
FF1_03. I criticize myself for having irrational or inappropriate emotions.	☐	☐	☐	☐	☐
FF1_04. I perceive my feelings and emotions without having to react to them.	☐	☐	☐	☐	☐
FF1_05. When I do things, my mind wanders off and I'm easily distracted.	☐	☐	☐	☐	☐
FF1_06. When I take a shower or bath, I stay alert to the sensations of water on my body.	☐	☐	☐	☐	☐
FF1_07. I can easily put my beliefs, opinions, and expectations into words.	☐	☐	☐	☐	☐
FF1_08. I don't pay attention to what I'm doing because I'm daydreaming, worrying, or otherwise distracted.	☐	☐	☐	☐	☐
FF1_09. I watch my feelings without getting lost in them.	☐	☐	☐	☐	☐
FF1_10. I tell myself I shouldn't be feeling the way I'm feeling.	☐	☐	☐	☐	☐
FF1_11. I notice how foods and drinks affect my thoughts, bodily sensations, and emotions.	☐	☐	☐	☐	☐
FF1_12. It's hard for me to find the words to describe what I'm thinking.	☐	☐	☐	☐	☐
FF1_13. I am easily distracted.	☐	☐	☐	☐	☐
FF1_14. I believe some of my thoughts are abnormal or bad and I shouldn't think that way.	☐	☐	☐	☐	☐
FF1_15. I pay attention to sensations, such as the wind in my hair or sun on my face.	☐	☐	☐	☐	☐

	1 – Never or very rarely true	2 – Rarely true	3 – Sometimes true	4- Often true	5 – Very often or always true
FF1_16. I have trouble thinking of the right words to express how I feel about things.	☐	☐	☐	☐	☐
FF1_17. I make judgments about whether my thoughts are good or bad.	☐	☐	☐	☐	☐
FF1_18. I find it difficult to stay focused on what's happening in the present.	☐	☐	☐	☐	☐
FF1_19. When I have distressing thoughts or images, I "step back" and am aware of the thought or image without getting taken over by it.	☐	☐	☐	☐	☐
FF1_20. I pay attention to sounds, such as clocks ticking, birds chirping, or cars passing.	☐	☐	☐	☐	☐
FF1_21. In difficult situations, I can pause without immediately reacting.	☐	☐	☐	☐	☐
FF1_22. When I have a sensation in my body, it's difficult for me to describe it because I can't find the right words.	☐	☐	☐	☐	☐
FF1_23. It seems I am "running on automatic" without much aware- ness of what I'm doing.	☐	☐	☐	☐	☐
FF1_24. When I have distressing thoughts or images, I feel calm soon after.	☐	☐	☐	☐	☐
FF1_25. I tell myself that I shouldn't be thinking the way I'm thinking.	☐	☐	☐	☐	☐
FF1_26. I notice the smells and aromas of things.	☐	☐	☐	☐	☐
FF1_27. Even when I'm feeling terribly upset, I can find a way to put it into words.	☐	☐	☐	☐	☐
FF1_28. I rush through activities without being really attentive to them.	☐	☐	☐	☐	☐

	1 – Never or very rarely true	2 – Rarely true	3 – Sometimes true	4- Often true	5 – Very often or always true
FF1_29. When I have distressing thoughts or images, I am able just to notice them without reacting.	☐	☐	☐	☐	☐
FF1_30. I think some of my emotions are bad or inappropriate and I shouldn’t feel them.	☐	☐	☐	☐	☐
FF1_31. I notice visual elements in art or nature, such as colors, shapes, textures, or patterns of light and shadow.	☐	☐	☐	☐	☐
FF1_32. My natural tendency is to put my experiences into words.	☐	☐	☐	☐	☐
FF1_33. When I have distressing thoughts or images, I just notice them and let them go.	☐	☐	☐	☐	☐
FF1_34. I do jobs or tasks automatically without being aware of what I’m doing.	☐	☐	☐	☐	☐
FF1_35. When I have distressing thoughts or images, I judge myself as good or bad depending what the thought or image is about.	☐	☐	☐	☐	☐
FF1_36. I pay attention to how my emotions affect my thoughts and behavior.	☐	☐	☐	☐	☐
FF1_37. I can usually describe how I feel at the moment in consider- able detail.	☐	☐	☐	☐	☐
FF1_38. I find myself doing things without paying attention.	☐	☐	☐	☐	☐
FF1_39. I disapprove of myself when I have irrational ideas.	☐	☐	☐	☐	☐

After self-report questionnaires are complete, participants will be asked to perform the following three activities.

Heart Beat Detection Task (5 minutes)

Commented [SF12]: NEW measure

Open up the Kardia Mobile App. Have the participant sit in a chair with both feet on the ground and forearms on the table in front of him/her.

"We will now have you complete an activity where you will be asked to sit quietly and see if you can detect and count your heart beat. We will be using this ECG device."

Show participant the Kardia Mobile device and how it works. Demonstrate by completing a reading.

"We will have you complete this task three times at varying intervals. When I say 'begin' I want you to try your best to detect and count your heart beat. When I say 'stop' please let me know how many times you think your heart beat during the time interval. Try your best."

Have the participant place both hands on the Kardia Moble ECG device. Face the display screen away from the participant and towards yourself. Have the participant begin when the signal is strong and the timer begins. Have him/her stop when the timer ends. Record both the reading from the device and the particpant's estimated heart beat count. Repeat for three time intervals: 30 seconds, 30 seconds, 60 seconds. IMPORTANT: Do not let the participant know how long the time intervals are in length in order to deter guessing.

HEART BEAT DETECTION DATA SHEET (*example*)

INTERVAL	Heart Rate (Kardia Mobile)	# of Beats (calculation)	# of Beats (ppt count)
**1 – 30s**			
**2 – 30s**			
**3 – 60s**			

5-Trial Adjusting Delay Task (2-3 minutes)

Commented [SF13]: New Measure

"You will now complete a series of decision-making tasks. You will be asked to make choices between different amounts of money given to you now or after a delay. These are hypothetical choices, but please choose your answer as if the items were to be delivered as described. Each task will start with some brief instructions on the screen. Read these instructions, and press the 5 key on the keyboard when you are ready to begin. There are no right or wrong answers in the tasks, just choose which option you prefer in each case. Please take your time and answer thoughtfully. To select the option on the left side of the screen, press the left arrow, and to select the option on the right side of the screen, press the right arrow."

Sustained Attention to Response Task (15 minutes)

The Sustained Attention to Response Task (SART) is a computerized test of sustained attention, response inhibition (executive function) and self-regulation. Subjects are instructed to press a key in response to rapidly displayed integers (1-9) and withhold response to a designated "no-go" integer. SART errors consist of summed commission errors (button press on no-go trial) and omission errors (button not pressed on "go" integers). SART performance is associated with prefrontal cortex functioning, has been found to increase with mindfulness training and is correlated with scores on mindfulness questionnaires (specifically, the Mindful Attention Awareness Scale).

Attention Control: Attention Network Test (20 minutes)

Commented [SF14]: Dropped to reduce participant burden.

Attention Network Test (ANT) is a brief computerized battery measuring three independent behavioral components of attention: Conflict resolution (ability to overcome distracting stimuli), spatial Orienting (the benefit of valid spatial pre-cues), and Alerting (the benefit of temporal pre-cues). Efficiency of orienting is examined by changes in RT that accompany cues indicating where the target will occur. The efficiency of the executive conflict resolution network is examined by requiring the subject to respond by pressing two keys indicating the direction (left or right) of a central arrow surrounded by congruent, incongruent or neutral flankers. Moderate to high reliabilities are found for all networks.

Appendix 5

Home Baseline Assessment (v.2.0) –

with track changes

HOME BASELINE ASSESSMENT

QUESTIONNAIRES ANSWERED BY PARTICIPANTS

AT BASELINE (VIA ONLINE OR PAPER FORM)

Questionnaire Table of Contents

Introductory Questions....3
Background Questions....5
Eating Practices....10
Alcohol Consumption....13
Smoking....14
About You....15
Parent's Education....38
Your Childhood Experiences....40
Connor-Davidson Resilience Scale 10 (CD-RISC-10)....48
Chronic Illness....49
Blood Pressure and Blood Pressure Medication Use....50
More About You....51
Family History of Hypertension....55
END SCRIPT....56

Introduction

We appreciate you taking the time to participate in this research study. These questionnaires will ask a series of questions on various aspects of your health, health behaviours, family, and other life circumstances. It should take approximately one hour. Please keep in mind that you can refuse to answer any questions that you are not comfortable with.

Introductory Questions

IQ1_01. Please enter the 4-digit ID number you were given. ___ ___ ___ ___.

IQ1_02. What is your main reason for participating in this study?

IQ1_03. What do you care about most?

IQ1_04. What gives you the most pleasure in your life?

IQ1_05. What are your greatest worries?

Please list three personal goals you have for taking this mindfulness program:

PG1_01.____

PG1_02. ____

PG1_03. ____

Background Questions

~~BQ1_01. How many years old are you?~~

Comment [D1]: Already captured in SCR-I

BQ1_02. Are you Latino or Hispanic?

- ☐ No → ***skip to B3***
- ☐ Yes
- ☐ I do not know
- ☐ I prefer not to answer

B1_02a. Which of the following represents your family's country of origin? ***(check all that apply)***

- ☐ Cuba
- ☐ Mexico
- ☐ Puerto Rico
- ☐ Spain
- ☐ South America
- ☐ Columbia
- ☐ Dominican Republic
- ☐ Other Central American
- ☐ Other: ________________________
- ☐ I do not know
- ☐ I prefer not to answer

BQ1_03. If you were asked to put yourself into only one of these groups, in which one would you place yourself? ***(select <u>one</u> only):***

- ☐ Asian
- ☐ Pacific Islander
- ☐ African American/Black
- ☐ Caucasian/White
- ☐ Native American
- ☐ Other : _________________________
- ☐ I do not know
- ☐ I prefer not to answer

BQ1_04. Which of the following best describes your current work situation? ***(select <u>one</u> only)***

- ☐ Working full-time
- ☐ Working part-time
- ☐ Retired
- ☐ Unemployed: Looking for work
- ☐ Unemployed: Not currently looking for work
- ☐ Unemployed due to disability
- ☐ Keeping house or raising children full-time
- ☐ Military
- ☐ Full-time student
- ☐ Other:________________________
- ☐ I do not know
- ☐ I prefer not to answer

BQ1_05. What is the highest grade or level of regular school you have completed?

☐ Elementary School
☐ Junior High
☐ High School
☐ Associate degree (Junior College)

☐ College
☐ Graduate School
☐ Other: ______________________________

☐ I do not know
☐ I prefer not to answer

Comment [D2]: Reduced the education variables down to one question

~~BQ1_06. What is the highest degree you earned? ***(select one only)***~~

~~☐ Elementary school~~
~~☐ Some high school, but no GED~~
~~☐ GED~~
~~☐ High school~~
~~☐ Associate degree (Junior College)~~
~~☐ Bachelor's degree~~
~~☐ Master's degree~~
~~☐ Doctorate (PhD, EdD, etc)~~
~~☐ Professional (MD, JD, DDS, DVM, etc.)~~
~~☐ Other: ______________________________~~
~~☐ I do not know~~
~~☐ I prefer not to answer~~

~~BQ1_07. Did you ever attend any other school like a technical, vocational, or trade school?~~

~~☐ No~~
~~☐ Yes~~
~~☐ I do not know~~
~~☐ I prefer not to answer~~

~~BQ1_08. In total, about how many full-time years of education have you had, including 1st grade and all years of school after 1st grade?~~

~~________ years~~

BQ1_09a. Do you currently live alone?

- ☐ No
- ☐ Yes → ***skip to next section***
- ☐ I do not know
- ☐ I prefer not to answer

BQ1_09b. How many people currently live in your household, including yourself?

____

BQ1_09c. Of these people, how many are under 18?

____

BQ1_09d. Of the adults in your household (including yourself), how many bring income into the household?

____

Physical Activity

Comment [D3]: Moved to the In-Person baseline assessment.

Physical activities are activities where you move and increase your heart rate above its resting rate, whether you do them for pleasure, work, or transportation. The following questions ask about the amount and intensity of physical activity you usually do. The intensity of the activity is related to *the amount of energy* you use doing these activities.

Examples of physical activity intensity levels:

Light activities
Your heart beats slightly faster than normal
You can talk and sing

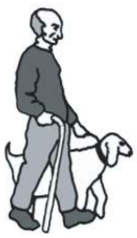
Light exercise

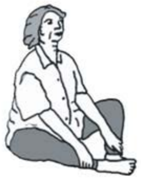
Light stretching

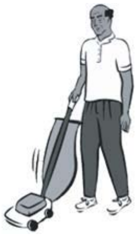
Light vacuuming or yard work

Moderate activities
Your heart beats faster than normal
You can talk but not sing

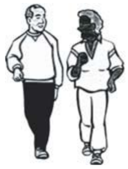
Brisk walking

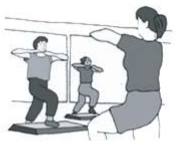
Aerobics class

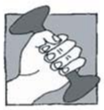
Strength training

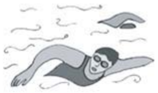
Swim gently

Vigorous activities
Your heart rate increases a lot
You can't talk, or your talking is broken up by large breaths

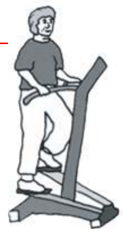
Aerobics classes

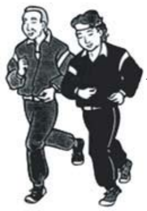
Jogging, Running, or Power Walking

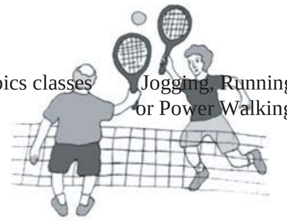
Singles tennis, Racquetball, Pickle ball

How physically active are you?

	YES	NO	I do not know	I prefer not to answer
PA1_01. I rarely or never do any physical	☐	☐	☐	☐

activities.				
PA1_02. I do some light and/or moderate physical activities, but not every week.	☐	☐	☐	☐
PA1_03. I do some light physical activity every week.	☐	☐	☐	☐
PA1_04. I do moderate physical activity every week but less than 5 days per week or less than 30 minutes on those days.	☐	☐	☐	☐
PA1_05. I do vigorous physical activities every week, but less than 3 days per week or less than 20 minutes on those days.	☐	☐	☐	☐
PA1_06. I do 30 minutes or more per day of moderate physical activities 5 or more days per week.	☐	☐	☐	☐
PA1_07. I do 20 minutes or more per day of vigorous physical activities 3 or more days per week.	☐	☐	☐	☐
PA1_08. I do activities to increase muscle strength, such as lifting weights or calisthenics, once a week or more.	☐	☐	☐	☐
PA1_09. I do activities to improve flexibility, such as stretching or yoga, once a week or more.	☐	☐	☐	☐

Eating Practices

	Definitely True	Mostly True	Mostly False	Definitely False
EE1_01. I deliberately take small helpings to control my weight.	☐	☐	☐	☐
EE1_02. I start to eat when I feel anxious.	☐	☐	☐	☐
EE1_03. Sometimes when I start eating, I just can't seem to stop.	☐	☐	☐	☐
EE1_04. When I feel sad, I often eat too much.	☐	☐	☐	☐
EE1_05. I don't eat some foods because they make me fat.	☐	☐	☐	☐
EE1_06. Being with someone who is eating, often makes me want to also eat.	☐	☐	☐	☐
EE1_07. When I feel tense or ''wound up'', I often feel I need to eat.	☐	☐	☐	☐
EE1_08. I often get so hungry that my stomach feels like a bottomless pit.	☐	☐	☐	☐
EE1_09. I'm always so hungry that it's hard for me to stop eating before finishing all of the food on my plate.	☐	☐	☐	☐
EE1_10. When I feel lonely, I console myself by eating.	☐	☐	☐	☐
EE1_11. I consciously hold back on how much I eat at meals to keep from gaining weight.	☐	☐	☐	☐
EE1_12. When I smell a sizzling steak or see a juicy piece of meat, I find it very difficult to keep from eating even if I've just finished a meal.	☐	☐	☐	☐
EE1_13. I'm always hungry enough to eat at any time.	☐	☐	☐	☐
EE1_14. If I feel nervous, I try to calm down by eating.	☐	☐	☐	☐
EE1_15. When I see something that looks very delicious, I often get so hungry that I have to eat right away.	☐	☐	☐	☐
EE1_16. When I feel depressed, I want to eat.	☐	☐	☐	☐

	Almost Never	Seldom	Usually	Almost Always
EE1_17. How often do you avoid ‘stocking up’’ on tempting foods?	☐	☐	☐	☐

	Unlikely	A little likely	Somewhat likely	Very likely
EE1_18. How likely are you to make an effort to eat less than you want?	☐	☐	☐	☐

	Never	Rarely	Sometimes	At least once a week
EE1_19. Do you go on eating binges even though you’re not hungry?	☐	☐	☐	☐

	Only at mealtimes	Sometimes between meals	Often between meals	Almost always
EE1_20. How often do you feel hungry?	☐	☐	☐	☐

	1	2	3	4	5	6	7	8
EE1_21. On a scale from 1 to 8, where 1 means no restraint in eating and 8 means total restraint, what number would you give yourself? Mark the number that best applies to you:	☐	☐	☐	☐	☐	☐	☐	☐

Table Salt Use:

TS1_01 Please report your average total use, during the past year, of "salt added at the table". Would you say…

- Never .... ☐
- Less than once per month .... ☐
- 1-3 shakes per month... ☐
- 1 shake per week .... ☐
- 2-4 shakes per week .... ☐
- 5-6 shakes per week .... ☐
- 1 shake per day .... ☐
- 2-3 shakes per day .... ☐
- 4-5 shakes per day .... ☐
- 6+ shakes per day .... ☐

Alcohol Consumption

A drink of alcohol is defined as 1 can or bottle of beer, 1 glass of wine, 1 can or bottle of wine cooler, 1 cocktail, or 1 shot of liquor.

AC1_01. During the past 30 days, how many days per week or per month did you have at least 1 drink of any alcoholic beverage? [if none, ***skip to next section***]

____

AC1_02. On the days when you drank, about how many drinks did you drink on average?

____

AC1_03. ***Men:*** Considering all types of alcoholic beverages, how many times during the past 30 days did you have **5** or more drinks on an occasion?

Women: Considering all types of alcoholic beverages, how many times during the past 30 days did you have ***4*** or more drinks on an occasion?

____

Smoking

SM1_01. Have you smoked at least 100 cigarettes in your entire life?

- ☐ Yes
- ☐ No
- ☐ I Do Not Know
- ☐ Prefer not to answer

SM1_02. Did you ever become a daily smoker (that is, smoke every day or nearly every day for two months or longer)?

- ☐ Yes
- ☐ No → ***skip to next section***
- ☐ I Do Not Know
- ☐ Prefer not to answer

SM1_03. How old were you when you last smoked daily?

Age ______ (in years)

☐ I Do Not Know ☐ Prefer not to answer ☐ Still smoking daily

SM1_04. Do you smoke cigarettes now?

- ☐ Yes
- ☐ No → ***skip to next section***
- ☐ I Do Not Know
- ☐ Prefer not to answer

SM1_04a. How many cigarettes per day do you smoke? (One pack equals 20 cigarettes)

Number of cigarettes ________

- ☐ I Do Not Know
- ☐ Prefer not to answer

About You

Please bring to mind a type of very tasty food that may contribute to hypertension through high salt intake or through eating too many calories (e.g., sweet sugary dessert, salty snack foods, etc.).

Think about the LAST WEEK you MOST WANTED this type of food. For each item, select a number (0 to 10) to indicate your rating.

At that time…	**Not at All 0**	**1**	**2**	**3**	**4**	**5**	**6**	**7**	**8**	**9**	**Extremely 10**
1. …how much did you want it?	☐	☐	☐	☐	☐	☐	☐	☐	☐	☐	☐
2. …how much did you need it?	☐	☐	☐	☐	☐	☐	☐	☐	☐	☐	☐
3. …how strong was the urge to have it?	☐	☐	☐	☐	☐	☐	☐	☐	☐	☐	☐
At that time, how vividly did you…											
4. …picture it?	☐	☐	☐	☐	☐	☐	☐	☐	☐	☐	☐
5. …imagine its taste?	☐	☐	☐	☐	☐	☐	☐	☐	☐	☐	☐
6. …imagine its smell?	☐	☐	☐	☐	☐	☐	☐	☐	☐	☐	☐
7. …imagine what it would feel like in your mouth or throat?	☐	☐	☐	☐	☐	☐	☐	☐	☐	☐	☐
8. …imagine how your body would feel?	☐	☐	☐	☐	☐	☐	☐	☐	☐	☐	☐
At that time…											
9. …how hard were you trying not to think about it?	☐	☐	☐	☐	☐	☐	☐	☐	☐	☐	☐
10. …how intrusive were the thoughts?	☐	☐	☐	☐	☐	☐	☐	☐	☐	☐	☐
11. …how hard was it to think about anything else?	☐	☐	☐	☐	☐	☐	☐	☐	☐	☐	☐

Please bring to mind any times in the LAST WEEK when you had a desire to do sedentary activities (e.g., read a book, watch a movie, be on the computer, etc.) instead of physical activities (e.g., walking, gardening, exercise).

Think about the LAST WEEK you MOST WANTED to do a sedentary activity. For each item, select a number (0 to 10) to indicate your rating.

At that time…	**Not at All 0**	**1**	**2**	**3**	**4**	**5**	**6**	**7**	**8**	**9**	**Extremely 10**
1. …how much did you want it?	☐	☐	☐	☐	☐	☐	☐	☐	☐	☐	☐
2. …how much did you need it?	☐	☐	☐	☐	☐	☐	☐	☐	☐	☐	☐
3. …how strong was the urge to have it?	☐	☐	☐	☐	☐	☐	☐	☐	☐	☐	☐
At that time, how vividly did you…											
4. …picture it?	☐	☐	☐	☐	☐	☐	☐	☐	☐	☐	☐
5. …imagine how your body would feel?	☐	☐	☐	☐	☐	☐	☐	☐	☐	☐	☐
At that time…											
6. …how hard were you trying not to think about it?	☐	☐	☐	☐	☐	☐	☐	☐	☐	☐	☐
7. …how intrusive were the thoughts?	☐	☐	☐	☐	☐	☐	☐	☐	☐	☐	☐
8. …how hard was it to think about anything else?	☐	☐	☐	☐	☐	☐	☐	☐	☐	☐	☐

Please bring to mind any times in the LAST WEEK when you had a desire to drink alcohol, such as wine, beer or spirits.

Think about the LAST WEEK you MOST WANTED alcohol. For each item, select a number (0 to 10) to indicate your rating.

At that time…	**Not at All 0**	**1**	**2**	**3**	**4**	**5**	**6**	**7**	**8**	**9**	**Extremely 10**
1. …how much did you want it?	☐	☐	☐	☐	☐	☐	☐	☐	☐	☐	☐
2. …how much did you need it?	☐	☐	☐	☐	☐	☐	☐	☐	☐	☐	☐
3. …how strong was the urge to have it?	☐	☐	☐	☐	☐	☐	☐	☐	☐	☐	☐
At that time, how vividly did you…											
4. …picture it?	☐	☐	☐	☐	☐	☐	☐	☐	☐	☐	☐
5. …imagine its taste?	☐	☐	☐	☐	☐	☐	☐	☐	☐	☐	☐
6. …imagine its smell?	☐	☐	☐	☐	☐	☐	☐	☐	☐	☐	☐
7. …imagine what it would feel like in your mouth or throat?	☐	☐	☐	☐	☐	☐	☐	☐	☐	☐	☐
8. …imagine how your body would feel?	☐	☐	☐	☐	☐	☐	☐	☐	☐	☐	☐
At that time…											
9. …how hard were you trying not to think about it?	☐	☐	☐	☐	☐	☐	☐	☐	☐	☐	☐
10. …how intrusive were the thoughts?	☐	☐	☐	☐	☐	☐	☐	☐	☐	☐	☐
11. …how hard was it to think about anything else?	☐	☐	☐	☐	☐	☐	☐	☐	☐	☐	☐

Using the scale provided, please indicate how much each of the following statements reflects how you typically are.

	Not at all	A little	Somewhat	Fair Amount	Very much
SC1_01. I am good at resisting temptation.	☐	☐	☐	☐	☐
SC1_02. I have a hard time breaking bad habits.	☐	☐	☐	☐	☐
SC1_03. I am lazy.	☐	☐	☐	☐	☐
SC1_04. I say inappropriate things.	☐	☐	☐	☐	☐
SC1_05. I do certain things that are bad for me, if they are fun.	☐	☐	☐	☐	☐
SC1_06. I refuse things that are bad for me.	☐	☐	☐	☐	☐
SC1_07. I wish I had more self-discipline.	☐	☐	☐	☐	☐
SC1_08. People would say that I have iron self-discipline.	☐	☐	☐	☐	☐
SC1_09. Pleasure and fun sometimes keep me from getting work done.	☐	☐	☐	☐	☐
SC1_10. I have trouble concentrating.	☐	☐	☐	☐	☐
SC1_11. I am able to work effectively toward long-term goals.	☐	☐	☐	☐	☐
SC1_12. Sometimes I can't stop myself from doing something, even if I know it is wrong.	☐	☐	☐	☐	☐
SC1_13. I often act without thinking through all the alternatives.	☐	☐	☐	☐	☐

Please read each statement carefully before answering. To the left of each item, indicate how often you behave in the stated manner, using the following scale:

	Almost never	Not very often	Sometimes	Frequently	Almost always
CO1_O1. When I fail at something important to me, I become consumed by feelings of inadequacy.					
CO1_02. I try to be understanding and patient towards those aspects of my personality I don't like.					
CO1_O3. When something painful happens I try to take a balanced view of the situation.					
CO1_O4. When I'm feeling down, I tend to feel like most other people are probably happier than I am.					
CO1_O5. I try to see my failings as part of the human condition.					
CO1_O6. When I'm going through a very hard time, I give myself the caring and tenderness I need.					
CO1_O7. When something upsets me I try to keep my emotions in balance.					
CO1_O8. When I fail at something that's important to me, I tend to feel alone in my failure					
CO1_O9. When I'm feeling down I tend to obsess and fixate on everything that's wrong.					

	Almost never	Not very often	Sometimes	Frequently	Almost always
CO1_10. When I feel inadequate in some way, I try to remind myself that feelings of inadequacy are shared by most people.					
CO1_11. I'm disapproving and judgmental about my own flaws and inadequacies.					
CO1_12. I'm intolerant and impatient towards those aspects of my personality I don't like.					

The questions in this scale ask you about your feelings and thoughts during the last month. In each case, you will be asked to indicate by circling how often you felt or thought a certain way. Although some of the questions are similar, there are differences between them and you should treat each one as a separate question. The best approach is to answer fairly quickly. That is, don't try to count up the number of times you felt a particular way, but rather indicate the alternative that seems like a reasonable estimate.

Comment [D4]: Replaced the 10-item PSS with the 14-item PSS.

	Never<br>0	Almost Never<br>1	Sometimes<br>2	Fairly Often<br>3	Very Often<br>4
**1. In the last month, how often have you been upset because of something that happened unexpectedly?**	○	○	○	○	○
**2. In the last month, how often have you felt that you were unable to control the important things in your life?**	○	○	○	○	○
**3. In the last month, how often have you felt nervous and "stressed"?**	○	○	○	○	○
**4. In the last month, how often have you dealt successfully with day to day problems and annoyances?**	○	○	○	○	○
**5. In the last month, how often have you felt that you were effectively coping with important changes that were occurring in your life?**	○	○	○	○	○
**6. In the last month, how often have you felt confident about your ability to handle your personal problems?**	○	○	○	○	○
**7. In the last month, how often have you felt that things were going your way?**	○	○	○	○	○
**8. In the last month, how often have you found that you could not cope with all the things that you had to do?**	○	○	○	○	○
**9. In the last month, how often have you been able to control irritations in your life?**	○	○	○	○	○
**10. In the last month, how often have you felt that you were on top of things?**	○	○	○	○	○

	Never	Almost Never	Sometimes	Fairly Often	Very Often
	0	1	2	3	4
**11. In the last month, how often have you been angered because of things that happened that were outside of your control?**	○	○	○	○	○
**12. In the last month, how often have you found yourself thinking about things that you have to accomplish?**	○	○	○	○	○
**13. In the last month, how often have you been able to control the way you spend your time?**	○	○	○	○	○
**14. In the last month, how often have you felt difficulties were piling up so high that you could not overcome them?**	○	○	○	○	○

Please indicate how often the following statements apply to you by checking the box that best describes your experience.

	Almost Never (0-10%)	Sometimes (11-35%)	About Half The Time (36-65%)	Most of the Time (66-90%)	Almost Always (91-100%)
ER1_01. I am clear about my feelings.	☐	☐	☐	☐	☐
ER1_02. I pay attention to how I feel.	☐	☐	☐	☐	☐
ER1_03. I experience my emotions as overwhelming and out of control.	☐	☐	☐	☐	☐
ER1_04. I have no idea how I am feeling.	☐	☐	☐	☐	☐
ER1_05. I have difficulty making sense out of my feelings.	☐	☐	☐	☐	☐
ER1_06. I am attentive to my feelings.	☐	☐	☐	☐	☐
ER1_07. I know exactly how I am feeling.	☐	☐	☐	☐	☐
ER1_08. I care about what I am feeling.	☐	☐	☐	☐	☐
ER1_09. I am confused about how I feel.	☐	☐	☐	☐	☐
ER1_10. When I'm upset, I acknowledge my emotions.	☐	☐	☐	☐	☐
ER1_11. When I'm upset, I become angry with myself for feeling that way.	☐	☐	☐	☐	☐
ER1_12. When I'm upset, I become embarrassed for feeling that way.	☐	☐	☐	☐	☐

	Almost Never (0-10%)	Sometimes (11-35%)	About Half The Time (36-65%)	Most of the Time (66-90%)	Almost Always (91-100%)
ER1_13. When I'm upset, I have difficulty getting work done.	☐	☐	☐	☐	☐
ER1_14. When I'm upset, I become out of control.	☐	☐	☐	☐	☐
ER1_15. When I'm upset, I believe that I will remain that way for a long time.	☐	☐	☐	☐	☐
ER1_16. When I'm upset, I believe that I will end up feeling very depressed.	☐	☐	☐	☐	☐
ER1_17. When I'm upset, I believe that my feelings are valid and important.	☐	☐	☐	☐	☐
ER1_18. When I'm upset, I have difficulty focusing on other things.	☐	☐	☐	☐	☐
ER1_19. When I'm upset, I feel out of control.	☐	☐	☐	☐	☐
ER1_20. When I'm upset, I can still get things done.	☐	☐	☐	☐	☐
ER1_21. When I'm upset, I feel ashamed at myself for feeling that way.	☐	☐	☐	☐	☐
ER1_22. When I'm upset, I know that I can find a way to eventually feel better.	☐	☐	☐	☐	☐

	Almost Never (0-10%)	Sometimes (11-35%)	About Half The Time (36-65%)	Most of the Time (66-90%)	Almost Always (91-100%)
ER1_23. When I'm upset, I feel like I am weak.	☐	☐	☐	☐	☐
ER1_24. When I'm upset, I feel like I can remain in control of my behaviours.	☐	☐	☐	☐	☐
ER1_25. When I'm upset, I feel guilty for feeling that way.	☐	☐	☐	☐	☐
ER1_26. When I'm upset, I have difficulty concentrating.	☐	☐	☐	☐	☐
ER1_27. When I'm upset, I have difficulty controlling my behaviours.	☐	☐	☐	☐	☐
ER1_28. When I'm upset, I believe there is nothing I can do to make myself feel better.	☐	☐	☐	☐	☐
ER1_29. When I'm upset, I become irritated at myself for feeling that way.	☐	☐	☐	☐	☐
ER1_30. When I'm upset, I start to feel very bad about myself.	☐	☐	☐	☐	☐
ER1_31. When I'm upset, I believe that wallowing in it is all I can do.	☐	☐	☐	☐	☐
ER1_32. When I'm upset, I lose control over my behaviour.	☐	☐	☐	☐	☐

	Almost Never (0-10%)	Sometimes (11-35%)	About Half The Time (36-65%)	Most of the Time (66-90%)	Almost Always (91-100%)
ER1_33. When I'm upset, I have difficulty thinking about anything else.	☐	☐	☐	☐	☐
ER1_34. When I'm upset I take time to figure out what I'm really feeling.	☐	☐	☐	☐	☐
ER1_35. When I'm upset, it takes me a long time to feel better.	☐	☐	☐	☐	☐
ER1_36. When I'm upset, my emotions feel overwhelming.	☐	☐	☐	☐	☐

This scale is made up of a list of statements each of which may or may not be true about you. For each statement, select “definitely true” if you are sure it is true about you and “probably true” if you think it is true but are not absolutely certain. Similarly, you should select “definitely false” if you are sure the statement is false and “probably false” if you think it is false but are not absolutely certain.

	Definitely False	Probably False	Probably True	Definitely True
**IS1_01** If I wanted to go on a trip for a day (for example, to the country or mountains), I would have a hard time finding someone to go with me.	☐	☐	☐	☐
**IS1_02** I feel that there is no one I can share my most private worries and fears with.	☐	☐	☐	☐
**IS1_03** If I were sick, I could easily find someone to help me with my daily chores.	☐	☐	☐	☐
**IS1_04** There is someone I can turn to for advice about handling problems with my family.	☐	☐	☐	☐
**IS1_05** If I decide one afternoon that I would like to go to a movie that evening, I could easily find someone to go with me.	☐	☐	☐	☐
**IS1_06** When I need suggestions on how to deal with a personal problem, I know someone I can turn to.	☐	☐	☐	☐
**IS1_07** I don’t often get invited to do things with others.	☐	☐	☐	☐
**IS1_08** If I had to go out of town for a few weeks, it would be difficult to find someone who would look after my house or apartment (the plants, pets, garden, etc.).	☐	☐	☐	☐
**IS1_09** If I wanted to have lunch with someone, I could easily find someone to join me.	☐	☐	☐	☐

	Definitely False	Probably False	Probably True	Definitely True
**IS1_10** If I was stranded 10 miles from home, there is someone I could call who could come and get me.	☐	☐	☐	☐
**IS1_11** If a family crisis arose, it would be difficult to find someone who could give me good advice about how to handle it.	☐	☐	☐	☐
**IS1_12** If I needed some help in moving to a new house or apartment, I would have a hard time finding someone to help me.	☐	☐	☐	☐

Please indicate how often each of the statements below is descriptive of you.

**Statement**	Never	Rarely	Sometimes	Often
LS1_01. I feel in tune with the people around me	◻	◻	◻	◻
LS1_02. I lack companionship	◻	◻	◻	◻
LS1_03. There is no one I can turn to	◻	◻	◻	◻
LS1_04. I do not feel alone	◻	◻	◻	◻
LS1_05. I feel part of a group of friends	◻	◻	◻	◻
LS1_06. I have a lot in common with the people around me	◻	◻	◻	◻
LS1_07. I am no longer close to anyone	◻	◻	◻	◻
LS1_08. My interests and ideas are not shared by those around me	◻	◻	◻	◻
LS1_09. I am an outgoing person	◻	◻	◻	◻
LS1_10. There are people I feel close to	◻	◻	◻	◻
LS1_11. I feel left out	◻	◻	◻	◻
LS1_12. My social relationships are superficial	◻	◻	◻	◻
LS1_13. No one really knows me well	◻	◻	◻	◻
LS1_14. I feel isolated from others	◻	◻	◻	◻
LS1_15. I can find companionship when I want it	◻	◻	◻	◻
LS1_16. There are people who really understand me	◻	◻	◻	◻
LS1_17. I am unhappy being so withdrawn	◻	◻	◻	◻
LS1_18. People are around me but not with me	◻	◻	◻	◻
LS1_19. There are people I can talk to	◻	◻	◻	◻
LS1_20. There are people I can turn to	◻	◻	◻	◻

Below you will find a list of statements. Please indicate how often each statement applies to you generally in daily life.

	0- Never	1	2	3	4	5 - Always
IA1_01. When I am tense I notice where the tension is located in my body.	☐	☐	☐	☐	☐	☐
IA1_02. I notice when I am uncomfortable in my body.	☐	☐	☐	☐	☐	☐
IA1_03. I notice where in my body I am comfortable.	☐	☐	☐	☐	☐	☐
IA1_04. I notice changes in my breathing, such as whether it slows down or speeds up.	☐	☐	☐	☐	☐	☐
IA1_05. I do not notice (I ignore) physical tension or discomfort<br>until they become more severe.	☐	☐	☐	☐	☐	☐
IA1_06. I distract myself from sensations of discomfort.	☐	☐	☐	☐	☐	☐
IA1_07. When I feel pain or discomfort, I try to power through it.	☐	☐	☐	☐	☐	☐
IA1_08. When I feel physical pain, I become upset.	☐	☐	☐	☐	☐	☐
IA1_09. I start to worry that something is wrong if I feel any discomfort.	☐	☐	☐	☐	☐	☐
IA1_10. I can notice an unpleasant body sensation without worrying about it.	☐	☐	☐	☐	☐	☐
IA1_11. I can pay attention to my breath without being distracted by things happening around me.	☐	☐	☐	☐	☐	☐
IA1_12. I can maintain awareness of my inner bodily sensations even when there is a lot going on around me.	☐	☐	☐	☐	☐	☐
IA1_13. When I am in conversation with someone, I can pay attention to my posture.	☐	☐	☐	☐	☐	☐
IA1_14. I can return awareness to my body if I am distracted.	☐	☐	☐	☐	☐	☐
IA1_15. I can refocus my attention from thinking to sensing my body.	☐	☐	☐	☐	☐	☐
IA1_16. I can maintain awareness of my whole body even when a part of me is in pain or discomfort.	☐	☐	☐	☐	☐	☐
IA1_17. I am able to consciously focus on my body as a whole.	☐	☐	☐	☐	☐	☐
IA1_18. I notice how my body changes when I am angry.	☐	☐	☐	☐	☐	☐
IA1_19. When something is wrong in my life I can feel it in my body.	☐	☐	☐	☐	☐	☐
IA1_20. I notice that my body feels different after a peaceful experience.	☐	☐	☐	☐	☐	☐
IA1_21. I notice that my breathing becomes free and easy when I feel comfortable.	☐	☐	☐	☐	☐	☐
IA1_22. I notice how my body changes when I feel happy / joyful.	☐	☐	☐	☐	☐	☐

	0- Never	1	2	3	4	5 - Always
IA1_23. When I feel overwhelmed I can find a calm place inside.	☐	☐	☐	☐	☐	☐
IA1_24. When I bring awareness to my body I feel a sense of calm.	☐	☐	☐	☐	☐	☐
IA1_25. I can use my breath to reduce tension.	☐	☐	☐	☐	☐	☐
IA1_26. When I am caught up in thoughts, I can calm my mind by focusing on my body/breathing.	☐	☐	☐	☐	☐	☐
IA1_27. I listen for information from my body about my emotional state.	☐	☐	☐	☐	☐	☐
IA1_28. When I am upset, I take time to explore how my body feels.	☐	☐	☐	☐	☐	☐
IA1_29. I listen to my body to inform me about what to do.	☐	☐	☐	☐	☐	☐
IA1_30. I am at home in my body.	☐	☐	☐	☐	☐	☐
IA1_31. I feel my body is a safe place.	☐	☐	☐	☐	☐	☐
IA1_32. I trust my body sensations.	☐	☐	☐	☐	☐	☐

We are interested in your recent experiences. Below is a list of things that people sometimes experience. Next to each item are five choices: "never", "rarely", "sometimes", "often", and "all the time". Please choose one of these to indicate how much you currently have experiences similar to those described.

Please do not spend too long on each item–it is your first response that we are interested in. Please be sure to answer every item.

	Never	Rarely	Sometimes	Often	All the time
RD1_01. I think about what will happen in the future.	☐	☐	☐	☐	☐
RD1_02. I remind myself that thoughts aren't facts.	☐	☐	☐	☐	☐
RD1_03. I am better able to accept myself as I am.	☐	☐	☐	☐	☐
RD1_04. I notice all sorts of little things and details in the world around me.	☐	☐	☐	☐	☐
RD1_05. I am kinder to myself when things go wrong.	☐	☐	☐	☐	☐
RD1_06. I can slow my thinking at times of stress.	☐	☐	☐	☐	☐
RD1_07. I wonder what kind of person I really am.	☐	☐	☐	☐	☐
RD1_08. I am not so easily carried away by my thoughts and feelings.	☐	☐	☐	☐	☐
RD1_09. I notice that I don't take difficulties so personally.	☐	☐	☐	☐	☐
RD1_10. I can separate myself from my thoughts and feelings.	☐	☐	☐	☐	☐
RD1_11. I analyze why things turn out the way they do.	☐	☐	☐	☐	☐
RD1_12. I can take time to respond to difficulties.	☐	☐	☐	☐	☐
RD1_13. I think over and over again about what others have said to me.	☐	☐	☐	☐	☐
RD1_14. I can treat myself kindly.	☐	☐	☐	☐	☐
RD1_15. I can observe unpleasant feelings without being drawn into them.	☐	☐	☐	☐	☐
RD1_16. I have the sense that I am fully aware of what is going on around me and inside me.	☐	☐	☐	☐	☐
RD1_17. I can actually see that I am not my thoughts.	☐	☐	☐	☐	☐
RD1_18. I am consciously aware of a sense of my body as a whole.	☐	☐	☐	☐	☐
RD1_19. I think about the ways in which I am different from other people.	☐	☐	☐	☐	☐
RD1_20. I view things from a wider perspective.	☐	☐	☐	☐	☐

How do you cope with events? Everyone gets confronted with negative or unpleasant events now and then and everyone responds to them in his or her own way. By the following questions you are asked to indicate what you generally think, when you experience negative or unpleasant events.

	(Almost) Never	Some-times	Regularly	Often	(Almost) Always
CR1_01. I think that I have to accept that this has happened.	□	□	□	□	□
CR1_02. I often think about how I feel about what I have experienced.	□	□	□	□	□
CR1_03. I think I can learn something from the situation.	□	□	□	□	□
CR1_04. I feel that I am the one who is responsible for what has happened.	□	□	□	□	□
CR1_05. I think that I have to accept the situation.	□	□	□	□	□
CR1_06. I am preoccupied with what I think and feel about what I have experienced.	□	□	□	□	□
CR1_07. I think of pleasant things that have nothing to do with it.	□	□	□	□	□
CR1_08. I think that I can become a stronger person as a result of what has happened.	□	□	□	□	□
CR1_09. I keep thinking about how terrible it is what I have experienced.	□	□	□	□	□
CR1_10. I feel that others are responsible for what has happened.	□	□	□	□	□
CR1_11. I think of something nice instead of what has happened.	□	□	□	□	□
CR1_12. I think about how to change the situation.	□	□	□	□	□
CR1_13. I think that it hasn't been too bad compared to other things.	□	□	□	□	□
CR1_14. I think that basically the cause must lie within myself.	□	□	□	□	□
CR1_15. I think about a plan of what I can do best.	□	□	□	□	□
CR1_16. I tell myself that there are worse things in life.	□	□	□	□	□
CR1_17. I continually think how horrible the situation has been.	□	□	□	□	□
CR1_18. I feel that basically the cause lies with others.	□	□	□	□	□

Instructions: Below is a collection of statements about your everyday experience. Using the scale below, please indicate how frequently or infrequently you currently have each experience. Please answer according to what really reflects your experience rather than what you think your experience should be. Please treat each item separately from every other item.

Please indicate the degree to which you agree with each of the following items using the scale below. Simply check your response to each item

Comment [D5]: Moved to the In-Person Baseline assessment

-	Almost always	Very frequently	Somewhat frequently	Somewhat infrequently	Very infrequently	Almost never	I do not know	I prefer not to answer
MA1_01. I could be experiencing some emotion and not be conscious of it until some time later.	☐	☐	☐	☐	☐	☐	☐	☐
MA1_02. I break or spill things because of carelessness, not paying attention, or thinking of something else.	☐	☐	☐	☐	☐	☐	☐	☐
MA1_03. I find it difficult to stay focused on what's happening in the present.	☐	☐	☐	☐	☐	☐	☐	☐
MA1_04. I tend to walk quickly to get where I'm going without paying attention to what I experience along the way.	☐	☐	☐	☐	☐	☐	☐	☐
MA1_05. I tend not to notice feelings of physical tension or discomfort until they really grab my attention.	☐	☐	☐	☐	☐	☐	☐	☐
MA1_06. I forget a person's name almost as soon as I've been told it for the first time.	☐	☐	☐	☐	☐	☐	☐	☐

	Almost always	Very frequently	Somewhat frequently	Somewhat infrequently	Very infrequently	Almost never	I do not know	I prefer not to answer
MA1_07. It seems I am "running on automatic" without much awareness of what I'm doing.	☐	☐	☐	☐	☐	☐	☐	☐
MA1_08. I rush through activities without being really attentive to them.	☐	☐	☐	☐	☐	☐	☐	☐
MA1_09. I get so focused on the goal I want to achieve that I lose touch with what I am doing right now to get there.	☐	☐	☐	☐	☐	☐	☐	☐
MA1_10. I do jobs or tasks automatically, without being aware of what I'm doing.	☐	☐	☐	☐	☐	☐	☐	☐
MA1_11. I find myself listening to someone with one ear, doing something else at the same time.	☐	☐	☐	☐	☐	☐	☐	☐
MA1_12. I drive places on "automatic pilot" and then wonder why I went there.	☐	☐	☐	☐	☐	☐	☐	☐
MA1_13. I find myself preoccupied with the future or the past.	☐	☐	☐	☐	☐	☐	☐	☐
MA1_14. I find myself doing things without paying attention.	☐	☐	☐	☐	☐	☐	☐	☐
MA1_15. I snack without being aware that I'm eating.	☐	☐	☐	☐	☐	☐	☐	☐

Please rate each of the following statements using the scale provided. Write the number in the blank that best describes *your own opinion* of what is *generally true for you.*

Comment [D6]: Moved to the In-Person Baseline assessment

	Never	Almost Never	Sometimes	Fairly Often	Often	I do not know	I prefer not to answer
FF1_01. When I'm walking, I deliberately notice the sensations of my body moving.	☐	☐	☐	☐	☐	☐	☐
FF1_02. I'm good at finding words to describe my feelings.	☐	☐	☐	☐	☐	☐	☐
FF1_03. I criticize myself for having irrational or inappropriate emotions.	☐	☐	☐	☐	☐	☐	☐
FF1_04. I perceive my feelings and emotions without having to react to them.	☐	☐	☐	☐	☐	☐	☐
FF1_05. When I do things, my mind wanders off and I'm easily distracted.	☐	☐	☐	☐	☐	☐	☐
FF1_06. When I take a shower or bath, I stay alert to the sensations of water on my body.	☐	☐	☐	☐	☐	☐	☐
FF1_07. I can easily put my beliefs, opinions, and expectations into words.	☐	☐	☐	☐	☐	☐	☐
FF1_08. I don't pay attention to what I'm doing because I'm daydreaming, worrying, or otherwise distracted.	☐	☐	☐	☐	☐	☐	☐
FF1_09. I watch my feelings without getting lost in them.	☐	☐	☐	☐	☐	☐	☐
FF1_10. I tell myself I shouldn't be feeling the way I'm feeling.	☐	☐	☐	☐	☐	☐	☐
FF1_11. I notice how foods and drinks affect my thoughts, bodily sensations, and emotions.	☐	☐	☐	☐	☐	☐	☐
FF1_12. It's hard for me to find the words to describe what I'm thinking.	☐	☐	☐	☐	☐	☐	☐
FF1_13. I am easily distracted.	☐	☐	☐	☐	☐	☐	☐
FF1_14. I believe some of my thoughts are abnormal or bad and I shouldn't think that way.	☐	☐	☐	☐	☐	☐	☐
FF1_15. I pay attention to sensations, such as the wind in my hair or sun on my face.	☐	☐	☐	☐	☐	☐	☐

	Never	Almost Never	Sometimes	Fairly Often	Often	I do not know	I prefer not to answer
FF1_16. I have trouble thinking of the right words to express how I feel about things.	☐	☐	☐	☐	☐	☐	☐
FF1_17. I make judgments about whether my thoughts are good or bad.	☐	☐	☐	☐	☐	☐	☐
FF1_18. I find it difficult to stay focused on what's happening in the present.	☐	☐	☐	☐	☐	☐	☐
FF1_19. When I have distressing thoughts or images, I "step back" and am aware of the thought or image without getting taken over by it.	☐	☐	☐	☐	☐	☐	☐
FF1_20. I pay attention to sounds, such as clocks ticking, birds chirping, or cars passing.	☐	☐	☐	☐	☐	☐	☐
FF1_21. In difficult situations, I can pause without immediately reacting.	☐	☐	☐	☐	☐	☐	☐
FF1_22. When I have a sensation in my body, it's difficult for me to describe it because I can't find the right words.	☐	☐	☐	☐	☐	☐	☐
FF1_23. It seems I am "running on automatic" without much aware- ness of what I'm doing.	☐	☐	☐	☐	☐	☐	☐
FF1_24. When I have distressing thoughts or images, I feel calm soon after.	☐	☐	☐	☐	☐	☐	☐
FF1_25. I tell myself that I shouldn't be thinking the way I'm thinking.	☐	☐	☐	☐	☐	☐	☐
FF1_26. I notice the smells and aromas of things.	☐	☐	☐	☐	☐	☐	☐
FF1_27. Even when I'm feeling terribly upset, I can find a way to put it into words.	☐	☐	☐	☐	☐	☐	☐
FF1_28. I rush through activities without being really attentive to them.	☐	☐	☐	☐	☐	☐	☐

	Never	Almost Never	Sometimes	Fairly Often	Often	I do not know	I prefer not to answer
~~FF1_29. When I have distressing thoughts or images, I am able just to notice them without reacting.~~	☐	☐	☐	☐	☐	☐	☐
~~FF1_30. I think some of my emotions are bad or inappropriate and I shouldn't feel them.~~	☐	☐	☐	☐	☐	☐	☐
~~FF1_31. I notice visual elements in art or nature, such as colors, shapes, textures, or patterns of light and shadow.~~	☐	☐	☐	☐	☐	☐	☐
~~FF1_32. My natural tendency is to put my experiences into words.~~	☐	☐	☐	☐	☐	☐	☐
~~FF1_33. When I have distressing thoughts or images, I just notice them and let them go.~~	☐	☐	☐	☐	☐	☐	☐
~~FF1_34. I do jobs or tasks automatically without being aware of what I'm doing.~~	☐	☐	☐	☐	☐	☐	☐
~~FF1_35. When I have distressing thoughts or images, I judge myself as good or bad depending what the thought or image is about.~~	☐	☐	☐	☐	☐	☐	☐
~~FF1_36. I pay attention to how my emotions affect my thoughts and behavior.~~	☐	☐	☐	☐	☐	☐	☐
~~FF1_37. I can usually describe how I feel at the moment in consider- able detail.~~	☐	☐	☐	☐	☐	☐	☐
~~FF1_38. I find myself doing things without paying attention.~~	☐	☐	☐	☐	☐	☐	☐
~~FF1_39. I disapprove of myself when I have irrational ideas.~~	☐	☐	☐	☐	☐	☐	☐

Parent's Education

CS1_01. Please check the box beside the highest grade or degree that your BIOLOGICAL MOTHER completed.

Never went to school	☐
Grades 1 to 3	☐
Grades 4 to 8	☐
Grades 9 to 11	☐
Grade 12	☐
GED	☐
One or more years of Vocational or Professional School after High School	☐
One or more years of College	☐
One or more years of Graduate or Professional School after College	☐
I Do Not Know	☐
I prefer not to answer	☐

CS1_02. Please check the box beside the highest grade or degree that your BIOLOGICAL FATHER completed.

Never went to school	☐
Grades 1 to 3	☐
Grades 4 to 8	☐
Grades 9 to 11	☐
Grade 12	☐
GED	☐
One or more years of Vocational or Professional School after High School	☐
One or more years of College	☐
One or more years of Graduate or Professional School after College	☐
I Do Not Know	☐
I prefer not to answer	☐

Now please think of the two most important adults in your home between the time you were born and age 18 years. Please check the category below that best described their level of education during this time period.

CS1_03. First adult's highest level of education:

Never went to school	☐
Grades 1 to 3	☐
Grades 4 to 8	☐
Grades 9 to 11	☐
Grade 12	☐
GED	☐
One or more years of Vocational or Professional School after High School	☐
One or more years of College	☐
One or more years of Graduate or Professional School after College	☐
I Do Not Know	☐
I prefer not to answer	☐

CS1_04. Second adult's highest level of education

Never went to school	☐
Grades 1 to 3	☐
Grades 4 to 8	☐
Grades 9 to 11	☐
Grade 12	☐
GED	☐
One or more years of Vocational or Professional School after High School	☐
One or more years of College	☐
One or more years of Graduate or Professional School after College	☐
I Do Not Know	☐
I prefer not to answer	☐

Your Childhood Experiences

The following questions ask about some difficult experiences that you might have had as a child. These questions may be emotionally difficult to answer. Just as a reminder, you do not need answer any questions that you would prefer not to. Your answers to these questions, as with all questions, will remain confidential.

~~CE1_01. Before you were 18 years old, did a parent or other adult in the household often or very often…~~

Comment [D7]: Replaced with other childhood scales. See below.

~~Swear at you, insult you, put you down, or humiliate you?~~
~~or~~
~~Act in a way that made you afraid that you might be physically hurt?~~

~~□ No~~
~~□ Yes~~
~~□ I do not know~~
~~□ I prefer not to answer~~

~~CE1_02.Before you were 18 years old, did a parent or other adult in the household often or very often…~~

~~Push, grab, slap, or throw something at you?~~
~~or~~
~~Ever hit you so hard that you had marks or were injured?~~

~~□ No~~
~~□ Yes~~
~~□ I do not know~~
~~□ I prefer not to answer~~

~~CE1_03. Before you were 18 years old, did an adult or person at least 5 years older than you ever…~~

~~Touch or fondle you or have you touch their body in a sexual way?~~
~~or~~
~~Attempt or actually have oral, anal, or vaginal intercourse with you?~~
~~□ No~~
~~□ Yes~~
~~□ I do not know~~
~~□ I prefer not to answer~~

CE1_04. Before you were 18 years old, did you often or very often feel that …

No one in your family loved you or thought you were important or special?

or

Your family didn't look out for each other, feel close to each other, or support each other?

- ☐ No
- ☐ Yes
- ☐ I do not know
- ☐ I prefer not to answer

CE1_05. Before you were 18 years old, did you often or very often feel that …

You didn't have enough to eat, had to wear dirty clothes, and had no one to protect you?

or

Your parents were too drunk or high to take care of you or take you to the doctor if you needed it?

- ☐ No
- ☐ Yes
- ☐ I do not know
- ☐ I prefer not to answer

CE1_06. Before you were 18 years old, was a biological parent ever lost to you through divorce, abandonment, or other reason?

- ☐ No
- ☐ Yes
- ☐ I do not know
- ☐ I prefer not to answer

CE1_07. Before you were 18 years old, was your mother or stepmother:

Often or very often pushed, grabbed, slapped, or had something thrown at her?

or

Sometimes, often, or very often kicked, bitten, hit with a fist, or hit with something hard?

or

Ever repeatedly hit over at least a few minutes or threatened with a gun or knife?

- ☐ No
- ☐ Yes
- ☐ I do not know
- ☐ I prefer not to answer

~~CE1_08.Before you were 18 years old, did you live with anyone who was a problem drinker or alcoholic, or who used street drugs?~~
~~□ No~~
~~□ Yes~~
~~□ I do not know~~
~~□ I prefer not to answer~~

~~CE1_09.Before you were 18 years old, was a household member depressed or mentally ill, or did a household member attempt suicide?~~

~~□ No~~
~~□ Yes~~
~~□ I do not know~~
~~□ I prefer not to answer~~

~~CE1_10.Before you were 18 years old, did a household member go to prison?~~

~~□ No~~
~~□ Yes~~
~~□ I do not know~~
~~□ I prefer not to answer~~

Childhood Trauma Questionnaire (CTQ)

Comment [D8]: NEW

**When I was growing up:**	**Never True**	**Rarely True**	**Sometimes True**	**Often True**	**Very Often True**
1. I didn't have enough to eat.	1	2	3	4	5
2. I knew there was someone to take care of me and protect me.	1	2	3	4	5
3. People in my family called me things like "stupid", "lazy", or "ugly."	1	2	3	4	5
4. My parents were too drunk or high to take care of the family.	1	2	3	4	5
5. There was someone in my family who helped me feel important or special.	1	2	3	4	5
6. I had to wear dirty clothes.	1	2	3	4	5

7. I felt loved.	1	2	3	4	5
8. I thought that my parents wished I had never been born.	1	2	3	4	5
9. I got hit so hard by someone in my family that I had to see a doctor or go to the hospital.	1	2	3	4	5
10. There was nothing I wanted to change about my family.	1	2	3	4	5
11. People in my family hit me so hard that it left me with bruises or marks.	1	2	3	4	5
12. I was punished with a belt, a board, a cord, or some other hard object.	1	2	3	4	5
13. People in my family looked out for each other.	1	2	3	4	5
14. People in my family said hurtful or insulting things to me.	1	2	3	4	5
15. I believe that I was physically abused.	1	2	3	4	5
16. I had the perfect childhood.	1	2	3	4	5
17. I got hit or beaten so badly that it was noticed by someone like a teacher, neighbor, or doctor.	1	2	3	4	5
18. I felt that someone in my family hated me.	1	2	3	4	5
19. People in my family felt close to each other.	1	2	3	4	5
20. Someone tried to touch me in a sexual way, or tried to make me touch them.	1	2	3	4	5
21. Someone threatened to hurt me or tell lies about me unless I did something sexual with them.	1	2	3	4	5
22. I had the best family in the world.	1	2	3	4	5
23. Someone tried to make me do sexual things or watch sexual things.	1	2	3	4	5

24. Someone molested me.	1	2	3	4	5
25. I believe that I was emotionally abused.	1	2	3	4	5
26. There was someone to take me to the doctor if I needed it.	1	2	3	4	5
27. I believe that I was sexually abused.	1	2	3	4	5
28. My family was a source of strength and support.	1	2	3	4	5

Food Insecurity Items
National Comorbidity Survey Replication – Adolescent Supplement

Comment [D9]: NEW

Adapted by Eric Loucks for Adult-Administered Retrospective Reporting During Childhood

During childhood, were you ever hungry but did not eat because you could not afford to buy food?

1) Yes
2) No

During childhood, did you ever eat less than you felt you should because you or your family didn't have money to buy food?

1) Yes
2) No

How often during childhood did you not have enough money to buy food?

1) Never
2) Rarely
3) Sometimes
4) Often

How often during childhood could you not afford to eat balanced meals?

1) Never
2) Rarely
3) Sometimes
4) Often

AS YOU REMEMBER YOUR MOTHER FIGURE:
Please circle the appropriate number. If you had more than one mother figure, choose the one you were with longest, or the one you found most difficult to live with.

Comment [D10]: NEW

WHICH MOTHER FIGURE ARE YOU DESCRIBING BELOW?

1. Natural mother
2. Step-mother/ father's live-in partner
3. Other relative (e.g., aunt, grandmother)
4. Other non-relative (e.g., foster mother, godmother)
5. Other (describe) ______________________________

	NO<br>NOT AT ALL		UNSURE		YES<br>DEFINITELY
1. She was concerned about my worries ………	1	2	3	4	5
2. She was interested in how I did at school.	1	2	3	4	5
3. She tried to make me feel better when I was upset…………..	1	2	3	4	5
4. She was interested in who my friends were	1	2	3	4	5
5. She was concerned about where I was and what I was doing…	1	2	3	4	5
6. She cared for me when I was sick ………….	1	2	3	4	5

AS YOU REMEMBER YOUR FATHER FIGURE:
Please circle the appropriate number. If you had more than one father figure, choose the one you were with longest, or the one you found most difficult to live with. If you had no father in the household then leave out this section.

WHICH FATHER FIGURE ARE YOU DESCRIBING BELOW?

1. Natural father
2. Step-father/ mother's live-in partner
3. Other relative (e.g., uncle, grandfather)
4. Other non-relative (e.g., foster father, godfather)
5. Other (describe) ___________________________

	NO<br>NOT AT ALL		UNSURE		YES<br>DEFINITELY
1. He was concerned about my worries ………	1	2	3	4	5
2. He was interested in how I did at school.	1	2	3	4	5
3. He tried to make me feel better when I was upset…………..	1	2	3	4	5
4. He was interested in who my friends were	1	2	3	4	5
5. He was concerned about where I was and what I was doing…	1	2	3	4	5
6. He cared for me when I was sick …………	1	2	3	4	5

Comment [D11]: NEW

Connor-Davidson Resilience Scale 10 (CD-RISC-10)

*Please indicate how much you agree with the following statements as they apply to you over the last **month**. If a particular situation has not occurred recently, answer according to how you think you would have felt.*

		not true at all (0)	rarely true (1)	sometimes true (2)	often true (3)	true nearly all the time (4)
1.	I am able to adapt when changes occur.	☐	☐	☐	☐	☐
2.	I can deal with whatever comes my way.	☐	☐	☐	☐	☐
3.	I try to see the humorous side of things when I am faced with problems.	☐	☐	☐	☐	☐
4.	Having to cope with stress can make me stronger.	☐	☐	☐	☐	☐
5.	I tend to bounce back after illness, injury, or other hardships.	☐	☐	☐	☐	☐
6.	I believe I can achieve my goals, even if there are obstacles.	☐	☐	☐	☐	☐
7.	Under pressure, I stay focused and think clearly.	☐	☐	☐	☐	☐
8.	I am not easily discouraged by failure.	☐	☐	☐	☐	☐
9.	I think of myself as a strong person when dealing with life's challenges and difficulties.	☐	☐	☐	☐	☐
10.	I am able to handle unpleasant or painful feelings like sadness, fear, and anger.	☐	☐	☐	☐	☐

Chronic Illness

CD1_01. Do you have a chronic illness, health problem or disease?

□ Yes

□ No (if responding "no", please skip to the next section).

CD1_02. Please select the illness from the list below that has the greatest effect on your life or feels like the *most important* for you to be able to manage. You may have more than one, but the purpose of this question is to identify what you might view as your PRIMARY chronic illness/problem/disease.

- ☐ Diabetes
- ☐ Heart Disease
- ☐ Hypertension
- ☐ Obesity
- ☐ Metabolic Syndrome
- ☐ Arthritis
- ☐ Chronic Pain
- ☐ Asthma
- ☐ COPD
- ☐ Depression
- ☐ Anxiety
- ☐ Insomnia
- ☐ Substance use
- ☐ Tobacco use
- ☐ Alcohol overuse
- ☐ Prescription medication overuse
- ☐ Illicit drug use
- ☐ Other ______________________________

We would like to know how confident you are in doing certain activities related to the chronic illness or disease you selected above. For each of the following questions, please choose the number that corresponds to your confidence that you can do the tasks regularly at the present time. Please keep the chronic illness or disease in mind as you answer the following questions.

How confident are you that….	Not at All Confident 1	2	3	4	5	6	7	8	9	Totally Confident 10
CD1_03a …you can keep the fatigue caused by your disease from interfering with the things you want to do?	□	□	□	□	□	□	□	□	□	□
CD1_03b …you can keep the physical discomfort or pain of your disease from interfering with the things you want to do?	□	□	□	□	□	□	□	□	□	□
CD1_03c …you can keep the emotional distress caused by your disease from interfering with the things you want to do?	□	□	□	□	□	□	□	□	□	□
CD1_03d …you can keep any other symptoms or health problems you have from interfering with the things you want to do?	□	□	□	□	□	□	□	□	□	□
CD1_03e …you can do the different tasks and activities needed to manage your health condition so as to reduce you need to see a doctor?	□	□	□	□	□	□	□	□	□	□
CD1_03f …you can do things other than just taking medication to reduce how much your illness affects your everyday life?	□	□	□	□	□	□	□	□	□	□

Blood Pressure and Blood Pressure Medication Use

The following questions are about blood pressure.

BP1_1. Does your blood pressure tend to be HIGHER when you have it measured in a clinical setting, such as a doctor's office? This is sometimes called "white coat hypertension."

- □ No
- □ Yes → **SKIP TO BM1_01**
- □ I do not know
- □ I prefer not to answer

BP1_2. Does your blood pressure tend to be LOWER when you have it measured in a clinical setting, such as a doctor's office? This is sometimes called "masked hypertension."

- □ No
- □ Yes
- □ I do not know
- □ I prefer not to answer

~~BM1_01. Do you currently take medication for your blood pressure?~~
~~□ Yes~~
~~□ No (if responding "no", please skip to the next page).~~

	~~Yes~~	~~No~~
~~BM1_02. Do you sometimes forget to take your blood pressure pills?~~	~~□~~	~~□~~
~~BM1_03. People sometimes miss taking their medications for reasons other than forgetting. Thinking over the past two weeks, were there any days when you did not take your blood pressure medicine?~~	~~□~~	~~□~~
~~BM1_04. Have you ever cut back or stopped taking your blood pressure medicine without telling your doctor because you felt worse when you took it?~~	~~□~~	~~□~~
~~BM1_05. When you travel or leave home, do you sometimes forget to bring along your blood pressure medicine?~~	~~□~~	~~□~~
~~BM1_06. Did you take all your blood pressure medicine yesterday?~~	~~□~~	~~□~~
~~BM1_07. When you feel like your symptoms are under control, do you sometimes stop taking your blood pressure medicine?~~	~~□~~	~~□~~
~~BM1_08. Taking blood pressure medicine every day is a real inconvenience for some people. Do you ever feel hassled about sticking to your treatment plan?~~	~~□~~	~~□~~

~~BM1_09. How often do you have difficulty remembering to take all your blood pressure medicine?~~
~~□ Never/rarely~~
~~□ Once in a while~~
~~□ Sometimes~~
~~□ Usually~~
~~□ All the time~~

Comment [D12]: REMOVED

More About You

Below are some modifiable factors that likely influence blood pressure. These may not all apply to you, as you may already have excellent levels of these factors.

Physical activity:

The United States Office of Disease Prevention and Health Promotion 2008 Physical Activity Guidelines for Americans states that "Most health benefits occur with at least 150 minutes (2 hours and 30 minutes) a week of moderate intensity physical activity, such as brisk walking. Additional benefits occur with more physical activity. Both aerobic (endurance) and muscle-strengthening (resistance) physical activity are beneficial."

RC1_01. How motivated are you to make changes to your physical activity, using a scale of one to ten, where one = definitely not ready to change, and 10 = definitely ready to change?

Little intention of changing			Mixed feelings towards taking action				Motivated to take action		
1	2	3	4	5	6	7	8	9	10
☐	☐	☐	☐	☐	☐	☐	☐	☐	☐

RC1_02. On a scale of 1-10, with 10 being 100% confident, how confident are you that you can make changes in your physical activity?

Not confident			Moderately confident				Confident		
1	2	3	4	5	6	7	8	9	10
☐	☐	☐	☐	☐	☐	☐	☐	☐	☐

Diet:

The Dietary Approaches to Stop Hypertension (DASH) diet eating plan is a diet rich in fruits, vegetables, low fat or nonfat dairy. It also includes mostly whole grains; lean meats, fish and poultry; nuts and beans. It is high fiber and low to moderate in fat. It is a plan that follows US guidelines for sodium content, along with vitamins and minerals. It can be considered to be an Americanized version of the Mediterranean diet.

RC1_03. How motivated are you to make changes to your diet to be consistent with the DASH diet, using a scale of one to ten, where one = definitely not ready to change, and 10 = definitely ready to change?

Little intention of changing			Mixed feelings towards taking action				Motivated to take action		
1	2	3	4	5	6	7	8	9	10
☐	☐	☐	☐	☐	☐	☐	☐	☐	☐

RC1_04. On a scale of 1-10, with 10 being 100% confident, how confident are you that you can make changes in your diet to be more consistent with the DASH diet?

Not confident			Moderately confident				Confident		
1	2	3	4	5	6	7	8	9	10
☐	☐	☐	☐	☐	☐	☐	☐	☐	☐

Salt Intake:

The 2010 Dietary Guidelines for Americans recommend that everyone age 2 years and up should consume less than 2,300 milligrams (mg) of sodium each day. Some groups of people should further limit sodium intake to 1,500 mg per day, including:

- Adults age 51 years or older.
- All African Americans.
- Anyone who has high blood pressure, diabetes, or chronic kidney disease.

RC1_05. How motivated are you to make changes to your salt intake, using a scale of one to ten, where one = definitely not ready to change, and 10 = definitely ready to change?

Little intention of changing			Mixed feelings towards taking action				Motivated to take action		
1	2	3	4	5	6	7	8	9	10
☐	☐	☐	☐	☐	☐	☐	☐	☐	☐

RC1_06. On a scale of 1-10, with 10 being 100% confident, how confident are you that you can make changes in your salt intake?

Not confident			Moderately confident				Confident		
1	2	3	4	5	6	7	8	9	10
☐	☐	☐	☐	☐	☐	☐	☐	☐	☐

Overweight/Obesity:

Extensive scientific evidence shows that being overweight or obese increases risk of having high blood pressure.

RC1_07. How motivated are you to make changes to your body weight, using a scale of one to ten, where one = definitely not ready to change, and 10 = definitely ready to change?

Little intention of changing			Mixed feelings towards taking action				Motivated to take action		
1	2	3	4	5	6	7	8	9	10
☐	☐	☐	☐	☐	☐	☐	☐	☐	☐

RC1_08. On a scale of 1-10, with 10 being 100% confident, how confident are you that you can make changes in your body weight?

Not confident			Moderately confident				Confident		
1	2	3	4	5	6	7	8	9	10
☐	☐	☐	☐	☐	☐	☐	☐	☐	☐

Stress, and Stress Response:

Several studies showed that stress, and being slower at emotionally recovering from stressful events, increase risk of hypertension.

RC1_09. How motivated are you to make changes to the amount of stress in your life, or your response to that stress, using a scale of one to ten, where one = definitely not ready to change, and 10 = definitely ready to change?

Little intention of changing			Mixed feelings towards taking action				Motivated to take action		
1	2	3	4	5	6	7	8	9	10
☐	☐	☐	☐	☐	☐	☐	☐	☐	☐

RC1_10. On a scale of 1-10, with 10 being 100% confident, how confident are you that you can make changes in the amount of stress in your life, or your response to that stress?

Not confident			Moderately confident				Confident		
1	2	3	4	5	6	7	8	9	10
☐	☐	☐	☐	☐	☐	☐	☐	☐	☐

Alcohol Consumption:

Heavy and regular use of alcohol can increase blood pressure substantially. The American Heart Association recommends limiting alcohol consumption to no more than two drinks per day for men and one drink per day for women.

RC1_11. How motivated are you to make changes to the amount of alcohol you consume, using a scale of one to ten, where one = definitely not ready to change, and 10 = definitely ready to change?

Little intention of changing			Mixed feelings towards taking action				Motivated to take action		
1	2	3	4	5	6	7	8	9	10
☐	☐	☐	☐	☐	☐	☐	☐	☐	☐

RC1_12. On a scale of 1-10, with 10 being 100% confident, how confident are you that you can make changes in the amount of alcohol you consume?

Not confident			Moderately confident				Confident		
1	2	3	4	5	6	7	8	9	10
☐	☐	☐	☐	☐	☐	☐	☐	☐	☐

Blood Pressure (Antihypertensive) Medication Use:

Blood pressure medication has been shown in many studies to be very effective at lowering blood pressure.

RC1_13. How motivated are you to make changes to your blood pressure medication use, using a scale of one to ten, where one = definitely not ready to change, and 10 = definitely ready to change?

Little intention of changing			Mixed feelings towards taking action				Motivated to take action		
1	2	3	4	5	6	7	8	9	10
☐	☐	☐	☐	☐	☐	☐	☐	☐	☐

RC1_14. On a scale of 1-10, with 10 being 100% confident, how confident are you that you can make changes your blood pressure medication use?

Not confident			Moderately confident				Confident		
1	2	3	4	5	6	7	8	9	10
☐	☐	☐	☐	☐	☐	☐	☐	☐	☐

Family History of Hypertension

FH1_01. Did your biological mother ever have hypertension?

- □ No
- □ Yes
- □ I do not know
- □ I prefer not to answer

FH1_02. Did your biological father ever have hypertension?

- □ No
- □ Yes
- □ I do not know
- □ I prefer not to answer

FH1_03. How many full brothers and sisters do you have ***(Please include any brothers or sisters who may have died, but do not include half or step brothers and sisters).***

- □ I do not have any brothers or sisters → S***kip to the page***
- ____ brothers, ______ sisters
- □ I do not know
- □ I prefer not to answer

FH1_04. Of these brothers and sisters, how many have ever had hypertension?

- ________ ***(if none, write 0)***
- □ I do not know
- □ I prefer not to answer

Your Sleep

Comment [D13]: MOVED to in-person baseline assessment

The following question relates to your usual sleep habits during the past month only. Your answer should indicate the most accurate reply for the majority of days and nights in the past month.

SL1_04. **During the past month**, how many hours of actual sleep did you get on average at night? (This may be different than the number of hours you spent in bed.)

AVERAGE HOURS OF SL1_EEP PER NIGHT ____________

☐ I do not know
☐ I prefer not to answer

END SCRIPT

Thank you for completing this survey!

Please note that these responses will not be seen immediately. Resources are shown below if you feel that you would like to talk with someone immediately for assistance.

National Suicide Prevention Lifeline: 1-800-273-8255
National Sexual Assault Hotline: 1-800-656-4673

Other options are to:

- Call your doctor's office
- Call 911 for emergency services
- Go to the nearest hospital emergency room.

Appendix 6

MB-BP Study Safety Plan (v.2.0) –

with track changes

Brown University Mindfulness & Cardiovascular Health Lab
SAFETY PLAN

Blood Pressure Safety Guidelines:

Warning - Participant's Blood Pressure is out-of-range of the predetermined 'safe' range (i.e., systolic blood pressure > 200mmHg or < 90mmHg OR diastolic blood pressure > 110mmHg). See below for the study safety protocol.

If SBP>200 and/or DBP>110 and NO symptoms (e.g., chest pain, shortness of breath, dizziness, headache), strongly encourage the participant to see his/her doctor right away or to go to urgent care.

If SBP<90 and NO symptoms (e.g., chest pain, shortness of breath, dizziness, passing out), strongly encourage the participant to see his/her doctor right away or to go to urgent care.

*****If there are symptoms, call 911 immediately.*****

Uncontrolled Hypertension *– not an immediate safety threat, but BP is high enough that follow up is needed. See below for details.*

If a participant's average systolic blood pressure is 140 mmHg or greater or if his/her average diastolic blood pressure is 90 mmHg or greater then he or she has uncontrolled hypertension.

Notify the participant of his/her blood pressure readings and work with the participant to determine whether or not he/she has a primary care physician or other health care provider who is aware of his/her uncontrolled hypertension.

- If physician is aware and participant is under treatment, then no action is required.
- If participant's physician is not aware of the uncontrolled hypertension, then collect health care provider info from participant and request permission to notify his/her physician of the reading.
- If participant does not have a health care provider and/or does not have health insurance, then offer participant assistance with creating an action plan including assisting participant with finding a health care provider using the RI Department of Health provider search engine: http://www.health.state.ri.us/find/primarycare/.

Document the situation and outcome and notify the study coordinator.

Irregular ECG Reading *– as indicated by the Kardia Mobile* device during the "Heart Beat Detection Task". Follow the protocol below.*

If during the Heart Beat Detection task, the Kardia Mobile device indicates that there was a '*possible Atrial Fibrillation*' present…

- Notify the participant of the result of the reading; make sure to emphasize that the ECG result presents only *potential* findings and that further follow up is recommended.
- If SYMPTOMS present, have ppt call his/her physician immediately or offer to call 911; ECG readings can even be sent to physician via email for review if participant prefers.
- If NO symptoms, save the ECG results and immediately send them to Dr. Hank Wu (wen-chih_wu@brown.edu) for further review. Dr. Wu is a board-certified cardiologist serving on the study Data Safety Monitoring Board (DSMB). Review will take place within 36 hours.
- Follow up with the participant with Dr. Wu's recommendations / analysis.

* The Kardia Mobile device is designed to record, store, and transfer single-channel electrocardiogram (ECG) rhythms.

Anxiety and Depression Symptomology:

After the participant has completed the Beck Anxiety Inventory questionnaire (BAI) and the Centers for Epidemiologic Studies Depressive Symptomatology (CESD-R) questionnaire, ask the participant to wait for a few minutes while you check that all forms and assessments are completed. While the participant is waiting ~~in a different room~~, check the scores according to the criteria below. If any scores trigger the safety plan, move forward with steps below as written.

Commented [SF1]: No longer necessary since the check can occur discretely in the same room. The RA can step out of the room to talk with the PI, clinician, and/or study coordinator if necessary.

Beck Anxiety Inventory (BA)

If participant scores ≥26 on the Beck Anxiety Inventory, while having the participant wait, immediately notify the collaborating psychiatrist, Dr. Ellen Flynn (cell phone # 401-258-9829), to determine need for a psychiatric consultation. She has provided her cell phone #, and your phone # will be entered into her phone recognition software to indicate it is a call from the ~~MBHT~~ Mindfulness & CV Health Lab Staff. It is likely she will answer the phone. If Dr. Flynn cannot be reached, please leave a phone message for her with your contact information and provide her with the Beck Anxiety Inventory results on the message. If Dr. Flynn is not immediately reachable, or following participant release after consulting with Dr. Flynn, immediately contact the PI, Dr. Eric Loucks (cell phone #401-369-0443).

Depressive Symptomatology (DS)

The CESD-R will be administered during the in-person assessment visits, and scores will be reviewed immediately upon completion of the in-person assessments.

1. Sadness (dysphoria): Question numbers 2,4, 6
2. Loss of Interest (anhedonia): Question numbers 8, 10
3. Appetite: Question numbers 1, 18
4. Sleep: Question numbers 5, 11, 19
5. Thinking / concentration: Question numbers 3, 20
6. Guilt (worthlessness): Question numbers 9, 17
7. Tired (fatigue): Question numbers 7, 16
8. Movement (agitation): Question numbers 12, 13
9. Suicidal ideation: Question numbers 14, 15

Participants are considered to meet criteria for major depressive episode if they have anhedonia or dysphoria nearly every day for the past two weeks, plus symptoms in an additional 4 DSM symptom groups noted as occurring nearly every day for the past two weeks. If participants meet criteria for major depressive episode, while having the participant wait, immediately notify the collaborating psychiatrist, Dr. Ellen Flynn (cell phone # 401-258-9829), to determine need for a psychiatric consultation. She has provided her cell phone #, and your phone # will be entered into her phone recognition software to indicate it is a call from the Mindfulness & CV Health Lab Staff.~~MBHT study.~~ It is likely she will answer the phone. If Dr. Flynn cannot be reached, please leave a phone message for her with your contact information and provide her with the Depressive Symptomatology results on the message. If Dr. Flynn is not immediately reachable, or following participant release after consulting with Dr. Flynn, immediately contact the PI, Dr. Eric Loucks (cell phone #401-369-0443).

For both situations described above, make sure to check in with the study participant to let them know that they screened high for depressive symptomology and/or anxiety and that we want to make sure they have the resources/support that they feel they need. Ask the participants if they have someone to talk to and if they would like to speak with the study clinician, who would be happy to give them a follow up call.

Commented [SF2]: The study clinician is available to talk to participants who are flagged for DEP / ANX. She can assist with directing them to resources if needed. Note that she provides data safety monitoring and consultation, but is not directly involved in the data collection nor the study intervention.

Suicidal Ideation:

Commented [SF3]: Revised per feedback / discussion with study clinician. Items 14 and 15 on the CESD-R capture suicidal ideation on a spectrum and does not always equate to immediate intent to harm oneself.

If participants respond having any suicidal ideation (DS questions 14 or 15), perform the following 2 steps:

1. **Check in with the participant and let them know we are concerned for their well-being. Ask if they are in danger of harming themselves in the next 24 hours.**
2. **If they are, immediately call 911 and Dr. Ellen Flynn.**

Specifically, while the participant is in the ~~waiting~~ assessment room, *call 911 immediately, and tell them:*

Commented [SF4]: More private setting where the participant is already located at this point in the study assessment.

"My name is _____. I am working on a research study at Brown University. I have a study participant in ~~the waiting room~~ our office who has shared that he/she is

currently suicidal." Please provide the participants' name to the 911 operator, as requested.

Finally, after calling 911, please call Dr. Ellen Flynn, who is the psychiatrist supporting this study. Her cell phone # is 401-258-9829. Please provide her with the same information as was done during the 911 call.

While speaking calmly with the participant, let them know that you have called 911 and Dr. Flynn, and why (i.e. because we are concerned about you). You can speak with participant to keep him/her in the ~~waiting~~ assessment room if 911 has sent assistance, but the discussion should not be clinical in nature.

Examples of questions that could be asked in order to keep them in the ~~waiting~~ assessment room:

- "Tell me what is going on."
- "What's happening right now?"
- Tell me more about why you are interesting in being part of this study.
- What are you hoping to get out of this study?

The following information can be provided to study participants.

National Suicide Prevention Lifeline: 1-800-273-8255

Other options are to:

- Call your doctor's office
- Call 911 for emergency services
- Go to the nearest hospital emergency room.

Local Non-Urgent Free or Inexpensive Mental Health Services:
Gateway Healthcare: 401-729-8701
Anchor Counseling Center: 401-475-9979
The Providence Center: 401-276-4020

---

3. If the participant is NOT immediately suicidal, still let the participant know that we are concerned for his/her safety and that we would like to follow up with our study clinician. While the participant is still in the assessment room, immediately notify the collaborating psychiatrist, Dr. Ellen Flynn (cell phone # 401-258-9829), to determine need for a psychiatric consultation. If Dr. Flynn is not immediately reachable, or following participant release after consulting with Dr. Flynn, immediately contact the PI, Dr. Eric Loucks (cell phone #401-369-0443).

Appendix 7

Letter to health care provider if a participant screens for uncontrolled hypertension (>140/90 mmHg) AND has indicated that his/her provider is unaware of high BP levels.

BROWN
School of Public Health

Mindfulness & Cardiovascular Health Lab

Center for Population Health
& Clinical Epidemiology

Date

Dear [Provider Name]:

We are writing to inform you that one of your patient's, [PPT NAME], screened for having blood pressure above 140/90 mmHg (see blood pressure readings below) during an in-person research assessment in our office.

We inform the health care provider overseeing the care of any of our study participants, who have blood pressure above 140/90 mmHg, so that their physicians are aware.

The Mindfulness-Based Blood Pressure Reduction (MB-BP) Study is a five year National Institutes of Health grant that is evaluating the effects of a mindfulness-based intervention customized to blood pressure reduction. Effects of the intervention have been promising particularly in patients with uncontrolled hypertension, as shown in Figure 1. Included in this letter is our study advertisement card. Feel free to call or email if you have any further questions, or would be interested in referring patients to this study.

Sincerely,

Eric B. Loucks, PhD

Figure 1.

Associations of Mindfulness-Based Blood Pressure Reduction (MB-BP) intervention with systolic blood pressure at baseline (prior to MB-BP) and 3 months follow-up. Error bars represent SEM; n=43.

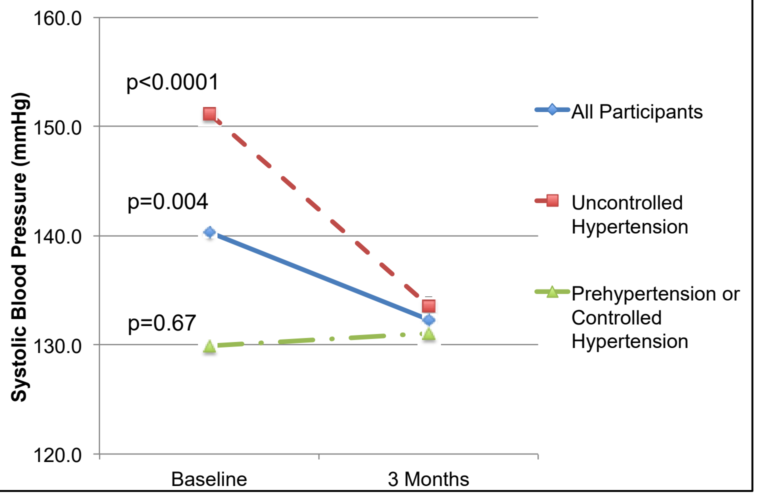

Appendix 8

Recruitment Materials
with website noted

MINDFULNESS-BASED INTERVENTION FOR BLOOD PRESSURE REDUCTION

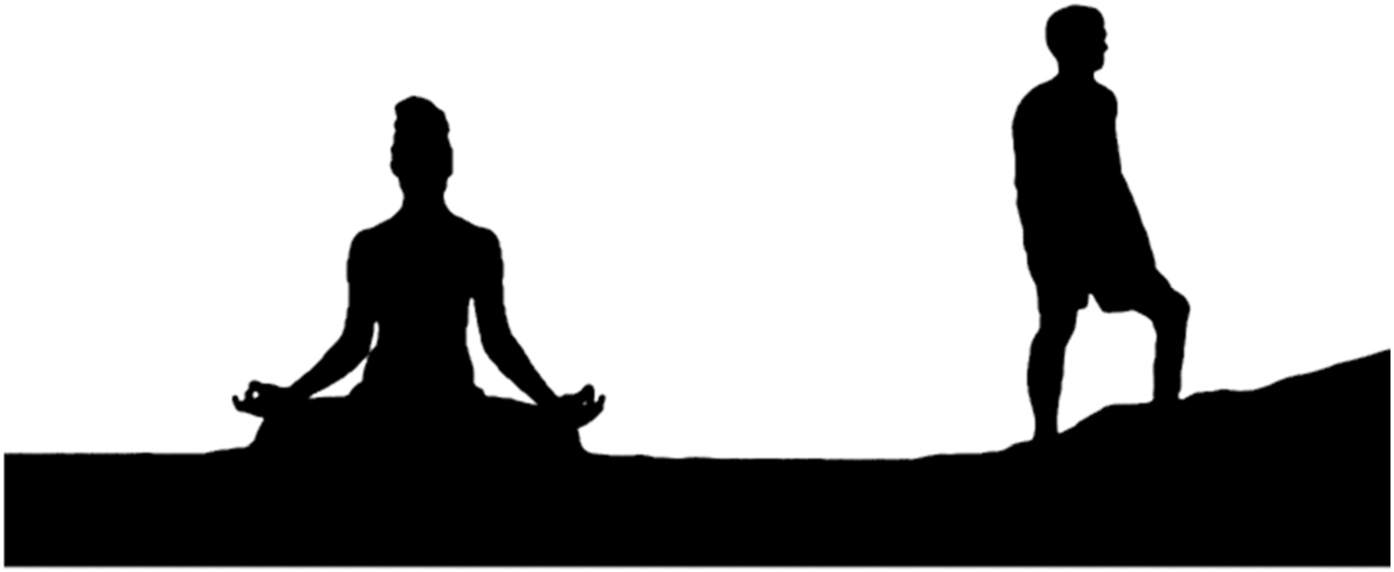

Free to qualified participants

This is a 9-week program that includes free training in mindfulness meditation, mindful movements, education about hypertension, and support for reducing hypertension risk factors. The research study is testing whether this intervention lowers blood pressure. The course consists of nine weekly sessions that are 2.5 hours, as well as an all-day weekend retreat. People who have a current regular meditation practice (i.e. meditate more than once per week) are not eligible.

Research participation includes interviews, questionnaires and health measurements, such as blood pressure, height, and weight, completed both before and after the meditation program.

Contact: For more information on our research study, or to see if you qualify, contact the Brown University Mindfulness & Cardiovascular Health Lab at 401-400-4768 (call or text), mindfulness@brown.edu (email) or visit us online at www.mindfulhearthealth.org.

Brown University
School of Public Health

BROWN

Mindfulness Blood Pressure Study
Call/Text: 401-400-4768
Email: mindfulness@brown.edu

Mindfulness Blood Pressure Study
Call/Text: 401-400-4768
Email: mindfulness@brown.edu

Mindfulness Blood Pressure Study
Call/Text: 401-400-4768
Email: mindfulness@brown.edu

Mindfulness Blood Pressure Study
Call/Text: 401-400-4768
Email: mindfulness@brown.edu

Mindfulness Blood Pressure Study
Call/Text: 401-400-4768
Email: mindfulness@brown.edu

Mindfulness Blood Pressure Study
Call/Text: 401-400-4768
Email: mindfulness@brown.edu

Mindfulness Blood Pressure Study
Call/Text: 401-400-4768
Email: mindfulness@brown.edu

Mindfulness Blood Pressure Study
Call/Text: 401-400-4768
Email: mindfulness@brown.edu

Mindfulness Blood Pressure Study
Call/Text: 401-400-4768
Email: mindfulness@brown.edu

Mindfulness Blood Pressure Study
Call/Text: 401-400-4768
Email: mindfulness@brown.edu

Mindfulness Blood Pressure Study
Call/Text: 401-400-4768
Email: mindfulness@brown.edu

Mindfulness Blood Pressure Study
Call/Text: 401-400-4768
Email: mindfulness@brown.edu

MINDFULNESS-BASED INTERVENTION
FOR BLOOD PRESSURE REDUCTION

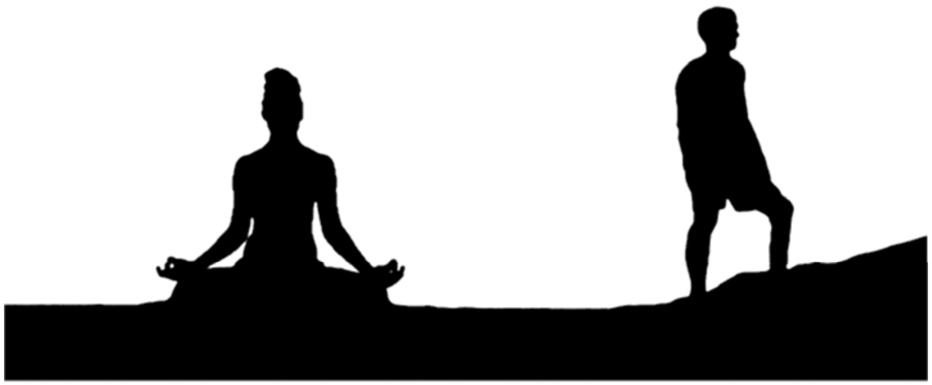

Free to qualified participants

For more information on our research study, or to see if you qualify, contact the Brown University Mindfulness & Cardiovascular Health Lab at 401-400-4768 (call or text), mindfulness@brown.edu (email) or visit us online at www.mindfulhearthealth.org.

Brown University
School of Public Health
BROWN

This is a 9-week program that includes free training in mindfulness meditation, mindful movements, education about hypertension, and support for reducing hypertension risk factors. The study is testing whether this intervention lowers blood pressure. The course consists of nine weekly sessions that are 2.5 hours, as well as an all-day weekend retreat. People who have a current regular meditation practice (i.e. meditate more than once per week) are not eligible.

Research participation includes interviews, questionnaires and health measurements, such as blood pressure, height, and weight, completed both before and after the meditation program.

Contact: For more information, or to see if you qualify, contact the Brown University Mindfulness & Cardiovascular Health Lab at 401-400-4768 (call or text), mindfulness@brown.edu (email) or visit us online at www.mindfulhearthealth.org.

Hi Ita,

Below are our responses; revised documents are attached. Thank you again for your time and attention on this!

Best,
Fran
____

Hi Eric,

Your new amendment submission received a pre-review and looks really good. However, I have the following questions/requests to clear up any confusion before IRB review:

Questions/Requests:

1. The study is currently approved to enroll 260 participants. Do you need/want to increase the study's N for the Pilot Trial?

   NOT AT THIS TIME.

2. In the consent document's "Nature and Purpose of the Study" section (pg. 1, 3rd paragraph, 2nd sentence), please remove "Using a covariate adaptive randomization process," as it is not in lay language and will not help participant understanding of the randomization process.

   ALL SET. WE HAVE REMOVED THE PHRASE MENTIONED ABOVE.

3. In the consent document's "Benefits" section (pg. 6), please re-word the last sentence as it is unclear. Also, if I understand the intent of the sentence, what guarantees that the monitor will work forever?

   WE HAVE REVISED THE SENTENCE TO READ: *"As part of the study, you will receive a wireless blood pressure monitor that will be yours to keep. This monitor may provide additional opportunity to monitor your blood pressure at home, which may benefit your health by providing additional biofeedback."*

4. In the *Letter to health care provider* (1st paragraph), please revise the first sentence to read: "We are writing to inform you that one of your *patients*, [PPT NAME] *is in a Brown University research study and* screened for ...." (italics show the change)

   DONE. SEE ATTACHED.

5. In the *Letter to health care provider* (2nd paragraph), please begin the 1st sentence with "*With the participant's permission*, we ...." (italics show the change)

Brown University

Research Protections Office
Institutional Review Board
Modification Request

Date of Request: 8/28/17 **Investigator's Name and Title:** Eric Loucks, PhD, Assistant Professor

Study Title: Mindfulness-based Blood Pressure Reduction (MB-BP) Study (#1412001171)

Original Type of Review: ☐ Exempt ☐ Expedited ☒ Full Board

1) **Provide a brief lay summary of the overall project. Include enough detail to allow the IRB to evaluate the requested change(s) within the context of the overall project.** (Attach summary to this form)

   See attached summary

2) **Provide a detailed description of the changes being requested** (Use additional pages, if necessary):

   See attached summary

3.) **State the reason (justification) for the requested modification.** (Use additional pages, if necessary):

   See attached summary

4.) **What is your assessment of how the changes will affect the overall risk/benefit ratio of the study and the willingness of individuals to participate?**

   See attached summary

5.) **Does the requested modification require new documents or changes to the approved consent form or other documents?**

- ☐ Consent/assent documents (attach revised version with changes highlighted)
- ☐ New/revised instruments (attach - if revised, highlight changes)
- ☒ New/revised advertising materials (attach - if revised, highlight changes)

Do you have a conflict of interest on this project according to Brown's policy? ☐**YES** ☒**NO**

If YES, has this conflict been previously disclosed to the IRB? ☐**YES** ☐**NO**

PI signature: 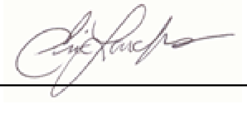 ______________________________ Date: _____8/28/2017________

1. **Provide a brief lay summary of the overall project. Include enough detail to allow the IRB to evaluate the requested change(s) within the context of the overall project.**

<u>*Original lay summary providing broad overview of the project*</u>*:*

The World Health Organization reported that suboptimal blood pressure (BP) is responsible for more than half of cardiovascular disease mortality world-wide. Furthermore, greater than half of those with hypertension have uncontrolled BP. <u>*A 2009 Institute of Medicine report recommended prioritizing research to "Compare the effectiveness of mindfulness-based interventions (e.g. yoga, meditation, deep breathing training) and usual care in treating… cardiovascular risk factors."*</u> Evidence-based mindfulness interventions, including Mindfulness-Based Stress Reduction, may have some effects on blood pressure, where a recent meta-analysis and systematic review of 4 randomized controlled trials demonstrated significant effects, but evidence of heterogeneity in effect sizes. The methodologically highest quality studies had the smallest effect sizes (range 0-5 mmHg). Mindfulness-Based Stress Reduction (MBSR) has been customized to a number of disease processes, such as Mindfulness-Based Cognitive Therapy for patients with recurrent depression, and Mindfulness-Based Relapse Prevention for patients with substance use addictions. Effect sizes have been increased by customizing mindfulness interventions to diseases of interest. The same may be true for hypertension, <u>*however mindfulness interventions customized for prehypertensive/hypertensive patients have never been investigated.*</u> <u>*Until methodologically rigorous studies to evaluate customized interventions for hypertension are performed, we will not know if the observed preliminary effects of general mindfulness interventions on blood pressure reduction could be much more effective with a tailored approach.*</u> Consequently, we propose to conduct a stage I behavioral therapy intervention study to evaluate whether MBSR customized to prehypertensive and hypertensive patients has the potential to provide clinically relevant reductions in BP. Consequently the specific aims are:

Stage 1a: Therapy Development/Manual Writing

1. To outline and evaluate key novel elements of mindfulness-based hypertension therapy (MBHT), customized from the evidence-based MBSR. *We hypothesize that the most important novel element will be generation of mindfulness skills specifically applied to hypertension risk factors such as diet, physical activity, obesity, alcohol consumption and antihypertensive medication adherence.* This aim will be achieved using (1) focus groups of participants undergoing the MBHT behavioral intervention, (2) discussion with experts (including cardiologists, epidemiologists, mindfulness experts, mindfulness intervention instructors) prior to, and following pilot testing of MBHT in participants, and (3) clinical judgment of the investigators performing the intervention.
2. To determine effectiveness of MBHT on primary outcomes (systolic blood pressure, retention rates, recruitment rates, and adverse effects) and secondary outcomes (hypertension risk factors such as diet, physical activity, obesity, and antihypertensive medication adherence) in hypertension subgroups, specifically participants with (1) prehypertension, (2) controlled hypertension, and (3) uncontrolled hypertension. Initial decisions about the targeted sample based on hypertension status will be made.
3. To develop an MBHT therapist manual and training program, including procedures for training, supervising, and evaluating therapists. Furthermore, acceptable therapist characteristics will be developed. The manual and training program will include themes such as specification of unique and common elements of MBHT *vs.* other interventions, description of interventions excluded from MBHT, and specification of key treatment

parameters such as frequency and duration of treatment, session length, topics addressed, sequence of sessions, as well as therapist adherence and competency measures. The MBHT training will consist of a therapist manual, a formal didactic training seminar, and at least one closely supervised training session.

Stage Ib: Pilot Trial

4. To determine whether a mindfulness-based hypertension therapy (MBHT) intervention, customized from the evidence-based MBSR, has promise to be an effective behavioral therapy for participants with hypertension and/or prehypertension. We will perform a randomized controlled pilot trial for MBHT *vs.* enhanced usual care control. *We hypothesize that MBHT will have adequate recruitment rates (≥10% of prehypertensive/hypertensive participants invited from physicians' offices), fairly low drop out rates (<15%), and medium effect sizes (e.g. 5-10 mmHg systolic BP) for reduction in blood pressure.*

These findings will provide publishable pilot data that will inform future randomized clinical trials that evaluate effects of MBHT on long-term changes in blood pressure *vs.* usual care and active control groups. <u>*If proven effective, MBHT could be offered as a complementary program in the prehypertensive/hypertensive patient population that contributes to over half of the cardiovascular disease mortality world-wide.*</u>

<u>Project update as of August 2017</u>:

The Mindfulness-Based Blood Pressure Reduction (MB-BP) Study, formerly known as Mindfulness-Based Hypertension Therapy (MBHT), is currently in Year 2 of a five year NIH UH2 grant. At this time we are in between phase 1 and phase 2 of the study.

We are in the final data collection phase of Stage 1a of the project, which involved a prospective single arm trial during the intervention development phase. All participants under this phase have been enrolled and follow up assessments are being conducted. In total we ran three separate Mindfulness-Based Blood Pressure reduction (MB-BP) intervention courses, with a total sample size of 43 eligible participants who both enrolled and completed the 9-week intervention. We had eight eligible participants enroll in the study who later decided to not complete the course. Assessments will continue through the 12 month follow up period with an estimated stage 1 data collection completion date of October 18, 2017.

In June we began enrollment for the next phase (referenced as Stage 1b above, but now called 'Stage 2a') of the study, which is to conduct a Randomized Controlled Trial (RCT) with *enhanced usual care*. This phase will continue for the next 12-18 months, at which point we will transition to the third and final phase, an RCT with an *active* control (i.e., Mindfulness-Based Stress Reduction - MBSR) as well as ambulatory blood pressure monitoring.

2. Provide a detailed description of the changes being requested (Use additional pages, if necessary):

This amendment covers the following requests:

1) **NEW Infographic Card** to be provided to individuals during recruitment efforts. We are requesting permission to use the attached infographic card (to be printed on glossy paper front and back) as part of our recruitment strategy. The infographic card provides simple visual language and information on mindfulness and how it may be connected to improved behavioral outcomes (i.e., lowered blood pressure).

2) **NEW Recruitment Card** – in addition to the already approved MB-BP recruitment material, we are requesting permission to use the attached alternative MB-BP recruitment card. The language about the study is pulled directly from the other material, but is organized slightly different, with section headings. The front graphic and layout is also slightly different.

3) **Permission to conduct direct mailing(s) at partnering provider office –** we are currently partnering with The Brown University Center for Primary Care and Prevention (CPCP) at Memorial Hospital of RI. Under the leadership of Dr. Charles "Chuck" Eaton, we have permission to conduct a direct mailing to a targeted segment of their patient population. Specifically, they have agreed to send out a recruitment mailing to patients within their practice, who meet certain study eligibility requirements (i.e., hypertensive with no history of mental illness). The mailing would include the following materials: (a) Attached cover letter co-signed by the patient's primary care provider and the MB-BP Study Principal Investigator; (b) revised MB-BP study recruitment card; and (c) attached mindfulness study infographic.

   In order to protect patient confidentiality, the direct mailing will be conducted at the CPCP office under the direct 'oversight' of CPCP staff. MB-BP staff will not remove any patient information from the CPCP office and will only have access to the minimal data needed to conduct the direct mailing (i.e., name and address ONLY). All other filtering will be done by CPCP staff and providers. Additionally, CPCP providers will be given the opportunity to use their own judgement to filter out any additional patients they determine to be 'unsuited' for the mailing.

   Note that the Brown University Center for Primary Care and Prevention, Memorial Hospital, and CPCP providers will not be directly involved in the research study nor the collection of data.

3. State the reason (justification) for the requested amendment:

Our preliminary analyses suggest that the largest effects of the mindfulness intervention are seen in individuals with uncontrolled hypertension (i.e., above 140/90 mmHg). By revising our recruitment approach to more intentionally target this study population, we can further explore the connection between mindfulness and blood pressure.

4. What is your assessment of how the changes will affect the overall risk/benefit ratio of the study and the willingness of individuals to participate?

We do not anticipate that the new recruitment material will effect the risk/benefit ratio. However, there is a potential risk involved in the direct mailing. Specifically, there may be some patients who do not wish to receive the mailing. However, no response is required. The cover letter included in the mailing also invites patients to follow up with their CPCP provider if they have any concerns or questions.

5. Does the requested amendment require new documents or changes to the approved consent form or other documents?

All of the revisions outlined in this amendment only pertain to study recruitment material. See below for a list of attachments.

Appendix 1	Infographic on Mindfulness and Health - *NEW*
Appendix 2	MB-BP Recruitment Card - *NEW*
Appendix 3	Direct Mailing Cover Letter - *NEW*

Appendix 1

NEW - Infographic on Mindfulness and Health

MINDFULNESS AND CARDIOVASCULAR HEALTH LAB

BROWN UNIVERSITY

"We provide research and education on evidence-based programs through which mindfulness can improve cardiovascular health."

WHAT IS MINDFULNESS?

"Paying attention in a sustained and non-judgmental way, to what is going on in your body, your mind, and in the world around you. It is being awake and aware, living in the present"

Jon Kabot-Zinn

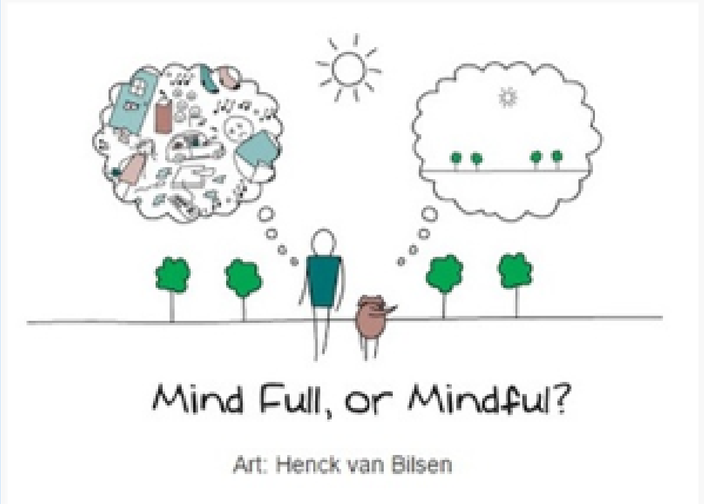

HOW CAN IT HELP?

Life can get crazy...

Sometimes we get the itch of a craving...

It can be different for everyone...

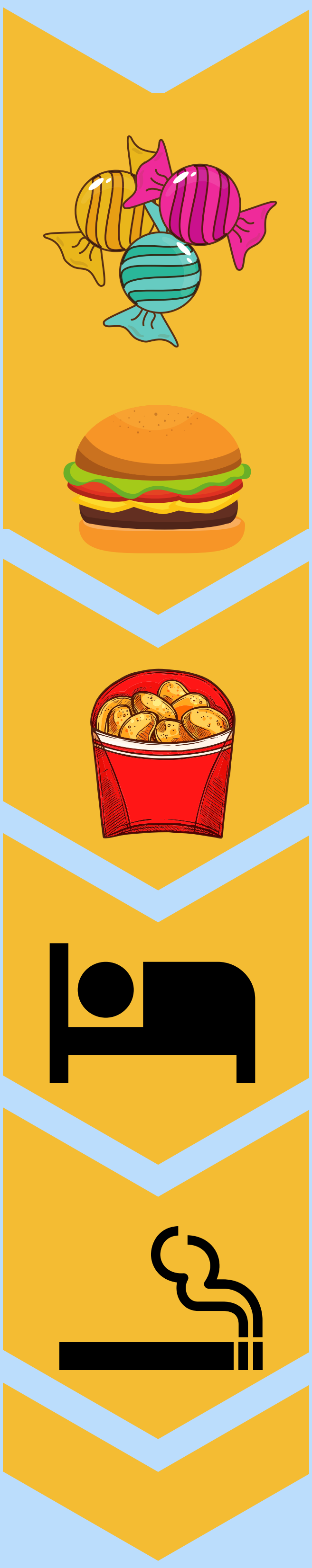

For some people it can be food...

sweet...

fatty...

or salty.

Others may have a craving for sleep and rest.

Some have a few drinks or smoke cigarettes.

Mindfulness gives us non-judgmental awareness in the moment allowing us to stop and see experiences as they are.

The "itch" is still there but we can make a decision on whether we would like to act on it or not.

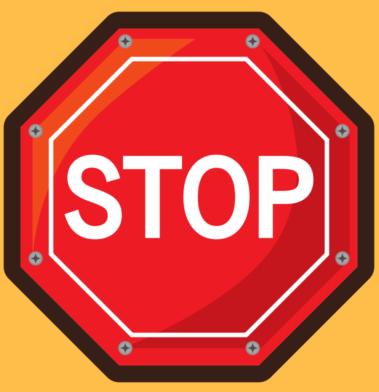

http://www.mindfulhearthealth.org/

HOW DOES IT WORK?

Researchers believe that mindfulness can help us make decisions in our lives by improving our ability to practice three different things...

Attention Control

"I can be more focused on the good feelings in life"

Emotional Regulation

"I'm angry and sad but it won't control my actions"

Self Awareness

"This is where my mind is going. I'm worried what can I do?

SELF-REGULATION

"This is how I am feeling and that's OK. I'll choose what to do next."

Research has shown that those with high levels of mindfulness...

- Have an 86% chance of being in good cardiovascular health

  Loucks et al. Int J Behav Med. 2015

- Are 25% less likely to be obese

  Loucks et al. Int J Behav Med. 2015

- Have 17 mmHg lower systolic blood if hypertensive before learning mindfulness.

  Loucks et al. In Progress.

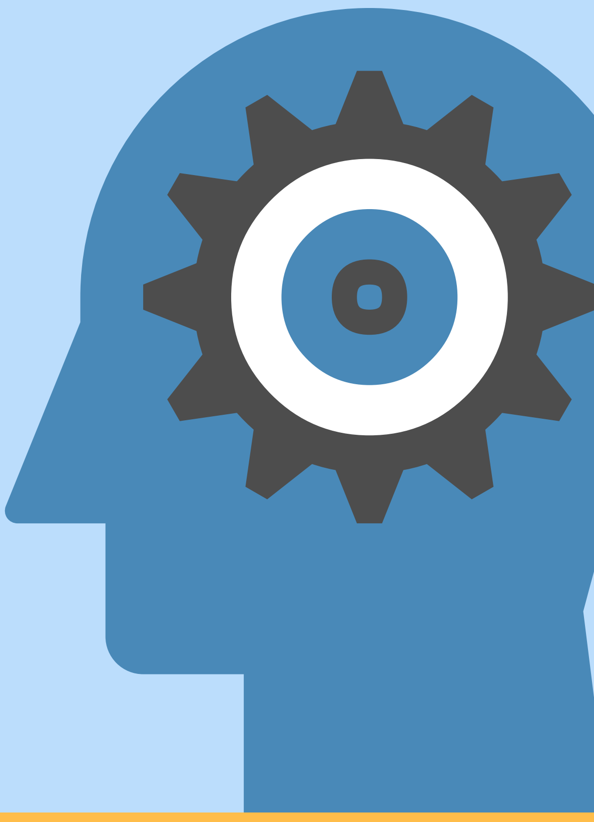

BROWN School of Public Health

For more information please contact us at:

Mindfulness and Cardiovascular Health Lab
Brown University School of Public Health
Box G-S121-2 Providence RI 02912

Phone: 401-400-4768
Email: mindfulness@brown.edu
Website: mindfulhearthealth.org

Appendix 2

NEW – MB-BP Study Recruitment Card

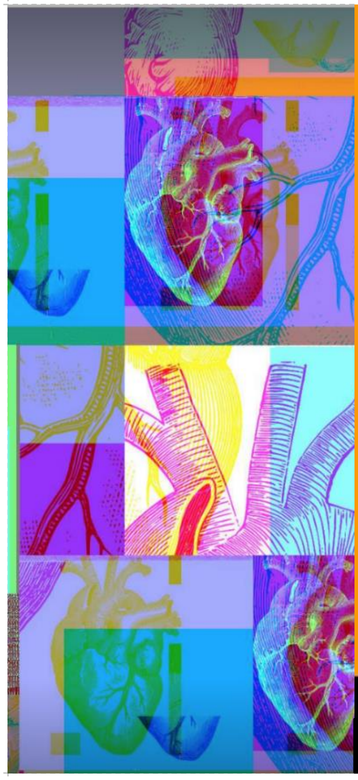

MINDFULNESS-BASED INTERVENTION

FOR BLOOD PRESSURE REDUCTION

Free to qualified participants

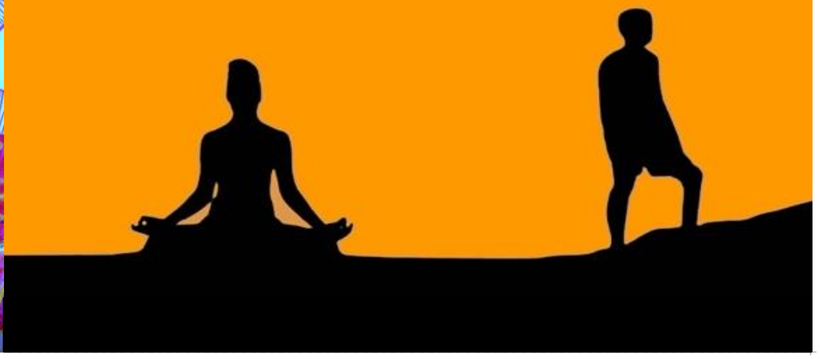

What is the MB-BP Program?
The Mindfulness-Based Blood Pressure Reduction (MB-BP) program is a 9-week course providing free training in mindfulness meditation, mindful movements, education about hypertension, and support for reducing hypertension risk factors. It is part of a larger research study funded by the National Institutes of Health. The course consists of 9 weekly sessions that are 2.5 hours, as well as an all-day weekend retreat.

Why are we doing this?
The research study is testing whether the 9-week intervention lowers blood pressure.

How do you qualify?
To see if you may qualify for the study, contact our office by phone or email. People who have a current regular meditation practice (i.e. meditate more than once per week) are not eligible.

What would I be asked to do?
In addition to the 9-week course, research participation includes interviews, questionnaires and health measurements, such as blood pressure, height, and weight, completed both before and after the mindfulness program.

For more information, please contact:

Brown University Mindfulness and Cardiovascular Health Lab:
Phone: 401-400-4768 (*call or text*)
Email: mindfulness@brown.edu
Website: www.mindfulhearthealth.org

BROWN
School of Public Health

Appendix 3

NEW – Cover Letter for Direct Mailing

BROWN
School of Public Health

Mindfulness & Cardiovascular Health Lab
Brown University School of Public Health
Centers for Epidemiology &
Environmental Health

[DATE]

PATIENT NAME
ADDRESS

Dear [PATIENT NAME]:

You have been identified as someone who would potentially be eligible for an important and novel research study at Brown University known as the Mindfulness-Based Blood Pressure Reduction (MB-BP) program.

The MB-BP Study is a free 9-week course designed for individuals who wish to lower their blood pressure. The program provides weekly training in mindfulness meditation, mindful movements, education about hypertension, and support for targeting hypertension risk factors, such as diet, physical activity, stress, and medication adherence. As a research participant you would also be given cash incentives (total of $100) for your time and effort.

Please note that this study is being conducted by Brown University and is funded by the National Institute of Health (NIH). The Center for Primary Care and Prevention is not conducting the research nor does your health care provider have a direct interest in the study.

We invite you to take a moment to read through the enclosed information cards. To find out more about the MB-BP study and to see if you are eligible, contact the Brown Mindfulness & Cardiovascular Health Lab at 401-400-4768 or email at mindfulness@brown.edu. You can also visit the study webpage: www.mindfulhearthealth.org. If you have any questions or concerns regarding your blood pressure and overall health, please don't hesitate to contact your primary care provider directly.

Sincerely,

[PATIENT PRIMARY CARE PHYSICIAN]	Eric B. Loucks, Ph.D.
Center for Primary Care and Prevention	Principal Investigator, MB-BP Study
Memorial Hospital of Rhode Island	Director, Brown Mindfulness Center
111 Brewster Street	Associate Professor, Department of Epidemiology
Pawtucket, RI 02860	Brown University

DONE. SEE ATTACHED.

6. As a note, the study title listed on the *Modification Request* form is old, as this title changed with Amendment #6. You do not need to update the title on this form, but to avoid confusion, please be sure the title is correct with future submissions.

   THANK YOU. WE WILL MAKE SURE TO USE THE UPDATED TITLE MOVING FORWARD.

Safety Plan Questions/Requests:

1. The safety plan does not address two of the new measures: *Childhood Trauma Questionnaire* and *Connor-Davidson Resilience Scale 10 (CD-RISC-10)*. Please include these measures, and make any appropriate changes to the safety plan.

   THE CD-RISC-10 IS DESIGNED TO MEASURE RESILIENCY. IT IS NOT INTENDED TO SERVE AS A MENTAL HEALTHER SCREENER. HOWEVER, IF SOMEONE DOES EXPERIENCE DISTRESS AFTER COMPLETING IT OR ANY OF THE MEASURES IN THE HOME ONLINE SURVEY, HE/SHE WILL BE PROVIDED WITH RESOURCES AT THE END OF THE SURVEY (SEE END PAGE SCRIPT IN THE ATTACHED HOME SURVEY)

   IN ADDITION TO THESE RESOURCES, WE HAVE NOW ALSO ADDED A PROMPT WITHIN THE ONLINE SURVEY FOLLOWING THE CHILDHOOD TRAUMA QUESTIONNAIRE (CTQ) WHERE INDIVIDUALS WHO SCORED AS MODERATE OR SEVERE ON THE CTQ SCALE WILL BE OFFERED THE OPTION OF BEING FOLLOWED UP BY STUDY STAFF / STUDY CLINICIAN SEE ATTACHED HOME SURVEY AND SAFTEY PROTOCOL FOR THESE REVISIONS.

2. The "Uncontrolled Hypertension" section (pg. 1, 2nd bullet) says that you will collect provider information from participants and notify the provider of the BP reading. I was unable to find this addition in the consent document. If it is not there, please add it. Please also add if contacting a participant's provider is optional and what will happen if a participant does not what their research data shared with their provider.

   WE HAVE ADDED THIS INFORMATION TO THE CONSENT DOCUMENT (SEE PG.5, 2ND PARAGRAPH OF THE ATTACHED REVISED CONSENT FORM). IF A PARTICIPANT DOES NOT WISH US TO CONTACT HIS/HER PCP, THEN THEY WILL STILL BE ABLE TO PARTICIPATE IN THE STUDY. NOTE THAT THE SAFETY PROTOCOL FOR EXTREMELY HIGH AND LOW BLOOD PRESSURE READINGS STILL HOLDS.

3. The "Uncontrolled Hypertension" section (pg. 1, 3rd bullet) describes assisting uninsured participants without providers with the process of finding providers and health insurance. This can be a large and time-consuming task. How will resources be allocated for this process?

   WE ARE WILLING TO DEVOTE STAFF TIME TO THIS EFFORT (UP TO AN HOUR PER PARTICIPANT IF NEEDED). HOWEVER, GIVEN OUR PAST STUDY POPULATION WE DO NOT ANTICIPATE THIS SITUATION WILL COME UP OFTEN. THE DEPARTMENT OF HEALTH ONLINE RESOURCE THAT WE INTEND TO USE ALLOWS FOR PROVIDER SEARCHES TO BE MADE BY CITY/TOWN OR BY HEALTH INSURANCE CARRIER. THERE IS ALSO A LIST OF HEALTH CLINICS THAT HAVE EXPERIENCE SERVING THE UNDER- / UNINSURED. ALTHOUGH WE ARE WILLING TO ASSIST A PARTICIAPNT WITH FINDING CARE, WE WILL NOT OVER PROMISE ON THIS EFFORT. THE CONSENT FORM IS FORTHRIGHT IN STATING THAT WE CANNOT GUARANTEE SUCCESS.

4. The "Irregular ECG Reading" section (pg. 2, 2nd bullet) states that participants with "possible Atrial Fibrillation" will be asked to call their provider and then call 911. Is it appropriate to call the provider first, before 911? What if the participant does not want their provider or 911 to be called? Should this decision be left up to the participant?

   THIS MIGHT HAVE BEEN A MISCOMMUNICATION. RATHER THAN CALL 911 AND THEN THE PROVIDER, WE INTEND TO ACTUALLY PROVIDE THE PARTICIPANT THE OPTION OF WHO WE FOLLOW UP WITH (911 OR HIS/HER HEALTH CARE PROVIDER). TO CLARIFY FURTHER, THIS IS ONLY IF SYMPTOMS ARE PRESENT (E.G., CHEST PAIN, SHORTNESS OF BREATH, DIZZINESS, PASSING OUT). A PARTICIPANT CAN ALWAYS REFUSE FOLLOW UP. REGARDLESS, PER OUR SAFETY PROTOCOL, WE WILL SEND THE ECG READING TO OUR STUDY CARDIOLOGIST FOR REVIEW.

5. New language in the "Beck Anxiety Inventory (BA)" and "Depressive Symptomatology (DS)" sections refer to Dr. Fynn's phone number and "your phone #." To whom is "your" referring?

   ‘YOUR’ IN THIS CONTEXT IS REFERRING TO THE RESEARCH ASSISTANT ADMINISTERING THE IN PERSON ASSESSMENT.

6. The "Depressive Symptomatology (DS)" section (3rd paragraph) says that participants will be asked if they would like to talk to the study clinician. This

decision should not be left up to the participants, especially since they may need to talk to someone but not verbalize it. Please revise the plan to state that Dr. Flynn will be contacted to assess the participant.

WE HAVE MADE THIS CHANGE AS REQUESTED. TO CLARIFY WE ALWAYS PASS ALONG THE DEPRESSIVE SYMPTOMOLOGY RESULTS TO OUR STUDY CLINICIAN FOR REVIEW IF A SAFETY FLAG COMES UP.

Brown University
Research Protections Office
Institutional Review Board
Modification Request

Date of Request: 11/6/17 **Investigator's Name and Title:** Eric Loucks, PhD, Assistant Professor

Study Title: Mindfulness-based Blood Pressure Reduction (MB-BP) Study (#1412001171)

Original Type of Review: ☐ Exempt ☐ Expedited ☒ Full Board

1) **Provide a brief lay summary of the overall project. Include enough detail to allow the IRB to evaluate the requested change(s) within the context of the overall project.** (Attach summary to this form)

   See attached summary

2) **Provide a detailed description of the changes being requested** (Use additional pages, if necessary):

   See attached summary

3.) **State the reason (justification) for the requested modification.** (Use additional pages, if necessary):

   See attached summary

4.) **What is your assessment of how the changes will affect the overall risk/benefit ratio of the study and the willingness of individuals to participate?**

   See attached summary

5.) **Does the requested modification require new documents or changes to the approved consent form or other documents?**

   X Consent/assent documents (attach revised version with changes highlighted)
   ☒ New/revised instruments (attach - if revised, highlight changes)
   ☐ New/revised advertising materials (attach - if revised, highlight changes)

Do you have a conflict of interest on this project according to Brown's policy? ☐**YES** ☒**NO**

If YES, has this conflict been previously disclosed to the IRB? ☐**YES** ☐**NO**

PI signature: [signature] Date: 11/6/17

1. **Provide a brief lay summary of the overall project. Include enough detail to allow the IRB to evaluate the requested change(s) within the context of the overall project.**

Original lay summary providing broad overview of the project:

The World Health Organization reported that suboptimal blood pressure (BP) is responsible for more than half of cardiovascular disease mortality world-wide. Furthermore, greater than half of those with hypertension have uncontrolled BP. *A 2009 Institute of Medicine report recommended prioritizing research to "Compare the effectiveness of mindfulness-based interventions (e.g. yoga, meditation, deep breathing training) and usual care in treating… cardiovascular risk factors."* Evidence-based mindfulness interventions, including Mindfulness-Based Stress Reduction, may have some effects on blood pressure, where a recent meta-analysis and systematic review of 4 randomized controlled trials demonstrated significant effects, but evidence of heterogeneity in effect sizes. The methodologically highest quality studies had the smallest effect sizes (range 0-5 mmHg). Mindfulness-Based Stress Reduction (MBSR) has been customized to a number of disease processes, such as Mindfulness-Based Cognitive Therapy for patients with recurrent depression, and Mindfulness-Based Relapse Prevention for patients with substance use addictions. Effect sizes have been increased by customizing mindfulness interventions to diseases of interest. The same may be true for hypertension, *however mindfulness interventions customized for prehypertensive/hypertensive patients have never been investigated. Until methodologically rigorous studies to evaluate customized interventions for hypertension are performed, we will not know if the observed preliminary effects of general mindfulness interventions on blood pressure reduction could be much more effective with a tailored approach.* Consequently, we propose to conduct a stage I behavioral therapy intervention study to evaluate whether MBSR customized to prehypertensive and hypertensive patients has the potential to provide clinically relevant reductions in BP. Consequently the specific aims are:

Stage 1a: Therapy Development/Manual Writing

1. To outline and evaluate key novel elements of mindfulness-based hypertension therapy (MBHT), customized from the evidence-based MBSR. *We hypothesize that the most important novel element will be generation of mindfulness skills specifically applied to hypertension risk factors such as diet, physical activity, obesity, alcohol consumption and antihypertensive medication adherence.* This aim will be achieved using (1) focus groups of participants undergoing the MBHT behavioral intervention, (2) discussion with experts (including cardiologists, epidemiologists, mindfulness experts, mindfulness intervention instructors) prior to, and following pilot testing of MBHT in participants, and (3) clinical judgment of the investigators performing the intervention.
2. To determine effectiveness of MBHT on primary outcomes (systolic blood pressure, retention rates, recruitment rates, and adverse effects) and secondary outcomes (hypertension risk factors such as diet, physical activity, obesity, and antihypertensive medication adherence) in hypertension subgroups, specifically participants with (1) prehypertension, (2) controlled hypertension, and (3) uncontrolled hypertension. Initial decisions about the targeted sample based on hypertension status will be made.
3. To develop an MBHT therapist manual and training program, including procedures for training, supervising, and evaluating therapists. Furthermore, acceptable therapist characteristics will be developed. The manual and training program will include themes such as specification of unique and common elements of MBHT *vs.* other interventions, description of interventions excluded from MBHT, and specification of key treatment

parameters such as frequency and duration of treatment, session length, topics addressed, sequence of sessions, as well as therapist adherence and competency measures. The MBHT training will consist of a therapist manual, a formal didactic training seminar, and at least one closely supervised training session.

Stage Ib: Pilot Trial

4. To determine whether a mindfulness-based hypertension therapy (MBHT) intervention, customized from the evidence-based MBSR, has promise to be an effective behavioral therapy for participants with hypertension and/or prehypertension. We will perform a randomized controlled pilot trial for MBHT *vs.* enhanced usual care control. *We hypothesize that MBHT will have adequate recruitment rates (≥10% of prehypertensive/hypertensive participants invited from physicians' offices), fairly low drop out rates (<15%), and medium effect sizes (e.g. 5-10 mmHg systolic BP) for reduction in blood pressure.*

These findings will provide publishable pilot data that will inform future randomized clinical trials that evaluate effects of MBHT on long-term changes in blood pressure *vs.* usual care and active control groups. <u>*If proven effective, MBHT could be offered as a complementary program in the prehypertensive/hypertensive patient population that contributes to over half of the cardiovascular disease mortality world-wide.*</u>

<u>Project update as of November 2017</u>:

The Mindfulness-Based Blood Pressure Reduction (MB-BP) Study, formerly known as Mindfulness-Based Hypertension Therapy (MBHT), is currently in Year 2 of a five year NIH UH2 grant.

Data collection for Stage 1a of the project has ended and analysis is on-going. In total we ran three separate Mindfulness-Based Blood Pressure reduction (MB-BP) intervention courses, with a total sample size of 43 eligible participants who both enrolled and completed the 9-week intervention. We had eight eligible participants enroll in the study who later decided to not complete the course.

In June 2017 we began enrollment for the next phase (referenced as Stage 1b above, but now called 'Stage 2a') of the study, which is to conduct a Randomized Controlled Trial (RCT) with *enhanced usual care*. This phase will continue for the next 12-18 months, at which point we will transition to the third and final phase, an RCT with an *active* control (i.e., Mindfulness-Based Stress Reduction - MBSR) as well as ambulatory blood pressure monitoring.

2. Provide a detailed description of the changes being requested (Use additional pages, if necessary):

This amendment covers two requests:

(1) First, we are requesting permission to modify the already approved focus group discussion questions into a one-on-one qualitative phone interview (see enclosed "Semi-structured qualitative phone interview"). Our intent is to then randomly select and re-contact Stage 1 participants, who did not participate in the focus group discussions, and invite them to take part in the one-on-one qualitative phone interviews instead. We would attempt to complete ten interviews. Participants who complete the phone interviews would be mailed $25 gift cards for their time and effort.

(2) Second, we would like to re-contact participants who completed the course and invite them to come back into the office for a quick two year in-person assessment. This optional assessment is estimated to take 20-30 minutes to complete and would be a sub-set of what is already approved by the IRB (i.e., blood pressure, weight, hypertension medication questions, and mindfulness practice questions). Participants would be given $50 USD for this additional component. For participants who have already been consented, we would have them sign the attached addendum to the consent form. For newly enrolled participants we would ask them to sign the attached revised consent form. It is already part of the approved study protocol and screening process to ask participants if we can keep their information on file to contact them about possible future studies or opportunities to participate in research. We would only recontact participants who gave us permission to do so.

3. State the reason (justification) for the requested amendment:

(1) The data collected in the proposed phone interviews will complement the qualitative data previously collected in the focus group discussions. It will also hopefully help to address any potential selection bias due to the more engaged and enthusiastic participants opting to take part in the focus group discussions.

(2) Analysis of the one year follow up data has been very promising, showing sustained reductions in blood pressure and other important outcomes. There are very few research studies that explore sustained effects at 12 months or even 24 months. Recontacting participants to have them complete a 2 year follow up would offer novel research findings to the field of mindfulness and cardiovascular health.

4. What is your assessment of how the changes will affect the overall risk/benefit ratio of the study and the willingness of individuals to participate?

We do not anticipate any shifts in the risk / benefit ratio of the study. Participation in either the phone interviews or the 2 year follow ups is optional. Study participants who do chose to complete a 30 minute phone interview will be given a $25 gift card. Participants who opt to come back in for a two year follow up will be given $50 USD.

5. Does the requested amendment require new documents or changes to the approved consent form or other documents?

See enclosed attachments related to this amendment:

Appendix 1 - Protocol for semi-structured qualitative phone interview scripts and talking points.

Appendix 2 – Addendum to consent form to be signed by already enrolled participants

Appendix 3 – Revised Informed Consent (v.2.2) – with track changes

Appendix 4 – Two year in-person follow up assessment

Appendix 1 -
Semi-structured qualitative phone interviews

Modified based on already approved focus group discussion protocol.

MB-BP qualitative phone interview protocol

Semi-Structured Script and Talking Points
"Hello, my name is __________ and I am calling from Brown University regarding the Mindfulness Based Blood Pressure Reduction Study that you took part in just over a year ago. As we begin to evaluate the intervention we would like to get a better understanding of what worked and didn't work by conducting personalized one-on-one phone interviews with yourself and other graduates of the program.<br><br>The interview would be done by phone, would take 20 minutes to a half an hour to complete, and could be scheduled at a time that is convenient for you. You would also receive a $25 gift card for your time. Would you be willing to offer feedback on your experiences with the mindfulness program?"<br><br>TALKING POINTS –<br><br>● **Confidentiality**: As a reminder, everything you say will remain confidential. No one outside of our project will be able to see your answers and your name and other identifying information will not be used in any reports or publications.<br><br>● Why and How? "We are hoping to get your feedback about the mindfulness course and use it to make the program more effective. Please feel free to share your point of view and know that we welcome both positive and negative feedback. There are no right or wrong answers just your perspective about the program."<br><br>**Important points:**<br>○ We are trying to improve the intervention<br>○ Discussing your opinions on the different activities<br>○ Please share your point of view<br>○ No wrong answers<br>○ We equally welcome positive and negative feedback<br><br>● **Suggestions:** "We would like to record this call with your permission so that it can be transcribed later by members of our team. If you would prefer not to be recorded, please let me know. Also, please understand that any report we write regarding your feedback and what we hear from you today will not be identified with you in any way and remains anonymous."<br><br>Suggestions to help us have a good discussion<br>■ Speak up<br>■ Audio recording if that is ok<br>■ Any report that we write about what we hear today will not be associated with your identity<br><br>● **What to expect:** "So before we begin I just want to let you know that we will be asking you a series of open ended questions. My role is listen and ask questions to better understand your experiences. So I might ask you for clarification or more detail about a comment but your thoughts are the important part of this discussion. However, in the interest of getting to all the questions I may need to move us along pleas know that this is only due to time considerations."<br><br>○ My role is to listen ask questions that help to understand your experience<br>○ In the interest of time, I may move to the next question to get your thoughts regarding the whole program<br><br>Any questions before we begin?
Discuss Class Overview: Each sessions central activity<br><br>"First I am going to read through a list of the major class activities you participated in. While I read through the activities I would like you to think about which was the most memorable for you and why."<br><br>"So, hearing the course activities, which is most memorable for you? Why?"
"Next I have a series of 4 questions for you about your experiences in the program."<br><br>1. What was most helpful about this course, and why?
2. After going through this mindfulness intervention, what is your understanding of how it works to improve your blood pressure?
3. We want to make this intervention better. You have been through it once. How do you think we can make it better?
4. Every instructor can improve. How can this instructor improve?
Read the survey: record responses.<br>• Each weekly session was 2.5 hours long. Do you think the session should be 2 hours, 2.5 or 3 and why?<br>• The retreat day was scheduled to be 7.5 hours long. How long do you think the day should be (5,6,7, or 8) and why?<br>• Please provide us with an additional feedback we may not have asked about at this time.

Appendix 2 -

Addendum to Informed Consent

To be signed by participants who have already enrolled in the study and who opt to complete the optional two year follow up assessment

Brown University
Consent to Participate in a Research Study
Addendum to provide additional information to subject after original consent and to seek permission to complete a two year follow up assessment

---

IRB Study #__1412001171__________
Consent Form Version Date: _12/6/2017__
Title of Study: Mindfulness-Based Blood Pressure Reduction (MB-BP) Study
Principal Investigator: Eric B. Loucks, PhD__

Study Contact telephone number: 401-400-4768
Study Contact email: mindfulness@brown.edu

The following information should be read as an addition to the original Consent form that you read and signed at the beginning of the study. Unless specifically stated otherwise in the following paragraphs, all information contained in that original Consent Form is still true and remains in effect. Your participation continues to be voluntary. You may refuse to participate, or may withdraw your consent to participate at any time, and for any reason, without jeopardizing your future care at this institution or your relationship with your health care provider.

New or additional information
You are being asked to participate in an optional two year follow up assessment that will take place in-person at the Brown University Mindfulness & Cardiovascular Health Lab located at 121 South Main Street, Providence, RI 02912. The two year follow up assessment is estimated to take around 20 to 30 minutes to complete and will consist of a subset of measures that you previously completed as a participant in the MB-BP Study. Specifically, we will be taking your blood pressure and weight as well as asking you a series of questions about hypertension medications and mindfulness practices.

Subject's Agreement:
I have read the information provided above. I have asked all the questions I have at this time. I voluntarily agree to continue to participate in this research study.

_______________________________________ ______________
Signature of Research Subject Date

____
Printed Name of Research Subject

_______________________________________ ______________
Signature of Research Team Member Obtaining Consent Date

____
Printed Name of Research Team Member Obtaining Consent

Appendix 3 -
Revised Informed Consent
(v.2.2 – with track changes)

For newly enrolled participants to complete
at time of screening.

The Mindfulness-Based Blood Pressure Reduction (MB-BP) Study

Agreement to Participate in a Research Study

Investigation of the Effects of Mindfulness on Blood Pressure and Well-Being

You are being asked to take part in a Brown University research study about the effects of mindfulness and hypertension education on blood pressure and risk factors for hypertension. This form will explain the purpose of the study, how the study will be carried out and what you will be expected to do. It will also explain the possible risks and possible benefits of being in the study. If any part of the following description is not clear to you, you are encouraged to contact the researcher to answer any questions before you decide whether to take part in the study. If you decide to participate, please fill out and sign the last page of this form.

1a. Nature and Purpose of the Study

The purpose of the study is to investigate the impact of mindfulness practices and health education on blood pressure. You have been selected for this study because you expressed interest in the project and because you met entrance criteria for having prehypertension or hypertension, or another cardiovascular risk factor that could be influenced by this program. Eligibility for the study is still being assessed. Therefore, it is possible you may not be eligible for the study even after signing this consent form. Your participation in this study is voluntary and can be withdrawn at any point in the project.

In order to assess the effects of the customized mindfulness intervention, you will be asked to complete some questionnaires and laboratory assessments before and after the intervention. Specifically, assessments will be completed at: baseline, 10 weeks, 6 months and 1 year. To express our gratitude for participation, you will be given $25, $25, and $50 (up to $100 USD total) at the 10 week, 6 month and 12 month follow ups respectively. As part of the study you will also be given a wireless blood pressure monitor (estimated value of $90) to use throughout the study. There is an optional 2 year follow up that you may be invited to complete, for which you will be given an additional $50 USD.

This is a Randomized Control Trial. Participants enrolled into the study will be randomly assigned to one of two groups: (1) the intervention group or (2) the wait-list control group. The wait-list control group will be given the opportunity to participate in the intervention after the six month follow up assessments are completed. Both the intervention and the control group will be asked to participate in the research assessments.

1b. Explanation of Procedures

If you agree to participate, you will be asked to consent to the following:

1) Participation in an interview in which you will be asked questions about past and present mental health, including depression and suicide (previously completed with your verbal consent).

2) Completion of an in-person screening assessment, during which your blood pressure, height, weight and other basic demographic and health data will be collected and assessed in order to determine eligibility for the study.

3) Completion of questionnaires administered in-person and online that ask about a wide range of topics, including your diet, physical activity, smoking, medication use, personality, emotions, attention and past experiences, including stressful or traumatic experiences. These questions will probe sensitive psychological areas, including physical, emotion and sexual abuse. These questionnaires may take up to 3 hours to complete. By completing the interviews and questionnaires, you are giving the researchers permission to use the information you have provided. You have the right not to answer any of the questions.

4) Directly assessed blood pressure, heart rate, height, weight, physical activity, and antihypertensive (blood pressure) medication use at baseline and after the mindfulness course. Physical activity will be assessed for a week at a time using small actigraphy monitors (i.e., Fitbits) that attach to your wrist. If you take antihypertensive medication, we will provide you with an electronic bottle cap that will automatically record when the pill bottle is opened during the study. This will help us measure how often the medication is used. We will also provide you with a wireless blood pressure monitor and will ask that you take your blood pressure at home systematically during each of the research assessment periods (i.e., baseline, 10 week, 6 month and 12 month).

5) You will be asked to perform some cognitive tasks. Some of these tasks may involve computer-based tests of attention or decision-making. Together these tests may take as long as 20 minutes.

6) During the in-person assessments you will also be given a battery of stress tests that are designed to induce a stress response so that we can monitor your cardiovascular response and recovery.

7) Attendance at an information session that is to be held within two weeks of the start of the intervention.

8) If randomized into the intervention group, you will participate in the mindfulness program, which consists of 9 weekly sessions of 2.5 hours each and will include one 7.5 hour weekend retreat. Daily at home practice assignments may take as long as one hour and consist of practicing mindfulness exercises with the aid of a guided meditations and completing worksheets related to stress, thoughts, and common reactions to various types of events. If you are randomized into the wait-list control group, you will be invited to take part in the mindfulness class after the completion of the 6 month follow up assessments.

9) Class sessions may be audio taped so we can analyze the quality of the treatment you receive. The recordings will be transcribed so that we may analyze the text. The recordings will be identified by study number, will only be heard by study staff and will be destroyed after transcription.

10) You may be asked to complete a few short questionnaires each week during the 9 week condition.

11) After 10 weeks from the start of the intervention, you will be asked to complete questionnaires and return to the laboratory to repeat the same procedures for a second day of testing. If in the intervention arm, you will also be invited to participate in a focus group to share any advice you may have on how to improve the intervention.

12) Six months and one year after the beginning of the study, you will be asked to return to the laboratory to repeat the same testing procedures.

~~12)~~13) In addition to the six month and one year follow up, you will also be re-contacted to participate in an optional, very brief two year follow up assessment. The two year follow up assessment is estimated to take around 20 to 30 minutes to complete and will consist of a subset of measures that you would have previously completed as a participant in the MB-BP Study. Specifically, we will be taking your blood pressure and weight as well as asking you a series of questions about hypertension medications and mindfulness practices. For this additional follow up you will be given $50 USD.

Table Summarizing Activities and Time Commitment for this Study.

**Activity**	**Estimated Time Commitment**
In-person screening assessment	0.5 hours
Baseline<br>In-person assessment<br>Online questionnaire<br>At home health monitoring (e.g., fitbit, BP, etc.)	 2.0 hours<br>1.0 hours<br>1.0 hours
Information session	1.0 hour
Intervention*<br>Mindfulness course<br>Home practice assigned during course<br><br>**Intervention group only; control group will be invited to take part in a class post 6 month follow up but it will not be required as part of the study.*	Nine 2.5 hour sessions<br>7.5 hour all day retreat<br>Up to 1hr daily home practice assignments<br>*Total course time: 30.0 hours*<br>*Max. practice time: 48 hours*
Focus group participation post intervention (*intervention group only*)	1.5 hours
Follow Ups – 10 week, 6 month, 12 month<br>In-person assessments<br>Online questionnaires<br>At home health monitoring (e.g., fitbit, BP, etc.)	Total follow up time:<br>6.0 hours<br>3.0 hours<br>3.0 hours
Follow Up – 2 year (in person only)	30 minutes
**TOTAL ESTIMATED TIME COMMITMENT**	~~17.5~~18.0 hours – CONTROL<br>97.~~0~~5 hours - INTERVENTION

Feedback:

At the end of the study, you will receive individual feedback about the changes that occurred since the first assessment. Specifically, you will receive an individualized handout listing % change (increase or decrease) on scales of attention, stress, mood, health behaviours, weight, and blood pressure across the study.

Uncontrolled Hypertension:
If during the in-person assessments it appears that you have uncontrolled hypertension (your average systolic blood pressure reading is 140 mmHg or greater and/or your average diastolic blood pressure is 90 mmHg or greater) AND you indicate to us that you are not currently being treated for hypertension, then we will be requesting your permission to contact your health care provider to notify him/her of the blood pressure results. If you do not have a health care provider and/or do not have health insurance, our staff will provide you with resources to help you search for one; although we cannot guarantee that we will be able to find you one nor that it will be free. It is your choice on whether or not you would like us to follow up with your health care provider. Your participation in the study is not contingent on this communication; however, it is our recommendation that all individuals with uncontrolled hypertension be under the care of a health care professional.

2. Discomforts and Risks

The risks to you in this study are small. The questionnaires used in the study are routine, standardized forms for epidemiologic research. Certain questions may be upsetting as they may probe sensitive psychological areas and inquire about upsetting or traumatic events, including physical, sexual or emotional abuse and/or current psychiatric symptoms. The cognitive tests and stress battery may also invoke a stress response that may be uncomfortable. All aspects of the study are voluntary; you have the right to skip anything during the study that makes you uncomfortable.

Meditation-based interventions may results in discomfort with attention to unpleasant thoughts, feelings or body sensations. Some individuals may experience an initial increase in undesirable feelings with increased attention to them.

It is possible that injuries could be sustained during the study either from the gentle mindful movements (i.e., yoga), or from physical activities that participants engage in as a way to reduce blood pressure. To help limit this, you will receive a handout showing the yoga poses that will be offered during the course that you can show your health care provider so that they can advise on which poses to do, and which to avoid. Modifications of poses will be available as needed. None of the poses (or the yoga as a whole) are mandatory to be done. You will also be encouraged to explore physical activities that promote strength and conditioning as a way to reduce blood pressure. You will be encouraged to not go beyond any physical limits of your body, and will be encouraged to ask your healthcare provider about advised physical activities and mindful movements if you have any physical limitations.

While physical and mental injury is always a possibility the potential for harm is limited. Note that a research injury is any physical or mental injury or illness caused by your participation in

the study. If you are injured by a medical treatment or procedure that you would have received even if you were not in the study, that is not a research injury. To help avoid research injury and potential added medical expenses, it is important to follow all study directions carefully. If you are covered by insurance and suffer a research injury, it is possible that some or all of the costs of treating your condition could appropriately be billed to your insurance company. If such costs are not covered your health insurance company, it is possible you would have to pay for these costs out of pocket. Brown University's policies do not cover payment for such things as lost wages, medical care expenses, or pain and suffering.

Precautions should be taken to avoid injuries. If you do become injured during the study, you should call your doctor immediately. You should also alert the study staff that you have been injured. Heart attack and sudden death related to heart problems have been known to occur in people while they are exercising. This is very rare, however. Estimates of sudden cardiac death range from 0 to 2 per 100,000 hours. However, the researchers cannot guarantee that no complications will happen to you.

3. Benefits

We cannot and do not guarantee or promise that you will receive any direct benefits from this study. However, participation in the study creates the potential benefit of a) identifying effective treatments for elevated blood pressure, b) gaining knowledge of the effects of mindfulness practices, c) receiving information about your psychological and physical functioning. As part of the study, you will receive a wireless blood pressure monitor that will be yours to keep. This monitor may provide additional opportunity to monitor your blood pressure at home, which may benefit your health by providing additional biofeedback.

4. Alternative Therapies

A number of different therapies, including antihypertensive medication, diet changes, physical activity, and reducing excessive alcohol consumption may also be beneficial for reducing blood pressure. Education about these therapies are integrated into this course, but other forms of these alternative therapies are also available in the community.

5. Confidentiality

Your responses for this study will be kept confidential. All data that we collect will be linked to a study ID# instead of your name. All questionnaires in this study will be filled out through an online survey or a paper version of the survey if you prefer. All of these questionnaires will be linked solely to your study ID#, so that your identity is protected and your answers are confidential. Although these measures have been taken to protect your personal information, complete confidentiality cannot be guaranteed when transmitting information over the internet.

While your confidentiality is protected to the extent of the law, there are limitations to confidentiality. If your questionnaire responses indicate that you pose a serious danger to yourself or to another person, then a collaborator (Dr. Ellen Flynn) who is a licensed psychiatrist, may contact you to discuss your responses and possible referral to a treatment provider. Questionnaire items that may warrant follow-up include endorsements of statements about

hurting yourself, any high scores in depression, anxiety, or other clinically significant problems. You should also know that there are times when the law might require the release of your responses without your permission. For example, State law requires researchers to report abuse or neglect of children to the Department of Children, Youth and Families (DCYF). State law also requires researchers to report abuse or neglect of people age 60 and older to the Division of Elderly Affairs.

The findings of the study may be used for medical publication. Your name will not be used in any published reports about this study. Results will be reported in a summarized manner in such a way that you cannot be identified. All personally identifiable information will be "de-identified" and only a unique code number will be used. Study records will be identified with a unique code number and initials. All study records and specimens will be stored in a secure storage area.

Keeping study records: The Principal Investigator for this study will keep your research records indefinitely for research purposes.

Certificate of Confidentiality: This research is covered by a Certificate of Confidentiality from the National Institutes of Health. The researchers with this Certificate may not disclose or use information, documents, or biospecimens that may identify you in any federal, state, or local civil, criminal, administrative, legislative, or other action, suit, or proceeding, or be used as evidence, for example, if there is a court subpoena, unless you have consented for this use. Information, documents, or biospecimens protected by this Certificate cannot be disclosed to anyone else who is not connected with the research except, if there is a federal, state, or local law that requires disclosure (such as to report child abuse or communicable diseases but not for federal, state, or local civil, criminal, administrative, legislative, or other proceedings, see below); if you have consented to the disclosure, including for your medical treatment; or if it is used for other scientific research, as allowed by federal regulations protecting research subjects.

The Certificate cannot be used to refuse a request for information from personnel of the United States federal or state government agency sponsoring the project that is needed for auditing or program evaluation by the National Center for Complementary and Integrative Health, which is funding this project. You should understand that a Certificate of Confidentiality does not prevent you from voluntarily releasing information about yourself or your involvement in this research. If you want your research information released to an insurer, medical care provider, or any other person not connected with the research, you must provide consent to allow the researchers to release it. The Certificate of Confidentiality will not be used to prevent disclosure as required by federal, state, or local law of situations of child abuse and neglect, or harm to self or others.

6. Refusal/Withdrawal

Participation is voluntary, and you may decide to opt out or refuse any part of participation, including not answering certain questions. If you decide now to participate, you can change your mind later and quit the study. The decision to not participate or to withdraw from the study will not adversely affect current or future interactions with Brown University. The decision to not participate or to withdraw from the study will also not adversely affect your relationship with your physician.

If you decide not to participate, or if you quit the study, we will provide you with referrals for alternative treatments, if desired.

7. Contact Information

If you have questions about study procedures at any time you may contact the researchers: Dr. Eric B. Loucks, email: eric.loucks@brown.edu, telephone (401) 863-6283. If you would like more information about the rules for research studies, or the rights of people who take part in those studies, you may contact the Brown University Human Research Protection Program, telephone number 1-866-309-2095 or 401-863-3050.

A description of this clinical trial will be available on http://www.Clinical Trials.gov, as required by U.S. Law. This Web site will not include information that can identify you. At most, the Web site will include a summary of the results. You can search this Web site at any time.

CONSENT FORM:
Please sign and return.

Please read the following agreement and sign to consent to participating.

I HAVE READ THE ABOVE DESCRIPTION OF THIS STUDY. ALL OF MY QUESTIONS HAVE BEEN SATISFACTORILY ANSWERED, AND, I AGREE TO PARTICIPATE IN THIS RESEARCH STUDY.

____
PRINT NAME

___________________________________ _________________
Signature of participant **Date**

STUDY PARTICIPANT CONTACT INFORMATION

Name (print):____

Permanent Address:____

Email(s):____

Telephone:________________________**(cell)** ________________________**(other)**

Appendix 4 -
Two year in-person follow up assessment

Estimated to take 20-30 minutes to complete and consists of a subset of already approved measures

MB-BP STUDY: 2 YEAR IN PERSON FOLLOW UP ASSESSMENT

PID. Participant ID # __________
BA01. Staff ID # __________
BA02. Today's date (MMDDYY): ___________

Blood Pressure:
BA03a. Blood pressure 1st reading, systolic blood pressure: _________ mmHg
BA03b. Blood pressure 1st reading, diastolic blood pressure: __________ mmHg
BA03c. Blood pressure 2nd reading, systolic blood pressure: _________ mmHg
BA03d. Blood pressure 2nd reading, diastolic blood pressure: __________ mmHg
BA03e. Blood pressure 3rd reading, systolic blood pressure: _________ mmHg
BA03f. Blood pressure 3rd reading, diastolic blood pressure: __________ mmHg

If the difference of the 2nd and 3rd systolic BP reading is 20 mmHg or greater OR the difference of the 2nd and 3rd diastolic reading is 10 mmHg or greater, then repeat the BP readings. Otherwise, skip to BA07.

BA06a. Repeated blood pressure 1st reading, systolic blood pressure: _________ mmHg
BA06b. Repeated blood pressure 1st reading, diastolic blood pressure: __________ mmHg
BA06c. Repeated blood pressure 2nd reading, systolic blood pressure: _________ mmHg
BA06d. Repeated blood pressure 2nd reading, diastolic blood pressure: __________ mmHg
BA06e. Repeated blood pressure 3rd reading, systolic blood pressure: _________ mmHg
BA06f. Repeated blood pressure 3rd reading, diastolic blood pressure: __________ mmHg

> Blood Pressure Safety Protocol – The out-of-range blood pressure values are as follows: systolic blood pressure >200 mmHg or <90 mmHg; diastolic blood pressure >110 mmHg.
>
> In absence of symptoms (chest pain, shortness of breath, dizziness, headache), for a SBP>200 or DBP>110 or both, we will strongly encourage participants to see their doctor right away or to go to urgent care. If there are symptoms, we will immediately call 911.
>
> In absence of symptoms (chest pain, shortness of breath, dizziness, passing out), for a SBP<90, we will strongly encourage participants to see their doctor right away or to go to urgent care. If there are symptoms, we will immediately call 911.
>
> Follow Safety Protocol for Uncontrolled Hypertension (140/90 mmHg or greater)

BA07. Blood pressure cuff size used: □ S □ Reg □ L □ XL
BA08. Arm that cuff was placed on: □ L □ R

BP monitor BP monitor used:
- ❍ Unit #1 - HEM-705CP (1)
- ❍ Unit #2 - HEM-705CP (2)
- ❍ Other (specify: (3) ____________________

We are now going to take your weight. *Have ppt remove his/her shoes and empty his/her pockets. Remove bulky clothing as well.*
BA10. Weight: ______ . __ lb (one decimal place)

Medications (ME)

The next questions are about medications used to treat hypertension or high blood pressure.

ME01. Not including vitamins and supplements, do you currently take any prescription medications or over-the-counter drugs for high blood pressure?

- ☐ No
- ☐ Yes
- ☐ Don't know
- ☐ Prefer not to answer

ME01a IF NO, Have you ever taken medication for high blood pressure?

- ☐ No → ***skip to end of medications questions***
- ☐ Yes → ***skip to end of medications questions***

If you brought your medications with you, please take them out now as we will use them to complete the next section.

ME01b INTERVIEWER CHECKPOINT - Please select one:

- ☐ Rx info not known or available – ***skip to end of ME questions; will need to follow up***
- ☐ Ppt brought medications -- ***continue***
- ☐ Ppt did not bring medications but knows info – ***continue, may need follow up***

ME01c In total how many different medications and/or over the counter drugs do you currently take for high blood pressure? Again, do not count vitamins or supplements. _________ # of BP meds

Next we're going to ask you questions about each of the medications you currently take for high blood pressure.

ME02-10a. What is the name of the first prescription medication or over-the-counter drug that you take?

____

☐ Don't know ☐ Prefer not to answer

ME02-10b. What is the dosage form?

Oral
- ☐ Pill, tablet, or capsule
- ☐ Sublingual or orally-disintegrating tablet
- ☐ Liquid solution or suspension (drink, syrup)
- ☐ Powder

Topical
- ☐ Liquid, cream, gel, or ointment
- ☐ Ear drops (otic)
- ☐ Eye drops (ophthalmic)
- ☐ Skin patch (transdermal)

Inhaled
- ☐ Inhaler or nebulizer

Injected
- ☐ Injection

Suppository
- ☐ Rectal (e.g., enema)
- ☐ Vaginal (e.g., douche, pessary)

Other:
- ☐ Other _________
- ☐ Don't know
- ☐ Prefer not to answer

ME02-10c. How frequently do you take it?

- ☐ _________ times per day
- ☐ _________ times per week
- ☐ _________ times per month
- ☐ Don't know
- ☐ Prefer not to answer

ME02-10d. What is the strength? ***(Record strength of how it is actually taken, not how it is prescribed.)***_________ %

- ☐ _________ mg
- ☐ _________ mcg
- ☐ _________ grams
- ☐ _________ I.U.
- ☐ _________ Other unit: _____________
- ☐ Don't know
- ☐ Prefer not to answer

ME02-10f. Do you take it regularly or only as needed?

- ☐ Regularly
- ☐ Only as needed
- ☐ Don't know
- ☐ Prefer not to answer

ME02-10g. For how long have you been taking it?

- ☐ For _________ days
- ☐ For _________ weeks
- ☐ For _________ months
- ☐ For _________ years
- ☐ Don't know
- ☐ Prefer not to answer

ME02-10h. What is the medication used for?

- ☐ High blood pressure
- ☐ Prescribed for something other than high blood pressure but also acts as a hypertensive medication (*describe:* ____)

ME02-10i. ***Interviewer comments*:**

____

Repeat for all blood pressure medications the participant is currently taking.

<u>Self-report scales for participant to complete</u>:

PROMIS Global Health Scale v.1.2 – *standardized scale (found on proceeding pages)*

SLEEP – *1 question only taken from the Pittsburgh Sleep Quality Index*

Mindful Attention Awareness Scale (MAAS) - *standardized scale (found on proceeding pages)*

Five Facet Mindfulness Questionnaire (FFMQ) - *standardized scale (found on proceeding pages)*

SART and Mindfulness Practice questions *found on proceeding pages*

Global Health

Please respond to each question or statement by marking one box per row.

		Excellent	Very good	Good	Fair	Poor
Global01	In general, would you say your health is:	☐ 5	☐ 4	☐ 3	☐ 2	☐ 1
Global02	In general, would you say your quality of life is:	☐ 5	☐ 4	☐ 3	☐ 2	☐ 1
Global03	In general, how would you rate your physical health?	☐ 5	☐ 4	☐ 3	☐ 2	☐ 1
Global04	In general, how would you rate your mental health, including your mood and your ability to think?	☐ 5	☐ 4	☐ 3	☐ 2	☐ 1
Global05	In general, how would you rate your satisfaction with your social activities and relationships?	☐ 5	☐ 4	☐ 3	☐ 2	☐ 1
Global09r	In general, please rate how well you carry out your usual social activities and roles. (This includes activities at home, at work and in your community, and responsibilities as a parent, child, spouse, employee, friend, etc.)......	☐ 5	☐ 4	☐ 3	☐ 2	☐ 1
		**Completely**	**Mostly**	**Moderately**	**A little**	**Not at all**
Global06	To what extent are you able to carry out your everyday physical activities such as walking, climbing stairs, carrying groceries, or moving a chair?	☐ 5	☐ 4	☐ 3	☐ 2	☐ 1

22 August 2016
© 2010-2016 PROMIS Health Organization and PROMIS Cooperative Group

In the past 7 days…

		Never	Rarely	Sometimes	Often	Always
Global10r	How often have you been bothered by emotional problems such as feeling anxious, depressed or irritable?	☐ 5	☐ 4	☐ 3	☐ 2	☐ 1
		None	Mild	Moderate	Severe	Very severe
Global08r	How would you rate your fatigue on average?	☐ 5	☐ 4	☐ 3	☐ 2	☐ 1

Global07r	How would you rate your pain on average?	☐ 0 No pain	☐ 1	☐ 2	☐ 3	☐ 4	☐ 5	☐ 6	☐ 7	☐ 8	☐ 9	☐ 10 Worst pain imaginable

SLEEP - The following question relates to your usual sleep habits during the past month only. Your answer should indicate the most accurate reply for the majority of days and nights in the past month.

SL1_04. **During the past month**, how many hours of actual sleep did you get on average at night? (This may be different than the number of hours you spent in bed.)

AVERAGE HOURS OF SL1_EEP PER NIGHT ___________

☐ I do not know

☐ I prefer not to answer

MAAS Instructions: Below is a collection of statements about your everyday experience. Using the scale below, please indicate how frequently or infrequently you currently have each experience. Please answer according to what really reflects your experience rather than what you think your experience should be. Please treat each item separately from every other item.

Please indicate the degree to which you agree with each of the following items using the scale below. Simply check your response to each item

	**Almost always**	**Very frequently**	**Somewhat frequently**	**Somewhat infrequently**	**Very infrequently**	**Almost never**
MA1_01. I could be experiencing some emotion and not be conscious of it until some time later.	☐	☐	☐	☐	☐	☐
MA1_02. I break or spill things because of carelessness, not paying attention, or thinking of something else.	☐	☐	☐	☐	☐	☐
MA1_03. I find it difficult to stay focused on what's happening in the present.	☐	☐	☐	☐	☐	☐
MA1_04. I tend to walk quickly to get where I'm going without paying attention to what I experience along the way.	☐	☐	☐	☐	☐	☐
MA1_05. I tend not to notice feelings of physical tension or discomfort until they really grab my attention.	☐	☐	☐	☐	☐	☐
MA1_06. I forget a person's name almost as soon as I've been told it for the first time.	☐	☐	☐	☐	☐	☐

	Almost always	Very frequently	Somewhat frequently	Somewhat infrequently	Very infrequently	Almost Never
MA1_07. It seems I am "running on automatic" without much awareness of what I'm doing.	☐	☐	☐	☐	☐	☐
MA1_08. I rush through activities without being really attentive to them.	☐	☐	☐	☐	☐	☐
MA1_09. I get so focused on the goal I want to achieve that I lose touch with what I am doing right now to get there.	☐	☐	☐	☐	☐	☐
MA1_10. I do jobs or tasks automatically, without being aware of what I'm doing.	☐	☐	☐	☐	☐	☐
MA1_11. I find myself listening to someone with one ear, doing something else at the same time.	☐	☐	☐	☐	☐	☐
MA1_12. I drive places on "automatic pilot" and then wonder why I went there.	☐	☐	☐	☐	☐	☐
MA1_13. I find myself preoccupied with the future or the past.	☐	☐	☐	☐	☐	☐
MA1_14. I find myself doing things without paying attention.	☐	☐	☐	☐	☐	☐
MA1_15. I snack without being aware that I'm eating.	☐	☐	☐	☐	☐	☐

FFMQ - Please rate each of the following statements using the scale provided. Write the number in the blank that best describes *your own opinion* of what is *generally true for you*.

	1 – Never or very rarely true	2 – Rarely true	3 – Sometimes true	4- Often true	5 – Very often or always true
FF1_01. When I'm walking, I deliberately notice the sensations of my body moving.	☐	☐	☐	☐	☐
FF1_02. I'm good at finding words to describe my feelings.	☐	☐	☐	☐	☐
FF1_03. I criticize myself for having irrational or inappropriate emotions.	☐	☐	☐	☐	☐
FF1_04. I perceive my feelings and emotions without having to react to them.	☐	☐	☐	☐	☐
FF1_05. When I do things, my mind wanders off and I'm easily distracted.	☐	☐	☐	☐	☐
FF1_06. When I take a shower or bath, I stay alert to the sensations of water on my body.	☐	☐	☐	☐	☐
FF1_07. I can easily put my beliefs, opinions, and expectations into words.	☐	☐	☐	☐	☐
FF1_08. I don't pay attention to what I'm doing because I'm daydreaming, worrying, or otherwise distracted.	☐	☐	☐	☐	☐
FF1_09. I watch my feelings without getting lost in them.	☐	☐	☐	☐	☐
FF1_10. I tell myself I shouldn't be feeling the way I'm feeling.	☐	☐	☐	☐	☐
FF1_11. I notice how foods and drinks affect my thoughts, bodily sensations, and emotions.	☐	☐	☐	☐	☐
FF1_12. It's hard for me to find the words to describe what I'm thinking.	☐	☐	☐	☐	☐
FF1_13. I am easily distracted.	☐	☐	☐	☐	☐
FF1_14. I believe some of my thoughts are abnormal or bad and I shouldn't think that way.	☐	☐	☐	☐	☐
FF1_15. I pay attention to sensations, such as the wind in my hair or sun on my face.	☐	☐	☐	☐	☐

	1 – Never or very rarely true	2 – Rarely true	3 – Sometimes true	4- Often true	5 – Very often or always true
FF1_16. I have trouble thinking of the right words to express how I feel about things.	☐	☐	☐	☐	☐
FF1_17. I make judgments about whether my thoughts are good or bad.	☐	☐	☐	☐	☐
FF1_18. I find it difficult to stay focused on what's happening in the present.	☐	☐	☐	☐	☐
FF1_19. When I have distressing thoughts or images, I "step back" and am aware of the thought or image without getting taken over by it.	☐	☐	☐	☐	☐
FF1_20. I pay attention to sounds, such as clocks ticking, birds chirping, or cars passing.	☐	☐	☐	☐	☐
FF1_21. In difficult situations, I can pause without immediately reacting.	☐	☐	☐	☐	☐
FF1_22. When I have a sensation in my body, it's difficult for me to describe it because I can't find the right words.	☐	☐	☐	☐	☐
FF1_23. It seems I am "running on automatic" without much aware- ness of what I'm doing.	☐	☐	☐	☐	☐
FF1_24. When I have distressing thoughts or images, I feel calm soon after.	☐	☐	☐	☐	☐
FF1_25. I tell myself that I shouldn't be thinking the way I'm thinking.	☐	☐	☐	☐	☐
FF1_26. I notice the smells and aromas of things.	☐	☐	☐	☐	☐
FF1_27. Even when I'm feeling terribly upset, I can find a way to put it into words.	☐	☐	☐	☐	☐
FF1_28. I rush through activities without being really attentive to them.	☐	☐	☐	☐	☐

	1 – Never or very rarely true	2 – Rarely true	3 – Sometimes true	4- Often true	5 – Very often or always true
FF1_29. When I have distressing thoughts or images, I am able just to notice them without reacting.	☐	☐	☐	☐	☐
FF1_30. I think some of my emotions are bad or inappropriate and I shouldn't feel them.	☐	☐	☐	☐	☐
FF1_31. I notice visual elements in art or nature, such as colors, shapes, textures, or patterns of light and shadow.	☐	☐	☐	☐	☐
FF1_32. My natural tendency is to put my experiences into words.	☐	☐	☐	☐	☐
FF1_33. When I have distressing thoughts or images, I just notice them and let them go.	☐	☐	☐	☐	☐
FF1_34. I do jobs or tasks automatically without being aware of what I'm doing.	☐	☐	☐	☐	☐
FF1_35. When I have distressing thoughts or images, I judge myself as good or bad depending what the thought or image is about.	☐	☐	☐	☐	☐
FF1_36. I pay attention to how my emotions affect my thoughts and behavior.	☐	☐	☐	☐	☐
FF1_37. I can usually describe how I feel at the moment in consider- able detail.	☐	☐	☐	☐	☐
FF1_38. I find myself doing things without paying attention.	☐	☐	☐	☐	☐
FF1_39. I disapprove of myself when I have irrational ideas.	☐	☐	☐	☐	☐

Sustained Attention to Response Task

The Sustained Attention to Response Task (SART) is a computerized test of sustained attention, response inhibition (executive function) and self-regulation. Subjects are instructed to press a key in response to rapidly displayed integers (1-9) and withhold response to a designated "no-go" integer. SART errors consist of summed commission errors (button press on no-go trial) and omission errors (button not pressed on "go" integers). SART performance is associated with prefrontal cortex functioning, has been found to increase with mindfulness training and is correlated with scores on mindfulness questionnaires (specifically, the Mindful Attention Awareness Scale).

MP1_01 Think about the last 12 months. During that time, have you practiced mindfulness, either formally or informally, in any way?

No .... 0 **(*Skip to MP1_03*)**
Yes 1

On average, how minutes PER WEEK do you engage in the following types of mindfulness activities?	Average # Minutes per week	Less than weekly	*Don't Know*	*Prefer not to answer*
MP1_02a Body Scan	____	666	777	888
MP1_02b Yoga	____	666	777	888
MP1_02c Awareness of breath meditation	____	666	777	888
MP1_02d Sitting Meditation	____	666	777	888
MP1_02e Walking Meditation	____	666	777	888
MP1_02f Loving-kindness Meditation	____	666	777	888
MP1_02g Mountain Meditation	____	666	777	888
MP1_02h Visual Meditation	____	666	777	888
MP1_02i Eating Meditation	____	666	777	888
MP1_02j Meditation moving through regions, such as breath, physical sensations, sound, thoughts, and open awareness	____	666	777	888
MP1_02k Goal-related activity (e.g., physical activity, diet change, etc.). *Please describe*_____________________________	____	666	777	888

MP1_02l Other mindful activities:<br>Other 1 *describe:* ________________ ....<br>Other 2 *describe:* ________________ ....<br>Other 3 *describe:* ________________	____	666	777	888

MP1_03 Which of the following statements, BEST describes your current attitude towards mindfulness?

I do not plan on practicing mindfulness 1
I see value in mindfulness but do not practice it regularly...........2
I practice mindfulness regularly..3
Other (*please describe* ____________________________)......4
Don't Know 7
Prefer not to answer..8

MP1_04 Which of the following, if any, have you participated in [since completing the course / the last six months]?

None, I have not practiced mindfulness 00

Check ALL that apply

MB-BP Study Regular 1-1.5 hour Booster Sessions.......[ ]
MB-BP Study All Day Retreats.......................................[ ]
Meditation group *not* related the MB-BP Study ....[ ]
Mindful yoga group *not* related the MB-BP Study ....[ ]
Other 1 *describe:* ________________ ....[ ]
Other 2 *describe:* ________________ ....[ ]
Other 3 *describe:* ________________ ....[ ]
Don't Know..77
Prefer not to answer..88

MP1_05 We are interested in the ways in which mindfulness practice may or may not impact your life. Please describe below your relationship to mindfulness since completing your course.

Don't Know 7
Prefer not to answer..8

Brown University

Research Protections Office
Institutional Review Board
Modification Request

Date of Request: 5/8/18 **Investigator's Name and Title:** Eric Loucks, PhD, Assistant Professor

Study Title: Mindfulness-Based Blood Pressure Reduction (MB-BP) Study (#1412001171)

Original Type of Review: ☐ Exempt ☐ Expedited ☒ Full Board

1) **Provide a brief lay summary of the overall project. Include enough detail to allow the IRB to evaluate the requested change(s) within the context of the overall project.** (Attach summary to this form)

   See attached summary

2) **Provide a detailed description of the changes being requested** (Use additional pages, if necessary):

   See attached summary

3.) **State the reason (justification) for the requested modification.** (Use additional pages, if necessary):

   See attached summary

4.) **What is your assessment of how the changes will affect the overall risk/benefit ratio of the study and the willingness of individuals to participate?**

   See attached summary

5.) **Does the requested modification require new documents or changes to the approved consent form or other documents?**

   ☒ Consent/assent documents (attach revised version with changes highlighted)

   ☒ New/revised instruments (attach - if revised, highlight changes)

   ☐ New/revised advertising materials (attach - if revised, highlight changes)

Do you have a conflict of interest on this project according to Brown's policy? ☐**YES** ☒**NO**

If YES, has this conflict been previously disclosed to the IRB? ☐**YES** ☐**NO**

PI signature: [signature] Date: 5/9/18

1. **Provide a brief lay summary of the overall project. Include enough detail to allow the IRB to evaluate the requested change(s) within the context of the overall project.**

Original lay summary providing broad overview of the project:

The World Health Organization reported that suboptimal blood pressure (BP) is responsible for more than half of cardiovascular disease mortality world-wide. Furthermore, greater than half of those with hypertension have uncontrolled BP. *A 2009 Institute of Medicine report recommended prioritizing research to "Compare the effectiveness of mindfulness-based interventions (e.g. yoga, meditation, deep breathing training) and usual care in treating… cardiovascular risk factors."* Evidence-based mindfulness interventions, including Mindfulness-Based Stress Reduction, may have some effects on blood pressure, where a recent meta-analysis and systematic review of 4 randomized controlled trials demonstrated significant effects, but evidence of heterogeneity in effect sizes. The methodologically highest quality studies had the smallest effect sizes (range 0-5 mmHg). Mindfulness-Based Stress Reduction (MBSR) has been customized to a number of disease processes, such as Mindfulness-Based Cognitive Therapy for patients with recurrent depression, and Mindfulness-Based Relapse Prevention for patients with substance use addictions. Effect sizes have been increased by customizing mindfulness interventions to diseases of interest. The same may be true for hypertension, *however mindfulness interventions customized for prehypertensive/hypertensive patients have never been investigated. Until methodologically rigorous studies to evaluate customized interventions for hypertension are performed, we will not know if the observed preliminary effects of general mindfulness interventions on blood pressure reduction could be much more effective with a tailored approach.* Consequently, we propose to conduct a stage I behavioral therapy intervention study to evaluate whether MBSR customized to prehypertensive and hypertensive patients has the potential to provide clinically relevant reductions in BP. Consequently the specific aims are:

Stage 1a: Therapy Development/Manual Writing

1. To outline and evaluate key novel elements of mindfulness-based hypertension therapy (MBHT), customized from the evidence-based MBSR. *We hypothesize that the most important novel element will be generation of mindfulness skills specifically applied to hypertension risk factors such as diet, physical activity, obesity, alcohol consumption and antihypertensive medication adherence.* This aim will be achieved using (1) focus groups of participants undergoing the MBHT behavioral intervention, (2) discussion with experts (including cardiologists, epidemiologists, mindfulness experts, mindfulness intervention instructors) prior to, and following pilot testing of MBHT in participants, and (3) clinical judgment of the investigators performing the intervention.
2. To determine effectiveness of MBHT on primary outcomes (systolic blood pressure, retention rates, recruitment rates, and adverse effects) and secondary outcomes (hypertension risk factors such as diet, physical activity, obesity, and antihypertensive medication adherence) in hypertension subgroups, specifically participants with (1) prehypertension, (2) controlled hypertension, and (3) uncontrolled hypertension. Initial decisions about the targeted sample based on hypertension status will be made.
3. To develop an MBHT therapist manual and training program, including procedures for training, supervising, and evaluating therapists. Furthermore, acceptable therapist characteristics will be developed. The manual and training program will include themes such as specification of unique and common elements of MBHT *vs.* other interventions, description of interventions excluded from MBHT, and specification of key treatment

parameters such as frequency and duration of treatment, session length, topics addressed, sequence of sessions, as well as therapist adherence and competency measures. The MBHT training will consist of a therapist manual, a formal didactic training seminar, and at least one closely supervised training session.

Stage Ib: Pilot Trial

4. To determine whether a mindfulness-based hypertension therapy (MBHT) intervention, customized from the evidence-based MBSR, has promise to be an effective behavioral therapy for participants with hypertension and/or prehypertension. We will perform a randomized controlled pilot trial for MBHT *vs.* enhanced usual care control. *We hypothesize that MBHT will have adequate recruitment rates (≥10% of prehypertensive/hypertensive participants invited from physicians' offices), fairly low drop out rates (<15%), and medium effect sizes (e.g. 5-10 mmHg systolic BP) for reduction in blood pressure.*

These findings will provide publishable pilot data that will inform future randomized clinical trials that evaluate effects of MBHT on long-term changes in blood pressure *vs.* usual care and active control groups. *If proven effective, MBHT could be offered as a complementary program in the prehypertensive/hypertensive patient population that contributes to over half of the cardiovascular disease mortality world-wide.*

Project update as of May 2018:

The Mindfulness-Based Blood Pressure Reduction (MB-BP) Study, formerly known as Mindfulness-Based Hypertension Therapy (MBHT), is currently in Year 2 of a five year NIH UH2 grant.

With the exception of the two year follow ups, data collection for the single arm trial (Stage 1a) of the project has ended and analysis is on-going. In total we ran three separate Mindfulness-Based Blood Pressure reduction (MB-BP) intervention courses, with a total sample size of 43 eligible participants who both enrolled and completed the 9-week intervention. We had eight eligible participants enroll in the study who later decided to not complete the course.

In June 2017 we began enrollment for the next phase (referenced as Stage 1b above, but now called 'Stage 2a') of the study, which is to conduct a Randomized Controlled Trial (RCT) with *enhanced usual care*. Data collection for this phase will continue through September 2019.

2. Provide a detailed description of the changes being requested (Use additional pages, if necessary):

This amendment covers the following seven requests/revisions which are outlined below:

(1) *Permission to drop the 12 month and two year follow-ups for Stage 2a participants only.* We plan on continuing long term follow up with Stage 1 participants as originally proposed. Stage 2a participants who have already completed their six month follow ups would be notified of the revision and would be sent their remaining participant incentive balance in the form of a check or money order. Participants who have already enrolled in the study but have not yet completed their six month (now final) assessment, would be notified of the revision at the final follow up and would be given the remaining incentive balance at that visit. Newly enrolled participants would follow the new structure of assessments where they would receive $50 USD at each of the two follow ups (10 weeks and 6 months). Note that this revision is reflected in the following attached documents: revised informed consent form (Appendix 1) and revised phone screener (Appendix 2).

(2) *Permission to reduce the number of home blood pressure readings and to drop the mandatory introduction session that is part of the course.* We would like to reduce the number of home blood pressure readings that we ask participants to take from six home readings spread out over two week periods to just three readings spread out over one week periods. The introduction session that we had been using as a 'run-in' period for the intervention is no longer needed and we would like to remove this additional study requirement.

(3) *Approval to assist our collaborators at UMass Medical School with the initial screening and scheduling processes for their fMRI study.* We currently have Brown IRB permission to assist with recruitment of this study. Under the revised protocol, MB-BP participants, who verbally consent to hear more about the fMRI study after listening to our research staff go over the approved one-page fMRI study talking points document, will then be invited to go through the initial pre-screening process (see attached pre-screener found in Appendix 3). Screening for the fMRI study would involve asking enrolled MB-BP participants a brief subset of questions that are designed to assess an individual's eligibility for safely completing a fMRI. In other words, it identifies individuals who may be put at risk for harm if scanned (e.g., pregnant woman, individuals who experience claustrophobia, people with metal implants, etc.). Participants who are identified as potentially eligible through this screening process and who express interest in participating would then be tentatively scheduled for a fMRI up at the UMass facility. This information would then be relayed to the research staff up at UMass Medical School, who would then proceed to follow up with the eligible participants to both confirm the appointment as well as go through the next steps of the screening process and study protocol. It is important to point out that there is an additional screening process that takes place in person prior to the individual going through the fMRI imaging. The fMRI study is approved by the UMass Medical School IRB.

(4) *Permission to conduct the 9-week mindfulness intervention off site in safe, community-based locations.* Currently the 9-week mindfulness intervention only takes place on-site at Brown University. We are seeking permission to be able to offer the class off site in the community in locations that are accessible to the populations we are attempting to enroll and serve. For example, we are partnering with the Lifespan Community Health Institute located on Prairie Avenue in Providence, Rhode Island and have been invited to hold a class in their facility.

(5) *Recruitment partnership at Lifespan Emergency Department* – we are partnering with Dr. Laura McPeake as well as other Lifespan physicians in the Rhode Island Hospital Emergency Department to assist with MB-BP recruitment. Dr. McPeake and colleagues are willing to hand out our Brown IRB approved recruitment material to patients presenting at the RIH ED who potentially would be a good fit for the study (i.e., diagnosed with uncontrolled hypertension). Note that we have spoken with Candy Frater in the RIH Research Protection Office and, per her instruction, will put in a new protocol submission to Lifespan IRB requesting permission to recruit in the RIH ED. We will wait for both Brown IRB and Lifespan IRB approval is received before recruiting in this capacity.

(6) *Permission to conduct qualitative interviews with low SES population to explore perceptions of and openness to mindfulness-based interventions.* The qualitative interviews we are proposing would be conducted in-person in a safe, mutually agreed upon location where privacy is able to be maintained. Interviews are expected to take around 20-30 minutes on average and participants would be given a $10 gift card for participating. All interviews will be conducted by a trained research assistant, Julie Webb, who is a graduate student working in the Mindfulness and Cardiovascular Health Lab. We are hoping to conduct 25-30 qualitative interviews in total, depending on budget, study timeline, and RA availability. Participants will be recruited primarily through word of mouth via already established social networks. Additionally, Ms. Webb will have the opportunity to recruit by attending health fairs and free blood pressure screenings in the Providence community which are being offered by the Lifespan Community Health Institute, who is an existing partner of ours. Individuals who agree to participate will complete the attached bulleted informed consent form. The only eligibility requirements for the qualitative interviews is persons must be 18 years of age or older, able to speak and read in English, and self-identify as low SES. Note that Ms. Webb was originally going to complete these interviews as part of a class project and did not plan on publishing results. She later connected with the MB-BP Study PI, Dr. Eric Loucks, and together they were able to create an opportunity for Ms. Webb's research to be used to help inform and grow the MB-BP study. For this reason, Ms. Webb already has existing data that was collected previous to IRB-approval. In addition to requesting permission to recruit and interview new participants for the qualitative interviews, we are requesting permission for Ms. Webb to go back to individuals who already spoke with her and to seek their informed consent to allow Ms. Webb to use their results as part of the research. Related attachments include: Bulleted Informed Consent Form for qualitative interviews (Appendix 4a); Qualitative Interview questions and talking points – note that these are written to be at an 8th grade reading level or lower (see Appendix 4b); demographic self-report questionnaire to be completed by participants after the qualitative interview (Appendix 4c); and an IRB Form 1 investigator form for Julie Webb as a student investigator working with Dr. Loucks on the MB-BP project (Appendix 4d).

(7) Lastly, we are taking this opportunity to update our existing consent forms to adapt to the new Common Rule requirements. Attached is our MB-BP informed consent form revised to follow the adult template posted online by the Brown University RPO (See Appendix 1).

3. State the reason (justification) for the requested amendment:

(1) We are proposing to drop the 12 month and 2 year follows ups for stage 2a. Exploration of long term effects of the intervention is to be isolated in Stage 1 participant only due shifts in resource allocation and research priorities. Additionally, the 12-month assessment was dropped due to complications in dealing with cross over affects due to controls being invited to participate in the mindfulness intervention after their six month follows up are completed. It is a simpler research design to have the six month follow up be the final point of assessment for all Stage 2a participants enrolled.

(2) We believe that by dropping the number of home blood pressure readings and by removing the introduction session we will reduce participant burden and increase the quality of the data that we are getting. Additionally we have some concern that the home blood pressure readings are in themselves an active component and may be interfering with the study design a bit by making the control group more active than originally intended. By reducing the number of home blood pressure readings we are hoping to make the control group less active.

(3) After conducting one round of fMRI study recruitment we realized it makes more sense for the Brown University research staff to screen and schedule the MB-BP participants for their fMRIs rather than have the UMass staff make a separate phone call to participants. This is for a few reasons. First, the Brown staff have already established rapport with participants. It is easier and more effective to explain the details of the fMRI study in-person and to go through the screening process than it is to try to reconnect with the participant by phone on a separate occasion. We found the participants did not always pick up their phones when UMass called possibly because they were busy and/or because they did not recognize the number. We also found several participants were discovered to be not a good fit for the fMRI study once they learned that it involved being in a tight space for extended period of time (e.g., inducing feelings of claustrophobia). We would have saved the participants the trouble of making time for a separate screening call if we were able to screen them in person at Brown. We don’t anticipate the screener to take too much additional time (i.e., we estimate around 5-10 minutes).

(4) As recruitment efforts ramp up and we seek to enroll a more diverse and generalizable study population, we are making an effort to address some of the known barriers to participation in the mindfulness intervention, such as transportation. We have heard from individuals that it can sometimes be intimidating to travel to a prestigious institution such as Brown and that it would be more appealing to take part in a class located within a familiar community setting. When selecting locations, we will keep safety, accessibility, and confidentiality in mind.

(5) We are continually looking for additional avenues for study recruitment, particularly in locations where we have the opportunity to reach a more diverse racial and SES population. The RIH ED partnership is one such avenue. It also allows us to more effectively target individuals struggling with uncontrolled hypertension (i.e., our target population).

(6) Little is known about the impact and perception of Mindfulness-based interventions in low SES communities. We are partnering with Julie Webb, a graduate student at Brown University and member of our Mindfulness and Cardiovascular Health lab team, to conduct qualitative interviews with low SES individuals to learn more about this topic. Information learned will allow us to more

effectively recruit low SES populations as well as better serve these populations with our existing MB-BP intervention.

(7) Per the Brown University RPO's request, we are updating our existing consent forms to follow the new formatting and content requirements required by Common Rule.

4. What is your assessment of how the changes will affect the overall risk/benefit ratio of the study and the willingness of individuals to participate?

Overall we believe that the changes outline in this amendment will significantly reduce participant burden. Specifically, Stage 2a participants will no longer be asked to complete long-term follow ups (i.e., 12mo and 2yr). We are also reducing the number of home blood pressure readings requested at each time point from six readings down to three readings. The introduction session is being removed from the study protocol.

The revisions also reflect an effort on our part to address participant barriers, such as transportation, and to make the MB-BP intervention overall more accessible to individuals in the local communities.

We do not anticipate any increase risk to study participants.

5. Does the requested amendment require new documents or changes to the approved consent form or other documents?

See enclosed attachments related to this amendment:

Appendix 1 - Revised informed consent form (v.3.0) – using new Standard Consent Template found online

Appendix 2 – Revised phone screener document (v.3.0) – with track changes

Appendix 3 – UMass Medical School fMRI Study Prescreening form to be completed by Brown University MB-BP research staff

Appendix 4 – Materials related to qualitative interviews with low SES individuals

Appendix 4a: Bulleted Informed Consent Form for qualitative interviews

Appendix 4b: Qualitative Interview questions and talking points

Appendix 4c: Demographic self-report questionnaire to be completed by participants after the in-person qualitative interview

Appendix 4d: IRB Form 1 investigator form for Julie Webb

Appendix 1 -
Revised Informed Consent Form
for MB-BP Study Stage 2a (v.3.0)

BROWN

BROWN UNIVERSITY
CONSENT FOR RESEARCH PARTICIPATION

The Mindfulness-Based Blood Pressure Reduction (MB-BP) Study
Version 3.0, May 10, 2018

KEY INFORMATION:
You are invited to take part in a Brown University research study called MB-BP. Your participation is voluntary.

- PURPOSE: In this study, we are looking to see if mindfulness practices improve blood pressure, and if education about hypertension risk factors may also improve blood pressure.
- PROCEDURES: If eligible and selected to receive the intervention, you will be asked to take part in a 9-week mindfulness course where you will free receive training in meditation, mindful movements, and the roles of things like diet, physical activity and medication in reducing blood pressure. As part of the project, you will be asked to participate in health assessments before and after the class.
- TIME INVOLVED: The mindfulness class involves 30 hours of class time and up to 48 hours of at home practice spread out of the course of 9-weeks. The three research assessments are estimated to take around four hours each or around 12.5 hours in total.
- COMPENSATION: You will receive $100 USD in total compensation for your time.
- RISKS: The risks to you in this study are small. They include possible discomfort during research assessments and/or the 9-week intervention. All aspects of the study are voluntary.
- BENEFITS: There are no guaranteed direct benefits to participating in this study. We are investigating whether or not the 9-week intervention actually works to lower blood pressure.
- ALTERNATIVES TO PARTICIPATION: A number of different therapies, including antihypertensive medication, diet changes, physical activity, and reducing excessive alcohol consumption may also be beneficial for reducing blood pressure. Education about these therapies are integrated into this course, but other forms of these alternative therapies are also available in the community.

This remainder of this form will explain in detail the purpose of the study, how the study will be carried out and what you will be expected to do. It will also explain the possible risks and possible benefits of being in the study. If any part of the following description is not clear to you, you are encouraged to contact the researcher to answer any questions before you decide whether to take part in the study. If you decide to participate, please fill out and sign the last page of this form.

1. Researcher(s):
The Principal Investigator on this project is Dr. Eric B. Loucks. He can be reached at 401-863-6283 or by email at eric.loucks@brown.edu. Research staff working on the study can be contacted by phone or email at: 401-400-4768 and mindfulness@brown.edu.

2. What is this study about?
The purpose of the study is to investigate the impact of mindfulness practices and health education on blood pressure. You have been selected for this study because you expressed interest in the project and because you met entrance criteria for having prehypertension or hypertension, or another cardiovascular risk factor that could be influenced by this program. Eligibility for the study is still being assessed. Therefore, it is possible you may not be eligible for the study even after signing this

consent form. Your participation in this study is voluntary and can be withdrawn at any point in the project.

In order to assess the effects of the customized mindfulness intervention, you will be asked to complete some questionnaires and laboratory assessments before and after the intervention. Specifically, assessments will be completed at: baseline, 10 weeks, and 6 months. To express our gratitude for participation, you will be given $50 at each of the follow ups ($100 USD total). As part of the study you will also be given a wireless blood pressure monitor (estimated value of $90) to use throughout the study.

This is a Randomized Control Trial. Participants enrolled into the study will be randomly assigned to one of two groups: (1) the intervention group or (2) the wait-list control group. The wait-list control group will be given the opportunity to participate in the intervention after the six month follow up assessments are completed. Both the intervention and the control group will be asked to participate in the research assessments.

3. What will I be asked to do?

If you agree to participate, you will be asked to consent to the following:

a) Participation in a screening interview in which you will be asked questions about past and present mental health, including depression and suicide (previously completed with your verbal consent).

b) Completion of an in-person screening assessment, during which your blood pressure, height, weight and other basic demographic and health data will be collected and assessed in order to determine eligibility for the study.

c) Completion of questionnaires administered in-person and online that ask about a wide range of topics, including your diet, physical activity, smoking, medication use, personality, emotions, attention and past experiences, including stressful or traumatic experiences. These questions will probe sensitive psychological areas, including physical, emotion and sexual abuse. These questionnaires may take up to 3 hours to complete. By completing the interviews and questionnaires, you are giving the researchers permission to use the information you have provided. You have the right not to answer any of the questions.

d) Directly assessed blood pressure, heart rate, height, weight, physical activity, and antihypertensive (blood pressure) medication use at baseline and after the mindfulness course. Physical activity will be assessed for a week at a time using small actigraphy monitors (i.e., Fitbits) that attach to your wrist. If you take antihypertensive medication, we will provide you with an electronic bottle cap that will automatically record when the pill bottle is opened during the study. This will help us measure how often the medication is used. We will also provide you with a wireless blood pressure monitor and will ask that you take your blood pressure at home systematically during each of the research assessment periods (i.e., baseline, 10 weeks, and 6 months).

e) You will be asked to perform some cognitive tasks. Some of these tasks may involve computer-based tests of attention or decision-making. Together these tests may take as long as 20 minutes.

f) During the in-person assessments you will also be given a battery of stress tests that are designed to induce a stress response so that we can monitor your cardiovascular response and recovery.

g) There will be two follow ups that take place after 10 weeks and 6 months from the start of the intervention. The assessments and questionnaires you will complete for the follow ups will be the same as those completed at baseline.

If randomized into the intervention group…

h) You will be asked to participate in the mindfulness program, which consists of 9 weekly sessions of 2.5 hours each and will include one 7.5 hour weekend retreat. Daily at home practice assignments may take as long as one hour and consist of practicing mindfulness exercises with the aid of a guided meditations and completing worksheets related to stress, thoughts, and common reactions to various types of events. Individuals randomized into the wait-list control group, will be invited to take part in the mindfulness class after the completion of the 6 month follow up assessments.

i) Class sessions may be recorded so we can analyze the quality of the treatment you receive. The recordings will be transcribed so that we may analyze the text. The recordings will be identified by study number, will only be heard by study staff and will be destroyed after transcription.

j) You may be asked to complete a few short questionnaires each week during the 9 week condition.

Table Summarizing Activities and Time Commitment for this Study.

**Activity**	**Estimated Time Commitment**
In-person screening assessment	0.5 hours
Baseline<br>In-person assessment<br>Online questionnaire<br>At home health monitoring (e.g., fitbit, BP, etc.)	 2.5 hours<br>1.0 hours<br>0.5 hours
Intervention*<br>Mindfulness course<br>Home practice assigned during course<br><br>**Intervention group only; control group will be invited to take part in a class post 6 month follow up but it will not be required as part of the study.*	Nine 2.5 hour sessions<br>7.5 hour all day retreat<br>Up to 1hr daily home practice assignments<br>*Total course time: 30.0 hours*<br>*Max. practice time: 48 hours*
Follow Ups – 10 week ($50) and 6 month ($50)<br>In-person assessments<br>Online questionnaires<br>At home health monitoring (e.g., fitbit, BP, etc.)	Total follow up time:<br>5.0 hours<br>2.0 hours<br>1.0 hours
**TOTAL ESTIMATED TIME COMMITMENT**	12.5 hours – CONTROL<br>80.5 hours - INTERVENTION

Your participation in this study may last up to 6 months in duration and is estimated to take up to 80.5 hours in total.

Feedback: At the end of the study, you will receive individual feedback about the changes that occurred since the first assessment. Specifically, you will receive an individualized handout listing % change (increase or decrease) on scales of attention, stress, mood, health behaviors, weight, and blood pressure across the study.

Uncontrolled Hypertension: If during the in-person assessments it appears that you have stage 2 uncontrolled hypertension (your average systolic blood pressure reading is 140 mmHg or greater and/or your average diastolic blood pressure is 90 mmHg or greater) AND you indicate to us that you are not currently being treated for hypertension, then we will be requesting your permission to contact your health care provider to notify him/her of the blood pressure results. If you do not have a health care provider and/or do not have health insurance, our staff will provide you with resources to help you search for one; although we cannot guarantee that we will be able to find you one nor that it will be free. It is your choice on whether or not you would like us to follow up with your health care provider. Your participation in the study is not contingent on this communication; however, it is our recommendation that all individuals with uncontrolled hypertension be under the care of a health care professional.

4. **Will I be paid?**

You will be given $50 at each of the two follow up assessments ($100 USD total).

5. **What are the risks?**

The risks to you in this study are small. The questionnaires used in the study are routine, standardized forms for epidemiologic research. Certain questions may be upsetting as they may probe sensitive psychological areas and inquire about upsetting or traumatic events, including physical, sexual or emotional abuse and/or current psychiatric symptoms. The cognitive tests and stress battery may also invoke a stress response that may be uncomfortable. All aspects of the study are voluntary; you have the right to skip anything during the study that makes you uncomfortable.

Meditation-based interventions may results in discomfort with attention to unpleasant thoughts, feelings or body sensations. Some individuals may experience an initial increase in undesirable feelings with increased attention to them.

It is possible that injuries could be sustained during the study either from the gentle mindful movements (i.e., yoga), or from physical activities that participants engage in as a way to reduce blood pressure. To help limit this, you will receive a handout showing the yoga poses that will be offered during the course that you can show your health care provider so that they can advise on which poses to do, and which to avoid. Modifications of poses will be available as needed. None of the poses (or the yoga as a whole) are mandatory to be done. You will also be encouraged to explore physical activities that promote strength and conditioning as a way to reduce blood pressure. You will be encouraged to not go beyond any physical limits of your body, and will be encouraged to ask your healthcare provider about advised physical activities and mindful movements if you have any physical limitations.

While physical and mental injury is always a possibility the potential for harm is limited. Note that a research injury is any physical or mental injury or illness caused by your participation in the study. If you are injured by a medical treatment or procedure that you would have received even if you were not in the study, that is not a research injury. To help avoid research injury and potential added medical expenses, it is important to follow all study directions carefully. If you are covered by insurance and suffer a research injury, it is possible that some or all of the costs of treating your condition could appropriately be billed to your insurance company. If such costs are not covered your health insurance company, it is possible you would have to pay for these costs out of pocket. Brown

University's policies do not cover payment for such things as lost wages, medical care expenses, or pain and suffering.

Precautions should be taken to avoid injuries. If you do become injured during the study, you should call your doctor immediately. You should also alert the study staff that you have been injured. Heart attack and sudden death related to heart problems have been known to occur in people while they are exercising. This is very rare, however. Estimates of sudden cardiac death range from 0 to 2 per 100,000 hours. However, the researchers cannot guarantee that no complications will happen to you.

6. What are the benefits?

We cannot and do not guarantee or promise that you will receive any direct benefits from this study. However, participation in the study creates the potential benefit of a) identifying effective treatments for elevated blood pressure, b) gaining knowledge of the effects of mindfulness practices, c) receiving information about your psychological and physical functioning. As part of the study, you will receive a wireless blood pressure monitor that will be yours to keep. This monitor may provide additional opportunity to monitor your blood pressure at home, which may benefit your health by providing additional biofeedback.

7. How will my information be protected?

Your responses for this study will be kept confidential. All data that we collect will be linked to a study ID# instead of your name. All questionnaires in this study will be filled out through an online survey or a paper version of the survey if you prefer. All of these questionnaires will be linked solely to your study ID#, so that your identity is protected and your answers are confidential. All paper forms and data collection tools will be kept in a locked filing cabinet in a secure location. Study consent forms, including this one, will be kept in a locked filing cabinet separate from the research data. Note that although these measures have been taken to protect your personal information, complete confidentiality cannot be guaranteed when transmitting information over the internet.

While your confidentiality is protected to the extent of the law, there are limitations to confidentiality. If your questionnaire responses indicate that you pose a serious danger to yourself or to another person, then a collaborator (Dr. Ellen Flynn) who is a licensed psychiatrist, may contact you to discuss your responses and possible referral to a treatment provider. Questionnaire items that may warrant follow-up include endorsements of statements about hurting yourself, any high scores in depression, anxiety, or other clinically significant problems. You should also know that there are times when the law might require the release of your responses without your permission. For example, State law requires researchers to report abuse or neglect of children to the Department of Children, Youth and Families (DCYF). State law also requires researchers to report abuse or neglect of people age 60 and older to the Division of Elderly Affairs.

The findings of the study may be used for medical publication. Your name will not be used in any published reports about this study. Results will be reported in a summarized manner in such a way that you cannot be identified. All personally identifiable information will be "de-identified" and only a unique code number will be used. Study records will be identified with a unique code number and initials. All study records and specimens will be stored in a secure storage area.

Keeping study records: The Principal Investigator for this study will keep your research records indefinitely for research purposes.

Certificate of Confidentiality: This research is covered by a Certificate of Confidentiality from the National Institutes of Health. The researchers with this Certificate may not disclose or use information, documents, or biospecimens that may identify you in any federal, state, or local civil, criminal, administrative, legislative, or other action, suit, or proceeding, or be used as evidence, for example, if there is a court subpoena, unless you have consented for this use. Information, documents, or biospecimens protected by this Certificate cannot be disclosed to anyone else who is not connected with the research except, if there is a federal, state, or local law that requires disclosure (such as to report child abuse or communicable diseases but not for federal, state, or local civil, criminal, administrative, legislative, or other proceedings, see below); if you have consented to the disclosure, including for your medical treatment; or if it is used for other scientific research, as allowed by federal regulations protecting research subjects.

The Certificate cannot be used to refuse a request for information from personnel of the United States federal or state government agency sponsoring the project that is needed for auditing or program evaluation by the National Center for Complementary and Integrative Health, which is funding this project. You should understand that a Certificate of Confidentiality does not prevent you from voluntarily releasing information about yourself or your involvement in this research. If you want your research information released to an insurer, medical care provider, or any other person not connected with the research, you must provide consent to allow the researchers to release it. The Certificate of Confidentiality will not be used to prevent disclosure as required by federal, state, or local law of situations of child abuse and neglect, or harm to self or others.

Finally, Brown University staff sometimes review studies like this one to make sure they are being done safely and correctly. If a review of this study takes place, your records may be examined. The reviewers will protect your confidentiality.

8. Are there any alternatives to this study?

A number of different therapies, including antihypertensive medication, diet changes, physical activity, and reducing excessive alcohol consumption may also be beneficial for reducing blood pressure. Education about these therapies are integrated into this course, but other forms of these alternative therapies are also available in the community.

9. What if I want to stop?

You do not have to be in this study if you do not want to be. Even if you decide to be in this study, you can change your mind and stop at any time. If you refuse to participate in or leave the study, your current or future relationship with Brown University as well as with your physician will; not be affected. If you decide not to participate, or if you quit the study, we will provide you with referrals for alternative treatments, if desired.

10. Who can I talk to if I have questions about this study?

If you have any questions about your participation in this study, you can call the Senior Project Coordinator, Frances Saadeh, at 401-400-4768 or email at mindfulness@brown.ledu. You may also contact the Principal Investigator at any time: Dr. Eric B. Loucks, email: eric.loucks@brown.edu, telephone (401) 863-6283.

A description of this clinical trial will be available on http://www.Clinical Trials.gov, as required by U.S. Law. This Web site will not include information that can identify you. At most, the Web site will include a summary of the results. You can search this Web site at any time.

11. Who can I talk to if I have questions about my rights as a participant?

If you have questions about your rights as a research participant, you can contact Brown University's Human Research Protection Program at 401-863-3050 or email them at IRB@Brown.edu.

12. Consent to Participate

Your signature below shows that you have read and understood the information in this document, and that you agree to volunteer as a research participant for this study.

You will be offered a copy of this form.

Participant's Signature and Date / PRINTED NAME

Research Staff Signature and Date / PRINTED NAME

Appendix 2 -

Revised Phone Screener for MB-BP

v.3.0 – track changes used to show revisions

PHONE-DELIVERED SCREENING QUESTIONNAIRE

PHONE SCREENING QUESTIONNAIRE - PART 1 of 2

The script is shown below in ***bold italics.***

PID. Participant ID: ___________

SQ01. Staff ID: ______________

SQ02. Today's Date (MMDDYY): ________________

SQ03. Current Time (24 hour time, e.g. 14:45): ________

Please now call the participant.

SQ04. Was the participant reached?   YES   NO

Hello, my name is ____________. I am calling from the Brown University School of Public Health because (name of participant) expressed interest in participating in our mindfulness blood pressure study. Is he/she available to talk at this time?

I would like to do a 10-15 minute phone interview with you to determine if you are a good match for this particular study.

SQ05. ***Is now a good time to speak?***

[If yes, proceed to SQ05 script below]

If no… ***When would be a good time to talk?***

SQ06. Day to call back (DDMMYY) ____________

SQ07. Time to call back ______ AM/PM

OK, great. I'm going to give you a quick overview of the study first and can then move on to the phone screener to see if you may qualify for the next part of the study. Feel free to interrupt me at any point if you have a question. Okay?

STUDY OVERVIEW: It is a research study funded by the National Institute of Health and In this study, we are looking to see if mindfulness practices improve blood pressure, and if education about hypertension risk factors may also improve blood pressure. If you are eligible and are randomly selected to take part in the intervention, we will provide you with training in meditation, mindful movements, and the roles of things like diet, physical activity and medication in reducing blood pressure.

You will be taught by a very experienced teacher who is an expert in these fields. The course is free and will take place over a 9-week period, where you come to a class once each week for 2.5 hours each time. There is also a one-day weekend retreat that will be 7.5 hours long. [Go over days / times of course and retreat]

As part of the project, we will also ask you to participate in health assessments before and after the intervention. Health assessments include measures such as blood pressure, height and weight, and questionnaires about your health and experiences. These assessments will take place at ~~four~~ three points throughout the study. At baseline, 10 weeks, and 6 months ~~and 12 months~~.

It is important to note that this is a Randomized Controlled Trial, which means that, if eligible, you will be randomly assigned to one of two groups. The intervention group will be offered the 9 week mindfulness course right away, while the wait-list control group will be invited to take the course after the six month follow up assessment is completed. You will be notified of the randomization results 4-7 days prior to the start of class.

As an expression of gratitude for your time and effort, you will be given $50 USD ~~cash incentives~~ at each of the ~~three~~ two follow up assessments, so $100 in total. ~~You will be given $25 at the first two follow ups and then $50 at the final 12 month follow up.~~ You will also receive a state-of-the-art wireless blood pressure monitor that will be yours to keep. You will be asked to use this monitor to take your blood pressure at home throughout the study.

Do you have any questions about the mindfulness intervention or the research study as a whole? [*Answer questions*****]***

SQ08. ***Does this study sound like something you would be interested in doing? Yes No***

[If yes, proceed to next statement below. If no, politely thank the participant for considering being in this study, and end the call].

Great. There are a few things that I would like to go over before we start the interview. First, some of the questions that I will ask now to figure out if you are eligible to be in this study will be of personal nature, including asking about your mental health and life's experiences.

SQ09. ***Are you in a private place to talk?***

[If yes, proceed to text below. If no, reschedule meeting using variables SQ06 and SQ07 above].

Because this interview is of a personal nature, it is important that you understand that everything you say will be kept strictly confidential. No one outside of our project will ever be able to see your answers, and we will not keep your name in the same place as any of your answers. If you are not eligible after the phone screen, we will destroy your information. If you like, though, we can keep your information on file for future studies.

Ok, to begin, I am going to start by recording your name and contact information.

SQ10. Participant's First Name: ______________________________________

SQ11. Participant's Last Name:____

Participant's Address (in case we need to send any study materials to you):
SQ12a. Street address: _____________
SQ12b. City: _____________________
SQ12c: State: ________
SQ12d: Zip Code: ________________

SQ13a. Participant's Phone number #1 (in case we need to contact you by phone)
______________________________________ Type of Phone: work / home / cell / other

SQ13b. Participant's Phone number #2
______________________________________ Type of Phone: work / home / cell / other

SQ14. Participant's email address (or mailing address if no email):_______________________

SQ15. Notes from interviewer related to participants' contact information (if any):
____
____
____

PHONE SCREENING QUESTIONNAIRE - PART 2 of 2

PID. Participant ID #: ______________

SQ.recruit How did you find out about the study? *Select one.*

From a friend / family member / coworker .... [ ]
From a former or current participant.. [ ]
From PCP or other health care provider....................................... [ ]
Facebook.. [ ]
Twitter... [ ]
Craigslist.. [ ]
Saw an orange flier / card (note location __________)................ [ ]
Other (please describe ____________________________)......... [ ]

INCLUSION CRITERIA: All answers in 3rd column must be YES. If an answer is NO, immediately proceed to question SQ40.			
			Comments
SQ26.	***What is your age?*** [Is age at least 18 years?]	YES NO	
SQ27.	***Can you read and write in English?***	YES NO	

EXCLUSION CRITERIA: All answers in 3rd column must be NO with the exception of SQ32a and SQ33a. If an answer (other than SQ32a and SQ33a) is YES, then immediately proceed to question SQ40.			
			Comments
I will now start to ask some questions about your mental health.			
SQ28.	***Has anyone ever told you that you have bipolar disorder or manic depression?***	**YES NO**	
SQ29.	***Has anyone ever used the word "Borderline" to describe you?***	**YES NO**	
SQ30a.<br><br>SQ30b.	***Have you ever had a hallucination or seen things that other people can't see, or hear things other people can't hear?***<br><br>***Have you ever been diagnosed with schizophrenia or psychosis?***	**YES NO**<br><br>**YES NO**	
 SQ31_1<br>SQ31_2<br>SQ31_3<br>SQ31_4<br>SQ31_5<br>SQ31_6<br>SQ31_7<br>SQ31_8	***Have you ever taken any of the following medications that I am about to read to you?***<br>***Lithium***<br>***Seroquel (quetiapine)***<br>***Abilify (aripiprazole)***<br>***Zyprexa (olanzapine)***<br>***Clozaril (clozapine)***<br>***Haldol/Haloperidol***<br>***Geodon (ziprasidone)***<br>***Risperdal (risperidone)***	 **YES NO DK**<br>**YES NO DK**<br>**YES NO DK**<br>**YES NO DK**<br>**YES NO DK**<br>**YES NO DK**<br>**YES NO DK**<br>**YES NO DK**	
SQ32a	***Have you ever had a suicide attempt?***	**YES NO**	

SQ32b<br><br>SQ32c<br><br><br><br>SQ32d	***[If yes, ask…] Have you considered killing yourself during the past month?***<br><br>***[If yes, ask…] Are you currently suicidal?***<br><br>***[If yes, keep participant on the phone, and follow suicide safety plan below]***<br><br>***[If no, ask…] Are you getting any help for that? If not, then provide list of resources from Safety Plan including Gateway, Anchor and The Providence Center] Not urgent, but inform Dr. Flynn about what was discussed.***	**YES NO**<br><br>**YES NO**<br><br>**YES NO**	

EXCLUSION CRITERIA: All answers in 3rd column must be NO with the exception of SQ32a and SQ33a. If an answer (other than SQ32a and SQ33a) is YES, then immediately proceed to question SQ40.

SQ33a<br><br>SQ33b<br><br>SQ33c	***Would you say you have a trauma history? [If yes…]***<br>***In the past month, have you had any problems with dissociation (memory loss)?***<br>***In the past month, have you had any flashbacks (i.e. sudden and disturbing vivid memory) about the trauma?***	**YES NO**<br><br>**YES NO**<br><br>**YES NO**	
SQ34a<br><br>SQ34b	***In the past month, have you had any problems with obsessions or compulsions, such as washing your hands or checking the oven over and over again?***<br>***[If yes…] Has anyone diagnosed you with obsessive compulsive disorder?***	**YES NO**<br><br>**YES NO**	
SQ35	***In the past month, have you had a panic attack (i.e. sweating, heart palpitations, nausea, trouble breathing, fear of dying/choking/going crazy)?***	**YES NO**	
SQ36	***Have you had any problems with alcoholism or drug use in the past year?***	**YES NO**	
SQ37	***In the past year, have you had an eating disorder, such as starving, binge eating, or vomiting?***	**YES NO**	
SQ38a<br><br>SQ38b	***Do you currently have a mindfulness practice, such as meditation or yoga?***<br><br>***If Yes –***<br>***Please tell me more about your mindfulness practice, including how often you practice per week.***	**YES NO**<br><br>**Fill in response in comments section to the right**	

SQ38c	***Do you currently practice meditation more than once per week? (yoga does not count as meditation in this context)***	**YES NO**	
SQ39	***This class will take place at Brown University in-person. Do you have any medical or mobility issues that would affect you being able to attend class?***	**YES NO**	
SQ40	Participant qualifies for next step of study (next step is 1st blood pressure screening)	**YES NO**<br><br>**If YES go to SQ41.**<br><br>**If NO, go to SQ42.**	

SQ41a. ***Thank you for taking the time to answer these questions. You qualify for the next stage of screening, which is to take your blood pressure at our office. If you're still interested, we'd like to schedule a time to have you come in to complete the in-person screener. It will only take 30 minutes or less and will involve taking your blood pressure 3-5 times, as well as measuring your height and weight and asking a few questions. Is there a day or time that works best for you?*** *[Schedule the In-Person Screening visit now]*

SQ41b. ***INTENTIONALLY REMOVED***

Thank you for your time and interest in this study. We look forward to meeting you in person on [DATE / TIME]. Do you have any questions before we end this call?

IF INELIGIBLE:

SQ42a***. Thank you for taking the time to answer these questions. According to the survey, you do not qualify for the study at this time. There may be other studies you quality for.***

SQ42b. ***Would you like me to keep your information to pass on to these studies?*** YES / NO

SQ42c. [If yes…] ***OK, thank you. We will keep this information for future studies you may qualify for. Thank you for taking the time to talk with me today. Can I answer any questions before hanging up?***

SQ42d. [If no…] ***OK, our copy of this information will be destroyed. Thank you for taking the time to talk with me today. Can I answer any questions before hanging up?***

SAFETY PLAN – PHONE SCREENER ONLY

Enter details below on paper during screening (These variables should are also be entered in the survey via questions SQ10-SQ13). Destroy this paper after screening is complete.

Participant's First Name: ___________________________________

Participant's Last Name:_____________________________________

Participant's Address:

Street address: ____________

City: ___________________

State: _______

Zip Code: _______________

Participant's Phone number #1: ___________________________________

Participant's Phone number #2___________________________________

During the phone-based screening, if participants respond yes to "Are you currently suicidal?", the interviewer should perform the following 2 steps:

1. **Immediately have 911 and Dr. Ellen Flynn called by a colleague who has been informed beforehand that this is a possibility.**

Specifically, while keeping the participant on the phone, show the text below in the box to a colleague.

I have a study participant on the phone who is currently suicidal. Please call 911 immediately, and tell them:<br><br>***"I am calling on behalf of [my name] who is performing a research study at Brown University. He has a participant on the phone who says they are currently suicidal." Please provide the participants' contact information to the 911 operator (i.e. name, address, phone #, email address) as requested. This information is shown above.***<br><br>***Finally, after calling 911, please call Dr. Ellen Flynn, who is the psychiatrist supporting this study. Her cell phone # is 401-258-9829. Please provide her with the same information as was done during the 911 call.***

2. **While speaking calmly with the participant, let them know what you are doing. Specifically, let them know we are calling our study's psychiatrist Dr. Flynn and 911, and why you are doing that (i.e. because we are concerned about you). You can speak with participant to keep him/her on the phone, but the discussion should not be clinical in nature.**

Examples of questions that could be asked in order to keep them on the phone:

- "Tell me what is going on."
- "What's happening right now?"
- Tell me more about why you are interesting in being part of this study.
- What are you hoping to get out of this study?

The following information can be provided to study participants if they state they have had considered killing themselves in the past month. If they are currently suicidal, the main priority is to keep them on the phone while 911 and Dr. Flynn are being contacted.

National Suicide Prevention Lifeline: 1-800-273-8255

Other options are to:

- Call your doctor's office
- Call 911 for emergency services
- Go to the nearest hospital emergency room.

Local Non-Urgent Free or Inexpensive Mental Health Services:
Gateway Healthcare: 401-729-8701
Anchor Counseling Center: 401-475-9979
The Providence Center: 401-276-4020

Appendix 3 -
UMass Medical School fMRI Study
Prescreening Form

To be completed by Brown University MB-BP research staff in-person with MB-BP participants.

UMass Medical School Mindful Self-Regulation for blood pressure MRI study – PRESCREENER to be administered by Brown University MB-BP Research Staff

Date: ________________ Time: _________ Interviewer: ____________________________

PID # ___________________________

In order to determine if you are a good match for this particular study, I will be asking you some questions about your health and history which will take around 3-5 minutes to complete. This is a pre-screening process to determine if you might be eligible for the MRI study conducted at UMass Medical School. This questionnaire is optional and even if you are determined to be eligible for the study, you do not have to agree to take part.

Before I begin with the screening process, I want to let you know that some of the questions I am going to ask you to see if you may be eligible are of a personal nature. For this reason, it is important that you understand that everything you say will be kept strictly confidential. No one outside of our project will ever be able to see your answers, and we will not keep your name in the same place as any of your answers. You may stop or ask questions at any time. If you don't understand what I am asking, please stop me and ask me to explain. If you are eligible for the study and agree to be contacted by the UMass Medical School research staff, we will pass along your name and contact information to their group for follow up.

**INCLUSION CRITERIA: Need YES to both Yes/No questions**		
1. Are you at least 18 years of age?	Yes ___	No ___
2. Are you currently enrolled in the Brown University Mindfulness-Based Blood Pressure Reduction Study?	Yes ___	No ___

**EXCLUSION CRITERIA: To be eligible must answer NO to all Yes/No questions below**		
16. Do you experience claustrophobia?	Yes: ___	No: ___
18. [If female ask:] Are you pregnant?	Yes: ___	No: ___
19. Do you have a serious psychiatric, cognitive or medical disorder which could interfere with completion of the study?	Yes: ___	No: ___

MRI and Safety		
21. Do you have any metal in/on your body that cannot be removed? E.g.<br>☐ surgical implants<br>☐ pins<br>☐ pacemakers<br>☐ prosthetic heart valve<br>☐ aneurism clips or other vascular stent, filters, or other devices<br>☐ surgical clips<br>☐ staples<br>☐ neuro-stimulator devices<br>☐ implanted infusion pumps<br>☐ cochlear (ear) implants<br>☐ Ocular (eye) implants or metal fragments in eyes<br>☐ other prostheses<br>☐ other: _________ Describe: ____________________________	Yes: ___	No: ___
22. Do you have braces or any other metal which may be potentially magnetic?<br>a. This includes braces, false teeth, retainer, special hair dye, etc.	Yes: ___	No: ___
23. Do you have any tattoos?<br>a. If yes, is there any chance that it/they contain metallic pigments?	Yes: ___	No: ___
24. Have you ever been exposed to shrapnel or metal filings (e.g. employed as a machinist, metal worker, welder, or other?	Yes: ___	No: ___
25. Is there any chance that you have ever been hit with any stray metal (BB's, metal shavings, etc)	Yes: ___	No: ___
26. Any condition known to be incompatible with MRI scan	Yes: ___	No: ___
27. (If ppt ever had an MRI or other scan, ask:) Were there any difficulties?	Yes: ___	No: ___
28. Are you over 300 lbs? (weight______)	Yes: ___	No: ___

If the candidate is NOT eligible for the current study:
Unfortunately, it appears that you are not eligible for the fMRI study being conducted at UMass Medical School. END SCREENER

If the candidate appears to be eligible for the current study, and is interested:
So it appears that you did pass the first phase of screening for the fMRI study being conducted at UMass Medical School. The next step would be to have one of their research staff members contact you by phone to complete the screening process and to tell you more about the study. If you are interested in this, I can pass along your information to them. I can also tentatively schedule an appointment for the baseline fMRI imaging?

CHECK ONE:
□ INELIGIBLE □ NOT INTERESTED □ PASSED PRESCREEN, INTERESTED

If the candidate appears to be eligible for the current study, and is interested:

Pre-Screen		
**Mindful Self-Regulation for blood pressure MRI study**		
**Name:**		
**Email:**		**Screening Date:**<br>___ ___ / ___ ___ / ___ ___ ___ ___<br>d d m m y y y y
**Phone 1**	**Phone 2**	
Ok to leave a message?<br>Yes____ No____	Ok to leave a message?<br>Yes____ No____	

Preferred method of contact? Phone/email (circle one)
Scheduling considerations:

TENTATIVE BASELINE fMRI Imaging Appointment:

____ (Date and Time)

Appendix 4a: Bulleted Informed Consent Form for qualitative interviews

BROWN UNIVERSITY

CONSENT FOR RESEARCH PARTICIPATION

Exploring perceptions of and experiences with mindfulness among low-SES populations: Qualitative Interviews for the Mindfulness-Based Blood Pressure Reduction (MB-BP) Study

Version 1.0, May 13, 2018

You are invited to take part in a Brown University research study. Your participation is voluntary.

- RESEARCHER: The lead researcher on this specific project is Julie Webb. She can be reached by phone at 314-403-3054 or email Julie_Webb@brown.edu. The Principal Investigator on the overall MB-BP study is Dr. Eric B. Loucks: eric.loucks@brown.edu.

- PURPOSE: The purpose of this research study is to talk with individuals who self-identify as being from a low socio-economic status and, by doing so, learn more about their perceptions of and experiences with mindfulness. You are being asked to be in this study because you indicated that you are of low-SES and that you might be willing to share your experiences with the researcher, Julie Webb.

- PROCEDURES: You will be asked to meet one-on-one with the researcher, Julie Webb, for a brief interview about your perceptions of and experiences with mindfulness. You do not have to have any previous experiences with mindfulness activities nor do you have to answer every question asked. There are no right or wrong answers. Rather we are looking for your honest and individualized feedback.

  With your permission we will be recording the interview so that we can better and more accurately capture your responses. The recording will only be used by the research staff to transcribe and analyze the results. Your name will not be used in any report or publication. Your answers along with the responses of other individuals interviewed will be combined in order to look for common themes and experiences.

  At the end of the interview, the researcher will ask you to complete a brief questionnaire that asks you some basic questions about yourself.

- TIME INVOLVED: The study will take 20-30 minutes of your time.

- COMPENSATION: You will receive a $10 gift card for your time.

- RISKS: The risks to you in this study are small. You may experience discomfort answering questions, but you have the right to not answer any question you do not wish to and you can end the interview at any time.

- BENEFITS: You may not directly benefit from being in this research study. In talking with the researcher you may learn about mindfulness and the related-research in the Rhode Island area

that is ongoing. Your participation may also help better tailor mindfulness programs to low-SES populations.

- CONFIDENTIALITY: Your responses for this study will be kept confidential to the extent that we are able. All data will be linked to a study ID# instead of your name. This consent form along with any paper forms used will be kept in a locked filing cabinet in a secure location. Although these measures have been taken to protect your personal information, complete confidentiality cannot be guaranteed when transmitting information over the internet.

  While your confidentiality is protected to the extent of the law, there are limitations to confidentiality. If your questionnaire responses indicate that you pose a serious danger to yourself or to another person, then a collaborator (Dr. Ellen Flynn) who is a licensed psychiatrist, may contact you to discuss your responses and possible referral to a treatment provider. You should also know that there are times when the law might require the release of your responses without your permission. For example, State law requires researchers to report abuse or neglect of children to the Department of Children, Youth and Families (DCYF). State law also requires researchers to report abuse or neglect of people age 60 and older to the Division of Elderly Affairs.

  The findings of the study may be used for medical publication. Your name will not be used in any published reports about this study. Results will be reported in a summarized manner in such a way that you cannot be identified. All personally identifiable information will be "deidentified" and only a unique code number will be used. Brown University staff sometimes review studies like this one to make sure they are being done safely and correctly. If a review of this study takes place, your records may be examined. The reviewers will protect your confidentiality.

- VOLUNTARY: You do not have to be in this study if you do not want to be. Even if you decide to be in this study, you can change your mind and stop at any time.

- CONTACT INFORMATION: If you have any questions about your participation in this study, you can call me at 314-403-3054 or email me at Julie_Webb@brown.edu or you can contact my advisor Dr. Eric B. Loucks at eric.loucks@brown.edu.

- YOUR RIGHTS: If you have questions about your rights as a research participant, you can contact Brown University's Human Research Protection Program at 401-863-3050 or email them at IRB@Brown.edu.

Your signature below shows that you have read and understood the information in this document, and that you agree to volunteer as a research participant for this study.

You will be offered a copy of this form.

Participant's Signature and Date / PRINTED NAME

Appendix 4b: Qualitative Interview questions and talking points

Perceptions of and experiences with mindfulness: qualitative interviews with low-SES populations

Semi-Structured Script and Talking Points

Begin by going through the informed consent process using the IRB approved bulleted consent form. Make sure to answer participant's questions and to provide them with a hard copy of the informed consent form.

TALKING POINTS –

- **Confidentiality**: As a reminder, everything you say will remain confidential. No one outside of our project will be able to see your answers and your name and other identifying information will not be used in any reports or publications.

- **Why and How?** We are hoping to get your feedback about your experiences with and perceptions of mindfulness. Please feel free to share your point of view and know that we welcome both positive and negative feedback. There are no right or wrong answers.

- **Suggestions:** With your permission we would like to record this conversation so that it can be transcribed and analyzed. If you would prefer not to be recorded, please let me know. Also, please understand that any report we write regarding your feedback and what we hear from you today will not be identified with you in any way and remains anonymous.

  Suggestions to help us have a good discussion
    - Speak up
    - Audio recording if that is ok
    - Any report that we write about what we hear today will not be associated with your identity
    - There are no right or wrong answers.
    - Feel free to ask questions at any time. There are no bad, or stupid questions.

- **What to expect:** Before we begin I just want to let you know that we will be asking you a series of open ended questions. My role is listen and ask questions to better understand your experiences. So I might ask you for clarification or more detail about a comment but your thoughts are the important part of this discussion. However, in the interest of getting to all the questions I may need to move us along pleas know that this is only due to time considerations.

    - My role is to listen ask questions that help to understand your experience
    - In the interest of time, I may move to the next question to get your thoughts regarding the whole program

Any questions before we begin? Do you consent to having the conversation recorded?

As part of the research study we are interested in understanding your thoughts, feelings, and previous experiences with mindfulness.

1. When you hear the word 'mindfulness' what do you think of?

Mindfulness can be described in different ways, but it is often considered to be "paying attention, on purpose, in the present moment, non-judgmentally".

2. Keeping this definition in mind, on a scale of 1-10, where 1 is not interested at all, and 10 is extremely interested (show participant a Likert scale from 1-10), how interested are you in learning more about mindfulness?

   Probes: *Please share with me why you feel this amount of interest in learning about mindfulness.*

As part of this project, we are trying to learn more about what kinds of people are interested in learning about mindfulness, and if you are interested, better understanding how mindfulness training might be best delivered to you.

At this time I want to get your thoughts on a few different mindfulness programs that are currently being offered. There are quite a few different kinds of mindfulness programs these days. One is a 9 week course that includes a 2.5-hour session once each week and an all-day mindfulness retreat. It trains people how to meditate, and how to apply mindfulness skills to health conditions and stressors in their day-to-day lives. It takes place in a group setting.

3. On a scale of 1-10, where 1 is not interested at all, and 10 is extremely interested (show participant a likert scale from 1-10), how interested are you in taking this kind of a mindfulness course?

   Probe: *Please share with me why you feel this amount of interest in taking this mindfulness program.*

   Probe: What appeals to you? What might hinder your participation? Does the length of the course impact your decision to participate? Is so, what would be an ideal length for you?

Another way people often learn about mindfulness is online for a similar amount of time. For example, on a computer online with a live teacher interacting with you and other students.

4. On a scale of 1-10, where 1 is not interested at all, and 10 is extremely interested (show participant a Likert scale from 1-10), how interested are you in taking an online mindfulness course?

   Probe: *Please share with me why you feel this amount of interest in taking this mindfulness program.*

   Probe: *What makes learning mindfulness online more or less interesting compared to learning in-person?*

One more way people can learn about mindfulness is via an app on their phone. The apps often deliver brief mindfulness lessons or guided meditations, but usually don't have much if any interaction with a live person.

5. On a scale of 1-10, where 1 is not interested at all, and 10 is extremely interested (show participant a likert scale from 1-10), how interested are you in taking a mindfulness course via a phone app?

   Probe: *Please share with me why you feel this amount of interest in taking this mindfulness program.*

   Probe: *What makes learning mindfulness by a phone app more or less interesting compared to learning in-person?*

A final way people can learn about mindfulness is via what we call a “flipped classroom” where they may learn some of the more straight-forward lessons using an app on their phone, or computure. Then for a smaller amount of time, they come to a classroom and learn from a teach, often in a group, and get indivudalized feedback on their experience.

6. On a scale of 1-10, where 1 is not interested at all, and 10 is extremely interested (show participant a likert scale from 1-10), how interested are you in taking a mindfulness course via this kind of “flipped classroom” approach?

   Probe: *Please share with me why you feel this amount of interest in taking this mindfulness program.*

   Probe: *What makes learning mindfulness by a flipped classroom approach more or less interesting compared to learning in-person, online or via an app?*

The last questions we have are about the course structure and instructor.

1. How important is it for you to have an instructor of your own race or ethnicity if you were to participate in a mindfulness-based intervention?

Probe: *Why?*

2. How important is it for you to have an instructor speaking your own first language if you were to participant in a mindfulness-based intervention?

Probe: *Why?*

3. If you were going to participate in a mindfulness program, what is your typically availability during the week, including typical days or hours of the day?

4. How much would transportation influence your ability to participate in an in-person mindfulness intervention?

Any other closing thoughts or questions?

SHOW CARD 1 – LIKERT SCALE

Not Interested At All

Extremely Interested

1	2	3	4	5	6	7	8	9	10

Appendix 4c: Demographic self-report questionnaire

To be completed by participants after the in-person qualitative interview

Exploring perceptions of and experiences with mindfulness among low-SES populations:
Self-Report Questionnaire to be completed post-interview. Questionnaire will be administered using Qualtrics survey platform; however, paper copies will be available if participants prefer or if computer issues arise.

1.1 Fielded Questionnaire

Thank you for taking the time to talk with us about your experience with and perceptions of mindfulness. To know more about the individuals who we have spoken with please take a moment to answer a few questions about yourself. As a reminder all of the questions are optional and results will be reported in aggregate not individually.

BQ1. What is your age? _____ years old

BQ2. What is your gender?

☐ Male
☐ Female
☐ Other: *specify* ________

BQ3. Are you Latino or Hispanic?

☐ No → ***skip to BQ4***
☐ Yes

BQ3a. Which of the following represents your family's country of origin? ***(check all that apply)***

☐ Cuba
☐ Mexico
☐ Puerto Rico
☐ Spain
☐ South America
☐ Columbia
☐ Dominican Republic
☐ Other Central American
☐ Other: ________________________

BQ4. If you were asked to put yourself into only one of these groups, in which one would you place yourself? ***(select one only):***

☐ Asian
☐ Pacific Islander
☐ African American/Black
☐ Caucasian/White
☐ Native American
☐ Other : ________________________

BQ5. Which of the following best describes your current work situation? ***(select <u>one</u> only)***

- ☐ Working full-time
- ☐ Working part-time
- ☐ Retired
- ☐ Unemployed: Looking for work
- ☐ Unemployed: Not currently looking for work
- ☐ Unemployed due to disability
- ☐ Keeping house or raising children full-time
- ☐ Military
- ☐ Full-time student
- ☐ Other:______________________

BQ6. Please consider your current or most recent job activity, in what kind of business or industry do (did) you work?

____

(For example: manufacturing, sales, health care, restaurant, construction)

☐ I have never been employed → ***skip to BQ7***

BQ6a. What kind of work do/did you do? (Job Title)

____

(For example: registered nurse, personnel manager, supervisor of order department, gasoline engine assembler, grinder operator.)

BQ7. Are you currently married or living with a partner?

- ☐ No → ***skip to BQ8***
- ☐ Yes

BQ7a. Do you share income and expenses with your wife/husband/ partner, or do you each have independent control of your finances?

- ☐ We share income and expenses
- ☐ We <u>do not</u> share income and expenses

BQ8. Which of the following best describes your current spouse/partner's current work situation? ***(select <u>one</u> only)***

☐ Working full-time
☐ Working part-time
☐ Retired
☐ Unemployed: Looking for work
☐ Unemployed: Not currently looking for work
☐ Unemployed due to disability
☐ Keeping house or raising children full-time
☐ Military
☐ Full-time student
☐ Other: ___________________________

BQ9. In what kind of business or industry does/did your current spouse/partner work?

____
(For example: manufacturing, sales, health care, restaurant, construction)

☐ He/she has never been employed → ***skip to BQ10***

BQ9a. What kind of work does/did your spouse/partner do? (Job Title)

____
(For example: registered nurse, personnel manager, supervisor of order department, gasoline engine assembler, grinder operator.)

BQ10. What is the highest grade or level of regular school you have completed?

☐ Elementary School
☐ Junior High
☐ High School
☐ College
☐ Graduate School

BQ11. What is the highest degree you earned? ***(select <u>one</u> only)***

☐ GED
☐ High school diploma or equivalency
☐ Associate degree (Junior College)
☐ Bachelor’s degree
☐ Master’s degree
☐ Doctorate (Phd, EdD, etc)
☐ Professional (MD, JD, DDS, DVM, etc.)
☐ Other: ______________________________
☐ None of the above (less than GED or less than high school graduation)

BQ12. Did you ever attend any other school like a technical, vocational, or trade school?

☐ No
☐ Yes

BQ13. In total, about how many full-time years of education have you had, including 1st grade and all years of school after 1st grade?

________ years

BQ14. Do you currently live alone?

☐ No
☐ Yes → ***skip to BQ15***

BQ14a. How many people are currently living in your household, including yourself?

_________ people

BQ14b. Of these people, how many are under 18?

_________ people ***(if none, write 0)***

BQ14c. Of the adults in your household (including yourself), how many bring income into the household?

_________ people ***(if none, write 0)***

BQ15. How much did YOU earn, before taxes and other deductions, during the past 12 months?
(Please include all income from your job(s), money from government programs, child support, interest and dividends, money from rental property, etc.)

- □ Less than $5,000
- □ $5,000 through $11,999
- □ $12,000 through $15,999
- □ $16,000 through $24,999
- □ $25,000 through $34,999
- □ $35,000 through $49,999
- □ $50,000 through $74,999
- □ $75,000 through $99,999
- □ $100,000 and greater

BQ16**.** How much did your HOUSEHOLD earn, before taxes and other deductions, during the past 12 months? ***(Please include all income from your job(s), your family member's job(s), money from government programs, child support, interest and dividends, money from rental property, etc.)***

- □ Less than $5,000
- □ $5,000 through $11,999
- □ $12,000 through $15,999
- □ $16,000 through $24,999
- □ $25,000 through $34,999
- □ $35,000 through $49,999
- □ $50,000 through $74,999
- □ $75,000 through $99,999
- □ $100,000 and greater

THANK YOU!

Appendix 4d: IRB Form 1 investigator form for Julie Webb

BROWN

BROWN UNIVERSITY
INSTITUTIONAL REVIEW BOARD
HUMAN RESEARCH PROTOCOL

Protocol Title: Perceptions of and experiences with mindfulness: qualitative interviews with low-SES populations / part of the Mindfulness-Based Blood Pressure Reduction (MB-BP) Study
Principal Investigator: Julie Webb (for qualitative interviews only); Eric B. Loucks, PhD is PI on MB-BP Study already IRB approved
Department: Behavioral and Social Sciences / Epidemiology
Funding Source (if no external funding for the project, enter "University"): NIH – UH2 grant
If externally funded, Coeus Institute Proposal # for the project:

(1) Attach to this form the information required for a complete protocol, as outlined beginning on page 3 of this form, Instructions & Information. Additional information about preparing a protocol can be found here.

(2) Select the appropriate type and category number of review. See descriptions of Expedited categories. If no expedited categories completely describe the proposed research, select "Full Board.")
☐ **Expedited #** ☒ **Full Board**

(3) **Investigator Conflict of Interest Statement:**
The *Brown University Conflict of Interest Policy for Officers of Instruction and Research* (*"COI Policy"*) defines the term "Investigator" as "the project director or principal investigator and any other person, regardless of title or position (e.g., full or part-time faculty member, staff member, student, trainee, collaborator, or consultant), who is **responsible** for the **design, conduct, or reporting** of sponsored research." Using this definition of "Investigator," please ensure that all Investigators on this protocol answer questions 3(a) and 3(b) below [attach additional sheets for any Investigators who are not the PI; they only need to answer 3(a) and 3(b)]:
(a) Have you completed a conflict of interest disclosure (i.e. *Annual COI Assurance Form* **or** *COI Reporting Form*) within the past 12 months and is it accurate and up-to-date as of the time of this submission, as required by the COI Policy? (You may access the system here to confirm.)
☐YES ☒NO
(b) Do you have a significant financial interest (SFI) that is related to this research protocol? "Related" could mean the research involves products, technology, intellectual property, or services made, owned, or provided by the entity/ies in which you have an SFI and/or that the SFI could be affected by the proposed research or its results. ☐YES ☒NO

Principal Investigator certifies to the following: *(1)* The rights and welfare of the participants are adequately protected. *(2)* The risks to an individual are outweighed by the potential benefits to him/her or by the importance of the knowledge to be gained. *(3)* This protocol is accurate and complete; if the project scope or design is later changed, the PI will resubmit for review. *(4)* All research personnel, including the PI, has been, or will be, adequately educated in human research protections prior to beginning work on the project.

Principal Investigator signature: [signature] **Date:** 5/14/18

===

(Advisor's signature is required for all graduate/medical student projects.)
Advisor certifies to the following: Advisor has read the protocol and approves of the project.

Advisor's signature: [signature] **Date:** 5/14/18
Print name:

===

For IRB Use Only
FULL BOARD PROTOCOLS - Institutional Review Board Members: If approving the proposed project, please certify to the best of your knowledge to the following: *(1)* IRB Member is familiar with the above described proposed research. *(2)* The rights and welfare of the research participants will be adequately safeguarded by the procedures described. *(3)* The potential benefits justify the risks involved. *(4)* IRB Member has no vested interest in the project.

IRB Member Signature: ______________________ Date: ________

Signature of the Authorized Official of the IRB: ______________________ **Date:** ________

Amendment Request
Institutional Review Board

BROWN

Principal Investigator: Eric B. Loucks, PhD

Title: Mindfulness-Based Blood Pressure Reduction (MB-BP) Study

(#1412001171) **Date of Request**: 8/7/2018

Original Type of Review: ☐ Exempt ☐ Expedited ☒ Full Board

1.) Provide a brief lay summary of the overall project. Include enough detail to allow the IRB to evaluate the requested change(s) within the context of the overall project:
See attached summary

2.) Provide a detailed description of the changes being requested:
See attached summary

3.) State the reason (justification) for the requested amendment:
See attached summary

4.) What is your assessment of how the changes will affect the overall risk/benefit ratio of the study and the willingness of individuals to participate?
See attached summary

5.) New documents / changes to existing documents:

(a) Does the requested amendment require new documents or changes to the approved consent form or other documents?

- ☒ Consent/assent documents (attach revised version with changes highlighted)
- ☒ New/revised instruments (attach -if revised, highlight changes)
- ☐ New/revised advertising materials (attach -if revised, highlight changes)

(b) Do **you** have a significant financial interest (SFI) that is related to this research protocol? "Related" could mean the research involves products, technology, intellectual property, or services made, owned, or provided by the entity/ies in which you have an SFI and/or that the SFI could be affected by the proposed research or its results. ☐ YES ☒ NO

If **YES**, has this conflict been previously disclosed to the IRB?

☐ YES
☐ NO

If **NO**, please describe: Click or tap here to enter text.

6.) Personnel Changes/New Investigator(s):

The *Brown University Conflict of Interest Policy for Officers of Instruction and Research ("COI Policy")* defines the term "Investigator" as "the project director or principal investigator ***and any other person, regardless of title or position*** (e.g., full or part-time faculty member, staff member, student, trainee, collaborator, or consultant), who is **responsible** for the **design, conduct, or reporting** of sponsored research."

Using this definition of "Investigator," have you added any new Brown Investigators to this project since your most recent IRB approval (initial approval, or approval of your most recent progress report or amendment)?

☐ YES ☒ NO (If no, stop here. No need to proceed to the next question.)

7.) New Investigator(s) Conflict of Interest:

Any **new Investigators** (if applicable) must answer the below questions. Please include additional sheets if needed to identify all new Investigators by name and title.

Name of Investigator: Click or tap here to enter text.

Title:

(a) Have you completed a conflict of interest disclosure (i.e., *Annual COI Assurance Form* **or** *COI Reporting Form*) within the past 12 months and is it accurate and up-to-date as of the time of this submission, as required by the COI Policy? (You may access the system here to confirm.)

☐ YES ☐ NO

(b) Do you have a significant financial interest (SFI) that is related to this research protocol? "Related" could mean the research involves products, technology, intellectual property, or services made, owned, or provided by the entity/ies in which you have an SFI and/or that the SFI could be affected by the proposed research or its results. ☐ YES ☐ NO

Please note that if any new Investigators have an SFI related to this research protocol, the IRB may require modifications to the informed consent document(s).

If **YES**, please describe: Click or tap here to enter text.

PI Electronic Signature: 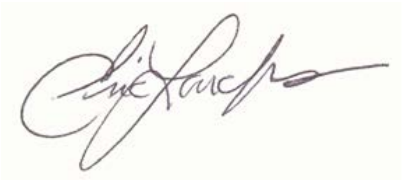
PI Name: Eric B. Loucks, Ph.D.
Date: 8/7/2018

1. **Provide a brief lay summary of the overall project. Include enough detail to allow the IRB to evaluate the requested change(s) within the context of the overall project.**

Original lay summary providing broad overview of the project:

The World Health Organization reported that suboptimal blood pressure (BP) is responsible for more than half of cardiovascular disease mortality world-wide. Furthermore, greater than half of those with hypertension have uncontrolled BP. *A 2009 Institute of Medicine report recommended prioritizing research to "Compare the effectiveness of mindfulness-based interventions (e.g. yoga, meditation, deep breathing training) and usual care in treating… cardiovascular risk factors."* Evidence-based mindfulness interventions, including Mindfulness-Based Stress Reduction, may have some effects on blood pressure, where a recent meta-analysis and systematic review of 4 randomized controlled trials demonstrated significant effects, but evidence of heterogeneity in effect sizes. The methodologically highest quality studies had the smallest effect sizes (range 0-5 mmHg). Mindfulness-Based Stress Reduction (MBSR) has been customized to a number of disease processes, such as Mindfulness-Based Cognitive Therapy for patients with recurrent depression, and Mindfulness-Based Relapse Prevention for patients with substance use addictions. Effect sizes have been increased by customizing mindfulness interventions to diseases of interest. The same may be true for hypertension, *however mindfulness interventions customized for prehypertensive/hypertensive patients have never been investigated.* *Until methodologically rigorous studies to evaluate customized interventions for hypertension are performed, we will not know if the observed preliminary effects of general mindfulness interventions on blood pressure reduction could be much more effective with a tailored approach.* Consequently, we propose to conduct a stage I behavioral therapy intervention study to evaluate whether MBSR customized to prehypertensive and hypertensive patients has the potential to provide clinically relevant reductions in BP. Consequently the specific aims are:

Stage 1a: Therapy Development/Manual Writing

1. To outline and evaluate key novel elements of mindfulness-based hypertension therapy (MBHT), customized from the evidence-based MBSR. *We hypothesize that the most important novel element will be generation of mindfulness skills specifically applied to hypertension risk factors such as diet, physical activity, obesity, alcohol consumption and antihypertensive medication adherence.* This aim will be achieved using (1) focus groups of participants undergoing the MBHT behavioral intervention, (2) discussion with experts (including cardiologists, epidemiologists, mindfulness experts, mindfulness intervention instructors) prior to, and following pilot testing of MBHT in participants, and (3) clinical judgment of the investigators performing the intervention.
2. To determine effectiveness of MBHT on primary outcomes (systolic blood pressure, retention rates, recruitment rates, and adverse effects) and secondary outcomes (hypertension risk factors such as diet, physical activity, obesity, and antihypertensive medication adherence) in hypertension subgroups, specifically participants with (1) prehypertension, (2) controlled hypertension, and (3) uncontrolled hypertension. Initial decisions about the targeted sample based on hypertension status will be made.
3. To develop an MBHT therapist manual and training program, including procedures for training, supervising, and evaluating therapists. Furthermore, acceptable therapist characteristics will be developed. The manual and training program will include themes such as specification of unique and common elements of MBHT *vs.* other interventions, description of interventions excluded from MBHT, and specification of key treatment

parameters such as frequency and duration of treatment, session length, topics addressed, sequence of sessions, as well as therapist adherence and competency measures. The MBHT training will consist of a therapist manual, a formal didactic training seminar, and at least one closely supervised training session.

Stage Ib: Pilot Trial

4. To determine whether a mindfulness-based hypertension therapy (MBHT) intervention, customized from the evidence-based MBSR, has promise to be an effective behavioral therapy for participants with hypertension and/or prehypertension. We will perform a randomized controlled pilot trial for MBHT *vs.* enhanced usual care control. *We hypothesize that MBHT will have adequate recruitment rates (≥10% of prehypertensive/hypertensive participants invited from physicians' offices), fairly low drop out rates (<15%), and medium effect sizes (e.g. 5-10 mmHg systolic BP) for reduction in blood pressure.*

These findings will provide publishable pilot data that will inform future randomized clinical trials that evaluate effects of MBHT on long-term changes in blood pressure *vs.* usual care and active control groups. *If proven effective, MBHT could be offered as a complementary program in the prehypertensive/hypertensive patient population that contributes to over half of the cardiovascular disease mortality world-wide.*

Project update as of August 2018:

The Mindfulness-Based Blood Pressure Reduction (MB-BP) Study, formerly known as Mindfulness-Based Hypertension Therapy (MBHT), is currently in Year 2 of a five year NIH UH2 grant.

With the exception of the two year follow ups, data collection for the single arm trial (Stage 1a) of the project has ended and analysis of this dataset is on-going. In total we ran three separate Mindfulness-Based Blood Pressure reduction (MB-BP) intervention courses, with a total sample size of 43 eligible participants who both enrolled and completed the 9-week intervention. We had eight eligible participants enroll in the study who later decided to not complete the course and/or who were lost to follow up.

In June 2017 we began enrollment for the next phase of the study (referenced as Stage 1b above, but now called 'Stage 2a'), which is to conduct a Randomized Controlled Trial (RCT) with *enhanced usual care*. Data collection for this phase will continue through September 2019. To date, 82 individuals have been enrolled and randomized into Stage 2a.

2. Provide a detailed description of the changes being requested (Use additional pages, if necessary):

This amendment covers the following four requests/revisions which are outlined below:

(1) *Permission to conduct abbreviated 1 year follow up assessments with participants randomized to the intervention arm only.* In our last amendment (#11) we sought to remove long term follow up with Stage 2a participants. However, after further discussions with NCCIH program officers we realized that in order to meet our project aims we will need to analyze the long term effects of the intervention on our primary and secondary outcomes. That being said, we are asking for permission to conduct abbreviated 1 year follow up assessments with intervention participants only. To carry this out we will use the attached revised informed consent form (v.3.1) with all newly enrolled participants. Individuals who have already been consented and enrolled into the study, will be given an addendum to informed consent (see attached) which notifies them of the changes to the study protocol (i.e., request to follow up with intervention arm at 1 year). The abbreviated 1 year follow up assessment is also attached for review and approval and consists of a subset of already approved measures estimated to take 45 minutes to an hour to complete. Participants will be given $50 USD for completion of the 1 year follow up.

(2) *Request to use a general release form to video record mindfulness classes for the purposes of quality control and instructor training / fidelity.* The attached general liability release form would be provided to participants prior to participation in the 9-week mindfulness course. The release form seeks permission to record the intervention sessions for the purpose of instructor training, fidelity and quality control. The recording would capture only the instructor's face and not that of the participants. However, participants' voices would be recorded. Since this is something that some individuals may not feel comfortable with, research staff will be trained to review this protocol with all individuals seeking to enroll in our study at the time of enrollment and informed consent. Note that there is already language in the currently approved informed consent form that discusses recording of the intervention. In order to take part in the intervention, individuals must be comfortable with the recording since this is a necessary component of the research study.

(3) *Removal of actigraph devices (i.e., fitbits) from study protocol.* In an effort to reduce participant burden we are seeking permission to discontinue use of fitbit devices to monitor participants' physical activity. This revision is reflected in the revised informed consent form.

(4) *Permission to add a block of questions to the 6 month in-person follow up assessment to be asked of control group participants only.* In an effort to better understand the experience of control group participants in the study between the time of their enrollment and the time of their final six month follow up assessments, we would like to ask a block of questions (see attached questionnaire) that would collect information on behavior and attitudes that may contribute to the interpretation of results, such as engagement in mindfulness practices or changes in health behaviors that took place independent of the study intervention. The additional questions are estimated to take around 15 minutes to complete and would take place at the end of the 6 month in-person follow up assessment.

3. State the reason (justification) for the requested amendment:

Justification for all revisions is provided above.

4. What is your assessment of how the changes will affect the overall risk/benefit ratio of the study and the willingness of individuals to participate?

The revisions outlined in this amendment do not increase participant burden beyond what was previously approved, If anything participant burden will be reduced as the 1 year follow up is an abbreviated assessment as compared to the other two follow ups. Additionally, removal of the fitbits from study protocol to monitor physical activity offers a significant decrease in participant burden. The additional questions for study control group members are brief and will allow control group members a platform to communicate about their experience, positive or negative, in the study. Finally, the use of the general release form and disclosure about recording intervention sessions at the time of enrollment allows participants to be fully informed of the study protocol. We do not anticipate any increase risk to study participants.

5. Does the requested amendment require new documents or changes to the approved consent form or other documents?

See enclosed attachments related to this amendment:

Appendix 1 - Revised Informed Consent (v.3.1) – with track changes

Appendix 2 – Addendum to consent form to be signed by already enrolled participants

Appendix 3 – Abbreviated 1 year follow up assessment for intervention group only

Appendix 4 – General liability release form

Appendix 5 – Additional questions to be asked of control group participants at the time of the 6 month in-person follow up assessments.

Appendix 1 -

Revised Informed Consent

(v.3.1 – with track changes)

For newly enrolled participants to complete

at time of screening.

BROWN UNIVERSITY
CONSENT FOR RESEARCH PARTICIPATION

The Mindfulness-Based Blood Pressure Reduction (MB-BP) Study
Version 3.1, ~~May 29~~August 7, 2018

KEY INFORMATION:
You are invited to take part in a Brown University research study called MB-BP. Your participation is voluntary.

- PURPOSE: In this study, we are looking to see if mindfulness practices improve blood pressure, and if education about hypertension risk factors may also improve blood pressure.
- PROCEDURES: If eligible and selected to receive the intervention, you will be asked to take part in a 9-week mindfulness course where you will free receive training in meditation, mindful movements, and the roles of things like diet, physical activity and medication in reducing blood pressure. As part of the project, you will be asked to participate in multiple health assessments before and after the class. We will also ask you to use ~~an activity monitor to record physical activity and~~ a home blood pressure monitor to take your blood pressure at home nine times throughout the study.
- TIME INVOLVED: The mindfulness class involves 30 hours of class time and up to 48 hours of at home practice spread out of the course of 9-weeks. The three research assessments are estimated to take around four hours each or around 12.5 hours in total. The assessments will take place at three times throughout the study: at baseline before class begins and then at 10 weeks and 6 months follow up. The total estimated time involved for this study is up to 90.5 hours spread out over 6 months. An abbreviated 1 year follow up assessment will also be administered to individuals randomized to the intervention group to assess long term effects of the mindfulness class.
- COMPENSATION: You will receive $~~100~~ 50 USD ~~in total~~per follow up as compensation for your time.
- RISKS: The risks to you in this study are small. They include possible discomfort during research assessments and/or the 9-week intervention as well as possible physical injury during the mindfulness intervention. All aspects of the study are voluntary.
- BENEFITS: There are no guaranteed direct benefits to participating in this study. We are investigating whether or not the 9-week intervention actually works to lower blood pressure.
- ALTERNATIVES TO PARTICIPATION: A number of different therapies, including antihypertensive medication, diet changes, physical activity, and reducing excessive alcohol consumption may also be beneficial for reducing blood pressure. Education about these therapies are integrated into this course, but other forms of these alternative therapies are also available in the community.

This remainder of this form will explain in detail the purpose of the study, how the study will be carried out and what you will be expected to do. It will also explain the possible risks and possible benefits of being in the study. If any part of the following description is not clear to you, you are encouraged to contact the researcher to answer any questions before you decide whether to take part in the study. If you decide to participate, please fill out and sign the last page of this form.

1. Researcher(s):

The Principal Investigator on this project is Dr. Eric B. Loucks. He can be reached at 401-863-6283 or by email at eric.loucks@brown.edu. Research staff working on the study can be contacted by phone or email at: 401-400-4768 and mindfulness@brown.edu.

2. What is this study about?

The purpose of the study is to investigate the impact of mindfulness practices and health education on blood pressure. You have been selected for this study because you expressed interest in the project and because you met entrance criteria for having prehypertension or hypertension, or another cardiovascular risk factor that could be influenced by this program. Eligibility for the study is still being assessed. Therefore, it is possible you may not be eligible for the study even after signing this consent form. Your participation in this study is voluntary and can be withdrawn at any point in the project.

In order to assess the effects of the customized mindfulness intervention, you will be asked to complete some questionnaires and laboratory assessments before and after the intervention. Specifically, assessments will be completed at: baseline, 10 weeks, and 6 months. To express our gratitude for participation, you will be given $50 at each of the follow ups ($100 USD total). As part of the study you will also be given a wireless blood pressure monitor (estimated value of $90) to use throughout the study. Members of the intervention group will also be asked to complete an abbreviated 1 year follow up assessment estimated to take 45-60 minutes to complete in order to assess long term effects of the mindfulness intervention.

This is a Randomized Control Trial. Participants enrolled into the study will be randomly assigned to one of two groups: (1) the intervention group or (2) the wait-list control group. The wait-list control group will be given the opportunity to participate in the intervention after the six month follow up assessments are completed. Both the intervention and the control group will be asked to participate in the research assessments.

3. What will I be asked to do?

If you agree to participate, you will be asked to consent to the following:

a) Participation in a screening interview in which you will be asked questions about past and present mental health, including depression and suicide (previously completed with your verbal consent).

b) Completion of an in-person screening assessment, during which your blood pressure, height, weight and other basic demographic and health data will be collected and assessed in order to determine eligibility for the study.

c) Completion of questionnaires administered in-person and online that ask about a wide range of topics, including your diet, physical activity, smoking, medication use, personality, emotions, attention and past experiences, including stressful or traumatic experiences. These questions will probe sensitive psychological areas, including physical, emotion and sexual abuse. These questionnaires may take up to 3 hours to complete. By completing the interviews and questionnaires, you are giving the researchers permission to use the information you have provided. You have the right not to answer any of the questions.

d) Directly assessed blood pressure, heart rate, height, weight, physical activity, and antihypertensive (blood pressure) medication use at baseline and after the mindfulness course. ~~Physical activity will be assessed for a week at a time using small actigraphy monitors (i.e., Fitbits) that attach to your wrist.~~ If you take antihypertensive medication, we will provide you with an electronic bottle cap that will automatically record when the pill bottle is opened during the study. This will help us measure how often the medication is used. We will also provide you with a wireless blood pressure

monitor and will ask that you take your blood pressure at home systematically during each of the research assessment periods (i.e., baseline, 10 weeks, and 6 months).

e) You will be asked to perform some cognitive tasks. Some of these tasks may involve computer-based tests of attention or decision-making. Together these tests may take as long as 20 minutes.

f) During the in-person assessments you will also be given a battery of stress tests that are designed to induce a stress response so that we can monitor your cardiovascular response and recovery.

g) There will be two follow ups that take place after 10 weeks and 6 months from the start of the intervention. The assessments and questionnaires you will complete for the follow ups will be the same as those completed at baseline. An abbreviated 1 year follow up will also be administered to members of the intervention group.

If randomized into the intervention group…

h) You will be asked to participate in the mindfulness program, which consists of 9 weekly sessions of 2.5 hours each and will include one 7.5 hour weekend retreat. Daily at home practice assignments may take as long as one hour and consist of practicing mindfulness exercises with the aid of a guided meditations and completing worksheets related to stress, thoughts, and common reactions to various types of events. Individuals randomized into the wait-list control group, will be invited to take part in the mindfulness class after the completion of the 6 month follow up assessments.

i) Class sessions may be recorded so we can analyze the quality of the treatment you receive. The recordings will be transcribed so that we may analyze the text. The recordings will be identified by study number, will only be heard by study staff and will be destroyed after transcription.

j) You may be asked to complete a few short questionnaires each week during the 9 week condition.

Table Summarizing Activities and Time Commitment for this Study.

**Activity**	**Estimated Time Commitment**
In-person screening assessment	0.5 hours
Baseline<br>In-person assessment<br>Online questionnaire<br>At home health monitoring (~~e.g., fitbit, BP, etc.~~i.e., blood pressure)	 2.5 hours<br>1.0 hours<br>0.25 hours
Intervention*<br>Mindfulness course<br>Home practice assigned during course<br><br>**Intervention group only; control group will be invited to take part in a class post 6 month follow up but it will not be required as part of the study.*	Nine 2.5 hour sessions<br>7.5 hour all day retreat<br>Up to 1hr daily home practice assignments<br>*Total course time: 30.0 hours*<br>*Max. practice time: 48 hours*

Follow Ups – 10 week ($50) and 6 month ($50)<br>In-person assessments<br>Online questionnaires<br>At home health monitoring (i.e., blood pressure)<br>1 year follow up (*intervention group only*) ($50)~~(e.g., fitbit, BP, etc.)~~	Total follow up time:<br>5.0 hours<br>2.0 hours<br>~~1.0~~0.5 hours<br>0.75 hours
**TOTAL ESTIMATED TIME COMMITMENT**	11.75~~12.5~~ hours – CONTROL<br>90.5 hours - INTERVENTION

Your participation in this study may last up to 6 months in duration (1 year for intervention group members) and is estimated to take up to 11.75 or 9~~8~~0.5 hours in total depending on which group you are assigned to.

Feedback: At the end of the study, you will receive individual feedback about the changes that occurred since the first assessment. Specifically, you will receive an individualized handout listing % change (increase or decrease) on scales of attention, stress, mood, health behaviors, weight, and blood pressure across the study.

Uncontrolled Hypertension: If during the in-person assessments it appears that you have stage 2 uncontrolled hypertension (your average systolic blood pressure reading is 140 mmHg or greater and/or your average diastolic blood pressure is 90 mmHg or greater) AND you indicate to us that you are not currently being treated for hypertension, then we will be requesting your permission to contact your health care provider to notify him/her of the blood pressure results. If you do not have a health care provider and/or do not have health insurance, our staff will provide you with resources to help you search for one; although we cannot guarantee that we will be able to find you one nor that it will be free. It is your choice on whether or not you would like us to follow up with your health care provider. Your participation in the study is not contingent on this communication; however, it is our recommendation that all individuals with uncontrolled hypertension be under the care of a health care professional.

4. Will I be paid?

You will be given $50 at each of the follow up assessments ($100 USD total for control group members and up to $150 USD for intervention group members).

5. What are the risks?

The risks to you in this study are small. The questionnaires used in the study are routine, standardized forms for epidemiologic research. Certain questions may be upsetting as they may probe sensitive psychological areas and inquire about upsetting or traumatic events, including physical, sexual or emotional abuse and/or current psychiatric symptoms. The cognitive tests and stress battery may also invoke a stress response that may be uncomfortable. All aspects of the study are voluntary; you have the right to skip anything during the study that makes you uncomfortable.

Meditation-based interventions may results in discomfort with attention to unpleasant thoughts, feelings or body sensations. Some individuals may experience an initial increase in undesirable feelings with increased attention to them.

It is possible that injuries could be sustained during the study either from the gentle mindful movements (i.e., yoga), or from physical activities that participants engage in as a way to reduce blood pressure. To help limit this, you will receive a handout showing the yoga poses that will be offered during the course that you can show your health care provider so that they can advise on which poses to do, and which to avoid. Modifications of poses will be available as needed. None of the poses (or the yoga as a whole) are mandatory to be done. You will also be encouraged to explore physical activities that promote strength and conditioning as a way to reduce blood pressure. You will be encouraged to not go beyond any physical limits of your body, and will be encouraged to ask your healthcare provider about advised physical activities and mindful movements if you have any physical limitations.

While physical and mental injury is always a possibility the potential for harm is limited. Note that a research injury is any physical or mental injury or illness caused by your participation in the study. If you are injured by a medical treatment or procedure that you would have received even if you were not in the study, that is not a research injury. To help avoid research injury and potential added medical expenses, it is important to follow all study directions carefully. If you are covered by insurance and suffer a research injury, it is possible that some or all of the costs of treating your

condition could appropriately be billed to your insurance company. If such costs are not covered your health insurance company, it is possible you would have to pay for these costs out of pocket. Brown University's policies do not cover payment for such things as lost wages, medical care expenses, or pain and suffering.

Precautions should be taken to avoid injuries. If you do become injured during the study, you should call your doctor immediately. You should also alert the study staff that you have been injured. Heart attack and sudden death related to heart problems have been known to occur in people while they are exercising. This is very rare, however. Estimates of sudden cardiac death range from 0 to 2 per 100,000 hours. However, the researchers cannot guarantee that no complications will happen to you.

6. What are the benefits?

We cannot and do not guarantee or promise that you will receive any direct benefits from this study. However, participation in the study creates the potential benefit of a) identifying effective treatments for elevated blood pressure, b) gaining knowledge of the effects of mindfulness practices, c) receiving information about your psychological and physical functioning. As part of the study, you will receive a wireless blood pressure monitor that will be yours to keep. This monitor may provide additional opportunity to monitor your blood pressure at home, which may benefit your health by providing additional biofeedback.

7. How will my information be protected?

Your responses for this study will be kept confidential. All data that we collect will be linked to a study ID# instead of your name. All questionnaires in this study will be filled out through an online survey or a paper version of the survey if you prefer. All of these questionnaires will be linked solely to your study ID#, so that your identity is protected and your answers are confidential. All paper forms and data collection tools will be kept in a locked filing cabinet in a secure location. Study consent forms, including this one, will be kept in a locked filing cabinet separate from the research data. Note that although these measures have been taken to protect your personal information, complete confidentiality cannot be guaranteed when transmitting information over the internet.

While your confidentiality is protected to the extent of the law, there are limitations to confidentiality. If your questionnaire responses indicate that you pose a serious danger to yourself or to another person, then a collaborator (Dr. Ellen Flynn) who is a licensed psychiatrist, may contact you to discuss your responses and possible referral to a treatment provider. Questionnaire items that may warrant follow-up include endorsements of statements about hurting yourself, any high scores in depression, anxiety, or other clinically significant problems. You should also know that there are times when the law might require the release of your responses without your permission. For example, State law requires researchers to report abuse or neglect of children to the Department of Children, Youth and Families (DCYF). State law also requires researchers to report abuse or neglect of people age 60 and older to the Division of Elderly Affairs.

The findings of the study may be used for medical publication. Your name will not be used in any published reports about this study. Results will be reported in a summarized manner in such a way that you cannot be identified. All personally identifiable information will be "de-identified" and only a unique code number will be used. Study records will be identified with a unique code number and initials. All study records and specimens will be stored in a secure storage area.

Keeping study records: The Principal Investigator for this study will keep your research records indefinitely for research purposes.

Certificate of Confidentiality: This research is covered by a Certificate of Confidentiality from the National Institutes of Health. The researchers with this Certificate may not disclose or use information, documents, or biospecimens that may identify you in any federal, state, or local civil, criminal, administrative, legislative, or other action, suit, or proceeding, or be used as evidence, for example, if there is a court subpoena, unless you have consented for this use. Information, documents, or biospecimens protected by this Certificate cannot be disclosed to anyone else who is not connected with the research except, if there is a federal, state, or local law that requires disclosure (such as to report child abuse or communicable diseases but not for federal, state, or local civil, criminal, administrative, legislative, or other proceedings, see below); if you have consented to the disclosure, including for your medical treatment; or if it is used for other scientific research, as allowed by federal regulations protecting research subjects.

The Certificate cannot be used to refuse a request for information from personnel of the United States federal or state government agency sponsoring the project that is needed for auditing or program evaluation by the National Center for Complementary and Integrative Health, which is funding this project. You should understand that a Certificate of Confidentiality does not prevent you from voluntarily releasing information about yourself or your involvement in this research. If you want your research information released to an insurer, medical care provider, or any other person not connected with the research, you must provide consent to allow the researchers to release it. The Certificate of Confidentiality will not be used to prevent disclosure as required by federal, state, or local law of situations of child abuse and neglect, or harm to self or others.

Finally, Brown University staff sometimes review studies like this one to make sure they are being done safely and correctly. If a review of this study takes place, your records may be examined. The reviewers will protect your confidentiality.

8. Are there any alternatives to this study?

A number of different therapies, including antihypertensive medication, diet changes, physical activity, and reducing excessive alcohol consumption may also be beneficial for reducing blood pressure. Education about these therapies are integrated into this course, but other forms of these alternative therapies are also available in the community.

9. What if I want to stop?

You do not have to be in this study if you do not want to be. Even if you decide to be in this study, you can change your mind and stop at any time. If you refuse to participate in or leave the study, your current or future relationship with Brown University as well as with your physician will; not be affected. If you decide not to participate, or if you quit the study, we will provide you with referrals for alternative treatments, if desired.

10. Who can I talk to if I have questions about this study?

If you have any questions about your participation in this study, you can call the Senior Project Coordinator, Frances Saadeh, at 401-400-4768 or email at mindfulness@brown.ledu. You may also contact the Principal Investigator at any time: Dr. Eric B. Loucks, email: eric.loucks@brown.edu, telephone (401) 863-6283.

A description of this clinical trial will be available on http://www.Clinical Trials.gov, as required by U.S. Law. This Web site will not include information that can identify you. At most, the Web site will include a summary of the results. You can search this Web site at any time.

11. Who can I talk to if I have questions about my rights as a participant?

If you have questions about your rights as a research participant, you can contact Brown University's Human Research Protection Program at 401-863-3050 or email them at IRB@Brown.edu.

12. Consent to Participate

Your signature below shows that you have read and understood the information in this document, and that you agree to volunteer as a research participant for this study.

You will be offered a copy of this form.

____

Participant's Signature and Date / PRINTED NAME

____

Research Staff Signature and Date / PRINTED NAME

Appendix 2 -

Addendum to Informed Consent

To be signed by participants who have already enrolled in the study and who opt to complete the abbreviated 1 year follow up assessment

Brown University Consent to Participate in a Research Study
Addendum to provide additional information to subject after original consent and to seek permission to complete a 1 year follow up assessment

---

IRB Study #__1412001171___________
Consent Form Version Date: _8/7/2018__
Title of Study: Mindfulness-Based Blood Pressure Reduction (MB-BP) Study
Principal Investigator: Eric B. Loucks, PhD__

Contact Information - If you have questions about study procedures at any time you may contact the researchers: Dr. Eric B. Loucks, email: eric.loucks@brown.edu, telephone (401) 863-6283. If you would like more information about the rules for research studies, or the rights of people who take part in those studies, you may contact the Brown University Human Research Protection Program, telephone number 1-866-309-2095 or 401-863-3050.

The following information should be read as an addition to the original Consent form that you read and signed at the beginning of the study. Unless specifically stated otherwise in the following paragraphs, all information contained in that original Consent Form is still true and remains in effect. Your participation continues to be voluntary. You may refuse to participate, or may withdraw your consent to participate at any time, and for any reason, without jeopardizing your future care at this institution or your relationship with your health care provider.

New or additional information
You are being asked to participate in a 1 year follow up assessment that will take place in-person at the Brown University Mindfulness & Cardiovascular Health Lab located at 121 South Main Street, Providence, RI 02912. The 1 year follow up assessment is estimated to take around 45 to 60 minutes to complete and will consist of a subset of measures that you previously completed as a participant in the MB-BP Study. Specifically, we will be taking your blood pressure and weight as well as asking you a series of questions about hypertension medications, mindfulness practices, dietary consumption, and a brief series of questions about yourself. You will receive $50 USD for your time and effort.

Subject's Agreement: (*check each item below*)
- ☐ I have read the information provided above.
- ☐ I have been given a copy of the original consent form that I signed at the time of enrollment and have had an opportunity to review this document.
- ☐ I have asked all the questions I have at this time. I voluntarily agree to continue to participate in this research study.

_______________________________________ ______________
Signature of Research Subject Date

____
Printed Name of Research Subject

_______________________________________ ______________
Signature of Research Team Member Obtaining Consent Date

____
Printed Name of Research Team Member Obtaining Consent

Appendix 3 -

1 year follow up assessment

To be administered to individuals in the intervention group only. Estimated to take 45-60 minutes to complete and consists of a subset of already approved measures.

MB-BP STUDY: 1 YEAR IN PERSON FOLLOW UP ASSESSMENT

PID. Participant ID # __________
BA01. Staff ID # __________
BA02. Today's date (MMDDYY): ___________

Blood Pressure:
BA03a. Blood pressure 1st reading, systolic blood pressure: _________ mmHg
BA03b. Blood pressure 1st reading, diastolic blood pressure: __________ mmHg
BA03c. Blood pressure 2nd reading, systolic blood pressure: _________ mmHg
BA03d. Blood pressure 2nd reading, diastolic blood pressure: __________ mmHg
BA03e. Blood pressure 3rd reading, systolic blood pressure: _________ mmHg
BA03f. Blood pressure 3rd reading, diastolic blood pressure: __________ mmHg

If the difference of the 2nd and 3rd systolic BP reading is 20 mmHg or greater OR the difference of the 2nd and 3rd diastolic reading is 10 mmHg or greater, then repeat the BP readings. Otherwise, skip to BA07.

BA06a. Repeated blood pressure 1st reading, systolic blood pressure: _________ mmHg
BA06b. Repeated blood pressure 1st reading, diastolic blood pressure: __________ mmHg
BA06c. Repeated blood pressure 2nd reading, systolic blood pressure: _________ mmHg
BA06d. Repeated blood pressure 2nd reading, diastolic blood pressure: __________ mmHg
BA06e. Repeated blood pressure 3rd reading, systolic blood pressure: _________ mmHg
BA06f. Repeated blood pressure 3rd reading, diastolic blood pressure: __________ mmHg

Blood Pressure Safety Protocol – The out-of-range blood pressure values are as follows: systolic blood pressure >200 mmHg or <90 mmHg; diastolic blood pressure >110 mmHg.

In absence of symptoms (chest pain, shortness of breath, dizziness, headache), for a SBP>200 or DBP>110 or both, we will strongly encourage participants to see their doctor right away or to go to urgent care. If there are symptoms, we will immediately call 911.

In absence of symptoms (chest pain, shortness of breath, dizziness, passing out), for a SBP<90, we will strongly encourage participants to see their doctor right away or to go to urgent care. If there are symptoms, we will immediately call 911.

Follow Safety Protocol for Uncontrolled Hypertension (140/90 mmHg or greater)

BA07. Blood pressure cuff size used: □ S □ Reg □ L □ XL
BA08. Arm that cuff was placed on: □ L □ R

BP monitor BP monitor used:
❍ Unit #1 - HEM-705CP (1)
❍ Unit #2 - HEM-705CP (2)
❍ Other (specify: (3) ____________________

We are now going to take your weight. *Have ppt remove his/her shoes and empty his/her pockets. Remove bulky clothing as well.*
BA10. Weight: ______ . __ lb (one decimal place)

Medications (ME)

The next questions are about medications used to treat hypertension or high blood pressure.

ME01. Not including vitamins and supplements, do you currently take any prescription medications or over-the-counter drugs for high blood pressure?

- ☐ No
- ☐ Yes
- ☐ Don't know
- ☐ Prefer not to answer

ME01a IF NO, Have you ever taken medication for high blood pressure?

- ☐ No → ***skip to end of medications questions***
- ☐ Yes → ***skip to end of medications questions***

If you brought your medications with you, please take them out now as we will use them to complete the next section.

ME01b INTERVIEWER CHECKPOINT - Please select one:

- ☐ Rx info not known or available – ***skip to end of ME questions; will need to follow up***
- ☐ Ppt brought medications -- ***continue***
- ☐ Ppt did not bring medications but knows info – ***continue, may need follow up***

ME01c In total how many different medications and/or over the counter drugs do you currently take for high blood pressure? Again, do not count vitamins or supplements. _________ # of BP meds

Next we're going to ask you questions about each of the medications you currently take for high blood pressure.

ME02-10a. What is the name of the first prescription medication or over-the-counter drug that you take?

____

☐ Don't know ☐ Prefer not to answer

ME02-10b. What is the dosage form?

Oral
- ☐ Pill, tablet, or capsule
- ☐ Sublingual or orally-disintegrating tablet
- ☐ Liquid solution or suspension (drink, syrup)
- ☐ Powder

Topical
- ☐ Liquid, cream, gel, or ointment
- ☐ Ear drops (otic)
- ☐ Eye drops (ophthalmic)
- ☐ Skin patch (transdermal)

Inhaled
- ☐ Inhaler or nebulizer

Injected
- ☐ Injection

Suppository
- ☐ Rectal (e.g., enema)
- ☐ Vaginal (e.g., douche, pessary)

Other:
- ☐ Other _________
- ☐ Don't know
- ☐ Prefer not to answer

ME02-10c. How frequently do you take it?

- ☐ _________ times per day
- ☐ _________ times per week
- ☐ _________ times per month
- ☐ Don't know
- ☐ Prefer not to answer

ME02-10d. What is the strength? ***(Record strength of how it is actually taken, not how it is prescribed.)***_________ %

- ☐ _________ mg
- ☐ _________ mcg
- ☐ _________ grams
- ☐ _________ I.U.
- ☐ _________ Other unit: _____________
- ☐ Don't know
- ☐ Prefer not to answer

ME02-10f. Do you take it regularly or only as needed?

- ☐ Regularly
- ☐ Only as needed
- ☐ Don't know
- ☐ Prefer not to answer

ME02-10g. For how long have you been taking it?

- ☐ For _________ days
- ☐ For _________ weeks
- ☐ For _________ months
- ☐ For _________ years
- ☐ Don't know
- ☐ Prefer not to answer

ME02-10h. What is the medication used for?

- ☐ High blood pressure
- ☐ Prescribed for something other than high blood pressure but also acts as a hypertensive medication (*describe:* ____)

ME02-10i. ***Interviewer comments*:**

____

Repeat for all blood pressure medications the participant is currently taking.

<u>Summary of Self-report scales for participant to complete at 1 year follow up</u>:

The below measures are all found on the proceeding pages and represent a sub-set of the already approved measures being used in MB-BP Stage 2a assessments.

Food Frequency Questionnaire (FFQ) *–four-page standardized food frequency questionnaire produced by the Harvard School of Public Health*

Alcohol Use questions – *same questions used in the currently approved follow up questionnaires*

SLEEP *– 1 question only taken from the Pittsburgh Sleep Quality Index*

Multidimensional Assessment of Interoceptive Awareness (MAIA) - *standardized scale*

Difficulties in Emotion Regulation Scale (DERS) - *standardized scale*

Mindfulness Practice questions *found on proceeding pages*

FOOD FREQUENCY QUESTIONNAIRE: *RA Script to be read to participants:* At this time, we are going to have you complete a few forms on your own. The first is a Food Frequency Questionnaire that will ask you about the types of foods and drinks that you consume. It should take around 15 to 20 minutes to complete. Please let me know if you have any questions.

[ACTUAL MEASURE IS INCLUDED AS ATTACHMENT]

Alcohol Consumption

A drink of alcohol is defined as 1 can or bottle of beer, 1 glass of wine, 1 can or bottle of wine cooler, 1 cocktail, or 1 shot of liquor.

AC1_01. During the past 30 days, how many days per week or per month did you have at least 1 drink of any alcoholic beverage? [if none, ***skip to next section***]

____

AC1_02. On the days when you drank, about how many drinks did you drink on average?

____

AC1_03. ***Men:*** Considering all types of alcoholic beverages, how many times during the past 30 days did you have **5** or more drinks on an occasion?

Women: Considering all types of alcoholic beverages, how many times during the past 30 days did you have ***4*** or more drinks on an occasion?

____

SLEEP - The following question relates to your usual sleep habits during the past month only. Your answer should indicate the most accurate reply for the majority of days and nights in the past month.

SL1_04. **During the past month**, how many hours of actual sleep did you get on average at night? (This may be different than the number of hours you spent in bed.)

AVERAGE HOURS OF SL1_EEP PER NIGHT ___________

□ I do not know
□ I prefer not to answer

(MAIA) Below you will find a list of statements. Please indicate how often each statement applies to you generally in daily life.

	0- Never	1	2	3	4	5 - Always
IA1_01. When I am tense I notice where the tension is located in my body.	☐	☐	☐	☐	☐	☐
IA1_02. I notice when I am uncomfortable in my body.	☐	☐	☐	☐	☐	☐
IA1_03. I notice where in my body I am comfortable.	☐	☐	☐	☐	☐	☐
IA1_04. I notice changes in my breathing, such as whether it slows down or speeds up.	☐	☐	☐	☐	☐	☐
IA1_05. I do not notice (I ignore) physical tension or discomfort<br>until they become more severe.	☐	☐	☐	☐	☐	☐
IA1_06. I distract myself from sensations of discomfort.	☐	☐	☐	☐	☐	☐
IA1_07. When I feel pain or discomfort, I try to power through it.	☐	☐	☐	☐	☐	☐
IA1_08. When I feel physical pain, I become upset.	☐	☐	☐	☐	☐	☐
IA1_09. I start to worry that something is wrong if I feel any discomfort.	☐	☐	☐	☐	☐	☐
IA1_10. I can notice an unpleasant body sensation without worrying about it.	☐	☐	☐	☐	☐	☐
IA1_11. I can pay attention to my breath without being distracted by things happening around me.	☐	☐	☐	☐	☐	☐
IA1_12. I can maintain awareness of my inner bodily sensations even when there is a lot going on around me.	☐	☐	☐	☐	☐	☐
IA1_13. When I am in conversation with someone, I can pay attention to my posture.	☐	☐	☐	☐	☐	☐
IA1_14. I can return awareness to my body if I am distracted.	☐	☐	☐	☐	☐	☐
IA1_15. I can refocus my attention from thinking to sensing my body.	☐	☐	☐	☐	☐	☐
IA1_16. I can maintain awareness of my whole body even when a part of me is in pain or discomfort.	☐	☐	☐	☐	☐	☐
IA1_17. I am able to consciously focus on my body as a whole.	☐	☐	☐	☐	☐	☐
IA1_18. I notice how my body changes when I am angry.	☐	☐	☐	☐	☐	☐
IA1_19. When something is wrong in my life I can feel it in my body.	☐	☐	☐	☐	☐	☐
IA1_20. I notice that my body feels different after a peaceful experience.	☐	☐	☐	☐	☐	☐
IA1_21. I notice that my breathing becomes free and easy when I feel comfortable.	☐	☐	☐	☐	☐	☐
IA1_22. I notice how my body changes when I feel happy / joyful.	☐	☐	☐	☐	☐	☐

	0- Never	1	2	3	4	5 - Always
IA1_23. When I feel overwhelmed I can find a calm place inside.	☐	☐	☐	☐	☐	☐
IA1_24. When I bring awareness to my body I feel a sense of calm.	☐	☐	☐	☐	☐	☐
IA1_25. I can use my breath to reduce tension.	☐	☐	☐	☐	☐	☐
IA1_26. When I am caught up in thoughts, I can calm my mind by focusing on my body/breathing.	☐	☐	☐	☐	☐	☐
IA1_27. I listen for information from my body about my emotional state.	☐	☐	☐	☐	☐	☐
IA1_28. When I am upset, I take time to explore how my body feels.	☐	☐	☐	☐	☐	☐
IA1_29. I listen to my body to inform me about what to do.	☐	☐	☐	☐	☐	☐
IA1_30. I am at home in my body.	☐	☐	☐	☐	☐	☐
IA1_31. I feel my body is a safe place.	☐	☐	☐	☐	☐	☐
IA1_32. I trust my body sensations.	☐	☐	☐	☐	☐	☐

(DERS) Please indicate how often the following statements apply to you by checking the box that best describes your experience.

	Almost Never (0-10%)	Sometimes (11-35%)	About Half The Time (36-65%)	Most of the Time (66-90%)	Almost Always (91-100%)
ER1_01. I am clear about my feelings.	☐	☐	☐	☐	☐
ER1_02. I pay attention to how I feel.	☐	☐	☐	☐	☐
ER1_03. I experience my emotions as overwhelming and out of control.	☐	☐	☐	☐	☐
ER1_04. I have no idea how I am feeling.	☐	☐	☐	☐	☐
ER1_05. I have difficulty making sense out of my feelings.	☐	☐	☐	☐	☐
ER1_06. I am attentive to my feelings.	☐	☐	☐	☐	☐
ER1_07. I know exactly how I am feeling.	☐	☐	☐	☐	☐
ER1_08. I care about what I am feeling.	☐	☐	☐	☐	☐
ER1_09. I am confused about how I feel.	☐	☐	☐	☐	☐
ER1_10. When I'm upset, I acknowledge my emotions.	☐	☐	☐	☐	☐
ER1_11. When I'm upset, I become angry with myself for feeling that way.	☐	☐	☐	☐	☐
ER1_12. When I'm upset, I become embarrassed for feeling that way.	☐	☐	☐	☐	☐
ER1_13. When I'm upset, I have difficulty getting work done.	☐	☐	☐	☐	☐
ER1_14. When I'm upset, I become out of control.	☐	☐	☐	☐	☐
ER1_15. When I'm upset, I believe that I will remain that way for a long time.	☐	☐	☐	☐	☐
ER1_16. When I'm upset, I believe that I will end up feeling very depressed.	☐	☐	☐	☐	☐
ER1_17. When I'm upset, I believe that my feelings are valid and important.	☐	☐	☐	☐	☐
ER1_18. When I'm upset, I have difficulty focusing on other things.	☐	☐	☐	☐	☐
ER1_19. When I'm upset, I feel out of control.	☐	☐	☐	☐	☐
ER1_20. When I'm upset, I can still get things done.	☐	☐	☐	☐	☐
ER1_21. When I'm upset, I feel ashamed at myself for feeling that way.	☐	☐	☐	☐	☐
ER1_22. When I'm upset, I know that I can find a way to eventually feel better.	☐	☐	☐	☐	☐

	Almost Never (0-10%)	Sometimes (11-35%)	About Half The Time (36-65%)	Most of the Time (66-90%)	Almost Always (91-100%)
ER1_23. When I'm upset, I feel like I am weak.	☐	☐	☐	☐	☐
ER1_24. When I'm upset, I feel like I can remain in control of my behaviours.	☐	☐	☐	☐	☐
ER1_25. When I'm upset, I feel guilty for feeling that way.	☐	☐	☐	☐	☐
ER1_26. When I'm upset, I have difficulty concentrating.	☐	☐	☐	☐	☐
ER1_27. When I'm upset, I have difficulty controlling my behaviours.	☐	☐	☐	☐	☐
ER1_28. When I'm upset, I believe there is nothing I can do to make myself feel better.	☐	☐	☐	☐	☐
ER1_29. When I'm upset, I become irritated at myself for feeling that way.	☐	☐	☐	☐	☐
ER1_30. When I'm upset, I start to feel very bad about myself.	☐	☐	☐	☐	☐
ER1_31. When I'm upset, I believe that wallowing in it is all I can do.	☐	☐	☐	☐	☐
ER1_32. When I'm upset, I lose control over my behaviour.	☐	☐	☐	☐	☐
ER1_33. When I'm upset, I have difficulty thinking about anything else.	☐	☐	☐	☐	☐
ER1_34. When I'm upset I take time to figure out what I'm really feeling.	☐	☐	☐	☐	☐
ER1_35. When I'm upset, it takes me a long time to feel better.	☐	☐	☐	☐	☐
ER1_36. When I'm upset, my emotions feel overwhelming.	☐	☐	☐	☐	☐

Mindfulness Practice Questions

MP1_01 Think about the last 6 months. During that time, have you practiced mindfulness, either formally or informally, in any way?

No .... 0 (***Skip to MP1_03***)
Yes 1

On average, how minutes PER WEEK do you engage in the following types of mindfulness activities?	Average # Minutes per week	Less than weekly	*Don't Know*	*Prefer not to answer*
MP1_02a Body Scan	____	666	777	888
MP1_02b Yoga	____	666	777	888
MP1_02c Awareness of breath meditation..	____	666	777	888
MP1_02d Sitting Meditation..	____	666	777	888
MP1_02e Walking Meditation..	____	666	777	888
MP1_02f Loving-kindness Meditation..	____	666	777	888
MP1_02g Mountain Meditation..	____	666	777	888
MP1_02h Visual Meditation..	____	666	777	888
MP1_02i Eating Meditation..	____	666	777	888
MP1_02j Meditation moving through regions, such as breath, physical sensations, sound, thoughts, and open awareness	____	666	777	888
MP1_02k Goal-related activity (e.g., physical activity, diet change, etc.). *Please describe*______________________________..................	____	666	777	888
MP1_02l Other mindful activities:<br>Other 1 *describe:* ________________ ....<br>Other 2 *describe:* ________________ ....<br>Other 3 *describe:* ________________	____	666	777	888

MP1_03 Which of the following statements, BEST describes your current attitude towards mindfulness?

I do not plan on practicing mindfulness 1
I see value in mindfulness but do not practice it regularly...........2
I practice mindfulness regularly...3
Other (*please describe* ____________________________)......4
Don't Know 7
Prefer not to answer...8

MP1_04 Which of the following, if any, have you participated in [since completing the course / the last six months]?

None, I have not practiced mindfulness 00

Check ALL that apply

MB-BP Study Regular 1-1.5 hour Booster Sessions.......[ ]
MB-BP Study All Day Retreats.......................................[ ]
Meditation group *not* related the MB-BP Study ....[ ]
Mindful yoga group *not* related the MB-BP Study ....[ ]
Other 1 *describe:* ________________ ....[ ]
Other 2 *describe:* ________________ ....[ ]
Other 3 *describe:* ________________ ....[ ]
Don't Know...77
Prefer not to answer...88

MP1_05 We are interested in the ways in which mindfulness practice may or may not impact your life. Please describe below your relationship to mindfulness since completing your course.

Don't Know 7
Prefer not to answer...8

Please use #2 pencil only.

ID: ______ — __

1. Do you currently take multi-vitamins? (Please report other individual vitamins in the next section.)

- No
- Yes ► a) How many do you take per week? ◯ 2 or less ◯ 3–5 ◯ 6–9 ◯ 10 or more
- b) What specific brand (or equivalency) do you usually take?
  - ◯ Centrum Silver ◯ Centrum ◯ Other ► e.g., AARP Alphabet II Formula 643 Multivitamins and Minerals
  - ◯ Theragran M ◯ One-A-Day Essential

Not counting multi-vitamins, do you take any of the following preparations?

a) Vitamin A	◯ No	◯ Yes, seasonal only ◯ Yes, most months	If Yes,	Dose per day:	◯ Less than 10,000 IU	◯ 10,000 to 15,000 IU	◯ 16,000 to 22,000 IU	◯ 23,000 IU or more	◯ Don't know
b) Potassium	◯ No	◯ Yes	If Yes,	Dose per day:	◯ Less than 2.5 mEq (100 mg)	◯ 3 to 10 mEq	◯ 11 to 20 mEq	◯ 21 mEq or more	◯ Don't know
c) Vitamin C	◯ No	◯ Yes, seasonal only ◯ Yes, most months	If Yes,	Dose per day:	◯ Less than 400 mg	◯ 400 to 700 mg	◯ 750 to 1250 mg	◯ 1300 mg or more	◯ Don't know
d) Vitamin B_6	◯ No	◯ Yes	If Yes,	Dose per day:	◯ Less than 50 mg	◯ 50 to 99 mg	◯ 100 to 149 mg	◯ 150 mg or more	◯ Don't know
e) Vitamin E	◯ No	◯ Yes	If Yes,	Dose per day:	◯ Less than 100 IU	◯ 100 to 250 IU	◯ 300 to 500 IU	◯ 600 IU or more	◯ Don't know
			Type:	◯ Natural	◯ Regular (dl)	◯ Unknown			
f) Calcium (Include Calcium in Tums, etc.)	◯ No	◯ Yes	If Yes,	Dose per day (elemental calcium):	◯ Less than 600 mg	◯ 600 to 900 mg	◯ 901 to 1500 mg	◯ 1501 mg or more	◯ Don't know
g) Selenium	◯ No	◯ Yes	If Yes,	Dose per day:	◯ Less than 80 mcg	◯ 80 to 130 mcg	◯ 140 to 250 mcg	◯ 260 mcg or more	◯ Don't know
h) Vitamin D (In calcium supplement or separately)	◯ No	◯ Yes, seasonal only ◯ Yes, most months	If Yes,	Dose per day:	◯ Less than 300 IU	◯ 300 to 500 IU	◯ 600 to 900 IU	◯ 1000 IU or more	◯ Don't know
i) Zinc	◯ No	◯ Yes	If Yes,	Dose per day:	◯ Less than 25 mg	◯ 25 to 74 mg	◯ 75 to 100 mg	◯ 101 mg or more	◯ Don't know

2. Are there other supplements that you take on a regular basis?

◯ Metamucil/Citrucel	◯ Flax Seed	◯ Niacin	◯ Choline	◯ DHEA
◯ Cod Liver Oil	◯ Beta-carotene	◯ Chromium	◯ Folic Acid	◯ Iron
◯ Vitamin B_{12}	◯ Magnesium	◯ Lecithin	◯ B-Complex	◯ Other (Please specify)
◯ Flax Seed Oil	◯ Fish oil	◯ Coenzyme Q_{10}	◯ Lycopene	

3. How many teaspoons of sugar do you add to your beverages or food each day? ► ____ tsp.

4. What brand and type of cold breakfast cereal do you usually eat? ► Specify cereal brand & type (e.g., Kellogg's Raisin Bran)

◯ Don't eat cold breakfast cereal.

5. What form of margarine or spread do you usually use (exclude pure butter)? ► What specific brand & type of margarine (e.g., Shedd's Country Crock plus calcium and vitamins)

◯ None **Form?** ◯ Stick ◯ Tub ◯ Spray ◯ Squeeze (liquid)

Type? ◯ Reg ◯ Light ◯ Nonfat

6. For each food listed, fill in the circle indicating how often on average you have used the amount specified during the past year.

Ch rb cf sw gn t k w

AVERAGE USE LAST YEAR

DAIRY FOODS		Never, or less than once per month	1–3 per month	1 per week	2–4 per week	5–6 per week	1 per day	2–3 per day	4–5 per day	6+ per day
Milk (8 oz. glass)	Skim milk	◯	◯	Ⓦ	◯	◯	Ⓓ	◯	◯	◯
	1 or 2 % milk	◯	◯	Ⓦ	◯	◯	Ⓓ	◯	◯	◯
	Whole milk	◯	◯	Ⓦ	◯	◯	Ⓓ	◯	◯	◯
	Soy milk	◯	◯	Ⓦ	◯	◯	Ⓓ	◯	◯	◯
Cream, e.g., coffee, whipped or sour cream (1 Tbs)		◯	◯	Ⓦ	◯	◯	Ⓓ	◯	◯	◯
Non-dairy coffee whitener (1 Tbs)		◯	◯	Ⓦ	◯	◯	Ⓓ	◯	◯	◯
Frozen yogurt, sherbet or low-fat ice cream (1 cup)		◯	◯	Ⓦ	◯	◯	Ⓓ	◯	◯	◯
Regular ice cream (1 cup)		◯	◯	Ⓦ	◯	◯	Ⓓ	◯	◯	◯
Yogurt (1 cup)	Low-carb, artificially sweetened or plain	◯	◯	Ⓦ	◯	◯	Ⓓ	◯	◯	◯
	Sweetened–with fruit or other flavoring	◯	◯	Ⓦ	◯	◯	Ⓓ	◯	◯	◯
Spreads added to food or bread; exclude use in cooking	Margarine	◯	◯	Ⓦ	◯	◯	Ⓓ	◯	◯	◯
	Pure Butter	◯	◯	Ⓦ	◯	◯	Ⓓ	◯	◯	◯
Cottage or ricotta cheese (1/2 cup)		◯	◯	Ⓦ	◯	◯	Ⓓ	◯	◯	◯
Cream cheese (1 oz.)		◯	◯	Ⓦ	◯	◯	Ⓓ	◯	◯	◯
Other cheese, e.g., American, cheddar, etc., plain or as part of a dish (1 slice or 1 oz. serving)		◯	◯	Ⓦ	◯	◯	Ⓓ	◯	◯	◯

► **What type of cheese do you usually eat?** ◯ Soy ◯ Regular ◯ Low fat or Lite ◯ Nonfat ◯ None

6. ***(continued)*** **For each food listed, fill in the circle indicating how often on average you have used the amount specified during the past year.**

Please try to average your seasonal use of foods over the entire year. For example, if a food such as cantaloupe is eaten 4 times a week during the approximate 3 months that it is in season, then the average use would be once per week.

FRUITS	Never, or less than once per month	1–3 per month	1 per week	2–4 per week	5–6 per week	1 per day	2–3 per day	4–5 per day	6+ per day
Raisins (1 oz. or small pack) or grapes (1/2 cup)			W			D			
Prunes or dried plums (6 prunes or 1/4 cup)			W			D			
Prune juice (small glass)			W			D			
Bananas (1)			W			D			
Cantaloupe (1/4 melon)			W			D			
Avocado (1/2 fruit or 1/2 cup)			W			D			
Fresh apples or pears (1)			W			D			
Apple juice or cider (small glass)			W			D			
Oranges (1)			W			D			
Orange juice (small glass): Calcium fortified			W			D			
Orange juice (small glass): Regular (not calcium fortified)			W			D			
Grapefruit (1/2) or grapefruit juice (small glass)			W			D			
Other fruit juices (small glass)			W			D			
Strawberries, fresh, frozen or canned (1/2 cup)			W			D			
Blueberries, fresh, frozen or canned (1/2 cup)			W			D			
Peaches or plums (1 fresh or 1/2 cup canned)			W			D			
Apricots (1 fresh, 1/2 cup canned or 5 dried)			W			D			

VEGETABLES	Never, or less than once per month	1–3 per month	1 per week	2–4 per week	5–6 per week	1 per day	2–3 per day	4–5 per day	6+ per day
Tomatoes (2 slices)			W			D			
Tomato or V-8 juice (small glass)			W			D			
Tomato sauce (1/2 cup) e.g., spaghetti sauce			W			D			
Salsa, picante or taco sauce (1/4 cup)			W			D			
String beans (1/2 cup)			W			D			
Beans or lentils, baked, dried or soup (1/2 cup)			W			D			
Tofu, soy burger, soybeans, miso or other soy protein			W			D			
Peas or lima beans (1/2 cup fresh, frozen, canned)			W			D			
Broccoli (1/2 cup)			W			D			
Cauliflower (1/2 cup)			W			D			
Cabbage or coleslaw (1/2 cup)			W			D			
Brussels sprouts (1/2 cup)			W			D			
Carrots, raw (1/2 carrot or 2–4 sticks)			W			D			
Carrots, cooked (1/2 cup) or carrot juice (2–3 oz.)			W			D			
Corn (1 ear or 1/2 cup frozen or canned)			W			D			
Mixed or stir-fry vegetables (1/2 cup), veg. soup (1 cup)			W			D			
Yams or sweet potatoes (1/2 cup)			W			D			
Dark orange (winter) squash (1/2 cup)			W			D			
Eggplant, zucchini or other summer squash (1/2 cup)			W			D			
Kale, mustard greens or chard (1/2 cup)			W			D			
Spinach, cooked (1/2 cup)			W			D			
Spinach, raw as in salad (1 cup)			W			D			
Iceberg or head lettuce (1 serving)			W			D			
Romaine or leaf lettuce (1 serving)			W			D			
Celery (2–3 sticks)			W			D			
Peppers: green, yellow or red (3 slices)			W			D			
Onions as a garnish or in salad (1 slice)			W			D			
Onions as a cooked vegetable, rings or soup (1/2 cup)			W			D			

EGGS, MEAT, ETC.	Never, or less than once per month	1–3 per month	1 per week	2–4 per week	5–6 per week	1 per day	2–3 per day	4–5 per day	6+ per day
Eggs (1): Omega-3 fortified including yolk			W			D			
Eggs (1): Regular eggs including yolk			W			D			
Beef or pork hot dogs (1)			W			D			
Chicken or turkey hot dogs or sausage (1)			W			D			
Chicken/turkey sandwich or frozen dinner			W			D			
Other chicken or turkey, with skin (3 oz.)			W			D			
Other chicken or turkey, without skin (3 oz.)- including ground			W			D			
Bacon (2 slices)			W			D			

6. ***(continued)*** **For each food listed, fill in the circle indicating how often on average you have used the amount specified during the past year.**

EGGS, MEAT, ETC.	Never, or less than once per month	1–3 per month	1 per week	2–4 per week	5–6 per week	1 per day	2–3 per day	4–5 per day	6+ per day
Salami, bologna, or other processed meat sandwiches			W			D			
Other processed meats, e.g., sausage, kielbasa, etc. (2 oz. or 2 small links)			W			D			
Hamburger (1 patty) – Lean or extra lean			W			D			
Hamburger (1 patty) – Regular			W			D			
Beef, pork, or lamb as a sandwich or mixed dish, e.g., stew, casserole, lasagna, frozen dinners, etc.			W			D			
Pork as a main dish, e.g., ham or chops (4–6 oz.)			W			D			
Beef or lamb as a main dish, e.g., steak, roast (4–6 oz.)			W			D			
Canned tuna fish (3–4 oz.)			W			D			
Breaded fish cakes, pieces, or fish sticks (1 serving, store bought)			W			D			
Shrimp, lobster, scallops as a main dish			W			D			
Dark meat fish, e.g., tuna steak, mackerel, salmon, sardines, bluefish, swordfish (3–5 oz.)			W			D			
Other fish, e.g., cod, haddock, halibut (3–5 oz.)			W			D			

BREADS, CEREALS, STARCHES	Never, or less than once per month	1–3 per month	1 per week	2–4 per week	5–6 per week	1 per day	2–3 per day	4–5 per day	6+ per day
Cold breakfast cereal (1 serving)			W			D			
Cooked oatmeal/cooked oat bran (1 cup)			W			D			
Other cooked breakfast cereal (1 cup)			W			D			
Bread (1 slice) – White bread, including pita			W			D			
Bread (1 slice) – Rye/Pumpernickel			W			D			
Bread (1 slice) – Whole wheat, oatmeal, other whole grain			W			D			
Crackers, regular or lowfat e.g., Triscuits, Ritz (6)			W			D			
Bagels, English muffins, or rolls (1)			W			D			
Muffins or biscuits (1)			W			D			
Pancakes or waffles (2 small pieces)			W			D			
Brown rice (1 cup)			W			D			
White rice (1 cup)			W			D			
Pasta, e.g., spaghetti, noodles, couscous, etc. (1 cup)			W			D			
Tortillas (2)			W			D			
French Fries (6 oz. or 1 serving)			W			D			
Potatoes, baked, boiled (1) or mashed (1 cup)			W			D			
Potato chips or corn/tortilla chips (small bag or 1 oz.)			W			D			
Pizza (2 slices)			W			D			

		BEVERAGES	Never, or less than once per month	1–3 per month	1 per week	2–4 per week	5–6 per week	1 per day	2–3 per day	4–5 per day	6+ per day
CARBONATED BEVERAGES. Consider the serving size as 1 glass, bottle or can for these carbonated beverages.	Low-Calorie (sugar-free) types	Low-calorie beverage with caffeine, e.g., Diet Coke, Diet Mt. Dew			W			D			
		Other low-cal bev. without caffeine, e.g., Diet 7-Up			W			D			
	Regular types (not sugar-free)	Carbonated beverage with caffeine & sugar, e.g., Coke, Pepsi, Mt. Dew, Dr. Pepper			W			D			
		Other carbonated beverage with sugar, e.g., 7-Up, Root Beer, Ginger Ale, Caffeine-Free Coke			W			D			
OTHER BEVERAGES		Other sugared beverages: Punch, lemonade, sports drinks, or sugared ice tea (1 glass, bottle, can)			W			D			
		Beer, regular (1 glass, bottle, can)			W			D			
		Light Beer, e.g., Bud Light (1 glass, bottle, can)			W			D			
		Red wine (5 oz. glass)			W			D			
		White wine (5 oz. glass)			W			D			
		Liquor, e.g., vodka, gin, etc. (1 drink or shot)			W			D			
		Water: bottled, sparkling, or tap (8 oz. cup)			W			D			
		Herbal tea or decaffeinated tea (8 oz. cup)			W			D			
		Tea with caffeine (8 oz. cup), including green tea			W			D			
		Decaffeinated coffee (8 oz. cup)			W			D			
		Coffee with caffeine (8 oz. cup)			W			D			
		Dairy coffee drink (hot/cold) e.g., Cappuccino (16 oz.)			W			D			

6. ***(continued)*** **For each food listed, fill in the circle indicating how often on average you have used the amount specified during the past year.**

SWEETS, BAKED GOODS, MISCELLANEOUS		Never, or less than once per month	1–3 per month	1 per week	2–4 per week	5–6 per week	1 per day	2–3 per day	4–5 per day	6+ per day
Milk chocolate (bar or pack), e.g., Hershey's, M&M's				W			D			
Dark chocolate, e.g., Hershey's Dark or Dove Dark				W			D			
Candy bars, e.g., Snickers, Milky Way, Reeses				W			D			
Candy without chocolate (1 oz.)				W			D			
Cookies (1)	Fat free or reduced fat			W			D			
	Other			W			D			
Brownies (1)				W			D			
Doughnuts (1)				W			D			
Cake	Fat free or reduced fat			W			D			
	Other			W			D			
Pie, homemade or ready made (slice)				W			D			
Jams, jellies, preserves, syrup, or honey (1 Tbs)				W			D			
Peanut butter (1 Tbs)				W			D			
Popcorn (3 cups)	Fat free or light			W			D			
	Regular			W			D			
Sweet roll, coffee cake or other pastry (serving)	Fat free or reduced fat			W			D			
	Other			W			D			
Breakfast bars, e.g., Nutrigrain, granola, Kashi (1)				W			D			
Energy bars, e.g., Clif, Luna, Glucerna, Powerbar (1)				W			D			
Low Carb bars, e.g., Atkins, Zone, South Beach (1)				W			D			
Pretzels (1 small bag or serving)				W			D			
Peanuts (small packet or 1 oz.)				W			D			
Walnuts (1 oz.)				W			D			
Other nuts (small packet or 1 oz.)				W			D			
Oat bran, added to food (1 Tbs)				W			D			
Other bran (wheat, etc.), added to food (1 Tbs)				W			D			
Chowder or cream soup (1 cup)				W			D			
Ketchup or red chili sauce (1 Tbs)				W			D			
Splenda (1 packet)				W			D			
Other artificial sweetener (1 packet)				W			D			
Olive oil added to food or bread (1 Tbs)				W			D			
Low-fat or fat-free mayonnaise (1 Tbs)				W			D			
Regular mayonnaise (1 Tbs)				W			D			
Salad dressing (1-2 Tbs)				W			D			

Type of salad dressing: ◯ Nonfat ◯ Low-fat ◯ Olive oil ◯ Other vegetable oil

7. Liver: (beef, calf or pork 4 oz.) ◯ Never ◯ Less than 1/mo ◯ 1/mo ◯ 2–3/mo ◯ 1/week or more
Liver: (chicken or turkey 1 oz.) ◯ Never ◯ Less than 1/mo ◯ 1/mo ◯ 2–3/mo ◯ 1/week or more

8. How often do you eat fried or sautéed food at home? (Exclude "Pam"-type spray)
◯ Less than once a week ◯ 1–3 times per week ◯ 4–6 times per week ◯ Daily

9. What kind of fat is usually used for frying and sautéing at home? (Exclude "Pam"-type spray)
◯ Real butter ◯ Margarine ◯ Olive oil ◯ Vegetable oil ◯ Veg. shortening ◯ Lard ◯ N/A

10. What kind of fat is usually used for baking at home?
◯ Real butter ◯ Margarine ◯ Olive oil ◯ Vegetable oil ◯ Veg. shortening ◯ Lard ◯ N/A

11. What type of cooking oil is usually used at home? (e.g., Mazola Corn Oil) **Specify brand and type** →

12. How often do you eat deep fried chicken, fish, shrimp, clams or onion rings away from home?
◯ Less than once a week ◯ 1–3 times per week ◯ 4–6 times per week ◯ Daily

13. How often do you eat toasted breads, bagel or English muffin (e.g., slice or 1 half bagel)?
◯ Less than once a week ◯ 1–3 times per week ◯ 4–6 times per week ◯ Daily ◯ 2+ times/day

14. Are there any other important foods that you usually eat at least once per week?

Include for example: Applesauce, mushrooms, bulgur, radish, horseradish, Eggbeaters, dates, figs, rhubarb, mango, mixed dried fruit, papaya, wheat germ, custard, venison, hot peppers, pickles, olives, SlimFast, Ensure (regular or plus), Glucerna Shake.

(Do not include dry spices and do not list something that has been listed in the previous sections.)

	Other foods that you usually eat at least once per week	Servings per week
(a)		
(b)		
(c)		

Appendix 4 -

General release form for permission to record mindfulness sessions

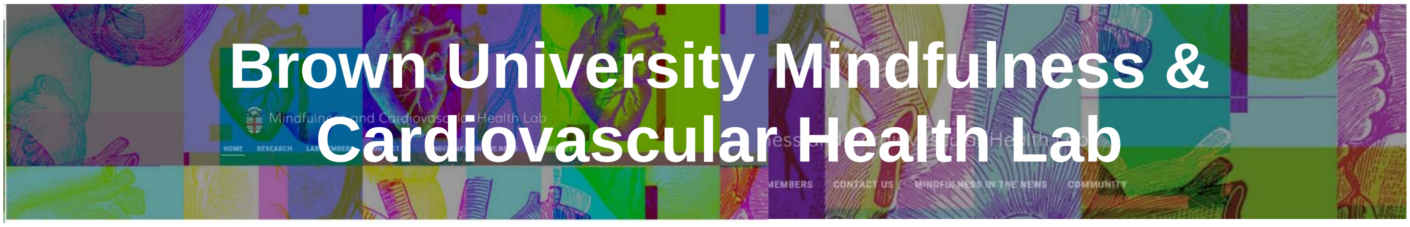

Brown University Mindfulness & Cardiovascular Health Lab

GENERAL RELEASE TO VIDEO RECORD MB-BP CLASSES

We are looking forward to your participation in the upcoming 9-week Mindfulness-Based Blood Pressure Reduction (MB-BP) class being held at the Brown University School of Public Health.

We want to make you aware that the **teacher of the class** may be video recording the classes for training and educational purposes only. Class videotapes may be made available to MB-BP supervisors, teacher trainers, teacher trainees and researchers through the Mindfulness Center at Brown University. It may be used by them for professional education and training purposes via various secured outlets, including a *password-protected and private* YouTube channel, *a password-protected and private* DropBox account, and electronic academic learning software tools. **You will not be videotaped**; however, your voices will be recorded on tape.

Please sign below to indicate that you give permission to the MB-BP instructor to videotape and record you during the administration of the MB-BP program. This release includes the right to record your likeness, voice and activities during class (including any of your property.) For the instructors, their name and likeness will be videotaped and recorded. For participants, effort will be made to omit references to you by name and to capture only your voice and not your image; however, depending on your location in the room, it is possible that your likeness may be briefly videotaped and recorded. It is possible that your first name will be spoken and recorded.

You understand that Brown University will own the program and may use, copy, distribute, display and perform the entirety or portions of the program (including recordings or images of your voice and likeness) in any manner in conjunction with their educational mission. You hereby transfer to Brown University any ownership interest you might otherwise have in any resulting image, photograph, videotape, transcript or recording of the resulting program. You waive any associated claims of invasion of privacy, moral rights, or rights of publicity relating with the MB-BP instructor's use of the program.

You understand that he/she is in no way obligated to use the program and that you will not receive any compensation for the rights granted in this release.

____________________________________ __________________________

Signature Date

____

Printed Name

Appendix 5 -

Additional questions to be asked of control group members at the end of the 6 month in-person follow up assessments

Follow up questions for control group members to be administered at the end of the 6 month in-person follow up assessment

At this time, we would like to take a moment and ask you some questions about your experience as a member of the waitlist control group. In doing so, we are hoping to get your feedback, both positive and negative, on the Mindfulness-Based Blood Pressure Reduction Study as well as find out if you made any changes in your life as a result of enrolling in the study. In answering the questions, please feel free to share your point of view and know that we welcome both positive and negative feedback. There are no right or wrong answers and your participation is optional.

CT01 You first enrolled in the MB-BP program back in [*Month / Year*]. Since that date, have you made any changes to your diet or to your physical activity?

No .... 0 *(Skip to CT02)*
Yes 1

CT01a What changes did you make?

CT01b What do you think prompted you to make those changes?

CT02 We provided you with a blood pressure monitor at the first assessment. Besides the readings we had you take for the study, did you use the blood pressure monitor?

No .... 0 *(Skip to CT03)*
Yes 1

CT02a On average, how often did you take your blood pressure at home over the last six months?

CT02b What changes, if any, have you made to your lifestyle as a result of using the monitor? (If no changes, prompt: What are some of the barriers you experience that hinder making changes to your lifestyle?)

CT03 Since enrolling in the MB-BP Study, have you engaged in any type of mindfulness practice on your own independent of the program?

No .... 0 *(Skip to CT04)*
Yes 1

CT03a What type of mindfulness programs and/or practices have you participated in since enrolling in the study? How often have you engaged in these practices on average?

CT03b What changes, if any, have you made to your lifestyle as a result of mindfulness practices?

CT04 Since enrolling in the study, have you made any other changes in your life as a result of your participation in the mindfulness program that we haven't discussed? For example, have you made any modifications to your blood pressure medication or alcohol intake over the last six months?

No .... 0 *(Skip to CT05)*
Yes 1

CT04a Can you explain those changes?

The last two question are about ways to improve the control group experience.

CT05. As a control group participant it can often be difficult to wait six months for the ability to take the 9-week mindfulness intervention. What was your experience like having to wait the six months? Did you experience any difficulty or frustration in waiting for the program?

CT06. What, if anything, could we have done to improve this experience for you?

BROWN

Amendment Request
Institutional Review Board

Principal Investigator: Eric B. Loucks, PhD

Title: Mindfulness-Based Blood Pressure Reduction (MB-BP) Study (#1412001171)

Date of Request: 12/14/2018

Original Type of Review: ☐ Exempt ☐ Expedited ☒ Full Board

1.) Provide a brief lay summary of the overall project. Include enough detail to allow the IRB to evaluate the requested change(s) within the context of the overall project:
See attached summary

2.) Provide a detailed description of the changes being requested:
See attached summary

3.) State the reason (justification) for the requested amendment:
See attached summary

4.) What is your assessment of how the changes will affect the overall risk/benefit ratio of the study and the willingness of individuals to participate?
See attached summary

5.) New documents / changes to existing documents:

(a) Does the requested amendment require new documents or changes to the approved consent form or other documents?

☒ Consent/assent documents (attach revised version with changes highlighted)
☒ New/revised instruments (attach -if revised, highlight changes)
☐ New/revised advertising materials (attach -if revised, highlight changes)

(b) Do **you** have a significant financial interest (SFI) that is related to this research protocol? "Related" could mean the research involves products, technology, intellectual property, or services made, owned, or provided by the entity/ies in which you have an SFI and/or that the SFI could be affected by the proposed research or its results. ☐ YES ☒ NO

If **YES**, has this conflict been previously disclosed to the IRB?

☐ YES
☐ NO

If **NO**, please describe: Click or tap here to enter text.

6.) Personnel Changes/New Investigator(s):

The *Brown University Conflict of Interest Policy for Officers of Instruction and Research ("COI Policy")* defines the term "Investigator" as "the project director or principal investigator ***and any other person, regardless of title or position*** (e.g., full or part-time faculty member, staff member, student, trainee, collaborator, or consultant), who is **responsible** for the **design, conduct, or reporting** of sponsored research."

Using this definition of "Investigator," have you added any new Brown Investigators to this project since your most recent IRB approval (initial approval, or approval of your most recent progress report or amendment)?

☐ YES ☒ NO (If no, stop here. No need to proceed to the next question.)

7.) New Investigator(s) Conflict of Interest:

Any **new Investigators** (if applicable) must answer the below questions. Please include additional sheets if needed to identify all new Investigators by name and title.

Name of Investigator: Click or tap here to enter text.

Title: Click or tap here to enter text.

(a) Have you completed a conflict of interest disclosure (i.e., *Annual COI Assurance Form* **or** *COI Reporting Form*) within the past 12 months and is it accurate and up-to-date as of the time of this submission, as required by the COI Policy? (You may access the system here to confirm.)

☐ YES ☐ NO

(b) Do you have a significant financial interest (SFI) that is related to this research protocol? "Related" could mean the research involves products, technology, intellectual property, or services made, owned, or provided by the entity/ies in which you have an SFI and/or that the SFI could be affected by the proposed research or its results. ☐ YES ☐ NO

Please note that if any new Investigators have an SFI related to this research protocol, the IRB may require modifications to the informed consent document(s).

If **YES**, please describe: Click or tap here to enter text.

PI Electronic Signature: 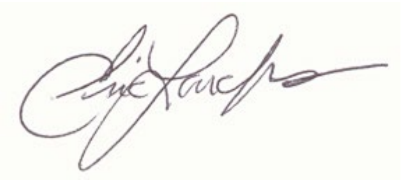

PI Name: Eric B. Loucks, Ph.D.

Date: 12/14/2018

1. **Provide a brief lay summary of the overall project. Include enough detail to allow the IRB to evaluate the requested change(s) within the context of the overall project.**

Original lay summary providing broad overview of the project:

The World Health Organization reported that suboptimal blood pressure (BP) is responsible for more than half of cardiovascular disease mortality world-wide. Furthermore, greater than half of those with hypertension have uncontrolled BP. *A 2009 Institute of Medicine report recommended prioritizing research to "Compare the effectiveness of mindfulness-based interventions (e.g. yoga, meditation, deep breathing training) and usual care in treating… cardiovascular risk factors."* Evidence-based mindfulness interventions, including Mindfulness-Based Stress Reduction, may have some effects on blood pressure, where a recent meta-analysis and systematic review of 4 randomized controlled trials demonstrated significant effects, but evidence of heterogeneity in effect sizes. The methodologically highest quality studies had the smallest effect sizes (range 0-5 mmHg). Mindfulness-Based Stress Reduction (MBSR) has been customized to a number of disease processes, such as Mindfulness-Based Cognitive Therapy for patients with recurrent depression, and Mindfulness-Based Relapse Prevention for patients with substance use addictions. Effect sizes have been increased by customizing mindfulness interventions to diseases of interest. The same may be true for hypertension, *however mindfulness interventions customized for prehypertensive/hypertensive patients have never been investigated.* Until methodologically rigorous studies to evaluate customized interventions for hypertension are performed, we will not know if the observed preliminary effects of general mindfulness interventions on blood pressure reduction could be much more effective with a tailored approach. Consequently, we propose to conduct a stage I behavioral therapy intervention study to evaluate whether MBSR customized to prehypertensive and hypertensive patients has the potential to provide clinically relevant reductions in BP. Consequently the specific aims are:

Stage 1a: Therapy Development/Manual Writing

1. To outline and evaluate key novel elements of mindfulness-based hypertension therapy (MBHT), customized from the evidence-based MBSR. *We hypothesize that the most important novel element will be generation of mindfulness skills specifically applied to hypertension risk factors such as diet, physical activity, obesity, alcohol consumption and antihypertensive medication adherence.* This aim will be achieved using (1) focus groups of participants undergoing the MBHT behavioral intervention, (2) discussion with experts (including cardiologists, epidemiologists, mindfulness experts, mindfulness intervention instructors) prior to, and following pilot testing of MBHT in participants, and (3) clinical judgment of the investigators performing the intervention.
2. To determine effectiveness of MBHT on primary outcomes (systolic blood pressure, retention rates, recruitment rates, and adverse effects) and secondary outcomes (hypertension risk factors such as diet, physical activity, obesity, and antihypertensive medication adherence) in hypertension subgroups, specifically participants with (1) prehypertension, (2) controlled hypertension, and (3) uncontrolled hypertension. Initial decisions about the targeted sample based on hypertension status will be made.
3. To develop an MBHT therapist manual and training program, including procedures for training, supervising, and evaluating therapists. Furthermore, acceptable therapist characteristics will be developed. The manual and training program will include themes such as specification of unique and common elements of MBHT *vs.* other interventions, description of interventions excluded from MBHT, and specification of key treatment parameters such as frequency and duration of treatment, session length, topics addressed, sequence of sessions, as well as therapist adherence and competency measures. The MBHT training will consist of a therapist manual, a formal didactic training seminar, and at least one closely supervised training session.

Stage Ib: Pilot Trial

4. To determine whether a mindfulness-based hypertension therapy (MBHT) intervention, customized from the evidence-based MBSR, has promise to be an effective behavioral therapy for participants with hypertension and/or prehypertension. We will perform a randomized controlled pilot trial for MBHT *vs.* enhanced usual care control. *We hypothesize that MBHT will have adequate recruitment rates (≥10% of prehypertensive/hypertensive participants invited from physicians' offices), fairly low drop out rates (<15%), and medium effect sizes (e.g. 5-10 mmHg systolic BP) for reduction in blood pressure.*

These findings will provide publishable pilot data that will inform future randomized clinical trials that evaluate effects of MBHT on long-term changes in blood pressure *vs.* usual care and active control groups. *If proven effective, MBHT could be offered as a complementary program in the prehypertensive/hypertensive patient population that contributes to over half of the cardiovascular disease mortality world-wide.*

Project update as of December 2018:

The Mindfulness-Based Blood Pressure Reduction (MB-BP) Study, formerly known as Mindfulness-Based Hypertension Therapy (MBHT), is currently in Year 4 of a five year NIH (formerly UH2, now UH3) grant.

Data collection for Stage 1a (single arm clinical trial) of the project has officially ended and the first manuscript is about to be submitted for publication. In total we ran three separate Mindfulness-Based Blood Pressure reduction (MB-BP) intervention courses during this stage of the study, with a total analyzable sample size of 44 participants. There were 48 participants who started the nine-week course; however, five of those individuals withdrew from the intervention prior to completion. One of the five came back into the office for the first follow up assessment and was included in the 'intention to treat' analyses.

In June 2017 (still under NCCIH UH2 funding) we began enrollment for the next phase of the study (referenced as Stage 1b in the lay summary above, but now called 'Stage 2a'), which is to conduct a Randomized Controlled Trial (RCT) with *enhanced usual care*. Data collection for this phase will continue through February 2020. To date, 82 individuals have been enrolled and randomized into Stage 2a.

We were officially approved by NCCIH for UH3 funding of our study in September 2018. This funding will allow us to continue data collection for the Stage 2a RCT with enhanced usual care. However, it is important to note that some of the aims of the study have shifted since the original grant was awarded in September 2015. The primary objectives for UH3 are included in this amendment along with other revisions made to the study protocol for UH3. Approval of this IRB amendment will represent alignment with the currently approved NCCIH UH3 study protocol (v.2.5 – December 11, 2018) and the MB-BP Study Protocol reviewed and approved by Brown University IRB.

2. Provide a detailed description of the changes being requested (Use additional pages, if necessary):

The primary objective of this modification submission is to align the most recent version of the study protocol (v.2.5) approved by the funding agency, the National Center for Complementary and Integrative Health (NCCIH), with the study protocol approved by the Brown University IRB.

As part of the transition to UH3, an official site initiation visit (SIV) was conducted by an external monitoring agency (Westat) on September 13, 2018. The resulting SIV Report was emailed to us on October 9, 2018 by Westat representative, Bert Arevalo. The majority of the revisions to the study protocol outlined in this amendment (#13) came about as a result of the October 9, 2018 SIV Report.

Outlined below are the individual revisions that will need to be made to the Brown University IRB-approved study protocol in order for it to be aligned to the NCCIH study protocol (v.2.5 – December 11, 2018). Per the Brown IRB's request, we are not submitting the actual NCCIH protocol in full at this time. However, if and when the revisions outlined below are approved, the study protocol approved by both the funding agency (NCCIH) and by Brown University will be in sync.

Requested revisions include:

(1) Update of study objectives to align with the currently approved UH3 NCCIH Study Protocol (v.2.5).

> *Related Attachment - Appendix A: MB-BP Study Objectives for UH3AT009145, directly taken from NCCIH approved study protocol (v.2.5 – December 11, 2018, p.12)*

(2) Update of study measures and procedures to align with UH3 NCCIH Study Protocol (v.2.5). We are requesting permission to add only one new measure (i.e., medication use). This measure had previously been included in our study protocol but was removed with Amendment #8; at this time, we would like to add it back in. The other revisions we are requesting involve removing and reordering measures. See Appendix B for a complete list of the NCCIH approved measures found in v.2.5 of the study protocol.

> *Related Attachment - Appendix B: Complete list of measures and study procedures taken directly from the NCCIH approved Study Protocol (v.2.5 – December 11, 2018). Note that this document has been annotated to assist in review. All the study procedures and measures listed in this document have already been approved by the Brown IRB unless otherwise noted with track changes comment bubbles.*

Below we have outlined in more detail the requested revisions to our study measures and assessment questionnaires to achieve alignment with the NCCIH approved study protocol (v.2.5). Specifically, we are requesting permission to:

a) *Add back in the previously removed question block on medication history*, which was removed from our study protocol in Brown IRB Amendment #8 (approved 6/12/17) but previous to that point had been approved by the Brown University IRB. The external monitor at the SIV recommended for safety monitoring and for analytical purposes that we should add back in the collection of medications. Note that these questions are administered at each of the in-person assessments, so that we can capture prescribed and over the counter medication use throughout the course of study involvement. Other than this one addition, there are no other new measures being added to our study protocol.

Related Attachments - Appendix C: Previously approved medication question block. See also Appendices D and E for marked up in-person assessment questionnaires using track changes feature.

b) *Remove and reorder selected measures* - In order to reduce participant burden, we have removed the following measures from the home baseline survey: the introduction and personal goal questions; the Cognitive Emotion Regulation Questionnaire (CERQ-18); and the Readiness to change for hypertensive risk factors questions. The Mindful Attention Awareness Scale (MAAS) has also been removed as a measure; it was taken out of the in-person baseline assessment. Note that these items were able to be removed without a large impact on the analyses since they are not part of the primary or secondary outcomes and since we already have sufficient data from UH2 Stage 1 and Stage 2a using these measures.

   In addition to removing items, we have also moved a few items around in order to improve the survey flow and to reduce the overall length of the home survey. The changes include: (a) moving the Multidimensional Assessment of Interoceptive Awareness (MAIA), the Perceived Stress Scale (PSS-14), and the Difficulties in Emotion Regulation Scale (DERS) from the home baseline questionnaire to the in-person baseline assessment; and (b) moving race-ethnicity questions and questions on blood pressure from the home baseline survey to the in-person screener.

   Note that all revisions (i.e., deletions and reordering of measures) have been noted directly in the attached questionnaires using the track changes feature.

   Related Attachments – Appendix D: Revised in-person screener (v.3.0 – track changes); Appendix E: Revised in-person baseline assessment (v.3.0 – track changes); and Appendix F: Revised online home baseline* survey (v.3.0 - track changes)*

   **For clarification – although called the 'baseline' assessments, both of these documents represent not only the baseline measures but also those administered at follow up.*

(3) New UH3 Data and Safety Monitoring Plan (DSMP) – As part of the UH3 phase of the MB-BP study a new NCCIH DSMP was created. Study PI (Loucks) and research staff consulted with data and safety monitoring expert, Willoughby Britton, on how best to monitor and report adverse events when conducting research studies implementing mindfulness-based interventions. The result of this consultation was the creation of a new and improved UH3 DSMP, one that meets the CONSORT Harms Criteria.[1]

To expedite the review of the UH3 DSMP (v.1.0 – November 14, 2018), we have included both a clean version as well as an annotated version with comments highlighting the major areas of change and rationale behind the revisions.

Related Attachments – Appendix G: NCCIH Data and Safety Monitoring Plan (DSMP) – clean version (v.1.0 – November 14, 2018 – NEW for UH3); Appendix H: NCCIH Data and Safety Monitoring Plan (DSMP) – annotated version

(4) Updated informed consent form (v.3.2 – December 14, 2018) – the informed consent form was updated to include additional language around potential risks related to meditation, the addition of medication use questions, and shifting in time allocation for the research assessments. Finally, on the bottom of p.3 of the consent, we have added a new study requirement. We are asking that all participants randomly assigned

to the waitlist control group refrain from "initiating and engaging in mindfulness practices and formal meditation during the six-month period of [their] participation in the research study." This is a new component to UH3. Although we would never remove someone from the actual study if they choose to engage in mindfulness, we may end up having to exclude his/her data from the analyses. To avoid this, we will be reviewing this request with all participants during the informed consent process prior to enrollment.

Related Attachments – Appendix I: Revised UH3 Stage 2a MB-BP Informed Consent Form (v.3.2 - December 13, 2018) – track changes

3. State the reason (justification) for the requested amendment:

Justification for all revisions is provided above.

4. What is your assessment of how the changes will affect the overall risk/benefit ratio of the study and the willingness of individuals to participate?

We have taken a number of steps towards reducing participant burden and more effectively monitoring participant safety that are all outlined in this amendment. This includes creating a new and revised Data and Safety Monitoring Plan (DSMP) for UH3, improving survey flow, as well as shortening the overall home baseline survey length. Many of these revisions were implemented based on participant feedback during the UH2 phase of the study.

5. Does the requested amendment require new documents or changes to the approved consent form or other documents?

See enclosed attachments related to this amendment:

Appendix A - MB-BP Study Objectives for UH3AT009145 directly taken from NCCIH approved study protocol (v.2.5 – December 11, 2018, p.12)

Appendix B – Complete list of measures and study procedures taken directly from the NCCIH approved Study Protocol (v.2.5 – December 11, 2018, Section 6) – *annotated to denote revisions*

Appendix C – Previously approved medication question block

Appendix D – Revised in-person screener (v.3.0) – *with track changes*

Appendix E – Revised in-person baseline assessment (v.3.0) – *with track changes*

Appendix F – Revised home baseline questionnaire (v.3.0) – *with track changes*

Appendix G – NCCIH Data and Safety Monitoring Plan (DSMP) – *full version, clean (v.1.0 – November 14, 2018 – NEW for UH3)*

Appendix H - NCCIH Data and Safety Monitoring Plan (DSMP) v.1.0 – *annotated version to highlight updates to UH3*

Appendix I - Revised informed consent form (v.3.2 – December 13, 2018) – *with track changes*

REFERENCES:

1. Ioannidis JP, Evans SJ, Gotzsche PC, et al. Better reporting of harms in randomized trials: an extension of the CONSORT statement. Annals of internal medicine. 2004;141(10):781-788.

Appendix A – MB-BP Study Objectives for UH3AT009145

Directly taken from NCCIH approved study protocol

(v.2.5 – December 11, 2018, p.12)

1. STUDY OBJECTIVES

1.1 Primary Objective

1. ***Impacts of MB-BP on Primary Self-Regulation Targets:*** Identify the impacts of MB-BP *vs*. enhanced usual care on the primary self-regulation target, specifically an assay of self-related processes (MAIA) described in Table 1. We hypothesize that MB-BP will significantly improve the MAIA in directions of better self-regulation, compared to control.
    a. Secondary analyses will evaluate impacts on secondary self-regulation targets including an assay of emotion regulation (DERS), and cognitive processes (SART), described in Table 1.
    b. Exploratory analyses will evaluate engagement of MB-BP *vs*. enhanced usual care with triangulated self-regulation target assays described in **Table 1** such as emotion regulation and stress (Pittsburgh Stress Battery, Perceived Stress Scale), self-related processes (Heart Beat Detection Task, Interoceptive Awareness fMRI Task), and cognitive processes (Mindful Attention Awareness Scale). Measures such as the neuroimaging Interoceptive Awareness fMRI Task will replicate assays in the MINDFUL-PC study.
2. ***Self-Regulation Targets as Mediators of MB-BP Effect on Medical Regimen Adherence and Health Behavior Change:*** Evaluate the degree to which the engagement of MB-BP with self-regulation targets translates into improved prehypertension/hypertension medical regimen adherence, specifically for the Dietary Approaches to Stop Hypertension (DASH)-consistent diet. We hypothesize that MB-BP will increase the DASH diet score compared to control, in participants with low DASH diet adherence at baseline (DASH diet score <5.5), and that the self-regulation primary outcomes in Aim 1 are significant mediators.
3. ***Further develop an MB-BP therapist manual and training program, including procedures for training, supervising, and evaluating therapists.*** The PI will implement training he receives from the University of Bangor in the United Kingdom in May 2018 to implement the Mindfulness-Based Interventions Teacher Assessment Criteria (MBI-TAC) for MB-BP instructors, which is the most respected quantitative and qualitative tool developed to provide feedback for enhancing MBI teacher effectiveness, and establishing teacher certification.[1-3]

Appendix B – Complete list of measures and study procedures taken directly from the NCCIH approved Study Protocol (v.2.5 – December 11, 2018, Section 6)

Annotated to denote revisions being requested

Excerpt from NCCIH approved Study Protocol (v.2.5 – December 11, 2018)

6. STUDY PROCEDURE (annotated)

6.1 Schedule of Evaluations

Variables Measured	Assessment Times			
	Baseline	10 weeks	6 months	1 year*
Demographics	X			
Family history of hypertension	X			
Childhood Socioeconomic Status	X			
Adverse Childhood Experiences	X			
Depressive Symptomatology	X	X	X	
Anxiety	X	X	X	
Medication Use	X	X	X	X
Anti-Hypertensive Medication Adherence	X	X	X	
Blood pressure	X	X	X	X
Anthropometry	X	X	X	X
Physical Activity	X	X	X	
Diet	X	X	X	X
Alcohol consumption	X	X	X	X
Cigarette Smoking	X	X	X	
Sleep Duration	X	X	X	X
Mindfulness	X	X	X	
Mindfulness home practice	X	X	X	X
Emotional Eating	X	X	X	
Self-Compassion	X	X	X	
Perceived Stress	X	X	X	
Emotional Regulation	X	X	X	X
Interoception	X	X	X	X
Heartbeat Detection	X	X	X	
Decentering	X	X	X	
Attention Control	X	X	X	
Craving for Hypertensive Risk Factors	X	X	X	
Social Integration	X	X	X	
Loneliness	X	X	X	
PROMIS Global Health	X	X	X	
Self-Control	X	X	X	
Resilience	X	X	X	
Self-efficacy for managing chronic disease	X	X	X	
Stress Reactivity	X	X	X	
Delay Discounting	X	X	X	
Semi-structured exit interviews at 6 months			X	

**1-year assessments consist of a subset of the other two follow ups and will be completed only with intervention group as controls are offered course post 6-month follow-up*

6.2 Description of Evaluations

6.2.1 Screening Evaluation and Consenting Procedure

Please see **Appendices C and D** for the consenting process prior to the phone screening, and prior to the in-person screening.

Final screening evaluations will occur at least one week prior to baseline assessments. Baseline assessments will occur within 4 weeks of intervention initiation.

Phone-Based Screening: For people who indicate interest in the study, this screening will take place by phone using trained research assistants to assess the exclusion criteria described above, with the exception of blood pressure which will be assessed in-person

In-Person Screening: If participants remain eligible after the phone-based screening, they will attend an in-person screening for blood pressure and medication assessment. If mean blood pressure is elevated (≥120 mmHg systolic and/or ≥80 mmHg diastolic pressure), participants will be invited to return for a second blood pressure reading. At that time, if the mean blood pressure across both assessment times is ≥120 mmHg systolic or ≥80 mmHg diastolic pressure, they will be invited to participate in the study.

6.2.2 Enrollment

The enrollment date is the day that the individual has met all the screening criteria, signs the informed consent form, and confirms agreement to participate in the study.

6.2.3 Baseline Assessments

(1) *Demographics:* age, race/ethnicity, socioeconomic status (education, employment), and household structure.
(2) *Family History of Hypertension (FH):* Assesses biological parents' history of having hypertension, based on questions from New England Family Study LEAP Project.
(3) *Childhood socioecononomic status:* retrospective reporting of parents' education, based on standardized questionnaires used in the Atherosclerosis Risk in Communities (ARIC) study.
(4) *Adverse Childhood Experiences:* Measured using the standardized Childhood Trauma Questionnaire (CTQ), Childhood food insecurity questionnaire, and the Childhood Experiences of Care and Abuse Inventory neglect subscale. [184,195-200]
(5) *Depressive symptomatology:* Assessed using Center for Epidemiologic Studies Depression Scale Revised (CESD-R). The CESD survey has been used extensively in the epidemiologic literature to assess depressive symptomatology.[201] The scale was updated to the CESD-R by Van Dam *et al.*, which allows diagnosable criteria similar to Diagnostic and Statistical Manual (DSM) of Mental Disorders.[202]
(6) *Anxiety:* Assessed using the validated Beck Anxiety Inventory.[203-209]
(7) *Medication use:* Assessed directly from participants' medication bottles and self-report using standardized forms, including medication name, dose, frequency of use, and reason of use.
(8) *Antihypertensive medication adherence:* measured continuously using electronic medication bottle caps (eCAPS, Ottawa, Canada).[185]
(9) *Systolic and diastolic blood pressure:* Clinical blood pressure will be measured

using a calibrated Omron HEM-705CPN following American Heart Association and Joint National Committee (JNC) guidelines.[183,210,211] Additionally, participants will be asked to complete three at home blood pressure readings using a validated home blood pressure monitor (Omron, Model PB786N) provided at baseline.

(10) *Anthropometry:* height and weight directly assessed using standard epidemiologic methods.[186]

(11) *Physical activity:* The International Physical Activity Questionnaire which has undergone substantial validity and reliability testing.[187-189] Adherence to Joint National Commission-7 (JNC-7) guidelines is 30 min aerobic physical activity ≥4 days per week.[183]

(12) *Diet*: assessed utilizing the validated Food Frequency that allows for calculation of hypertension-related dietary factors, including salt intake, alcohol consumption, total caloric consumption, fruit and vegetable consumption, and Dietary Approaches to Stop Hypertension (DASH) eating pattern score.[182]

(13) *Alcohol consumption:* additional self-report standardized questions assessing current alcohol consumption taken from the Behavioral Risk Factor Surveillance Survey (BRFSS).[212]

(14) *Cigarette smoking:* current smoking assessed using self-report standardized questions from the New England Family Study.

(15) *Sleep duration:* Sleep duration is assessed using a single question on sleep duration from the validated Pittsburgh Sleep Quality Index (PSQI).[213-215]

(16) *Mindfulness:* Assessed using the validated Five Facet Mindfulness Questionnaire.[216]

(17) *Mindfulness Home Practice:* questions capturing individuals at home mindfulness practice pre- and post- intervention are administered at each of the time points.

(18) *Emotional eating:* measured using the Three Factor Eating Questionnaire Revised 21-item (TFEQ-R21).[217,218]

(19) *Self-compassion:* Assessed using the validated Self-Compassion Scale Short Form (SCS-SF).[121]

(20) *Perceived stress:* Assessed using the validated 14-item Perceived Stress Scale.[219,220]

(21) *Emotion regulation:* Measured using the validated Difficulties in Emotion Regulation Scale.[221]

(22) *Interoception:* Assessed directly using the Heartbeat Detection Task and in secondary self-report using the validated Multidimensional Assessment of Interoceptive Awareness (MAIA).[222-225]

(23) *Decentering:* Assessed using the validated Experiences Questionnaire.[226,227]

(24) *Attention control:* Assessed using the Sustained Attention to Response Task (SART). The SART is a validated computerized test of sustained attention, response inhibition (executive function) and self-regulation.[228-230 231-233]

(25) *Craving:* craving for hypertension risk factors, including palatable foods, alcohol, and sedentary activities will be assessed using the validated Craving Experiences Questionnaire. [234]

(26) *Social integration:* Measured using the validated 12-item Interpersonal Support Evaluation List (ISEL-12) measure of social support.[235]

(27) *Loneliness:* Assessed using the validated R-UCLA Loneliness Scale.[236]

(28) *Global Health:* individual physical, mental and social health are measured using the validated NIH PROMIS Global Health v1.2 scale.[237]

(29) *Self-control:* assessed using the validated Self-Control Scale short form.[238,239]

(30) *Resilience:* measured using the validated 10-item Connor-Davidson Resilience

Scale (CD-RISC-10).[240]

(31) *Self-efficacy for chronic disease management:* measured using the 6-item Self-efficacy for Managing Chronic Disease scale (SECD-6).[241]

(32) *Stress Reactivity:* Assessed using the Pittsburg Stress Battery a standardized protocol of 3 computerized tasks designed to induce a stress response indicated by evaluated cardiovascular (CV) reactivity.[242]

(33) *Delayed Discounting:* Assessed using the validated 5-Trial Adjusting Delay Discounting Task. [243]

(34) *Functional Magnetic Resonance Imaging (fMRI):* Participants in the UH3 phase who elect to take part in the fMRI imaging study will undergo an fMRI scanning session for approximately 40 minutes at baseline and 10-week follow-up. Scans will be acquired with a 3T scanner while the subject is in the resting state. Final sample size will be 24 per group (n=48); enrolling up to 60 participants in total to reach target sample size. Participants will undergo a separate informed consent and screening process for the fMRI imaging, so that they can be in the MB-BP study without imaging if they prefer. Refer to the separate fMRI study protocol for details.

6.2.4 Randomization and Intervention Allocation

Randomization will occur following the completion of baseline assessments prior to the initiation of study intervention. Only individuals who complete the baseline assessments and indicate they are available for the upcoming courses will be included in the randomization process.

Stratified randomization will be used, as simple randomization can fail if it creates groups unbalanced for critical features known to affect outcomes.[7,8] Stratified randomization can reduce both types I and II error, improve trial efficiency, and facilitate subgroup and interim analyses.[7] Variables used to create strata include age (≤60 *vs.* >60 years), gender (male *vs.* female), and/or uncontrolled hypertension (≥140 mmHg systolic pressure, or ≥90 mmHg diastolic pressure) *vs*. prehypertension (120 to <140 mmHg systolic pressure, and 80 to <90 mmHg diastolic pressure). Simple random sampling will occur within each of the eight strata, thus allowing for each arm of the study to be more balanced with respect to age, gender, and hypertension category. The total size of each stratum will vary from cohort to cohort. To conduct the randomization, a list of participant IDs and strata characteristics will be provided, and a trained researcher not affiliated with the study will perform the randomization on participants after baseline assessment and final determination of eligibility is complete. Randomization will be done using an online computer software program known as Research Randomizer (Version 4.0).[9] The randomization process will occur after each new round of enrollment (i.e., unique cohort). If the cohort sample size is such that there are not enough participants for balance within the three strata, we will prioritize stratification by two key strata instead of three, specifically by baseline blood pressure status and gender. Furthermore, after randomization within strata, if sample sizes in the entire sample for cohort differ by more than one participant per group, then all imbalanced groups are re-randomized until the entire sample differs by no more than 1 participant per group (e.g. n=8 MB-BP, n=9 enhanced usual care control). Expected cohort sizes, consistent with the SARP are 15-20 participants. We anticipate needing to run 6 to 7 cohorts to reach the target sample size.

Study enrollment will continue until target enrollment goals are reached (i.e., n=50 for MBBP intervention group and n=50 for enhanced usual care control group).

6.2.5 Blinding

All study staff will be blinded to the participant treatment allocation with the exception of the instructor, individual who performs the randomization, and staff member coordinating participants within each course. All staff performing participant assessments will be blinded to the participant treatment allocation to promote equipoise. Data analyses will be performed by a statistician blinded to treatment allocation type. The data manager will be able to break blinding if needed (e.g. for Data and Safety Monitoring Board). Circumstances for breaking the blind would be a large number of adverse experiences (>10% of enrolled participants reporting AEs rated as severe or life threatening) taking place in one or more study group. In this case, the data safety monitoring board would be notified, and could break the blind to help determine the cause of the adverse experiences.

6.2.6 Follow-up Visits

Follow-up assessments will be scheduled and conducted within the pre-defined assessment windows outlined previously in section 3, study design. Questionnaires and assessments administered at 10 weeks and 6-months follow-up are identical to those administered at the first in-person screening assessment and at baseline, with the exception that questionnaires for which the answers should not change or be informative (age, race/ethnicity, education, adverse childhood experiences, family history of hypertension) are not given at follow-ups. In addition, adverse events are monitored and documented at each of the follow-up periods as well as throughout the duration of an individual’s study involvement according to the data safety monitoring protocol put forth in this grant. Participants are also asked a set of semi-structured questions at the end of their six month follow up that inquire about their experience as either a control group member or intervention group participant.

6.2.7 Completion/Final Evaluation

For control group participants, the 6-month evaluation is the final visit. Upon completion of this assessment, they will be eligible to participate in the MB-BP training. Individuals in the intervention group will be assessed at 1 year follow up with a subset of measures (see Table 6.1 Schedule of Evaluation) in order to assess long term effects of the intervention.

Appendix C – Previously approved medication question block and medication data collection form

Administered at each of the in-person assessments

Medications (ME)

ME01. Do you take any prescription medications or over-the-counter drugs?

- ☐ No → *skip to end of medications questions*
- ☐ Yes
- ☐ Don't know
- ☐ Prefer not to answer

If you brought your medications with you, please take them out now as we will use them to complete the next section.

ME01b INTERVIEWER CHECKPOINT - Please select one:

- ☐ Rx info not known or available – *skip to end of ME questions; will need to follow up*
- ☐ Ppt brought medications -- *continue*
- ☐ Ppt did not bring medications but knows info – *continue, may need follow up*

ME01c In total how many different medications and/or over the counter drugs do you currently take? Again, do not count vitamins or supplements. _________ # of meds

(REPEAT QUESTION BLOCK BELOW FOR ALL MEDICATIONS)

ME02a. What is the name of the first prescription medication or over-the-counter drug that you take?

☐ Label product name:

____

☐ Label generic name:

____

☐ Don't know ☐ Prefer not to answer

ME02b. What is the dosage form?

Oral
- ☐ Pill, tablet, or capsule
- ☐ Sublingual or orally-disintegrating tablet
- ☐ Liquid solution or suspension (drink, syrup)
- ☐ Powder

Topical
- ☐ Liquid, cream, gel, or ointment
- ☐ Ear drops (otic)
- ☐ Eye drops (ophthalmic)
- ☐ Skin patch (transdermal)

Inhaled
- ☐ Inhaler or nebulizer

Injected
- ☐ Injection

Suppository
- ☐ Rectal (e.g., enema)
- ☐ Vaginal (e.g., douche, pessary)

Other:
- ☐ Don't know
- ☐ Prefer not to answer

ME02c. How frequently do you take it?

- ☐ _________ times per day
- ☐ _________ times per week
- ☐ _________ times per month
- ☐ Don’t know
- ☐ Prefer not to answer

ME02d. What is the strength? *(Record strength of how it is actually taken, not how it is prescribed.)*

- ☐ _________ %
- ☐ _________ mg
- ☐ _________ mcg
- ☐ _________ grams
- ☐ _________ I.U.
- ☐ _________ Other unit: _____________
- ☐ Don’t know
- ☐ Prefer not to answer

ME02f. Do you take it regularly or only as needed?

- ☐ Regularly
- ☐ Only as needed
- ☐ Don’t know
- ☐ Prefer not to answer

ME02g. For how long have you been taking it?

- ☐ For _________ days
- ☐ For _________ weeks
- ☐ For _________ months
- ☐ For _________ years
- ☐ Don’t know
- ☐ Prefer not to answer

ME02h. What is the medication used for?

____

ME02i. *Interviewer comments*:

____

ME02j. Do you take any other prescription medications or over-the-counter drugs?

- ☐ No → *skip to end of medications questions*
- ☐ Yes
- ☐ Don’t know
- ☐ refer not to answer

REPEAT QUESTION BLOCK UNTIL ALL CURRENT MEDICATIONS ARE CAPTURED

Appendix D – Revised In-Person Screener

(v.3.0 – December 13, 2018)

with Track Changes highlighting revisions

IN-PERSON SCREENING ASSESSMENT FORM

The following measures are found in this assessment:

- Systolic and diastolic blood pressure
- Anthropometry – directly assessed height and weight
- Date of birth / age or participant at time of screening
- Gender
- Race-ethncity
- Family history of hypertension
- Medication use

Commented [SF1]: NO CHANGE IN THESE MEASURES

Commented [SF2]: MOVED here from the home survey. Still self-report

Commented [SF3]: Added this back in. It was previously approved by the Brown IRB but was removed under Amendment #8. NCCIH has asked us to add it back in.

Deleted: Version 2.0, April 14, 2017

PID. Participant ID # __________
BA01. Staff ID # __________
BA02. Today's date (MMDDYY): ___________

Blood Pressure:

BA03a. Blood pressure 1st reading, systolic blood pressure: _________ mmHg
BA03b. Blood pressure 1st reading, diastolic blood pressure: __________ mmHg
BA03c. Blood pressure 2nd reading, systolic blood pressure: _________ mmHg
BA03d. Blood pressure 2nd reading, diastolic blood pressure: __________ mmHg
BA03e. Blood pressure 3rd reading, systolic blood pressure: _________ mmHg
BA03f. Blood pressure 3rd reading, diastolic blood pressure: __________ mmHg

BA04. Were the 2nd and 3rd systolic blood pressure readings within 20 mmHg of each other?
□ Yes
□ No ***(repeat measurements)***

BA05. Were the 2nd and 3rd diastolic blood pressure readings within 10 mmHg of each other?
□ Yes
□ No ***(repeat measurements)***

BA06a. Repeated blood pressure 1st reading, systolic blood pressure: _________ mmHg
BA06b. Repeated blood pressure 1st reading, diastolic blood pressure: __________ mmHg
BA06c. Repeated blood pressure 2nd reading, systolic blood pressure: _________ mmHg
BA06d. Repeated blood pressure 2nd reading, diastolic blood pressure: _________ mmHg
BA06e. Repeated blood pressure 3rd reading, systolic blood pressure: _________ mmHg
BA06f. Repeated blood pressure 3rd reading, diastolic blood pressure: __________ mmHg

Blood Pressure Safety Protocol – The out-of-range blood pressure values are as follows: systolic blood pressure >200 mmHg or <90 mmHg; diastolic blood pressure >110 mmHg.

In absence of symptoms (chest pain, shortness of breath, dizziness, headache), for a SBP>200 or DBP>110 or both, we will strongly encourage participants to see their doctor right away or to go to urgent care. If there are symptoms, we will immediately call 911.

In absence of symptoms (chest pain, shortness of breath, dizziness, passing out), for a SBP<90, we will strongly encourage participants to see their doctor right away or to go to urgent care. If there are symptoms, we will immediately call 911.

BA07. Blood pressure cuff size used: □ S □ Reg □ L □ XL

BA08. Arm that cuff was placed on: □ L □ R

BP monitor BP monitor used:
❍ Unit #1 - HEM-705CP (1)
❍ Unit #2 - HEM-705CP (2)
❍ Other (specify: (3) ___________________

Deleted: Version 2.0, April 14, 2017

ELIGIBILITY OUTCOME - based on the average of the 2nd and 3rd BP (repeat) readings, note the participant's eligibility below and read the corresponding script:

Not Eligible: mean systolic BP < 120mmHg AND mean diastolic BP < 80mmHg

Unfortunately, at this time you are not eligible for the study. *If participant is interested, we can keep his/her contact information on file for future studies. END ASSESSMENT.*

Eligible: mean systolic BP ≥ 120mmHg OR mean diastolic BP ≥ 80mmHg

Congratulations, you are eligible for the study! At this time we would like to take your height and weight, ask a few follow up questions and go over the next steps of the study. *Note that if the participant does not wish to continue, thank the participant for his/her time and then END THE ASSESSMENT. Otherwise, continue below.*

We are now going to take your height and weight. *Have ppt remove his/her shoes and empty his/her pockets. Remove bulky clothing as well.*

Commented [SF4]: Added direction for RA to read.

BA09. Height: ______ . __ cm (one decimal place)

BA10. Weight: ______ . __ lbs (one decimal place)

At this time, we will have you complete a few questions on the computer. (*Turn the computer screen towards the participant and have them complete.*)

Commented [SF5]: Added transition sentence for self-report section.

BA11. What is your date of birth? _____ / _____/ _____ [*mm/dd/yyyy*]

BA12. How old are you?: ______ years

BA13. *Are you…* □ Male □ Female □ Other (*please specify: ____________)?*

BQ1_02. Are you Latino or Hispanic?

Commented [SF6]: Race-ethnicity – moved here from home survey. Still self-reported.

□ No → ***skip to BQ3***
□ Yes
□ I do not know
□ I prefer not to answer

Deleted: Version 2.0, April 14, 2017

B1_02a. Which of the following represents your family's country of origin? ***(check all that apply)***

- □ Cuba
- □ Mexico
- □ Puerto Rico
- □ Spain
- □ South America
- □ Columbia
- □ Dominican Republic
- □ Other Central American
- □ Other: ______________________
- □ I do not know
- □ I prefer not to answer

BQ1_03. If you were asked to put yourself into only one of these groups, in which one would you place yourself? ***(select <u>one</u> only):***

- □ Asian
- □ Pacific Islander
- □ African American/Black
- □ Caucasian/White
- □ Native American
- □ Other ___________________
- □ I do not know
- □ I prefer not to answer

<u>Blood Pressure and Blood Pressure Medication Use</u>

Commented [SF7]: Moved here from the home survey. Questions previously approved.

The following questions are about blood pressure.

BP1_1. Does your blood pressure tend to be HIGHER when you have it measured in a clinical setting, such as a doctor's office? This is sometimes called "white coat hypertension."

- □ No
- □ Yes → **SKIP TO FH1_01**
- □ I do not know
- □ I prefer not to answer

BP1_2. Does your blood pressure tend to be LOWER when you have it measured in a clinical setting, such as a doctor's office? This is sometimes called "masked hypertension."

- □ No
- □ Yes
- □ I do not know
- □ I prefer not to answer

Deleted: Version 2.0, April 14, 2017

Family History of Hypertension

Commented [SF8]: Moved here from the home survey. Questions previously approved.

FH1_01. Did your biological mother ever have hypertension?

□ No
□ Yes
□ I do not know
□ I prefer not to answer

FH1_02. Did your biological father ever have hypertension?

□ No
□ Yes
□ I do not know
□ I prefer not to answer

FH1_03. How many full brothers and sisters do you have ***(Please include any brothers or sisters who may have died, but do not include half or step brothers and sisters).***

□ I do not have any brothers or sisters → ***Skip to ME01***

□ ENTER # ______brothers, #______ sisters

□ I do not know
□ I prefer not to answer

FH1_04. Of these brothers and sisters, how many have ever had hypertension?

________ ***(if none, write 0)***
□ I do not know
□ I prefer not to answer

Thank you for completing the self-report questions. At this time please notify the research assistant that you have completed this section.

Deleted: Version 2.0, April 14, 2017

Medications (ME)

The final section asks about medications.

ME01. Do you take any prescription medications or over-the-counter drugs?

- ☐ No → *skip to end of medications questions*
- ☐ Yes
- ☐ Don't know
- ☐ Prefer not to answer

If you brought your medications with you, please take them out now as we will use them to complete the next section.

ME01b INTERVIEWER CHECKPOINT - Please select one:

- ☐ Rx info not known or available – ***skip to end of ME questions; will need to follow up***
- ☐ Ppt brought medications -- ***continue***
- ☐ Ppt did not bring medications but knows info – ***continue, may need follow up***

ME01c In total how many different medications and/or over the counter drugs do you currently take? Again, do not count vitamins or supplements. _________ # of meds

(REPEAT QUESTION BLOCK BELOW FOR ALL MEDICATIONS)

ME02a.What is the name of the first prescription medication or over-the-counter drug that you take?

☐ Label product name: ____

☐ Label generic name: ____

☐ Don't know ☐ Prefer not to answer

ME02b.What is the dosage form?

Oral
- ☐ Pill, tablet, or capsule
- ☐ Sublingual or orally-disintegrating tablet
- ☐ Liquid solution or suspension (drink, syrup)
- ☐ Powder

Topical
- ☐ Liquid, cream, gel, or ointment
- ☐ Ear drops (otic)
- ☐ Eye drops (ophthalmic)
- ☐ Skin patch (transdermal)

Inhaled
- ☐ Inhaler or nebulizer

Injected
- ☐ Injection

Suppository
- ☐ Rectal (e.g., enema)
- ☐ Vaginal (e.g., douche, pessary)

Other:
- ☐ Don't know
- ☐ Prefer not to answer

Commented [SF9]: This section was previously approved by the Brown IRB but was removed under Amendment #8. NCCIH has asked us to add it back in.

Deleted: Version 2.0, April 14, 2017

ME02c.How frequently do you take it?

- ☐ _________ times per day
- ☐ _________ times per week
- ☐ _________ times per month
- ☐ Don't know
- ☐ Prefer not to answer

ME02d.What is the strength? *(Record strength of how it is actually taken, not how it is prescribed.)*

- ☐ _________ %
- ☐ _________ mg
- ☐ _________ mcg
- ☐ _________ grams
- ☐ _________ I.U.
- ☐ _________ Other unit: _____________
- ☐ Don't know
- ☐ Prefer not to answer

ME02f. Do you take it regularly or only as needed?

- ☐ Regularly
- ☐ Only as needed
- ☐ Don't know
- ☐ Prefer not to answer

ME02g.For how long have you been taking it?

- ☐ For _________ days
- ☐ For _________ weeks
- ☐ For _________ months
- ☐ For _________ years
- ☐ Don't know
- ☐ Prefer not to answer

ME02h. What is the medication used for?

____

ME02i. *Interviewer comments*:
____

ME02j. <u>Do you take any other prescription medications or over-the-counter drugs?</u>

- ☐ No → *skip to end of medications questions*
- ☐ Yes
- ☐ Don't know
- ☐ refer not to answer

REPEAT QUESTION BLOCK UNTIL ALL CURRENT MEDICATIONS ARE CAPTURED

Deleted: Version 2.0, April 14, 2017

BA14. *Does the participant currently take any prescription medications to reduce blood pressure?*

□ Yes – *briefly go over the eCAP protocol, including mentioning that the equipment will be given to participant at baseline*

□ No – ***ASK ME01 –***

ME01a Have you ever taken medication for high blood pressure?

☐ No → ***skip to end of medications questions***
☐ Yes → ***skip to end of medications questions***

BA14a Have you made any changes to your blood pressure medication in the past two weeks? (YES / NO) ***If no, skip to end.***

BA14b Describe changes to BP medication below (e.g., timing and description of change) and use this information when scheduling the baseline assessment:

Go over the next phase of the study, including discussing the eCAPs (if applicable). Before participant leaves, schedule the in-person baseline assessment (must be within 1-4 weeks of the scheduled intervention) or have the Project Coordinator follow up to schedule.

END ASSESSMENT

Deleted: 2.0, April 14, 2017

Appendix E – Revised In-Person Baseline* Assessment (v.3.0 – December 13, 2018)

with Track Changes highlighting revisions

IN-PERSON BASELINE*

QUESTIONNAIRE AND ASSESSMENT FORMS

* Questionnaires administered at follow ups are the same as baseline minus any measures that are not subject to change over time (e.g., race-ethnicity, childhood experiences, etc.)

The following measures are found in the in-person baseline assessment:

- Systolic and diastolic blood pressure
- Anthropometry – directly assessed weight (height captured previously)
- Medication use (updated medication history since last assessment)
- Antihypertensive medication adherence - electronic bottle cap (eCAP) (if applicable)
- Depressive symptomology (CESD-R) and Anxiety (BAI)
- Stress reactivity as measured by the Pittsburgh Stress Battery
- Diet / Food Frequency Questionnaire (FFQ)
- PROMIS Global Health v.1.2
- Sleep duration
- Physical activity (IPAQ-long)
- Interoception (MAIA)
- Emotion Regulation (DERS)
- Interoception direct measure - Heart beat dection task
- Pecerived Stress Scale (PSS-14)
- Delayed discounting task
- Attention control direct measure (SART)

Commented [SF1]: This is not NEW. Was added as a clarifying statement.

Commented [SF2]: Unless otherwise stated, these measures have not been changed and have already been approved by the Brown IRB.

Commented [SF3]: Previously approved. Looking to add medication use back into our protocol after having removed it (Amend. #8)

Commented [SF4]: The MAIA and DERS were both moved here from the home baseline survey. Intended to replace the FFMQ and MAAS, so the total administration time does not change.

Commented [SF5]: Also moved here from home baseline survey.

PID. Participant ID # __________
BA01. Staff ID # __________
BA02. Today's date (MMDDYY): ___________

Blood Pressure:
BA03a. Blood pressure 1st reading, systolic blood pressure: _________ mmHg
BA03b. Blood pressure 1st reading, diastolic blood pressure: __________ mmHg
BA03c. Blood pressure 2nd reading, systolic blood pressure: _________ mmHg
BA03d. Blood pressure 2nd reading, diastolic blood pressure: __________ mmHg
BA03e. Blood pressure 3rd reading, systolic blood pressure: _________ mmHg
BA03f. Blood pressure 3rd reading, diastolic blood pressure: __________ mmHg

If the difference of the 2nd and 3rd systolic BP reading is 20 mmHg or greater OR the difference of the 2nd and 3rd diastolic reading is 10 mmHg or greater, then repeat the BP readings. Otherwise, skip to BA07.

BA06a. Repeated blood pressure 1st reading, systolic blood pressure: _________ mmHg
BA06b. Repeated blood pressure 1st reading, diastolic blood pressure: __________ mmHg
BA06c. Repeated blood pressure 2nd reading, systolic blood pressure: _________ mmHg
BA06d. Repeated blood pressure 2nd reading, diastolic blood pressure: __________ mmHg
BA06e. Repeated blood pressure 3rd reading, systolic blood pressure: _________ mmHg
BA06f. Repeated blood pressure 3rd reading, diastolic blood pressure: __________ mmHg

Blood Pressure Safety Protocol – The out-of-range blood pressure values are as follows: systolic blood pressure >200 mmHg or <90 mmHg; diastolic blood pressure >110 mmHg.

In absence of symptoms (chest pain, shortness of breath, dizziness, headache), for a SBP>200 or DBP>110 or both, we will strongly encourage participants to see their doctor right away or to go to urgent care. If there are symptoms, we will immediately call 911.

In absence of symptoms (chest pain, shortness of breath, dizziness, passing out), for a SBP<90, we will strongly encourage participants to see their doctor right away or to go to urgent care. If there are symptoms, we will immediately call 911.

Follow Safety Protocol for Uncontrolled Hypertension (140/90 mmHg or greater)

BA07. Blood pressure cuff size used: □ S □ Reg □ L □ XL
BA08. Arm that cuff was placed on: □ L □ R

BP monitor BP monitor used:

- ❍ Unit #1 - HEM-705CP (1)
- ❍ Unit #2 - HEM-705CP (2)
- ❍ Other (specify: (3) ____________________

We are now going to take your weight. *Have ppt remove his/her shoes and empty his/her pockets. Remove bulky clothing as well.*
BA10. Weight: ______ . __ lb (one decimal place)

Medications (ME)

The next section asks about medications.

ME.change1 At the time we last met with you, you reported….

Review most recently reported medication list and detail with the study participant (i.e., name, strength, dosage, etc.)

Have you made any changes to your medications since that date (e.g., added or removed medications, changed dosage, frequency of use, etc.)?

- ☐ No → *skip to end of medications questions*
- ☐ Yes (Use medication question block to capture updated medication list)

Commented [SF6]: These questions were previously approved; we are seeking permission to add them back in.

Questions for Participants to Answer on Their Own In-Person:
For each statement, please place a mark in the column that best describes how you have been feeling.

	Not at all or less than 1 day last week	1 or 2 days last week	3 to 4 days last week	5 to 7 days last week	Nearly every day for two weeks
DS_1. My appetite was poor.	☐	☐	☐	☐	☐
DS_2. I could not shake off the blues.	☐	☐	☐	☐	☐
DS_3. I had trouble keeping my mind on what I was doing.	☐	☐	☐	☐	☐
DS_4. I felt depressed.	☐	☐	☐	☐	☐
DS_5. My sleep was restless.	☐	☐	☐	☐	☐
DS_6. I felt sad.	☐	☐	☐	☐	☐
DS_7. I could not get going.	☐	☐	☐	☐	☐
DS_8. Nothing made me happy.	☐	☐	☐	☐	☐
C_9. I felt like a bad person.	☐	☐	☐	☐	☐
DS_10. I lost interest in my usual activities.	☐	☐	☐	☐	☐
DS_11. I slept much more than usual.	☐	☐	☐	☐	☐
DS_12. I felt like I was moving too slowly.	☐	☐	☐	☐	☐
DS_13. I felt fidgety.	☐	☐	☐	☐	☐
DS_14. I wished I were dead.	☐	☐	☐	☐	☐
DS_15. I wanted to hurt myself.	☐	☐	☐	☐	☐
DS_16. I was tired all the time.	☐	☐	☐	☐	☐
DS_17. I did not like myself.	☐	☐	☐	☐	☐
DS_18. I lost a lot of weight without trying to.	☐	☐	☐	☐	☐
DS_19. I had a lot of trouble getting to sleep.	☐	☐	☐	☐	☐
DS_20. I could not focus on the important things.	☐	☐	☐	☐	☐

Below is a list of common symptoms of anxiety. Please carefully read each item in the list. Indicate how much you have been bothered by that symptom during the past month, including today, by marking the box in the corresponding space in the column next to each symptom.

	Not At All	Mildly – it didn't bother me much	Moderately – it wasn't pleasant at all times	Severely – it bothered me a lot
BE_1. Numbness or tingling	☐	☐	☐	☐
BE_2. Feeling hot	☐	☐	☐	☐
BE _3. Wobbliness in legs	☐	☐	☐	☐
BE _4. Unable to relax	☐	☐	☐	☐
BE _5. Fear of worst happening	☐	☐	☐	☐
BE _6. Dizzy or lightheaded	☐	☐	☐	☐
BE _7. Heart pounding/racing	☐	☐	☐	☐
BE _8. Unsteady	☐	☐	☐	☐
BE _9. Terrified or afraid	☐	☐	☐	☐
BE _10. Nervous	☐	☐	☐	☐
BE _11. Feeling of choking	☐	☐	☐	☐
BE _12. Hands trembling	☐	☐	☐	☐
BE _13. Shaky/unsteady	☐	☐	☐	☐
BE _14. Fear of losing control	☐	☐	☐	☐
BE _15. Difficulty in breathing	☐	☐	☐	☐
BE _16. Fear of dying	☐	☐	☐	☐
BE _17. Scared	☐	☐	☐	☐
BE _18. Indigestion	☐	☐	☐	☐
BE _19. Faint/lightheaded	☐	☐	☐	☐
BE _20. Face flushed	☐	☐	☐	☐
BE _21. Hot/cold sweats	☐	☐	☐	☐

Pittsburgh Stress Battery: Set up and Overview

Commented [SF7]: No change to the stress test.

"During the next part of the assessment you will be asked to complete a series of three activities, each averaging around 4-6 minutes. These tests may be difficult for you to complete and may results in feelings of stress or anxiety. We are interested in how your body responds to stress both during the tests and after the stressors are encountered. For this reason, we will be monitoring your blood pressure and heart rate before, during and after the three activities. Let us know at any point if you need to take a break. Your participation in these activities is voluntary."

SETUP:

1. Have the participant sit in the assessment chair facing the table.
2. Place the appropriate blood pressure cuff on the participant's non-dominant arm and turn the monitor away from the participant and towards yourself.
3. Set up the study laptop for the first of the three activities and then read the script below.

"To begin, I am going to take your baseline blood pressure and heart rate."

4. Record the participant's blood pressure and heart rate (Reading #1) in the data table associated with the participant.
5. Keep the blood pressure cuff on the participant for the entire battery of stress tests. Move on to the first of the three stress tests below.

Pittsburgh Stress Battery: Stress Activity #1 STROOP

"The first activity we are going to have you do is a color naming task. In this task you will see one word in the middle of the screen and four words along the bottom. You will be asked to choose the word along the bottom that names the color that the center word is printed in."

"The task can be difficult because you have to focus on the COLOR in which the center word is printed in and not on what the word reads. Here is an example:"

[REFER TO SHOW CARD #1, POINT TO IMAGE #1]

"In this example, you would select the first of the four words on the bottom - that is "red" [POINT TO RED] ***since the color of the word at the top is printed in the color RED."***

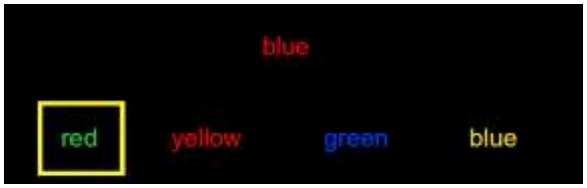

[NOW POINT TO IMAGE #2]
"Here is how we would like you to place your hand on the keyboard." [DEMONSTRATE FOR THE PPT ON THE KEYBOARD AND BY USING IMAGE #2]

"The buttons under each finger are for the four color name selections. Your index finger is on button 1, your middle finger is on button 2, ring finger on button 3 and pinky on button 4." (Note that this order would be reversed for left handed individuals.)

[STILL REFERING TO IMAGE #2]
"In the example here in Image #2, would you press button 1 (index finger), button 2 (middle finger), button 3 (ring finger) or button 4 (pinky finger)?"

[ALLOW PPT TO RESPOND BEFORE PROVIDING ANSWER]
Answer = button 2 (middle finger)

[If the participant answers, incorrectly, move on to Image #3 and ask:] ***"Let's try one more. In Image #3, which button would you press?"***

[ALLOW PPT TO RESPOND BEFORE PROVIDING ANSWER]
Answer = button 1 (index finger)

"We are almost ready to begin. While you are completing the task, we are going to turn on an audio recording that provides addition interference for the task. The entire activity takes 4 minutes to complete. We will be taking a blood pressure reading in the middle of the activity. Please continue to work on the task during the blood pressure reading. Remember to always focus on the color of the middle word and not on what the word reads. Ready?"

SET UP:

1. On study laptop, open up the ePrime software and navigate to the STROOP file.
2. Enter the PID and session number [Baseline=1; 10wk=2; 6mo=3; 12mo=4].
3. Before beginning, use the iPad to set up the timer and to open up the audio interference file.

"When I say 'begin', please hit the 'Enter' button on the keyboard. Make sure to keep your hand in the correct position on numbers 1-4."

"Ready? Begin. "

4. As soon as the ppt begins, make sure to turn on the audio interference file and start the timer.
5. At minute #2 of the test, take the participant's blood pressure and heart rate (Reading #2) and record the readings in the data table associated with the participant.
6. Once the test is complete, move the data file associated with the ppt into the correct data folder.
7. Move on to the next stress activity.

Pittsburgh Stress Battery: Stress Activity #2 Mental Math

Case Math – Mental Math Tool

https://www.preplounge.com/en/mental-math.php

INSTRUCTIONS:

1. Open up the Case Math website (overview page) on the study laptop and read the directions below before beginning.

   "You will now be asked to complete a series of mental math problems using an online program known as Case Math. To start you will be given 60 seconds to complete as many basic arithmetic problems as you can. For each problem you answer correctly, you will be given an additional two seconds of time. Answer the questions as quickly and as accurately as you are able. You will be evaluated both on your speed and your accuracy. Any questions?"

   [ANSWER QUESTIONS AND THEN USE SHOW CARD #2 FOR NEXT SCRIPT]

   "To begin, click the blue 'Start' button found in the bottom left corner under Random-Easy. The test will start right away. Solve the problems in your head as you are able. Use the keyboard on the laptop to enter your answer and then click 'Enter' to submit. Remember that you are being evaluated on both speed and accuracy."

2. Instruct the participant to complete the first Trial.
3. Once complete, record the results in the stress battery data table associated with this participant.
4. Review the results. If the participant scored 60% or greater, then have him/her move on to the "Random-Medium" level for Trial 2. Otherwise, repeat "Random-Easy" for Trial 2.
5. To start the next trial, navigate back to the 'overview' page and read the following:

   "We are now going to have you complete the exercise again. Remember that you are being evaluated on both your speed and accuracy. For this trial, please select the 'Random-[EASY/MEDIUM]' level. Click start when ready."

6. Have the participant complete Trial 2.
7. Record the results of the second math test in the table and then navigate back to the overview grid.
8. For the last trial, again increase the difficulty level if they scored 60% or greater. Otherwise make no change in difficulty. Read the following:
9. ***"You will now repeat this exercise, but during this trial we will take your heart rate and blood pressure. Please try to keep your arm with the cuff as still as possible and still attempt to do your best on the math test. For this trial, please select the 'Random-[EASY/MEDIUM/HARD]' level. Click start when ready."***
10. Once the final trial is initiated, proceed to take the BP and HR reading (Reading #3). Record the blood pressure, heart rate, and math results in the data table.

Pittsburgh Stress Battery: Stress Activity #3 Mirror Tracing

INSTRUCTIONS:

"For the last activity, you will complete four online mirror-tracing trials.

1. Open up the Mirror Tracing Qualtrics survey on the study laptop and enter the PID, cohort, and assessment info.
2. Refer to Show Card 3 and read the directions below:

   "There will be four trials to this test. In each trial, you will see two rectangular panels. The "drawing panel" is at the bottom; this is where you will move the mouse cursor. The "mirror panel" is at the top, this will record your mouse movements in mirrored format as you try to trace the figure.

   To begin a trial, move the mouse so that your cursor rests over the green circle in the drawing panel and then click the left mouse button to signal that you are ready to begin.

   Once the trial begins, a red target circle appears in the mirror panel. Trace the figure working from the starting point towards the red target circle. The trial ends automatically when you reach the red circle.

   When your mouse trail is red, you are within the lines and are earning points. When your mouse trail is blue you are outside the line and are not earning points. Your score is shown at the top of the drawing panel.

   Your score is based on the percentage of tracing made within the lines, so simply drawing straight to the red target will not earn a high score. Please try your best to earn a good score.

   You will have 45 seconds to complete each trial. You will hear an audio warning indicating when time is almost up. When 45 seconds have passed, you will be instructed to immediately move your cursor to the red dot to end the trial, regardless of whether or not you completed the tracing task. Any questions?"

3. Answer questions and then cue up the audio timer. ***"When you are ready, you may click to the next screen to begin."***
4. For all four trials, start the audio timer as soon as the participant clicks on the green circle. Stop the recording as soon as the trial is complete.
5. AFTER TRIAL 3 BUT BEFORE TRIAL 4 READ – ***"For the last trial, I will be taking your blood pressure and heart rate during the test. As much as you can, try not to move during the reading. Focus on completing the task quickly and accurately."***
6. As soon as the participant clicks the green circle in Trial 4, start the audio timer as well as the Blood Pressure cuff (Blood Pressure / Heart Rate Reading #4). Record readings on the data sheet.
7. Once complete, have the participant review his/her scores and then close the Qualtrics survey.

Pittsburgh Stress Battery

SHOW CARD #1 – STROOP TEST

Image #1 -

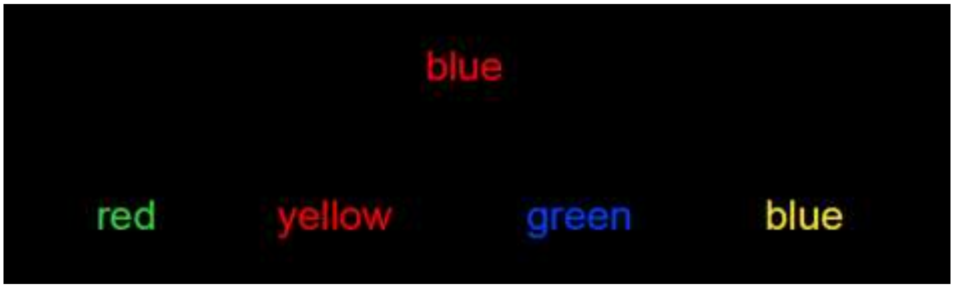

Image #2 -

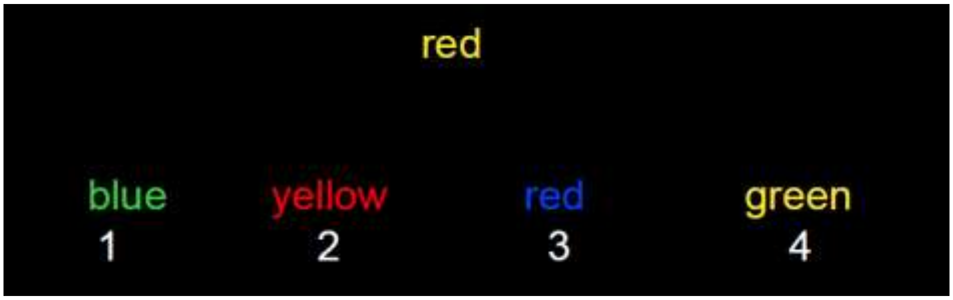

Image #3 -

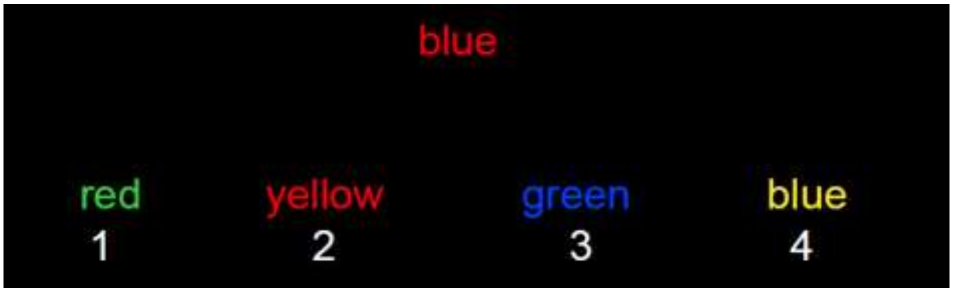

Pittsburgh Stress Battery

SHOW CARD #2 – MENTAL MATH TEST

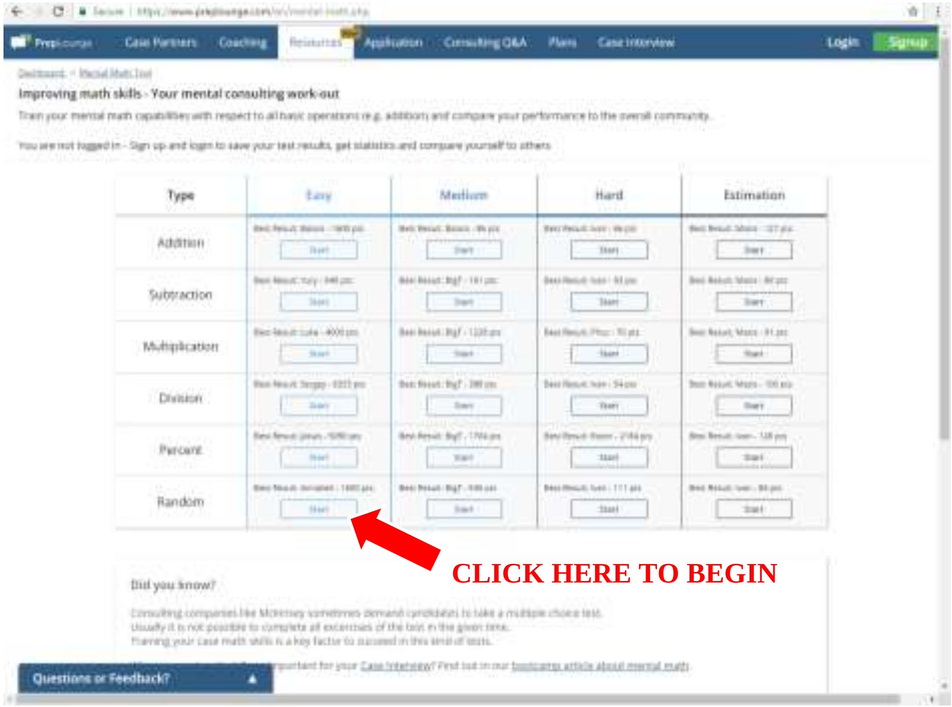

Pittsburgh Stress Battery

SHOW CARD #3 – Mirror Tracing Task

Image 1:

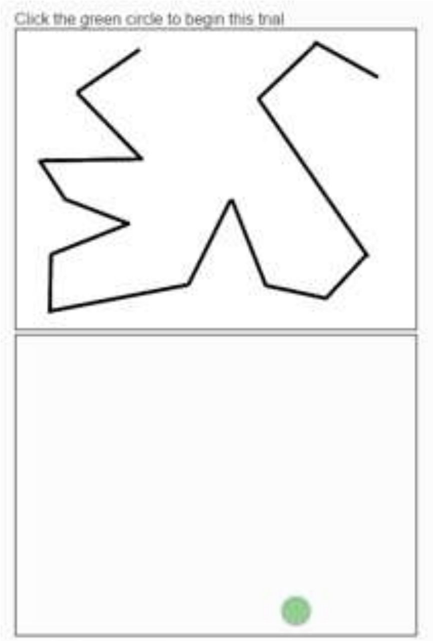

Image 2:

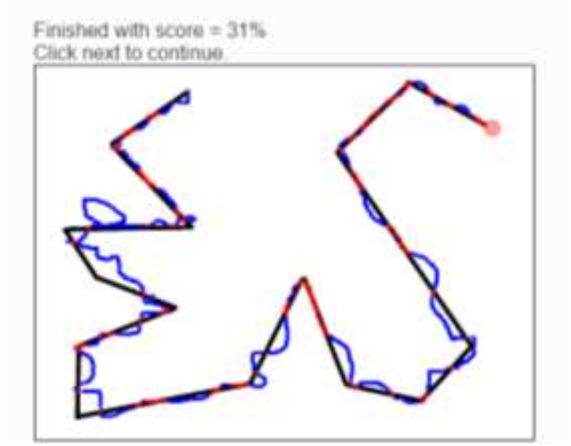

Pittsburgh Stress Battery – 30 MINUTE RECOVERY PERIOD

RA Script to be read to participants:

Great. You are all set with the stress tests. You can now go ahead and relax while I set up the next part of the study. One of the things we are interested in is how individuals recover from stress. While you are relaxing we are going to have you complete a few simple forms. While you complete the forms we will be taking your blood pressure and heart rate three more times – now, 15 minutes later, and 30 minutes later.

*Set up the participant with the below self-report forms. Before he/she begins, take the first of three recovery period readings (*Reading #5*). Start timer. Take two more readings at 15 minutes (*Reading #6*) and then 30 minutes (*Reading #7*).*

SELF REPORT FORMS TO BE COMPLETED DURING RECOVERY PERIOD:

FOOD FREQUENCY QUESTIONNAIRE: The first form we will have you complete is a Food Frequency Questionnaire that will ask you about the types of foods and drinks that you consume. It should take around 20 minutes to complete. Please let me know if you have any questions.

PROMIS Global Health Scale v.1.2 – *standardized scale (found on proceeding pages)*

SLEEP – *1 question only taken from the Pittsburgh Sleep Quality Index*

International Physical Activity Questionnaire (IPAQ) – *standardized scale (found on proceeding pages)*

Multidemsional Assessment of Interoceptive Awareness (MAIA) - *standardized scale (found on proceeding pages)*

Difficulties in Emotion Regulation (DERS) – *standardized scale (found on proceeding pages)*

Commented [SF8]: Removed the MAAS to reduce overall participant burden.

Moved the MAIA and the DERS measures here from the home survey to shorten the home survey.

Moved the FFMQ to the home survey as it is not a primary outcome.

Deleted: Mindful Attention Awareness Scale (MAAS) - *standardized scale (found on proceeding pages)¶*

Deleted: Five Facet Mindfulness Questionnaire (FFMQ) - *standardized scale (found on proceeding pages)*

Global Health

Please respond to each question or statement by marking one box per row.

		Excellent	Very good	Good	Fair	Poor
Global01	In general, would you say your health is:	☐ 5	☐ 4	☐ 3	☐ 2	☐ 1
Global02	In general, would you say your quality of life is:...	☐ 5	☐ 4	☐ 3	☐ 2	☐ 1
Global03	In general, how would you rate your physical health?	☐ 5	☐ 4	☐ 3	☐ 2	☐ 1
Global04	In general, how would you rate your mental health, including your mood and your ability to think?	☐ 5	☐ 4	☐ 3	☐ 2	☐ 1
Global05	In general, how would you rate your satisfaction with your social activities and relationships?	☐ 5	☐ 4	☐ 3	☐ 2	☐ 1
Global09r	In general, please rate how well you carry out your usual social activities and roles. (This includes activities at home, at work and in your community, and responsibilities as a parent, child, spouse, employee, friend, etc.)......	☐ 5	☐ 4	☐ 3	☐ 2	☐ 1

		Completely	Mostly	Moderately	A little	Not at all
Global06	To what extent are you able to carry out your everyday physical activities such as walking, climbing stairs, carrying groceries, or moving a chair?	☐ 5	☐ 4	☐ 3	☐ 2	☐ 1

© 2010-2016 PROMIS Health Organization and PROMIS Cooperative Group

	In the past 7 days…	Never	Rarely	Sometimes	Often	Always
Global10r	How often have you been bothered by emotional problems such as feeling anxious, depressed or irritable?	☐ 5	☐ 4	☐ 3	☐ 2	☐ 1
		None	Mild	Moderate	Severe	Very severe
Global08r	How would you rate your fatigue on average?	☐ 5	☐ 4	☐ 3	☐ 2	☐ 1

Global07r	How would you rate your pain on average?	☐ 0 No pain	☐ 1	☐ 2	☐ 3	☐ 4	☐ 5	☐ 6	☐ 7	☐ 8	☐ 9	☐ 10 Worst pain imaginable

SLEEP - The following question relates to your usual sleep habits during the past month only. Your answer should indicate the most accurate reply for the majority of days and nights in the past month.

SL1_04. **During the past month**, how many hours of actual sleep did you get on average at night? (This may be different than the number of hours you spent in bed.)

AVERAGE HOURS OF SL1_EEP PER NIGHT ___________

□ I do not know
□ I prefer not to answer

INTERNATIONAL PHYSICAL ACTIVITY QUESTIONNAIRE

We are interested in finding out about the kinds of physical activities that people do as part of their everyday lives. The questions will ask you about the time you spent being physically active in the **last 7 days**. Please answer each question even if you do not consider yourself to be an active person. Please think about the activities you do at work, as part of your house and yard work, to get from place to place, and in your spare time for recreation, exercise or sport.

Think about all the **vigorous** and **moderate** activities that you did in the **last 7 days**. **Vigorous** physical activities refer to activities that take hard physical effort and make you breathe much harder than normal. **Moderate** activities refer to activities that take moderate physical effort and make you breathe somewhat harder than normal.

PART 1: JOB-RELATED PHYSICAL ACTIVITY

The first section is about your work. This includes paid jobs, farming, volunteer work, course work, and any other unpaid work that you did outside your home. Do not include unpaid work you might do around your home, like housework, yard work, general maintenance, and caring for your family. These are asked in Part 3.

1. Do you currently have a job or do any unpaid work outside your home?

☐ *Yes*

☐ No → ***Skip to PART 2: TRANSPORTATION***

The next questions are about all the physical activity you did in the **last 7 days** as part of your paid or unpaid work. This does not include traveling to and from work.

2. During the **last 7 days**, on how many days did you do **vigorous** physical activities like heavy lifting, digging, heavy construction, or climbing up stairs **as part of your work**? Think about only those physical activities that you did for at least 10 minutes at a time.

_____ **days per week**

☐ No vigorous job-related physical activity → ***Skip to question 4***

3. How much time did you usually spend on one of those days doing **vigorous** physical activities as part of your work?

_____ **hours per day**
_____ **minutes per day**

4. Again, think about only those physical activities that you did for at least 10 minutes at a time. During the **last 7 days**, on how many days did you do **moderate** physical activities like carrying light loads **as part of your work**? Please do not include walking.

_____ **days per week**

☐ No moderate job-related physical activity → ***Skip to question 6***

5. How much time did you usually spend on one of those days doing **moderate** physical activities as part of your work?

_____ **hours per day**
_____ **minutes per day**

6. During the **last 7 days**, on how many days did you **walk** for at least 10 minutes at a time **as part of your work**? Please do not count any walking you did to travel to or from work.

_____ **days per week**

☐ No job-related walking → ***Skip to PART 2: TRANSPORTATION***

7. How much time did you usually spend on one of those days **walking** as part of your work?

_____ **hours per day**
_____ **minutes per day**

PART 2: TRANSPORTATION PHYSICAL ACTIVITY

These questions are about how you traveled from place to place, including to places like work, stores, movies, and so on.

8. During the **last 7 days**, on how many days did you **travel in a motor vehicle** like a train, bus, car, or tram?

_____ **days per week**

☐ No traveling in a motor vehicle → ***Skip to question 10***

9. How much time did you usually spend on one of those days **traveling** in a train, bus, car, tram, or other kind of motor vehicle?

_____ **hours per day**
_____ **minutes per day**

Now think only about the **bicycling** and **walking** you might have done to travel to and from work, to do errands, or to go from place to place.

10. During the **last 7 days**, on how many days did you **bicycle** for at least 10 minutes at a time to go **from place to place**?

_____ **days per week**

☐ No bicycling from place to place → ***Skip to question 12***

11. How much time did you usually spend on one of those days to **bicycle** from place to place?

_____ **hours per day**
_____ **minutes per day**

12. During the **last 7 days**, on how many days did you **walk** for at least 10 minutes at a time to go **from place to place**?

_____ **days per week**

☐ No walking from place to place → ***Skip to PART 3: HOUSEWORK, HOUSE MAINTENANCE, AND CARING FOR FAMILY***

13. How much time did you usually spend on one of those days walking from place to place?

_____ **hours per day**
_____ **minutes per day**

PART 3: HOUSEWORK, HOUSE MAINTENANCE, AND CARING FOR FAMILY

This section is about some of the physical activities you might have done in the **last 7 days** in and around your home, like housework, gardening, yard work, general maintenance work, and caring for your family.

14. Think about only those physical activities that you did for at least 10 minutes at a time. During the **last 7 days**, on how many days did you do **vigorous** physical activities like heavy lifting, chopping wood, shoveling snow, or digging **in the garden or yard**?

_____ **days per week**

☐ No vigorous activity in garden or yard → ***Skip to question 16***

15. How much time did you usually spend on one of those days doing **vigorous** physical activities in the garden or yard?

_____ **hours per day**
_____ **minutes per day**

16. Again, think about only those physical activities that you did for at least 10 minutes at a time. During the **last 7 days**, on how many days did you do **moderate** activities like carrying light loads, sweeping, washing windows, and raking **in the garden or yard**?

_____ **days per week**

☐ No moderate activity in garden or yard → ***Skip to question 18***

17. How much time did you usually spend on one of those days doing **moderate** physical activities in the garden or yard?

_____ **hours per day**
_____ **minutes per day**

18. Once again, think about only those physical activities that you did for at least 10 minutes at a time. During the **last 7 days**, on how many days did you do **moderate** activities like carrying light loads, washing windows, scrubbing floors and sweeping **inside your home**?

_____ **days per week**

☐ No moderate activity inside home → ***Skip to PART 4: RECREATION, SPORT AND LEISURE-TIME PHYSICAL ACTIVITY***

19. How much time did you usually spend on one of those days doing **moderate** physical activities inside your home?

_____ **hours per day**
_____ **minutes per day**

PART 4: RECREATION, SPORT, AND LEISURE-TIME PHYSICAL ACTIVITY

This section is about all the physical activities that you did in the **last 7 days** solely for recreation, sport, exercise or leisure. Please do not include any activities you have already mentioned.

20. Not counting any walking you have already mentioned, during the **last 7 days**, on how many days did you **walk** for at least 10 minutes at a time **in your leisure time**?

_____ **days per week**

☐ No walking in leisure time → ***Skip to question 22***

21. How much time did you usually spend on one of those days **walking** in your leisure time?

_____ **hours per day**
_____ **minutes per day**

22. Think about only those physical activities that you did for at least 10 minutes at a time. During the **last 7 days**, on how many days did you do **vigorous** physical activities like aerobics, running, fast bicycling, or fast swimming **in your leisure time**?

_____ **days per week**

☐ No vigorous activity in leisure time → ***Skip to question 24***

23. How much time did you usually spend on one of those days doing **vigorous** physical activities in your leisure time?

_____ **hours per day**
_____ **minutes per day**

24. Again, think about only those physical activities that you did for at least 10 minutes at a time. During the **last 7 days**, on how many days did you do **moderate** physical activities like bicycling at a regular pace, swimming at a regular pace, and doubles tennis **in your leisure time**?

_____ **days per week**

☐ No moderate activity in leisure time → ***Skip to PART 5: TIME SPENT SITTING***

25. How much time did you usually spend on one of those days doing **moderate** physical activities in your leisure time?
_____ **hours per day**
_____ **minutes per day**

PART 5: TIME SPENT SITTING

The last questions are about the time you spend sitting while at work, at home, while doing course work and during leisure time. This may include time spent sitting at a desk, visiting friends, reading or sitting or lying down to watch television. Do not include any time spent sitting in a motor vehicle that you have already told me about.

26. During the **last 7 days**, how much time did you usually spend **sitting** on a **weekday**?

_____ **hours per day**
_____ **minutes per day**

27. During the **last 7 days**, how much time did you usually spend **sitting** on a **weekend day**?

_____ **hours per day**
_____ **minutes per day**

MAIA (self report – have participant complete) - Below you will find a list of statements. Please indicate how often each statement applies to you generally in daily life.

	0- Never	1	2	3	4	5 - Alwa[illegible]
IA1_01. When I am tense I notice where the tension is located in my body.	☐	☐	☐	☐	☐	☐
IA1_02. I notice when I am uncomfortable in my body.	☐	☐	☐	☐	☐	☐
IA1_03. I notice where in my body I am comfortable.	☐	☐	☐	☐	☐	☐
IA1_04. I notice changes in my breathing, such as whether it slows down or speeds up.	☐	☐	☐	☐	☐	☐
IA1_05. I do not notice (I ignore) physical tension or discomfort until they become more severe.	☐	☐	☐	☐	☐	☐
IA1_06. I distract myself from sensations of discomfort.	☐	☐	☐	☐	☐	☐
IA1_07. When I feel pain or discomfort, I try to power through it.	☐	☐	☐	☐	☐	☐
IA1_08. When I feel physical pain, I become upset.	☐	☐	☐	☐	☐	☐
IA1_09. I start to worry that something is wrong if I feel any discomfort.	☐	☐	☐	☐	☐	☐
IA1_10. I can notice an unpleasant body sensation without worrying about it.	☐	☐	☐	☐	☐	☐
IA1_11. I can pay attention to my breath without being distracted by things happening around me.	☐	☐	☐	☐	☐	☐
IA1_12. I can maintain awareness of my inner bodily sensations even when there is a lot going on around me.	☐	☐	☐	☐	☐	☐
IA1_13. When I am in conversation with someone, I can pay attention to my posture.	☐	☐	☐	☐	☐	☐
IA1_14. I can return awareness to my body if I am distracted.	☐	☐	☐	☐	☐	☐
IA1_15. I can refocus my attention from thinking to sensing my body.	☐	☐	☐	☐	☐	☐
IA1_16. I can maintain awareness of my whole body even when a part of me is in pain or discomfort.	☐	☐	☐	☐	☐	☐
IA1_17. I am able to consciously focus on my body as a whole.	☐	☐	☐	☐	☐	☐
IA1_18. I notice how my body changes when I am angry.	☐	☐	☐	☐	☐	☐
IA1_19. When something is wrong in my life I can feel it in my body.	☐	☐	☐	☐	☐	☐
IA1_20. I notice that my body feels different after a peaceful experience.	☐	☐	☐	☐	☐	☐
IA1_21. I notice that my breathing becomes free and easy when I feel comfortable.	☐	☐	☐	☐	☐	☐
IA1_22. I notice how my body changes when I feel happy / joyful.	☐	☐	☐	☐	☐	☐

Commented [SF9]: Removed the MAAS in order to reduce participant burden. Not a primary or secondary outcome. Moved the MAIA here from the home survey.

Moved the MAIA and DERS here from the home baseline survey.

Deleted: MAAS Instructions: Below is a collection of statements about your everyday experience. Using the scale below, please indicate how frequently or infrequently you currently have each experience. Please answer according to what really reflects your experience rather than what you think your experience should be. Please treat each item separately from every other item. ¶

¶

***Please indicate the degree to which you agree with each of the following items using the scale below. Simply check your response to each item*¶**

...

	0- Never	1	2	3	4	5 - Always
IA1_23. When I feel overwhelmed I can find a calm place inside.	☐	☐	☐	☐	☐	☐
IA1_24. When I bring awareness to my body I feel a sense of calm.	☐	☐	☐	☐	☐	☐
IA1_25. I can use my breath to reduce tension.	☐	☐	☐	☐	☐	☐
IA1_26. When I am caught up in thoughts, I can calm my mind by focusing on my body/breathing.	☐	☐	☐	☐	☐	☐
IA1_27. I listen for information from my body about my emotional state.	☐	☐	☐	☐	☐	☐
IA1_28. When I am upset, I take time to explore how my body feels.	☐	☐	☐	☐	☐	☐
IA1_29. I listen to my body to inform me about what to do.	☐	☐	☐	☐	☐	☐
IA1_30. I am at home in my body.	☐	☐	☐	☐	☐	☐
IA1_31. I feel my body is a safe place.	☐	☐	☐	☐	☐	☐
IA1_32. I trust my body sensations.	☐	☐	☐	☐	☐	☐

DERS - **Please indicate how often the following statements apply to you by checking the box that best describes your experience.**

	Almost Never (0-10%)	Sometimes (11-35%)	About Half The Time (36-65%)	Most of the Time (66-90%)	Almost Always (91-100%)
ER1_01. I am clear about my feelings.	☐	☐	☐	☐	☐
ER1_02. I pay attention to how I feel.	☐	☐	☐	☐	☐
ER1_03. I experience my emotions as overwhelming and out of control.	☐	☐	☐	☐	☐
ER1_04. I have no idea how I am feeling.	☐	☐	☐	☐	☐
ER1_05. I have difficulty making sense out of my feelings.	☐	☐	☐	☐	☐
ER1_06. I am attentive to my feelings.	☐	☐	☐	☐	☐
ER1_07. I know exactly how I am feeling.	☐	☐	☐	☐	☐
ER1_08. I care about what I am feeling.	☐	☐	☐	☐	☐
ER1_09. I am confused about how I feel.	☐	☐	☐	☐	☐
ER1_10. When I'm upset, I acknowledge my emotions.	☐	☐	☐	☐	☐

Deleted: FFMQ - Please rate each of the following statements using the scale provided. Write the number in the blank that best describes *your own opinion* of what is *generally true for you*. ¶

...

ER1_11. When I'm upset, I become angry with myself for feeling that way.	☐	☐	☐	☐	☐
ER1_12. When I'm upset, I become embarrassed for feeling that way.	☐	☐	☐	☐	☐
	Almost Never (0-10%)	Sometimes (11-35%)	About Half The Time (36-65%)	Most of the Time (66-90%)	Almost Always (91-100%)
ER1_13. When I'm upset, I have difficulty getting work done.	☐	☐	☐	☐	☐
ER1_14. When I'm upset, I become out of control.	☐	☐	☐	☐	☐
ER1_15. When I'm upset, I believe that I will remain that way for a long time.	☐	☐	☐	☐	☐
ER1_16. When I'm upset, I believe that I will end up feeling very depressed.	☐	☐	☐	☐	☐
ER1_17. When I'm upset, I believe that my feelings are valid and important.	☐	☐	☐	☐	☐
ER1_18. When I'm upset, I have difficulty focusing on other things.	☐	☐	☐	☐	☐
ER1_19. When I'm upset, I feel out of control.	☐	☐	☐	☐	☐
ER1_20. When I'm upset, I can still get things done.	☐	☐	☐	☐	☐
ER1_21. When I'm upset, I feel ashamed at myself for feeling that way.	☐	☐	☐	☐	☐
ER1_22. When I'm upset, I know that I can find a way to eventually feel better.	☐	☐	☐	☐	☐

	Almost Never (0-10%)	Sometimes (11-35%)	About Half The Time (36-65%)	Most of the Time (66-90%)	Almost Always (91-100%)
ER1_23. When I'm upset, I feel like I am weak.	☐	☐	☐	☐	☐
ER1_24. When I'm upset, I feel like I can remain in control of my behaviours.	☐	☐	☐	☐	☐
ER1_25. When I'm upset, I feel guilty for feeling that way.	☐	☐	☐	☐	☐
ER1_26. When I'm upset, I have difficulty concentrating.	☐	☐	☐	☐	☐
ER1_27. When I'm upset, I have difficulty controlling my behaviours.	☐	☐	☐	☐	☐
ER1_28. When I'm upset, I believe there is nothing I can do to make myself feel better.	☐	☐	☐	☐	☐
ER1_29. When I'm upset, I become irritated at myself for feeling that way.	☐	☐	☐	☐	☐
ER1_30. When I'm upset, I start to feel very bad about myself.	☐	☐	☐	☐	☐
ER1_31. When I'm upset, I believe that wallowing in it is all I can do.	☐	☐	☐	☐	☐
ER1_32. When I'm upset, I lose control over my behaviour.	☐	☐	☐	☐	☐

	Almost Never (0-10%)	Sometimes (11-35%)	About Half The Time (36-65%)	Most of the Time (66-90%)	Almost Always (91-100%)
ER1_33. When I'm upset, I have difficulty thinking about anything else.	☐	☐	☐	☐	☐
ER1_34. When I'm upset I take time to figure out what I'm really feeling.	☐	☐	☐	☐	☐
ER1_35. When I'm upset, it takes me a long time to feel better.	☐	☐	☐	☐	☐
ER1_36. When I'm upset, my emotions feel overwhelming.	☐	☐	☐	☐	☐

Commented [SF10]: Moved the FFMQ to the home baseline survey.

Deleted: ¶

After self-report questionnaires are complete, participants will be asked to perform the following three activities.

Heart Beat Detection Task (5 minutes)

Open up the Kardia Mobile App. Have the participant sit in a chair with both feet on the ground and forearms on the table in front of him/her.

"We will now have you complete an activity where you will be asked to sit quietly and see if you can detect and count your heart beat. We will be using this ECG device."

Show participant the Kardia Mobile device and how it works. Demonstrate by completing a reading.

"We will have you complete this task three times at varying intervals. When I say 'begin' I want you to try your best to detect and count your heart beat. When I say 'stop' please let me know how many times you think your heart beat during the time interval. Try your best."

Have the participant place both hands on the Kardia Moble ECG device. Face the display screen away from the participant and towards yourself. Have the participant begin when the signal is strong and the timer begins. Have him/her stop when the timer ends. Record both the reading from the device and the particpant's estimated heart beat count. Repeat for three time intervals: 30 seconds, 30 seconds, 60 seconds. IMPORTANT: Do not let the participant know how long the time intervals are in length in order to deter guessing.

HEART BEAT DETECTION DATA SHEET (*example*)

INTERVAL	Heart Rate (Kardia Mobile)	# of Beats (calculation)	# of Beats (ppt count)
**1 – 30s**			
**2 – 30s**			
**3 – 60s**			

PSS-14 - The questions in this scale ask you about your feelings and thoughts during the last month. In each case, you will be asked to indicate by circling how often you felt or thought a certain way. Although some of the questions are similar, there are differences between them and you should treat each one as a separate question. The best approach is to answer fairly quickly. That is, don't try to count up the number of times you felt a particular way, but rather indicate the alternative that seems like a reasonable estimate.

Commented [SF11]: PSS-14 was moved here from the home baseline survey.

	Never 0	Almost Never 1	Sometimes 2	Fairly Often 3	Very Often 4
1. In the last month, how often have you been upset because of something that happened unexpectedly?	○	○	○	○	○
2. In the last month, how often have you felt that you were unable to control the important things in your life?	○	○	○	○	○
3. In the last month, how often have you felt nervous and "stressed"?	○	○	○	○	○
4. In the last month, how often have you dealt successfully with day to day problems and annoyances?	○	○	○	○	○
5. In the last month, how often have you felt that you were effectively coping with important changes that were occurring in your life?	○	○	○	○	○
6. In the last month, how often have you felt confident about your ability to handle your personal problems?	○	○	○	○	○
7. In the last month, how often have you felt that things were going your way?	○	○	○	○	○
8. In the last month, how often have you found that you could not cope with all the things that you had to do?	○	○	○	○	○
9. In the last month, how often have you been able to control irritations in your life?	○	○	○	○	○
10. In the last month, how often have you felt that you were on top of things?	○	○	○	○	○

	Never	Almost Never	Sometimes	Fairly Often	Very Often
	0	1	2	3	4
**11. In the last month, how often have you been angered because of things that happened that were outside of your control?**	○	○	○	○	○
**12. In the last month, how often have you found yourself thinking about things that you have to accomplish?**	○	○	○	○	○
**13. In the last month, how often have you been able to control the way you spend your time?**	○	○	○	○	○
**14. In the last month, how often have you felt difficulties were piling up so high that you could not overcome them?**	○	○	○	○	○

5-Trial Adjusting Delay Task (2-3 minutes)

"You will now complete a series of decision-making tasks. You will be asked to make choices between different amounts of money given to you now or after a delay. These are hypothetical choices, but please choose your answer as if the items were to be delivered as described. Each task will start with some brief instructions on the screen. Read these instructions, and press the 5 key on the keyboard when you are ready to begin. There are no right or wrong answers in the tasks, just choose which option you prefer in each case. Please take your time and answer thoughtfully. To select the option on the left side of the screen, press the left arrow, and to select the option on the right side of the screen, press the right arrow."

Sustained Attention to Response Task (15 minutes)

The Sustained Attention to Response Task (SART) is a computerized test of sustained attention, response inhibition (executive function) and self-regulation. Subjects are instructed to press a key in response to rapidly displayed integers (1-9) and withhold response to a designated "no-go" integer. SART errors consist of summed commission errors (button press on no-go trial) and omission errors (button not pressed on "go" integers). SART performance is associated with prefrontal cortex functioning, has been found to increase with mindfulness training and is correlated with scores on mindfulness questionnaires (specifically, the Mindful Attention Awareness Scale).

Appendix F – Revised Home Baseline* Assessment (v.3.0 – December 13, 2018)

with Track Changes highlighting revisions

HOME BASELINE ASSESSMENT

QUESTIONNAIRES ANSWERED BY PARTICIPANTS

AT BASELINE* (VIA ONLINE OR PAPER FORM)

* Questionnaires administered at follow ups are the same as baseline minus any measures that are not subject to change over time (e.g., race-ethnicity, childhood experiences, etc.)

The following measures are found in the home assessment:

- Demographics / background questions
- Eating practices / emotional eating (TFEQ-R21)
- Table salt
- Alcohol consumption
- Cigarette smoking
- Craving
- Self-control short form
- Self-compassion (SCS-SF)
- Mindfulness as measured by the FFMQ
- Social integration (ISEL-12)
- Loneliness (R-UCLA Loneliness Scale)
- Decentering (Experiences Questionnaire)
- Childhood SES
- Adverse childhood experiences (CTQ, food insecurity, and neglect subscale)
- Resilience (CD-RISC-10)
- Self-efficacy for chronic disease management (SECD-6)

The following measures were removed from the home assessment:

- Introductory questions
- Personal goals
- Emotion regulation (CERQ-18)
- Readiness to change for hypertensive risk factors

The following measures were moved:

- Race-ethnicity
- Perceived stress scale (PSS-14)
- Difficulties in Emotion Regulation (DERS)
- Interoception (MAIA)
- Blood pressure questions and family history of hypertension

Deleted: ¶

Commented [SF1]: This is not NEW. Was added as a clarifying statement.

Commented [SF2]: Unless otherwise stated, these measures have not been changed and have already been approved by the Brown IRB.

Commented [SF3]: Moved here from the in-person baseline assessment

Questionnaire Table of Contents

Background Questions 3

Eating Practices 5

Alcohol Consumption 7

Smoking 8

About You 9

Parent's Education 22

Your Childhood Experiences 24

Connor-Davidson Resilience Scale 10 (CD-RISC-10) 30

Chronic Illness 31

END SCRIPT 32

Introduction

We appreciate you taking the time to participate in this research study. These questionnaires will ask a series of questions on various aspects of your health, health behaviours, family, and other life circumstances. It should take approximately one hour. Please keep in mind that you can refuse to answer any questions that you are not comfortable with.

IQ1_01. Please enter the 4-digit ID number you were given. ___ ___ ___ ___.

Background Questions

To start, please answer a few questions about you.

BQ1_04. Which of the following best describes your current work situation? ***(select one only)***

- ☐ Working full-time
- ☐ Working part-time
- ☐ Retired
- ☐ Unemployed: Looking for work
- ☐ Unemployed: Not currently looking for work
- ☐ Unemployed due to disability
- ☐ Keeping house or raising children full-time
- ☐ Military
- ☐ Full-time student
- ☐ Other:________________________
- ☐ I do not know
- ☐ I prefer not to answer

BQ1_05. What is the highest grade or level of regular school you have completed?

- ☐ Elementary School
- ☐ Junior High
- ☐ High School
- ☐ Associate degree (Junior College)
- ☐ College
- ☐ Graduate School
- ☐ Other: ______________________________
- ☐ I do not know
- ☐ I prefer not to answer

Deleted: Introductory Questions¶

Commented [FBS4]: Removed both the INTRO and Personal Goal questions in order to reduce participant burden. Not a primary or secondary outcome.

Deleted: ¶

Deleted: ¶

Deleted: IQ1_02. What is your main reason for participating in this study?¶
¶
¶
¶
¶
¶
IQ1_03. What do you care about most?¶
¶
¶
¶
¶
¶
IQ1_04. What gives you the most pleasure in your life?¶
¶
¶
¶
¶
¶
IQ1_05. What are your greatest worries?¶

Deleted: Page Break

Deleted: **Please list three personal goals you have for taking this mindfulness program:**¶
¶
PG1_01. ____¶
¶
PG1_02. ____¶
¶
PG1_03

Deleted: . ____¶
Page Break

Commented [SF6]: Moved race-ethnicity questions to the in-person screener

BQ 6-8 Intentionally skipped

Commented [SF7]: These were removed in a previous IRB amendement

BQ1_09a. Do you currently live alone?

- ☐ No
- ☐ Yes → ***skip to next section***
- ☐ I do not know
- ☐ I prefer not to answer

BQ1_09b. How many people currently live in your household, including yourself?

____

BQ1_09c. Of these people, how many are under 18?

____

BQ1_09d. Of the adults in your household (including yourself), how many bring income into the household?

____

Eating Practices

	Definitely True	Mostly True	Mostly False	Definitely False
EE1_01. I deliberately take small helpings to control my weight.	□	□	□	□
EE1_02. I start to eat when I feel anxious.	□	□	□	□
EE1_03. Sometimes when I start eating, I just can't seem to stop.	□	□	□	□
EE1_04. When I feel sad, I often eat too much.	□	□	□	□
EE1_05. I don't eat some foods because they make me fat.	□	□	□	□
EE1_06. Being with someone who is eating, often makes me want to also eat.	□	□	□	□
EE1_07. When I feel tense or ''wound up'', I often feel I need to eat.	□	□	□	□
EE1_08. I often get so hungry that my stomach feels like a bottomless pit.	□	□	□	□
EE1_09. I'm always so hungry that it's hard for me to stop eating before finishing all of the food on my plate.	□	□	□	□
EE1_10. When I feel lonely, I console myself by eating.	□	□	□	□
EE1_11. I consciously hold back on how much I eat at meals to keep from gaining weight.	□	□	□	□
EE1_12. When I smell a sizzling steak or see a juicy piece of meat, I find it very difficult to keep from eating even if I've just finished a meal.	□	□	□	□
EE1_13. I'm always hungry enough to eat at any time.	□	□	□	□
EE1_14. If I feel nervous, I try to calm down by eating.	□	□	□	□
EE1_15. When I see something that looks very delicious, I often get so hungry that I have to eat right away.	□	□	□	□
EE1_16. When I feel depressed, I want to eat.	□	□	□	□

	Almost Never	Seldom	Usually	Almost Always
EE1_17. How often do you avoid ‘stocking up’’ on tempting foods?	☐	☐	☐	☐

	Unlikely	A little likely	Somewhat likely	Very likely
EE1_18. How likely are you to make an effort to eat less than you want?	☐	☐	☐	☐

	Never	Rarely	Sometimes	At least once a week
EE1_19. Do you go on eating binges even though you’re not hungry?	☐	☐	☐	☐

	Only at mealtimes	Sometimes between meals	Often between meals	Almost always
EE1_20. How often do you feel hungry?	☐	☐	☐	☐

	1	2	3	4	5	6	7	8
EE1_21. On a scale from 1 to 8, where 1 means no restraint in eating and 8 means total restraint, what number would you give yourself? Mark the number that best applies to you:	☐	☐	☐	☐	☐	☐	☐	☐

Table Salt Use:

TS1_01 Please report your <u>average</u> total use, <u>during the past year</u>, of “salt added at the table”. Would you say…

- Never .... □
- Less than once per month .... □
- 1-3 shakes per month .... □
- 1 shake per week .... □
- 2-4 shakes per week .... □
- 5-6 shakes per week .... □
- 1 shake per day .... □
- 2-3 shakes per day .... □
- 4-5 shakes per day .... □
- 6+ shakes per day .... □

Alcohol Consumption

A drink of alcohol is defined as 1 can or bottle of beer, 1 glass of wine, 1 can or bottle of wine cooler, 1 cocktail, or 1 shot of liquor.

AC1_01. During the past 30 days, how many days per week or per month did you have at least 1 drink of any alcoholic beverage? [if none, ***skip to next section***]

____

AC1_02. On the days when you drank, about how many drinks did you drink on average?

____

AC1_03. ***<u>Men:</u>*** Considering all types of alcoholic beverages, how many times during the past 30 days did you have **<u>5</u>** or more drinks on an occasion?

<u>Women:</u> Considering all types of alcoholic beverages, how many times during the past 30 days did you have ***<u>4</u>*** or more drinks on an occasion?

____

Smoking

SM1_01. Have you smoked at least 100 cigarettes in your entire life?

- ☐ Yes
- ☐ No
- ☐ I Do Not Know
- ☐ Prefer not to answer

SM1_02. Did you ever become a daily smoker (that is, smoke every day or nearly every day for two months or longer)?

- ☐ Yes
- ☐ No → ***skip to next section***
- ☐ I Do Not Know
- ☐ Prefer not to answer

SM1_03. How old were you when you last smoked daily?

Age ______ (in years)

☐ I Do Not Know ☐ Prefer not to answer ☐ Still smoking daily

SM1_04. Do you smoke cigarettes now?

- ☐ Yes
- ☐ No → ***skip to next section***
- ☐ I Do Not Know
- ☐ Prefer not to answer

SM1_04a. How many cigarettes per day do you smoke? (One pack equals 20 cigarettes)

Number of cigarettes ________

- ☐ I Do Not Know
- ☐ Prefer not to answer

About You

Please bring to mind a type of very tasty food that may contribute to hypertension through high salt intake or through eating too many calories (e.g., sweet sugary dessert, salty snack foods, etc.).

Think about the LAST WEEK you MOST WANTED this type of food. For each item, select a number (0 to 10) to indicate your rating.

At that time…	Not at All 0	1	2	3	4	5	6	7	8	9	Extremely 10
1. …how much did you want it?	☐	☐	☐	☐	☐	☐	☐	☐	☐	☐	☐
2. …how much did you need it?	☐	☐	☐	☐	☐	☐	☐	☐	☐	☐	☐
3. …how strong was the urge to have it?	☐	☐	☐	☐	☐	☐	☐	☐	☐	☐	☐
At that time, how vividly did you…											
4. …picture it?	☐	☐	☐	☐	☐	☐	☐	☐	☐	☐	☐
5. …imagine its taste?	☐	☐	☐	☐	☐	☐	☐	☐	☐	☐	☐
6. …imagine its smell?	☐	☐	☐	☐	☐	☐	☐	☐	☐	☐	☐
7. …imagine what it would feel like in your mouth or throat?	☐	☐	☐	☐	☐	☐	☐	☐	☐	☐	☐
8. …imagine how your body would feel?	☐	☐	☐	☐	☐	☐	☐	☐	☐	☐	☐
At that time…											
9. …how hard were you trying not to think about it?	☐	☐	☐	☐	☐	☐	☐	☐	☐	☐	☐
10. …how intrusive were the thoughts?	☐	☐	☐	☐	☐	☐	☐	☐	☐	☐	☐
11. …how hard was it to think about anything else?	☐	☐	☐	☐	☐	☐	☐	☐	☐	☐	☐

Please bring to mind any times in the LAST WEEK when you had a desire to do sedentary activities (e.g., read a book, watch a movie, be on the computer, etc.) instead of physical activities (e.g., walking, gardening, exercise).

Think about the LAST WEEK you MOST WANTED to do a sedentary activity. For each item, select a number (0 to 10) to indicate your rating.

At that time…	**Not at All 0**	**1**	**2**	**3**	**4**	**5**	**6**	**7**	**8**	**9**	**Extremely 10**
1. …how much did you want it?	◻	◻	◻	◻	◻	◻	◻	◻	◻	◻	◻
2. …how much did you need it?	◻	◻	◻	◻	◻	◻	◻	◻	◻	◻	◻
3. …how strong was the urge to have it?	◻	◻	◻	◻	◻	◻	◻	◻	◻	◻	◻
At that time, how vividly did you…											
4. …picture it?	◻	◻	◻	◻	◻	◻	◻	◻	◻	◻	◻
5. …imagine how your body would feel?	◻	◻	◻	◻	◻	◻	◻	◻	◻	◻	◻
At that time…											
6. …how hard were you trying not to think about it?	◻	◻	◻	◻	◻	◻	◻	◻	◻	◻	◻
7. …how intrusive were the thoughts?	◻	◻	◻	◻	◻	◻	◻	◻	◻	◻	◻
8. …how hard was it to think about anything else?	◻	◻	◻	◻	◻	◻	◻	◻	◻	◻	◻

Please bring to mind any times in the LAST WEEK when you had a desire to drink alcohol, such as wine, beer or spirits.

Think about the LAST WEEK you MOST WANTED alcohol. For each item, select a number (0 to 10) to indicate your rating.

At that time…	Not at All 0	1	2	3	4	5	6	7	8	9	Extremely 10
1. …how much did you want it?	□	□	□	□	□	□	□	□	□	□	□
2. …how much did you need it?	□	□	□	□	□	□	□	□	□	□	□
3. …how strong was the urge to have it?	□	□	□	□	□	□	□	□	□	□	□
At that time, how vividly did you…											
4. …picture it?	□	□	□	□	□	□	□	□	□	□	□
5. …imagine its taste?	□	□	□	□	□	□	□	□	□	□	□
6. …imagine its smell?	□	□	□	□	□	□	□	□	□	□	□
7. …imagine what it would feel like in your mouth or throat?	□	□	□	□	□	□	□	□	□	□	□
8. …imagine how your body would feel?	□	□	□	□	□	□	□	□	□	□	□
At that time…											
9. …how hard were you trying not to think about it?	□	□	□	□	□	□	□	□	□	□	□
10. …how intrusive were the thoughts?	□	□	□	□	□	□	□	□	□	□	□
11. …how hard was it to think about anything else?	□	□	□	□	□	□	□	□	□	□	□

Using the scale provided, please indicate how much each of the following statements reflects how you typically are.

	Not at all	A little	Somewhat	Fair Amount	Very much
SC1_01. I am good at resisting temptation.	☐	☐	☐	☐	☐
SC1_02. I have a hard time breaking bad habits.	☐	☐	☐	☐	☐
SC1_03. I am lazy.	☐	☐	☐	☐	☐
SC1_04. I say inappropriate things.	☐	☐	☐	☐	☐
SC1_05. I do certain things that are bad for me, if they are fun.	☐	☐	☐	☐	☐
SC1_06. I refuse things that are bad for me.	☐	☐	☐	☐	☐
SC1_07. I wish I had more self-discipline.	☐	☐	☐	☐	☐
SC1_08. People would say that I have iron self-discipline.	☐	☐	☐	☐	☐
SC1_09. Pleasure and fun sometimes keep me from getting work done.	☐	☐	☐	☐	☐
SC1_10. I have trouble concentrating.	☐	☐	☐	☐	☐
SC1_11. I am able to work effectively toward long-term goals.	☐	☐	☐	☐	☐
SC1_12. Sometimes I can't stop myself from doing something, even if I know it is wrong.	☐	☐	☐	☐	☐
SC1_13. I often act without thinking through all the alternatives.	☐	☐	☐	☐	☐

Please read each statement carefully before answering. To the left of each item, indicate how often you behave in the stated manner, using the following scale:

	Almost never	Not very often	Sometimes	Frequently	Almost always
CO1_O1. When I fail at something important to me, I become consumed by feelings of inadequacy.					
CO1_02. I try to be understanding and patient towards those aspects of my personality I don't like.					
CO1_O3. When something painful happens I try to take a balanced view of the situation.					
CO1_O4. When I'm feeling down, I tend to feel like most other people are probably happier than I am.					
CO1_O5. I try to see my failings as part of the human condition.					
CO1_O6. When I'm going through a very hard time, I give myself the caring and tenderness I need.					
CO1_O7. When something upsets me I try to keep my emotions in balance.					
CO1_O8. When I fail at something that's important to me, I tend to feel alone in my failure					
CO1_O9. When I'm feeling down I tend to obsess and fixate on everything that's wrong.					

	Almost never	Not very often	Sometimes	Frequently	Almost always
CO1_10. When I feel inadequate in some way, I try to remind myself that feelings of inadequacy are shared by most people.					
CO1_11. I'm disapproving and judgmental about my own flaws and inadequacies.					
CO1_12. I'm intolerant and impatient towards those aspects of my personality I don't like.					

Commented [SF9]: Moved the PSS-14 to the in-person baseline assessment

Deleted: The questions in this scale ask you about your feelings and thoughts during the last month. In each case, you will be asked to indicate by circling how often you felt or thought a certain way. Although some of the questions are similar, there are differences between them and you should treat each one as a separate question. The best approach is to answer fairly quickly. That is, don't try to count up the number of times you felt a particular way, but rather indicate the alternative that seems like a reasonable estimate

Deleted: .¶

FFMQ - Please rate each of the following statements using the scale provided. Write the number in the blank that best describes *your own opinion* of what is *generally true for you.*

	1 – Never or very rarely true	2 – Rarely true	3 – Sometimes true	4- Often true	5 – Very often or always true
FF1_01. When I'm walking, I deliberately notice the sensations of my body moving.	☐	☐	☐	☐	☐
FF1_02. I'm good at finding words to describe my feelings.	☐	☐	☐	☐	☐
FF1_03. I criticize myself for having irrational or inappropriate emotions.	☐	☐	☐	☐	☐
FF1_04. I perceive my feelings and emotions without having to react to them.	☐	☐	☐	☐	☐
FF1_05. When I do things, my mind wanders off and I'm easily distracted.	☐	☐	☐	☐	☐
FF1_06. When I take a shower or bath, I stay alert to the sensations of water on my body.	☐	☐	☐	☐	☐
FF1_07. I can easily put my beliefs, opinions, and expectations into words.	☐	☐	☐	☐	☐
FF1_08. I don't pay attention to what I'm doing because I'm daydreaming, worrying, or otherwise distracted.	☐	☐	☐	☐	☐
FF1_09. I watch my feelings without getting lost in them.	☐	☐	☐	☐	☐
FF1_10. I tell myself I shouldn't be feeling the way I'm feeling.	☐	☐	☐	☐	☐
FF1_11. I notice how foods and drinks affect my thoughts, bodily sensations, and emotions.	☐	☐	☐	☐	☐
FF1_12. It's hard for me to find the words to describe what I'm thinking.	☐	☐	☐	☐	☐
FF1_13. I am easily distracted.	☐	☐	☐	☐	☐
FF1_14. I believe some of my thoughts are abnormal or bad and I shouldn't think that way.	☐	☐	☐	☐	☐
FF1_15. I pay attention to sensations, such as the wind in my hair or sun on my face.	☐	☐	☐	☐	☐

Commented [SF10]: Moved the DERS to the in-person assessments as part of the 30 minute recovery survey.

Moved the FFMQ here from the in-person assessment.

Deleted: **Please indicate how often the following statements apply to you by checking the box that best describes your experience.** ¶

Formatted: Space Before: 0 pt, After: 0 pt

Deleted: Page Break ¶

	1 – Never or very rarely true	2 – Rarely true	3 – Sometimes true	4- Often true	5 – Very often or always true
FF1_16. I have trouble thinking of the right words to express how I feel about things.	□	□	□	□	□
FF1_17. I make judgments about whether my thoughts are good or bad.	□	□	□	□	□
FF1_18. I find it difficult to stay focused on what’s happening in the present.	□	□	□	□	□
FF1_19. When I have distressing thoughts or images, I “step back” and am aware of the thought or image without getting taken over by it.	□	□	□	□	□
FF1_20. I pay attention to sounds, such as clocks ticking, birds chirping, or cars passing.	□	□	□	□	□
FF1_21. In difficult situations, I can pause without immediately reacting.	□	□	□	□	□
FF1_22. When I have a sensation in my body, it’s difficult for me to describe it because I can’t find the right words.	□	□	□	□	□
FF1_23. It seems I am “running on automatic” without much aware- ness of what I’m doing.	□	□	□	□	□
FF1_24. When I have distressing thoughts or images, I feel calm soon after.	□	□	□	□	□
FF1_25. I tell myself that I shouldn’t be thinking the way I’m thinking.	□	□	□	□	□
FF1_26. I notice the smells and aromas of things.	□	□	□	□	□
FF1_27. Even when I’m feeling terribly upset, I can find a way to put it into words.	□	□	□	□	□
FF1_28. I rush through activities without being really attentive to them.	□	□	□	□	□

	1 – Never or very rarely true	2 – Rarely true	3 – Sometimes true	4- Often true	5 – Very often or always true
FF1_29. When I have distressing thoughts or images, I am able just to notice them without reacting.	☐	☐	☐	☐	☐
FF1_30. I think some of my emotions are bad or inappropriate and I shouldn’t feel them.	☐	☐	☐	☐	☐
FF1_31. I notice visual elements in art or nature, such as colors, shapes, textures, or patterns of light and shadow.	☐	☐	☐	☐	☐
FF1_32. My natural tendency is to put my experiences into words.	☐	☐	☐	☐	☐
FF1_33. When I have distressing thoughts or images, I just notice them and let them go.	☐	☐	☐	☐	☐
FF1_34. I do jobs or tasks automatically without being aware of what I’m doing.	☐	☐	☐	☐	☐
FF1_35. When I have distressing thoughts or images, I judge myself as good or bad depending what the thought or image is about.	☐	☐	☐	☐	☐
FF1_36. I pay attention to how my emotions affect my thoughts and behavior.	☐	☐	☐	☐	☐
FF1_37. I can usually describe how I feel at the moment in consider- able detail.	☐	☐	☐	☐	☐
FF1_38. I find myself doing things without paying attention.	☐	☐	☐	☐	☐
FF1_39. I disapprove of myself when I have irrational ideas.	☐	☐	☐	☐	☐

This scale is made up of a list of statements each of which may or may not be true about you. For each statement, select "definitely true" if you are sure it is true about you and "probably true" if you think it is true but are not absolutely certain. Similarly, you should select "definitely false" if you are sure the statement is false and "probably false" if you think it is false but are not absolutely certain.

	Definitely False	Probably False	Probably True	Definitely True
**IS1_01** If I wanted to go on a trip for a day (for example, to the country or mountains), I would have a hard time finding someone to go with me.	☐	☐	☐	☐
**IS1_02** I feel that there is no one I can share my most private worries and fears with.	☐	☐	☐	☐
**IS1_03** If I were sick, I could easily find someone to help me with my daily chores.	☐	☐	☐	☐
**IS1_04** There is someone I can turn to for advice about handling problems with my family.	☐	☐	☐	☐
**IS1_05** If I decide one afternoon that I would like to go to a movie that evening, I could easily find someone to go with me.	☐	☐	☐	☐
**IS1_06** When I need suggestions on how to deal with a personal problem, I know someone I can turn to.	☐	☐	☐	☐
**IS1_07** I don't often get invited to do things with others.	☐	☐	☐	☐
**IS1_08** If I had to go out of town for a few weeks, it would be difficult to find someone who would look after my house or apartment (the plants, pets, garden, etc.).	☐	☐	☐	☐
**IS1_09** If I wanted to have lunch with someone, I could easily find someone to join me.	☐	☐	☐	☐

	Definitely False	Probably False	Probably True	Definitely True
**IS1_10** If I was stranded 10 miles from home, there is someone I could call who could come and get me.	☐	☐	☐	☐
**IS1_11** If a family crisis arose, it would be difficult to find someone who could give me good advice about how to handle it.	☐	☐	☐	☐
**IS1_12** If I needed some help in moving to a new house or apartment, I would have a hard time finding someone to help me.	☐	☐	☐	☐

Please indicate how often each of the statements below is descriptive of you.

**Statement**	Never	Rarely	Sometimes	Often
LS1_01. I feel in tune with the people around me	☐	☐	☐	☐
LS1_02. I lack companionship	☐	☐	☐	☐
LS1_03. There is no one I can turn to	☐	☐	☐	☐
LS1_04. I do not feel alone	☐	☐	☐	☐
LS1_05. I feel part of a group of friends	☐	☐	☐	☐
LS1_06. I have a lot in common with the people around me	☐	☐	☐	☐
LS1_07. I am no longer close to anyone	☐	☐	☐	☐
LS1_08. My interests and ideas are not shared by those around me	☐	☐	☐	☐
LS1_09. I am an outgoing person	☐	☐	☐	☐
LS1_10. There are people I feel close to	☐	☐	☐	☐
LS1_11. I feel left out	☐	☐	☐	☐
LS1_12. My social relationships are superficial	☐	☐	☐	☐
LS1_13. No one really knows me well	☐	☐	☐	☐
LS1_14. I feel isolated from others	☐	☐	☐	☐
LS1_15. I can find companionship when I want it	☐	☐	☐	☐
LS1_16. There are people who really understand me	☐	☐	☐	☐
LS1_17. I am unhappy being so withdrawn	☐	☐	☐	☐
LS1_18. People are around me but not with me	☐	☐	☐	☐
LS1_19. There are people I can talk to	☐	☐	☐	☐
LS1_20. There are people I can turn to	☐	☐	☐	☐

We are interested in your recent experiences. Below is a list of things that people sometimes experience. Next to each item are five choices: “never”, “rarely”, “sometimes”, “often”, and “all the time”. Please choose one of these to indicate how much you currently have experiences similar to those described.

Please do not spend too long on each item–it is your first response that we are interested in. Please be sure to answer every item.

	Never	Rarely	Sometimes	Often	All the time
RD1_01. I think about what will happen in the future.	☐	☐	☐	☐	☐
RD1_02. I remind myself that thoughts aren’t facts.	☐	☐	☐	☐	☐
RD1_03. I am better able to accept myself as I am.	☐	☐	☐	☐	☐
RD1_04. I notice all sorts of little things and details in the world around me.	☐	☐	☐	☐	☐
RD1_05. I am kinder to myself when things go wrong.	☐	☐	☐	☐	☐
RD1_06. I can slow my thinking at times of stress.	☐	☐	☐	☐	☐
RD1_07. I wonder what kind of person I really am.	☐	☐	☐	☐	☐
RD1_08. I am not so easily carried away by my thoughts and feelings.	☐	☐	☐	☐	☐
RD1_09. I notice that I don’t take difficulties so personally.	☐	☐	☐	☐	☐
RD1_10. I can separate myself from my thoughts and feelings.	☐	☐	☐	☐	☐
RD1_11. I analyze why things turn out the way they do.	☐	☐	☐	☐	☐
RD1_12. I can take time to respond to difficulties.	☐	☐	☐	☐	☐
RD1_13. I think over and over again about what others have said to me.	☐	☐	☐	☐	☐
RD1_14. I can treat myself kindly.	☐	☐	☐	☐	☐
RD1_15. I can observe unpleasant feelings without being drawn into them.	☐	☐	☐	☐	☐
RD1_16. I have the sense that I am fully aware of what is going on around me and inside me.	☐	☐	☐	☐	☐
RD1_17. I can actually see that I am not my thoughts.	☐	☐	☐	☐	☐
RD1_18. I am consciously aware of a sense of my body as a whole.	☐	☐	☐	☐	☐
RD1_19. I think about the ways in which I am different from other people.	☐	☐	☐	☐	☐
RD1_20. I view things from a wider perspective.	☐	☐	☐	☐	☐

Commented [SF11]: The MAIA scale is one of the primary outcomes, so it was moved to the in-person assessment.

Deleted: ¶
Below you will find a list of statements. Please indicate how often each statement applies to you generally in daily life. ¶
¶ ...

Deleted: Page Break ¶

Commented [SF12]: Removed CERQ-18 to reduce participant burden.

Deleted: How do you cope with events? Everyone gets confronted with negative or unpleasant events now and then and everyone responds to them in his or her own way. By the following questions you are asked to indicate what you generally think, when you experience negative or unpleasant events.¶
¶ ...

Parent's Education

CS1_01. Please check the box beside the highest grade or degree that your BIOLOGICAL MOTHER completed.

Never went to school	☐
Grades 1 to 3	☐
Grades 4 to 8	☐
Grades 9 to 11	☐
Grade 12	☐
GED	☐
One or more years of Vocational or Professional School after High School	☐
One or more years of College	☐
One or more years of Graduate or Professional School after College	☐
I Do Not Know	☐
I prefer not to answer	☐

CS1_02. Please check the box beside the highest grade or degree that your BIOLOGICAL FATHER completed.

Never went to school	☐
Grades 1 to 3	☐
Grades 4 to 8	☐
Grades 9 to 11	☐
Grade 12	☐
GED	☐
One or more years of Vocational or Professional School after High School	☐
One or more years of College	☐
One or more years of Graduate or Professional School after College	☐
I Do Not Know	☐
I prefer not to answer	☐

Now please think of the two most important adults in your home between the time you were born and age 18 years. Please check the category below that best described their level of education during this time period.

CS1_03. First adult's highest level of education:

Never went to school	☐
Grades 1 to 3	☐
Grades 4 to 8	☐
Grades 9 to 11	☐
Grade 12	☐
GED	☐
One or more years of Vocational or Professional School after High School	☐
One or more years of College	☐
One or more years of Graduate or Professional School after College	☐
I Do Not Know	☐
I prefer not to answer	☐

CS1_04. Second adult's highest level of education

Never went to school	☐
Grades 1 to 3	☐
Grades 4 to 8	☐
Grades 9 to 11	☐
Grade 12	☐
GED	☐
One or more years of Vocational or Professional School after High School	☐
One or more years of College	☐
One or more years of Graduate or Professional School after College	☐
I Do Not Know	☐
I prefer not to answer	☐

<u>Your Childhood Experiences</u>

The following questions ask about some difficult experiences that you might have had as a child. These questions may be emotionally difficult to answer. Just as a reminder, you do not need answer any questions that you would prefer not to. Your answers to these questions, as with all questions, will remain confidential.

Childhood Trauma Questionnaire (CTQ)

**When I was growing up:**	**Never True**	**Rarely True**	**Sometimes True**	**Often True**	**Very Often True**
1. I didn't have enough to eat.	1	2	3	4	5
2. I knew there was someone to take care of me and protect me.	1	2	3	4	5
3. People in my family called me things like "stupid", "lazy", or "ugly."	1	2	3	4	5
4. My parents were too drunk or high to take care of the family.	1	2	3	4	5
5. There was someone in my family who helped me feel important or special.	1	2	3	4	5
6. I had to wear dirty clothes.	1	2	3	4	5
7. I felt loved.	1	2	3	4	5
8. I thought that my parents wished I had never been born.	1	2	3	4	5
9. I got hit so hard by someone in my family that I had to see a doctor or go to the hospital.	1	2	3	4	5
10. There was nothing I wanted to change about my family.	1	2	3	4	5
11. People in my family hit me so hard that it left me with bruises or marks.	1	2	3	4	5
12. I was punished with a belt, a board, a cord, or some other hard object.	1	2	3	4	5

13. People in my family looked out for each other.	1	2	3	4	5
14. People in my family said hurtful or insulting things to me.	1	2	3	4	5
15. I believe that I was physically abused.	1	2	3	4	5
16. I had the perfect childhood.	1	2	3	4	5
17. I got hit or beaten so badly that it was noticed by someone like a teacher, neighbor, or doctor.	1	2	3	4	5
18. I felt that someone in my family hated me.	1	2	3	4	5
19. People in my family felt close to each other.	1	2	3	4	5
20. Someone tried to touch me in a sexual way, or tried to make me touch them.	1	2	3	4	5
21. Someone threatened to hurt me or tell lies about me unless I did something sexual with them.	1	2	3	4	5
22. I had the best family in the world.	1	2	3	4	5
23. Someone tried to make me do sexual things or watch sexual things.	1	2	3	4	5
24. Someone molested me.	1	2	3	4	5
25. I believe that I was emotionally abused.	1	2	3	4	5
26. There was someone to take me to the doctor if I needed it.	1	2	3	4	5
27. I believe that I was sexually abused.	1	2	3	4	5
28. My family was a source of strength and support.	1	2	3	4	5

SAFETY PROMPT – THE BELOW TEXT WILL BE DISPLAYED TO PPTS WHO SCORE AS MODERATE TO SEVERE ON THE CHILDHOOD TRAUMA QUESTIONNAIRE (CTQ):

Thank you for completing that section. Your well-being is important to us. Please note that based on your responses, this information will be passed along to our study clinician for further review and possible follow up. To assist with that review, please answer the below questions.

Are you currently experiencing any distress related to your experiences as a child? YES / NO

IF YES – describe below:

IF EXPERIENCING DISTRESS – Are you currently under the care of a counsellor, therapist, etc. for these symptoms of distress?

Thank you. This information will be sent along to the study clinician for further review and possible follow up. However, note that the data will not be officially seen and sent along until your survey is completed and submitted. If you wish to get in touch with us sooner, please call 401-400-4768 or, if it is an emergency, please call 911.

Food Insecurity Items
National Comorbidity Survey Replication – Adolescent Supplement

Adapted by Eric Loucks for Adult-Administered Retrospective Reporting During Childhood

During childhood, were you ever hungry but did not eat because you could not afford to buy food?

1) Yes
2) No

During childhood, did you ever eat less than you felt you should because you or your family didn't have money to buy food?

1) Yes
2) No

How often during childhood did you not have enough money to buy food?

1) Never
2) Rarely
3) Sometimes
4) Often

How often during childhood could you not afford to eat balanced meals?

1) Never
2) Rarely
3) Sometimes
4) Often

AS YOU REMEMBER YOUR MOTHER FIGURE:
Please circle the appropriate number. If you had more than one mother figure, choose the one you were with longest, or the one you found most difficult to live with.

WHICH MOTHER FIGURE ARE YOU DESCRIBING BELOW?

1. Natural mother
2. Step-mother/ father's live-in partner
3. Other relative (e.g., aunt, grandmother)
4. Other non-relative (e.g., foster mother, godmother)
5. Other (describe) ___________________________

	NO<br>NOT AT ALL		UNSURE		YES<br>DEFINITELY
1. She was concerned about my worries ………	1	2	3	4	5
2. She was interested in how I did at school.	1	2	3	4	5
3. She tried to make me feel better when I was upset…………..	1	2	3	4	5
4. She was interested in who my friends were	1	2	3	4	5
5. She was concerned about where I was and what I was doing…	1	2	3	4	5
6. She cared for me when I was sick …………	1	2	3	4	5

AS YOU REMEMBER YOUR FATHER FIGURE:
Please circle the appropriate number. If you had more than one father figure, choose the one you were with longest, or the one you found most difficult to live with. If you had no father in the household then leave out this section.

WHICH FATHER FIGURE ARE YOU DESCRIBING BELOW?

1. Natural father
2. Step-father/ mother's live-in partner
3. Other relative (e.g., uncle, grandfather)
4. Other non-relative (e.g., foster father, godfather)
5. Other (describe) ___________________________

	**NO**				**YES**
	**NOT AT ALL**		**UNSURE**		**DEFINITELY**
1. He was concerned about my worries ………	1	2	3	4	5
2. He was interested in how I did at school.	1	2	3	4	5
3. He tried to make me feel better when I was upset…………..	1	2	3	4	5
4. He was interested in who my friends were	1	2	3	4	5
5. He was concerned about where I was and what I was doing…	1	2	3	4	5
6. He cared for me when I was sick …………	1	2	3	4	5

Connor-Davidson Resilience Scale 10 (CD-RISC-10)

*Please indicate how much you agree with the following statements as they apply to you over the last **month**. If a particular situation has not occurred recently, answer according to how you think you would have felt.*

		not true at all (0)	rarely true (1)	sometimes true (2)	often true (3)	true nearly all the time (4)
1.	I am able to adapt when changes occur.	☐	☐	☐	☐	☐
2.	I can deal with whatever comes my way.	☐	☐	☐	☐	☐
3.	I try to see the humorous side of things when I am faced with problems.	☐	☐	☐	☐	☐
4.	Having to cope with stress can make me stronger.	☐	☐	☐	☐	☐
5.	I tend to bounce back after illness, injury, or other hardships.	☐	☐	☐	☐	☐
6.	I believe I can achieve my goals, even if there are obstacles.	☐	☐	☐	☐	☐
7.	Under pressure, I stay focused and think clearly.	☐	☐	☐	☐	☐
8.	I am not easily discouraged by failure.	☐	☐	☐	☐	☐
9.	I think of myself as a strong person when dealing with life's challenges and difficulties.	☐	☐	☐	☐	☐
10.	I am able to handle unpleasant or painful feelings like sadness, fear, and anger.	☐	☐	☐	☐	☐

Chronic Illness

CD1_01. Do you have a chronic illness, health problem or disease?

□ Yes
□ No (if responding "no", please skip to the next section).

CD1_02. Please select the illness from the list below that has the greatest effect on your life or feels like the *most important* for you to be able to manage. You may have more than one, but the purpose of this question is to identify what you might view as your PRIMARY chronic illness/problem/disease.

- ☐ Diabetes
- ☐ Heart Disease
- ☐ Hypertension
- ☐ Obesity
- ☐ Metabolic Syndrome
- ☐ Arthritis
- ☐ Chronic Pain
- ☐ Asthma
- ☐ COPD
- ☐ Depression
- ☐ Anxiety
- ☐ Insomnia
- ☐ Substance use
- ☐ Tobacco use
- ☐ Alcohol overuse
- ☐ Prescription medication overuse
- ☐ Illicit drug use
- ☐ Other ______________________

We would like to know how confident you are in doing certain activities related to the chronic illness or disease you selected above. For each of the following questions, please choose the number that corresponds to your confidence that you can do the tasks regularly at the present time. Please keep the chronic illness or disease in mind as you answer the following questions.

How confident are you that….	Not at All Confident 1	2	3	4	5	6	7	8	9	Totally Confident 10
CD1_03a …you can keep the fatigue caused by your disease from interfering with the things you want to do?	□	□	□	□	□	□	□	□	□	□
CD1_03b …you can keep the physical discomfort or pain of your disease from interfering with the things you want to do?	□	□	□	□	□	□	□	□	□	□
CD1_03c …you can keep the emotional distress caused by your disease from interfering with the things you want to do?	□	□	□	□	□	□	□	□	□	□
CD1_03d …you can keep any other symptoms or health problems you have from interfering with the things you want to do?	□	□	□	□	□	□	□	□	□	□
CD1_03e …you can do the different tasks and activities needed to manage your health condition so as to reduce you need to see a doctor?	□	□	□	□	□	□	□	□	□	□
CD1_03f …you can do things other than just taking medication to reduce how much your illness affects your everyday life?	□	□	□	□	□	□	□	□	□	□

END SCRIPT

Thank you for completing this survey!

Please note that these assessments will not be reviewed immediately. Everyone participating in this research study will receive a list of contacts if they want to speak to someone about any health concerns or abuse. Resources are shown below if you feel that you would like to talk with someone immediately for assistance.

National Suicide Prevention Lifeline: 1-800-273-8255
National Sexual Assault Hotline: 1-800-656-4673

Other options are to:

- Call your doctor's office
- Call 911 for emergency services
- Go to the nearest hospital emergency room.

Deleted: **More About You**¶
Below are some modifiable factors that likely influence blood pressure. These may not all apply to you, as you may already have excellent levels of these factors. ¶
¶
Physical activity:¶
The United States Office of Disease Prevention and Health Promotion 2008 Physical Activity Guidelines for Americans states that "Most health benefits occur with at least 150 minutes (2 hours and 30 minutes) a week of moderate intensity physical activity, such as brisk walking. Additional benefits occur with more physical activity. Both aerobic (endurance) and muscle-strengthening (resistance) physical activity are beneficial."¶
¶
RC1_01. How motivated are you to make changes to your physical activity, using a scale of one to ten, where one = definitely not ready to change, and 10 = definitely ready to change?¶
¶
Little intention of changing
...

Commented [SF13]: Removed readiness to change question block in order to reduce ppt burden.

Commented [SF14]: Moved questions on blood pressure and family history of hypertension to the in-person screener

Appendix G – NCCIH Data and Safety Monitoring Plan (DSMP) – full version, clean (v.1.0 – November 14, 2018 – NEW for UH3)

Data and Safety Monitoring Plan (DSMP)
Mindfulness-Based Blood Pressure Reduction: Stage 2a RCT

**Name of Sponsor:**	National Center for Complementary and Integrative Health
**Grant Number:**	5UH3AT009145-04
**Version Date:**	November 14, 2018
**Version Number:**	1.0

NCCIH Template Tool Revision History:

Version		
**Number**	**Date**	**Summary of Revisions Made:**
**1.0**	**21Feb2012**	**First approved version**
**2.0**	**13Apr2016**	**Added cover page, version numbers, and updated DSM Plan to be consistent with Protocol template**
**n/a**	**5Nov2018**	**Text in red was added by Willoughby Britton to update the NCCIH DSMP template to meet CONSORT Harms Criteria[1]**

UH3 DSMP Revision History

Version Number: v.1.0
Version Date: November 14, 2018
Notes: Original draft was created by data and safety monitoring expert, Willoughby Britton, PhD in cooperation with the MB-BP Principal Investigator (Loucks), Study Coordinator (Saadeh) and other research staff.

Version Number:
Version Date:
Summary of Revisions Made:

Version Number:
Version Date:
Summary of Revisions Made:

Version Number:
Version Date:
Summary of Revisions Made:

Version Number:
Version Date:
Summary of Revisions Made:

Table of Contents

UH3 DSMP Revision History 2
1 STUDY OVERVIEW 5
1.1 Purpose of Study 5
1.2 Adherence Statement 5
2 PROTOCOL AMENDMENTS 6
3 MULTI-SITE STUDIES 6
4 CONFIDENTIALITY 7
4.1 Protection of Subject Privacy 7
4.2 Confidentiality During Adverse Event (AE) Reporting 8
5 EXPECTED RISKS 8
6 ADVERSE EVENT/ UNANTICIPATED PROBLEMS 10
6.1 Definitions 10
6.1.1 Adverse Event (AE) 10
6.1.2 Unanticipated Problems (UP) 11
6.1.3 Serious Adverse Event (SAE) 11
6.2 Time Period and Frequency for Event Assessment and Follow-Up 12
6.2.1 Safety Check-ins 13
6.2.2 Mental health 15
6.2.3 Physical Health 16
6.2.4 Other 17
6.3 Characteristics of an Adverse Event 18
6.3.1 Relationship to Study Intervention 18
6.3.2 Expectedness of SAEs 19
6.3.3 Severity of Event 19
6.4 Reporting Procedures 19
6.4.1 Reporting for Multi-Center Trials 19
6.4.2 Unanticipated Problem Reporting 20
6.4.3 Adverse Event Reporting of Non-IND Studies 20
6.4.4 Adverse Event Reporting for IND Studies 21
6.4.5 Events of Special Interest (if applicable) 21
6.4.6 Reporting of Pregnancy 21
6.5 Halting Rules 21
7 QUALITY CONTROL AND QUALITY ASSURANCE 21

7.1 Subject Accrual and Compliance 21
7.1.1 Measurement and Reporting of Subject Accrual 21
7.1.2 Measurement and Reporting of Participant Adherence to Treatment Protocol 22
7.2 Justification of Sample Size 22
7.3 Stopping Rules 22
7.4 Designation of a Monitoring Committee 23
7.5 Safety Review Plan 23
7.6 Study Report Outline for DSMB Annual Reports 24
7.7 Submission of On-Site Monitoring/Audit and Inspection Reports 24
7.8 Table A – Data Review Summary 25
8 DATA HANDLING AND RECORD KEEPING 26
8.1 Data Management Responsibilities 26
8.2 Database Protection 26
8.3 Source Document Protection 26
8.4 Schedule and Content of Reports 26
9 INFORMED CONSENT 27
10 REPORTING CHANGES IN STUDY STATUS 27
11 SUPPLEMENTS/APPENDICES 27

1 STUDY OVERVIEW

1.1 Purpose of Study

The primary objectives of the study are:

1. ***Impacts of MB-BP on Primary Self-Regulation Targets:*** Identify the impacts of MB-BP *vs*. enhanced usual care on the primary self-regulation target, specifically an assay of self-related processes (MAIA), described in Table 1. We hypothesize that MB-BP will significantly improve the MAIA in directions of better self-regulation, compared to control.
    a. Secondary analyses will evaluate impacts on secondary self-regulation targets including an assay of emotion regulation (DERS), and cognitive processes (SART), described in Table 1.
    b. Exploratory analyses will evaluate engagement of MB-BP *vs*. enhanced usual care with triangulated self-regulation target assays described in **Table 1** such as emotion regulation and stress (Pittsburgh Stress Battery, Perceived Stress Scale), self-related processes (Heart Beat Detection Task, Interoceptive Awareness fMRI Task), and cognitive processes (Mindful Attention Awareness Scale). Measures such as the neuroimaging Interoceptive Awareness fMRI Task will replicate assays in the MINDFUL-PC study.
2. ***Self-Regulation Targets as Mediators of MB-BP Effect on Medical Regimen Adherence and Health Behavior Change:*** Evaluate the degree to which the engagement of MB-BP with self-regulation targets translates into improved prehypertension/hypertension medical regimen adherence, specifically for the Dietary Approaches to Stop Hypertension (DASH)-consistent diet. We hypothesize that MB-BP will increase the DASH diet score compared to control, in participants with low DASH diet adherence at baseline (DASH diet score <5.5), and that the self-regulation primary outcome in Aim 1 (i.e. MAIA) is a significant mediator.
3. ***Further develop an MB-BP therapist manual and training program, including procedures for training, supervising, and evaluating therapists.*** The PI will implement training he receives from the University of Bangor in the United Kingdom in May 2018 to implement the Mindfulness-Based Interventions Teacher Assessment Criteria (MBI-TAC) for MB-BP instructors, which is the most respected quantitative and qualitative tool developed to provide feedback for enhancing MBI teacher effectiveness, and establishing teacher certification.[2-4]

1.2 Adherence Statement

The Data Safety Monitoring Plan (DSMP) outlined below for UH3AT009145 will adhere to the protocol approved by the Brown University IRB.

Text in red was added by Willoughby Britton to update the NCCIH DSMP template v.2.0 to meet CONSORT Harms Criteria.[1]

2 PROTOCOL AMENDMENTS

All protocol amendments, other than minor administrative changes as defined by the NCCIH Guidance on Changes in Clinical Studies in Active Awards will be submitted in a prospective manner to NCCIH except when necessary to protect the safety, rights, or welfare of subjects. Upon submitting the proposed changes to NCCIH the revisions will also be communicated to the Data and Safety Monitoring Board (DSMB) and to the Brown University IRB.

3 MULTI-SITE STUDIES

There is only one study site, Brown University, carrying out the Mindfulness-Based Blood Pressure Reduction (MB-BP) Study protocol. The University of Massachusetts Medical School will conduct the fMRI imaging study that involves recruiting, screening, and scanning a subset of MB-BP study participants. The MB-BP Study and the fMRI imaging study have separate study protocols, but share the same DSMP. Brown University will be considered the Coordinating Center. However, since the fMRI study involves its own study protocol, each local PI and study coordinator will be responsible for the following:

•Design and develop the protocol and template informed consent documents for use at each collaborating institution

•Ensure informed consent is obtained and documented from each subject in compliance with federal regulations

•Store and/or manage data and data analysis activities

•Provide study specific training to the research personnel at the affiliated sites

•Develop and provide protocol specific case report forms for each affiliated site

•Ensure that affiliated sites are using the correct version of the protocol and consent document.

•Track subject enrollment

•Ensure that collaborating sites are utilizing quality control measures to assure data accuracy and completeness.

Coordinating Center Responsibilities: The Coordinating Center (Brown University) will perform the following tasks:

•Review and approve all documents used at affiliate sites

•Ascertain each protocol is reviewed and approved by the IRB at the collaborating institution prior to enrollment of subjects at that site

•Ensure that each collaborating institution holds an applicable OHRP approved Federal Wide Assurance (FWA)

•Collect and maintain critical documents from affiliated investigators, e.g. resume/CV, medical license, certification of completion of training, laboratory certifications and laboratory norms, signed COI disclosure forms (for studies involving investigator sponsored INDs and IDEs)

•Store and/or manage data and safety monitoring activities

•Maintain documentation of all affiliated sites IRB approvals for the protocol

•Coordinate randomization as applicable

•Register subjects and track subject enrollment

•Track, report and maintain documentation of all serious adverse events and unanticipated problems and disseminating the information to affiliate sites

•Provide periodic updates to affiliated investigators on subject enrollment, general study progress, and relevant scientific advances

•Assure that all relevant IRB correspondence (continuing review and amendments) and study status changes are communicated to all affiliate sites

4 CONFIDENTIALITY

4.1 Protection of Subject Privacy

Subject confidentiality is strictly held in trust by the investigators, study staff, and the sponsor(s) and their agents. This confidentiality is extended to cover testing of biological samples and genetic tests in addition to any study information relating to subjects.

The study protocol, documentation, data, and all other information generated will be held in strict confidence. No information concerning the study or the data will be released to any unauthorized third party without prior written approval of the sponsor.

The study monitor or other authorized representatives of the sponsor may inspect all study documents and records required to be maintained by the investigator, including but not limited to, medical records (office, clinic, or hospital) for the study subjects. The clinical study site will permit access to such records.

This research is covered by a Certificate of Confidentiality from the National Institutes of Health.

4.2 Confidentiality During Adverse Event (AE) Reporting

AE reports and annual summaries will not include subject or group-identifiable material. Each report will only include the identification code.

5 EXPECTED RISKS

Meditation-related risks: NCCIH states that meditation is generally safe for healthy people, but that adverse effects have also been reported. [5] Undesirable side effects and risks of meditation have been documented in more than 40 scientific reports [for reviews see [6-8]] and are listed in the Mindfulness-Based Intervention Guidelines.[9,10] More common, less serious side effects that have been reported by individuals within the context of MBIs or of individuals who are meditating less than an hour per day include: increased depression, anxiety or panic, re-experiencing of traumatic memories, dissociation, executive dysfunction, headaches/body pain and insomnia.[6,11-16] A few case reports of more serious side effects including mania, psychosis, and suicidality have been reported, mostly in the contexts of intensive retreats (>5 hrs/day) or in conjunction with pre-existing psychopathology.[6,8,9,17] The frequency of serious adverse effects in the context of MBIs is estimated to be less than 1%, although adequate estimates are not available.[18]

A number of actions have been taken to minimize meditation-related risks at different stages of the study. During the pre-enrollment stage, individuals with severe mental illness are excluded from the study and all risks are clearly communicated in the consent form. During treatment, meditations are relatively short and interspersed with dyads and reflections. Mindfulness homework assigned as part of the intervention is optional and is recommended to not exceed 1 hour per day. Teachers query participants about their experiences with meditation, and provide corrective feedback or modifications when needed. Developing strategies for working with physical and emotional discomfort is an explicit goal of the program. Because not all participants feel comfortable disclosing difficulties in class, an online “safety check-in” questionnaire will query meditation-related risks (see section 6.2.1). Dr. Ellen Flynn, a licensed psychiatrist, will be available to advise on any psychological events that occur, and provide referrals for treatment if needed. Additionally, Dr. Willoughby Britton will provide expert consult on safety monitoring and reporting, including providing DSMP specific training to research staff and investigators.

Assessment-related risks: Research subjects participating in this study may have feelings of loss of privacy from being contacted about participating in the study, and possible psychological distress caused by questions asked during the in-person and online questionnaires that bring up painful memories or feelings. However, the resulting

potential for injury to research subjects is judged to be minimal. We have already contacted and clinically evaluated thousands of participants from other studies such as the New England Family and Women's Health Initiative using similar assessment procedures to this study, with good responses from the participants.

Loss of confidentiality: Likelihood: rare. Minimization: Confidentiality will be maintained by using deidentifying data sets. All paper forms and data collection tools, including the informed consent forms, will be kept in a locked filing cabinet in a secure location. All electronic data files containing identifying information will be encrypted with a cloud-based software. Note that although these measures have been taken to protect participants' personal information, complete confidentiality cannot be guaranteed when transmitting information over the internet. All information obtained from participants will be accessible only to research staff.

Injury due to physical activities: It is possible that injuries could be sustained from (1) the gentle mindful movements (yoga), or (2) physical activities that participants engage in as a result of the intervention encouraging exploration of physical activity as a way to reduce blood pressure. *(1) Mindful movements:* Participants receive a handout during the orientation showing the yoga poses that will be offered during the course. They are encouraged to explore limits in their body related to movement, but not to go beyond those limits Participants are asked to listen to what their body is telling them more closely than what the mindful movement instructor is telling them. Modifications of poses are available, including for those limited to chairs or wheelchairs. Participants are encouraged to bring the handout of poses to their health care providers if they have any physical limitations, so that the providers can advise on which poses to do, and which to avoid. *(2) Physical activities:* Participants are encouraged to explore physical activities that promote strength and conditioning as a way to reduce blood pressure. As with the mindful movements, they are encouraged to explore limits in their body related to movement, but not to go beyond those limits. Participants are asked to listen to what their body is telling them more closely than what the mindful movement instructor is telling them. Furthermore, they are encouraged to ask their healthcare provider about advised physical activities if they have any physical limitations.

Risks associated with fMRI: The fMRI study will be conducted using a 3T MR scanner at UMass Medical School, which has been approved for research and clinical studies in children and adults by the FDA. Magnetic resonance (MR) technology does not use X-rays, but instead uses strong magnetic fields and radio waves. Individuals interested in participating in the fMRI study will complete a screening questionnaire to assess eligibility, including asking whether they have devices that can be affected by MRI or conditions (e.g., claustrophobia, body mass greater than 300 lbs) that prohibit the ability to be scanned. Participants are screened immediately prior to each MRI scan to ensure

participant safety. Significant risks also can arise if ferromagnetic materials are brought into the high magnetic field environment of the scanner and immediate vicinity, as they can become hazardous projectiles. These types of items are not permitted in the scanning area. The MR exams are painless, and except for the pulsating sounds, subjects will not be aware that MR scanning is taking place. With proper safety precautions in terms of the avoidance of metal objects, there are no known health risks associated with MRI. The safety of MRI is reflected in the fact that it is used in standard medical practice without the requirement for informed patient consent. Most people experience no ill effects from the magnetic field, but some report claustrophobia, dizziness, mild nausea, headaches, a metallic taste in their mouth, double vision, or a sensation of flashing lights. These symptoms are transient and resolve quickly after the subject exits the scanner. The technologist will be able to hear subjects at all times and subjects are free to end the procedure at any time. In rare cases, a very slight, uncomfortable tingling of the back due to the rapid switching of the magnetic field has been reported during certain types of scans. Subjects are asked to report this immediately so the scan can be changed to avoid this. Although these precautions will avoid all known risks associated with MR, this procedure may involve risks that are currently unknown. The scanner is noisy, but does not harm hearing. For comfort, subjects will be given earplugs to muffle the noise.

Risk of adverse events during the study: It is possible that some patients will have an adverse event during the study, including increased stress or anxiety. Participants with major mental health conditions, such as schizophrenia, history of psychosis, bipolar depression, suicidal ideation, borderline personality disorder, post-traumatic stress disorder, obsessive compulsive disorder, panic attacks, current alcohol or substance abuse, or an eating disorder are ineligible for the study. We expect risk of adverse events to be very low. For further discussion of AE and SAE monitoring and reporting refer to Section 6.2 below.

Impact statement: These risks are considered to be minimal and are addressed in the protocol and consent form.

6 ADVERSE EVENT/ UNANTICIPATED PROBLEMS

6.1 Definitions

6.1.1 ***Adverse Event (AE)***

An adverse event (AE) is any untoward medical occurrence in a subject during participation in the clinical study or with use of the experimental agent being studied. An adverse finding can include a sign, symptom, abnormal assessment (laboratory test

value, vital signs, electrocardiogram finding, etc.), or any combination of these regardless of relationship to participation in the study.

6.1.2 *Unanticipated Problems (UP)*

The Office for Human Research Protections (OHRP) considers unanticipated problems involving risks to subjects or others to include, in general, any incident, experience, or outcome that meets **all** of the following criteria:

- Unexpected in terms of nature, severity, or frequency given (a) the research procedures that are described in the protocol-related documents, such as the IRB-approved research protocol and informed consent document; and (b) the characteristics of the subject population being studied;
- Related or possibly related to participation in the research ("possibly related" means there is a reasonable possibility that the incident, experience, or outcome may have been caused by the procedures involved in the research); and
- Suggests that the research places subjects or others at a greater risk of harm (including physical, psychological, economic, or social harm) than was previously known or recognized.

6.1.3 *Serious Adverse Event (SAE)*

A serious adverse event (SAE) is one that meets one or more of the following criteria:

- Results in death
- Is life-threatening (places the subject at immediate risk of death from the event as it occurred)
- Results in inpatient hospitalization or prolongation of existing hospitalization
- Results in a persistent or significant disability or incapacity
- Results in a congenital anomaly or birth defect

An important medical event that may not result in death, be life threatening, or require hospitalization may be considered an SAE when, based upon appropriate medical judgment, the event may jeopardize the subject and may require medical or surgical intervention to prevent one of the outcomes listed in this definition.

6.2 Time Period and Frequency for Event Assessment and Follow-Up

Safety monitoring will occur continuously throughout the study using both active and passive monitoring methods outlined in the table and discussed in further detail below.

TIMELINE OF SAFETY-RELATED ASSESSMENTS					
**Measure**	**Mode of measurement**	**During treatment**	**Post-treatment**	**In-person Follow-ups**	
6.2.1 Safety-check-ins					
Tier 1<br>Safety check-in	Online self-report survey	Every 2 weeks<br>(week 2,4,6,8)	Week 10<br>(in-person)	Switch to monthly up through 6 month and then final check-in conducted at 1 year	
Tier 2<br>Safety check-in	Online self-report survey + phone call	Administered immediately following a positive report of AE/SAE in Tier 1 survey. Tier 2 phone follow up will be made upon receipt of the Tier 2 data.			
6.2.2 Mental health					
CESD-R (includes suicidal ideation)	In person<br>Self-report	Baseline (pre-treatment)	Week 10	6 months	
BAI	In person<br>Self-report	Baseline (pre-treatment)	Week 10	6 months	
6.2.3 Physical Health					
Blood pressure	In person<br>physiological	Screener and Baseline	Week 10	6 months	1 year
Cardiac abnormalities	In person<br>physiological	Baseline	Week 10	6 months	
Injuries	To be captured in the Safety check-in's outline above				
6.2.4 Other					
Participant-initiated	Passive monitoring	continuous			
Attrition	Online self-report	continuous			
fMRI Study	Passive monitoring	fMRI research staff will document and report any AE/SAE events discovered during the baseline and/or 10 week imaging visits			

All reported AEs, SAEs, and unanticipated problems will be recorded throughout the study using the data collection systems set up and detailed in the Manual of Operating Procedures (MOP).

The research staff will record all reportable events with start dates occurring any time after informed consent is obtained but no later than the final 1 year assessment. At each study visit, the research staff will inquire about the occurrence of AE/SAEs since the last visit (or time of most recent reporting). Events will be followed for outcome information until resolution or stabilization or until the grant funding ends.

6.2.1 ***Safety Check-ins***

All participants enrolled in the study, regardless of treatment allocation, will receive a two-tiered safety monitoring ‘check-in’ every 2 weeks during the treatment phase of the study; every month during months 3-6 of the follow-up phase; and at the final 1 year time point. The two-tiered system is designed to detect and follow up on AEs that are at least moderate in severity (interfere with ADL), and to minimize staff and participant burden that would otherwise occur if all mild events were queried and documented. Refer to Appendix A1 for the complete safety check-in surveys.

Tier 1 safety check-in survey:

Tier 1 of the safety check-in involves sending all active, enrolled participants an email (or placing a phone call from a research staff member, if no email provided) containing a link to a brief online survey that queries events with moderate or greater levels of severity. Specifically, study participants will be asked:

"During the past [two weeks / month / 6 months] have you experienced any of the following:

- change in prescribed or over the counter medication use (y/n),
- sustained any physical injuries (y/n),
- underwent any medical procedures (y/n),
- received any medical/clinical care outside of routine or preventative health care (y/n),
- been hospitalized (y/n)?"
- experienced physical or mental health symptoms that interfered with your daily activities (y/n)

Any participant who endorses one or more of the above tier 1 questions will automatically receive a tier 2 survey and follow-up phone call from study staff.

Tier-2 safety check-in Survey:

CONSORT-Harms guidelines[1] recommend using standardized and validated scales that assess treatment-specific symptoms, because they have more accurate detection rates than passive monitoring[14] or open-ended queries[15,16] and are low cost, low burden and require no special training to administer. In addition, patient or consumer-based self-reports (rather than face-to-face interviews or clinician ratings) are recommended for reporting sensitive or socially undesirable information such as negative reactions to treatment.[17-19] To accommodate these recommendations, and to create systematic query of treatment-specific negative events in all treatment arms, the Tier 2 survey specifically queries the most common meditation-related side effects (anxiety, depression, dissociation, flashbacks etc.) using patient-reported outcomes measurement information system (PROMIS) or NeuroQol items (or other validated scales of construct is not available), and PROMIS response options (never- very often).

Additionally, participants will be asked to provide further detail (i.e., date of onset, symptomology, circumstance surrounding the event, relatedness to the intervention, etc.) on the AE/SAE reported in the Tier 1 survey. Detail provided will be used to guide the Tier 2 phone calls made by research study staff.

Both the Tier-1 and Tier-2 Safety Check-in Surveys are located in Appendix A1.

Tier 2 Safety Check-in phone call: Any participant who indicates that he or she experienced an AE or SAE will then receive a follow up call from a trained research staff member. The purpose of the follow up call will be to further document the details of the AE/SAE, assess need for treatment modification, referrals and reporting.

Adverse Events form

Specifically, the staff member will use the Adverse Events Form and accompanying documents (i.e., the Severity Grading Tool, Relatedness Assessment Tool, and List of Previously-Reported Meditation Effects (all found in the Appendices)) in order to ascertain:

a) A description of the AE/SAE
b) Start and end dates
c) Severity grading (using Severity grading Tool)
d) Relationship to treatment (using Relatedness Assessment Tool + List of Previously-Reported Meditation Effects in the Appendix)
e) Action taken (if any)
f) Outcome (as assessed after 2 weeks or at next check-in time point)
g) Expectedness (Refer to List of Previously-Reported Meditation Effects in the Appendix)

h) Seriousness (See Section 6.1.3 of this document and/or Severity Grading Tool for definition of SAE; use Serious Adverse Events Form in Appendix if event is Serious)

Research staff members conducting the safety monitoring phone interviews will document the details of the AE/SAE, which will then be included in the participant file as well as in the annual Data Safety Monitoring Reports presented to the DSMB. Reporting procedures for AEs and SAEs related to the study will be followed including reporting all SAEs to the study PI and DSMB committee chair.

6.2.2 ***Mental health***

Participants assigned to any treatment group may experience mental health or suicidal ideation during the course of their study involvement. All study participants will be monitored for AEs and SAEs by study staff on a monthly basis until the time of their study completion. Additionally, participants will be asked to complete questionnaires about anxiety, depression and suicidal ideation, specifically the Beck Anxiety Inventory and the Center for Epidemiology Study Depression Scale Revised (CESD-R), at each of their in-person assessments, excluding the one year follow up. Dr. Flynn, a licensed psychiatrist with extensive experience evaluating research participants for clinical deterioration or suicidality, will serve as the study clinician.

Beck Anxiety Inventory (BA): If participant scores ≥26 on the Beck Anxiety Inventory, a safety flag will appear notifying the research assistant (RA) administering the assessment. The RA will then implement the MB-BP safety protocol, which is reviewed and approved by the Brown University IRB. Staff are trained on the safety protocol and a hard copy of the protocol is kept in an accessible location in the assessment office at all times.

Depressive Symptomatology: The CESD-R will be administered during the in-person assessment visits, and scores will be reviewed immediately upon completion of the in-person assessments.

1. Sadness (dysphoria): Question numbers 2,4, 6
2. Loss of Interest (anhedonia): Question numbers 8, 10
3. Appetite: Question numbers 1, 18
4. Sleep: Question numbers 5, 11, 19
5. Thinking / concentration: Question numbers 3, 20
6. Guilt (worthlessness): Question numbers 9, 17
7. Tired (fatigue): Question numbers 7, 16
8. Movement (agitation): Question numbers 12, 13
9. Suicidal ideation: Question numbers 14, 15

Participants are considered to meet criteria for major depressive episode if they have anhedonia or dysphoria nearly every day for the past two weeks, plus symptoms in an additional 4 DSM symptom groups noted as occurring nearly every day for the past two weeks. If participants meet criteria for major depressive episode, a safety flag will appear notifying the research assistant (RA) administering the assessment. The RA will then implement the MB-BP safety protocol, which is reviewed and approved by the Brown University IRB. Staff are trained on the safety protocol and a hard copy of the protocol is kept in an accessible location in the assessment office at all times.

If participants respond having any suicidal ideation (CES-D questions 14 or 15), staff will again be instructed to follow the IRB approved safety protocol.

6.2.3 ***Physical Health***

Possible atrial fibrillation detected during Heartbeat Detection Task: If possible atrial fibrillation is indicated by the Kardia Mobile device during the "Heart Beat Detection Task" a safety flag will appear notifying the research assistant (RA) administering the assessment. The RA will then implement the MB-BP safety protocol.

Out-of-range blood pressure readings: If during an in-person assessment the participants systolic blood pressure (SBP) and/or diastolic blood pressure (DBP) falls outside of the acceptable range outlined in the safety protocol, the RA will be notified and the IRB approved safety protocol will be implemented.

Injury due to physical activities: It is possible that injuries could be sustained from (1) the gentle mindful movements (yoga), or (2) physical activities that participants engage in as a result of the intervention encouraging exploration of physical activity as a way to reduce blood pressure.

(1) Mindful movements: Participants receive a handout during the orientation showing the yoga poses that will be offered during the course. They are encouraged to explore limits in their body related to movement, but not to go beyond those limits Participants are asked to listen to what their body is telling them more closely than what the mindful movement instructor is telling them. Modifications of poses are available, including for those limited to chairs or wheelchairs. Participants are encouraged to bring the handout of poses to their health care providers if they have any physical limitations, so that the providers can advise on which poses to do, and which to avoid.

(2) Physical activities: Participants are encouraged to explore physical activities that promote strength and conditioning as a way to reduce blood pressure. As with the mindful movements, they are encouraged to explore limits in their body

related to movement, but not to go beyond those limits. Participants are asked to listen to what their body is telling them more closely than what the mindful movement instructor is telling them. Furthermore, they are encouraged to ask their healthcare provider about advised physical activities if they have any physical limitations.

Note that adverse events related to physical injuries will be captured during the routine safety check-ins.

6.2.4 ***Other***

Participant Initiated (Passive monitoring): Participants are encouraged to contact meditation instructors and/or study staff if any physical or mental health symptoms arise or other study or meditation-related problems occur. Participants may report AEs at any time throughout the study. Events will be evaluated with the Adverse Events Form by study staff.

Attrition: Reasons for attrition are also an important source of AEs, but are rarely assessed adequately, as participants are unlikely to give honest answers if queried directly by study staff. To increase the accuracy of attrition reason reporting, participants will asked to complete a brief online Participant-initiated dropout reason survey (see Appendix A7).

Investigator-initiated withdrawals: A participant may also be withdrawn from the study and/or intervention by the researcher. In this case, the researcher or other study staff should complete the Attrition information form, and describe reasons for attrition.

fMRI Study Safety Monitoring: it is possible participants may experience or report an AE or SAE during their involvement with the fMRI Study. Research staff will use the Adverse Events Form and accompanying documents found in the Appendices to document all AE/SAE discovered at the time of involvement in the fMRI imaging study. The logged events will then be communicated to the Coordinating Center, so that they can be included in the participant file as well as in the annual Data and Safety Monitoring Reports presented to the DSMB. Reporting procedures for AEs and SAEs related to the study will be followed including reporting all SAEs to the study PI and DSMB committee chair.

6.3 Characteristics of an Adverse Event

6.3.1 *Relationship to Study Intervention*

According NIH guidelines, an event is considered related to the treatment (Possible, Probable, Definite) if: a) The event is known to occur with the study intervention; b) There is a temporal relationship between the intervention and event onset; c) The event abates when the intervention is discontinued; or d) The event reappears upon a re-challenge with the intervention. An event is considered Not Related (Unlikely, Not Related) if: a) There is no temporal relationship between the intervention and event onset or b) An alternate etiology has been established.

Relatedness to treatment will be assessed according to 8 causality and relatedness criteria that regulatory agencies such as the World Health Organization (WHO), the Federal Drug Administration (FDA), and the National Institutes of Health (NIH) use to make health policy decisions.[20-23] These criteria are designed to assess treatment-relatedness in individual cases in the absence of prospective or epidemiological (base rate) data. The 8 standard causality/relatedness criteria are 1) prior published reports, and expert judgment, 2) subjective attribution, 3) temporal proximity (challenge), or exacerbation, 4) consistency, 5) dose-response gradient 6) de-challenge, 7) re-challenge, and 8) specificity.[21,24-30]

The Relatedness Criteria Definitions table below provides detailed definitions, and the Relatedness Assessment Tool (Appendix A4) provides a systematic method for assessing relatedness in individual cases.

Relatedness Criteria Definitions	
1. Previous Reports/known effects and expert judgment	Symptom has been previously reported in association with meditation or judged to be caused by meditation by experts (meditation teachers/clinicians)
2. Subjective attribution	Participant thinks symptom was caused by meditation
3. Temporal proximity OR Exacerbation	Symptom occurred for the first time OR pre-existing symptoms got worse during/following treatment
4. Consistency	More than one occasion following meditation
5. Dose/gradient	Symptom was worse with higher dose of treatment
6. De-challenge	Decreased when stopped meditation
7. Re-challenge	Re-appeared or increased when meditation restarted
8. Specificity	Presence of plausible alternative cause: Something other than meditation (medical illness, life event, substances) could account for symptom (meditation played no causal role).

6.3.2 *Expectedness of SAEs*

The Study PI and Data Safety Monitoring Board (DSMB) will be responsible for determining whether an SAE is expected or unexpected. An adverse event will be considered unexpected if the nature, severity, or frequency of the event is not consistent with the risk information previously described for the intervention. Specifically, an event is considered expected if it appears on the List of Previously-Reported Meditation Effects in the Appendix A5.

6.3.3 *Severity of Event*

Severity is graded from 1 to 5 with corresponding description as mild, moderate, severe, very severe and death according to the Common Terminology Criteria for Adverse Events (CTCAE) and other sources for grading the severity of adverse events. Seriousness, which requires a different reporting procedure is defined according to the Office for Human Research Protections, *Guidance on Reviewing and Reporting Unanticipated Problems Involving Risks to Subjects or Others and Adverse Events.*[31-33] See Appendix A3 for the Severity Grading Tool.

6.4 Reporting Procedures

6.4.1 *Reporting for Multi-Center Trials*

There is only one study site, Brown University, carrying out the Mindfulness-Based Blood Pressure Reduction (MB-BP) Study protocol. The University of Massachusetts Medical School will conduct the fMRI imaging study that involves recruiting, screening, and scanning a subset of MB-BP study participants. The MB-BP Study and the fMRI imaging study have separate study protocols, but share the same DSMP.

The site research staff and local PI must immediately report to the coordinating center PI any serious adverse event, whether or not considered study related, including those listed in the protocol or investigator brochure and must include an assessment of whether there is a reasonable possibility that the study caused the event within 48 hours of PI awareness of the event.

They must also report any unanticipated problems within the same timeframe. The Site PI or research staff must also report any protocol deviations or violations to the coordinating center PI within 7 days of PI awareness. Participating centers must also submit all reports to their local IRB in accordance with their institutional policies.

All other AEs documented by the fMRI Study Research Staff will be reported to the coordinating center on an annual basis in the process of compiling the data for the annual report and in the annual AE summary which will be provided to NCCIH and to the DSMB.

6.4.2 ***Unanticipated Problem Reporting***

Incidents or events that meet the OHRP criteria for unanticipated problems require the creation and completion of an unanticipated problem report form. OHRP recommends that investigators include the following information when reporting an adverse event, or any other incident, experience, or outcome as an unanticipated problem to the IRB:

- Appropriate identifying information for the research protocol, such as the title, investigator's name, and the IRB project number;
- A detailed description of the adverse event, incident, experience, or outcome;
- An explanation of the basis for determining that the adverse event, incident, experience, or outcome represents an unanticipated problem;
- A description of any changes to the protocol or other corrective actions that have been taken or are proposed in response to the unanticipated problem.

To satisfy the requirement for prompt reporting, unanticipated problems will be reported using the following timeline:

- Unanticipated problems that are serious adverse events will be reported to the IRB, DSMB, and NCCIH within 7 days of the investigator becoming aware of the event.
- Any other unanticipated problem will be reported to the IRB, DSMB, and NCCIH within 14 days of the investigator becoming aware of the problem.

All unanticipated problems should be reported to appropriate institutional officials (as required by an institution's written reporting procedures), the supporting agency head (or designee), and OHRP within one month of the IRB's receipt of the report of the problem from the investigator.

6.4.3 ***Adverse Event Reporting of Non-IND Studies***

SAEs that are unanticipated, serious, and possibly related to the study intervention will be reported to the DSMB, IRB, and NCCIH in accordance with requirements.

- Unexpected fatal or life-threatening AEs related to the intervention will be reported to the NCCIH Program Officer, and DSMB within 3 days of the investigator becoming aware of the event. Other serious and unexpected AEs related to the intervention will be reported within 7 days.
- Anticipated or unrelated SAEs will be handled in a less urgent manner but will be reported to the DSMB, IRB, and other oversight organizations in accordance with their requirements. and will be reported to NCCIH on an annual basis.
- All other AEs documented during the course of the trial will be reported to NCCIH on an annual basis by way of inclusion in the annual report and in the annual AE

summary which will be provided to NCCIH and to the Independent Monitors. The DSMB Report will state that all AEs have been reviewed.

6.4.4 *Adverse Event Reporting for IND Studies*

Not Applicable.

6.4.5 *Events of Special Interest (if applicable)*

Not Applicable.

6.4.6 *Reporting of Pregnancy*

Not Applicable.

6.5 Halting Rules

This study will be stopped prior to its completion if: (1) the intervention is associated with adverse effects that call into question the safety of the intervention; (2) difficulty in study recruitment or retention will significantly impact the ability to evaluate the primary outcomes; (3) any new information becomes available during the trial that necessitates stopping the trial. The study may also be discontinued at any time by the IRB, the NCCIH, or other government agencies as part of their duties to ensure that research subjects are protected.

7 QUALITY CONTROL AND QUALITY ASSURANCE

There are a number of quality control measures established in the MB-BP study in order to help ensure data integrity and study credibility. For example, research staff administering in-person assessments are required to complete quality control assessment checklists to ensure that all aspects of the assessment are completed in a standardized and thorough manner. The checklist is reviewed by the Senior Project Coordinator as a method to identify potential issues with data quality and as a method for ongoing RA training. Other quality control checklists are utilized at each stage of the data collection periods to ensure that all participants in a given cohort are followed up with and to double check for the presence and completeness of data that was known to be collected.

All equipment utilized in the lab are calibrated at least quarterly. Logs of each calibration are kept. Research staff complete routine retraining on clinical skills, such as taking blood pressure and anthropometric readings.

7.1 Subject Accrual and Compliance

7.1.1 *Measurement and Reporting of Subject Accrual*

Review of the rate of subject accrual and compliance with inclusion/exclusion criteria will occur monthly during the recruitment phase to ensure that a sufficient number of participants are being enrolled, in keeping with proposed recruitment projections, and

that they meet eligibility criteria and fulfill the targeted ethnic diversity goals outlined in the grant proposal (Targeted/Planned Enrollment Table).

7.1.2 *Measurement and Reporting of Participant Adherence to Treatment Protocol*

Adherence to the prescribed MB-BP practices will be monitored through class attendance, practice logs and weekly health goals. Adherence data will be collected weekly during the course of the intervention. We discussed best practices to measure and monitor adherence to treatment protocol with leading meditation researchers, and meditation logs remain their recommended method at this time. Innovative approaches are being explored by Dr. Lazar and other investigators, including smartphone apps linked to audio homework files, with timers linked to data exports. However, these technologies are not yet ready for use. Data analyses will evaluate effect modification by adherence to the MB-BP practices.

7.2 Justification of Sample Size

The goal of the study is to evaluate the effect of the Mindfulness-Based Blood Pressure Reduction (MB-BP) intervention on the primary and secondary outcomes as compared to the enhanced usual care group. To do this we estimate needing to enroll 50 individuals per group for both the MB-BP intervention arm and the enhanced usual care arm. For details on the power calculations used refer to the study protocol. For the fMRI imaging study we estimate needing a final sample size of 48 (24 participants per group).

Our calculations assume drop-out rates will average around 10-15% across each group, with the control group exhibiting the highest rates of withdrawal and loss to follow up. These estimates are based on the Stage 1 and 2a MB-BP trials in the UH2 phase.

7.3 Stopping Rules

This study will be stopped prior to its completion if: (1) the intervention is associated with adverse effects that call into question the safety of the intervention; (2) difficulty in study recruitment or retention will significantly impact the ability to evaluate the study endpoints; (3) any new information becomes available during the trial that necessitates stopping the trial; or (4) other situations occur that might warrant stopping the trial.

The study may also be discontinued at any time by the IRB, the NCCIH, or other government agencies as part of their duties to ensure that research subjects are protected.

7.4 Designation of a Monitoring Committee

The Data and Safety Monitoring Board (DSMB) for this study is comprised of Drs. Donald Edmondson, Gaurav Choudhary, and Tao Liu. These individuals are not associated with this research project and work independently of the PI, Dr. Eric Loucks. Investigator. They are not part of the key personnel involved in this grant. No member of the Committee has collaborated or co-published with the PI within the past three years. They are qualified to review the patient safety data generated by this study because of their unique expertise in the areas of cardiology, psychology/psychiatry, epidemiology, and biostatistics.

The Data Safety Monitoring Committee will provide external monitoring, and will meet annually by phone, video conference, or in-person. They will be provided data annually in order to evaluate potential effects of the RCT on major outcomes (e.g. medical regimen adherence). Any serious adverse effects will be immediately reported to the principal investigator (Loucks) and the committee chair.

Oversight of the external Data and Safety Monitoring Committee will be conducted by the chair, Dr. Donald Edmondson, PhD, who is Associate Professor of Behavioral Medicine at Columbia University Medical Center. He is a Psychologist, and has extensive research experience in evaluating effects of stress and psychosocial factors on cardiovascular disease outcomes.

The Data and Safety Monitoring committee will also include a board-certified cardiologist, Dr. Gaurav Choudhary, and a biostatistian, Dr. Tao Liu. Dr. Choudhary, MD, is Associate Professor of Medicine and Associate Chief of Staff at the Providence VA Medical Center. He is a practicing clinical cardiologist with research in cardiology, and will be able to advise on clinical outcomes and any cardiovascular complications arising from the study, in addition to methodological concerns. Dr. Liu, PhD, is an Assistant Professor of Biostatistics at Brown University, experienced in clinical trials. He will receive preliminary analyses from the primary statistician, and will be given access to all data from the study, to evaluate any evidence of serious adverse effects or other concerns.

7.5 Safety Review Plan

Study progress and safety will be reviewed monthly (and more frequently if needed). Progress reports, including patient recruitment, retention/attrition, and AEs will be provided to the DSMB prior to each annual review. Annual Reports will be compiled and will include a list and summary of AEs/SAEs. Additionally, Annual Reports will address (1) whether AE rates are consistent with pre-study assumptions; (2) reason for dropouts from the study; (3) whether all participants met entry criteria; (4) whether continuation of the study is justified on the basis that additional data are needed to accomplish the stated aims of the study; and (5) conditions whereby the study might be terminated prematurely. Annual Reports will be sent to the DSMB and will be forwarded to NCCIH. The IRB and other applicable recipients will review progress of this study on an annual

basis.

The PI will also send copies of signed recommendations and comments from the DSMB or DSMB Chair to the NCCIH Program Officer within 1 month of each monitoring review.

7.6 Study Report Outline for DSMB Annual Reports

The study team will generate annual Study Reports for the DSMB and will provide information on the following study parameters:

I. Recruitment Summary Information
   - A. Recruitment Status
     - i. Enrollment by Year/Month
     - ii. Comparison of Targeted to Actual Enrollment
   - B. Retention Status
     - i. Overall Subject Status
     - ii. Individual Subject Status

II. Study Data Reports/Tables or Figures
   - A. General Information
     - i. Enrollment
     - ii. Demographic/Baseline Data
     - iii. Subject Status
   - B. Safety Assessment
     - i. Treatment Duration for All Subjects
     - ii. AE Data
       - a. Overall Listing
       - b. Specific Symptom Listing
       - c. Out-of-Range Laboratory Values
       - d. Out-of-Range Depression and Anxiety Scores
       - e. SAE Listing
       - f. Subject Deaths

Study Report tables will be generated only from aggregate (not by group assignment) baseline and aggregate safety data for the study population.

7.7 Submission of On-Site Monitoring/Audit and Inspection Reports

The IRB, DSMB, and NCCIH Program Officials will receive copies of all study monitoring/audit or inspection reports as required by individual institutation. For example, the NCCIH (Westat) monitoring report will be submitted to the IRB and DSMB (NCCIH does not require copies of Westat monitoring reports).

7.8 Table A – Data Review Summary

Data type	Frequency of review	Reviewer
Subject accrual (including compliance with protocol enrollment criteria)	Monthly	PI, Internal QA Reviewer
	Annually	DSMB
Status of all enrolled subjects, as of date of reporting	Monthly	PI, Internal QA Reviewer
	Annually	DSMB
Data entry quality control checks on 10% of charts	As needed based on frequency of data entry	QA Reviewer
Adherence data regarding study visits and intervention<br>AEs and rates (including out-of-range lab values)	Annually	DSMB
	Ongoing and in preparation for DSMB Report Creation	PI, Internal QA Reviewer
AEs and rates (including out-of-range lab values)<br>SAEs (unexpected and related)	Annually	DSMB
	Annually	NCCIH, FDA (If Applicable)
	Per occurrence	PI, DSMB Chair<br>NIH/NCCIH, FDA (if applicable)
SAEs (expected or unrelated)	Per Occurrence	PI, Internal QA Reviewer
SAEs (expected or unrelated)<br>Unanticipated Problems	Annually	DSMB, NIH/NCCIH
	Monthly	PI, Internal QA Reviewer
Unanticipated Problems	Per Policy	IRB, FDA (if applicable)

8 DATA HANDLING AND RECORD KEEPING

The investigators are responsible for ensuring the accuracy, completeness, legibility, and timeliness of the data reported. All source documents should be completed in a neat, legible manner to ensure accurate interpretation of data. The investigators will maintain adequate case histories of study subjects, including accurate case report forms (CRFs), and source documentation.

8.1 Data Management Responsibilities

Data collection and accurate documentation are the responsibility of the study staff under the supervision of the investigator. All source documents and laboratory reports must be reviewed by the study team and data entry staff, who will ensure that they are accurate and complete. Unanticipated problems and adverse events must be reviewed by the investigator or designee.

8.2 Database Protection

The clinical data will be de-identified but linked. Private information such as name, date of birth, and address for recontacting will be kept in a password protected, encrypted database on a different disk than the clinical data held by the Project Coordinator and used for the purposes of contacting participants. The principal investigator will only be given access to identifiable personal information for the purposes of patient safety or monitoring by the NIH, data safety monitoring boards or HIPPA compliance officer approved agents. Electronic communication with outside collaborators will involve only unidentifiable information.

Data management will be performed by downloading data at minimum every 2 weeks during active data collection periods, and assessing data for missingness and errors. Data will be maintained in password-protected Microsoft Excel Spreadsheets, and then exported using .csv functions for analysis in SAS software.

8.3 Source Document Protection

All source documents, including all paper and electronic records for all enrolled participants (i.e., case report forms, laboratory reports, etc.) will be maintained in a secure location. Original paper documents, even if data entered, will be maintained for the duration of the study. Data authenticator information will be recorded.

8.4 Schedule and Content of Reports

For detail on the schedule and content of study reports refer to the study protocol and manual of operating procedures.

9 INFORMED CONSENT

Informed consent is a process that is initiated prior to the individual agreeing to participate in the study and continues throughout study participation. Extensive discussion of risks and possible benefits of study participation will be provided to subjects and their families, if applicable. A consent form describing in detail the study procedures and risks will be given to the subject. Consent forms will be IRB-approved, and the subject is required to read and review the document or have the document read to him or her. The investigator or designee will explain the research study to the subject and answer any questions that may arise. The subject will sign the informed consent document prior to any study-related assessments or procedures. Subjects will be given the opportunity to discuss the study with their surrogates or think about it prior to agreeing to participate. They may withdraw consent at any time throughout the course of the study. A copy of the signed informed consent document will be offered to subjects for their records. The rights and welfare of the subjects will be protected by emphasizing to them that the quality of their clinical care will not be adversely affected if they decline to participate in this study.

The consent process will be documented in the clinical or research record.

To complete the informed consent process at the end of study participation, study staff will inform the subject when his/her participation has come to an end.

10 REPORTING CHANGES IN STUDY STATUS

During the funding of this study, any action by the IRB, the DSMB, or one of the study investigators that results in a temporary or permanent suspension of the study will be reported to the NCCIH Program Official within 5 business days of notification.

11 SUPPLEMENTS/APPENDICES

Note that minor modifications may be made to the individual documents listed in the appendices during the course of the study.

- A1_Tier 1+ 2 Safety Check Surveys
- A2_Adverse Event Form
- A3_Severity Grading Tool
- A4_Relatedness Assessment Tool
- A5_List of Previously-Reported Meditation Effects
- A6_Serious Adverse Events Form
- A7_Attrition Information Form

REFERENCES:

1. Ioannidis JP, Evans SJ, Gotzsche PC, et al. Better reporting of harms in randomized trials: an extension of the CONSORT statement. *Annals of internal medicine.* 2004;141(10):781-788.
2. Crane RS, Eames C, Kuyken W, et al. Development and validation of the mindfulness-based interventions - teaching assessment criteria (MBI:TAC). *Assessment.* 2013;20(6):681-688.
3. Crane RS, Kuyken W, Williams JM, Hastings RP, Cooper L, Fennell MJ. Competence in Teaching Mindfulness-Based Courses: Concepts, Development and Assessment. *Mindfulness (N Y).* 2012;3(1):76-84.
4. Crane RS, Brewer J, Feldman C, et al. What defines mindfulness-based programs? The warp and the weft. *Psychol Med.* 2017;47(6):990-999.
5. NCCIH. What the Science Says About Safety and Side Effects of Meditation. *National Cennter for Complementary and Intrgrative Medicine (NCCIH), National Insitutes of health (NIH)*. *https://nccih.nih.gov/health/meditation/overview.htm#hed52018*.
6. Lindahl JR, Fisher NE, Cooper DJ, Rosen RK, Britton WB. The varieties of contemplative experience: A mixed-methods study of meditation-related challenges in Western Buddhists. *PloS one.* 2017;12(5):e0176239.
7. Lustyk M, Chawla N, Nolan R, Marlatt G. Mindfulness Medtation Research: Issues of participant screening, safety procedures, and researcher training. *Advances in Mind-Body Medicine.* 2009;24(1):20-30.
8. Kuijpers H, van der Heijden F, Tuinier S, Verhoeven W. Meditation-induced psychosis. *Psychopathology.* 2007;40:461-464.
9. Kuyken W, Crane W, Williams JM. Mindfulness-Based Cognitive Therapy (MBCT) Implementation Resources. *Oxford University, University of Exeter, Bangor University.* 2012.
10. Santorelli S, Meleo-Meyer F, Koerbel L, et al. Mindfulness-Based Stress Reduction (MBSR) Authorized Curriculum Guide. *Center for Mindfulness in Medicine, Health Care, and Society (CFM), University of Massachusetts Medical School.* 2017.
11. Lomas T, Cartwright T, Edginton T, Ridge D. A qualitative summary of experiential challenges associated with meditation practice. *Mindfulness.* 2014:1-13.
12. Cebolla A, García-Campayo J, Demarzo M, Soler J. Side Effects of Meditation: A Survey Among Spanish Meditators International Sympsosium for Contemplative Studies (ISCSS); Oct 30-Nov 2, 2014, 2014; Boston, MA.
13. Yorston G. Mania precipitated by meditation: a case report and literature review. *Mental Health, Religion & Culture.* 2001;4:209-214.
14. Stephens M, Talbot J, Routledge P, eds. *The Detection of New Adverse Reactions.* 4th ed. London: Macmillan Reference; 1998.
15. Wallin J, Sjovall J. Detection of adverse drug reactions in a clinical trial using two types of questioning. *Clin Ther.* 1981;3(6):450-452.

16. Bent S, Padula A, Avins AL. Brief communication: Better ways to question patients about adverse medical events: a randomized, controlled trial. *Annals of internal medicine.* 2006;144(4):257-261.
17. Fowler F. Mode effcts in a survey of Medicare prostate surgery patients. *Public Opinion Quarterly.* 1998;62:29-46.
18. Turner C, Lessler J, George B, Hubbard M, Watt M. Effects of mode of administration and wording on reporting of drug use. In: Turner CF, Lessler JT, Gfroerer JC, eds. *Survey Measurement of Drug Use: Methodological Studies*. Washington, D.C: Government Printing Office. ; 1992:177-220.
19. Weissman JS, Schneider EC, Weingart SN, et al. Comparing patient-reported hospital adverse events with medical record review: do patients know something that hospitals do not? *Annals of internal medicine.* 2008;149(2):100-108.
20. NIH. Adverse Event and Serious Adverse Event Guidelines. *OHRP Guidance on Reviewing and Reporting Unanticipated Problems Involving Risks to Subjects or Others and Adverse Events, OHRP Guidance*. National Insitutes of Health (NIH): Office for Human Research Protections, U.S. Department of health and Human Servcies; 2016.
21. Agbabiaka TB, Savovic J, Ernst E. Methods for causality assessment of adverse drug reactions: a systematic review. *Drug safety.* 2008;31(1):21-37.
22. Turner WM. The Food and Drug Administration algorithm. Special workshop--regulatory. *Drug information journal.* 1984;18(3-4):259-266.
23. WHO. *The use of the WHO-UMC system for standardized case causaility assessment.* who-umc.org: World Health Organization (WHO), Uppsala Monitoring Centre 2016.
24. Naranjo CA. A clinical pharmacologic perspective on the detection and assessment of adverse drug reactions. *Drug information journal.* 1986;20(4):387-393.
25. Naranjo CA, Busto U, Sellers EM. Difficulties in assessing adverse drug reactions in clinical trials. *Progress in neuro-psychopharmacology & biological psychiatry.* 1982;6(4-6):651-657.
26. Naranjo CA, Busto U, Sellers EM, et al. A method for estimating the probability of adverse drug reactions. *Clinical pharmacology and therapeutics.* 1981;30(2):239-245.
27. Hill AB. The Environment and Disease: Association or Causation? *Proceedings of the Royal Society of Medicine.* 1965;58:295-300.
28. Hill AB. The environment and disease: association or causation? *Journal of the Royal Society of Medicine.* 2015;108(1):32-37.
29. Gallagher RM, Kirkham JJ, Mason JR, et al. Development and inter-rater reliability of the Liverpool adverse drug reaction causality assessment tool. *PloS one.* 2011;6(12):e28096.
30. Theophile H, Arimone Y, Miremont-Salame G, et al. Comparison of three methods (consensual expert judgement, algorithmic and probabilistic approaches) of causality assessment of adverse drug reactions: an assessment using reports made to a French pharmacovigilance centre. *Drug safety.* 2010;33(11):1045-1054.

31. Linden M. How to define, find and classify side effects in psychotherapy: from unwanted events to adverse treatment reactions. *Clinical psychology & psychotherapy.* 2013;20(4):286-296.
32. OHRP. *Guidance on Reviewing and Reporting Unanticipated Problems Involving Risks to Subjects or Others and Adverse Events.* Office for Human Research Protections, US Department of Health and Human Services; 2007.
33. NCI. Common Terminology Criteria for Adverse Events (CTCAE) v5.0. *National Cancer Insititute, NIH, US Department of Health and Human Services.* 2017.

Appendix A1

Tier 1 and Tier 2 Safety Check Surveys

TIER 1 SAFETY-CHECK SURVEY		
**In the last { 2 weeks / month / 6 months }, have you have experienced the following:**		
	**NO**	**YES**
1. Change in prescribed or over the counter medication use		
2. Sustained any physical injuries		
3. Underwent any medical procedures		
4. Received any medical/clinical care outside of routine or preventative health care		
5. Been hospitalized		
6. Experienced physical or mental health symptoms that interfered with your daily activities		

IF YES TO ANY OF THE ABOVE, INITIATE:

- TIER 2 SURVEY
- FOLLOW-UP PHONE CALL

TIER 2 SAFETY CHECK-IN, Part 1

1. *If YES to Tier 1, Q1 (change in medication)* – You indicated that you recently made a change to your prescribed and/or over the counter medication use….

   1a. Please describe the changes that were made including providing information on the medications added, removed, or altered (i.e., names of medications, dosage, frequency, etc.).

   1b. When did the change in medication take place?

   1c. Please describe the circumstances / reasons for the change.

2. *If YES to Tier 1, Q2 (physical injury)* – You indicated that you recently sustained a physical injury….

   2a. Please describe the injury.

   2b. When were you injured?

   2c. How were you injured?

   2d. Did the injury include:
   Broken bone(s) – Y/N
   Lacerations requiring stiches – Y/N
   Concussion – Y/N

3. *If YES to Tier 1, Q3 (medical procedure)* – You indicated that you recently underwent a medical procedure…

   3a. Please describe the medical procedure.

   3b. When did it take place?

   3c. What was the procedure for?

4. *If YES to Tier 1, Q4 (non-preventive medical care)* – You indicated that you recently received medical / clinical care outside of routine or preventative health care…

   4a. Please describe the care that you received.

   4b. When did you receive the care?

   4c. Please describe the circumstances / reasons for receiving the care.

5. *If YES to Tier 1, Q5 (hospitalization)* – You indicated that you recently were hospitalized…

    5a. Where were you hospitalized?

    5b. What were the dates of your hospitalization?

    5c. Please describe the circumstances / reasons for your hospitalization.

6. *If YES to Tier 1, Q6 (physical/mental symptoms interfering with ADLs)* – You indicated that you recently experienced physical and/or mental health symptoms that interfered with your daily activities…

    3a. Please describe the symptoms you experienced and how they interfered with your daily activities.

    3b. When did the symptoms begin?

    3c. When did the symptoms end or are you still experiencing them?

    3d. To what extent are they interfering with your daily activities, would you say, {INSERT LIKERT SCALE HERE}

**TIER 2 SAFETY CHECK-IN, Part 2** (Britton and Lindahl 2018)					
**In the last { 2 weeks / month / 6 months }, please indicate how often you have experienced the following:**					
	**Never**	**Rarely** (once per week)	**Sometimes (**2-3 times per week)	**Often** (once a day)	**Very often** (several times/day)
1. I had trouble thinking clearly and/or making decisions					
2. I felt anxious					
3. I experienced repeated, disturbing memories, thoughts, or images of a stressful experience from the past					
4. I was bothered by little things					
5. I had trouble enjoying things that I used to enjoy					
6. I felt distant or cut off from other people					
7. I had difficulty sleeping					
8. I experienced headaches and/or body pain					
9. My hearing has become very sensitive					
10. Feeling disconnected from everything around you					
11. Other significant symptoms (describe) ________________________________					
Scale information: Britton, W.B., and Lindahl, J.R. (2018)<br><br>The scale represents the 10 most common meditation-related symptoms from the Varieties of Contemplative Experience Phenomenology Codebook (Lindahl et al 2017 *PLOS One*), as replicated in an NIH–funded MBI clinical trial (Britton, in prep), assessed by PROMIS, Neuro-QoL, and other validated questionnaire items, which were chosen by a modified Delphi method.					

Thank you for completing this survey! Your safety is important to us. You may receive a phone call from a trained staff member to follow up with you regarding these event(s).

However, please note that your responses may not be reviewed immediately. Everyone participating in this research study will receive a list of contacts if they want to speak to someone about any health concerns or abuse. Resources are shown below if you feel that you would like to talk with someone immediately for assistance.

National Suicide Prevention Lifeline: 1-800-273-8255
National Sexual Assault Hotline: 1-800-656-4673

Other options are to:

- Call your doctor's office
- Call 911 for emergency services
- Go to the nearest hospital emergency room

Appendix A2

Adverse Event Form

Adverse Event Form

STUDY NAME

**Site Name:____________________________**<br>**Pt_ID:__________________________**	**This form is cumulative and captures adverse events of a single participant throughout the study.**

Severity	Study Intervention Relationship	Action Taken Regarding Study Intervention	Outcome of AE	Expected	Serious Adverse Event (SAE)
1 = Mild<br>2 = Moderate<br>3 = Severe<br>4 = Very Severe<br>5= Death	0 = Not related<br>1 = Unlikely related<br>2 = Possibly related<br>3 = Probably related<br>4 = Definitely related	0 = None<br>1 = Treatment modification<br>2 = Medical Intervention<br>3 = Hospitalization<br>4 = Treatment discontinued<br>5 = Other	1 = Resolved<br>2 = Recovered with minor sequelae<br>3 = Recovered with major sequelae<br>4 = Ongoing/Continuing treatment<br>5 = Condition worsening<br>6 = Death<br>7 = Unknown	1 = Yes<br>2 = No	1 = Yes<br>2 = No (if yes, complete SAE form)

At end of study only: Check this box if participant had no adverse events ☐ **None**

Adverse Event	Start Date	Stop Date	Severity	Relationship	Action Taken	Outcome of AE	Expected?	SAE?

Appendix A3

Severity Grading Tool

SEVERITY GRADING TOOL

Grade	Description	Detailed Criteria (Linden, 2013; NCI, 2017; OHRP, 2007)
1	**Mild**	• May include transient distress and discomfort, but not enough to cause significant impairment, change in behavior or countermeasures.<br>• Transient or short duration (usually < than 1 week)<br>• Resolved on its own; no intervention needed<br>• No consequences
2	**Moderate**	• Minimal, local or noninvasive intervention indicated (individual meeting with meditation teacher)<br>• Significant distress or discomfort that involves:<br>o Countermeasures (non-prescription medicine) OR<br>o Change in behavior (cancelling plans, decreasing or discontinuing treatment) OR<br>o Impairment in at least one domain of functioning[a] or activities of daily living (ADL)[b] (25%-50% reduction from usual level)
3	**Severe**	• Clinically/medically significant but not immediately life-threatening; hospitalization or prolongation of hospitalization indicated; disabling; (source: CTCAE)<br>• Health professional/clinical attention needed. Can be outpatient or inpatient.<br>• Significant impairment in at least one domain of functioning[a], activities of daily living[b] or self-care[c] (>50% reduction in usual level)
4	**Very severe**	• Life-threatening consequences; urgent intervention indicated (source: CTCAE).<br>• Includes suicidal ideation or attempt<br>• If psychological in nature: requires in-patient hospitalization
5	**Death**	• Death
**SERIOUSNESS (requires different reporting process)**		
	**Serious**	• Grades 4 + 5 are serious; Grade 3 may be serious<br>• Results in death<br>• Is life-threatening<br>• Requires hospitalization or prolongs existing stay<br>• Results in congenital anomaly<br>• Causes permanent disability or requires medical/surgical intervention to prevent permanent disability or any of the above.

[a] Domains of functioning = occupational (work), educational (school), social (friendships, community), role (parenting, spouse), cognitive (memory, decision-making).
[b] Activities of Daily Living (ADL) = preparing meals, shopping for groceries or clothes, using the telephone, managing money, etc.
[c] Self care = bathing, dressing and undressing, feeding self, using the toilet, taking medications, and not bedridden.

OHRP. (2007). *Guidance on Reviewing and Reporting Unanticipated Problems Involving Risks to Subjects or Others and Adverse Events*: Office for Human Research Protections, US Department of Health and Human Services.

NCI. (2017). Common Terminology Criteria for Adverse Events (CTCAE) v5.0. *National Cancer Institute, NIH, U.S. Department of Health and Human Services*.

Linden, M. (2013). How to define, find and classify side effects in psychotherapy: from unwanted events to adverse treatment reactions. *Clin Psychol Psychother, 20*(4), 286-296.

Appendix A4

Relatedness Assessment Tool

RELATEDNESS ASSESSMENT TOOL			
	**YES**	**NO**	**unsure**
1. Are the symptoms on the list of known meditation-related experiences?<br>[See attached list of previously reported effects]	**+1**<br><br>[expected]	**0**<br><br>[unexpected]	**+0.5**
2. Do you think these experiences were caused in whole or in part by the study treatment? (Do you think meditation played a causal role?)	**+1**	**0**	**+0.5**
3a. Did the experiences appear for the FIRST time during or following exposure to the study treatment? (Did you ever experience this symptom before the study?= no)<br><br>OR<br><br>3b. Did pre-existing experiences or conditions increase or get worse after the study treatment?	**+1**	**0**	**+0.5**
4. Have these experiences occurred during/following exposure to the study treatment on more than one occasion?	**+1**	**0**	**+0.5**
5. Did the symptom get worse with a higher dose of treatment?	**+1**	**0**	**+0.5**
6. Did these experiences disappear or lessen after you stopped or decreased participating in the study treatment?	**+1**	**0**	**+0.5**
7. Did the experiences reappear or get worse when you started engaging with the study treatment again or increased the amount?	**+1**	**0**	**+0.5**
8. Are there alternative causes (other than exposure to the study treatment) that could have caused the experience *in its entirety*?	**-1**	**+1**	**+0.5**
**TOTAL (sum 1-8)**			
**Scoring:**<br>Not related = -1 to 0<br>Unlikely related = 1<br>Possibly related = 2 OR includes plausible alternative cause (#8)<br>Probably related =3-4<br>Definitely related =5-7			
**Sources:** (Agbabiaka et al., 2008; Gallagher et al., 2011; Hill, 1965, 2015; Naranjo, 1986; Naranjo et al., 1982; Naranjo et al., 1981; Theophile et al., 2010).			

Appendix A5

List of Previously-Reported Meditation Effects

LIST OF PREVIOUSLY-REPORTED MEDITATION EFFECTS

(From Lindahl et al 2017 The Varieties of Contemplative Experience, *PLOS ONE*)

**Affective Domain** (13 Categories)	**Description**
Affective Flattening, Emotional Detachment, or Alexithymia	A narrowed or diminished affective range, a lack of affective charge, and/or an inability to identify/distinguish emotions.
Affective Lability	Rapid shifts in mood, mood swings, a increased range of emotions, or strong, unwarranted reactions to situations.
Agitation or Irritability	An agitated or irritable mood, possibly accompanied by restlessness, distractibility or uneasiness.
Change in Doubt, Faith, Trust, or Commitment	Changes (increase or decrease) in doubt, faith, trust or commitment in relation to religious doctrines, practices, goals, community or in relation to oneself in any dimension of life, such as self-confidence.
Crying or Laughing	Crying and laughing, and associated vocalizations.
Depression, Dysphoria, or Grief	Low, depressed, or sad moods, usually coupled with physical and behavioral manifestations that may or may not affect normal functioning.
Empathic or Affiliative Changes	Increased or decreased empathic connection to other people or to environmental stimuli.
Fear, Anxiety, Panic, or Paranoia	Feelings of fright or distress--with or without an external referent--and their corresponding physiological and behavior responses.
Positive Affect	A state of positive or elevated mood or energy level, ranging on a continuum from low to high arousal.
Rage, Anger, or Aggression	Feelings of intense displeasure or a retaliatory response, often caused by some adverse stimulus provoking an uncomfortable emotion.
Re-experiencing of Traumatic Memories or Affect Without Recollection	Either a recollection of some past traumatic event in the subject's life that may or may not have been repressed, and which is generally associated with strong emotions, or the upwelling of strong emotions without any corresponding memory, content, thought or other identifiable stimulus.
Self-Conscious Emotions	Emotions relating to one's sense of self and identity, as well as the awareness of reactions of others to oneself, whether real or imagined.
Suicidality	Affect-driven ideation concerning wanting to die, not wanting to continue with life, wishing to no longer being alive, thinking about taking one's own life, or thinking about or making specific plans for taking one's own life.

**Cognitive Domain** (10 Categories)	**Description**
Change in Executive Functioning	Either an inability to perform cognitive functions of decision making, concentration, and memory that the person used to be able to perform, or an enhanced ability in these domains of executive functioning.
Change in Worldview	A shift in ways of thinking about the nature of self or reality, including a change in understanding or confusion about the nature of self or reality.
Clarity	Reports of clarity or lucidity as a mental state, quality of attention, or quality of consciousness, in which there is a heightened cognition of relevant stimuli and a diminished interference from non-relevant stimuli.
Delusional, Irrational, or Paranormal Beliefs	Holding with conviction and being influenced by one or more beliefs despite evidence to the contrary. Ascriptions of significance or meaning that are later disregarded or that might seem unusual or concerning to members of the practitioner's broader culture or particular subculture. Attributions of paranormal agency, origin, or explanation for cognitive experiences.
Disintegration of Conceptual Meaning Structures	Percepts arise but are processed without their associated conceptual meaning, resulting in an inability to form conceptual representations of the perceptual world.
Increased Cognitive Processing	Primarily a cognitive change in thought amount or speed of cognitive processing, though the increase in processing often coincides with a decrease in sensory gating that leads to the impression of taking in or processing more perceptual information than usual.
Mental Stillness	An state in which there are few identifiable thoughts, a perceived absence of thought, or a poor awareness about the thinking process in general.
Meta-Cognition	Meta-cognition, or meta-awareness, refers to an explicit knowledge of the content of thoughts or the thinking process. Meta-cognition can also entail a higher-order cognition of processes in other domains of experience, such affective, perceptual, somatic or sense of self.
Scrupulosity	Obsessive thinking, specifically about moral or religious issues and behaviors.
Vivid Imagery	An experience of intense, vivid and/or clear thoughts or mental images that arise involuntarily, or a report of an increased ability to visualize.
**Conative Domain** (3 Categories)	**Description**
Anhedonia or Avolition	Anhedonia is the inability to experience pleasure in activities previously found pleasurable. Avolition is the lack of drive or motivation to pursue goals previously valued as meaningful.
Change in Effort or Striving	The degree or intensity of attempts at pursuing something valued-as-good or as a means to a valued end. Effort may be mental, physical, or emotional.
Change in Motivation or Goal	The reasons, drives, and needs behind a practitioner's actions, which influence or determine their behavior, as well as their expectations concerning a particular behavior.

**Perceptual Domain**<br>(7 Categories)	**Description**
Derealization	Surroundings are perceived as strange, unreal, or dreamlike, or perception is experienced as mediated by a fog, a lens, or some other filter that results in feeling cut off from the world.
Dissolution of Objects or Phenomena	The dissolving or complete disappearance of visual objects or the entire visual field.
Distortions in Time or Space	An alteration in the subjective experience of spatial boundaries or relations and/or temporal causality or sequencing.
Hallucinations, Visions, or Illusions	A hallucination is an experience of a percept that is not externally stimulated, is not shared by others, and is not taken to be veridical. When a visual percept that is not shared by others is taken to be veridical, it is a vision. An illusion involves a percept that is distorted, changed, or has features added to the raw percept.
Perceptual Hypersensitivity	Unusual or atypical sensitivity to certain frequencies or volumes of sound (hyperacusis), to color (hyperchromia), to visual details, to light, to taste, to smell, or to embodiment.
Somatosensory Changes	A change in proprioceptive information that affects one's perception of relative positions or dimensions of body parts or the body more generally.
Visual Lights	Experience of a light or lights in field of vision that are vivid but not the result of external stimuli.
**Sense of Self Domain**<br>(6 Categories)	**Description**
Change in Self-Other or Self-World Boundaries	Expansion beyond or distortions in the typical sense of where the boundaries between self and other or self and world are delineated.
Change in Narrative Self	A report of a change in how the practitioner conceives of himself or herself as a person. Or, a change in the content of or their perspective on their story or personal identity.
Change in Sense of Embodiment	Feeling of being disembodied, located outside or at a distance from one's body, or located in an unusual location within one's body schema.
Loss of Sense of Agency	A loss of a sense of ownership or sense of control over one's actions.
Loss of Sense of Ownership	A loss of the usual sense of owning one's thoughts, body sensations, emotions, and/or memories.
Loss of Sense of Basic Self	A loss of the sense of existing, of being a self, or of having a self.
**Social Domain**<br>(5 Categories)	**Description**
Change in Relationship to Meditation Community	Changes in relationship with the meditation community (*Sangha*), whether increasing or decreasing degrees of affiliation with the community of teacher(s) and other practitioners.
Increased Sociality	Increased extraversion, social contact, friendships or other behavioral manifestations indicating an increased valuing of social engagement.

Integration Following Retreat or Intensive Practice	A destabilizing transition from intensive formal practice to informal practice, daily life, or life circumstances.
Occupational Impairment	An impaired ability to perform in an occupational environment.
Social Impairment	Behaviors indicative of a change in relationship to social networks or social situations that inhibits ordinary or desired functioning or level of engagement.
**Somatic Domain** (15 Categories)	**Description**
Appetitive or Weight Changes	Decreased or increased appetite, weight loss or gain.
Breathing Changes	Altered respiration rates that may manifest as a temporary cessation, or speeding up or slowing down of breathing.
Cardiac Changes	Irregular heartbeat, heart palpitations, or other significant irregularities.
Dizziness or Syncope	Dizziness, vertigo (feeling one is spinning or off-balance), lightheadedness (feeling one is about to faint), or syncope (a brief loss of consciousness and muscle strength, commonly called fainting, passing out or blacking out).
Fatigue or Weakness	A feeling of exhaustion, fatigue or weakness (general or localized).
Gastrointestinal Distress or Nausea	Gastrointestinal problems including (but not limited to) diarrhea, bloating, cramping, nausea and vomiting.
Headaches or Head Pressure	Ache, sharp pain, or pressure in the region of the head or neck.
Involuntary Movements	A motor movement usually under voluntary control that occurs without a conscious decision for movement.
Pain	Pain is an unpleasant physical sensation, either diffuse or acute, and lasting for variable amounts of time.
Parasomnias	Nightmares, vivid dreams, sleep paralysis or the alleviation of these symptoms.
Pressure, Tension or Release of Pressure, Tension	Bodily pressure or tension, or release of bodily pressure or tension, that can vary according to location (general or specific), intensity, or length of time.
Sexuality-Related Changes	Hypersexuality (very frequent or suddenly increased sexual urges or activity) or hyposexuality (notably decreased sexual urges or activity).
Sleep Changes	Changes in sleep amount, sleep need, or sleep depth.
Somatic Energy	A type of sensation moving throughout the body or throughout a body area described with language of vibration, energy, current, or other related metaphors.
Thermal Changes	Changes associated with heat or cold, whether a general change in sense of body temperature or localized to a specific body area.

Appendix A6

Serious Adverse Events Form

National Center for Complementary and Integrative Health

Serious Adverse Event (SAE)
Report Form

STUDY NAME

Protocol Number:____________________________

Site Name:______________________________

Pt ID:____________________________

Date Participant Reported:

__ __ / __ __ __ / __ __ __ __
d d m m m y y y y

1. SAE onset date: ___ ___ / ___ ___ ___ / ___ ___ ___ ___
   d d m m m y y y y

2. SAE stop date: ___ ___ / ___ ___ ___ / ___ ___ ___ ___
   d d m m m y y y y

3. Location of SAE:____________

4. Was this an unexpected adverse event? ☐ Yes ☐ No

5. Brief description of participants with no personal identifiers:

   Sex: ☐ F ☐ M Age: ________

   Diagnosis for study participation:____

   ____

6. Brief description of the nature of the SAE (attach description if more space is needed):

   ____

   ____

7. Category of the SAE:

   ☐ Date of death ___/_____/_______ (dd/mmm/yyyy)

   ☐ Life threatening

   ☐ Hospitalization – initial or prolonged

   ☐ Disability/incapacity

   ☐ Congenital anomaly/birth defect

   ☐ Required intervention to prevent permanent impairment

   ☐ Other:________________________________

8. Intervention type:

   ☐ Medication or nutritional supplement (specify):________________________________

   ☐ Device (specify):____

   ☐ Surgery (specify):____

   ☐ Behavioral/lifestyle (specify):____

9. Relationship of event to intervention:

- ☐ Unrelated (clearly not related to the intervention)
- ☐ Possible (may be related to intervention)
- ☐ Definite (clearly related to intervention)

10. Was study intervention discontinued due to event? ☐ Yes ☐ No

11. What medications or other steps were taken to treat the SAE?

____

12. List any relevant tests, laboratory data, and history, including preexisting medical conditions:

____

13. Type of report:

- ☐ Initial
- ☐ Follow-up
- ☐ Final

Signature of principal investigator: ________________________________ Date: _________________

Appendix A7

Attrition Information Form

ATTRITION INFORMATION FORM:

**ID#:**	**Dropout date:**
**Reason for participant dropout (choose 1 and document circumstances)**	
	Participant-initiated; Reason(s)
	Researcher-initiated; Reason(s)
	Unknown

Participant-initiated dropout reason survey (administered online):

**Please indicate how much each of the following influences your decision to leave the study**			
	**Not at all**	**Somewhat**	**Very Much**
1) Logistics (e.g. scheduling and time commitment)<br>Please describe:___________________________			
2) Personal or family reasons (e.g. other commitments related to work/family)<br>Please describe:___________________________			
3) Dissatisfied with treatment<br>Please describe:___________________________			
4) Poor mental health<br>Please describe:___________________________			
5) Poor physical health<br>Please describe:___________________________			
6) Other<br>Please describe:___________________________			

Research-initiated dropout reason survey (for researcher/staff)

Please describe reason(s) why participant was withdrawn from the study and/or analysis:

Appendix H – NCCIH Data and Safety Monitoring Plan (DSMP) – *ANNOTATED to highlight revisions*

(v.1.0 – November 14, 2018 – NEW for UH3)

Data and Safety Monitoring Plan (DSMP)

Mindfulness-Based Blood Pressure Reduction: Stage 2a RCT

**Name of Sponsor:**	National Center for Complementary and Integrative Health
**Grant Number:**	5UH3AT009145-04
**Version Date:**	November 14, 2018
**Version Number:**	1.0

NCCIH Template Tool Revision History:

Version		
**Number**	**Date**	**Summary of Revisions Made:**
**1.0**	**21Feb2012**	**First approved version**
**2.0**	**13Apr2016**	**Added cover page, version numbers, and updated DSM Plan to be consistent with Protocol template**
**n/a**	**5Nov2018**	**Text in red was added by Willoughby Britton to update the NCCIH DSMP template to meet CONSORT Harms Criteria[1]**

UH3 DSMP Revision History

Version Number: v.1.0
Version Date: November 14, 2018
Notes: Original draft was created by data and safety monitoring expert, Willoughby Britton, PhD in cooperation with the MB-BP Principal Investigator (Loucks), Study Coordinator (Saadeh) and other research staff.

Version Number:
Version Date:
Summary of Revisions Made:

Version Number:
Version Date:
Summary of Revisions Made:

Version Number:
Version Date:
Summary of Revisions Made:

Version Number:
Version Date:
Summary of Revisions Made:

Table of Contents

UH3 DSMP Revision History 2

1 STUDY OVERVIEW 5

1.1 Purpose of Study 5

1.2 Adherence Statement 5

2 PROTOCOL AMENDMENTS 6

3 MULTI-SITE STUDIES 6

4 CONFIDENTIALITY 7

4.1 Protection of Subject Privacy 7

4.2 Confidentiality During Adverse Event (AE) Reporting 8

5 EXPECTED RISKS 8

6 ADVERSE EVENT/ UNANTICIPATED PROBLEMS 10

6.1 Definitions 10

6.1.1 Adverse Event (AE) 10

6.1.2 Unanticipated Problems (UP) 11

6.1.3 Serious Adverse Event (SAE) 11

6.2 Time Period and Frequency for Event Assessment and Follow-Up 12

6.2.1 Safety Check-ins 13

6.2.2 Mental health 15

6.2.3 Physical Health 16

6.2.4 Other 17

6.3 Characteristics of an Adverse Event 18

6.3.1 Relationship to Study Intervention 18

6.3.2 Expectedness of SAEs 19

6.3.3 Severity of Event 19

6.4 Reporting Procedures 19

6.4.1 Reporting for Multi-Center Trials 19

6.4.2 Unanticipated Problem Reporting 20

6.4.3 Adverse Event Reporting of Non-IND Studies 20

6.4.4 Adverse Event Reporting for IND Studies 21

6.4.5 Events of Special Interest (if applicable) 21

6.4.6 Reporting of Pregnancy 21

6.5 Halting Rules 21

7 QUALITY CONTROL AND QUALITY ASSURANCE 21

7.1 Subject Accrual and Compliance 21
7.1.1 Measurement and Reporting of Subject Accrual 21
7.1.2 Measurement and Reporting of Participant Adherence to Treatment Protocol 22
7.2 Justification of Sample Size 22
7.3 Stopping Rules 22
7.4 Designation of a Monitoring Committee 23
7.5 Safety Review Plan 23
7.6 Study Report Outline for DSMB Annual Reports 24
7.7 Submission of On-Site Monitoring/Audit and Inspection Reports 24
7.8 Table A – Data Review Summary 25
8 DATA HANDLING AND RECORD KEEPING 26
8.1 Data Management Responsibilities 26
8.2 Database Protection 26
8.3 Source Document Protection 26
8.4 Schedule and Content of Reports 26
9 INFORMED CONSENT 27
10 REPORTING CHANGES IN STUDY STATUS 27
11 SUPPLEMENTS/APPENDICES 27

1 STUDY OVERVIEW

1.1 Purpose of Study

The primary objectives of the study are:

1. ***Impacts of MB-BP on Primary Self-Regulation Targets:*** Identify the impacts of MB-BP *vs*. enhanced usual care on the primary self-regulation target, specifically an assay of self-related processes (MAIA), described in Table 1. We hypothesize that MB-BP will significantly improve the MAIA in directions of better self-regulation, compared to control.
    a. Secondary analyses will evaluate impacts on secondary self-regulation targets including an assay of emotion regulation (DERS), and cognitive processes (SART), described in Table 1.
    b. Exploratory analyses will evaluate engagement of MB-BP *vs*. enhanced usual care with triangulated self-regulation target assays described in **Table 1** such as emotion regulation and stress (Pittsburgh Stress Battery, Perceived Stress Scale), self-related processes (Heart Beat Detection Task, Interoceptive Awareness fMRI Task), and cognitive processes (Mindful Attention Awareness Scale). Measures such as the neuroimaging Interoceptive Awareness fMRI Task will replicate assays in the MINDFUL-PC study.
2. ***Self-Regulation Targets as Mediators of MB-BP Effect on Medical Regimen Adherence and Health Behavior Change:*** Evaluate the degree to which the engagement of MB-BP with self-regulation targets translates into improved prehypertension/hypertension medical regimen adherence, specifically for the Dietary Approaches to Stop Hypertension (DASH)-consistent diet. We hypothesize that MB-BP will increase the DASH diet score compared to control, in participants with low DASH diet adherence at baseline (DASH diet score <5.5), and that the self-regulation primary outcome in Aim 1 (i.e. MAIA) is a significant mediator.
3. ***Further develop an MB-BP therapist manual and training program, including procedures for training, supervising, and evaluating therapists.*** The PI will implement training he receives from the University of Bangor in the United Kingdom in May 2018 to implement the Mindfulness-Based Interventions Teacher Assessment Criteria (MBI-TAC) for MB-BP instructors, which is the most respected quantitative and qualitative tool developed to provide feedback for enhancing MBI teacher effectiveness, and establishing teacher certification.[2-4]

1.2 Adherence Statement

The Data Safety Monitoring Plan (DSMP) outlined below for UH3AT009145 will adhere to the protocol approved by the Brown University IRB.

Text in red was added by Willoughby Britton to update the NCCIH DSMP template v.2.0 to meet CONSORT Harms Criteria.[1]

2 PROTOCOL AMENDMENTS

All protocol amendments, other than minor administrative changes as defined by the NCCIH Guidance on Changes in Clinical Studies in Active Awards will be submitted in a prospective manner to NCCIH except when necessary to protect the safety, rights, or welfare of subjects. Upon submitting the proposed changes to NCCIH the revisions will also be communicated to the Data and Safety Monitoring Board (DSMB) and to the Brown University IRB.

3 MULTI-SITE STUDIES

There is only one study site, Brown University, carrying out the Mindfulness-Based Blood Pressure Reduction (MB-BP) Study protocol. The University of Massachusetts Medical School will conduct the fMRI imaging study that involves recruiting, screening, and scanning a subset of MB-BP study participants. The MB-BP Study and the fMRI imaging study have separate study protocols, but share the same DSMP. Brown University will be considered the Coordinating Center. However, since the fMRI study involves its own study protocol, each local PI and study coordinator will be responsible for the following:

- •Design and develop the protocol and template informed consent documents for use at each collaborating institution
- •Ensure informed consent is obtained and documented from each subject in compliance with federal regulations
- •Store and/or manage data and data analysis activities
- •Provide study specific training to the research personnel at the affiliated sites
- •Develop and provide protocol specific case report forms for each affiliated site
- •Ensure that affiliated sites are using the correct version of the protocol and consent document.
- •Track subject enrollment
- •Ensure that collaborating sites are utilizing quality control measures to assure data accuracy and completeness.

Coordinating Center Responsibilities: The Coordinating Center (Brown University) will perform the following tasks:

- •Review and approve all documents used at affiliate sites
- •Ascertain each protocol is reviewed and approved by the IRB at the collaborating institution prior to enrollment of subjects at that site

- Ensure that each collaborating institution holds an applicable OHRP approved Federal Wide Assurance (FWA)

- Collect and maintain critical documents from affiliated investigators, e.g. resume/CV, medical license, certification of completion of training, laboratory certifications and laboratory norms, signed COI disclosure forms (for studies involving investigator sponsored INDs and IDEs)

- Store and/or manage data and safety monitoring activities

- Maintain documentation of all affiliated sites IRB approvals for the protocol

- Coordinate randomization as applicable

- Register subjects and track subject enrollment

- Track, report and maintain documentation of all serious adverse events and unanticipated problems and disseminating the information to affiliate sites

- Provide periodic updates to affiliated investigators on subject enrollment, general study progress, and relevant scientific advances

- Assure that all relevant IRB correspondence (continuing review and amendments) and study status changes are communicated to all affiliate sites

4 CONFIDENTIALITY

4.1 Protection of Subject Privacy

Subject confidentiality is strictly held in trust by the investigators, study staff, and the sponsor(s) and their agents. This confidentiality is extended to cover testing of biological samples and genetic tests in addition to any study information relating to subjects.

The study protocol, documentation, data, and all other information generated will be held in strict confidence. No information concerning the study or the data will be released to any unauthorized third party without prior written approval of the sponsor.

The study monitor or other authorized representatives of the sponsor may inspect all study documents and records required to be maintained by the investigator, including but not limited to, medical records (office, clinic, or hospital) for the study subjects. The clinical study site will permit access to such records.

This research is covered by a Certificate of Confidentiality from the National Institutes of Health.

4.2 Confidentiality During Adverse Event (AE) Reporting

AE reports and annual summaries will not include subject or group-identifiable material. Each report will only include the identification code.

5 EXPECTED RISKS

Meditation-related risks: NCCIH states that meditation is generally safe for healthy people, but that adverse effects have also been reported. [5] Undesirable side effects and risks of meditation have been documented in more than 40 scientific reports [for reviews see [6-8]] and are listed in the Mindfulness-Based Intervention Guidelines.[9,10] More common, less serious side effects that have been reported by individuals within the context of MBIs or of individuals who are meditating less than an hour per day include: increased depression, anxiety or panic, re-experiencing of traumatic memories, dissociation, executive dysfunction, headaches/body pain and insomnia.[6,11-16] A few case reports of more serious side effects including mania, psychosis, and suicidality have been reported, mostly in the contexts of intensive retreats (>5 hrs/day) or in conjunction with pre-existing psychopathology.[6,8,9,17] The frequency of serious adverse effects in the context of MBIs is estimated to be less than 1%, although adequate estimates are not available.[18]

A number of actions have been taken to minimize meditation-related risks at different stages of the study. During the pre-enrollment stage, individuals with severe mental illness are excluded from the study and all risks are clearly communicated in the consent form. During treatment, meditations are relatively short and interspersed with dyads and reflections. Mindfulness homework assigned as part of the intervention is optional and is recommended to not exceed 1 hour per day. Teachers query participants about their experiences with meditation, and provide corrective feedback or modifications when needed. Developing strategies for working with physical and emotional discomfort is an explicit goal of the program. Because not all participants feel comfortable disclosing difficulties in class, an online “safety check-in” questionnaire will query meditation-related risks (see section 6.2.1). Dr. Ellen Flynn, a licensed psychiatrist, will be available to advise on any psychological events that occur, and provide referrals for treatment if needed. Additionally, Dr. Willoughby Britton will provide expert consult on safety monitoring and reporting, including providing DSMP specific training to research staff and investigators.

Assessment-related risks: Research subjects participating in this study may have feelings of loss of privacy from being contacted about participating in the study, and possible psychological distress caused by questions asked during the in-person and online questionnaires that bring up painful memories or feelings. However, the resulting

potential for injury to research subjects is judged to be minimal. We have already contacted and clinically evaluated thousands of participants from other studies such as the New England Family and Women's Health Initiative using similar assessment procedures to this study, with good responses from the participants.

Loss of confidentiality: Likelihood: rare. Minimization: Confidentiality will be maintained by using deidentifying data sets. All paper forms and data collection tools, including the informed consent forms, will be kept in a locked filing cabinet in a secure location. All electronic data files containing identifying information will be encrypted with a cloud-based software. Note that although these measures have been taken to protect participants' personal information, complete confidentiality cannot be guaranteed when transmitting information over the internet. All information obtained from participants will be accessible only to research staff.

Injury due to physical activities: It is possible that injuries could be sustained from (1) the gentle mindful movements (yoga), or (2) physical activities that participants engage in as a result of the intervention encouraging exploration of physical activity as a way to reduce blood pressure. *(1) Mindful movements:* Participants receive a handout during the orientation showing the yoga poses that will be offered during the course. They are encouraged to explore limits in their body related to movement, but not to go beyond those limits Participants are asked to listen to what their body is telling them more closely than what the mindful movement instructor is telling them. Modifications of poses are available, including for those limited to chairs or wheelchairs. Participants are encouraged to bring the handout of poses to their health care providers if they have any physical limitations, so that the providers can advise on which poses to do, and which to avoid. *(2) Physical activities:* Participants are encouraged to explore physical activities that promote strength and conditioning as a way to reduce blood pressure. As with the mindful movements, they are encouraged to explore limits in their body related to movement, but not to go beyond those limits. Participants are asked to listen to what their body is telling them more closely than what the mindful movement instructor is telling them. Furthermore, they are encouraged to ask their healthcare provider about advised physical activities if they have any physical limitations.

Risks associated with fMRI: The fMRI study will be conducted using a 3T MR scanner at UMass Medical School, which has been approved for research and clinical studies in children and adults by the FDA. Magnetic resonance (MR) technology does not use X-rays, but instead uses strong magnetic fields and radio waves. Individuals interested in participating in the fMRI study will complete a screening questionnaire to assess eligibility, including asking whether they have devices that can be affected by MRI or conditions (e.g., claustrophobia, body mass greater than 300 lbs) that prohibit the ability to be scanned. Participants are screened immediately prior to each MRI scan to ensure

participant safety. Significant risks also can arise if ferromagnetic materials are brought into the high magnetic field environment of the scanner and immediate vicinity, as they can become hazardous projectiles. These types of items are not permitted in the scanning area. The MR exams are painless, and except for the pulsating sounds, subjects will not be aware that MR scanning is taking place. With proper safety precautions in terms of the avoidance of metal objects, there are no known health risks associated with MRI. The safety of MRI is reflected in the fact that it is used in standard medical practice without the requirement for informed patient consent. Most people experience no ill effects from the magnetic field, but some report claustrophobia, dizziness, mild nausea, headaches, a metallic taste in their mouth, double vision, or a sensation of flashing lights. These symptoms are transient and resolve quickly after the subject exits the scanner. The technologist will be able to hear subjects at all times and subjects are free to end the procedure at any time. In rare cases, a very slight, uncomfortable tingling of the back due to the rapid switching of the magnetic field has been reported during certain types of scans. Subjects are asked to report this immediately so the scan can be changed to avoid this. Although these precautions will avoid all known risks associated with MR, this procedure may involve risks that are currently unknown. The scanner is noisy, but does not harm hearing. For comfort, subjects will be given earplugs to muffle the noise.

Risk of adverse events during the study: It is possible that some patients will have an adverse event during the study, including increased stress or anxiety. Participants with major mental health conditions, such as schizophrenia, history of psychosis, bipolar depression, suicidal ideation, borderline personality disorder, post-traumatic stress disorder, obsessive compulsive disorder, panic attacks, current alcohol or substance abuse, or an eating disorder are ineligible for the study. We expect risk of adverse events to be very low. For further discussion of AE and SAE monitoring and reporting refer to Section 6.2 below.

Impact statement: These risks are considered to be minimal and are addressed in the protocol and consent form.

6 ADVERSE EVENT/ UNANTICIPATED PROBLEMS

6.1 Definitions

6.1.1 ***Adverse Event (AE)***

An adverse event (AE) is any untoward medical occurrence in a subject during participation in the clinical study or with use of the experimental agent being studied. An adverse finding can include a sign, symptom, abnormal assessment (laboratory test

value, vital signs, electrocardiogram finding, etc.), or any combination of these regardless of relationship to participation in the study.

6.1.2 *Unanticipated Problems (UP)*

The Office for Human Research Protections (OHRP) considers unanticipated problems involving risks to subjects or others to include, in general, any incident, experience, or outcome that meets **all** of the following criteria:

- Unexpected in terms of nature, severity, or frequency given (a) the research procedures that are described in the protocol-related documents, such as the IRB-approved research protocol and informed consent document; and (b) the characteristics of the subject population being studied;
- Related or possibly related to participation in the research ("possibly related" means there is a reasonable possibility that the incident, experience, or outcome may have been caused by the procedures involved in the research); and
- Suggests that the research places subjects or others at a greater risk of harm (including physical, psychological, economic, or social harm) than was previously known or recognized.

6.1.3 *Serious Adverse Event (SAE)*

A serious adverse event (SAE) is one that meets one or more of the following criteria:

- Results in death
- Is life-threatening (places the subject at immediate risk of death from the event as it occurred)
- Results in inpatient hospitalization or prolongation of existing hospitalization
- Results in a persistent or significant disability or incapacity
- Results in a congenital anomaly or birth defect

An important medical event that may not result in death, be life threatening, or require hospitalization may be considered an SAE when, based upon appropriate medical judgment, the event may jeopardize the subject and may require medical or surgical intervention to prevent one of the outcomes listed in this definition.

6.2 Time Period and Frequency for Event Assessment and Follow-Up

Safety monitoring will occur continuously throughout the study using both active and passive monitoring methods outlined in the table and discussed in further detail below.

TIMELINE OF SAFETY-RELATED ASSESSMENTS					
**Measure**	**Mode of measurement**	**During treatment**	**Post-treatment**	**In-person Follow-ups**	
6.2.1 Safety-check-ins					
Tier 1<br>Safety check-in	Online self-report survey	Every 2 weeks<br>(week 2,4,6,8)	Week 10<br>(in-person)	Switch to monthly up through 6 month and then final check-in conducted at 1 year	
Tier 2<br>Safety check-in	Online self-report survey + phone call	Administered immediately following a positive report of AE/SAE in Tier 1 survey. Tier 2 phone follow up will be made upon receipt of the Tier 2 data.			
6.2.2 Mental health					
CESD-R (includes suicidal ideation)	In person<br>Self-report	Baseline (pre-treatment)	Week 10	6 months	
BAI	In person<br>Self-report	Baseline (pre-treatment)	Week 10	6 months	
6.2.3 Physical Health					
Blood pressure	In person<br>physiological	Screener and Baseline	Week 10	6 months	1 year
Cardiac abnormalities	In person<br>physiological	Baseline	Week 10	6 months	
Injuries	To be captured in the Safety check-in's outline above				
6.2.4 Other					
Participant-initiated	Passive monitoring	continuous			
Attrition	Online self-report	continuous			
fMRI Study	Passive monitoring	fMRI research staff will document and report any AE/SAE events discovered during the baseline and/or 10 week imaging visits			

All reported AEs, SAEs, and unanticipated problems will be recorded throughout the study using the data collection systems set up and detailed in the Manual of Operating Procedures (MOP).

The research staff will record all reportable events with start dates occurring any time after informed consent is obtained but no later than the final 1 year assessment. At each study visit, the research staff will inquire about the occurrence of AE/SAEs since the last visit (or time of most recent reporting). Events will be followed for outcome information until resolution or stabilization or until the grant funding ends.

6.2.1 ***Safety Check-ins***

All participants enrolled in the study, regardless of treatment allocation, will receive a two-tiered safety monitoring ‘check-in’ every 2 weeks during the treatment phase of the study; every month during months 3-6 of the follow-up phase; and at the final 1 year time point. The two-tiered system is designed to detect and follow up on AEs that are at least moderate in severity (interfere with ADL), and to minimize staff and participant burden that would otherwise occur if all mild events were queried and documented. Refer to Appendix A1 for the complete safety check-in surveys.

Tier 1 safety check-in survey:

Tier 1 of the safety check-in involves sending all active, enrolled participants an email (or placing a phone call from a research staff member, if no email provided) containing a link to a brief online survey that queries events with moderate or greater levels of severity. Specifically, study participants will be asked:

"During the past [two weeks / month / 6 months] have you experienced any of the following:

- change in prescribed or over the counter medication use (y/n),
- sustained any physical injuries (y/n),
- underwent any medical procedures (y/n),
- received any medical/clinical care outside of routine or preventative health care (y/n),
- been hospitalized (y/n)?"
- experienced physical or mental health symptoms that interfered with your daily activities (y/n)

Any participant who endorses one or more of the above tier 1 questions will automatically receive a tier 2 survey and follow-up phone call from study staff.

Tier-2 safety check-in Survey:

CONSORT-Harms guidelines[1] recommend using standardized and validated scales that assess treatment-specific symptoms, because they have more accurate detection rates than passive monitoring[14] or open-ended queries[15,16] and are low cost, low burden and require no special training to administer. In addition, patient or consumer-based self-reports (rather than face-to-face interviews or clinician ratings) are recommended for reporting sensitive or socially undesirable information such as negative reactions to treatment.[17-19] To accommodate these recommendations, and to create systematic query of treatment-specific negative events in all treatment arms, the Tier 2 survey specifically queries the most common meditation-related side effects (anxiety, depression, dissociation, flashbacks etc.) using patient-reported outcomes measurement information system (PROMIS) or NeuroQol items (or other validated scales of construct is not available), and PROMIS response options (never- very often).

Additionally, participants will be asked to provide further detail (i.e., date of onset, symptomology, circumstance surrounding the event, relatedness to the intervention, etc.) on the AE/SAE reported in the Tier 1 survey. Detail provided will be used to guide the Tier 2 phone calls made by research study staff.

Both the Tier-1 and Tier-2 Safety Check-in Surveys are located in Appendix A1.

Tier 2 Safety Check-in phone call: Any participant who indicates that he or she experienced an AE or SAE will then receive a follow up call from a trained research staff member. The purpose of the follow up call will be to further document the details of the AE/SAE, assess need for treatment modification, referrals and reporting.

Adverse Events form

Specifically, the staff member will use the Adverse Events Form and accompanying documents (i.e., the Severity Grading Tool, Relatedness Assessment Tool, and List of Previously-Reported Meditation Effects (all found in the Appendices)) in order to ascertain:

a) A description of the AE/SAE
b) Start and end dates
c) Severity grading (using Severity grading Tool)
d) Relationship to treatment (using Relatedness Assessment Tool + List of Previously-Reported Meditation Effects in the Appendix)
e) Action taken (if any)
f) Outcome (as assessed after 2 weeks or at next check-in time point)
g) Expectedness (Refer to List of Previously-Reported Meditation Effects in the Appendix)

h) Seriousness (See Section 6.1.3 of this document and/or Severity Grading Tool for definition of SAE; use Serious Adverse Events Form in Appendix if event is Serious)

Research staff members conducting the safety monitoring phone interviews will document the details of the AE/SAE, which will then be included in the participant file as well as in the annual Data Safety Monitoring Reports presented to the DSMB. Reporting procedures for AEs and SAEs related to the study will be followed including reporting all SAEs to the study PI and DSMB committee chair.

6.2.2 ***Mental health***

Participants assigned to any treatment group may experience mental health or suicidal ideation during the course of their study involvement. All study participants will be monitored for AEs and SAEs by study staff on a monthly basis until the time of their study completion. Additionally, participants will be asked to complete questionnaires about anxiety, depression and suicidal ideation, specifically the Beck Anxiety Inventory and the Center for Epidemiology Study Depression Scale Revised (CESD-R), at each of their in-person assessments, excluding the one year follow up. Dr. Flynn, a licensed psychiatrist with extensive experience evaluating research participants for clinical deterioration or suicidality, will serve as the study clinician.

Beck Anxiety Inventory (BA): If participant scores ≥26 on the Beck Anxiety Inventory, a safety flag will appear notifying the research assistant (RA) administering the assessment. The RA will then implement the MB-BP safety protocol, which is reviewed and approved by the Brown University IRB. Staff are trained on the safety protocol and a hard copy of the protocol is kept in an accessible location in the assessment office at all times.

Depressive Symptomatology: The CESD-R will be administered during the in-person assessment visits, and scores will be reviewed immediately upon completion of the in-person assessments.

1. Sadness (dysphoria): Question numbers 2,4, 6
2. Loss of Interest (anhedonia): Question numbers 8, 10
3. Appetite: Question numbers 1, 18
4. Sleep: Question numbers 5, 11, 19
5. Thinking / concentration: Question numbers 3, 20
6. Guilt (worthlessness): Question numbers 9, 17
7. Tired (fatigue): Question numbers 7, 16
8. Movement (agitation): Question numbers 12, 13
9. Suicidal ideation: Question numbers 14, 15

Participants are considered to meet criteria for major depressive episode if they have anhedonia or dysphoria nearly every day for the past two weeks, plus symptoms in an additional 4 DSM symptom groups noted as occurring nearly every day for the past two weeks. If participants meet criteria for major depressive episode, a safety flag will appear notifying the research assistant (RA) administering the assessment. The RA will then implement the MB-BP safety protocol, which is reviewed and approved by the Brown University IRB. Staff are trained on the safety protocol and a hard copy of the protocol is kept in an accessible location in the assessment office at all times.

If participants respond having any suicidal ideation (CES-D questions 14 or 15), staff will again be instructed to follow the IRB approved safety protocol.

6.2.3 ***Physical Health***

Possible atrial fibrillation detected during Heartbeat Detection Task: If possible atrial fibrillation is indicated by the Kardia Mobile device during the "Heart Beat Detection Task" a safety flag will appear notifying the research assistant (RA) administering the assessment. The RA will then implement the MB-BP safety protocol.

Out-of-range blood pressure readings: If during an in-person assessment the participants systolic blood pressure (SBP) and/or diastolic blood pressure (DBP) falls outside of the acceptable range outlined in the safety protocol, the RA will be notified and the IRB approved safety protocol will be implemented.

Injury due to physical activities: It is possible that injuries could be sustained from (1) the gentle mindful movements (yoga), or (2) physical activities that participants engage in as a result of the intervention encouraging exploration of physical activity as a way to reduce blood pressure.

(1) Mindful movements: Participants receive a handout during the orientation showing the yoga poses that will be offered during the course. They are encouraged to explore limits in their body related to movement, but not to go beyond those limits Participants are asked to listen to what their body is telling them more closely than what the mindful movement instructor is telling them. Modifications of poses are available, including for those limited to chairs or wheelchairs. Participants are encouraged to bring the handout of poses to their health care providers if they have any physical limitations, so that the providers can advise on which poses to do, and which to avoid.

(2) Physical activities: Participants are encouraged to explore physical activities that promote strength and conditioning as a way to reduce blood pressure. As with the mindful movements, they are encouraged to explore limits in their body

related to movement, but not to go beyond those limits. Participants are asked to listen to what their body is telling them more closely than what the mindful movement instructor is telling them. Furthermore, they are encouraged to ask their healthcare provider about advised physical activities if they have any physical limitations.

Note that adverse events related to physical injuries will be captured during the routine safety check-ins.

6.2.4 ***Other***

Participant Initiated (Passive monitoring): Participants are encouraged to contact meditation instructors and/or study staff if any physical or mental health symptoms arise or other study or meditation-related problems occur. Participants may report AEs at any time throughout the study. Events will be evaluated with the Adverse Events Form by study staff.

Attrition: Reasons for attrition are also an important source of AEs, but are rarely assessed adequately, as participants are unlikely to give honest answers if queried directly by study staff. To increase the accuracy of attrition reason reporting, participants will asked to complete a brief online Participant-initiated dropout reason survey (see Appendix A7).

Investigator-initiated withdrawals: A participant may also be withdrawn from the study and/or intervention by the researcher. In this case, the researcher or other study staff should complete the Attrition information form, and describe reasons for attrition.

fMRI Study Safety Monitoring: it is possible participants may experience or report an AE or SAE during their involvement with the fMRI Study. Research staff will use the Adverse Events Form and accompanying documents found in the Appendices to document all AE/SAE discovered at the time of involvement in the fMRI imaging study. The logged events will then be communicated to the Coordinating Center, so that they can be included in the participant file as well as in the annual Data and Safety Monitoring Reports presented to the DSMB. Reporting procedures for AEs and SAEs related to the study will be followed including reporting all SAEs to the study PI and DSMB committee chair.

6.3 Characteristics of an Adverse Event

6.3.1 *Relationship to Study Intervention*

According NIH guidelines, an event is considered related to the treatment (Possible, Probable, Definite) if: a) The event is known to occur with the study intervention; b) There is a temporal relationship between the intervention and event onset; c) The event abates when the intervention is discontinued; or d) The event reappears upon a re-challenge with the intervention. An event is considered Not Related (Unlikely, Not Related) if: a) There is no temporal relationship between the intervention and event onset or b) An alternate etiology has been established.

Relatedness to treatment will be assessed according to 8 causality and relatedness criteria that regulatory agencies such as the World Health Organization (WHO), the Federal Drug Administration (FDA), and the National Institutes of Health (NIH) use to make health policy decisions.[20-23] These criteria are designed to assess treatment-relatedness in individual cases in the absence of prospective or epidemiological (base rate) data. The 8 standard causality/relatedness criteria are 1) prior published reports, and expert judgment, 2) subjective attribution, 3) temporal proximity (challenge), or exacerbation, 4) consistency, 5) dose-response gradient 6) de-challenge, 7) re-challenge, and 8) specificity.[21,24-30]

The Relatedness Criteria Definitions table below provides detailed definitions, and the Relatedness Assessment Tool (Appendix A4) provides a systematic method for assessing relatedness in individual cases.

Relatedness Criteria Definitions	
1. Previous Reports/known effects and expert judgment	Symptom has been previously reported in association with meditation or judged to be caused by meditation by experts (meditation teachers/clinicians)
2. Subjective attribution	Participant thinks symptom was caused by meditation
3. Temporal proximity OR Exacerbation	Symptom occurred for the first time OR pre-existing symptoms got worse during/following treatment
4. Consistency	More than one occasion following meditation
5. Dose/gradient	Symptom was worse with higher dose of treatment
6. De-challenge	Decreased when stopped meditation
7. Re-challenge	Re-appeared or increased when meditation restarted
8. Specificity	Presence of plausible alternative cause: Something other than meditation (medical illness, life event, substances) could account for symptom (meditation played no causal role).

6.3.2 *Expectedness of SAEs*

The Study PI and Data Safety Monitoring Board (DSMB) will be responsible for determining whether an SAE is expected or unexpected. An adverse event will be considered unexpected if the nature, severity, or frequency of the event is not consistent with the risk information previously described for the intervention. Specifically, an event is considered expected if it appears on the List of Previously-Reported Meditation Effects in the Appendix A5.

6.3.3 *Severity of Event*

Severity is graded from 1 to 5 with corresponding description as mild, moderate, severe, very severe and death according to the Common Terminology Criteria for Adverse Events (CTCAE) and other sources for grading the severity of adverse events. Seriousness, which requires a different reporting procedure is defined according to the Office for Human Research Protections, *Guidance on Reviewing and Reporting Unanticipated Problems Involving Risks to Subjects or Others and Adverse Events.*[31-33] See Appendix A3 for the Severity Grading Tool.

6.4 Reporting Procedures

6.4.1 *Reporting for Multi-Center Trials*

There is only one study site, Brown University, carrying out the Mindfulness-Based Blood Pressure Reduction (MB-BP) Study protocol. The University of Massachusetts Medical School will conduct the fMRI imaging study that involves recruiting, screening, and scanning a subset of MB-BP study participants. The MB-BP Study and the fMRI imaging study have separate study protocols, but share the same DSMP.

The site research staff and local PI must immediately report to the coordinating center PI any serious adverse event, whether or not considered study related, including those listed in the protocol or investigator brochure and must include an assessment of whether there is a reasonable possibility that the study caused the event within 48 hours of PI awareness of the event.

They must also report any unanticipated problems within the same timeframe. The Site PI or research staff must also report any protocol deviations or violations to the coordinating center PI within 7 days of PI awareness. Participating centers must also submit all reports to their local IRB in accordance with their institutional policies.

All other AEs documented by the fMRI Study Research Staff will be reported to the coordinating center on an annual basis in the process of compiling the data for the annual report and in the annual AE summary which will be provided to NCCIH and to the DSMB.

6.4.2 *Unanticipated Problem Reporting*

Incidents or events that meet the OHRP criteria for unanticipated problems require the creation and completion of an unanticipated problem report form. OHRP recommends that investigators include the following information when reporting an adverse event, or any other incident, experience, or outcome as an unanticipated problem to the IRB:

- Appropriate identifying information for the research protocol, such as the title, investigator's name, and the IRB project number;
- A detailed description of the adverse event, incident, experience, or outcome;
- An explanation of the basis for determining that the adverse event, incident, experience, or outcome represents an unanticipated problem;
- A description of any changes to the protocol or other corrective actions that have been taken or are proposed in response to the unanticipated problem.

To satisfy the requirement for prompt reporting, unanticipated problems will be reported using the following timeline:

- Unanticipated problems that are serious adverse events will be reported to the IRB, DSMB, and NCCIH within 7 days of the investigator becoming aware of the event.
- Any other unanticipated problem will be reported to the IRB, DSMB, and NCCIH within 14 days of the investigator becoming aware of the problem.

All unanticipated problems should be reported to appropriate institutional officials (as required by an institution's written reporting procedures), the supporting agency head (or designee), and OHRP within one month of the IRB's receipt of the report of the problem from the investigator.

6.4.3 *Adverse Event Reporting of Non-IND Studies*

SAEs that are unanticipated, serious, and possibly related to the study intervention will be reported to the DSMB, IRB, and NCCIH in accordance with requirements.

- Unexpected fatal or life-threatening AEs related to the intervention will be reported to the NCCIH Program Officer, and DSMB within 3 days of the investigator becoming aware of the event. Other serious and unexpected AEs related to the intervention will be reported within 7 days.
- Anticipated or unrelated SAEs will be handled in a less urgent manner but will be reported to the DSMB, IRB, and other oversight organizations in accordance with their requirements. and will be reported to NCCIH on an annual basis.
- All other AEs documented during the course of the trial will be reported to NCCIH on an annual basis by way of inclusion in the annual report and in the annual AE

summary which will be provided to NCCIH and to the Independent Monitors. The DSMB Report will state that all AEs have been reviewed.

6.4.4 *Adverse Event Reporting for IND Studies*

Not Applicable.

6.4.5 *Events of Special Interest (if applicable)*

Not Applicable.

6.4.6 *Reporting of Pregnancy*

Not Applicable.

6.5 Halting Rules

This study will be stopped prior to its completion if: (1) the intervention is associated with adverse effects that call into question the safety of the intervention; (2) difficulty in study recruitment or retention will significantly impact the ability to evaluate the primary outcomes; (3) any new information becomes available during the trial that necessitates stopping the trial. The study may also be discontinued at any time by the IRB, the NCCIH, or other government agencies as part of their duties to ensure that research subjects are protected.

7 QUALITY CONTROL AND QUALITY ASSURANCE

There are a number of quality control measures established in the MB-BP study in order to help ensure data integrity and study credibility. For example, research staff administering in-person assessments are required to complete quality control assessment checklists to ensure that all aspects of the assessment are completed in a standardized and thorough manner. The checklist is reviewed by the Senior Project Coordinator as a method to identify potential issues with data quality and as a method for ongoing RA training. Other quality control checklists are utilized at each stage of the data collection periods to ensure that all participants in a given cohort are followed up with and to double check for the presence and completeness of data that was known to be collected.

All equipment utilized in the lab are calibrated at least quarterly. Logs of each calibration are kept. Research staff complete routine retraining on clinical skills, such as taking blood pressure and anthropometric readings.

7.1 Subject Accrual and Compliance

7.1.1 *Measurement and Reporting of Subject Accrual*

Review of the rate of subject accrual and compliance with inclusion/exclusion criteria will occur monthly during the recruitment phase to ensure that a sufficient number of participants are being enrolled, in keeping with proposed recruitment projections, and

that they meet eligibility criteria and fulfill the targeted ethnic diversity goals outlined in the grant proposal (Targeted/Planned Enrollment Table).

7.1.2 ***Measurement and Reporting of Participant Adherence to Treatment Protocol***

Adherence to the prescribed MB-BP practices will be monitored through class attendance, practice logs and weekly health goals. Adherence data will be collected weekly during the course of the intervention. We discussed best practices to measure and monitor adherence to treatment protocol with leading meditation researchers, and meditation logs remain their recommended method at this time. Innovative approaches are being explored by Dr. Lazar and other investigators, including smartphone apps linked to audio homework files, with timers linked to data exports. However, these technologies are not yet ready for use. Data analyses will evaluate effect modification by adherence to the MB-BP practices.

7.2 Justification of Sample Size

The goal of the study is to evaluate the effect of the Mindfulness-Based Blood Pressure Reduction (MB-BP) intervention on the primary and secondary outcomes as compared to the enhanced usual care group. To do this we estimate needing to enroll 50 individuals per group for both the MB-BP intervention arm and the enhanced usual care arm. For details on the power calculations used refer to the study protocol. For the fMRI imaging study we estimate needing a final sample size of 48 (24 participants per group).

Our calculations assume drop-out rates will average around 10-15% across each group, with the control group exhibiting the highest rates of withdrawal and loss to follow up. These estimates are based on the Stage 1 and 2a MB-BP trials in the UH2 phase.

7.3 Stopping Rules

This study will be stopped prior to its completion if: (1) the intervention is associated with adverse effects that call into question the safety of the intervention; (2) difficulty in study recruitment or retention will significantly impact the ability to evaluate the study endpoints; (3) any new information becomes available during the trial that necessitates stopping the trial; or (4) other situations occur that might warrant stopping the trial.

The study may also be discontinued at any time by the IRB, the NCCIH, or other government agencies as part of their duties to ensure that research subjects are protected.

7.4 Designation of a Monitoring Committee

The Data and Safety Monitoring Board (DSMB) for this study is comprised of Drs. Donald Edmondson, Gaurav Choudhary, and Tao Liu. These individuals are not associated with this research project and work independently of the PI, Dr. Eric Loucks. Investigator. They are not part of the key personnel involved in this grant. No member of the Committee has collaborated or co-published with the PI within the past three years. They are qualified to review the patient safety data generated by this study because of their unique expertise in the areas of cardiology, psychology/psychiatry, epidemiology, and biostatistics.

The Data Safety Monitoring Committee will provide external monitoring, and will meet annually by phone, video conference, or in-person. They will be provided data annually in order to evaluate potential effects of the RCT on major outcomes (e.g. medical regimen adherence). Any serious adverse effects will be immediately reported to the principal investigator (Loucks) and the committee chair.

Oversight of the external Data and Safety Monitoring Committee will be conducted by the chair, Dr. Donald Edmondson, PhD, who is Associate Professor of Behavioral Medicine at Columbia University Medical Center. He is a Psychologist, and has extensive research experience in evaluating effects of stress and psychosocial factors on cardiovascular disease outcomes.

The Data and Safety Monitoring committee will also include a board-certified cardiologist, Dr. Gaurav Choudhary, and a biostatistian, Dr. Tao Liu. Dr. Choudhary, MD, is Associate Professor of Medicine and Associate Chief of Staff at the Providence VA Medical Center. He is a practicing clinical cardiologist with research in cardiology, and will be able to advise on clinical outcomes and any cardiovascular complications arising from the study, in addition to methodological concerns. Dr. Liu, PhD, is an Assistant Professor of Biostatistics at Brown University, experienced in clinical trials. He will receive preliminary analyses from the primary statistician, and will be given access to all data from the study, to evaluate any evidence of serious adverse effects or other concerns.

7.5 Safety Review Plan

Study progress and safety will be reviewed monthly (and more frequently if needed). Progress reports, including patient recruitment, retention/attrition, and AEs will be provided to the DSMB prior to each annual review. Annual Reports will be compiled and will include a list and summary of AEs/SAEs. Additionally, Annual Reports will address (1) whether AE rates are consistent with pre-study assumptions; (2) reason for dropouts from the study; (3) whether all participants met entry criteria; (4) whether continuation of the study is justified on the basis that additional data are needed to accomplish the stated aims of the study; and (5) conditions whereby the study might be terminated prematurely. Annual Reports will be sent to the DSMB and will be forwarded to NCCIH. The IRB and other applicable recipients will review progress of this study on an annual

basis.

The PI will also send copies of signed recommendations and comments from the DSMB or DSMB Chair to the NCCIH Program Officer within 1 month of each monitoring review.

7.6 Study Report Outline for DSMB Annual Reports

The study team will generate annual Study Reports for the DSMB and will provide information on the following study parameters:

I. Recruitment Summary Information
   A. Recruitment Status
      i. Enrollment by Year/Month
      ii. Comparison of Targeted to Actual Enrollment

   B. Retention Status
      i. Overall Subject Status
      ii. Individual Subject Status

II. Study Data Reports/Tables or Figures
   A. General Information
      i. Enrollment
      ii. Demographic/Baseline Data
      iii. Subject Status

   B. Safety Assessment
      i. Treatment Duration for All Subjects
      ii. AE Data
         a. Overall Listing
         b. Specific Symptom Listing
         c. Out-of-Range Laboratory Values
         d. Out-of-Range Depression and Anxiety Scores
         e. SAE Listing
         f. Subject Deaths

Study Report tables will be generated only from aggregate (not by group assignment) baseline and aggregate safety data for the study population.

7.7 Submission of On-Site Monitoring/Audit and Inspection Reports

The IRB, DSMB, and NCCIH Program Officials will receive copies of all study monitoring/audit or inspection reports as required by individual institutation. For example, the NCCIH (Westat) monitoring report will be submitted to the IRB and DSMB (NCCIH does not require copies of Westat monitoring reports).

7.8 Table A – Data Review Summary

Data type	Frequency of review	Reviewer
Subject accrual (including compliance with protocol enrollment criteria)	Monthly	PI, Internal QA Reviewer
	Annually	DSMB
Status of all enrolled subjects, as of date of reporting	Monthly	PI, Internal QA Reviewer
	Annually	DSMB
Data entry quality control checks on 10% of charts	As needed based on frequency of data entry	QA Reviewer
Adherence data regarding study visits and intervention<br>AEs and rates (including out-of-range lab values)	Annually	DSMB
	Ongoing and in preparation for DSMB Report Creation	PI, Internal QA Reviewer
AEs and rates (including out-of-range lab values)<br>SAEs (unexpected and related)	Annually	DSMB
	Annually	NCCIH, FDA (If Applicable)
	Per occurrence	PI, DSMB Chair<br>NIH/NCCIH, FDA (if applicable)
SAEs (expected or unrelated)	Per Occurrence	PI, Internal QA Reviewer
SAEs (expected or unrelated)<br>Unanticipated Problems	Annually	DSMB, NIH/NCCIH
	Monthly	PI, Internal QA Reviewer
Unanticipated Problems	Per Policy	IRB, FDA (if applicable)

8 DATA HANDLING AND RECORD KEEPING

The investigators are responsible for ensuring the accuracy, completeness, legibility, and timeliness of the data reported. All source documents should be completed in a neat, legible manner to ensure accurate interpretation of data. The investigators will maintain adequate case histories of study subjects, including accurate case report forms (CRFs), and source documentation.

8.1 Data Management Responsibilities

Data collection and accurate documentation are the responsibility of the study staff under the supervision of the investigator. All source documents and laboratory reports must be reviewed by the study team and data entry staff, who will ensure that they are accurate and complete. Unanticipated problems and adverse events must be reviewed by the investigator or designee.

8.2 Database Protection

The clinical data will be de-identified but linked. Private information such as name, date of birth, and address for recontacting will be kept in a password protected, encrypted database on a different disk than the clinical data held by the Project Coordinator and used for the purposes of contacting participants. The principal investigator will only be given access to identifiable personal information for the purposes of patient safety or monitoring by the NIH, data safety monitoring boards or HIPPA compliance officer approved agents. Electronic communication with outside collaborators will involve only unidentifiable information.

Data management will be performed by downloading data at minimum every 2 weeks during active data collection periods, and assessing data for missingness and errors. Data will be maintained in password-protected Microsoft Excel Spreadsheets, and then exported using .csv functions for analysis in SAS software.

8.3 Source Document Protection

All source documents, including all paper and electronic records for all enrolled participants (i.e., case report forms, laboratory reports, etc.) will be maintained in a secure location. Original paper documents, even if data entered, will be maintained for the duration of the study. Data authenticator information will be recorded.

8.4 Schedule and Content of Reports

For detail on the schedule and content of study reports refer to the study protocol and manual of operating procedures.

9 INFORMED CONSENT

Informed consent is a process that is initiated prior to the individual agreeing to participate in the study and continues throughout study participation. Extensive discussion of risks and possible benefits of study participation will be provided to subjects and their families, if applicable. A consent form describing in detail the study procedures and risks will be given to the subject. Consent forms will be IRB-approved, and the subject is required to read and review the document or have the document read to him or her. The investigator or designee will explain the research study to the subject and answer any questions that may arise. The subject will sign the informed consent document prior to any study-related assessments or procedures. Subjects will be given the opportunity to discuss the study with their surrogates or think about it prior to agreeing to participate. They may withdraw consent at any time throughout the course of the study. A copy of the signed informed consent document will be offered to subjects for their records. The rights and welfare of the subjects will be protected by emphasizing to them that the quality of their clinical care will not be adversely affected if they decline to participate in this study.

The consent process will be documented in the clinical or research record.

To complete the informed consent process at the end of study participation, study staff will inform the subject when his/her participation has come to an end.

10 REPORTING CHANGES IN STUDY STATUS

During the funding of this study, any action by the IRB, the DSMB, or one of the study investigators that results in a temporary or permanent suspension of the study will be reported to the NCCIH Program Official within 5 business days of notification.

11 SUPPLEMENTS/APPENDICES

Note that minor modifications may be made to the individual documents listed in the appendices during the course of the study.

- A1_Tier 1+ 2 Safety Check Surveys
- A2_Adverse Event Form
- A3_Severity Grading Tool
- A4_Relatedness Assessment Tool
- A5_List of Previously-Reported Meditation Effects
- A6_Serious Adverse Events Form
- A7_Attrition Information Form

REFERENCES:

1. Ioannidis JP, Evans SJ, Gotzsche PC, et al. Better reporting of harms in randomized trials: an extension of the CONSORT statement. *Annals of internal medicine.* 2004;141(10):781-788.
2. Crane RS, Eames C, Kuyken W, et al. Development and validation of the mindfulness-based interventions - teaching assessment criteria (MBI:TAC). *Assessment.* 2013;20(6):681-688.
3. Crane RS, Kuyken W, Williams JM, Hastings RP, Cooper L, Fennell MJ. Competence in Teaching Mindfulness-Based Courses: Concepts, Development and Assessment. *Mindfulness (N Y).* 2012;3(1):76-84.
4. Crane RS, Brewer J, Feldman C, et al. What defines mindfulness-based programs? The warp and the weft. *Psychol Med.* 2017;47(6):990-999.
5. NCCIH. What the Science Says About Safety and Side Effects of Meditation. *National Cennter for Complementary and Intrgrative Medicine (NCCIH), National Insitutes of health (NIH)*. *https://nccih.nih.gov/health/meditation/overview.htm#hed52018*.
6. Lindahl JR, Fisher NE, Cooper DJ, Rosen RK, Britton WB. The varieties of contemplative experience: A mixed-methods study of meditation-related challenges in Western Buddhists. *PloS one.* 2017;12(5):e0176239.
7. Lustyk M, Chawla N, Nolan R, Marlatt G. Mindfulness Medtation Research: Issues of participant screening, safety procedures, and researcher training. *Advances in Mind-Body Medicine.* 2009;24(1):20-30.
8. Kuijpers H, van der Heijden F, Tuinier S, Verhoeven W. Meditation-induced psychosis. *Psychopathology.* 2007;40:461-464.
9. Kuyken W, Crane W, Williams JM. Mindfulness-Based Cognitive Therapy (MBCT) Implementation Resources. *Oxford University, University of Exeter, Bangor University.* 2012.
10. Santorelli S, Meleo-Meyer F, Koerbel L, et al. Mindfulness-Based Stress Reduction (MBSR) Authorized Curriculum Guide. *Center for Mindfulness in Medicine, Health Care, and Society (CFM), University of Massachusetts Medical School.* 2017.
11. Lomas T, Cartwright T, Edginton T, Ridge D. A qualitative summary of experiential challenges associated with meditation practice. *Mindfulness.* 2014:1-13.
12. Cebolla A, García-Campayo J, Demarzo M, Soler J. Side Effects of Meditation: A Survey Among Spanish Meditators International Sympsosium for Contemplative Studies (ISCSS); Oct 30-Nov 2, 2014, 2014; Boston, MA.
13. Yorston G. Mania precipitated by meditation: a case report and literature review. *Mental Health, Religion & Culture.* 2001;4:209-214.
14. Stephens M, Talbot J, Routledge P, eds. *The Detection of New Adverse Reactions.* 4th ed. London: Macmillan Reference; 1998.
15. Wallin J, Sjovall J. Detection of adverse drug reactions in a clinical trial using two types of questioning. *Clin Ther.* 1981;3(6):450-452.

16. Bent S, Padula A, Avins AL. Brief communication: Better ways to question patients about adverse medical events: a randomized, controlled trial. *Annals of internal medicine.* 2006;144(4):257-261.
17. Fowler F. Mode effcts in a survey of Medicare prostate surgery patients. *Public Opinion Quarterly.* 1998;62:29-46.
18. Turner C, Lessler J, George B, Hubbard M, Watt M. Effects of mode of administration and wording on reporting of drug use. In: Turner CF, Lessler JT, Gfroerer JC, eds. *Survey Measurement of Drug Use: Methodological Studies*. Washington, D.C: Government Printing Office. ; 1992:177-220.
19. Weissman JS, Schneider EC, Weingart SN, et al. Comparing patient-reported hospital adverse events with medical record review: do patients know something that hospitals do not? *Annals of internal medicine.* 2008;149(2):100-108.
20. NIH. Adverse Event and Serious Adverse Event Guidelines. *OHRP Guidance on Reviewing and Reporting Unanticipated Problems Involving Risks to Subjects or Others and Adverse Events, OHRP Guidance*. National Insitutes of Health (NIH): Office for Human Research Protections, U.S. Department of health and Human Servcies; 2016.
21. Agbabiaka TB, Savovic J, Ernst E. Methods for causality assessment of adverse drug reactions: a systematic review. *Drug safety.* 2008;31(1):21-37.
22. Turner WM. The Food and Drug Administration algorithm. Special workshop--regulatory. *Drug information journal.* 1984;18(3-4):259-266.
23. WHO. *The use of the WHO-UMC system for standardized case causaility assessment.* who-umc.org: World Health Organization (WHO), Uppsala Monitoring Centre 2016.
24. Naranjo CA. A clinical pharmacologic perspective on the detection and assessment of adverse drug reactions. *Drug information journal.* 1986;20(4):387-393.
25. Naranjo CA, Busto U, Sellers EM. Difficulties in assessing adverse drug reactions in clinical trials. *Progress in neuro-psychopharmacology & biological psychiatry.* 1982;6(4-6):651-657.
26. Naranjo CA, Busto U, Sellers EM, et al. A method for estimating the probability of adverse drug reactions. *Clinical pharmacology and therapeutics.* 1981;30(2):239-245.
27. Hill AB. The Environment and Disease: Association or Causation? *Proceedings of the Royal Society of Medicine.* 1965;58:295-300.
28. Hill AB. The environment and disease: association or causation? *Journal of the Royal Society of Medicine.* 2015;108(1):32-37.
29. Gallagher RM, Kirkham JJ, Mason JR, et al. Development and inter-rater reliability of the Liverpool adverse drug reaction causality assessment tool. *PloS one.* 2011;6(12):e28096.
30. Theophile H, Arimone Y, Miremont-Salame G, et al. Comparison of three methods (consensual expert judgement, algorithmic and probabilistic approaches) of causality assessment of adverse drug reactions: an assessment using reports made to a French pharmacovigilance centre. *Drug safety.* 2010;33(11):1045-1054.

31. Linden M. How to define, find and classify side effects in psychotherapy: from unwanted events to adverse treatment reactions. *Clinical psychology & psychotherapy.* 2013;20(4):286-296.
32. OHRP. *Guidance on Reviewing and Reporting Unanticipated Problems Involving Risks to Subjects or Others and Adverse Events.* Office for Human Research Protections, US Department of Health and Human Services; 2007.
33. NCI. Common Terminology Criteria for Adverse Events (CTCAE) v5.0. *National Cancer Insititute, NIH, US Department of Health and Human Services.* 2017.

Appendix A1

Tier 1 and Tier 2 Safety Check Surveys

TIER 1 SAFETY-CHECK SURVEY		
**In the last { 2 weeks / month / 6 months }, have you have experienced the following:**		
	**NO**	**YES**
1. Change in prescribed or over the counter medication use		
2. Sustained any physical injuries		
3. Underwent any medical procedures		
4. Received any medical/clinical care outside of routine or preventative health care		
5. Been hospitalized		
6. Experienced physical or mental health symptoms that interfered with your daily activities		

IF YES TO ANY OF THE ABOVE, INITIATE:

- TIER 2 SURVEY
- FOLLOW-UP PHONE CALL

TIER 2 SAFETY CHECK-IN, Part 1

1. *If YES to Tier 1, Q1 (change in medication)* – You indicated that you recently made a change to your prescribed and/or over the counter medication use….

    1a. Please describe the changes that were made including providing information on the medications added, removed, or altered (i.e., names of medications, dosage, frequency, etc.).

    1b. When did the change in medication take place?

    1c. Please describe the circumstances / reasons for the change.

2. *If YES to Tier 1, Q2 (physical injury)* – You indicated that you recently sustained a physical injury….

    2a. Please describe the injury.

    2b. When were you injured?

    2c. How were you injured?

    2d. Did the injury include:
    Broken bone(s) – Y/N
    Lacerations requiring stiches – Y/N
    Concussion – Y/N

3. *If YES to Tier 1, Q3 (medical procedure)* – You indicated that you recently underwent a medical procedure…

    3a. Please describe the medical procedure.

    3b. When did it take place?

    3c. What was the procedure for?

4. *If YES to Tier 1, Q4 (non-preventive medical care)* – You indicated that you recently received medical / clinical care outside of routine or preventative health care…

    4a. Please describe the care that you received.

    4b. When did you receive the care?

    4c. Please describe the circumstances / reasons for receiving the care.

5. *If YES to Tier 1, Q5 (hospitalization)* – You indicated that you recently were hospitalized…

    5a. Where were you hospitalized?

    5b. What were the dates of your hospitalization?

    5c. Please describe the circumstances / reasons for your hospitalization.

6. *If YES to Tier 1, Q6 (physical/mental symptoms interfering with ADLs)* – You indicated that you recently experienced physical and/or mental health symptoms that interfered with your daily activities…

    3a. Please describe the symptoms you experienced and how they interfered with your daily activities.

    3b. When did the symptoms begin?

    3c. When did the symptoms end or are you still experiencing them?

    3d. To what extent are they interfering with your daily activities, would you say, {INSERT LIKERT SCALE HERE}

**TIER 2 SAFETY CHECK-IN, Part 2** (Britton and Lindahl 2018)					
**In the last { 2 weeks / month / 6 months }, please indicate how often you have experienced the following:**					
	**Never**	**Rarely** (once per week)	**Sometimes** **(**2-3 times per week)	**Often** (once a day)	**Very often** (several times/day)
1. I had trouble thinking clearly and/or making decisions					
2. I felt anxious					
3. I experienced repeated, disturbing memories, thoughts, or images of a stressful experience from the past					
4. I was bothered by little things					
5. I had trouble enjoying things that I used to enjoy					
6. I felt distant or cut off from other people					
7. I had difficulty sleeping					
8. I experienced headaches and/or body pain					
9. My hearing has become very sensitive					
10. Feeling disconnected from everything around you					
11. Other significant symptoms (describe) ______________________________					

Scale information: Britton, W.B., and Lindahl, J.R. (2018)

The scale represents the 10 most common meditation-related symptoms from the Varieties of Contemplative Experience Phenomenology Codebook (Lindahl et al 2017 *PLOS One*), as replicated in an NIH–funded MBI clinical trial (Britton, in prep), assessed by PROMIS, Neuro-QoL, and other validated questionnaire items, which were chosen by a modified Delphi method.

Thank you for completing this survey! Your safety is important to us. You may receive a phone call from a trained staff member to follow up with you regarding these event(s).

However, please note that your responses may not be reviewed immediately. Everyone participating in this research study will receive a list of contacts if they want to speak to someone about any health concerns or abuse. Resources are shown below if you feel that you would like to talk with someone immediately for assistance.

National Suicide Prevention Lifeline: 1-800-273-8255
National Sexual Assault Hotline: 1-800-656-4673

Other options are to:

- Call your doctor's office
- Call 911 for emergency services
- Go to the nearest hospital emergency room

Appendix A2

Adverse Event Form

Adverse Event Form

STUDY NAME

**Site Name:**___________________________<br>**Pt_ID:**_________________________	**This form is cumulative and captures adverse events of a single participant throughout the study.**

Severity	Study Intervention Relationship	Action Taken Regarding Study Intervention	Outcome of AE	Expected	Serious Adverse Event (SAE)
1 = Mild<br>2 = Moderate<br>3 = Severe<br>4 = Very Severe<br>5= Death	0 = Not related<br>1 = Unlikely related<br>2 = Possibly related<br>3 = Probably related<br>4 = Definitely related	0 = None<br>1 = Treatment modification<br>2 = Medical Intervention<br>3 = Hospitalization<br>4 = Treatment discontinued<br>5 = Other	1 = Resolved<br>2 = Recovered with minor sequelae<br>3 = Recovered with major sequelae<br>4 = Ongoing/Continuing treatment<br>5 = Condition worsening<br>6 = Death<br>7 = Unknown	1 = Yes<br>2 = No	1 = Yes<br>2 = No<br>(if yes, complete SAE form)

At end of study only: Check this box if participant had no adverse events ☐ **None**

Adverse Event	Start Date	Stop Date	Severity	Relationship	Action Taken	Outcome of AE	Expected?	SAE?

Appendix A3

Severity Grading Tool

SEVERITY GRADING TOOL		
**Grade**	**Description**	**Detailed Criteria (Linden, 2013; NCI, 2017; OHRP, 2007)**
**1**	**Mild**	• May include transient distress and discomfort, but not enough to cause significant impairment, change in behavior or countermeasures.<br>• Transient or short duration (usually < than 1 week)<br>• Resolved on its own; no intervention needed<br>• No consequences
**2**	**Moderate**	• Minimal, local or noninvasive intervention indicated (individual meeting with meditation teacher)<br>• Significant distress or discomfort that involves:<br>o Countermeasures (non-prescription medicine) OR<br>o Change in behavior (cancelling plans, decreasing or discontinuing treatment) OR<br>o Impairment in at least one domain of functioning[a] or activities of daily living (ADL)[b] (25%-50% reduction from usual level)
**3**	**Severe**	• Clinically/medically significant but not immediately life-threatening; hospitalization or prolongation of hospitalization indicated; disabling; (source: CTCAE)<br>• Health professional/clinical attention needed. Can be outpatient or inpatient.<br>• Significant impairment in at least one domain of functioning[a], activities of daily living[b] or self-care[c] (>50% reduction in usual level)
**4**	**Very severe**	• Life-threatening consequences; urgent intervention indicated (source: CTCAE).<br>• Includes suicidal ideation or attempt<br>• If psychological in nature: requires in-patient hospitalization
**5**	**Death**	• Death
**SERIOUSNESS (requires different reporting process)**		
	**Serious**	• Grades 4 + 5 are serious; Grade 3 may be serious<br>• Results in death<br>• Is life-threatening<br>• Requires hospitalization or prolongs existing stay<br>• Results in congenital anomaly<br>• Causes permanent disability or requires medical/surgical intervention to prevent permanent disability or any of the above.

[a] Domains of functioning = occupational (work), educational (school), social (friendships, community), role (parenting, spouse), cognitive (memory, decision-making).
[b] Activities of Daily Living (ADL) = preparing meals, shopping for groceries or clothes, using the telephone, managing money, etc.
[c] Self care = bathing, dressing and undressing, feeding self, using the toilet, taking medications, and not bedridden.

OHRP. (2007). *Guidance on Reviewing and Reporting Unanticipated Problems Involving Risks to Subjects or Others and Adverse Events*: Office for Human Research Protections, US Department of Health and Human Services.

NCI. (2017). Common Terminology Criteria for Adverse Events (CTCAE) v5.0. *National Cancer Institute, NIH, U.S. Department of Health and Human Services*.

Linden, M. (2013). How to define, find and classify side effects in psychotherapy: from unwanted events to adverse treatment reactions. *Clin Psychol Psychother, 20*(4), 286-296.

Appendix A4
Relatedness Assessment Tool

RELATEDNESS ASSESSMENT TOOL			
	**YES**	**NO**	**unsure**
1. Are the symptoms on the list of known meditation-related experiences?<br>[See attached list of previously reported effects]	**+1**<br><br>[expected]	**0**<br><br>[unexpected]	**+0.5**
2. Do you think these experiences were caused in whole or in part by the study treatment? (Do you think meditation played a causal role?)	**+1**	**0**	**+0.5**
3a. Did the experiences appear for the FIRST time during or following exposure to the study treatment? (Did you ever experience this symptom before the study?= no)<br><br>OR<br><br>3b. Did pre-existing experiences or conditions increase or get worse after the study treatment?	**+1**	**0**	**+0.5**
4. Have these experiences occurred during/following exposure to the study treatment on more than one occasion?	**+1**	**0**	**+0.5**
5. Did the symptom get worse with a higher dose of treatment?	**+1**	**0**	**+0.5**
6. Did these experiences disappear or lessen after you stopped or decreased participating in the study treatment?	**+1**	**0**	**+0.5**
7. Did the experiences reappear or get worse when you started engaging with the study treatment again or increased the amount?	**+1**	**0**	**+0.5**
8. Are there alternative causes (other than exposure to the study treatment) that could have caused the experience *in its entirety*?	**-1**	**+1**	**+0.5**
**TOTAL (sum 1-8)**			
**Scoring:**<br>Not related = -1 to 0<br>Unlikely related = 1<br>Possibly related = 2 OR includes plausible alternative cause (#8)<br>Probably related =3-4<br>Definitely related =5-7			
**Sources:** (Agbabiaka et al., 2008; Gallagher et al., 2011; Hill, 1965, 2015; Naranjo, 1986; Naranjo et al., 1982; Naranjo et al., 1981; Theophile et al., 2010).			

Appendix A5

List of Previously-Reported Meditation Effects

LIST OF PREVIOUSLY-REPORTED MEDITATION EFFECTS

(From Lindahl et al 2017 The Varieties of Contemplative Experience, *PLOS ONE*)

**Affective Domain** (13 Categories)	**Description**
Affective Flattening, Emotional Detachment, or Alexithymia	A narrowed or diminished affective range, a lack of affective charge, and/or an inability to identify/distinguish emotions.
Affective Lability	Rapid shifts in mood, mood swings, a increased range of emotions, or strong, unwarranted reactions to situations.
Agitation or Irritability	An agitated or irritable mood, possibly accompanied by restlessness, distractibility or uneasiness.
Change in Doubt, Faith, Trust, or Commitment	Changes (increase or decrease) in doubt, faith, trust or commitment in relation to religious doctrines, practices, goals, community or in relation to oneself in any dimension of life, such as self-confidence.
Crying or Laughing	Crying and laughing, and associated vocalizations.
Depression, Dysphoria, or Grief	Low, depressed, or sad moods, usually coupled with physical and behavioral manifestations that may or may not affect normal functioning.
Empathic or Affiliative Changes	Increased or decreased empathic connection to other people or to environmental stimuli.
Fear, Anxiety, Panic, or Paranoia	Feelings of fright or distress--with or without an external referent--and their corresponding physiological and behavior responses.
Positive Affect	A state of positive or elevated mood or energy level, ranging on a continuum from low to high arousal.
Rage, Anger, or Aggression	Feelings of intense displeasure or a retaliatory response, often caused by some adverse stimulus provoking an uncomfortable emotion.
Re-experiencing of Traumatic Memories or Affect Without Recollection	Either a recollection of some past traumatic event in the subject's life that may or may not have been repressed, and which is generally associated with strong emotions, or the upwelling of strong emotions without any corresponding memory, content, thought or other identifiable stimulus.
Self-Conscious Emotions	Emotions relating to one's sense of self and identity, as well as the awareness of reactions of others to oneself, whether real or imagined.
Suicidality	Affect-driven ideation concerning wanting to die, not wanting to continue with life, wishing to no longer being alive, thinking about taking one's own life, or thinking about or making specific plans for taking one's own life.

**Cognitive Domain** (10 Categories)	**Description**
Change in Executive Functioning	Either an inability to perform cognitive functions of decision making, concentration, and memory that the person used to be able to perform, or an enhanced ability in these domains of executive functioning.
Change in Worldview	A shift in ways of thinking about the nature of self or reality, including a change in understanding or confusion about the nature of self or reality.
Clarity	Reports of clarity or lucidity as a mental state, quality of attention, or quality of consciousness, in which there is a heightened cognition of relevant stimuli and a diminished interference from non-relevant stimuli.
Delusional, Irrational, or Paranormal Beliefs	Holding with conviction and being influenced by one or more beliefs despite evidence to the contrary. Ascriptions of significance or meaning that are later disregarded or that might seem unusual or concerning to members of the practitioner's broader culture or particular subculture. Attributions of paranormal agency, origin, or explanation for cognitive experiences.
Disintegration of Conceptual Meaning Structures	Percepts arise but are processed without their associated conceptual meaning, resulting in an inability to form conceptual representations of the perceptual world.
Increased Cognitive Processing	Primarily a cognitive change in thought amount or speed of cognitive processing, though the increase in processing often coincides with a decrease in sensory gating that leads to the impression of taking in or processing more perceptual information than usual.
Mental Stillness	An state in which there are few identifiable thoughts, a perceived absence of thought, or a poor awareness about the thinking process in general.
Meta-Cognition	Meta-cognition, or meta-awareness, refers to an explicit knowledge of the content of thoughts or the thinking process. Meta-cognition can also entail a higher-order cognition of processes in other domains of experience, such affective, perceptual, somatic or sense of self.
Scrupulosity	Obsessive thinking, specifically about moral or religious issues and behaviors.
Vivid Imagery	An experience of intense, vivid and/or clear thoughts or mental images that arise involuntarily, or a report of an increased ability to visualize.
**Conative Domain** (3 Categories)	**Description**
Anhedonia or Avolition	Anhedonia is the inability to experience pleasure in activities previously found pleasurable. Avolition is the lack of drive or motivation to pursue goals previously valued as meaningful.
Change in Effort or Striving	The degree or intensity of attempts at pursuing something valued-as-good or as a means to a valued end. Effort may be mental, physical, or emotional.
Change in Motivation or Goal	The reasons, drives, and needs behind a practitioner's actions, which influence or determine their behavior, as well as their expectations concerning a particular behavior.

**Perceptual Domain** (7 Categories)	**Description**
Derealization	Surroundings are perceived as strange, unreal, or dreamlike, or perception is experienced as mediated by a fog, a lens, or some other filter that results in feeling cut off from the world.
Dissolution of Objects or Phenomena	The dissolving or complete disappearance of visual objects or the entire visual field.
Distortions in Time or Space	An alteration in the subjective experience of spatial boundaries or relations and/or temporal causality or sequencing.
Hallucinations, Visions, or Illusions	A hallucination is an experience of a percept that is not externally stimulated, is not shared by others, and is not taken to be veridical. When a visual percept that is not shared by others is taken to be veridical, it is a vision. An illusion involves a percept that is distorted, changed, or has features added to the raw percept.
Perceptual Hypersensitivity	Unusual or atypical sensitivity to certain frequencies or volumes of sound (hyperacusis), to color (hyperchromia), to visual details, to light, to taste, to smell, or to embodiment.
Somatosensory Changes	A change in proprioceptive information that affects one's perception of relative positions or dimensions of body parts or the body more generally.
Visual Lights	Experience of a light or lights in field of vision that are vivid but not the result of external stimuli.
**Sense of Self Domain** (6 Categories)	**Description**
Change in Self-Other or Self-World Boundaries	Expansion beyond or distortions in the typical sense of where the boundaries between self and other or self and world are delineated.
Change in Narrative Self	A report of a change in how the practitioner conceives of himself or herself as a person. Or, a change in the content of or their perspective on their story or personal identity.
Change in Sense of Embodiment	Feeling of being disembodied, located outside or at a distance from one's body, or located in an unusual location within one's body schema.
Loss of Sense of Agency	A loss of a sense of ownership or sense of control over one's actions.
Loss of Sense of Ownership	A loss of the usual sense of owning one's thoughts, body sensations, emotions, and/or memories.
Loss of Sense of Basic Self	A loss of the sense of existing, of being a self, or of having a self.
**Social Domain** (5 Categories)	**Description**
Change in Relationship to Meditation Community	Changes in relationship with the meditation community (*Sangha*), whether increasing or decreasing degrees of affiliation with the community of teacher(s) and other practitioners.
Increased Sociality	Increased extraversion, social contact, friendships or other behavioral manifestations indicating an increased valuing of social engagement.

Integration Following Retreat or Intensive Practice	A destabilizing transition from intensive formal practice to informal practice, daily life, or life circumstances.
Occupational Impairment	An impaired ability to perform in an occupational environment.
Social Impairment	Behaviors indicative of a change in relationship to social networks or social situations that inhibits ordinary or desired functioning or level of engagement.
**Somatic Domain** (15 Categories)	**Description**
Appetitive or Weight Changes	Decreased or increased appetite, weight loss or gain.
Breathing Changes	Altered respiration rates that may manifest as a temporary cessation, or speeding up or slowing down of breathing.
Cardiac Changes	Irregular heartbeat, heart palpitations, or other significant irregularities.
Dizziness or Syncope	Dizziness, vertigo (feeling one is spinning or off-balance), lightheadedness (feeling one is about to faint), or syncope (a brief loss of consciousness and muscle strength, commonly called fainting, passing out or blacking out).
Fatigue or Weakness	A feeling of exhaustion, fatigue or weakness (general or localized).
Gastrointestinal Distress or Nausea	Gastrointestinal problems including (but not limited to) diarrhea, bloating, cramping, nausea and vomiting.
Headaches or Head Pressure	Ache, sharp pain, or pressure in the region of the head or neck.
Involuntary Movements	A motor movement usually under voluntary control that occurs without a conscious decision for movement.
Pain	Pain is an unpleasant physical sensation, either diffuse or acute, and lasting for variable amounts of time.
Parasomnias	Nightmares, vivid dreams, sleep paralysis or the alleviation of these symptoms.
Pressure, Tension or Release of Pressure, Tension	Bodily pressure or tension, or release of bodily pressure or tension, that can vary according to location (general or specific), intensity, or length of time.
Sexuality-Related Changes	Hypersexuality (very frequent or suddenly increased sexual urges or activity) or hyposexuality (notably decreased sexual urges or activity).
Sleep Changes	Changes in sleep amount, sleep need, or sleep depth.
Somatic Energy	A type of sensation moving throughout the body or throughout a body area described with language of vibration, energy, current, or other related metaphors.
Thermal Changes	Changes associated with heat or cold, whether a general change in sense of body temperature or localized to a specific body area.

Appendix A6

Serious Adverse Events Form

National Center for Complementary and Integrative Health

Serious Adverse Event (SAE)
Report Form

STUDY NAME

Protocol Number:___________________________

Site Name:____________________________

Pt ID:__________________________

Date Participant Reported:

__ __ / __ __ __ / __ __ __ __
d d m m m y y y y

1. SAE onset date: ___ ___ / ___ ___ ___ / ___ ___ ___ ___
   d d m m m y y y y

2. SAE stop date: ___ ___ / ___ ___ ___ / ___ ___ ___ ___
   d d m m m y y y y

3. Location of SAE:____________

4. Was this an unexpected adverse event? ☐ Yes ☐ No

5. Brief description of participants with no personal identifiers:

   Sex: ☐ F ☐ M Age: ________

   Diagnosis for study participation:____

   ____

6. Brief description of the nature of the SAE (attach description if more space is needed):

   ____

   ____

7. Category of the SAE:

   ☐ Date of death ___/_____/_______
   (dd/mmm/yyyy)

   ☐ Life threatening

   ☐ Hospitalization – initial or prolonged

   ☐ Disability/incapacity

   ☐ Congenital anomaly/birth defect

   ☐ Required intervention to prevent permanent impairment

   ☐ Other:________________________________

8. Intervention type:

   ☐ Medication or nutritional supplement (specify):____

   ☐ Device (specify):____

   ☐ Surgery (specify):____

   ☐ Behavioral/lifestyle (specify):____

9. Relationship of event to intervention:

☐ Unrelated (clearly not related to the intervention)
☐ Possible (may be related to intervention)
☐ Definite (clearly related to intervention)

10. Was study intervention discontinued due to event? ☐ Yes ☐ No

11. What medications or other steps were taken to treat the SAE?

____

12. List any relevant tests, laboratory data, and history, including preexisting medical conditions:

____

13. Type of report:

☐ Initial
☐ Follow-up
☐ Final

Signature of principal investigator: ________________________________ Date: _________________

Appendix A7
Attrition Information Form

ATTRITION INFORMATION FORM:

**ID#:**	**Dropout date:**
**Reason for participant dropout (choose 1 and document circumstances)**	
	Participant-initiated; Reason(s)
	Researcher-initiated; Reason(s)
	Unknown

Participant-initiated dropout reason survey (administered online):

**Please indicate how much each of the following influences your decision to leave the study**			
	**Not at all**	**Somewhat**	**Very Much**
1) Logistics (e.g. scheduling and time commitment)<br>Please describe:___________________________			
2) Personal or family reasons (e.g. other commitments related to work/family)<br>Please describe:___________________________			
3) Dissatisfied with treatment<br>Please describe:___________________________			
4) Poor mental health<br>Please describe:___________________________			
5) Poor physical health<br>Please describe:___________________________			
6) Other<br>Please describe:___________________________			

Research-initiated dropout reason survey (for researcher/staff)

Please describe reason(s) why participant was withdrawn from the study and/or analysis:

Appendix I – Revised Informed Consent

(v.3.2 – December 13, 2018)

with Track Changes highlighting revisions

BROWN

BROWN UNIVERSITY

CONSENT FOR RESEARCH PARTICIPATION

The Mindfulness-Based Blood Pressure Reduction (MB-BP) Study

Version 3.2, December 13, 2018

Deleted: 3.1, October 11, 2018

KEY INFORMATION:

You are invited to take part in a Brown University research study called MB-BP. Your participation is voluntary.

- PURPOSE: In this study, we are looking to see if mindfulness practices improve blood pressure, and if education about hypertension risk factors may also improve blood pressure.
- PROCEDURES: All enrolled participants will be asked to participate in multiple health assessments before and after the mindfulness class. We will also ask you to use a home blood pressure monitor to take your blood pressure at home nine times throughout the study. If eligible and selected to receive the intervention, you will be asked to take part in a 9-week mindfulness course where you will free receive training in meditation, mindful movements, and the roles of things like diet, physical activity and medication in reducing blood pressure. Control group participants will be offered the mindfulness class after their six month follow up assessments are complete.
- TIME INVOLVED: The mindfulness class involves 30 hours of class time and up to 48 hours of at home practice spread out of the course of 9-weeks. The three research assessments are estimated to take around four hours each or around 12.5 hours in total. The assessments will take place at three times throughout the study: at baseline before class begins and then at 10 weeks and 6 months follow up. The total estimated time involved for this study is up to 90.5 hours spread out over 6 months. An abbreviated 1 year follow up assessment will also be administered to individuals randomized to the intervention group to assess long term effects of the mindfulness class.
- COMPENSATION: You will receive $50 USD per follow up (i.e., 10 weeks, 6 month, and 1 year for intervention group; and 10 weeks and 6 month for control group) as compensation for your time (up to $150 USD in total for intervention group and up to $100 USD in total for control group). You will be given a wireless home blood pressure monitor to keep for completing your baseline assessment.
- RISKS: The risks to you in this study are small. They include possible discomfort during research assessments and/or the 9-week intervention as well as possible increases in anxiety, depression, or insomnia or physical injury during the mindfulness intervention. All aspects of the study are voluntary.
- BENEFITS: There are no guaranteed direct benefits to participating in this study. We are investigating whether or not the 9-week intervention actually works to lower blood pressure.
- ALTERNATIVES TO PARTICIPATION: A number of different therapies, including antihypertensive medication, diet changes, physical activity, and reducing excessive alcohol consumption may also be beneficial for reducing blood pressure. Education about these therapies are integrated into this course, but other forms of these alternative therapies are also available in the community.

Commented [SF1]: Added as a result of the updated DSMP.

This remainder of this form will explain in detail the purpose of the study, how the study will be carried out and what you will be expected to do. It will also explain the possible risks and possible benefits of being in the study. If any part of the following description is not clear to you, you are encouraged to contact the researcher to answer any questions before you decide whether to take part in the study. If you decide to participate, please fill out and sign the last page of this form.

Deleted: *3.1, October 11, 2018*

1. **Researcher(s):**

The Principal Investigator on this project is Dr. Eric B. Loucks. He can be reached at 401-863-6283 or by email at eric.loucks@brown.edu. Research staff working on the study can be contacted by phone or email at: 401-400-4768 and mindfulness@brown.edu.

2. **What is this study about?**

The purpose of the study is to investigate the impact of mindfulness practices and health education on blood pressure. You have been selected for this study because you expressed interest in the project and because you met entrance criteria for having prehypertension or hypertension, or another cardiovascular risk factor that could be influenced by this program. Eligibility for the study is still being assessed. Therefore, it is possible you may not be eligible for the study even after signing this consent form. Your participation in this study is voluntary and can be withdrawn at any point in the project.

In order to assess the effects of the customized mindfulness intervention, you will be asked to complete some questionnaires and laboratory assessments before and after the intervention. Specifically, assessments will be completed at: baseline, 10 weeks, and 6 months. To express our gratitude for participation, you will be given $50 at each of the follow ups ($100 USD total). As part of the study you will also be given a wireless blood pressure monitor (estimated value of $90) to use throughout the study. Members of the intervention group will also be asked to complete an abbreviated 1 year follow up assessment estimated to take 45-60 minutes to complete in order to assess long term effects of the mindfulness intervention.

This is a Randomized Control Trial. Participants enrolled into the study will be randomly assigned to one of two groups: (1) the intervention group or (2) the wait-list control group. The wait-list control group will be given the opportunity to participate in the intervention after the six month follow up assessments are completed. Both the intervention and the control group will be asked to participate in the research assessments.

3. **What will I be asked to do?**

If you agree to participate, you will be asked to consent to the following:

a) Participation in a screening interview in which you will be asked questions about past and present mental health, including depression and suicide (previously completed with your verbal consent).

b) Completion of an in-person screening assessment, during which your blood pressure, height, weight and other basic demographic and health data will be collected and assessed in order to determine eligibility for the study.

c) Completion of questionnaires administered in-person and online that ask about a wide range of topics, including your diet, physical activity, smoking, medication use, personality, emotions, attention and past experiences, including stressful or traumatic experiences. These questions will probe sensitive psychological areas, including physical, emotion and sexual abuse. These questionnaires may take up to 3 hours to complete. By completing the interviews and questionnaires, you are giving the researchers permission to use the information you have provided. You have the right not to answer any of the questions.

d) Directly assessed blood pressure, heart rate, height, weight, physical activity, and antihypertensive (blood pressure) medication use at baseline and after the mindfulness course. If you take antihypertensive medication, we will provide you with an electronic bottle cap that will automatically record when the pill bottle is opened during the study. This will help us measure

how often the medication is used. We will also provide you with a wireless blood pressure monitor and will ask that you take your blood pressure at home systematically during each of the research assessment periods (i.e., baseline, 10 weeks, and 6 months).

e) You will be asked to perform some cognitive tasks. Some of these tasks may involve computer-based tests of attention or decision-making. Together these tests may take as long as 20 minutes.

f) During the in-person assessments you will also be given a battery of stress tests that are designed to induce a stress response so that we can monitor your cardiovascular response and recovery.

g) There will be two follow ups that take place after 10 weeks and 6 months from the start of the intervention. The assessments and questionnaires you will complete for the follow ups will be the same as those completed at baseline. An abbreviated 1 year follow up will also be administered to members of the intervention group.

If randomized into the intervention group…

h) You will be asked to participate in the mindfulness program, which consists of 9 weekly sessions of 2.5 hours each and will include one 7.5 hour weekend retreat. Daily at home practice assignments may take as long as one hour and consist of practicing mindfulness exercises with the aid of a guided meditations and completing worksheets related to stress, thoughts, and common reactions to various types of events. Individuals randomized into the wait-list control group, will be invited to take part in the mindfulness class after the completion of the 6 month follow up assessments.

i) Class sessions may be video and/or audio recorded for training purposes and in order to analyze the quality of the treatment you receive. Class videotapes may be made available to MB-BP supervisors, teacher trainers, teacher trainees and researchers through the Mindfulness Center at Brown University. It may be used by them for professional education and training purposes via various secured outlets, including a password-protected and private YouTube channel, a password-protected and private DropBox account, and electronic academic learning software tools. The video monitor will focus on the face of the course instructor, and not on the faces of study participants. However, there is a possibility that your voice and face may be recorded. The study staff will have you review and sign a separate media permission form regarding this study requirement.

j) You may be asked to complete a few short questionnaires each week during the 9 week condition.

k) Additionally, if you are randomly selected to the waitlist control group, we will be asking you to refrain from initiating and engaging in mindfulness practices and formal meditation during the six-month period of your participation in this research study. As part of this research study we are evaluating the impact of the mindfulness course on the health of its participants. If the control group members engage in mindfulness during the intervention period, it may misguide the study results.

Commented [SF2]: This is a new component to UH3. Although we would never remove someone from the actual study if they choose to engage in mindfulness, we may end up having to exclude them from the analyses. To avoid this, we will be reviewing this piece with all participants during the informed consent process.

Table Summarizing Activities and Time Commitment for this Study.

Activity	Estimated Time Commitment
In-person screening assessment	0.75 hours
Baseline<br>In-person assessment<br>Online questionnaire<br>At home health monitoring (i.e., blood pressure)	 2.5 hours<br>0.75 hours<br>0.25 hours
Intervention*<br>Mindfulness course<br>Home practice assigned during course<br><br>**Intervention group only; control group will be invited to take part in a class post 6 month follow up but it will not be required as part of the study.*	Nine 2.5 hour sessions<br>7.5 hour all day retreat<br>Up to 1hr daily home practice assignments<br>*Total course time: 30.0 hours*<br>*Max. practice time: 48 hours*
Follow Ups – 10 week ($50) and 6 month ($50)<br>In-person assessments<br>Online questionnaires<br>At home health monitoring (i.e., blood pressure)<br>1 year follow up (*intervention group only*) ($50)	Total follow up time:<br>5.0 hours<br>1.75 hours<br>0.5 hours<br>Up to 1.0 hours
**TOTAL ESTIMATED TIME COMMITMENT**	11.5 hours – CONTROL<br>90.5 hours - INTERVENTION

Your participation in this study may last up to 6 months in duration (1 year for intervention group members) and is estimated to take up to 11.5 or 90.5 hours in total depending on which group you are assigned to.

Feedback: At the end of the study, you will receive individual feedback about the changes that occurred since the first assessment. Specifically, you will receive an individualized handout listing % change (increase or decrease) on scales of attention, stress, mood, health behaviors, weight, and blood pressure across the study.

Uncontrolled Hypertension: If during the in-person assessments it appears that you have stage 2 uncontrolled hypertension (your average systolic blood pressure reading is 140 mmHg or greater and/or your average diastolic blood pressure is 90 mmHg or greater) AND you indicate to us that you are not currently being treated for hypertension, then we will be requesting your permission to contact your health care provider to notify him/her of the blood pressure results. If you do not have a health care provider and/or do not have health insurance, our staff will provide you with resources to help you search for one; although we cannot guarantee that we will be able to find you one nor that it will be free. It is your choice on whether or not you would like us to follow up with your health care provider. Your participation in the study is not contingent on this communication; however, it is our recommendation that all individuals with uncontrolled hypertension be under the care of a health care professional.

4. Will I be paid?

You will be given $50 at each of the follow up assessments ($100 USD total for control group members and up to $150 USD for intervention group members).

Deleted: .5
Deleted: 1.0
Deleted: 2.0
Deleted: 75
Deleted: 75
Deleted: 7
Deleted: 7

5. **What are the risks?**

The risks to you in this study are small. The questionnaires used in the study are routine, standardized forms for epidemiologic research. Certain questions may be upsetting as they may probe sensitive psychological areas and inquire about upsetting or traumatic events, including physical, sexual or emotional abuse and/or current psychiatric symptoms. The cognitive tests and stress battery may also invoke a stress response that may be uncomfortable. All aspects of the study are voluntary; you have the right to skip anything during the study that makes you uncomfortable.

Meditation-based interventions may results in discomfort with attention to unpleasant thoughts, feelings or body sensations. Some individuals may experience re-experiencing of traumatic memories, increased anxiety, depression or insomnia.

Commented [SF3]: Added as a result of the updated DSMP.

It is possible that injuries could be sustained during the study either from the gentle mindful movements (i.e., yoga), or from physical activities that participants engage in as a way to reduce blood pressure. To help limit this, you will receive a handout showing the yoga poses that will be offered during the course that you can show your health care provider so that they can advise on which poses to do, and which to avoid. Modifications of poses will be available as needed. None of the poses (or the yoga as a whole) are mandatory to be done. You will also be encouraged to explore physical activities that promote strength and conditioning as a way to reduce blood pressure. You will be encouraged to not go beyond any physical limits of your body, and will be encouraged to ask your healthcare provider about advised physical activities and mindful movements if you have any physical limitations.

While physical and mental injury is always a possibility the potential for harm is limited. Note that a research injury is any physical or mental injury or illness caused by your participation in the study. If you are injured by a medical treatment or procedure that you would have received even if you were not in the study, that is not a research injury. To help avoid research injury and potential added medical expenses, it is important to follow all study directions carefully. If you are covered by insurance and suffer a research injury, it is possible that some or all of the costs of treating your condition could appropriately be billed to your insurance company. If such costs are not covered your health insurance company, it is possible you would have to pay for these costs out of pocket. Brown University's policies do not cover payment for such things as lost wages, medical care expenses, or pain and suffering.

Precautions should be taken to avoid injuries. If you do become injured during the study, you should call your doctor immediately. You should also alert the study staff that you have been injured. Heart attack and sudden death related to heart problems have been known to occur in people while they are exercising. This is very rare, however. Estimates of sudden cardiac death range from 0 to 2 per 100,000 hours. However, the researchers cannot guarantee that no complications will happen to you.

The mindfulness classes may be video and/or audio recorded for training and educational purposes. The video monitor will focus on the face of the course instructor, and not on the faces of study participants. However, there is a possibility that your voice and face may be recorded.

6. **What are the benefits?**

We cannot and do not guarantee or promise that you will receive any direct benefits from this study. However, participation in the study creates the potential benefit of a) identifying effective treatments for elevated blood pressure, b) gaining knowledge of the effects of mindfulness practices, c) receiving information about your psychological and physical functioning. As part of the study, you will receive a wireless blood pressure monitor that will be yours to keep. This monitor may provide additional

opportunity to monitor your blood pressure at home, which may benefit your health by providing additional biofeedback.

7. How will my information be protected?

Your responses for this study will be kept confidential. All data that we collect will be linked to a study ID# instead of your name. All questionnaires in this study will be filled out through an online survey or a paper version of the survey if you prefer. All of these questionnaires will be linked solely to your study ID#, so that your identity is protected and your answers are confidential. All paper forms and data collection tools will be kept in a locked filing cabinet in a secure location. Study consent forms, including this one, will be kept in a locked filing cabinet separate from the research data. All electronic data files containing identifying information will be encrypted with a cloud-based software. Note that although these measures have been taken to protect your personal information, complete confidentiality cannot be guaranteed when transmitting information over the internet.

While your confidentiality is protected to the extent of the law, there are limitations to confidentiality. If your questionnaire responses indicate that you pose a serious danger to yourself or to another person, then a collaborator (Dr. Ellen Flynn) who is a licensed psychiatrist, may contact you to discuss your responses and possible referral to a treatment provider. Questionnaire items that may warrant follow-up include endorsements of statements about hurting yourself, any high scores in depression, anxiety, or other clinically significant problems. You should also know that there are times when the law might require the release of your responses without your permission. For example, State law requires researchers to report abuse or neglect of children to the Department of Children, Youth and Families (DCYF). State law also requires researchers to report abuse or neglect of people age 60 and older to the Division of Elderly Affairs.

The findings of the study may be used for medical publication. Your name will not be used in any published reports about this study. Results will be reported in a summarized manner in such a way that you cannot be identified. All personally identifiable information will be "de-identified" and only a unique code number will be used. Study records will be identified with a unique code number and initials. All study records and specimens will be stored in a secure storage area.

Keeping study records: The Principal Investigator for this study will keep your research records indefinitely for research purposes.

Certificate of Confidentiality: This research is covered by a Certificate of Confidentiality from the National Institutes of Health. The researchers with this Certificate may not disclose or use information, documents, or biospecimens that may identify you in any federal, state, or local civil, criminal, administrative, legislative, or other action, suit, or proceeding, or be used as evidence, for example, if there is a court subpoena, unless you have consented for this use. Information, documents, or biospecimens protected by this Certificate cannot be disclosed to anyone else who is not connected with the research except, if there is a federal, state, or local law that requires disclosure (such as to report child abuse or communicable diseases but not for federal, state, or local civil, criminal, administrative, legislative, or other proceedings, see below); if you have consented to the disclosure, including for your medical treatment; or if it is used for other scientific research, as allowed by federal regulations protecting research subjects.

The Certificate cannot be used to refuse a request for information from personnel of the United States federal or state government agency sponsoring the project that is needed for auditing or program evaluation by the National Center for Complementary and Integrative Health, which is funding this project. You should understand that a Certificate of Confidentiality does not prevent you from

voluntarily releasing information about yourself or your involvement in this research. If you want your research information released to an insurer, medical care provider, or any other person not connected with the research, you must provide consent to allow the researchers to release it. The Certificate of Confidentiality will not be used to prevent disclosure as required by federal, state, or local law of situations of child abuse and neglect, or harm to self or others.

Finally, Brown University staff sometimes review studies like this one to make sure they are being done safely and correctly. If a review of this study takes place, your records may be examined. The reviewers will protect your confidentiality.

8. Are there any alternatives to this study?

A number of different therapies, including antihypertensive medication, diet changes, physical activity, and reducing excessive alcohol consumption may also be beneficial for reducing blood pressure. Education about these therapies are integrated into this course, but other forms of these alternative therapies are also available in the community.

9. What if I want to stop?

You do not have to be in this study if you do not want to be. Even if you decide to be in this study, you can change your mind and stop at any time. If you refuse to participate in or leave the study, your current or future relationship with Brown University as well as with your physician will; not be affected. If you decide not to participate, or if you quit the study, we will provide you with referrals for alternative treatments, if desired.

10. Who can I talk to if I have questions about this study?

If you have any questions about your participation in this study, you can call the Senior Project Coordinator, Frances Saadeh, at 401-400-4768 or email at mindfulness@brown.ledu. You may also contact the Principal Investigator at any time: Dr. Eric B. Loucks, email: eric.loucks@brown.edu, telephone (401) 863-6283.

A description of this clinical trial will be available on http://www.ClinicalTrials.gov, as required by U.S. Law. This Web site will not include information that can identify you. At most, the Web site will include a summary of the results. You can search this Web site at any time.

11. Who can I talk to if I have questions about my rights as a participant?

If you have questions about your rights as a research participant, you can contact Brown University's Human Research Protection Program at 401-863-3050 or email them at IRB@Brown.edu.

12. Consent to Participate

Your signature below shows that you have read and understood the information in this document, and that you agree to volunteer as a research participant for this study.

You will be offered a copy of this form.

____

Participant's Signature and Date / PRINTED NAME

____

Research Staff Signature and Date / PRINTED NAME

Clinical Intervention Study Protocol

FULL PROTOCOL TITLE

Mindfulness-Based Blood Pressure Reduction: Stage 2a RCT

Study Chairman or Principal Investigator:

Eric B. Loucks, PhD
Associate Professor, Department of Epidemiology
Brown University School of Public Health

Supported by:
The National Center for Complementary and Integrative Health

5UH3AT009145-04

Study Intervention Provided by:

N/A

Sponsor of IND (IDE):

N/A

Tool Revision History

Version Number: 2.1
Version Date: July 6, 2018
Summary of Revisions Made:

- Revised sample size from 202 to 122. The total sample size of n=122 is comprised of n=50 for the MB-BP intervention group; n=50 for the enhanced usual care control group; and n=22 for the exploratory MBSR group. This sample size reflects recruitment during UH3 phase rather than during both the UH2 and UH3 phases.
- Mediation analyses will be performed via meta-analysis of both samples from the UH2 and UH3 phases, for which the participants were assessed using identical methods. This will maximize statistical power for mediation analyses, which require greater power.
- Narrowed primary outcome to one (Multidimensional Assessment of Interoceptive Awareness), in order to minimize issues of multiple statistical testing and maximize statistical power, given the smaller sample size when analyses are restricted to the data collected during the UH3 phase.
- A unique clinicaltrials.gov registration will be created based on the UH3-specific aims, data collection, and analyses.

Version Number: 2.2
Version Date: August 9th, 2018

Summary of Revisions Made: Replaced Dr. Wen-Chih Wu with Dr. Gaurav Choudhary as the clinical cardiologist on the DSMB. This was done because Dr. Wu and Dr. Loucks have published together in the past three year. Dr. Choudhary and Dr. Loucks have not published together.

Version Number: 2.3
Version Date: August 16, 2018
Summary of Revisions Made:

- Addressed comments and requested revisions per OCRA 8/9/18 review. Comments were relayed by Program Director, Merav Sabri, to study PI, Eric Loucks in an email sent on August 10, 2018.
- Revised Appendix B as requested by OCRA. Also removed sub-Appendix A, Conducting Orientation Sessions at the Center for Mindfulness in Medicine, Health Care and Society University of Massachusetts Medical School, as it is not utilized in this study
- Updated phone screener document (Appendix C) to reflect OCRA review comments and updated study protocol.
- Replaced informed consent form (Appendix D) with updated version (v.3.2)

Version Number: 2.4
Version Date: November 14, 2018

Summary of Revisions Made: The modifications made to the study protocol outlined below were largely the result of items brought to the research team's attention during the NCCIH/Westat September 13, 2018 Site Initiation Visit (SIV) and in the related SIV Report sent on October 9, 2018.

- Updated the study team roster to include the co-PI (King); data safety monitoring expert (Britton); and the study clinicians (Wu and Flynn).
- Clarified the role UMass Medical School will play in conducting the fMRI imaging study and explicitly stated that the details of said study will be documented separately.
- Removed the MBSR exploratory arm. (Refer to Nov. 6, 2018 PI email to NCCIH PO for rationale. NCCIH provided written approval in Nov. 12, 2018 email response to PI)
- Revised (clarified) descriptive language around recruitment; screening; inclusion criteria; blinding; randomization procedures; intervention timing, duration, and content; assessment windows; and data management to more accurately reflect the plan for UH3 study procedures.
- Updated the full list of measures for UH3, which included the removal of: actigraphy devices, Mindful Skill Acquisition scale, dietary self-efficacy, and the readiness to change for hypertenstive risk factors questionnaire. Additionally, the following measures were added: the PROMIS Global Health v.1.2 scale, childhood food insecurity questionnaire, alcohol consumption, mindfulness home practice questions, the Connor-Davidson Resilience scale, the Self-efficacy for chronic disease management questionnaire, and 6-month semi-structured exit interview questions.
- Revised Section 7 (Safety Assessments) to reflect the new Data safety and monitoring plan (DSMP) put forth for UH3.
- Clarified language around the estimated enrollment and analyzable sample size for the fMRI Study.
- Corrected clerical and grammatical errors and cleaned up the language to be more concise and clear.

Version Number: 2.5

Version Date: December 11, 2018

Summary of Revisions Made: Addressed the comments and requested clarifications sent to study PI (Loucks) by NCCIH Program Director (M. Sabri) on December 6, 2018 at 6:18 PM EST. The specific revisions include:

- Adding clarifying language on study randomization and cohort size to section 6.2.4 (Randomization and Intervention Allocation).
- Expounding upon circumstances that might possibly lead to Investigator-initiated withdrawals, which are discussed in section 7.3.3. of the Safety Assessment section of the protocol.

TABLE OF CONTENTS

Page

Clinical Intervention Study Protocol 1
FULL PROTOCOL TITLE 2
Tool Revision History 3
TABLE OF CONTENTS 5
MB-BP STUDY TEAM ROSTER 8
PARTICIPATING STUDY SITES 9
PROTOCOL SUMMARY 9
1. STUDY OBJECTIVES 12
1.1 Primary Objective 12
2. BACKGROUND AND RATIONALE 12
2.1 Background on Condition, Disease, or Other Primary Study Focus 12
2.2 Study Rationale 15
3. STUDY DESIGN 15
4. SELECTION AND ENROLLMENT OF PARTICIPANTS 17
4.1 Inclusion Criteria 17
4.2 Exclusion Criteria 17
4.3 Study Enrollment Procedures 17
5. STUDY INTERVENTIONS 18
5.1 Interventions, Administration, and Duration 18
5.2 Handling of Study Interventions 19
5.3 Concomitant Interventions 19
5.4 Adherence Assessment 19
6. STUDY PROCEDURES 21
6.1 Schedule of Evaluations 21
6.2 Description of Evaluations 22
6.2.1 Screening Evaluation and Consenting Procedure 22
6.2.2 Enrollment 22
6.2.3 Baseline Assessments 22
6.2.4 Randomization and Intervention Allocation 24

6.2.5 Blinding ... 25
6.2.6 Follow-up Visits ... 25
6.2.7 Completion/Final Evaluation ... 25
7. SAFETY ASSESSMENTS ... 25
7.1 Expected Risks and Specification of Safety Parameters ... 25
7.2 Methods and Timing for Assessing, Recording, and Analyzing Safety Parameters ... 28
7.3 Adverse Events and Serious Adverse Events, Reporting Procedures, and Follow-up28
7.3.1 Mental Health ... 29
7.3.2 Physical Health ... 30
7.3.3 Other ... 30
7.4 Safety Monitoring ... 31
8. INTERVENTION DISCONTINUATION ... 33
9. STATISTICAL CONSIDERATIONS ... 33
9.1 General Design ... 33
9.2 Sample Size and Randomization ... 33
9.3 Definition of Populations ... 35
9.4 Interim Analyses and Stopping Rules ... 35
9.5 Outcomes ... 35
9.5.1 Primary Outcome ... 35
9.5.2 Secondary Outcomes ... 35
9.6 Data Analyses ... 35
10. DATA COLLECTION AND QUALITY ASSURANCE ... 36
10.1 Data Collection Forms ... 36
10.2 Data Management ... 36
10.3 Quality Assurance ... 36
10.3.1 Training ... 36
10.3.2 Quality Control Committee ... 36
10.3.3 Metrics ... 36
10.3.4 Protocol Deviations ... 38
10.3.5 Monitoring ... 38
11. PARTICIPANT RIGHTS AND CONFIDENTIALITY ... 38

11.1 Institutional Review Board (IRB) Review 38
11.2 Informed Consent Forms 38
11.3 Participant Confidentiality 38
11.4 Study Discontinuation 38
12. COMMITTEES 39
13. PUBLICATION OF RESEARCH FINDINGS 39
14. REFERENCES 39
15. SUPPLEMENTS/APPENDICES 55
Appendix A MB-BP Curriculum Guide 55
Appendix B MBSR Curriculum Guide 55
Appendix C MB-BP Phone Screener (verbal consent process) 55
Appendix D MB-BP Informed Consent 55
Appendix E Anthropometric Quality Control Manual 55
Appendix F Blood Pressure Quality Control Manual 55

MB-BP STUDY TEAM ROSTER

Eric B. Loucks, Ph.D.
Principal Investigator
Brown University School of Public Health
121 South Main St., Providence, RI 02912
Phone: 401-863-6283
Fax: 401-863-3713
Email: eric.loucks@brown.edu

Jean A. King, Ph.D.
Co-Principal Investigator
100 Institute Road, Worcester MA 01609-2280
Phone: 508-831-4677
Email: jaking@wpi.edu

Frances Saadeh, MPH
Senior Project Coordinator
121 South Main St., Providence, RI 02912
Phone: 401-863-6361
Email: frances_saadeh@brown.edu

Yu Li, PhD
Statistician
121 South Main St., Providence, RI 02912
Phone: 401-863-6792
Email: yu_li1@brown.edu

Roee Gutman, PhD
Senior Statistician
121 South Main St., Providence, RI 02912
Phone: 401-863-2682
Email: rgutman@stat.brown.edu

Study Clinicians

Ellen Flynn, MD – Clinical Psychiatrist
Women's Medicine Collaborative
146 West River Street, 3rd Floor, Providence, RI 02904
Phone: 401-793-7020
Email: eflynn@lifespan.org

Wen-Chih (Hank) Wu, MD, MPH – Cardiologist
164 Summit Ave, Providence, RI 02906
Providence, RI 02906
Phone: 401-793-5810
Fax: 401-793-5815
Email: wen-chih_wu@brown.edu

Data and Safety Monitoring Board (DSMB)

Donald Edmondson, PhD, MPH
DSMB Chair
622 W 168th Street, PH 9-317 New York, NY 10032
Phone: 212-342-3674
Fax: 212-305-0312
Email: dee2109@cumc.columbia.edu

Gaurav Choudhary, MD
Providence VA Medical Center
830 Chalkstone Avenue
Providence, RI 02908
Tel: 401-273-7100 ext 2029
Gaurav_Choudhary@brown.edu

Tao Liu, PhD
121 South Main Street, Providence, RI 02912
Phone: 401-863-6480
Fax: 401-863-9182
Email: tliu@stat.brown.edu

Data and Safety Monitoring Expert

Willoughby Britton, Ph.D.
Director, Clinical and Affective Neuroscience Laboratory
Assistant Professor (Research)
Department of Psychiatry and Human Behavior
Warren Alpert Medical School at Brown University
Phone: 520-245-1855
Email: willoughby_britton@brown.edu

PARTICIPATING STUDY SITES

There is one study site, Brown University, carrying out the Mindfulness-Based Blood Pressure Reduction (MB-BP) study protocol outlined in this document. The University of Massachusetts Medical School is conducting the fMRI Imaging study that will recruit and scan a subset of MB-BP study participants (see separate fMRI study protocol and related MOP for full detail).

PROTOCOL SUMMARY

Study Title

Mindfulness Based Blood Pressure Reduction: Stage 2a Randomized Controlled Trial

Objectives

1. ***Impacts of MB-BP on Primary Self-Regulation Targets:*** Identify the impacts of MB-BP *vs*. enhanced usual care on the primary self-regulation target, specifically an assay of self-related processes (MAIA), described in Table 1. We hypothesize that MB-BP will significantly improve the MAIA in directions of better self-regulation, compared to control.
    a. Secondary analyses will evaluate impacts on secondary self-regulation targets including an assay of emotion regulation (DERS), and cognitive processes (SART), described in Table 1.
    b. Exploratory analyses will evaluate engagement of MB-BP *vs*. enhanced usual care with triangulated self-regulation target assays described in **Table 1** such as emotion regulation and stress (Pittsburgh Stress Battery, Perceived Stress Scale), self-related processes (Heart Beat Detection Task, Interoceptive Awareness fMRI Task), and cognitive processes (Mindful Attention Awareness Scale). Measures such as the neuroimaging Interoceptive Awareness fMRI Task will replicate assays in the MINDFUL-PC study.
2. ***Self-Regulation Targets as Mediators of MB-BP Effect on Medical Regimen Adherence and Health Behavior Change:*** Evaluate the degree to which the engagement of MB-BP with self-regulation targets translates into improved prehypertension/hypertension medical regimen adherence, specifically for the Dietary Approaches to Stop Hypertension (DASH)-consistent diet. We hypothesize that MB-BP will increase the DASH diet score compared to control, in participants with low DASH diet adherence at baseline (DASH diet score <5.5), and that the self-regulation primary outcome in Aim 1 (i.e. MAIA) is a significant mediator.
3. ***Further develop an MB-BP therapist manual and training program, including procedures for training, supervising, and evaluating therapists.*** The PI will implement training he receives from the University of Bangor in the United Kingdom in May 2018 to implement the Mindfulness-Based Interventions Teacher Assessment Criteria (MBI-TAC) for MB-BP instructors, which is the most respected quantitative and qualitative tool developed to provide feedback for enhancing MBI teacher effectiveness, and establishing teacher certification.[1-3]

Design and Outcomes

During the UH3 phase, we will perform a Stage 2a[4] two-arm RCT of MB-BP *vs*. enhanced usual care control, enrolling 50 individuals aged 18 years of age and older per group. This is a pragmatic control group designed to inform physicians if MB-BP would be of service to refer patients, compared to enhanced usual care they could provide patients in well-resourced settings. This control group was decided upon through recommendations by the Research Coordinating Center at Columbia University, the Data Safety Monitoring Board, and several collaborating cardiologists and family physician clinician-researchers. Enhanced usual care involves every participant being provided with a validated home blood pressure monitor (Omron, Model PB786N), which has evidence in and of itself to potentially lower blood pressure,[5,6] and is beyond usual care at this time. All participants who have Stage 2 hypertension (blood pressure >140/90 mmHg) will be offered to have their physicians notified, if not already being overseen for uncontrolled hypertension. For participants with uncontrolled hypertension who do not have a physician, we will work with participants to provide access within constraints of their health insurance. Enhanced usual care group participants receive an educational brochure from the American Heart Association entitled “Understanding and Controlling Your High Blood Pressure Brochure” (product code 50-1731).

Blinding

All study staff will be blinded to the participant treatment allocation with the exception of the instructor, individual who performs the randomization, and staff member coordinating participants within each course. All staff performing participant assessments will be blinded to the participant treatment allocation to promote equipoise. Data analyses will be performed by a statistician blinded to treatment allocation type. The data manager will be able to break blinding if needed (e.g. for Data and Safety Monitoring Board).

Intervention Allocation

Stratified randomization will be used, as simple randomization can fail if it creates groups unbalanced for critical features known to affect outcomes.[7,8] Stratified randomization can reduce both types I and II error, improve trial efficiency, and facilitate subgroup and interim analyses.[7] Randomization will be done using an online computer software program known as Research Randomizer (Version 4.0).[9] The randomization process will occur after each new round of enrollment. Study enrollment will continue until target enrollment goals are reached (i.e., n=50 for MBBP intervention group, n=50 for enhanced usual care control group).

Interventions and Duration

Two-arm RCT comparing MB-BP vs. enhanced usual care. Follow-up assessments will take place within pre-defined assessment windows occurring around 10 weeks, 6 months, and 1 year post- intervention commencement. For participants on waitlist enhanced usual care, they will be offered MB-BP after the 6-month follow-up with no additional assessments unless they would like the assessments for follow-up information on changes in their health. Participants in the intervention group will be asked to come back at the 1-year follow up window for an abbreviated follow up assessment. Thus the total length of involvement for study participants will be up to one year from the time of enrollment to the time of the final research assessment. The study intervention lasts 9 weeks, may be nonconsecutive weeks in the event of a holiday or instructor availability, and takes place in the first three months for individuals in the intervention arm.

Sample Size and Population

We anticipate needing to recruit and enroll a total of 100 eligible individuals during Phase 3. This will consist of 50 individuals per group for both the MB-BP intervention arm and the enhanced usual care arm. (see Figure 1 below for detail).

For more detailed discussion of group allocation, stratification factors, and how it influences study population, please refer to Section 6.2.4.

Inclusion/exclusion criteria:

Inclusion Criterion: Elevated blood pressure defined as ≥120 mmHg systolic or ≥80 mmHg diastolic pressure;[10] able to speak, read, and write in English; all adults (≥18 years of age), genders and racial/ethnic groups are eligible to be included.

Exclusion Criteria: Exclusion criteria follow standard guidelines and recommendations:[11] (a) current regular meditation practice (>once/week); (b) serious medical illness precluding regular class attendance; (c) current substance abuse, suicidal ideation or eating disorder, (d) history of bipolar or psychotic disorders or self-injurious behaviors. These participants are excluded

because they may disrupt group participation, require additional or specialized treatment, or are already participating in practices similar to the intervention.

1. STUDY OBJECTIVES

1.1 Primary Objective

1. ***Impacts of MB-BP on Primary Self-Regulation Targets:*** Identify the impacts of MB-BP *vs*. enhanced usual care on the primary self-regulation target, specifically an assay of self-related processes (MAIA) described in Table 1. We hypothesize that MB-BP will significantly improve the MAIA in directions of better self-regulation, compared to control.
   a. Secondary analyses will evaluate impacts on secondary self-regulation targets including an assay of emotion regulation (DERS), and cognitive processes (SART), described in Table 1.
   b. Exploratory analyses will evaluate engagement of MB-BP *vs*. enhanced usual care with triangulated self-regulation target assays described in **Table 1** such as emotion regulation and stress (Pittsburgh Stress Battery, Perceived Stress Scale), self-related processes (Heart Beat Detection Task, Interoceptive Awareness fMRI Task), and cognitive processes (Mindful Attention Awareness Scale). Measures such as the neuroimaging Interoceptive Awareness fMRI Task will replicate assays in the MINDFUL-PC study.
2. ***Self-Regulation Targets as Mediators of MB-BP Effect on Medical Regimen Adherence and Health Behavior Change:*** Evaluate the degree to which the engagement of MB-BP with self-regulation targets translates into improved prehypertension/hypertension medical regimen adherence, specifically for the Dietary Approaches to Stop Hypertension (DASH)-consistent diet. We hypothesize that MB-BP will increase the DASH diet score compared to control, in participants with low DASH diet adherence at baseline (DASH diet score <5.5), and that the self-regulation primary outcomes in Aim 1 are significant mediators.
3. ***Further develop an MB-BP therapist manual and training program, including procedures for training, supervising, and evaluating therapists.*** The PI will implement training he receives from the University of Bangor in the United Kingdom in May 2018 to implement the Mindfulness-Based Interventions Teacher Assessment Criteria (MBI-TAC) for MB-BP instructors, which is the most respected quantitative and qualitative tool developed to provide feedback for enhancing MBI teacher effectiveness, and establishing teacher certification.[1-3]

2. BACKGROUND AND RATIONALE

2.1 Background on Condition, Disease, or Other Primary Study Focus

The World Health Organization reported that suboptimal blood pressure (BP) is responsible for more than half of cardiovascular disease mortality world-wide. Furthermore, greater than half of those with hypertension have uncontrolled BP.[12] *A 2009 Institute of Medicine report recommended prioritizing research to "Compare the effectiveness of mindfulness-based interventions (e.g. yoga, meditation, deep breathing training) and usual care in treating… cardiovascular risk factors."*[13] Evidence-based mindfulness interventions, including Mindfulness-Based Stress Reduction, may have some effects on blood pressure, where a recent meta-analysis and systematic review of 4 randomized controlled trials demonstrated

significant effects, but evidence of heterogeneity in effect sizes.[14] The methodologically highest quality studies had the smallest effect sizes (range 0-5 mmHg).[14] Mindfulness-Based Stress Reduction (MBSR) has been customized to a number of disease processes, such as Mindfulness-Based Cognitive Therapy for patients with recurrent depression, and Mindfulness-Based Relapse Prevention for patients with substance use addictions.[15-17] Effect sizes may be increased by customizing mindfulness interventions to diseases of interest. The same may be true for hypertension, *however mindfulness interventions customized for prehypertensive/hypertensive patients have never been investigated.* *Until methodologically rigorous studies to evaluate customized interventions for hypertension are performed, we will not know if the observed preliminary effects of general mindfulness interventions on blood pressure reduction could be much more effective with a tailored approach.*

The development of effective interventions that enhance the capacity for self-regulation among people with chronic health conditions is a major public health challenge in the United States and worldwide. The process of healthcare system transformation that is underway focuses on moving accountability for healthcare costs to healthcare systems.[18] Meanwhile, in order to survive, healthcare systems must rapidly learn to deliver interventions that can enhance their patients' capacity to self-manage these deadly and costly chronic health conditions.[19] While access to care[20] and health education[21] are essential, a person's capacity for self-regulation is often the primary limiting factor in their ability to adhere to their medical regimen, collaborate on illness self-management, and reduce health risk behaviors.[22-25] Self-regulation refers to the process of managing cognitive, emotional, and self-relevant resources to align mental states and behavior with goals.[26-28] Changing risk behaviors that influence hypertension risk, such as physical inactivity, diet, excessive alcohol use, and poor medication adherence, requires skills for self-regulation that are broadly applicable within multiple environments. A research collaboration that could test methods for engaging self-regulation mechanisms, identify specific target tests, and rapidly integrate these into an empirically-optimized clinical interventions that are ready for implementation and rapid dissemination within health care settings for patients with chronic illness would make a substantial impact on chronic illness management and the entire healthcare system. Mindfulness-Based Interventions (MBIs) have already begun to offer feasible basic building blocks for the rapid integration of an empirically-optimized, trans-diagnostic, self-regulation toolkit into healthcare across multiple contexts and age groups.

Mindfulness meditation, a form of training that involves maintaining a non-judgmental form of attention to immediate experience,[29] has been employed in both clinical and non-clinical settings to facilitate self-regulation and behavior change.[30-34] Mindfulness-Based Interventions (MBIs),[35] sometimes referred to as "3rd Wave Behavioral Therapies,"[36] have become widely used in both clinical and non-clinical settings, including prisons,[37,38] the military,[39-41] and both K-12[42-45] and higher education,[46] and are emerging as a wide-spread, potentially cost-saving,[47] comprehensive methods for enhancing self-regulation in multiple medical contexts. *While MBIs efficacy has been demonstrated for several conditions related to self-regulation,[48-50] the field lacks consensus about the mechanisms through which these interventions engage self-regulatory processes and impact of MBIs on medical regimen adherence and health behavior changes. Without this knowledge, a plethora of MBIs are being developed without systematically building on discoveries of which components of the interventions have the most impact on specific health behaviors and outcomes.* As a result, the interventions are not as rigorous in their design, which may limit their efficacy and account for some of the mixed results found in the literature.

Self-regulation requires both *initiating* behavior change and *maintaining* behavior change,[51,52] implying corrective adjustments originating within a person and taking place as needed in order

to maintain and sustain the intended goal. Self-regulatory failure is primarily determined by deficits in (or depletion of) self-regulatory strength,[53] highlighting the need for interventions that strengthen self-regulation capacity. Current behavioral models lack a full integration of tools for strengthening self-regulation towards both initiation and maintenance of behavior change.[51,52] Strategies focusing on extrinsic rewards or behavioral economics can help change behavior, but intrinsic motivation is ultimately essential to self-regulation.[54] Behavioral and psychological therapies can provide emotional regulation strategies and some even include motivational components;[55,56] however, they generally lack tools to strengthen core cognitive resources, such as attention and inhibitory control, which are necessary to support maintenance of behavior change. *In contrast, MBIs uniquely integrate training in the emotional, motivational, and cognitive aspects of self-regulation within one therapeutic intervention.* By frequently returning attention to the present moment with a specific orientation to experience (curious, open-minded, and accepting), mindfulness practice strengthens the capacity for frequent daily corrective adjustments needed to stay on track with intended goals. Studying how common self-regulation targets influence, and are influenced by, various components of MBIs provides a unique opportunity to develop an easy-to-disseminate, integrated therapeutic intervention optimized with the most potent components for enhancing self-regulation within the context of medical regimen adherence and the initiation and maintenance of health behaviors.

Following recent proposals on the mechanisms of action of mindfulness by our group[30,57] and others,[58-62] *three broad self-regulation domains of mindfulness can be identified:*

(A) Cognitive processes, including attention (i.e. orienting, alerting,[63,64] vigilance,[65] and conversely sleepiness, cognitive fatigue, attentional lapses, and mind-wandering[66]); executive function, conflict monitoring,[67,68] impulsivity and inhibitory control,[69,70] and metacognitive awareness.[71-73] Many studies indicate that meditation training, such as MBIs, engage these cognitive processes.[74-87]

(B) Emotion regulation, which is the capacity to alter the magnitude or duration of an emotional response.[88] Poor emotion regulation impairs the capacity for self-regulation behaviors that support health behaviors[89-92], including medical regimen adherence.[93] MBIs favorably engage measures of emotion regulation such as amygdala activation,[79,94-97] sympathetic hyperarousal,[98-103] and emotional responses to stressful situations,[102,104-111] although opposite findings have also been reported[112-114] which leads to the question (to be addressed by this collaborative) of which conditions affect target engagement.

(C) Self-related processes, including: *(i) self-efficacy* – the belief in one's capabilities to execute the courses of action required to manage prospective situations (such as changing and maintaining a medical regimen),[115,116] which is a central aspect of self-regulation and changes in health behavior;[117,118] *(ii) self-compassion* – the capacity to extend compassion to oneself in instances of perceived inadequacy or failure rather than engaging in either self-destructive behaviors (self-judgment, isolation, rumination) or in permissive, risky behaviors:[119-121] self-compassion has been found to promote health behaviors such as adhering to diets,[122] smoking cessation,[123] physical activity,[124] and seeking medical treatment when needed;[125] *(iii) self-related rumination or mind-wandering* – which may be beneficial in some cases[126-128] but detrimental in others[129-133] and is a central topic in MBI research, by our group[57,134] and others;[135] and *(iv) interoceptive awareness* – the awareness of internal manifestations of emotions and feelings, considered fundamental to the 'experiencing self',[136-141] a particular form of awareness which can be enhanced by the paying of purposeful, nonjudgmental attention ('mindfulness') to inner body sensations,[32,33,142] which is also of major interest in MBI research.[30,62,135,143-160]

Consequently, we propose to conduct a Stage 2a behavioral intervention study to evaluate whether MBSR customized to pre-hypertensive and hypertensive patients has the potential to provide clinically relevant reductions in BP.

2.2 Study Rationale

The MB-BP Study aims to customize the standardized MBSR[161-164] intervention to adult participants with prehypertension or uncontrolled hypertension. Similar to MBSR, the MB-BP intervention consists of eight 2.5-hour weekly group sessions (plus a 2.5-hour orientation session) and an 8-hour one-day session, led by a certified MBSR instructor with extensive cardiovascular disease and hypertension expertise.[165-178] MB-BP builds a foundation of mindfulness skills (e.g. meditation, self-awareness, etc.) through the MBSR curriculum. MB-BP then directs attention towards hypertension risk factors. The unique areas of MB-BP are education on hypertension risk factors, hypertension health effects, and specific mindfulness modules focused on awareness of diet, physical activity, medication adherence, weight loss, and alcohol consumption and their effects on well-being. A curriculum guide has been created based on the standardized MBSR manual (**Appendix A)**, and was further developed through the approaches described above, and sequentially revised based on participant feedback and preliminary findings.[163,164] MB-BP participants learn a range of mindfulness skills including body scan exercises, meditation and yoga. Homework consists of practicing skills for ≥45 min/day, 6 days/week.

3. STUDY DESIGN

We will perform a Stage 2a[4] two-arm RCT of MB-BP *vs*. enhanced usual care control. This is a pragmatic control group designed to inform physicians in well-resourced settings if MB-BP would be of service to refer patients to as compared to enhanced usual care patients. The study is intended to evaluate the impacts of MB-BP on the primary self-regulation outcome of interoceptive awareness (i.e. MAIA) as compared to an enhanced usual care group. Secondary self-regulation outcomes are described in the Objectives section outlined previously. This study also aims to evaluate the degree to which the engagement of MB-BP with self-regulation targets translates into improved prehypertension/hypertension medical regimen adherence, specifically for the Dietary Approaches to Stop Hypertension (DASH)-consistent diet.

The enhanced usual care control group was decided upon through recommendations by the Research Coordinating Center at Columbia University, the Data Safety Monitoring Board, and several collaborating cardiologists and family physician clinician-researchers. Enhanced usual care involves every participant being provided with a validated home blood pressure monitor (Omron, Model PB786N),[179] which has evidence in and of itself to potentially lower blood pressure, and is beyond usual care at this time.[6,180] All participants who have Stage 2 hypertension (blood pressure >140/90 mmHg) will be offered to have their physicians notified, if not already being overseen for uncontrolled hypertension. For participants with uncontrolled hypertension who do not have a physician, we will work with participants to provide access within constraints of their health insurance. Enhanced usual care group participants receive an educational brochure from the American Heart Association entitled “Understanding and Controlling Your High Blood Pressure Brochure” (product code 50-1731).

All research assessments will take place in the Brown University Mindfulness and Cardiovascular Health Lab in Providence, RI. The MB-BP intervention classes will be offered in both a University and community-based setting.

Baseline assessments for all enrolled participants will take place within four weeks of the start of the intervention. Follow-up assessments will take place during the 10 week, 6 month, and 1 year assessment windows, which are defined as: (a) “10 week” follow up assessments are to occur at least one day after and up to five weeks after the end of the intervention (i.e. the Week 8 class). Staff will prioritize completing the assessments within the first three weeks of the assessment window, but will allow for participants to complete up to five weeks after. (b) The “6-month” follow up assessments will be scheduled to occur six months from the orientation class date plus or minus 2.5 weeks. This allows for a five-week data collection period with prioritization given to the three-week window surrounding the official six-month date. (c) The “1 year” follow up assessments are to be completed with the intervention group participants only and will occur one year plus or minus a month after the start of the intervention (i.e., orientation date).

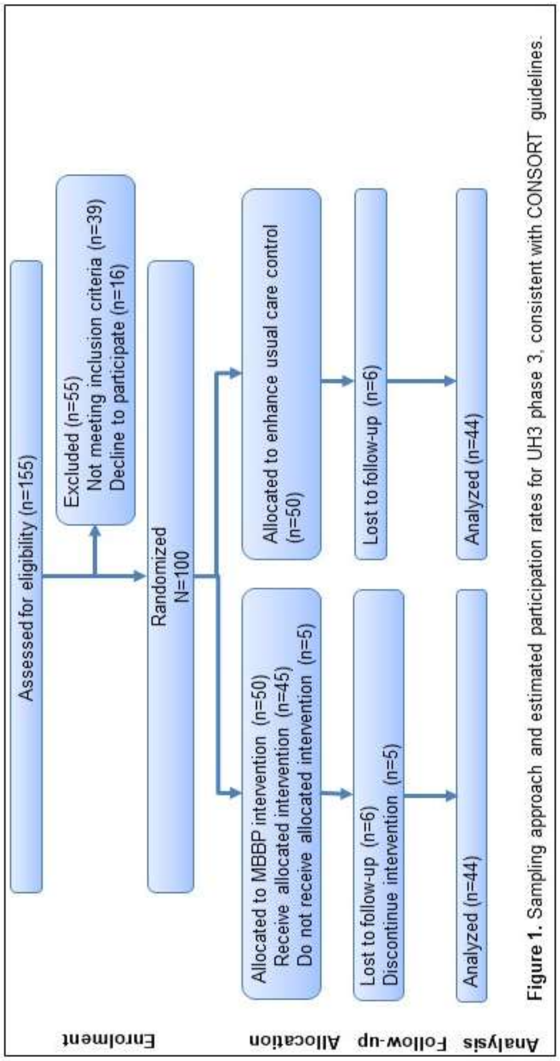

Figure 1. Sampling approach and estimated participation rates for UH3 phase 3, consistent with CONSORT guidelines.

If a participant is unable to complete an assessment within the defined data collection window, but still wishes to participate, we will allow for the participant to complete an online home survey and/or in person follow up assessment beyond the parameters defined above.

Participants who were randomized to the waitlist enhanced usual care group will be offered MB-BP after the 6 month follow-up assessment is complete with no additional assessments unless they would like the final one year assessment for follow-up information on changes in their health. Thus the total length of involvement for study participants will be up to one year from the time of enrollment to the time of the final research assessment. The study intervention lasts 9 weeks and takes place in the first two months for individuals in the intervention arm. The intervention will be offered several times a year over the course of the total study duration.

Prehypertension/Hypertension Medical Regimen Adherence Outcomes Assessment Methods:

Primary Prehypertension/Hypertension Medical Regimen Adherence Outcome: Dietary Approaches to Stop Hypertension (DASH) eating pattern score,[181] measured via 80-item Willet food frequency questionnaire,[182] assessing adherence to American Heart Association/American College of Cardiology (AHA/ACC) clinical practice hypertension guidelines DASH eating pattern score (range 0-8).[10,181,183]

Secondary outcomes of medical regimen adherence include the following: *(1) Alcohol consumption:* Amount and frequency of alcohol consumption, will be assessed via self-report utilizing standard questions from the Behavioral Risk Factor Surveillance Survey.[184] AHA/ACC hypertension clinical practice guideline cut-point of healthy alcohol intake is ≤ 2 drinks (e.g. 24

oz. beer, 10 oz. wine, or 3 oz. 80-proof whiskey) per day in men and ≤1 drink per day in women.[10] *(2) Electronically-Measured Antihypertensive Medication Adherence:* measured continuously using electronic medication bottle caps (eCAPS, Ottawa, Canada)[185] *(3) Body Mass Index:* height and weight directly assessed using standard epidemiologic methods, with change evaluated in participants considered overweight or obese (BMI≥25 kg/m^2).[186] *(4) Physical activity:* measured using the International Physical Activity Questionnaire which has undergone substantial validity and reliability testing.[187-189] Adherence to Joint National Commission-7 (JNC-7) guidelines is 30 min aerobic physical activity ≥4 days per week.[183]

Please see Section 6.2.4 for randomization, blinding and stratification methods.

Please see **Figure 1** for study groups including sample sizes. Please note that sample size recruited for the UH3 phase will be 50 participants per group for MB-BP and enhanced usual care control.

4. SELECTION AND ENROLLMENT OF PARTICIPANTS

4.1 Inclusion Criteria

Elevated blood pressure or hypertension defined as ≥120 mmHg systolic or ≥80 mmHg diastolic pressure.[10] Able to speak, read, and write in English. All adults (≥18 years of age), genders and racial/ethnic groups are eligible to be included.

4.2 Exclusion Criteria

Exclusion criteria follow standard guidelines and recommendations:[11] (a) current regular mindfulness meditation practice (>once/week); (b) serious medical illness or cognitive condition (e.g., dementia) precluding regular class attendance and/or participation; (c) current substance abuse, suicidal ideation or eating disorder, (d) history of bipolar or psychotic disorders or self-injurious behaviors. These participants are excluded because they may disrupt group participation, require additional or specialized treatment, or are already participating in practices similar to the intervention. Additionally, we will be asking participants randomized to the control group to restrain from engaging in any type of formal mindfulness practice more than weekly during the first six months of study involvement so as to not introduce confounding variables. Individuals will be made aware of this requirement at the time of informed consent (first in-person screening). Anyone who is unwilling to follow the treatement requirement would be ineligible for the study.

4.3 Study Enrollment Procedures

Participants will be recruited in part through cardiology and family practices via established relationships with physicians in Rhode Island and Massachusetts. Graduates from the MP-BP program have proven effective at recruiting their contacts. Furthermore, advertisements will be posted throughout Rhode Island and southern Massachusetts, and distributed via social media, inviting participants interested in lowering their blood pressure to enroll. Additionally, we will partner with local providers to recruit hypertensive patients through methods such as direct mailings and targeted recruitment at the Rhode Island Hospital Emergency Department.

Participants are randomly assigned a participant identifier at the time of screening. The key to participant name and identifier is kept in a secure location separate from the research data. The outcome of every screener completed is recorded in a password protected tracking system and reason for ineligibility is noted. Circumstances surrounding situations where eligible participants decline participation and cases where participants enroll but later withdraw are also documented in a tracking system.

Informed consent is collected at the time of the first in-person screener. Individuals wishing to enroll in the study are provided two copies of the written informed consent form which has been approved by the Brown University Institutional Review Board (IRB). In addition to allowing the participant time to read over the consent form, trained research staff review the important points of consent with each participant. The informed consent process is documented. A signed copy of the consent is kept on file in a secure location in the research lab separate from study data and a copy is offered to the participant to keep with his/her personal records. Due to the nature and delivery of the intervention, all participants must be able to read and write in English and be able to provide informed consent for their participation.

5. STUDY INTERVENTIONS

5.1 Interventions, Administration, and Duration

MB-BP Intervention Description: This study proposes to customize MBSR to participants with prehypertension/hypertension creating an intervention called Mindfulness Based Blood Pressure Reduction Study (MB-BP). Specifically, MB-BP is based on the standardized MBSR intervention described elsewhere,[161-164] and will consist of eight 2.5-hour weekly group sessions, a 2.5 hour orientation, and an 8-hour one-day session. MB-BP will be performed by qualified or certified MBSR instructors[190] with formal training in cardiovascular health (e.g. dietician, physician assistant, health and wellness coach, and those with an Associate's or Bachelor's degree in relevant health sciences), and further certification in MB-BP. MB-BP instructor training involves: (1) An initial 40 hour in-person or online videoconference training where the unique elements of MB-BP are introduced. (2) Two half-day in-person training retreats where MB-BP-specific teaching modules are practiced in peer groups, supervised by the senior MB-BP trainer, with peer and trainer feedback. (3) Studying specific evidence-based articles on hypertension etiology, treatment and prevention, as well as articles synthesizing evidence of mindfulness on hypertension and hypertension risk factors. [10,183,191-193] A written exam evaluates knowledge in this area, for which instructors-in-training need to pass. (4) Supervised teaching of MB-BP in non-study participants is done using the Mindfulness-Based Intervention Teacher Assessment Criteria (MBI-TAC), [1,2,194] and an annotated MB-BP Curriculum Guide until adequate quality is established within predefined criteria.

The unique areas of MB-BP are education on hypertension risk factors, hypertension health effects, and specific mindfulness modules focused on awareness of diet, physical activity, medication adherence, alcohol consumption, stress, and social support for behavior change. A Curriculum Guide has been created based on the standardized MBSR manual developed at UMass Medical School (**Appendix B**).[163,164] MB-BP sessions contain instruction and practice in mindfulness meditation, and conversations about stress and coping. Students learn a range of mindfulness skills including body scan exercises, meditation and yoga. Homework consists of

practicing skills for ≥45 min/day, 6 days/week. All MB-BP classes are to be held in either University or community-based locations in a comfortable, accessible environment where privacy is able to be maintained.

MB-BP builds a foundation of mindfulness skills (e.g. meditation, self-awareness, etc.) through the MBSR curriculum. MB-BP then directs attention towards hypertension risk factors. Early in the MB-BP, the importance of hypertension for health and mortality is described, along with hypertension risk factors. Participants will have their blood pressure and hypertension risk factors assessed at baseline, and be provided with this information during the first in-person MB-BP session. This phase aims to engage participants' interest in hypertension risk factors, and increase motivation for behavior change. MB-BP encourages participants to explore personal readiness for change in the different hypertension risk factors, and explore utilizing mindfulness practices to engage with those risk factors that they choose to. Each week, focus is provided on different hypertension risk factors. However, common themes exist across all hypertension risk factors including (1) awareness of thoughts, emotions and physical sensations particularly surrounding hypertension risk factors such as overconsumption of palatable foods, sedentary activities, alcohol consumption, medication adherence; (2) craving, particularly for hypertension risk factors such as overconsumption of palatable foods, sedentary activities, and alcohol consumption; (3) the impact of bringing mindfulness to every moment, particularly in relation to hypertension risk factors. For example, when consuming highly palatable food, bringing awareness to the emotions, thoughts and physical sensations prior to eating, during eating, and in the time afterwards. Participants are trained to bring non-judgmental attention to the often short-term pleasure of overconsumption of foods, sedentary activities, heavy alcohol consumption, or not taking medications, and bring non-judgmental attention to the longer term suffering associations with these activities. Through this process, participants are encouraged to reflect on if behavioral choices provide more benefit or harm to their well-being, and to choose the behaviors that bring benefit. (4) Self-care: as awareness of thoughts, emotions and physical sensations increases, and self-regulation will likely increases as a result of the meditation practices, the curriculum will emphasize to participants that it is common for people to start caring for themselves more. It is a way of better knowing ourselves, and through knowing ourselves in in each moment, we often want to care for ourselves in each moment. This may mean taking medication that will help our health, or being more physical active, eating more healthily, or consuming more moderate amounts of alcohol.

5.2 Handling of Study Interventions

Please see Appendices A and B for the MBSR and MB-BP curriculum guides.

5.3 Concomitant Interventions

Please see inclusion/exclusion criteria in Sections 4.1 and 4.2.

5.4 Adherence Assessment

Adherence to the prescribed MB-BP practices will be monitored through class attendance, practice logs and weekly health goals. Adherence data will be collected weekly during the course of the intervention. Dr. Schuman-Olivier is testing more

technologically enhanced forms of meditation logs (e.g. accelerometer-based ecological momentary assessment and actigraphy score; NCT01314378), and found large proportions of participants had difficulty using the device properly (e.g. failing to stop meditation timers), which hampered data quality (*paper in progress*). We discussed this issue with leading meditation researchers, and meditation logs remain their recommended method at this time. However, innovative approaches are being explored by Dr. Lazar, including smartphone apps linked to audio homework files, with timers linked to data exports. These technologies are not yet ready for use, but we will incorporate technological advances for homework monitoring once demonstrated to be effective. Data analyses will evaluate effect modification by adherence to the MB-BP practices.

6. STUDY PROCEDURES

6.1 Schedule of Evaluations

Variables Measured	Assessment Times			
	Baseline	10 weeks	6 months	1 year*
Demographics	X			
Family history of hypertension	X			
Childhood Socioeconomic Status	X			
Adverse Childhood Experiences	X			
Depressive Symptomatology	X	X	X	
Anxiety	X	X	X	
Medication Use	X	X	X	X
Anti-Hypertensive Medication Adherence	X	X	X	
Blood pressure	X	X	X	X
Anthropometry	X	X	X	X
Physical Activity	X	X	X	
Diet	X	X	X	X
Alcohol consumption	X	X	X	X
Cigarette Smoking	X	X	X	
Sleep Duration	X	X	X	X
Mindfulness	X	X	X	
Mindfulness home practice	X	X	X	X
Emotional Eating	X	X	X	
Self-Compassion	X	X	X	
Perceived Stress	X	X	X	
Emotional Regulation	X	X	X	X
Interoception	X	X	X	X
Heartbeat Detection	X	X	X	
Decentering	X	X	X	
Attention Control	X	X	X	
Craving for Hypertensive Risk Factors	X	X	X	
Social Integration	X	X	X	
Loneliness	X	X	X	
PROMIS Global Health	X	X	X	
Self-Control	X	X	X	
Resilience	X	X	X	
Self-efficacy for managing chronic disease	X	X	X	
Stress Reactivity	X	X	X	
Delay Discounting	X	X	X	
Semi-structured exit interviews at 6 months			X	

**1-year assessments consist of a subset of the other two follow ups and will be completed only with intervention group as controls are offered course post 6-month follow-up*

6.2 Description of Evaluations

6.2.1 Screening Evaluation and Consenting Procedure

Please see **Appendices C and D** for the consenting process prior to the phone screening, and prior to the in-person screening.

Final screening evaluations will occur at least one week prior to baseline assessments. Baseline assessments will occur within 4 weeks of intervention initiation.

Phone-Based Screening: For people who indicate interest in the study, this screening will take place by phone using trained research assistants to assess the exclusion criteria described above, with the exception of blood pressure which will be assessed in-person

In-Person Screening: If participants remain eligible after the phone-based screening, they will attend an in-person screening for blood pressure and medication assessment. If mean blood pressure is elevated (≥120 mmHg systolic and/or ≥80 mmHg diastolic pressure), participants will be invited to return for a second blood pressure reading. At that time, if the mean blood pressure across both assessment times is ≥120 mmHg systolic or ≥80 mmHg diastolic pressure, they will be invited to participate in the study.

6.2.2 Enrollment

The enrollment date is the day that the individual has met all the screening criteria, signs the informed consent form, and confirms agreement to participate in the study.

6.2.3 Baseline Assessments

(1) *Demographics:* age, race/ethnicity, socioeconomic status (education, employment), and household structure.
(2) *Family History of Hypertension (FH):* Assesses biological parents' history of having hypertension, based on questions from New England Family Study LEAP Project.
(3) *Childhood socioecononomic status:* retrospective reporting of parents' education, based on standardized questionnaires used in the Atherosclerosis Risk in Communities (ARIC) study.
(4) *Adverse Childhood Experiences:* Measured using the standardized Childhood Trauma Questionnaire (CTQ), Childhood food insecurity questionnaire, and the Childhood Experiences of Care and Abuse Inventory neglect subscale. [184,195-200]
(5) *Depressive symptomatology:* Assessed using Center for Epidemiologic Studies Depression Scale Revised (CESD-R). The CESD survey has been used extensively in the epidemiologic literature to assess depressive symptomatology.[201] The scale was updated to the CESD-R by Van Dam *et al*., which allows diagnosable criteria similar to Diagnostic and Statistical Manual (DSM) of Mental Disorders.[202]
(6) *Anxiety:* Assessed using the validated Beck Anxiety Inventory.[203-209]
(7) *Medication use:* Assessed directly from participants' medication bottles and self-report using standardized forms, including medication name, dose, frequency of use, and reason of use.
(8) *Antihypertensive medication adherence:* measured continuously using electronic medication bottle caps (eCAPS, Ottawa, Canada).[185]

(9) *Systolic and diastolic blood pressure:* Clinical blood pressure will be measured using a calibrated Omron HEM-705CPN following American Heart Association and Joint National Committee (JNC) guidelines.[183,210,211] Additionally, participants will be asked to complete three at home blood pressure readings using a validated home blood pressure monitor (Omron, Model PB786N) provided at baseline.

(10) *Anthropometry:* height and weight directly assessed using standard epidemiologic methods.[186]

(11) *Physical activity:* The International Physical Activity Questionnaire which has undergone substantial validity and reliability testing.[187-189] Adherence to Joint National Commission-7 (JNC-7) guidelines is 30 min aerobic physical activity ≥4 days per week.[183]

(12) *Diet*: assessed utilizing the validated Food Frequency that allows for calculation of hypertension-related dietary factors, including salt intake, alcohol consumption, total caloric consumption, fruit and vegetable consumption, and Dietary Approaches to Stop Hypertension (DASH) eating pattern score.[182]

(13) *Alcohol consumption:* additional self-report standardized questions assessing current alcohol consumption taken from the Behavioral Risk Factor Surveillance Survey (BRFSS).[212]

(14) *Cigarette smoking:* current smoking assessed using self-report standardized questions from the New England Family Study.

(15) *Sleep duration:* Sleep duration is assessed using a single question on sleep duration from the validated Pittsburgh Sleep Quality Index (PSQI).[213-215]

(16) *Mindfulness:* Assessed using the validated Five Facet Mindfulness Questionnaire.[216]

(17) *Mindfulness Home Practice:* questions capturing individuals at home mindfulness practice pre- and post- intervention are administered at each of the time points.

(18) *Emotional eating:* measured using the Three Factor Eating Questionnaire Revised 21-item (TFEQ-R21).[217,218]

(19) *Self-compassion:* Assessed using the validated Self-Compassion Scale Short Form (SCS-SF).[121]

(20) *Perceived stress:* Assessed using the validated 14-item Perceived Stress Scale.[219,220]

(21) *Emotion regulation:* Measured using the validated Difficulties in Emotion Regulation Scale.[221]

(22) *Interoception:* Assessed directly using the Heartbeat Detection Task and in secondary self-report using the validated Multidimensional Assessment of Interoceptive Awareness (MAIA).[222-225]

(23) *Decentering:* Assessed using the validated Experiences Questionnaire.[226,227]

(24) *Attention control:* Assessed using the Sustained Attention to Response Task (SART). The SART is a validated computerized test of sustained attention, response inhibition (executive function) and self-regulation.[228-230 231-233]

(25) *Craving:* craving for hypertension risk factors, including palatable foods, alcohol, and sedentary activities will be assessed using the validated Craving Experiences Questionnaire. [234]

(26) *Social integration:* Measured using the validated 12-item Interpersonal Support Evaluation List (ISEL-12) measure of social support.[235]

(27) *Loneliness:* Assessed using the validated R-UCLA Loneliness Scale.[236]

(28) *Global Health:* individual physical, mental and social health are measured using the validated NIH PROMIS Global Health v1.2 scale.[237]

(29) Self-control: assessed using the validated Self-Control Scale short form.[238,239]

(30) Resilience: measured using the validated 10-item Connor-Davidson Resilience Scale (CD-RISC-10).[240]

(31) Self-efficacy for chronic disease management: measured using the 6-item Self-efficacy for Managing Chronic Disease scale (SECD-6).[241]

(32) Stress Reactivity: Assessed using the Pittsburg Stress Battery a standardized protocol of 3 computerized tasks designed to induce a stress response indicated by evaluated cardiovascular (CV) reactivity.[242]

(33) Delayed Discounting: Assessed using the validated 5-Trial Adjusting Delay Discounting Task. [243]

(34) Functional Magnetic Resonance Imaging (fMRI): Participants in the UH3 phase who elect to take part in the fMRI imaging study will undergo an fMRI scanning session for approximately 40 minutes at baseline and 10-week follow-up. Scans will be acquired with a 3T scanner while the subject is in the resting state. Final sample size will be 24 per group (n=48); enrolling up to 60 participants in total to reach target sample size. Participants will undergo a separate informed consent and screening process for the fMRI imaging, so that they can be in the MB-BP study without imaging if they prefer. Refer to the separate fMRI study protocol for details.

6.2.4 Randomization and Intervention Allocation

Randomization will occur following the completion of baseline assessments prior to the initiation of study intervention. Only individuals who complete the baseline assessments and indicate they are available for the upcoming courses will be included in the randomization process.

Stratified randomization will be used, as simple randomization can fail if it creates groups unbalanced for critical features known to affect outcomes.[7,8] Stratified randomization can reduce both types I and II error, improve trial efficiency, and facilitate subgroup and interim analyses.[7] Variables used to create strata include age (≤60 *vs.* >60 years), gender (male *vs.* female), and/or uncontrolled hypertension (≥140 mmHg systolic pressure, or ≥90 mmHg diastolic pressure) *vs*. prehypertension (120 to <140 mmHg systolic pressure, and 80 to <90 mmHg diastolic pressure). Simple random sampling will occur within each of the eight strata, thus allowing for each arm of the study to be more balanced with respect to age, gender, and hypertension category. The total size of each stratum will vary from cohort to cohort. To conduct the randomization, a list of participant IDs and strata characteristics will be provided, and a trained researcher not affiliated with the study will perform the randomization on participants after baseline assessment and final determination of eligibility is complete. Randomization will be done using an online computer software program known as Research Randomizer (Version 4.0).[9] The randomization process will occur after each new round of enrollment (i.e., unique cohort). If the cohort sample size is such that there are not enough participants for balance within the three strata, we will prioritize stratification by two key strata instead of three, specifically by baseline blood pressure status and gender. Furthermore, after randomization within strata, if sample sizes in the entire sample for cohort differ by more than one participant per group, then all imbalanced groups are re-randomized until the entire sample differs by no more than 1 participant per group (e.g. n=8 MB-BP, n=9 enhanced usual care control). Expected cohort sizes, consistent with the

SARP are 15-20 participants. We anticipate needing to run 6 to 7 cohorts to reach the target sample size.

Study enrollment will continue until target enrollment goals are reached (i.e., n=50 for MBBP intervention group and n=50 for enhanced usual care control group).

6.2.5 Blinding

All study staff will be blinded to the participant treatment allocation with the exception of the instructor, individual who performs the randomization, and staff member coordinating participants within each course. All staff performing participant assessments will be blinded to the participant treatment allocation to promote equipoise. Data analyses will be performed by a statistician blinded to treatment allocation type. The data manager will be able to break blinding if needed (e.g. for Data and Safety Monitoring Board). Circumstances for breaking the blind would be a large number of adverse experiences (>10% of enrolled participants reporting AEs rated as severe or life threatening) taking place in one or more study group. In this case, the data safety monitoring board would be notified, and could break the blind to help determine the cause of the adverse experiences.

6.2.6 Follow-up Visits

Follow-up assessments will be scheduled and conducted within the pre-defined assessment windows outlined previously in section 3, study design. Questionnaires and assessments administered at 10 weeks and 6-months follow-up are identical to those administered at the first in-person screening assessment and at baseline, with the exception that questionnaires for which the answers should not change or be informative (age, race/ethnicity, education, adverse childhood experiences, family history of hypertension) are not given at follow-ups. In addition, adverse events are monitored and documented at each of the follow-up periods as well as throughout the duration of an individual's study involvement according to the data safety monitoring protocol put forth in this grant. Participants are also asked a set of semi-structured questions at the end of their six month follow up that inquire about their experience as either a control group member or intervention group participant.

6.2.7 Completion/Final Evaluation

For control group participants, the 6-month evaluation is the final visit. Upon completion of this assessment, they will be eligible to participate in the MB-BP training. Individuals in the intervention group will be assessed at 1 year follow up with a subset of measures (see Table 6.1 Schedule of Evaluation) in order to assess long term effects of the intervention.

7. SAFETY ASSESSMENTS

7.1 Expected Risks and Specification of Safety Parameters

Meditation-related risks: NCCIH states that meditation is generally safe for healthy people, but that adverse effects have also been reported. [5] Undesirable side effects and risks of meditation have been documented in more than 40 scientific reports [for reviews see [6-8]] and are listed in the Mindfulness-Based Intervention Guidelines.[9,10] More common, less serious side effects that

have been reported by individuals within the context of MBIs or of individuals who are meditating less than an hour per day include: increased depression, anxiety or panic, re-experiencing of traumatic memories, dissociation, executive dysfunction, headaches/body pain and insomnia.[6,11-16] A few case reports of more serious side effects including mania, psychosis, and suicidality have been reported, mostly in the contexts of intensive retreats (>5 hrs/day) or in conjunction with pre-existing psychopathology.[6,8,9,17] The frequency of serious adverse effects in the context of MBIs is estimated to be less than 1%, although adequate estimates are not available.[18]

A number of actions have been taken to minimize meditation-related risks at different stages of the study. During the pre-enrollment stage, individuals with severe mental illness are excluded from the study and all risks are clearly communicated in the consent form. During treatment, meditations are relatively short and interspersed with dyads and reflections. Mindfulness homework assigned as part of the intervention is optional and is recommended to not exceed 1 hour per day. Teachers query participants about their experiences with meditation, and provide corrective feedback or modifications when needed. Developing strategies for working with physical and emotional discomfort is an explicit goal of the program. Because not all participants feel comfortable disclosing difficulties in class, an online "safety check-in" questionnaire will query meditation-related risks (see section 6.2.1). Dr. Ellen Flynn, a licensed psychiatrist, will be available to advise on any psychological events that occur, and provide referrals for treatment if needed. Additionally, Dr. Willoughby Britton will provide expert consult on safety monitoring and reporting, including providing DSMP specific training to research staff and investigators.

Psychological distress: Research subjects participating in this study may have feelings of loss of privacy from being contacted about participating in the study, and possible psychological distress caused by questions asked during the in-person and online questionnaires that bring up painful memories or feelings. However, the resulting potential for injury to research subjects is judged to be minimal. We have already contacted and clinically evaluated thousands of participants from other studies such as the New England Family and Women's Health Initiative using similar assessment procedures to this study, with good responses from the participants. With regard to psychological distress from taking part in the MB-BP intervention, given that screening questions will exclude participants with substantial mental illness, and given the NCCIH statement above that "Meditation is considered to be safe for healthy people."[244] we expect that risk of psychological distress will be low. The risk of increased psychological distress from meditation will be clearly outlined in the consent form and participants will be encouraged to consult with both the course instructor and study staff in the case of any increased distress. Dr. Ellen Flynn, a licensed psychiatrist, will be available to advise on any psychological events that occur, and provide referrals for treatment if needed.

Loss of confidentiality: Likelihood: rare. Minimization: Confidentiality will be maintained by using deidentifying data sets. All paper forms and data collection tools, including the informed consent forms, will be kept in a locked filing cabinet in a secure location. All electronic data files containing identifying information will be encrypted with a cloud-based software. Note that although these measures have been taken to protect participants' personal information, complete confidentiality cannot be guaranteed when transmitting information over the internet. All information obtained from participants will be accessible only to research staff.

Injury due to physical activities: It is possible that injuries could be sustained from (1) the gentle mindful movements (yoga), or (2) physical activities that participants engage in as a result of the intervention encouraging exploration of physical activity as a way to reduce blood pressure. (1) Mindful movements: Participants receive a handout during the orientation showing the yoga

poses that will be offered during the course. They are encouraged to explore limits in their body related to movement, but not to go beyond those limits Participants are asked to listen to what their body is telling them more closely than what the mindful movement instructor is telling them. Modifications of poses are available, including for those limited to chairs or wheelchairs. Participants are encouraged to bring the handout of poses to their health care providers if they have any physical limitations, so that the providers can advise on which poses to do, and which to avoid. (2) Physical activities: Participants are encouraged to explore physical activities that promote strength and conditioning as a way to reduce blood pressure. As with the mindful movements, they are encouraged to explore limits in their body related to movement, but not to go beyond those limits. Participants are asked to listen to what their body is telling them more closely than what the mindful movement instructor is telling them. Furthermore, they are encouraged to ask their healthcare provider about advised physical activities if they have any physical limitations.

Risks associated with fMRI: The fMRI study will be conducted using a 3T MR scanner at UMass Medical School, which has been approved for research and clinical studies in children and adults by the FDA. Magnetic resonance (MR) technology does not use X-rays, but instead uses strong magnetic fields and radio waves. Individuals interested in participating in the fMRI study will complete a screening questionnaire to assess eligibility, including asking whether they have devices that can be affected by MRI or conditions (e.g., claustrophobia, body mass greater than 300 lbs.) that prohibit the ability to be scanned. Participants are screened immediately prior to each MRI scan to ensure participant safety. Significant risks also can arise if ferromagnetic materials are brought into the high magnetic field environment of the scanner and immediate vicinity, as they can become hazardous projectiles. These types of items are not permitted in the scanning area. The MR exams are painless, and except for the pulsating sounds, subjects will not be aware that MR scanning is taking place. With proper safety precautions in terms of the avoidance of metal objects, there are no known health risks associated with MRI. The safety of MRI is reflected in the fact that it is used in standard medical practice without the requirement for informed patient consent. Most people experience no ill effects from the magnetic field, but some report claustrophobia, dizziness, mild nausea, headaches, a metallic taste in their mouth, double vision, or a sensation of flashing lights. These symptoms are transient and resolve quickly after the subject exits the scanner. The technologist will be able to hear subjects at all times and subjects are free to end the procedure at any time. In rare cases, a very slight, uncomfortable tingling of the back due to the rapid switching of the magnetic field has been reported during certain types of scans. Subjects are asked to report this immediately so the scan can be changed to avoid this. Although these precautions will avoid all known risks associated with MR, this procedure may involve risks that are currently unknown. The scanner is noisy, but does not harm hearing. For comfort, subjects will be given earplugs to muffle the noise.

Risk of adverse events during the study: It is possible that some patients will have an adverse event during the study, including increased stress or anxiety. Participants with major mental health conditions, such as schizophrenia, history of psychosis, bipolar depression, suicidal ideation, borderline personality disorder, post-traumatic stress disorder, obsessive compulsive disorder, panic attacks, current alcohol or substance abuse, or an eating disorder are ineligible for the study. We expect risk of adverse events to be very low. For further discussion of AE and SAE monitoring and reporting refer to Section 6.2 below.

Impact statement: These risks are considered to be minimal and are addressed in the protocol and consent form.

7.2 Methods and Timing for Assessing, Recording, and Analyzing Safety Parameters

Safety monitoring will occur continuously throughout the study using both active and passive monitoring methods outlined and discussed in further detail below. All reported AEs, SAEs, and unanticipated problems will be recorded throughout the study using the data collection systems set up and detailed in the Data and Safety Monitoring Plan (DSMP)

The research staff will record all reportable events with start dates occurring any time after informed consent is obtained but no later than the final 1-year assessment. At each study visit, the research staff will inquire about the occurrence of AE/SAEs since the last visit (or time of most recent reporting). Events will be followed for outcome information until resolution or stabilization or until the grant funding ends.

7.3 Adverse Events and Serious Adverse Events, Reporting Procedures, and Follow-up

An adverse event (AE) is generally defined as any unfavorable and unintended diagnosis, symptom, sign (including an abnormal laboratory finding), syndrome or disease which either occurs during the study, having been absent at baseline, or if present at baseline, appears to worsen. A serious adverse event (SAE) is generally defined as any untoward medical occurrence that results in death, is life threatening, requires inpatient hospitalization or prolongation of existing hospitalization, results in persistent or significant disability/incapacity, or is a congenital anomaly.

Per NCCIH safety monitoring requirements, all AEs and SAEs captured and/or observed involving enrolled study participants will be recorded regardless of their relationship to the study intervention. Below we outline possible AEs and SAEs that may occur related to this research study and intervention; present our procedures for capturing and recoding; and detail the protocol for follow up of AEs. For further discussion of the procedures related to safety monitoring and follow up procedures refer to Section 7.4. Safety Monitoring.

Safety Check-ins: All participants enrolled in the study, regardless of treatment allocation, will receive a two-tiered safety monitoring 'check-in' every 2 weeks during the treatment phase of the study; every month during months 3-6 of the follow-up phase; and at the final 1-year time point. The two-tiered system is designed to detect and follow up on AEs that are at least moderate in severity (interfere with ADL), and to minimize staff and participant burden that would otherwise occur if all mild events were queried and documented.

Tier 1 of the safety check-in involves sending all active, enrolled participants an email (or placing a phone call from a research staff member, if no email provided) containing a link to a brief online survey that queries events with moderate or greater levels of severity. Any participant who endorses one or more tier 1 questions will automatically receive a tier 2 survey and follow-up phone call from study staff.

The Tier 2 survey specifically queries the most common meditation-related side effects (e.g. anxiety, depression, dissociation, flashbacks etc.) using patient-reported outcomes measurement information system (PROMIS) or NeuroQol items (or other validated scales if construct is not available), and PROMIS response options (never- very often).

Additionally, the tier 2 survey will ask participants to provide further detail (i.e., date of onset, symptomology, circumstance surrounding the event, relatedness to the intervention, etc.) regarding the AE/SAE reported in the tier 1 survey. Detail provided will be used to guide the tier 2 phone calls made by research study staff.

Tier 2 Safety Check-in phone call: Any participant who indicates that he or she experienced an AE or SAE will then receive a follow up call from a trained research staff member. The purpose of the follow up call will be to further document the details of the AE/SAE, assess need for treatment modification, referrals and reporting.

Research staff members conducting the safety monitoring phone interviews will document the details of the AE/SAE using the Adverse Events Form, which will then be included in the participant file as well as in the annual Data Safety Monitoring Reports presented to the DSMB. Reporting procedures for AEs and SAEs related to the study will be followed including reporting all SAEs to the study PI and DSMB committee chair.

7.3.1 Mental Health

Participants assigned to any treatment group may experience mental health or suicidal ideation during the course of their study involvement. All study participants will be monitored for AEs and SAEs by study staff on a monthly basis until the time of their study completion. Additionally, participants will be asked to complete questionnaires about anxiety, depression and suicidal ideation, specifically the Beck Anxiety Inventory and the Center for Epidemiology Study Depression Scale Revised (CESD-R), at each of their in-person assessments, excluding the one year follow up. Dr. Flynn, a licensed psychiatrist with extensive experience evaluating research participants for clinical deterioration or suicidality, will serve as the study clinician.

Beck Anxiety Inventory (BA): If participant scores ≥26 on the Beck Anxiety Inventory, a safety flag will appear notifying the research assistant (RA) administering the assessment. The RA will then implement the MB-BP safety protocol, which is reviewed and approved by the Brown University IRB. Staff are trained on the safety protocol and a hard copy of the protocol is kept in an accessible location in the assessment office at all times.

Depressive Symptomatology: The CESD-R will be administered during the in-person assessment visits, and scores will be reviewed immediately upon completion of the in-person assessments.

1. Sadness (dysphoria): Question numbers 2,4, 6
2. Loss of Interest (anhedonia): Question numbers 8, 10
3. Appetite: Question numbers 1, 18
4. Sleep: Question numbers 5, 11, 19
5. Thinking / concentration: Question numbers 3, 20
6. Guilt (worthlessness): Question numbers 9, 17
7. Tired (fatigue): Question numbers 7, 16
8. Movement (agitation): Question numbers 12, 13
9. Suicidal ideation: Question numbers 14, 15

Participants are considered to meet criteria for major depressive episode if they have anhedonia or dysphoria nearly every day for the past two weeks, plus symptoms in an additional 4 DSM symptom groups noted as occurring nearly every day for the past two weeks. If participants meet criteria for major depressive episode, a safety flag will appear notifying the research

assistant (RA) administering the assessment. The RA will then implement the MB-BP safety protocol, which is reviewed and approved by the Brown University IRB. Staff are trained on the safety protocol and a hard copy of the protocol is kept in an accessible location in the assessment office at all times.

If participants respond having any suicidal ideation (CES-D questions 14 or 15), staff will again be instructed to follow the IRB approved safety protocol.

7.3.2 Physical Health

Possible atrial fibrillation detected during Heartbeat Detection Task: If possible atrial fibrillation is indicated by the Kardia Mobile device during the "Heart Beat Detection Task" a safety flag will appear notifying the research assistant (RA) administering the assessment. The RA will then implement the MB-BP safety protocol.

Out-of-range blood pressure readings: If during an in-person assessment the participants systolic blood pressure (SBP) and/or diastolic blood pressure (DBP) falls outside of the acceptable range outlined in the safety protocol, the RA will be notified and the IRB approved safety protocol will be implemented.

Injury due to physical activities: It is possible that injuries could be sustained from (1) the gentle mindful movements (yoga), or (2) physical activities that participants engage in as a result of the intervention encouraging exploration of physical activity as a way to reduce blood pressure.

(1) Mindful movements: Participants receive a handout during the orientation showing the yoga poses that will be offered during the course. They are encouraged to explore limits in their body related to movement, but not to go beyond those limits Participants are asked to listen to what their body is telling them more closely than what the mindful movement instructor is telling them. Modifications of poses are available, including for those limited to chairs or wheelchairs. Participants are encouraged to bring the handout of poses to their health care providers if they have any physical limitations, so that the providers can advise on which poses to do, and which to avoid.

(2) Physical activities: Participants are encouraged to explore physical activities that promote strength and conditioning as a way to reduce blood pressure. As with the mindful movements, they are encouraged to explore limits in their body related to movement, but not to go beyond those limits. Participants are asked to listen to what their body is telling them more closely than what the mindful movement instructor is telling them. Furthermore, they are encouraged to ask their healthcare provider about advised physical activities if they have any physical limitations.

Note that adverse events related to physical injuries will be captured during the routine safety check-ins (discussed above).

7.3.3 Other

Participant Initiated (Passive monitoring): Participants are encouraged to contact meditation instructors and/or study staff if any physical or mental health symptoms arise or other study or meditation-related problems occur. Participants may report AEs at any time throughout the study. Events will be evaluated with the Adverse Events Form by study staff.

Attrition: Reasons for attrition are also an important source of AEs, but are rarely assessed adequately, as participants are unlikely to give honest answers if queried directly by study staff. To increase the accuracy of attrition reason reporting, participants will be asked to complete a brief online Participant-initiated dropout reason survey (see Appendix A7).

Investigator-initiated withdrawals: In rare circumstances, a study participant may be withdrawn from the study and/or intervention by the researcher. In this case, the researcher or other study staff should complete the Attrition information form, and describe reasons for attrition. Note, however, that this scenario would be very unusual, and has not happened once in the in the approximately 130 participants who have gone through the MP-BP study to date. Potential reasons for investigator-initiated withdrawal would be MB-BP classroom or assessment disruption in ways that are causing harm or an unsafe environment for other classroom participants, the instructor, or study staff. The screening questionnaire excludes participants with mental health criteria that puts them at higher risk for disruption. We expect this scenario to occur extremely rarely (as evidenced to date), but remains a possibility.

fMRI Study Safety Monitoring: it is possible participants may experience or report an AE or SAE during their involvement with the fMRI Study. Research staff will use the Adverse Events Form and accompanying documents found in the Appendices to document all AE/SAE discovered at the time of involvement in the fMRI imaging study. The logged events will then be communicated to the Coordinating Center, so that they can be included in the participant file as well as in the annual Data and Safety Monitoring Reports presented to the DSMB. Reporting procedures for AEs and SAEs related to the study will be followed including reporting all SAEs to the study PI and DSMB committee chair.

Non-Response to Treatment: The possibility that the treatment will not yield benefit is another possible risk and will be explained during informed consent procedures. Non-responders (identified as minimal change in medical regimen adherence from baseline assessment) will be provided with referrals to other treatment, if desired.

The data collection systems we have set up to monitor and record AEs and SAEs are specifically designed to avoid double capture. Unique participant identifiers, dates and details surrounding events, as well as steps taken to follow up are recorded.

7.4 Safety Monitoring

Oversight of internal monitoring of the participants' safety will be conducted by the local PI, Dr. Eric Loucks. Oversight of the external Data and Safety Monitoring Committee will be conducted by the chair (Dr. Edmondson). The Data and Safety Monitoring Committee will include experts in cardiology (Choudhary), psychology/psychiatry (Edmondson), epidemiology (Choudhary), and biostatistics (Liu).

Entities Conducting Monitoring: The Institutional Review Board (IRBs) at Brown University will review all research procedures, and will provide oversight. Internal monitoring will be done by the Brown University principal investigator (Dr. Loucks) and the Brown University IRB. The Data Safety Monitoring Committee will provide external monitoring, and will meet annually by phone, video conference, or in-person. They will be provided data annually in order to evaluate potential effects of the RCT on major outcomes (e.g. medical regimen adherence). Any serious adverse effects will be immediately reported to the principal investigator (Loucks) and the committee chair.

What is Monitored: Monitoring is done of all procedures to ensure that they conform to the approved protocol; of unforeseen circumstances that might arise and affect safety; of all reports of serious adverse events as defined in US Department of Health and Human Services regulations for the protection of human research subjects 45 CFR Part 46, and the FDA 312.32 (death, life-threatening experience, new or prolonged hospitalization, persistent or significant disability/incapacity); of other significant adverse events (adverse events that lead to drop out by participant or termination by the investigator); of unexpected adverse events resulting from the study; and of expected adverse events.

Monitoring is done of all study inclusion and exclusion criteria. During this clinical trial, we will notify officials, as mandated by law, if a participant reports intention to harm him/herself or others, or reports child abuse or abuse of an elder. Dr. Ellen Flynn, a licensed psychiatrist, will be available to advise on any psychological events that occur, and provide referrals for treatment if needed.

Frequency of Monitoring: All adverse events will be continuously monitored by the PI as they are documented by the study staff in accordance with the protocol (Please see section 7.3). Participants will be given contact information so that they can inform us of events that occur in between study visits. The PI will meet with staff weekly as schedules allow to review participant progress and to check in about the experiences with the experimental procedures, including adverse events. Any adverse events that are observed and/or reported will be reported to Dr. Loucks and the Data Safety Monitoring Committee chair under the proposed timelines in the DSMP. The Investigators and DSMB members will be available to meet outside of the regularly scheduled meetings (scheduled annually), if necessary, due to concerns regarding a particular participant or any problems that may arise for participants. If necessary, they will make appropriate recommendations for changes in protocol, or terminate the study. The Brown University IRB conducts the monitoring at the continuing reviews as scheduled, whenever modification requests are considered, and upon receiving reports of serious adverse events from the PI or anyone else.

Reporting Plan: Any serious adverse events that are observed and/or reported will be immediately reported to Dr. Loucks and the Data Safety Monitoring Committee Chair. Serious adverse events related to the study are then reported to the Brown University IRB and to NIH. Brown University's IRB requires fatalities related to the study be reported within 24 hours. All serious adverse events related to this study will be reported to the Brown University IRB immediately by telephone and by written report within 48 hours of our receipt of information regarding the event. All other adverse events related to the study will be reported at the continuing review. Serious adverse events related to the study will also be reported in writing to the NIH Project Officer within 48 hours of the PI becoming aware. All serious adverse events related to the study will be reported annually in the Progress Report sent to the NIH Project Officer. Data on all AEs and SAEs will be recorded.

Any actions taken by the IRB, other than acceptance of the adverse event report, will be reported to the NIH along with any changes or amendments to the protocol requested by the IRB in response to these reports. Proposed changes or amendments to the protocol in general must be approved in writing by both the Brown University IRB as well as by the funding agency, NCCIH.

8. INTERVENTION DISCONTINUATION

This study will be stopped prior to its completion if: (1) the intervention is associated with adverse effects that call into question the safety of the intervention; (2) difficulty in study recruitment or retention will significantly impact the ability to evaluate the study endpoints; (3) any new information becomes available during the trial that necessitates stopping the trial.

9. STATISTICAL CONSIDERATIONS

9.1 General Design

Analyses will evaluate (1) whether MB-BP influences self-regulation targets, (2) whether the MB-BP-induced changes in self-regulation targets are associated with changes in medical regimen adherence, and (3) whether MB-BP is associated with medical regimen adherence. Analyses will incorporate generalized linear models (GLM) with properly chosen link functions, performed using generalized estimating equations (GEE) with robust standard error estimators.[245,246] This provides an extension of regression analysis to the case of correlated or repeated observations, allows for inclusion of both continuous and discrete dependent variables, and enables modeling of covariance structures when observations are correlated across time. Following "intention-to-treat" principles, analyses will be conducted on all participants in the randomized controlled trial, regardless of intervention completion. Analyses will use GLM with identity links for normally distributed data. The between-groups independent variable in the GEE analysis is intervention condition, with control conditions as distinct referent groups. Categorical variables will be included in GLM/GEE models as indicator variables with one level chosen as referent. Continuous independent variables such as age and baseline blood pressure would be included as linear terms. In addition, we will include assessment time as a linear term and possibly with an additional quadratic term to capture possible non-linear relationship of time with the outcomes. It is conceivable that one would observe a steeper slope in the initial months that would decrease in the pursuing months. Thus, the use of a quadratic term for assessment time would be able to capture these phenomena. To evaluate self-regulation targets as mediators of the effects of MB-BP on medical regimen adherence, mediation analyses described by Valeri and VanderWeele will be employed.[247] Such analyses will allow for potential interactions between the exposure and mediator of interest and account for potential confounders of the exposure-mediator, mediator-outcome, and exposure-outcome relationships using standard regression techniques. These methods estimate total, indirect and direct effect sizes, as well as statistical variance.[247]

9.2 Sample Size and Randomization

Treatment Assignment Procedures

Statistical Power:

Utilizing effect sizes and statistical variance from the Stage 1 MB-BP clinical trial showed an increase of MAIA from baseline of mean 22.6 (SD=6.5) to mean 26.3 (SD=6.0) at 6-month follow-up, demonstrating a 3.7 increase in MAIA score ($p<0.001$). Power analyses using the T statistic and non-centrality parameter, with alpha (two-tailed) set at 0.05 and beta at 0.2, shows with a MAIA increase of 3.7 in MB-BP vs. control, sample size requirements are 46 per group. With DASH diet score increasing from 2.78 (SD=0.61) at baseline to 3.37 (SD=0.81) at 6-month follow-up in the Stage 1 MB-BP study in the 67% of participants with a low DASH score (<5.5), demonstrating a DASH diet score increase of 0.59 ($p<0.001$). Using an effect size of 0.5, power analyses using the T statistic and non-centrality parameter

suggest that, with alpha (two-tailed) set at 0.05 and beta at 0.2, a sample size of 33 per group will be sufficient.

Using simulations from Fritz and MacKinnon,[248] for 80% power to detect a mediated effect when effect of exposure on mediator, and mediator on outcome, is of small to medium strength (standardized Cohen's d effect sizes of 0.26-0.39 each), based on Sobel first-order test, we will need 90 participants. Given that 67% of Stage 1 participants had DASH diet score <5.5, recruiting a sample size of 160 participants across the UH2 and UH3 phases (Figure 1), this would allow for 107 participants with low DASH diet to be included in primary mediation analyses with DASH diet score as the outcome. Stage 1 effect sizes on DASH diet score were large Cohen's d (d=0.82). Effect sizes on the primary self-regulation outcome was medium (MAIA Cohen's d=0.59). Assuming associations between the MAIA and DASH diet score are in the small to medium range, the study should be adequately powered for mediation analyses. Please note that Stage 1 analyses also demonstrated significant improvements in other health behaviors, including alcohol consumption and physical activity following the MB-BP intervention. Secondary analyses will evaluate impacts of MB-BP on these health behaviors, but are not adequately powered for mediation analyses due to the lower proportion of participants that do not adhere to these behavioral AHA guidelines (i.e. approximately 15% of participants do not comply to AHA alcohol guidelines, and 32% do not adhere to physical activity guidelines in our Stage 1 sample).

The fMRI imaging analyses (n=24 per group) should be adequately powered, based on power calculations shown in the MINDFUL-PC study below and by power calculations outlined in the fMRI study protocol and MOP. To achieve this, we estimate having to enroll up to 60 eligible study participants into the fMRI study.

For fMRI self-regulation neural targets, resting State Functional Connectivity (rs-FC) will be expressed as correlation coefficients, transformed using Fisher's z-transformation for analysis. rs-FC will be obtained at two time points (baseline: T1; post-training: T2) for participants and controls. We will use a mixed effects model with baseline rs-FC as an additional predictor for the change outcome. There are two elements to the hypotheses – the determination of the significance of the change within group and the comparison of the change in the treatment group compared to the control group. For the comparison in change between treatment and control group, with a mixed analysis of variance (within-subjects rs-FC at T1 and T2, and between-subjects according to intervention; sample size, total n = 48, 24/group), we are able to detect an ES of 0.3 between the two treatment groups with 81.3% power (and an ES of .21 with 81.3% power for within-group mindfulness effect; an ES of 0.23 with 87.7% power for an interaction of treatment and time), with two-sided tests at alpha=0.05. Thus is reasonable power to detect small- to moderate-size differences in BOLD.[249]

Our calculations assume drop-out rates will average around 10-15% across each group, with the control group exhibiting the highest rates of withdrawal and loss to follow up. These estimates are based on the Stage 1 and 2a MB-BP trials in the UH2 phase.

Randomization procedure: Please see Section 3 (study design) above.

Blinding protocol: Please see Section 3 (study design) above.

9.3 Definition of Populations

Please see Section 9.1 for Intention-To-Treat analysis approach.

9.4 Interim Analyses and Stopping Rules

Please see Section 8 for stopping rules and related analyses.

9.5 Outcomes

9.5.1 Primary Outcome

Self-Regulation Primary Outcome: Multidimensional Assessment of Interoceptive Awareness (MAIA), a validated measure of body awareness.[222-224] We hypothesize that MB-BP will significantly improve the MAIA in directions of better self-regulation, compared to control.

9.5.2 Secondary Outcomes

Self-regulation outcomes foster triangulation, including further validated measures of interoceptive awareness (Heartbeat Detection Task,[250,251] Interoceptive Awareness fMRI Task[252-254]), stress and emotion regulation (Difficulties in Emotion Regulation Scale,[221] Pittsburgh Stress Battery,[255-259] Perceived Stress Scale,[219,220] Beck Anxiety Inventory,[203-209] CESD-R[201,202]), attention control (Sustained Attention to Response Task,[228-233]), self-compassion (Self-Compassion Scale Short Form[121]), and self-efficacy (Self control scale and SECD-6 Scale[260-267]).

Medical regimen adherence outcome: Diet, assessed utilizing Dietary Approaches to Stop Hypertension (DASH) eating pattern score,[181] measured via diet history food frequency questionnaire.[182] The measure assesses adherence to JNC-7 guidelines DASH eating pattern score (range 0-8).[181,183]

Medical regimen adherence secondary outcomes: *(1) Alcohol consumption:* Amount and frequency of alcohol consumption, will be assessed via self-report utilizing standard questions from the behavioral Risk Factor Surveillance Survey.[184] AHA/ACC hypertension clinical practice guideline cut-point of healthy alcohol intake is ≤ 2 drinks (e.g. 24 oz. beer, 10 oz. wine, or 3 oz. 80-proof whiskey) per day in men and ≤1 drink per day in women.[10] Medical regimen adherence will be defined as adherence to JNC-7-recommended behavioral and medication treatment of hypertension.[183] *(2) Electronically-Measured Antihypertensive Medication Adherence:* measured continuously using electronic medication bottle caps (eCAPS, Ottawa, Canada)[185] *(3) Body Mass Index:* height and weight directly assessed using standard epidemiologic methods, with change evaluated in participants considered overweight or obese (BMI≥25 kg/m^2).[186] *(4) Physical activity:* We will use the International Physical Activity Questionnaire which has undergone substantial validity and reliability testing.[187-189] Adherence to Joint National Commission-7 (JNC-7) guidelines is 30 min aerobic physical activity ≥4 days per week.[183]

9.6 Data Analyses

Please see Section 9.1 for analytic approach.

10. DATA COLLECTION AND QUALITY ASSURANCE

10.1 Data Collection Forms

Questionnaire data will typically be collected using Qualtrics, LLC (Provo, UT, USA) survey instruments, so that participants can complete questionnaires on their own time at home within the defined assessment windows, using their computers or smart phones. Exceptions to this include the phone screening questionnaire, and the baseline and follow-up questions on depression and anxiety (described in Section 6.2.2) to allow for a safety protocol to be followed if there are high levels of depression, anxiety or suicidal ideation. In the event that participants are uncomfortable or unable to use a computer for tests data will be collected using hard copies of surveys and entered by a trained member of the research staff.

Assessments of blood pressure, height, weight, and medication use are assessed in-person by trained research assistants blinded to treatment allocation.

Confidentiality of Patient Records: The clinical data will be de-identified but linked. Private information such as name, date of birth, and address for recontacting will be kept in a password protected, encrypted database on a different disk than the clinical data held by the Project Coordinator and used for the purposes of contacting participants. The principal investigator will only be given access to identifiable personal information for the purposes of patient safety or monitoring by the NIH, data safety monitoring boards or HIPPA compliance officer approved agents.

10.2 Data Management

Data management will be performed by downloading data at minimum every 2 weeks during active data collection periods, and assessing data for missingness and errors. Data will be maintained in password-protected Microsoft Excel Spreadsheets, and then exported using .csv functions for analysis in SAS software.

Please see Section 10.1 for data collection forms description.

10.3 Quality Assurance

10.3.1 Training

Describe types and mechanisms of training of staff for the study.

Please see **Appendices E and F** for manuals to perform staff training for assessments. Only trained, experienced, culturally competent interviewers will be hired to perform assessments.

10.3.2 Quality Control Committee

There is no formal quality control committee.

10.3.3 Metrics

Please see Appendices E and F for quality control metrics of blood pressure and anthropometry assessments.

MB-BP Competency and Treatment Fidelity: Treatment fidelity strategies will be performed in accordance with recommendations of the NIH Behavior Change consortium, specifically ensuring treatment fidelity in the following five areas: study design, training providers, delivery of treatment, receipt of treatment and enactment

of treatment skills,[268] as follows. *Study design*: All session durations will be recorded and any deviations from the planned duration will be documented. All class sessions will be taped (as conditions allow). Audiotapes will undergo a quality assessment audit by research technicians, who will review a ten percent randomly selected sample of the recordings. Research staff completing the audit will conduct competency ratings on these tapes using validated adherence scales (MBCT Adherence Scale, where items 1-11 in the scale are for MBSR, MB-BP and MBCT[269]). Data from the audit will be used to provide detailed feedback to treatment providers. We will ensure equivalent dose across conditions, including meditation, yoga and stress reduction training, through tracking the audio recordings. Possible setbacks in implementation of treatment will be addressed, including having a large pool of MBSR and MB-BP instructors in the event that specific instructors no longer teach classes. Instructor attrition will be tracked.

Provider training: MB-BP will be performed by qualified or certified MBSR instructors[191] with formal training in cardiovascular health (e.g. dietician, physician assistant, health and wellness coach, and those with an Associate's or Bachelor's degree in relevant health sciences), and further certification in MB-BP. MB-BP instructor training involves: (1) An initial 40 hour in-person or online videoconference training where the unique elements of MB-BP are introduced. (2) Two half-day in-person training retreats where MB-BP-specific teaching modules are practiced in peer groups, supervised by the senior MB-BP trainer, with peer and trainer feedback. (3) Studying specific evidence-based articles on hypertension etiology, treatment and prevention, as well as articles synthesizing evidence of mindfulness on hypertension and hypertension risk factors. [10,183,192-194] A written exam evaluates knowledge in this area, for which instructors-in-training need to pass. (4) Supervised teaching of MB-BP in non-study participants is done using the Mindfulness-Based Intervention Teacher Assessment Criteria (MBI-TAC), [1,2,195] and an annotated MB-BP Curriculum Guide until adequate quality is established within predefined criteria.

MBSR teacher certification is fairly extensive, and accreditation occurs through the University of Massachusetts Medical School Center for Mindfulness in Medicine, Health Care and Society, detailed elsewhere.[191] Examples of criteria for becoming a certified MBSR teaching include (i) completion of an eight-week MBSR course as a participant, (ii) completion of several multi-day residential training courses in mindfulness based stress reduction practice and teaching, (iii) substantial experience in teaching MBSR, (iv) strong references letters from colleagues and participants who have taken your MBSR courses, (v) completion of several multi-day mindfulness meditation retreats, (vi) have a graduate degree in a field connected to MBSR (e.g. education, psychology, medicine) or demonstration of equivalent understanding through work experience in a related field. There are 89 registered MBSR programs in Massachusetts (60), RI (9) and CT (20) that offer year-round program including the Center for Mindfulness where MBSR originated. Eligible programs must be 8 weeks and the instructors must have completed the MBSR Instructor certification training to participate in the study.

Delivery of treatment: We will assess participants' perceptions of provider warmth and credibility using brief measures based on the validated Working Alliance Inventory,[270] and Therapist Empathy Scale[271] at Weeks 4 and 8 of the intervention. Feedback will be provided to the interventionist, and measures of warmth and

credibility will be adjusted for in sensitivity analyses, to evaluate if results differ when these measures are included vs. excluded.

Receipt of treatment and enactment of treatment skills: Adherence to the prescribed MB-BP practices will be monitored through class attendance, practice logs and diaries. Adherence data will be collected weekly during the course of the intervention. Mindfulness will also be measured through the use of the validated Five Facet Mindfulness Questionnaire[218] as well as by asking about at home mindfulness practices post-intervention.

10.3.4 Protocol Deviations

Protocol deviations will be reported to the Brown University IRB and NCCIH. A description of the deviations, and any effects on the protection of human subjects will be documented. Investigator(s) will review protocol deviations and determine on a case by case base which data (if any) will be excluded from the final data set.

10.3.5 Monitoring

Please see Section 10.3.4. for description of the monitoring approaches.

11. PARTICIPANT RIGHTS AND CONFIDENTIALITY

11.1 Institutional Review Board (IRB) Review

The protocol, the informed consent document (**Appendix D**), and any subsequent modifications, will be reviewed and approved by the Brown University IRB responsible for oversight of the study.

11.2 Informed Consent Forms

A signed consent form will be obtained from each participant. The consent form will describe the purpose of the study, the procedures to be followed, and the risks and benefits of participation. A signed copy will be offered to each participant and this fact will be documented in the participant's record.

11.3 Participant Confidentiality

The clinical data will be de-identified but linked. Private information such as name, date of birth, and address for recontacting will be kept in a password protected, encrypted database on a different disk that the clinical data help by the Project Coordinator. All paper-based records (i.e. signed consent forms) will be kept in a secure physical location (e.g., locked filing cabinet). All computer entry and networking programs will be done using PIDs only. The principal investigator will only be given access to identifiable personal information for the purposes of patient safety or monitoring by the NIH, data safety monitoring boards or HIPPA compliance officer approved agents. Information will not be released without written permission of the participant, except as necessary for monitoring by IRB, the FDA, the NIH, and the OHRP.

11.4 Study Discontinuation

The study may be discontinued at any time by the IRB, the NCCIH, the OHRP, the FDA, or other government agencies as part of their duties to ensure that research

participants are protected.

12. COMMITTEES

Data Safety Monitoring Board:

Oversight of internal monitoring of the participants' safety will be conducted by the PI, Dr. Eric Loucks. Investigators on this application have extensive experience with clinical trials for mindfulness-based interventions and cardiovascular health outcomes. Oversight of the external Data and Safety Monitoring Committee will be conducted by the chair, Dr. Donald Edmondson, PhD, who is Assistant Professor of Behavioral Medicine at Columbia University Medical Center. He is a Psychologist, and has extensive research experience in evaluating effects of stress and psychosocial factors on cardiovascular disease outcomes, along with clinical trials methods expertise.

The Data and Safety Monitoring committee will also include a board-certified cardiologist, Dr. Gaurav Choudhary, and a biostatistician, Dr. Tao Liu. Dr. Choudhary, MD, is Associate Professor of Medicine at Brown University. He is a practicing clinical cardiologist with research in epidemiology and cardiology. He will be able to advise on clinical outcomes and any cardiovascular complications arising from the study. Dr. Liu, PhD, is an Associate Professor of Biostatistics at Brown University, experienced in clinical trials. He will receive all preliminary analyses from the primary statistician, and will have access to all data from the study, to evaluate any evidence of serious adverse effects or other concerns.

These individuals are not associated with this research project and thus work independently of the PI. They are also not part of the key personnel involved in this grant. They are qualified to review the patient safety data generated by this study because of their unique expertise in the areas of cardiology, psychology/psychiatry, epidemiology, and biostatistics.

Entities Conducting Monitoring

The Institutional Review Board (IRBs) at Brown University will review all research procedures, and will provide oversight. Internal monitoring will be done by the principal investigators (Dr. Loucks) and the Brown University IRB. The Data Safety Monitoring Committee will provide external monitoring, and will meet every six months by phone or in-person. During the randomized-controlled trial phases (phase 3 & 4), they will be provided data every six months to evaluate potential effects of the RCT on the primary outcome (i.e. medical regimen adherence). Any serious adverse effects will be immediately reported to the principal investigator (Loucks) and the committee chair (Edmondson).

13. PUBLICATION OF RESEARCH FINDINGS

Any presentation, abstract, or manuscript will be made available for review by the sponsor and the NCCIH prior to submission.

14. REFERENCES

1. Crane RS, Eames C, Kuyken W, et al. Development and validation of the mindfulness-based interventions - teaching assessment criteria (MBI:TAC). *Assessment.* 2013;20(6):681-688.

2. Crane RS, Kuyken W, Williams JM, Hastings RP, Cooper L, Fennell MJ. Competence in Teaching Mindfulness-Based Courses: Concepts, Development and Assessment. *Mindfulness (N Y).* 2012;3(1):76-84.
3. Crane RS, Brewer J, Feldman C, et al. What defines mindfulness-based programs? The warp and the weft. *Psychol Med.* 2017;47(6):990-999.
4. Onken LS, Carroll KM, Shoham V, Cuthbert BN, Riddle M. Reenvisioning Clinical Science: Unifying the Discipline to Improve the Public Health. *Clinical psychological science : a journal of the Association for Psychological Science.* 2014;2(1):22-34.
5. Tucker KL, Taylor KS, Crawford C, et al. Blood pressure self-monitoring in pregnancy: examining feasibility in a prospective cohort study. *BMC Pregnancy Childbirth.* 2017;17(1):442.
6. Cappuccio FP, Kerry SM, Forbes L, Donald A. Blood pressure control by home monitoring: meta-analysis of randomised trials. *Bmj.* 2004;329(7458):145.
7. Kernan WN, Viscoli CM, Makuch RW, Brass LM, Horwitz RI. Stratified randomization for clinical trials. *J Clin Epidemiol.* 1999;52(1):19-26.
8. Friedman LM, Furberg CD, DeMets DL. *Fundamentals of Clinical Trials.* New York, NY: Springer; 2010.
9. *Research Randomizer (Version 4.0) [Computer software]. Retrieved on June 22, 2013, from* http://www.randomizer.org/ [computer program]. 2013.
10. Whelton PK, Carey RM, Aronow WS, et al. 2017 ACC/AHA/AAPA/ABC/ACPM/AGS/APhA/ASH/ASPC/NMA/PCNA Guideline for the Prevention, Detection, Evaluation, and Management of High Blood Pressure in Adults: Executive Summary: A Report of the American College of Cardiology/American Heart Association Task Force on Clinical Practice Guidelines. *Hypertension.* 2017.
11. Santorelli SF, Kabat-Zinn J. *MBSR Curriculum Guide and Supporting Materials. Mindfulness Based Stress Reduction Professional Training. .* Worcester, MA: University of Massachusetts Center for Mindfulness; 2003.
12. World Health Report 2002. Reducing risks, promoting healthy life. World Health Organization. Geneva, Switzerland. *http://wwwwhoint/whr/2002.* 2002.
13. Institute of Medicine. *Initial National Priorities for Comparative Effectiveness Research.* Washington, D.C.2009.
14. Abbott RA, Whear R, Rodgers LR, et al. Effectiveness of mindfulness-based stress reduction and mindfulness based cognitive therapy in vascular disease: A systematic review and meta-analysis of randomised controlled trials. *Journal of psychosomatic research.* 2014;76(5):341-351.
15. Segal ZV, Williams JMG, Teasdale JD, Kabat-Zinn J. *Mindfulness-Based Cognitive Therapy for Depression.* New York, NY: The Guildford Press; 2012.
16. Bowen S, Witkiewitz K, Clifasefi SL, et al. Relative Efficacy of Mindfulness-Based Relapse Prevention, Standard Relapse Prevention, and Treatment as Usual for Substance Use Disorders: A Randomized Clinical Trial. *JAMA psychiatry.* 2014.
17. Kuyken W, Hayes R, Barrett B, et al. Effectiveness and cost-effectiveness of mindfulness-based cognitive therapy compared with maintenance antidepressant treatment in the prevention of depressive relapse or recurrence (PREVENT): a randomised controlled trial. *Lancet.* 2015;386(9988):63-73.
18. Fisher ES, Staiger DO, Bynum JPW, Gottlieb DJ. Creating accountable care organizations: the extended hospital medical staff. *Health affairs (Project Hope).*26(1):w44-57.
19. Bodenheimer T. Patient Self-management of Chronic Disease in Primary Care. *Jama.* 2002;288(19):2469-2469.
20. Pincus T. Social Conditions and Self-Management Are More Powerful Determinants of Health Than Access to Care. *Annals of internal medicine.* 1998;129(5):406-406.

21. Lorig KR, Mazonson PD, Holman HR. Evidence suggesting that health education for self-management in patients with chronic arthritis has sustained health benefits while reducing health care costs. *Arthritis and rheumatism.* 1993;36(4):439-446.
22. Williams GC, Rodin GC, Ryan RM, Grolnick WS, Deci EL. Autonomous regulation and long-term medication adherence in adult outpatients. *Health psychology : official journal of the Division of Health Psychology, American Psychological Association.* 1998;17(3):269-276.
23. Tucker CM. Self-Regulation Predictors of Medication Adherence Among Ethnically Different Pediatric Patients With Renal Transplants. *Journal of Pediatric Psychology.* 2001;26(8):455-464.
24. Horne R, Weinman J. Self-regulation and Self-management in Asthma: Exploring The Role of Illness Perceptions and Treatment Beliefs in Explaining Non-adherence to Preventer Medication. *Psychology & Health.* 2002;17(1):17-32.
25. Bandura A. Perceived self-efficacy in the exercise of control over AIDS infection. *Evaluation and Program Planning.* 1990;13(1):9-17.
26. Carver CS, Scheier M. *Attention and self-regulation: a control-theory approach to human behavior.* Springer-Verlag; 1981.
27. Carver CS, Scheier MF. *On the Self-Regulation of Behavior.* Cambridge University Press; 2001.
28. Vohs KD, Baumeister RF. Handbook of Self-Regulation, Second Edition: Research, Theory, and Applications. 2010:592.
29. Bishop SR, Lau M, Shapiro S, et al. Mindfulness: A proposed operational definition. *Clin Psychol-Sci Pr.* 2004;11(3):230-241.
30. Hölzel BK, Lazar SW, Gard T, Schuman-Olivier Z, Vago DR, Ott U. How does mindfulness meditation work? Proposing mechanisms of action from a conceptual and neural perspective. *Perspectives on Psychological Science.* 2011;6:537-559.
31. Vago DR. Mapping modalities of self-awareness in mindfulness practice: A potential mechanism for clarifying habits of mind. *Annals of the New York Academy of Sciences.* 2014;1307:28-42.
32. Kabat-Zinn J. Full Catastrophe Living (Revised Edition): Using the Wisdom of Your Body and Mind to Face Stress, Pain, and Illness. 2013.
33. Segal ZV, Williams JMG, Teasdale JD. Mindfulness-Based Cognitive Therapy for Depression (Second Edition). 2013.
34. *Acceptance and Commitment Therapy and the New Behavior Therapies,* 1-29(The Guilford Press 2004).
35. Cullen M. Mindfulness-Based Interventions: An Emerging Phenomenon. *Mindfulness.* 2011;2:186-193.
36. Hayes SC. Acceptance and commitment therapy, relational frame theory, and the third wave of behavioral and cognitive therapies. . *Behavior therapy.* 2004;35:639-665.
37. Samuelson M, Carmody J, Kabat-Zinn J, Bratt MA. Mindfulness-Based Stress Reductioin in Massachusetts Correctional Facilities. *The Prison Journal.* 2007;2:254-268.
38. Sumter MT, Monk-Turner E, Turner C. The benefits of meditation practice in the correctional setting. *Journal of correctional health care : the official journal of the National Commission on Correctional Health Care.* 2009;15(1):47-57; quiz 81.
39. Jha AP, Stanley EA, Kiyonaga A, Wong L, Gelfand L. Examining the protective effects of mindfulness training on working memory capacity and affective experience. *Emotion.* 2010;10(1):54-64.
40. Stanley EA, Schaldach JM, Kiyonaga A, Jha AP. Mindfulness-based Mind Fitness Training: A Case Study of a High-Stress Predeployment Military Cohort. *Cognitive and Behavioral Practice.* 2011;18(4):566-576.

41. Stanley EA, Jha AP. Mind fitness: Improving operational effectiveness and building warrior resilience. *Joint Force Quarterly.* 2009;55:144-151.
42. MLERN, Davidson R, Dunne J, et al. Contemplative Practices and Mental Training: Prospects for American Education. *Child Dev Perspect.* 2012;6(2):146-153.
43. Kaiser-Greenland S. *The Mindful Child.* New York: Free Press; 2010.
44. Greenberg M, Harris A. Nurturing Mindfulness in Children and Youth: Current State of Research. *Child development perspectives.* 2012; 6(2):161–166.
45. Meiklejohn J, Phillips C, Freedman M, et al. Integrating Mindfulness Training into K-12 Education: Fostering the Resilience of Teachers and Students. *Mindfulness.* 2012:1-17.
46. Shapiro S, Brown K, Astin J. Toward the Integration of Meditation into Higher Education: A Review of Research Evidence. *Teachers College Record.* 2011;113(3): 493-528.
47. McCabe Ruff K, Mackenzie ER. The Role of Mindfulness in Healthcare Reform: A Policy Paper. *Explore: The Journal of Science and Healing.* 2009;5(6):313-323.
48. Khoury B, Lecomte T, Fortin G, et al. Mindfulness-based therapy: A comprehensive meta-analysis. *Clinical Psychology Review.* 2013;33:763-771.
49. Goyal M, Singh S, Sibinga EMS, et al. Meditation programs for psychological stress and well-being: a systematic review and meta-analysis. *JAMA internal medicine.* 2014;174:357-368.
50. Olson KL, Emery CF. Mindfulness and weight loss: a systematic review. *Psychosomatic medicine.* 2015;77(1):59-67.
51. Rothman AJ. Toward a theory-based analysis of behavioral maintenance. *Health psychology.* 2000;19:64-69.
52. Rothman AJ, Baldwin AS, Hertel AW, Fuglestad PT. Self-Regulation and Behavior Change: Disentangling Behavioral Initiation and Behavioral Maintenance. In: Vohs KD, Baumeister RF, eds. *Handbook of Self-Regulation, Second Edition: Research, Theory, and Applications*. New York, NY: Guilford Press; 2011:106-122.
53. Muraven M, Baumeister RF. Self-regulation and depletion of limited resources: does self-control resemble a muscle? *Psychological bulletin.* 2000;126(2):247-259.
54. Deci EL, Ryan RM. *Intrinsic Motivation and Self-Determination in Human Behavior.* Springer Science & Business Media; 1985.
55. Hettema J, Steele J, Miller WR. Motivational interviewing. *Annual review of clinical psychology.* 2005;1:91-111.
56. Westra HA, Arkowitz H, Dozois DJA. Adding a motivational interviewing pretreatment to cognitive behavioral therapy for generalized anxiety disorder: a preliminary randomized controlled trial. *Journal of anxiety disorders.* 2009;23(8):1106-1117.
57. Vago DR, Silbersweig DA. Self-awareness, self-regulation, and self-transcendence (S-ART): a framework for understanding the neurobiological mechanisms of mindfulness. *Frontiers in Human Neuroscience.* 2012;6:296.
58. Fletcher LB, Schoendorff B, Hayes SC. Searching for Mindfulness in the Brain: A Process-Oriented Approach to Examining the Neural Correlates of Mindfulness. *Mindfulness.* 2010;1:41-63.
59. Teper R, Inzlicht M. Meditation, mindfulness and executive control: the importance of emotional acceptance and brain-based performance monitoring. *Social cognitive and affective neuroscience.* 2013;8:85-92.
60. Teper R, Segal ZV, Inzlicht M. Inside the Mindful Mind: How Mindfulness Enhances Emotion Regulation Through Improvements in Executive Control. *Current Directions in Psychological Science.* 2013;22:449-454.
61. Chiesa A, Serretti A, Jakobsen JC. Mindfulness: top-down or bottom-up emotion regulation strategy? *Clinical psychology review.* 2013;33:82-96.
62. Marchand WR. Neural mechanisms of mindfulness and meditation: Evidence from neuroimaging studies. *World Journal of Radiology.* 2014;6:471.

63. Fan J, Raz A, Posner M. Attentional Mechanisms. In: Aminoff M, Daroff R, eds. *Encyclopedia of Neurological Sciences*. New York: Elsevier; 2003:292-299.
64. Fan J, McCandliss BD, Sommer T, Raz A, Posner MI. Testing the efficiency and independence of attentional networks. *Journal of Cognitive Neuroscience.* 2002;14(3):340-347.
65. Langner R, Eickhoff SB. Sustaining attention to simple tasks: A meta-analytic review of the neural mechanisms of vigilant attention. *Psychol Bull.* 2013;139(4):870-900.
66. Sturm W, Willmes K. On the functional neuroanatomy of intrinsic and phasic alertness. *Neuroimage.* 2001;14(1 Pt 2):S76-84.
67. Ridderinkhof KR, van den Wildenberg WP, Segalowitz SJ, Carter CS. Neurocognitive mechanisms of cognitive control: the role of prefrontal cortex in action selection, response inhibition, performance monitoring, and reward-based learning. *Brain Cogn.* 2004;56(2):129-140.
68. Rueda MR, Posner MI, Rothbart MK. The development of executive attention: contributions to the emergence of self-regulation. *Dev Neuropsychol.* 2005;28(2):573-594.
69. Dillon DG, Pizzagalli DA. Inhibition of Action, Thought, and Emotion: A Selective Neurobiological Review. *Appl Prev Psychol.* 2007;12(3):99-114.
70. van Gaal S, Lamme VA, Fahrenfort JJ, Ridderinkhof KR. Dissociable brain mechanisms underlying the conscious and unconscious control of behavior. *J Cogn Neurosci.* 2011;23(1):91-105.
71. Schooler JW, Smallwood J, Christoff K, Handy TC, Reichle ED, Sayette MA. Meta-awareness, perceptual decoupling and the wandering mind. *Trends Cogn Sci.* 2011;15(7):319-326.
72. Critchley HD, Wiens S, Rotshtein P, Ohman A, Dolan RJ. Neural systems supporting interoceptive awareness. *Nat Neurosci.* 2004;7(2):189-195.
73. Vago DR, Pan H, Silbersweig DA, Stern E. Neural Substrates Underlying Modalities of Awareness in Mindfulness Practice. Paper presented at: American Neuropsychiatric Assocation Annual Meeting2013; Boston, MA.
74. Valentine E, Sweet P. Meditation and attention: A comparison of the effects of concentrative and mindfulness meditation on sustained attention. *Mental Health, Religion, and culture.* 1999;2(1):59-70.
75. Chan D, Woollacott M. Effects of level of meditation experience on attentional focus: is the efficiency of executive or orientation networks improved? *Journal of Alternative and Complementary Medicine.* 2007;13(6):651-657.
76. Davidson RJ, Goleman DJ, Schwartz GE. Attentional and affective concomitants of meditation: a cross-sectional study. *Journal of Abnormal Psychology.* 1976;85(2):235-238.
77. Slagter HA, Lutz A, Greischar LL, et al. Mental training affects distribution of limited brain resources. *PLoS biology.* 2007;5(6):e138.
78. Lazar SW, Bush G, Gollub RL, Fricchione GL, Khalsa G, Benson H. Functional brain mapping of the relaxation response and meditation. *Neuroreport.* 2000;11(7):1581-1585.
79. Brefczynski-Lewis JA, Lutz A, Schaefer HS, Levinson DB, Davidson RJ. Neural correlates of attentional expertise in long-term meditation practitioners. *Proc Natl Acad Sci U S A.* 2007;104(27):11483-11488.
80. Tang YY, Ma Y, Wang J, et al. Short-term meditation training improves attention and self-regulation. *Proceedings of the National Academy of Science USA.* 2007;104(43):17152-17156.
81. Srinivasan N, Baijal S. Concentrative meditation enhances preattentive processing: a mismatch negativity study. *Neuroreport.* 2007;18(16):1709-1712.

82. Pagnoni G, Cekic M. Age effects on gray matter volume and attentional performance in Zen meditation. *Neurobiology of Aging.* 2007;28(10):1623-1627.
83. Jha AP, Krompinger J, Baime MJ. Mindfulness training modifies subsystems of attention. *Cognitive, Affective, & Behavioral Neuroscience.* 2007;7(2):109-119.
84. Bushell WC. New beginnings: evidence that the meditational regimen can lead to optimization of perception, attention, cognition, and other functions. *Ann N Y Acad Sci.* 2009;1172:348-361.
85. Lutz A, Slagter HA, Rawlings NB, Francis AD, Greischar LL, Davidson RJ. Mental training enhances attentional stability: neural and behavioral evidence. *The Journal of neuroscience : the official journal of the Society for Neuroscience.* 2009;29(42):13418-13427.
86. Wenk-Sormaz H. Meditation can reduce habitual responding. *Advances in Mind-Body Medicine.* 2005;21:33-49.
87. Chambers R, Lo BCY, Allen NB. The impact of intensive mindfulness training on attentional control, cognitive style, and affect. *Cognitive Therapy and Research.* 2008;32(3):303-322.
88. Gross JJ. The emerging field of emotion regulation: An integrative review. *Review of General Psychology.* 1998;2:271-299.
89. Wallis DJ, Hetherington MM. Emotions and eating. Self-reported and experimentally induced changes in food intake under stress. *Appetite.* 2009;52(2):355-362.
90. Cosci F. Nicotine dependence and psychological distress: outcomes and clinical implications in smoking cessation. *Psychology Research and Behavior Management.* 2011;4:119-119.
91. Elfhag K, Rössner S. Who succeeds in maintaining weight loss? A conceptual review of factors associated with weight loss maintenance and weight regain. *Obesity reviews : an official journal of the International Association for the Study of Obesity.* 2005;6(1):67-85.
92. Wing RR, Papandonatos G, Fava JL, et al. Maintaining large weight losses: the role of behavioral and psychological factors. *Journal of consulting and clinical psychology.* 2008;76(6):1015-1021.
93. Magai C, Consedine N, Neugut AI, Hershman DL. Common Psychosocial Factors Underlying Breast Cancer Screening And Breast Cancer Treatment Adherence: A Conceptual Review And Synthesis. *Journal of Women's Health.* 2007;16(1):11-23.
94. Farb NA, Segal ZV, Mayberg H, et al. Attending to the present: mindfulness meditation reveals distinct neural modes of self-reference. *Social Cognitive and Affective Neuroscience.* 2007;2(4):313-322.
95. Taylor VA, Grant J, Daneault V, et al. Impact of mindfulness on the neural responses to emotional pictures in experienced and beginner meditators. *Neuroimage.* 2011.
96. Desbordes G, Negi LT, Pace TW, Wallace BA, Raison CL, Schwartz EL. Effects of mindful-attention and compassion meditation training on amygdala response to emotional stimuli in an ordinary, non-meditative state. *Front Hum Neurosci.* 2012;6:292.
97. Holzel BK, Carmody J, Evans KC, et al. Stress reduction correlates with structural changes in the amygdala. *Soc Cogn Affect Neurosci.* 2010;5(1):11-17.
98. Barnes VA, Treiber FA, Davis H. Impact of Transcendental Meditation on cardiovascular function at rest and during acute stress in adolescents with high normal blood pressure. *Journal of psychosomatic research.* 2001;51(4):597-605.
99. Maclean C, Walton K, Wenneberg S, et al. Altered responses of cortisol, GH, TSH and testosterone in acute stress after four months' practice of Transcendental Meditation (TM). *Ann N Y Acad Sci.* 1994;746:381-384.
100. Sudsuang R, Chentanez V, Veluvan K. The effect of buddhist meditation on serum cortisol and total protein levels, blood pressure, pulse rate, lung volume and reaction time. *Physiol Behav.* 1991;50:543-548.

101. Carlson LE, Speca M, Faris P, Patel KD. One year pre-post intervention follow-up of psychological, immune, endocrine and blood pressure outcomes of mindfulness-based stress reduction (MBSR) in breast and prostate cancer outpatients. *Brain, behavior, and immunity.* 2007;21(8):1038-1049.
102. Ortner CMN, Kilner S, Zelazo PD. Mindfulness meditation and emotional interference in a simple cognitive task. *Motivation and Emotion.* 2007; 31: 271-283.
103. Tang YY, Ma Y, Wang J, et al. Short-term meditation training improves attention and self-regulation. *Proc Natl Acad Sci U S A.* 2007;104(43):17152-17156.
104. Arch JJ, Craske MG. Mechanisms of mindfulness: emotion regulation following a focused breathing induction. *Behaviour research and therapy.* 2006;44(12):1849-1858.
105. Erisman SM, Roemer L. A preliminary investigation of the effects of experimentally induced mindfulness on emotional responding to film clips. *Emotion.* 2010;10(1):72-82.
106. Goldin PR, Gross JJ. Effects of mindfulness-based stress reduction (MBSR) on emotion regulation in social anxiety disorder. *Emotion.* 2010;10(1):83-91.
107. Brewer JA, Sinha R, Chen JA, et al. Mindfulness training and stress reactivity in substance abuse: results from a randomized, controlled stage I pilot study. *Substance abuse : official publication of the Association for Medical Education and Research in Substance Abuse.* 2009;30(4):306-317.
108. Broderick P. Mindfulness and coping with dysphoric mood: Contrasts with rumination and distraction. *Cognitive Therapy and Research.* 2005;29:501-510.
109. Campbell-Sills L, Barlow D, Brown T, Hofmann S. Effects of suppression and acceptance on emotional responses of individuals with anxiety and mood disorders. *Behaviour research and therapy.* 2006;44:1251-1263.
110. Kuehner C, Huffziger S, Liebsch K. Rumination, distraction and mindful self-focus: effects on mood, dysfunctional attitudes and cortisol stress response. *Psychol Med.* 2009;39(2):219-228.
111. Britton WB, Shahar B, Szepsenwol O, Jacobs WJ. Mindfulness-based cognitive therapy improves emotional reactivity to social stress: results from a randomized controlled trial. *Behavior therapy.* 2012;43(2):365-380.
112. Holmes DS. Meditation and somatic arousal reduction. *American Psychologist.* 1984;39(1):1-10.
113. Britton WB, Haynes PL, Fridel KW, Bootzin RR. Polysomnographic and subjective profiles of sleep continuity before and after mindfulness-based cognitive therapy in partially remitted depression. *Psychosom Med.* 2010;72(6):539-548.
114. Creswell JD, Pacilio LE, Lindsay EK, Brown KW. Brief mindfulness meditation training alters psychological and neuroendocrine responses to social evaluative stress. *Psychoneuroendocrinology.* 2014;44:1-12.
115. Bandura A. Self-Efficacy: The Exercise of Control. 1997:604.
116. Schwarzer R. Self-Efficacy: Thought Control Of Action. 1992.
117. Conner, Mark, Norman, Paul. Predicting Health Behaviour. 2005:385.
118. *The role of self-efficacy in health self-regulation,* 137-152(Hogrefe/Huber 2005).
119. Neff KD. The development and validation of a scale to measure self-compassion. *Self and Identity.* 2003;2:223-250.
120. Neff KD, Kirkpatrick KL, Rude SS. Self-compassion and adaptive psychological functioning. *Journal of Research in Personality.* 2007;41:139-154.
121. Neff KD, Vonk R. Self-compassion versus global self-esteem: two different ways of relating to oneself. *J Pers.* 2009;77(1):23-50.
122. Adams CE, Leary MR. Promoting Self–Compassionate Attitudes Toward Eating Among Restrictive and Guilty Eaters. *Journal of Social and Clinical Psychology.* 2007;26:1120-1144.

123. Who Benefits from Training in Self-Compassionate Self-Regulation? A Study of Smoking Reduction, Journal of Social and Clinical Psychology(2010).
124. Magnus CMR, Kowalski KC, McHugh T-LF. The Role of Self-compassion in Women's Self-determined Motives to Exercise and Exercise-related Outcomes. *Self and Identity.* 2010;9:363-382.
125. Terry ML, Leary MR. Self-compassion, self-regulation, and health. *Self and Identity.* 2011;10:352-362.
126. Nolen-Hoeksema S, Wisco BE, Lyubomirsky S. Rethinking rumination. *Perspectives on Psychological Science.* 2008;3:400-424.
127. Smallwood J, Andrews-Hanna J. Not all minds that wander are lost: The importance of a balanced perspective on the mind-wandering state. *Frontiers in Psychology.* 2013;4.
128. McMillan RL, Kaufman SB, Singer JL. Ode to positive constructive daydreaming. *Frontiers in Psychology.* 2013;4.
129. Killingsworth MA, Gilbert DT. A wandering mind is an unhappy mind. *Science.* 2010;330:932.
130. Epel ES, Puterman E, Lin J, Blackburn E, Lazaro A, Mendes WB. Wandering Minds and Aging Cells. *Clinical Psychological Science.* 2012;1:75-83.
131. Smallwood J, Fishman DJ, Schooler JW. Counting the cost of an absent mind: mind wandering as an underrecognized influence on educational performance. *Psychonomic Bulletin & Review.* 2007;14:230-236.
132. Braboszcz C, Delorme A. Lost in thoughts: Neural markers of low alertness during mind wandering. *NeuroImage.* 2010;54:3040-3047.
133. Mooneyham BW, Schooler JW. The costs and benefits of mind-wandering: a review. *Canadian journal of experimental psychology = Revue canadienne de psychologie expérimentale.* 2013;67:11-18.
134. Brewer JA, Worhunsky PD, Gray JR, Tang Y-Y, Weber J, Kober H. Meditation experience is associated with differences in default mode network activity and connectivity. *Proceedings of the National Academy of Sciences of the United States of America.* 2011;108:20254-20259.
135. Farb NAS, Segal ZV, Mayberg HS, et al. Attending to the present: mindfulness meditation reveals distinct neural modes of self-reference. *Social Cognitive and Affective Neuroscience.* 2007;2:313-322.
136. Craig A. How do you feel? Interoception: the sense of the physiological condition of the body. *Nature Reviews Neuroscience.* 2002;3:655-666.
137. *Interoception and emotion: A neuroanatomical perspective,* 06 272-290(Guilford Press 2008).
138. Craig A. The sentient self. *Brain Structure & Function.* 2010;214:563-577.
139. Damasio A, Carvalho GB. The nature of feelings: evolutionary and neurobiological origins. *Nature Reviews Neuroscience.* 2013;14:143-152.
140. Herbert BM, Pollatos O. The body in the mind: on the relationship between interoception and embodiment. *Topics in Cognitive Science.* 2012;4:692-704.
141. Füstös J, Gramann K, Herbert BM, Pollatos O. On the embodiment of emotion regulation: interoceptive awareness facilitates reappraisal. *Social cognitive and affective neuroscience.* 2013;8:911-917.
142. Mehling WE, Wrubel J, Daubenmier JJ, et al. Body Awareness: a phenomenological inquiry into the common ground of mind-body therapies. *Philosophy, ethics, and humanities in medicine : PEHM.* 2011;6:6.
143. Lutz A, Brefczynski-Lewis J, Johnstone T, Davidson RJ. Regulation of the neural circuitry of emotion by compassion meditation: Effects of meditative expertise. *PloS one.* 2008;3:e1897.

144. Lutz A, Greischar LL, Perlman DM, Davidson RJ. BOLD signal in insula is differentially related to cardiac function during compassion meditation in experts vs. novices. *NeuroImage.* 2009;47:1038-1046.
145. Lutz A, McFarlin DR, Perlman DM, Salomons TV, Davidson RJ. Altered anterior insula activation during anticipation and experience of painful stimuli in expert meditators. *NeuroImage.* 2013;64:538-546.
146. Lamm C, Singer T. The role of anterior insular cortex in social emotions. *Brain Structure & Function.* 2010:579-591.
147. Nakata H, Sakamoto K, Kakigi R. Meditation reduces pain-related neural activity in the anterior cingulate cortex, insula, secondary somatosensory cortex, and thalamus. *Frontiers in psychology.* 2014;5:1489.
148. Gard T, Hölzel BK, Sack AT, et al. Pain attenuation through mindfulness is associated with decreased cognitive control and increased sensory processing in the brain. *Cerebral Cortex.* 2012;22:2692-2702.
149. Allen M, Dietz M, Blair KS, et al. Cognitive-affective neural plasticity following active-controlled mindfulness intervention. *Journal of Neuroscience.* 2012;32:15601-15610.
150. Farb NAS, Anderson AK, Mayberg HS, Bean J, McKeon D, Segal ZV. Minding one's emotions: mindfulness training alters the neural expression of sadness. *Emotion.* 2010;10:25-33.
151. Farb NAS, Segal ZV, Anderson AK. Attentional modulation of primary interoceptive and exteroceptive cortices. *Cerebral Cortex.* 2013;23:114-126.
152. Farb NAS, Segal ZV, Anderson AK. Mindfulness meditation training alters cortical representations of interoceptive attention. *Social cognitive and affective neuroscience.* 2013;8:15-26.
153. Luders E, Kurth F, Mayer EA, Toga AW, Narr KL, Gaser C. The unique brain anatomy of meditation practitioners: alterations in cortical gyrification. *Frontiers in Human Neuroscience.* 2012;6:34.
154. Lazar SW, Kerr CE, Wasserman RH, et al. Meditation experience is associated with increased cortical thickness. *Neuroreport.* 2005;16:1893-1897.
155. Hölzel BK, Ott U, Gard T, et al. Investigation of mindfulness meditation practitioners with voxel-based morphometry. *Social cognitive and affective neuroscience.* 2008;3:55-61.
156. Grant JA, Courtemanche J, Rainville P. A non-elaborative mental stance and decoupling of executive and pain-related cortices predicts low pain sensitivity in Zen meditators. *Pain.* 2011;152:150-156.
157. Lutz J, Herwig U, Opialla S, et al. Mindfulness and emotion regulation--an fMRI study. *Social cognitive and affective neuroscience.* 2014;9:776-785.
158. Fox KCR, Nijeboer S, Dixon ML, et al. Is meditation associated with altered brain structure? A systematic review and meta-analysis of morphometric neuroimaging in meditation practitioners. *Neuroscience and biobehavioral reviews.* 2014;43C:48-73.
159. Zeidan F, Martucci KT, Kraft Ra, Gordon NS, McHaffie JG, Coghill RC. Brain mechanisms supporting the modulation of pain by mindfulness meditation. *Journal of Neuroscience.* 2011;31:5540-5548.
160. Brefczynski-Lewis JA, Lutz A, Schaefer HS, Levinson DB, Davidson RJ. Neural correlates of attentional expertise in long-term meditation practitioners. *Proceedings of the National Academy of Sciences of the United States of America.* 2007;104:11483-11488.
161. Kabat-Zinn J. An outpatient program in behavioral medicine for chronic pain patients based on the practice of mindfulness meditation: theoretical considerations and preliminary results. *Gen Hosp Psychiatry.* 1982;4(1):33-47.

162. Kabat-Zinn J, Massion AO, Kristeller J, et al. Effectiveness of a meditation-based stress reduction program in the treatment of anxiety disorders. *The American journal of psychiatry.* 1992;149(7):936-943.
163. Santorelli S, Bonus K, McCallum C, et al. Mindfulness-Based Stress Reduction: Guidelines and Standards of Practice. *Integrative Medicine Mindfulness Program at University of Wisconsin- Madison and the Center for Mindfulness, University of Massachusetts.* 2004.
164. MacCoon DG, Imel ZE, Rosenkranz MA, et al. The validation of an active control intervention for Mindfulness Based Stress Reduction (MBSR). *Behaviour research and therapy.* 2012;50(1):3-12.
165. Almeida ND, Loucks EB, Kubzansky L, et al. Quality of parental emotional care and calculated risk for coronary heart disease. *Psychosom Med.* 2010;72(2):148-155.
166. Everage NJ, Linkletter CD, Gjelsvik A, McGarvey ST, Loucks EB. Implementation of permutation testing to determine clustering of social and behavioral risk factors for coronary heart disease, National Health and Nutrition Examination Survey 2001-2004. *Annals of epidemiology.* 2013;23(7):381-387.
167. Liu SY, Buka SL, Kubzansky LD, Kawachi I, Gilman SE, Loucks EB. Sheepskin effects of education in the 10-year Framingham risk of coronary heart disease. *Soc Sci Med.* 2013;80:31-36.
168. Loucks EB, Abrahamowicz M, Xiao Y, Lynch JW. Associations of education with 30 year life course blood pressure trajectories: Framingham Offspring Study. *BMC Public Health.* 2011;11:139.
169. Loucks EB, Almeida ND, Taylor SE, Matthews KA. Childhood family psychosocial environment and coronary heart disease risk. *Psychosom Med.* 2011;73(7):563-571.
170. Loucks EB, Berkman LF, Gruenewald TL, Seeman TE. Social integration is associated with fibrinogen concentration in elderly men. *Psychosom Med.* 2005;67(3):353-358.
171. Loucks EB, Berkman LF, Gruenewald TL, Seeman TE. Relation of social integration to inflammatory marker concentrations in men and women 70 to 79 years. *Am J Cardiol.* 2006;97(7):1010-1016.
172. Loucks EB, Britton WB, Howe CJ, Eaton CB, Buka SL. Positive associations of dispositional mindfulness with cardiovascular health: The New England Family Study. *Int J Behav Med.* 2014:DOI 10.1007/s12529-12014-19448-12529.
173. Loucks EB, Buka SL, Rogers ML, et al. Education and coronary heart disease risk associations may be affected by early-life common prior causes: a propensity matching analysis. *Annals of epidemiology.* 2012;22(4):221-232.
174. Loucks EB, Lynch JW, Pilote L, et al. Life-course socioeconomic position and incidence of coronary heart disease: the Framingham Offspring Study. *American journal of epidemiology.* 2009;169(7):829-836.
175. Loucks EB, Magnusson KT, Cook S, Rehkopf DH, Ford ES, Berkman LF. Socioeconomic position and the metabolic syndrome in early, middle, and late life: evidence from NHANES 1999-2002. *Annals of epidemiology.* 2007;17(10):782-790.
176. Loucks EB, Taylor SE, Polak JF, Wilhelm A, Kalra P, Matthews KA. Childhood family psychosocial environment and carotid intima media thickness: the CARDIA study. *Soc Sci Med.* 2014;104:15-22.
177. Senese LC, Almeida ND, Fath AK, Smith BT, Loucks EB. Associations between childhood socioeconomic position and adulthood obesity. *Epidemiol Rev.* 2009;31:21-51.
178. Smith BT, Lynch JW, Fox CS, et al. Life-course socioeconomic position and type 2 diabetes mellitus: The Framingham Offspring Study. *American journal of epidemiology.* 2011;173(4):438-447.

179. Altunkan S, Iliman N, Altunkan E. Validation of the Omron M6 (HEM-7001-E) upper arm blood pressure measuring device according to the International Protocol in elderly patients. *Blood pressure monitoring.* 2008;13(2):117-122.
180. Tucker KL, Sheppard JP, Stevens R, et al. Self-monitoring of blood pressure in hypertension: A systematic review and individual patient data meta-analysis. *PLoS medicine.* 2017;14(9):e1002389.
181. Roger VL, Go AS, Lloyd-Jones DM, et al. Heart disease and stroke statistics--2012 update: a report from the American Heart Association. *Circulation.* 2012;125(1):e2-e220.
182. Subar AF, Thompson FE, Kipnis V, et al. Comparative validation of the Block, Willett, and National Cancer Institute food frequency questionnaires : the Eating at America's Table Study. *Am J Epidemiol.* 2001;154(12):1089-1099.
183. Chobanian AV, Bakris GL, Black HR, et al. Seventh report of the Joint National Committee on Prevention, Detection, Evaluation, and Treatment of High Blood Pressure. *Hypertension.* 2003;42(6):1206-1252.
184. Centers for Disease Control and Prevention. *Behavioral Risk Factor Surveillance System Survey (BRFSS) Questionnaire.* Atlanta, GA: US Department of Health and Human Services; 2011.
185. Information Mediary Corp. eCAP Validation Information. http://www.informationmediary.com/ecap. 2018.
186. Loucks EB, Gilman SE, Howe CJ, et al. Education and Coronary Heart Disease Risk: Potential Mechanisms Such as Literacy, Perceived Constraints, and Depressive Symptoms. *Health education & behavior : the official publication of the Society for Public Health Education.* 2014.
187. Craig CL, Marshall AL, Sjostrom M, et al. International physical activity questionnaire: 12-country reliability and validity. *Med Sci Sports Exerc.* 2003;35(8):1381-1395.
188. Lee PH, Macfarlane DJ, Lam TH, Stewart SM. Validity of the International Physical Activity Questionnaire Short Form (IPAQ-SF): a systematic review. *The international journal of behavioral nutrition and physical activity.* 2011;8:115.
189. Loucks EB, Britton WB, Howe CJ, Eaton CB, Buka SL. Positive Associations of Dispositional Mindfulness with Cardiovascular Health: the New England Family Study. *Int J Behav Med.* 2015;22(4):540-550.
190. University of Massachusetts Medical School. Center for Mindfulness in Medicine HC, and Society. 2013; http://www.umassmed.edu/cfm/oasis/ataglance/index.aspx
http://www.umassmed.edu/cfm/oasis/indepth/index.aspx. Accessed April 18, 2013, 2013.
191. Evans A, Crane R, Cooper L, et al. A Framework for Supervision for Mindfulness-Based Teachers: a Space for Embodied Mutual Inquiry. *Mindfulness (N Y).* 2015;6(3):572-581.
192. Centers for Disease Control and Prevention. High Blood Pressure. Website: https://www.cdc.gov/bloodpressure/index.htm; last accessed on Sept. 29, 2018. 2018.
193. American Heart Association. The Facts About High Blood Pressure. Website: http://www.heart.org/en/health-topics/high-blood-pressure/the-facts-about-high-blood-pressure. Last accessed: September 29, 2018. 2017.
194. Crane RS, Soulsby JG, Kuyken W, Williams JMG, Eames C. The Bangor, Exeter and Oxford Mindfulness-Based Interventions Teaching Assessment Criteria for assessing the competence and adherence of mindfulness-based class-based teaching within the UK context. *Document in development.* 2011.
195. Bernstein DP, Fink L, Handelsman L, et al. Initial reliability and validity of a new retrospective measure of child abuse and neglect. *The American journal of psychiatry.* 1994;151(8):1132-1136.
196. Bifulco A, Bernazzani O, Moran PM, Jacobs C. The childhood experience of care and abuse questionnaire (CECA.Q): validation in a community series. *The British journal of clinical psychology.* 2005;44(Pt 4):563-581.

197. Bifulco A, Brown GW, Harris TO. Childhood Experience of Care and Abuse (CECA): a retrospective interview measure. *Journal of child psychology and psychiatry, and allied disciplines.* 1994;35(8):1419-1435.
198. Bernstein DP, Ahluvalia T, Pogge D, Handelsman L. Validity of the Childhood Trauma Questionnaire in an adolescent psychiatric population. *J Am Acad Child Adolesc Psychiatry.* 1997;36(3):340-348.
199. Spinhoven P, Penninx BW, Hickendorff M, van Hemert AM, Bernstein DP, Elzinga BM. Childhood Trauma Questionnaire: factor structure, measurement invariance, and validity across emotional disorders. *Psychol Assess.* 2014;26(3):717-729.
200. Merrick MT, Ford DC, Ports KA, Guinn AS. Prevalence of Adverse Childhood Experiences From the 2011-2014 Behavioral Risk Factor Surveillance System in 23 States. *JAMA Pediatr.* 2018;172(11):1038-1044.
201. Andresen EM, Malmgren JA, Carter WB, Patrick DL. Screening for depression in well older adults: evaluation of a short form of the CES-D (Center for Epidemiologic Studies Depression Scale). *Am J Prev Med.* 1994;10(2):77-84.
202. Van Dam NT, Earleywine M. Validation of the Center for Epidemiologic Studies Depression Scale--Revised (CESD-R): pragmatic depression assessment in the general population. *Psychiatry Res.* 2011;186(1):128-132.
203. Beck AT, Epstein N, Brown G, Steer RA. An inventory for measuring clinical anxiety: psychometric properties. *Journal of consulting and clinical psychology.* 1988;56(6):893-897.
204. Dobson KS. An analysis of anxiety and depression scales. *J Pers Assess.* 1985;49(5):522-527.
205. Enns MW, Cox BJ, Parker JD, Guertin JE. Confirmatory factor analysis of the Beck Anxiety and Depression Inventories in patients with major depression. *J Affect Disord.* 1998;47(1-3):195-200.
206. Fydrich T, Dowdall D, Chambless DL. Reliability and validity of the Beck Anxiety Inventory. *J Anxiety Disord.* 1992;6:55-61.
207. Osman A, Kopper BA, Barrios FX, Osman JR, Wade T. The Beck Anxiety Inventory: reexamination of factor structure and psychometric properties. *J Clin Psychol.* 1997;53(1):7-14.
208. Kohn PM, Kantor L, DeCicco TL, Beck AT. The Beck Anxiety Inventory-Trait (BAIT): a measure of dispositional anxiety not contaminated by dispositional depression. *J Pers Assess.* 2008;90(5):499-506.
209. Piotrowski C. The status of the Beck Anxiety Inventory in contemporary research. *Psychol Rep.* 1999;85(1):261-262.
210. Pickering TG, Hall JE, Appel LJ, et al. Recommendations for blood pressure measurement in humans and experimental animals: part 1: blood pressure measurement in humans: a statement for professionals from the Subcommittee of Professional and Public Education of the American Heart Association Council on High Blood Pressure Research. *Circulation.* 2005;111(5):697-716.
211. Coleman A, Freeman P, Steel S, Shennan A. Validation of the Omron 705IT (HEM-759-E) oscillometric blood pressure monitoring device according to the British Hypertension Society protocol. *Blood pressure monitoring.* 2006;11(1):27-32.
212. Behavioral Risk Factor Surveillance System Survey Data. Department of Health and Human Services, Centers for Disease Control and Prevention; 2018. Accessed March 27 2108.
213. Buysse DJ, Reynolds CF, 3rd, Monk TH, Berman SR, Kupfer DJ. The Pittsburgh Sleep Quality Index: a new instrument for psychiatric practice and research. *Psychiatry Res.* 1989;28(2):193-213.

214. Buysse DJ, Reynolds CF, 3rd, Monk TH, Hoch CC, Yeager AL, Kupfer DJ. Quantification of subjective sleep quality in healthy elderly men and women using the Pittsburgh Sleep Quality Index (PSQI). *Sleep.* 1991;14(4):331-338.
215. Gentili A, Weiner DK, Kuchibhatla M, Edinger JD. Test-retest reliability of the Pittsburgh sleep quality index in nursing home residents. *J Am Geriatr Soc.* 1995;43(11):1317-1318.
216. Baer RA, Smith GT, Hopkins J, Krietemeyer J, Toney L. Using self-report assessment methods to explore facets of mindfulness. *Assessment.* 2006;13(1):27-45.
217. Karlsson J, Persson LO, Sjostrom L, Sullivan M. Psychometric properties and factor structure of the Three-Factor Eating Questionnaire (TFEQ) in obese men and women. Results from the Swedish Obese Subjects (SOS) study. *Int J Obes Relat Metab Disord.* 2000;24(12):1715-1725.
218. Cappelleri JC, Bushmakin AG, Gerber RA, et al. Psychometric analysis of the Three-Factor Eating Questionnaire-R21: results from a large diverse sample of obese and non-obese participants. *Int J Obes (Lond).* 2009;33(6):611-620.
219. Roberti JW, Harrington LN, Storch EA. Further psychometric support for the 10-item version of the perceived stress scale. *J Coll Counsel.* 2006;9:135-147.
220. Cohen S, Kamarck T, Mermelstein R. A global measure of perceived stress. *J Health Soc Behav.* 1983;24(4):385-396.
221. Gratz KL, Roemer L. Multidimensional assessment of emotion regulation and dysregulation: Development, factor structure, and initial validation of the Difficulties in Emotion Regulation Scale. *J Psychopathol Behav Assess.* 2004(26):41-54.
222. Mehling WE, Gopisetty V, Daubenmier J, Price CJ, Hecht FM, Stewart A. Body awareness: construct and self-report measures. *PLoS One.* 2009;4(5):e5614.
223. Mehling WE, Price C, Daubenmier JJ, Acree M, Bartmess E, Stewart A. The Multidimensional Assessment of Interoceptive Awareness (MAIA). *PLoS One.* 2012;7(11):e48230.
224. Bornemann B, Herbert BM, Mehling WE, Singer T. Differential changes in self-reported aspects of interoceptive awareness through 3 months of contemplative training. *Frontiers in psychology.* 2014;5:1504.
225. Brener J, Liu X, Ring C. A method of constant stimuli for examining heartbeat detection: comparison with the Brener-Kluvitse and Whitehead methods. *Psychophysiology.* 1993;30(6):657-665.
226. Fresco DM, Moore MT, van Dulmen MH, et al. Initial psychometric properties of the experiences questionnaire: validation of a self-report measure of decentering. *Behavior therapy.* 2007;38(3):234-246.
227. Fresco DM, Segal ZV, Buis T, Kennedy S. Relationship of posttreatment decentering and cognitive reactivity to relapse in major depression. *J Consult Clin Psychol.* 2007;75(3):447-455.
228. Robertson IH, Manly T, Andrade J, Baddeley BT, Yiend J. 'Oops!': performance correlates of everyday attentional failures in traumatic brain injured and normal subjects. *Neuropsychologia.* 1997;35(6):747-758.
229. Molenberghs P, Gillebert CR, Schoofs H, Dupont P, Peeters R, Vandenberghe R. Lesion neuroanatomy of the Sustained Attention to Response task. *Neuropsychologia.* 2009.
230. Fassbender C, Murphy K, Foxe JJ, et al. A topography of executive functions and their interactions revealed by functional magnetic resonance imaging. *Brain Res Cogn Brain Res.* 2004;20(2):132-143.
231. Morrison AB, Goolsarran M, Rogers SL, Jha AP. Taming a wandering attention: short-form mindfulness training in student cohorts. *Front Hum Neurosci.* 2014;7:897.
232. Cheyne JA, Carriere JS, Smilek D. Absent-mindedness: Lapses of conscious awareness and everyday cognitive failures. *Conscious Cogn.* 2006;15(3):578-592.

233. Mrazek MD, Smallwood J, Schooler JW. Mindfulness and mind-wandering: Finding convergence through opposing constructs. *Emotion.* 2012.
234. May J, Andrade J, Kavanagh DJ, et al. The craving experience questionnaire: a brief, theory-based measure of consummatory desire and craving. *Addiction.* 2014;109(5):728-735.
235. Cohen S, Mermelstein R, Kamarck T, Hoberman H. Measuring the functional components of social support. In: Sarason IG, Sarason BR, eds. *Social support: Theory, research and application*. The Hague, The Netherlands: Martinus Nijhoff; 1985.
236. Russell D, Peplau LA, Cutrona CE. The revised UCLA Loneliness Scale: concurrent and discriminant validity evidence. *J Pers Soc Psychol.* 1980;39(3):472-480.
237. Hays RD, Bjorner JB, Revicki DA, Spritzer KL, Cella D. Development of physical and mental health summary scores from the patient-reported outcomes measurement information system (PROMIS) global items. *Qual Life Res.* 2009;18(7):873-880.
238. Baumeister RF. Yielding to temptation: Self-control failure, impulsive purchasing, and consumer behavior. *J Consum Res.* 2002;28(4):670-676.
239. Tangney JP, Baumeister RF, Boone AL. High self-control predicts good adjustment, less pathology, better grades, and interpersonal success. *J Pers.* 2004;72(2):271-324.
240. Connor KM, Davidson JR. Development of a new resilience scale: the Connor-Davidson Resilience Scale (CD-RISC). *Depression and anxiety.* 2003;18(2):76-82.
241. Lorig KR, Sobel DS, Ritter PL, Laurent D, Hobbs M. Effect of a self-management program on patients with chronic disease. *Eff Clin Pract.* 2001;4(6):256-262.
242. Debski TT, Kamarck TW, Jennings JR, Young LW, Eddy MJ, Zhang YX. A computerized test battery for the assessment of cardiovascular reactivity. *International journal of bio-medical computing.* 1991;27(3-4):277-289.
243. Koffarnus MN, Bickel WK. A 5-trial adjusting delay discounting task: accurate discount rates in less than one minute. *Experimental and clinical psychopharmacology.* 2014;22(3):222-228.
244. National Center for Complementary and Integrative Health (NCCIH). Meditation: An Introduction. Uses of Meditation in the United States. http://nccih.nih.gov/health/meditation/overview.htm#meditation. 2014.
245. Liang K-Y, Zeger SL. Longitudinal data analysis using generalized linear models. *Biometrika.* 1986;73(1):13-22.
246. Zeger SL, Liang K-Y. Longitudinal data analysis for discrete and continuous outcomes. *Biometrics.* 1986;42:121-130.
247. Valeri L, Vanderweele TJ. Mediation Analysis Allowing for Exposure-Mediator Interactions and Causal Interpretation: Theoretical Assumptions and Implementation With SAS and SPSS Macros. *Psychological methods.* 2013.
248. Fritz MS, Mackinnon DP. Required sample size to detect the mediated effect. *Psychological science.* 2007;18(3):233-239.
249. Cohen J. *Statistical power analysis for the behavioral sciences (2nd ed.).* Hillsdale, NJ: Erlbaum; 1988.
250. O'Brien WH, Reid GJ, Jones KR. Differences in heartbeat awareness among males with higher and lower levels of systolic blood pressure. *International journal of psychophysiology : official journal of the International Organization of Psychophysiology.* 1998;29(1):53-63.
251. Bornemann B, Singer T. Taking time to feel our body: Steady increases in heartbeat perception accuracy and decreases in alexithymia over 9 months of contemplative mental training. *Psychophysiology.* 2016.
252. Avery JA, Drevets WC, Moseman SE, Bodurka J, Barcalow JC, Simmons WK. Major depressive disorder is associated with abnormal interoceptive activity and functional connectivity in the insula. *Biological psychiatry.* 2014;76(3):258-266.

253. Farb NA, Segal ZV, Anderson AK. Mindfulness meditation training alters cortical representations of interoceptive attention. *Soc Cogn Affect Neurosci.* 2013;8(1):15-26.
254. Lezak MD, Howieson DB, Loring DW. *Neuropsychological Assessment.* 4th ed. New York, NY: Oxford University Press; 2004.
255. Kamarck TW, Jennings JR, Pogue-Geile M, Manuck SB. A multidimensional measurement model for cardiovascular reactivity: stability and cross-validation in two adult samples. *Health Psychol.* 1994;13(6):471-478.
256. Matthews KA, Katholi CR, McCreath H, et al. Blood pressure reactivity to psychological stress predicts hypertension in the CARDIA study. *Circulation.* 2004;110(1):74-78.
257. Matthews KA, Woodall KL, Allen MT. Cardiovascular reactivity to stress predicts future blood pressure status. *Hypertension.* 1993;22(4):479-485.
258. Steptoe A, Marmot M. Impaired cardiovascular recovery following stress predicts 3-year increases in blood pressure. *J Hypertens.* 2005;23(3):529-536.
259. Steptoe A, Kivimaki M. Stress and cardiovascular disease. *Nature reviews Cardiology.* 2012;9(6):360-370.
260. Crane MM, Ward DS, Lutes LD, Bowling JM, Tate DF. Theoretical and Behavioral Mediators of a Weight Loss Intervention for Men. *Ann Behav Med.* 2016;50(3):460-470.
261. Wingo BC, Desmond Ra Fau - Brantley P, Brantley P Fau - Appel L, et al. Self-efficacy as a predictor of weight change and behavior change in the PREMIER trial. *Nutr Educ Behav.* 2013;45(1878-2620 (Electronic)):314-321.
262. Timmerman GM, Brown A. The effect of a mindful restaurant eating intervention on weight management in women. *Journal of nutrition education and behavior.* 2012;44(1):22-28.
263. Burke V, Mansour J Fau - Mori TA, Mori Ta Fau - Beilin LJ, Beilin Lj Fau - Cutt HE, Cutt He Fau - Wilson A, Wilson A. Changes in cognitive measures associated with a lifestyle program for treated hypertensives: a randomized controlled trial (ADAPT). 2008(0268-1153 (Print)).
264. Cornelio ME, Godin G, Rodrigues RC, de Freitas Agondi R, Alexandre NM, Gallani MC. Effect of a behavioral intervention of the SALdavel program to reduce salt intake among hypertensive women: A randomized controlled pilot study. *Eur J Cardiovasc Nurs.* 2016;15(3):e85-94.
265. Irwan AM, Kato M, Kitaoka K, Ueno E, Tsujiguchi H, Shogenji M. Development of the salt-reduction and efficacy-maintenance program in Indonesia. *Nursing & health sciences.* 2016;18(4):519-532.
266. Meuleman Y, Hoekstra T, Dekker FW, et al. Sodium Restriction in Patients With CKD: A Randomized Controlled Trial of Self-management Support. *Am J Kidney Dis.* 2017;69(5):576-586.
267. Choi SH, Choi-Kwon S. The effects of the DASH diet education program with omega-3 fatty acid supplementation on metabolic syndrome parameters in elderly women with abdominal obesity. *Nutr Res Pract.* 2015;9(2):150-157.
268. Bellg AJ, Borrelli B, Resnick B, et al. Enhancing treatment fidelity in health behavior change studies: best practices and recommendations from the NIH Behavior Change Consortium. *Health Psychol.* 2004;23(5):443-451.
269. Segal ZV, Teasdale JD, Williams JM, Gemar MC. The Mindfulness-Based Cognitive Therapy Adherence Scale: inter-rater reliability, adherence to protocol and treatment distinctiveness. *Clin Psychol Psychother.* 2002;9:131-138.
270. Horvath AO, Greenberg LS. Development and Validation of the Working Alliance Inventory. *J Couns Psychol.* 1989;36(2):223-233.
271. Burns DD, Auerbach A. Therapeutic empathy in cognitive-behavioural therapy: does it really make a difference? In: Salkovskis P, ed. *Frontiers of Cognitive Therapy*. New York: Guilford; 1996.

272. Britton W, Shahar B. Mindfulness Skill Acquisition Scale. *University of Arizona.* 2003.

15. SUPPLEMENTS/APPENDICES

Note that minor modifications may be made to the individual documents listed in the appendices during the course of the study.

**Appendix A**	**MB-BP Curriculum Guide**
**Appendix B**	**MBSR Curriculum Guide**
**Appendix C**	**MB-BP Phone Screener (verbal consent process)**
**Appendix D**	**MB-BP Informed Consent**
**Appendix E**	**Anthropometric Quality Control Manual**
**Appendix F**	**Blood Pressure Quality Control Manual**
