# Supplementary material for: Adapted Mindfulness Training for Interoception and Adherence to the DASH Diet: A Phase 2 Randomized Clinical Trial
Source: JAMA Netw Open. 2023 Nov 2;6(11):e2339243. doi: 10.1001/jamanetworkopen.2023.39243 (PMC10623198; doi:10.1001/jamanetworkopen.2023.39243)
Supplement: Supplement 2. — eMethods 1. MB-BP Program Description, With Emphasis on Dietary Change Components eMethods 2. Detailed Methods on Measurement of Primary Outcome (MAIA) and Secondary Outcome (DASH Diet Adherence) eMethods 3. Measurement of Covariates eTable 1. Evaluation of Differential Missingness eTable 2. Mediation Analyses [file jamanetwopen-e2339243-s002.pdf]

Supplementary Online Content

Loucks EB, Kronish IM, Saadeh FB, et al. Adapted mindfulness training for interoception and adherence to the Dietary Approaches to Stop Hypertension (DASH) diet: a randomized clinical trial. *JAMA Netw Open*. 2023;6(10):e2339243. doi:10.1001/jamanetworkopen.2023.39243

eMethods 1. MB-BP Program Description, With Emphasis on Dietary Change Components

eMethods 2. Detailed Methods on Measurement of Primary Outcome (MAIA) and Secondary Outcome (DASH Diet Adherence)

eMethods 3. Measurement of Covariates

eTable 1. Evaluation of Differential Missingness

eTable 2. Mediation Analyses

eReferences.

This supplementary material has been provided by the authors to give readers additional information about their work.

© 2023 Loucks EB et al. *JAMA Network Open*.

eMethods 1. MB-BP Program Description, With Emphasis on Dietary Change Components

MB-BP is based on, and time-matched to, the standardized Mindfulness-Based Stress Reduction (MBSR) program.[1] Both consist of a group orientation session, eight 2.5-hour weekly group sessions, and a 7.5-hour one-day group session. Recommended home mindfulness practice was ≥45 minutes/day, 6 days/week. The unique areas of MB-BP were education about hypertension risk factors, along with specific mindfulness modules focused on mindful awareness of diet, physical activity, medication adherence, alcohol consumption, stress, and social support for behavior change. Specific education on the DASH diet and the United States Department of Agriculture (USDA) and United States Department of Health and Human Services (HHS) dietary guidelines were provided.[2] A mindful eating practice involving emotionally challenging foods such as sweet or salty items was performed in class, with participants encouraged to notice and communicate with the group their physical sensations, thoughts and emotions before, during, and after eating the food. Home practices invited participants to set intentions for the week related to diet, for participants who were motivated to change their diet. Peer groups were formed with shared health behaviors participants were working on (e.g., diet, physical activity, antihypertensive medication adherence), to share progress, challenges, and experiences. During the retreat, a flavorful DASH diet-consistent lunch was provided, encouraging half the plate to be filled with fruits and vegetables, consistent with USDA and HHS dietary guidelines, [2] and a mindful eating exercise was performed noticing the physical sensations, thoughts and emotions before during and after eating the meal. Similar home practices were assigned. MB-BP builds a foundation of mindfulness skills (e.g., meditation, yoga, self-awareness, attention control, emotion regulation) through the MBSR curriculum. MB-BP directs those skills towards participants' adhering to behaviors that can lower BP (see manuscript **Figure 1**).[1] MB-BP participants have their BP and hypertension risk factors (including dietary patterns) directly assessed at

© 2023 Loucks EB et al. *JAMA Network Open*.

baseline, and are provided with this information during the orientation session. During this session, the importance of hypertension for health and mortality is described, along with hypertension risk factors. This phase aims to engage participants' interest in hypertension risk factors, and increase motivation for behavior change. MB-BP encourages participants to explore personal readiness for change in the different hypertension risk factors (including diet), and explore utilizing mindfulness practices to engage with those risk factors that they choose. Instructors hold twenty-minute one-on-one interviews with each participant at the beginning of the course to foster a relationship between the instructor and participant, identify reasons for participation, and pinpoint opportunities for the instructor to customize the course to individual participants.[1]

The course focuses on different determinants of BP each week. However, four common themes exist across all BP determinants, including: (1) Awareness of thoughts, emotions and physical sensations particularly surrounding determinants of BP such as food overconsumption, sedentary activities, alcohol consumption, and antihypertensive medication adherence. (2) Craving, particularly for determinants of BP such as overconsumption of palatable foods (e.g., those high in salt or sugar), sedentary activities, and alcohol consumption. (3) The impact of bringing mindfulness to every moment, particularly in relation to BP determinants, recognizing that this present moment is influenced by prior moments, including what we ate, the physical activity we had, and the amount of alcohol consumed. Participants are trained to bring non-judgmental attention to the often short-term pleasures of overconsumption of foods, sedentary activities, heavy alcohol consumption, or not taking antihypertensive medications, as well as to bring non-judgmental attention to the longer term suffering associated with these activities. Through this process, participants are encouraged to reflect on whether behavioral choices provide more benefit or harm to their well-being, and to choose beneficial behaviors. (4) Self-compassion: as self-regulatory and self-awareness skills increase as a result of the mindfulness practices; the curriculum emphasizes that it is common for participants to start caring for themselves in kinder ways. It is a way of better knowing ourselves, and through knowing ourselves in each

© 2023 Loucks EB et al. *JAMA Network Open*.

moment, we often want to care for ourselves in each moment. This may mean taking medication that will support health, or being more physically active, eating more healthily, or consuming alcohol in more moderate amounts. As a whole, the MB-BP program trains participants in mindfulness skills, and then supports them to apply those skills to determinants of BP most relevant in their lives. After the 8-week course is completed, the only curriculum offerings are optional weekly mindfulness community group meetings offered free to the public by the Mindfulness Center at Brown, one-day retreats three times per year, and website access to meditations and talks.[1]

© 2023 Loucks EB et al. *JAMA Network Open*.

Overview of how MB-BP customized Mindfulness-Based Stress Reduction (MBSR). All MBSR modules are maintained (not shown here), but many are slightly abbreviated to make room for the novel MB-BP modules shown below.

Module	Intervention Component	Customization Description	New (N) or modified (M)*	In Class (IC) v Home Practice (HP)	Time (min)
Orientation Session	Personalized Health Feedback on Hypertension Risk Factors	Provide participants health cards showing their baseline-assessed BP and hypertension risk factor levels, including *SBP, *DBP, *BMI, diet, physical activity, perceived stress, sleep, and antihypertensive medication use.	N	*IC*	10
	Introduce Impact of Hypertension on Cardiovascular Disease	Describe key modifiable hypertension risk factors, how the current societal environment promotes hypertension, how mindfulness meditation can impact risk factors.	N	*IC*	30
	Course Logistics Overview	Explore our behavioral, physical, emotional and cognitive patterns related to hypertension risk, more skillful responses to these patterns, as well as more mindful ways to communicate and choose nourishing behaviors and activities.	M	*IC*	10
1:1 Interviews	Individual Intake Interviews for Instructor with Each Participant	Participants are asked (1) what brought them to be interested in learning about mindfulness at this time in their lives; and (2) what is their relationship with determinants of blood pressure such as diet, overweight/obesity, physical activity, alcohol consumption, stress reactivity and antihypertensive medication use. Conversational explorations occur determining in what areas participants are ready to change, and how best the course can support them to do so.	M	*IC*	20
Class 1	Introduction Behavior Change Theory & Hypertension	Overview of the theory of behavioral medicine and the application of self-regulatory skills as related to hypertension.	M	*IC*	10
Class 2	Mindful Eating of Highly Palatable (e.g. high sugar/fat/salt) Food Item	Participants invited to engage in eating a highly palatable snack, and reflect on the effects they experience in thoughts, emotions, and physical sensations.	N	*IC*	20
	Pleasant Events Calendar	Suggest targeting pleasant events related to hypertension risk (e.g. eating or alcohol consumption).	M	*HP*	20
Class 3	Information & Review of Basic Forms of Physical Activity	(1) Strength training; (2) Aerobic training; (3) Flexibility training; according to American Heart Association guidelines. Dyad & group discussion.	N	*IC*	25
	Unpleasant Events Calendar	Suggest targeting unpleasant events related to hypertension risk (e.g. eating or alcohol consumption).	M	*HP*	20
	Physical Activity	Mindful attention to thoughts, emotions and physical sensations related to the physical activity beforehand, during and after engaging in the activity.	N	*HP*	30
Class 4	Aerobic Physical Activity	Walking/jogging, allowing modifications of physical activity as needed (e.g. body weight-based strength exercises). Bringing mindful awareness to physical activity, especially thoughts, emotions and physical sensations before, during and after physical activity. Group discussion.	N	*IC*	20
	Motivational Interviewing Module	Goal setting worksheet related to improving a determinant of blood pressure during the coming week; group discussion.	M	*IC*	20
	Blood Pressure Determinant Goal Setting	Pick a do-able goal related to diet, alcohol consumption, or physical activity for the week that participants feel may help them (e.g. eating more healthily, engaging in particular physical activity); make it specific; pick a way to measure it.	M	*HP*	
Class 5	Blood Pressure Determinant Goal Break Out Groups	Small group sharing, where group members cluster by blood pressure determinant they set goals on during prior week (e.g. physical activity, diet). Share experiences practicing with their goal over the past week.	N	*IC*	15
	Medication Adherence Group Discussion	Hand out fact sheet on antihypertensive medication classes, including potential benefits and adverse effects. Explore through group discussion participants' relationship with antihypertensive medication use. Sharing how it can be skillful to take medication if that is what body needs.	N	*IC*	15
	Motivational Interviewing Module	Goal setting worksheet related to improving a determinant of blood pressure during the coming week; group discussion.	M	*IC*	15
	Blood Pressure Determinant Goal Setting	Pick a do-able goal related to diet, alcohol consumption, physical activity or antihypertensive medication use for the week that participants feel may help them; make it specific; pick a way to measure it.	M	*HP*	
Class 6	Blood Pressure Determinant Goal Break Out Groups	Small group sharing, where group members cluster by blood pressure determinant they set goals on during prior week (e.g. physical activity, alcohol, diet). Share experiences practicing with their goal over the past week.	N	*IC*	20
	Social Support Module	Journaling exercise picking one of the areas you are exploring shifting (e.g. meditation, physical activity, diet, medication adherence) and the social support available in the participants life to support this goal. Group discussion.	N	*IC*	20
	Blood Pressure Determinant Goal Setting	Pick a do-able goal related to diet, alcohol consumption, physical activity, antihypertensive medication use, or increasing social support related to determinant of blood pressure, for the week; make it specific; pick a way to measure it.	M	*HP*	
Class 7	Optional Blood Pressure Determinant Goal Setting	Invitation, if participants would like to, pick a do-able goal related to determinant of blood pressure, for the week; make it specific; pick a way to measure it.	M	*HP*	10

© 2023 Loucks EB et al. *JAMA Network Open*.

Class 8	Theoretical Mechanism for Mindfulness on Cardiovascular Disease	Show the framework through which mindfulness may influence blood pressure and heart health, and how this course worked with those pathways.	N	*HP*	20
All-Day retreat	Aerobic Physical Activity	Silent aerobic or strength physical activity. Aware of physical sensations, thoughts and emotions.	N	IC	40
	Dietary Approaches to Stop Hypertension (DASH) diet lunch	DASH diet & 2015-2020 Dietary Guidelines for Americans inviting participants to fill their plates accordingly, noticing physical sensations, thoughts and emotions during the entire arch of lunch, including before, during, and after.	N	IC	60
	Self-Care Written Reflection	Invite participants to write about self-care in response to pre-set prompts.	N	IC	15

**BMI, body mass index; DBP, diastolic blood pressure; SBP, systolic blood pressure*.

© 2023 Loucks EB et al. *JAMA Network Open*.

eMethods 2. Detailed Methods on Measurement of Primary Outcome (MAIA) and Secondary Outcome (DASH Diet Adherence)

The pre-specified primary outcome was interoceptive awareness, measured using the validated Multidimensional Assessment of Interoceptive Awareness (MAIA).[3,4] While the MAIA has eight dimensions, each with a separate scale, we utilized an eight-dimension average score (range 0 to 5) across the eight scales (i.e., mean score of the eight scales) to avoid issues of multiple statistical testing, similar to prior research.[1,5] Secondary analyses evaluated the effect of the intervention on a six-dimension average MAIA score shown in factor analyses to represent one construct,[4,5] and on each of the eight MAIA dimensions. The MAIA assesses 5 overarching domains (numbered) with 8 scales (lettered; range 0 to 5) including the following: (1) Awareness of body sensations (i.e., (a)"Noticing", or awareness of uncomfortable, comfortable, and neutral body sensations); (2) Emotional Reaction and Attentional Response to Sensations (specifically (b) "Not Distracting": Tendency to ignore or distract oneself from sensations of pain or discomfort, and (c) "Not Worrying": Emotional distress or worry with sensations of pain or discomfort), (3) Capacity to Regulate Attention (i.e., ability to stay focused when facing numerous sensory stimuli competing for attention), including (d) "Attention Regulation" (i.e., Ability to sustain and control attention to body sensation); (4) Awareness of Mind-Body Integration (i.e., access to more developed levels of body awareness) (specifically (e) "Emotional Awareness": Awareness of the connection between body sensations and emotional states; (f) "Self-Regulation": Ability to regulate psychological distress by attention to body sensations; (g) "Body Listening": Actively listens to the body for insight); (5) Trusting Body Sensations (i.e., (h) "Trusting": Experiences one's body as safe and trustworthy).

Example MAIA questions for each of the 8 scales are as follows:

a) *Noticing scale:* "When I am tense I notice where the tension is located in my body."

© 2023 Loucks EB et al. *JAMA Network Open*.

b) *Not-Distracting scale:* “I distract myself from sensations of discomfort.”
c) *Not-Worrying scale:* “I can notice an unpleasant body sensation without worrying about it.”
d) *Attention Regulation scale:* “I can maintain awareness of my inner bodily sensations even when there is a lot going on around me.”
e) *Emotional Awareness scale:* “I notice how my body changes when I am angry.”
f) *Self-Regulation scale:* “When I am caught up in thoughts, I can calm my mind by focusing on my body/breathing.”
g) *Body Listening Scale:* “I listen to my body to inform me about what to do.”
h) *Trusting scale:* “I am at home in my body.”

Extent of adherence to the *Dietary Approaches to Stop Hypertension (DASH)-diet* was assessed using the Harvard 163-item 2007 Grid Food Frequency Questionnaire,[6] and coding of DASH diet adherence using methods developed by Folsom et al.[7] Specifically, the components of the DASH diet index were weighted and summed to calculate a single dietary concordance score (range 0-11). Quantitative criteria provided as part of the DASH diet were used to establish cut points for scoring the index items, detailed elsewhere.[7] For example, DASH dietary guidelines recommend four to five vegetable servings per day. Consequently, one point was assigned to participants consuming at least four daily vegetable servings, 0.5 points were assigned when vegetable intake approached the recommended level (two to three daily servings), and zero points were assigned when vegetable intake was much less than the recommended level (less than two daily servings).[7]

© 2023 Loucks EB et al. *JAMA Network Open*.

eMethods 3. Measurement of Covariates

Physical activity during the previous week was measured using the International Physical Activity Questionnaire (IPAQ) as total MET-minutes per week of physical activity, and time spent in sedentary sitting activities, with validation described elsewhere.[8]

Body mass index (kg/m^2) was calculated by weight and height measures obtained from participants wearing light clothing without shoes, using a calibrated stadiometer (SECA, Hamburg, Germany) and weighing scale (SECA, Model 22089, Hamburg, Germany) operated by trained technicians.

Alcohol consumption was assessed via a modified Centers for Disease Control and Prevention Behavioral Factor Surveillance System Questionnaire which has demonstrated concurrent validity with other nationally representative survey measures (e.g. NHIS, NHANES) in multiple studies evaluating alcohol consumption.[9]

Stress was assessed utilizing the 10-item Perceived Stress Scale (PSS-10) with established validity and reliability.[10]

Emotional eating was assessed using the Three Factor Eating Questionnaire Revised 21-item (TFEQ-R21).[11]

Mindfulness levels were assessed using the validated Five-Facet Mindfulness Questionnaire (FFMQ) a 39-item scale with established validity and reliability.[12]

© 2023 Loucks EB et al. *JAMA Network Open*.

eTable 1. Evaluation of Differential Missingness

Evaluation of whether missingness was differential by age, race/ethnicity, or education of participants.

Variable	Group	Baseline	10 Weeks	P for between-groups at 10 Weeks	6 Months	P for between-groups at 6 Months
Sample with DASH Diet Data, n	Control	100	84.0		79.0	
	MB-BP	100	83.0		79.0	
Age at Baseline, mean	Control	59.5	59.3	0.90	59.4	0.90
	MB-BP	59.5	59.5		59.2	
% Sample White Race/Ethnicity	Control	83.0	83.3	0.66	82.3	0.69
	MB-BP	79.0	80.7		79.8	
% Sample with College Education	Control	71.0	71.4	0.32	69.6	0.29
	MB-BP	74.0	77.1		76.0	

© 2023 Loucks EB et al. *JAMA Network Open*.

eTable 2. Mediation Analyses

Exploratory mediation analyses evaluating whether interoceptive awareness (assessed via Multidimensional Assessment of Interoceptive Awareness scale; MAIA) and mindfulness (assessed via Five Facet Mindfulness Questionnaire; FFMQ) mediate effects of MB-BP vs. control on the DASH diet score at 6 months follow-up in the 97 participants with low DASH diet adherence (scores <5.5) at baseline.

Mediator	Total Effect (p)	Natural Direct Effect (p)	Natural Indirect Effect (p)	Percent Mediated (p)
MAIA: 8-dimension average score	0.719 (0.005)	0.500 (0.11)	0.220 (0.25)	30.6% (0.28)
MAIA: 6-dimension average score[§]	0.721 (0.005)	0.526 (0.09)	0.195 (0.29)	27.0% (0.32)
MAIA Dimension: Noticing, score	0.718 (0.005)	0.588 (0.029)	0.130 (0.25)	18.1% (0.27)
MAIA Dimension: Not Distracting, score	0.778 (0.002)	0.772 (0.002)	0.006 (0.85)	0.8% (0.85)
MAIA Dimension: Not Worrying, score	0.772 (0.003)	0.729 (0.005)	0.043 (0.58)	5.6% (0.55)
MAIA Dimension: Attention Regulation, score	0.722 (0.005)	0.522 (0.07)	0.200 (0.19)	27.7% (0.23)
MAIA Dimension: Emotional Awareness, score	0.696 (0.007)	0.546 (0.09)	0.151 (0.46)	21.6% (0.47)
MAIA Dimension: Self-Regulation, score	0.706 (0.006)	0.518 (0.09)	0.188 (0.28)	26.7% (0.30)
MAIA Dimension: Body Listening, score	0.728 (0.004)	0.607 (0.06)	0.121 (0.56)	16.7% (0.56)
MAIA Dimension: Trusting, score	0.747 (0.003)	0.730 (0.008)	0.017 (0.87)	2.2% (0.87)
Mindfulness, FFMQ score	0.626 (0.02)	0.419 (0.17)	0.207 (0.19)	33.1% (0.23)

[§]6-dimension average excludes not distracting and not worrying dimensions

DASH, Dietary Approaches to Stop Hypertension; FFMQ, Five Facet Mindfulness Questionnaire; MAIA, Multidimensional Assessment of Interoceptive Awareness.

© 2023 Loucks EB et al. *JAMA Network Open*.

eReferences

1. Loucks EB, Nardi WR, Gutman R, et al. Mindfulness-Based Blood Pressure Reduction (MB-BP): Stage 1 single-arm clinical trial. *PLoS One*. 2019;14(11):e0223095. doi:10.1371/journal.pone.0223095
2. U.S. Department of Health and Human Services, U.S. Department of Agriculture. 2015–2020 Dietary Guidelines for Americans. 8th Edition. 2015;
3. Mehling WE, Price C, Daubenmier JJ, Acree M, Bartmess E, Stewart A. The Multidimensional Assessment of Interoceptive Awareness (MAIA). Research Support, N.I.H., Extramural. *PLoS One*. 2012;7(11):e48230. doi:10.1371/journal.pone.0048230
4. Ferentzi E, Olaru G, Geiger M, Vig L, Koteles F, Wilhelm O. Examining the Factor Structure and Validity of the Multidimensional Assessment of Interoceptive Awareness. *J Pers Assess*. Sep-Oct 2021;103(5):675-684. doi:10.1080/00223891.2020.1813147
5. Mehling WE, Todd J, Schuman-Olivier Z. Multidimensional Assessment of Interoceptive Awareness, Version 2 (MAIA-2). In: Medvedev ON, Krägeloh CU, Siegert RJ, Singh NN, eds. *Handbook of Assessment in Mindfulness Research*. Springer, Cham; 2022.
6. Willett WC, Sampson L, Stampfer MJ, et al. Reproducibility and validity of a semiquantitative food frequency questionnaire. *American journal of epidemiology*. Jul 1985;122(1):51-65.
7. Folsom AR, Parker ED, Harnack LJ. Degree of concordance with DASH diet guidelines and incidence of hypertension and fatal cardiovascular disease. *Am J Hypertens*. Mar 2007;20(3):225-32. doi:10.1016/j.amjhyper.2006.09.003
8. Craig CL, Marshall AL, Sjostrom M, et al. International physical activity questionnaire: 12-country reliability and validity. *Med Sci Sports Exerc*. Aug 2003;35(8):1381-95. doi:10.1249/01.MSS.0000078924.61453.FB
9. Pierannunzi C. A systematic review of publications assessing reliability and validity of the Behavioral Risk Factor Surveillance System (BRFSS), 2004-2011. *BMC medical research methodology*. 2013;13(1):1 - EOA.
10. Cohen S, Kamarck T, Mermelstein R. A global measure of perceived stress. *J Health Soc Behav*. Dec 1983;24(4):385-96.
11. Cappelleri JC, Bushmakin AG, Gerber RA, et al. Psychometric analysis of the Three-Factor Eating Questionnaire-R21: results from a large diverse sample of obese and non-obese participants. *Int J Obes (Lond)*. Jun 2009;33(6):611-20. doi:10.1038/ijo.2009.74
12. Baer RA, Smith GT, Hopkins J, Krietemeyer J, Toney L. Using self-report assessment methods to explore facets of mindfulness. *Assessment*. Mar 2006;13(1):27-45. doi:Doi 10.1177/1073191105283504

© 2023 Loucks EB et al. *JAMA Network Open*.
