# Supplementary material for: Adapted Mindfulness Training for Interoception and Adherence to the DASH Diet: A Phase 2 Randomized Clinical Trial
Source: JAMA Netw Open. 2023 Nov 2;6(11):e2339243. doi: 10.1001/jamanetworkopen.2023.39243 (PMC10623198; doi:10.1001/jamanetworkopen.2023.39243)
Supplement: Supplement 3. — Data Sharing Statement [file jamanetwopen-e2339243-s003.pdf]

Data Sharing Statement

Loucks. Adapted Mindfulness Training for Interoception and Adherence to the Dietary Approaches to Stop Hypertension (DASH) Diet. *JAMA Netw Open*. Published November 02, 2023. doi:10.1001/jamanetworkopen.2023.39243

Data
Data available: Yes
Data types: Deidentified participant data
How to access data: The data for this study will be available at the Open Science Framework and can be accessed at doi.org/10.17605/OSF.IO/86UCD.
When available: With publication

Supporting Documents
Document types: None

Additional Information
Who can access the data: In order to minimize the possibility of unintentionally sharing information that can be used to re-identify private information, a subset of the data generated for this study will be available at the Open Science Framework and can be accessed at doi.org/10.17605/OSF.IO/86UCD.
Types of analyses: Any purpose.
Mechanisms of data availability: The data generated for this study will be available at the Open Science Framework and can be accessed at doi.org/10.17605/OSF.IO/86UCD. If further data are desired, interested parties can contact the lead author to request those data which will be considered in alignment with Brown University's IRB guidelines for protection of human subject.
